# Automated assembly of oligosaccharides containing multiple *cis-*glycosidic linkages

**DOI:** 10.1038/ncomms12482

**Published:** 2016-09-01

**Authors:** Heung Sik Hahm, Mattan Hurevich, Peter H Seeberger

**Affiliations:** 1 Department of Bimolecular System, Max Planck Institute of Colloids and Interfaces, Am Mühlenberg 1, Potsdam 14424, Germany; 2 Institute of Chemistry and Biochemistry, Freie Universität Berlin, Arnimallee 22, Berlin 14195, Germany

## Abstract

Automated glycan assembly (AGA) has advanced from a concept to a commercial technology that rapidly provides access to diverse oligosaccharide chains as long as 30-mers. To date, AGA was mainly employed to incorporate *trans*-glycosidic linkages, where C2 participating protecting groups ensure stereoselective couplings. Stereocontrol during the installation of *cis*-glycosidic linkages cannot rely on C2-participation and anomeric mixtures are typically formed. Here, we demonstrate that oligosaccharides containing multiple *cis-*glycosidic linkages can be prepared efficiently by AGA using monosaccharide building blocks equipped with remote participating protecting groups. The concept is illustrated by the automated syntheses of biologically relevant oligosaccharides bearing various *cis*-galactosidic and *cis*-glucosidic linkages. This work provides further proof that AGA facilitates the synthesis of complex oligosaccharides with multiple *cis-*linkages and other biologically important oligosaccharides.

## Abstract (web summary)

Automated glycan assembly has proven a powerful method to rapidly synthesize large oligosaccharides, though stereoselective *cis*-glycosylation remains a challenge. Here, the authors demonstrate a system to selectively incorporate multiple *cis*-glycosidic linkages by use of remote participating groups.

## Graphical abstract

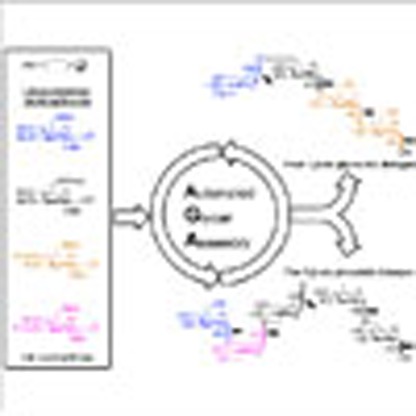

Carbohydrates are the most abundant biomolecules on earth and serve many functions, including structure, nutrition and information transfer^1^. The structural diversity and complexity of natural glycans, combined with the lack of amplification and expression methods, renders their isolation difficult and often impossible. The synthesis of complex glycans is time consuming and is performed by specialists. Automated glycan assembly (AGA) on solid support^2^,^3^ has accelerated the procurement of defined oligosaccharides for use as molecular tools on glycan arrays^4^ and as part of vaccines^5^. Using automated synthesis, oligosaccharides as large as 30-mers^6^ that represent different subclasses of glycans, including glycolipids^7^ and glycosaminoglycans^8^, are now accessible. AGA utilizes monomeric building blocks that are combined on a synthesizer executing a set of preprogrammed commands that are combined into modules^9^. To date, a set of monosaccharide building blocks has been identified for the efficient and reliable incorporation of *trans-*glycosidic linkages during AGA. These monomeric building blocks commonly take advantage of a C2 neighbouring group participation to ensure exclusive *trans*-glycosylations. Most oligosaccharides assembled by AGA to date contained either exclusively or predominantly *trans*-glycosidic linkages aside from few exceptions^10^,^11^. The selective formation of a 1,2-*cis*-glycosylic linkage during AGA of the tumour-associated hexasaccharide antigen Globo-H^10^, as well as a β-(1,4)-mannuronic acid alginate 12-mer containing 1,2-*cis*-mannosides^11^ relied on C2 non-participating protecting groups and benefited from leaving group effects^10^ and conformational influences^11^.

Stereoselective formation of 1,2-*cis*-glycosidic linkages still remains challenging^12^,^13^,^14^,^15^,^16^, because non-participating C2 protecting groups generally lead to mixtures of stereoisomers that have to be separated at the end of the synthesis (Fig. 1a). Various strategies for the stereoselective synthesis of 1,2-*cis-*glycosides have been described (Fig. 1). Intramolecular aglycon delivery (IAD)^12^, where the nucleophile is transferred from the adjacent C2 carbon to the anomeric position, is technically challenging when more than one *cis*-linkage is to be created. Hydrogen-bond-mediated aglycan delivery (HAD) method showed that installing picolinyl and picoloyl protecting groups on the C6 position is powerful for the synthesis of *cis*-glucosidic linkage, but fails for *cis*-galactosidic linkages^13^, as none of the hydroxyl groups at C3, C4 or C6 faces towards the bottom of the ring. Chiral auxiliaries at C2 provide selectivity^14^, but require two additional steps to be removed. Cleavage of the chiral auxiliary may result in a loss of benzyl ethers during solid-phase synthesis^15^. Additives can improve stereoselectivity by forming a less reactive intermediate *in situ* 16 but are hard to use during automated syntheses. Remote participation by protecting groups placed at the C3, C4 and/or C6 positions of glucose (Glc) and galactose (Gal) building blocks can control the stereoselectivity of glycosylations^17^,^18^,^19^. Building blocks containing common remote participating groups are attractive for automated synthesis as they fit the coupling–deprotection scheme and require no additional manipulations.

Here, we demonstrate that AGA of oligosaccharides containing 1,2-*cis*-glycosidic linkages is feasible when monosaccharide building blocks containing common remote participating groups are used. This work proves that identification and incorporation of reliable building blocks into AGA protocols provides accessibility to biologically relevant oligosaccharides containing *cis-*linkages.

## Results

### Selection of oligosaccharides bearing 1,2-*cis* glycosidic linkages

**Figure 2:**
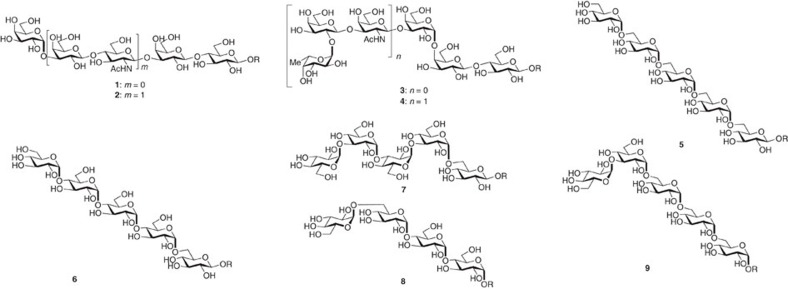
Oligosaccharides containing different *cis*-glycosidic linkages were assembled by automated synthesis. Oligosaccharides **1–4** contacting 1,2-*cis*-galactosidic linkages and oligosaccharides **5**–**9** containing either single or multiple 1,2-*cis*-glucosidic linkages. OR=O(CH_2_)_5_NH_2_.

To expand the scope of AGA to the stereoselective installation of *cis*-glycosidic linkages, we focused on *cis*-glucosides and *cis*-galactosides as the most prevalent 1,2-*cis*-linkages in mammalian and bacterial glycomes^20^,^21^. Oligosaccharides (**1**–**4)** bearing α-galactosidic linkages and α-glucans examples (**5**–**9**) were selected as targets to develop automated methods for stereoselective *cis*-glycosidic bond formation (Fig. 2).

### AGA of oligosaccharides containing 1,2-*cis*-glactosidic linkage

Since the stereo- and regiochemical information for glycosidic bond formation mainly resides in the building blocks^9^, the synthetic considerations focused initially on the identification of monomers containing remote participating groups. α-Gal trisaccharide epitope **1** (ref. 22) served as a model system to optimize the formation of α-(1,3) galactosides. The influence of remote participation on the stereochemical outcome of AGA couplings was explored using seven thiogalactoside building blocks (**13a–13g**) containing acetyl or benzoyl esters (Ac, Bz). Solid-support-bound disaccharide **14** was assembled using building blocks **11** and **12** on a polystyrene resin equipped with a photocleavable linker **10** (ref. 8). Disaccharide **14** served as the acceptor in glycosylations employing monomers **13a–13g**. Incorporation of each building block was achieved by the addition of twice five equivalents monomer that was activated at activation temperature *T*_a_ (−40 °C) for 5 min (*t*_1_) by the addition of a *N*-iodosuccinimide (NIS)/Trifluoromethanesulfonic acid (TfOH) activator solution, followed by incubation at the incubation temperature *T*_i_ (−20 °C) for 25 min (*t*_2_). Following ultraviolet cleavage from the polystyrene resin, trisaccharides (**15a–15g**) were analysed by normal-phase high-performance liquid chromatography (NP-HPLC) (Table 1) to determine conversion and stereoselectivity of the glycosylation reaction.

Galactose building block **13a** (ref. 23), protected only with non-participating benzyl ether groups, relies exclusively on the anomeric effect to drive α-galactoside formation^23^ and produced mainly the desired anomer **15a** (ref. 24) (*α*:*β*=13.8:1) (entry 1). Thiogalactosides **13b** (ref. 25) and **13c** bearing C6 acetyl or benzoyl esters resulted in lower selectivity (*α*:*β*=6.4−5.8:1) (entries 2 and 3). These results are consistent with reports that C6 esters decrease the α-selectivity of galactose^18^. The presence of a C4-acetyl or benzoyl ester in thiogalactosides **13d** and **13e** drastically improved the α-selectivity (26.5−26.2:1). Expanding on the C4-ester effect, a second ester was placed on thiogalactose building blocks. Glycosylation of **14** with C3-, C4-*bis*-acetylated thiogalactose **13f** proceeded with drastically improved selectivity (39.5:1) (Table 1, entry 6). Building block **13g**, carrying C4 and C6 esters proceeded with complete stereoselectivity but significant amounts of the disaccharide deletion sequence remained after the coupling (entry 7). This study proved that the 1,2-*cis*-galactosidic linkage can be installed by using building blocks that take advantage of remote participation groups but require no additives or chiral auxiliaries.

**Figure 3:**
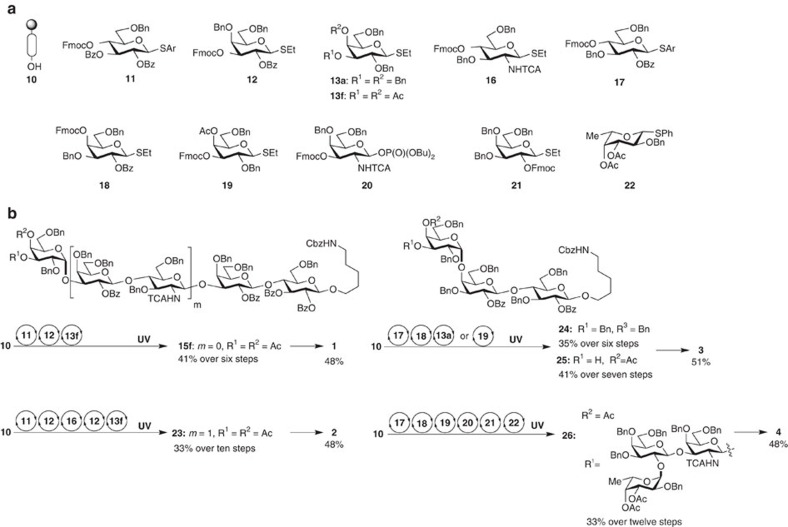
AGA of oligosaccharides containing 1,2-*cis*-glactosidic linkage. (**a**) Resin functionalized with the photolabile linker (**10**) and monosaccharide building blocks. (**b**) Automated assembly and post-automation steps yield conjugation-ready oligosaccharides **1**–**4**. For details, see Supplementary Tables 3–5.

With optimized building blocks in hand (Fig. 3a), the α-Gal epitope pentasaccharide **2** was prepared by AGA (Fig. 3b). All synthetic manipulations were executed on an automated oligosaccharide synthesizer working through preprogrammed steps that were combined into modules for each synthetic transformation. An initial acidic trimethylsilyl trifluoromethanesulfonate (TMSOTf) wash neutralizes basic residues that accumulate during dimethylformamide (DMF) washes and removes any water present in the synthesizer. Glycosylation with twice five equivalents monomer was followed by fluorenylmethyloxycarbonyl (Fmoc) removal by treatment with a solution of triethylamine (TEA) in DMF (v/v, 1/4). The glycosylation efficiency was estimated by quantitating the ultraviolet absorption of the released dibenzofulvene. Following automated assembly of the fully protected oligosaccharide targets, cleavage from the resin by ultraviolet irradiation was carried out using a continuous-flow photoreactor^8^. Purification by preparative NP-HPLC provided pentasaccharide **15f** (41% based on the resin loading over ten steps on resin) and **23** (33% over ten steps on resin). The conjugation-ready α-Gal epitope trisaccharide **1** (48**%** over two steps) and pentasaccharide **2** (48**%**) were purified by reverse-phase HPLC following deprotection of **15f** and **23** by methanolysis and hydrogenolysis.

Building blocks carrying remote participation groups were employed in AGA of Globo-series oligosaccharides that contain a Gal-α-(1,4)-Gal-β-(1,4)-Glc common trisaccharide core (**3**) (refs 26, 27). Globo-H (**4**), a hexasaccharide vaccine candidate currently in advanced clinical trials^28^,^29^ contains the core trisaccharide **3**. Thiogalactoside building block **19** was designed based on thiogalactoside **13f** to allow for C3 elongation *en route* to Globo-H and to benefit from remote participation. The influence of a remote C4 participation group present in monomer **19** was studied in the context of the AGA of trisaccharides **24** (ref. 24) and **25.** Trisaccharide **25** using optimized building block **19** was obtained in 41% yield, and with excellent selectivity (*α*:*β*=19:1), while **24** using perbenzylated thiogalactoside donor **13a** was isolated in 35% yield and moderate selectivity (*α*:*β*=4.3:1). This drastic improvement of stereoselective 1,2-*cis-*galactosidic bond formation emphasizes the importance of the remote participating group on the C4 for the efficient synthesis of complex oligosaccharides containing *cis*-galactoside(s) linkage using AGA. Similarly Globo-H hexasaccharide **4** was prepared by AGA using building blocks **17**–**22**. Fully protected oligosaccharides **25** and **26** were deprotected to furnish **3 (**51% yield) and **4** (42%) respectively. The stereoselectivity of α-galactoside formation can dramatically benefit from the use of ‘approved' building blocks with remote participation.

### AGA of α-glucans bearing multiple 1,2-*cis*-glucosidic linkages

The use of remote participation is not limited to the formation of *cis*-galactosidic linkages but can be generally employed as illustrated for 1,2-*cis*-glucosidic linkages^19^. Eight thioglucoside building blocks (**29a**–**29h**) exhibiting various protecting group patterns were synthesized to explore the influence of remote participation. AGA of disaccharides **30a**–**30j** was achieved using thioglycosides **17, 27** and **28,** and building blocks **29a**–**29h** before photocleavage from the polystyrene resin yielded disaccharides **30a**–**30j** that were analysed by HPLC (Table 2). Glucose building block **29h** with remote participating acetyl groups in the C3 and C6 positions in analogy to the galactose series, resulted in the best selectivity and further benefited from the addition of diethyl ether to promote the desired α-glucoside formation (Table 2).

**Figure 4:**
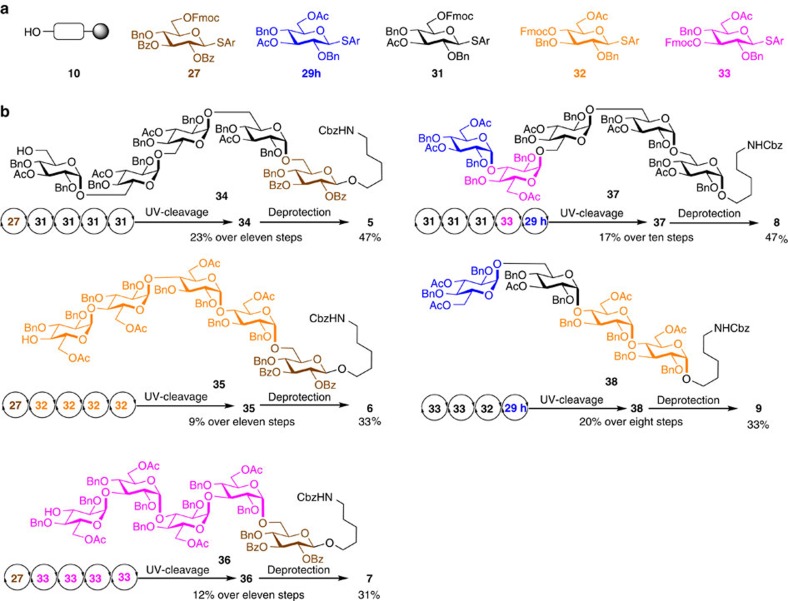
AGA of α-glucans bearing multiple 1,2-*cis*-glucosidic linkages. (**a**) Monosaccharide building blocks. (**b**) AGA of conjugation-ready oligosaccharides **5**–**9**. For details, see Supplementary Tables 8–10.

With reliable glucose building blocks and AGA protocols for the incorporation of α-glucosides in place, a series of α-glucans were prepared. The initial target, pentasaccharide **5** containing four consecutive α-(1→6)-glycosidic linkages^15^ was selected as proof-of-principle, since it had been prepared previously via the chiral auxiliary approach. AGA of pentasaccharide **5** was achieved by addition of the thioglycoside **27** to the support-bound linker **10** to form the initial β-glucoside linkage. Four successive glycosylation cycles using thioglycoside **31** introduced four consecutive α-1,2-*cis-*glucosidic linkages. Ultraviolet-induced cleavage of the fully protected oligosaccharide from the resin yielded protected pentasaccharide **34** in 23% over 11 steps on resin using NP-HPLC (Fig. 4b).

Pentasaccharide **6**, containing multiple α-(1→4) linkages, and **7** with multiple α-(1→3) glucosides were assembled using the corresponding building blocks (Fig. 4). The lower reactivity of the C4-hydroxyl group as glycosyl acceptor was compensated by prolonged glycosylation times (*t*_2_=50 min) during AGA of **35** using building blocks **27** and **32** (see Supplementary Table 8). Cleavage from the resin and preparative NP-HPLC yielded 9% of **35** over 11 steps. Using a similar AGA process, pentasaccharide **36** was obtained in 12% yield over 11 steps. The syntheses of **35** and **36** illustrated that glycosylations of secondary C3 or C4 hydroxyl groups are less efficient and less stereoselective than those involving primary C6 hydroxyl groups as nucleophiles. Such differences in glycosylation efficiency are well known for solution phase strategies. It is anticipated that the glycosylation modules (for example, number of glycosylation, reaction time or temperature) upon AGA will improve glycosylation efficiency and seteroselectivity. Removal of the protective groups from pentasaccharides **34–36** yielded conjugation-ready pentasaccharides **5 (**47% yield), **6** (33%) and **7 (**31%) following reverse-phase HPLC.

Two naturally occurring oligosaccharide fragments bearing multiple 1,2-*cis*-glucosidic linkages were assembled using the building blocks and protocols established above. The immune-modulatory pentasaccharide **8** (ref. 30), containing α-(1→3) and α-(1→6) glucosidic linkages, and tetrasaccharides **9** (ref. 31), known for activating toll-like receptor containing α-(1→4) and α-(1→6) glucosides, were synthesized. Deprotection of oligosaccharide **37** that was assembled in 17% yield over 10 steps and **38** yielding 20% over eight steps, furnished oligosaccharides **8 (**47% yield) and **9** (33%).

In conclusion, we demonstrate that building blocks bearing remote participating groups are effective in assembling oligosaccharides containing *cis*-glucosidic and *cis*-galactosidic linkages. Nine oligosaccharides (**1**–**9**) were assembled to illustrate the power of the AGA approach. Standardized synthesis as well as deprotection and purification protocols enabled us to procure conjugation-ready molecules. The reliable incorporation of *cis*-gluco- and *cis*-galactosides into oligosaccharides by automated synthesis complements earlier successes in installing *trans*-glycosides, to render AGA the method of choice for the procurement of complex glycans.

## Methods

### General

The synthesis of characterization of monomer building blocks (**11**, **12**, **13a**–**13g**, **16**–**22**, **27**, **28**, **29a**–**29h** and **31**–**33**) and the synthetic protocols including liquid chromatography (LC)–mass spectrometry (MS) chromatograms for AGA of oligosaccharides (**1**–**9**, **15a**–**15g**, **23**–**26**, **30a**–**30j** and **34**–**38**) are provided in Supplementary Methods. For ^1^H, ^13^C and two-dimensional (2D) NMR spectra of the compounds in the article, see Supplementary Figs 1–118. For the reaction conditions on AGA, see Supplementary Tables 1,3,6 and 8. For yields for oligosaccharides, see Supplementary Tables 2,4,5,7,9 and 10.

### Pre-automation steps

Synthesis of building blocks and the linker-bound resin are described in Supplementary Methods. For preparation of building block solutions, reagent solutions and programs for AGA, see Supplementary Methods.

### AGA

The automated synthesizer^8^ executes a series of commands that are combined into modules to achieve specific transformations (see Supplementary Methods and Supplementary Tables).

### Module 1: acid wash

The resin was washed with DMF, tetrahydrofuran (THF) and dichloromethane (DCM) (three times each with 2 ml for 15 s). The resin was swollen in 2 ml DCM, and the temperature of the reaction vessel was adjusted to −20 °C. For an acidic wash, 0.500 ml of the TMSOTf in DCM was delivered to the reaction vessel. After 1 min, the solution was drained. The resin was swollen in 2 ml DCM and the temperature of the reaction vessel was adjusted to the activation temperature (*T*_a_).

### Module 2: glycosylation using thioglycosides

During temperature adjustment, the DCM in the reaction vessel was drained and a solution of building block (5.0 equiv. in 1.0 ml DCM) was delivered to the reaction vessel. After the activation temperature (*T*_a_) was reached, the reaction was started by addition of 1.0 ml of NIS solution (5.5 equiv. in 1.0 ml) and TfOH (0.2 equiv. in 1.0 ml) in DCM and dioxane (v/v, 9/1). The glycosylation mixture was activated for an activation time (*t*_1_=5 min) at *T*_a_, linearly ramped to incubation temperature (*T*_i_), and finally incubated for an additional incubation time (*t*_2_) minutes at *T*_i_. Then, the reaction solution was drained and the resin was washed with DCM (six times with 2 ml for 15 s). This procedure was repeated twice. The values of *T*_a_, *t*_1_, *T*_i_ and *t*_2_ are shown in Supplementary Tables.

### Module 3: fmoc deprotection

The resin was washed with DMF (six times with 2 ml for 15 s), swollen in 2 ml DMF and the temperature of the reaction vessel was adjusted to 25 °C. The DMF was drained and 3.5 ml of a solution of TEA in DMF was delivered to the reaction vessel. After 5 min, the reaction solution was collected in the fraction collector of the oligosaccharide synthesizer. This procedure was repeated twice.

### Cleavage and purification

*Resin cleavage*. The FEP tubing of the photoreactor^8^ was washed with 20 ml DCM at a flow rate of 5 ml min^−1^ to prepare the reactor. For cleavage, the resin was slowly injected into the reactor using a disposable syringe (20 ml) and pushed through the tubing with 18 ml DCM (flow rate: 600 μl min^−1^). The tubing is washed with 20 ml DCM (flow rate: 2 ml min^−1^) to remove any remaining resin. The suspension leaving the reactor was passed through a filter to remove the resin. The system was re-equilibrated by washing the tubes with 20 ml DCM at a flow rate of 5 ml min^−1^. The cleavage procedure was performed twice and the resulting solution was evaporated before the crude material was analysed by matrix-assisted laser desorption/ionization–time of flight–MS (MALDI–TOF–MS), NMR and HPLC.

*Analytical HPLC*. The crude material was analysed by HPLC (column: Luna 5μ Sil 100A, (260 × 4.60 mm); flow rate: 1 ml min^−1^; eluents: hexane/ethyl acetate; gradient: 20% (5 min), 60% (in 40 min), 100% (in 5 min); and detection: evaporative light scattering detector (ELSD)).

*Preparative HPLC*. The crude mixture was dissolved in a minimum volume of DCM and 0.9 ml of 20% hexane in ethyl acetate and injected for purification using semi-preparative HPLC (column: Luna 5μ Sil (260 × 10 mm); flow rate: 5 ml min^−1^; eluents: 5% DCM in hexane/5% DCM in ethyl acetate; gradient: 20% (5 min), 60% (in 40 min), 100% (in 5 min); and detection: ELSD) to afford the fully protected target oligosaccharides.

*Deprotection conditions*. To a solution of the fully protected oligosaccharide in MeOH (5 ml) was added 58 μl of a 0.5 M NaOMe solution (0.25 equiv. per acetyl of benzoyl group) at 40 °C. The mixture was stirred until thin-layer chromatography (TLC) analysis indicated complete deprotection, then neutralized with 200 mg Amberite (400 mg per 100 μl of NaOMe solution). The amberlite was filtered off and the crude filtrate was evaporated and re-dissolved in MeOH, EtOAc and AcOH (v/v/v=5:0.5:0.2) before 5% Pd/C (W/V) was added, purged first with argon and then with hydrogen gas (H_2_), and was left to stir overnight at room temperature. The reaction mixture was filtered through a syringe filter with 20 ml of a water/methanol mixture (9:1) and the combined solution was evaporated to provide the crude product.

### Analysis of oligosaccharide products

*Analytical HPLC*. The crude material was analysed by HPLC (column: Hypercarb, (150 × 4.60 mm); flow rate: 0.8 ml min^−1^; eluents: 0.1% FA in acetonitrile/0.1% FA in water; gradient: 0% (10 min), 30% (in 30 min), 100% (in 5 min); and detection: ELSD).

*Preparative HPLC*. The crude product was purified by preparative HPLC (column: Hypercarb, (150 × 10.00 mm); flow rate: 3.6 ml min^−1^; eluents: 0.1% FA in acetonitrile/0.1% FA in water; gradient: 0% (10 min), 30% (in 30 min), 100% (in 5 min); and detection: ELSD) to afford the oligosaccharide product.

### Data availability

The raw data used to generate the figures and tables in this manuscript are available from the corresponding author on request.

## Additional information

**How to cite this article:** Hahm, H. S. *et al*. Automated assembly of oligosaccharides containing multiple *cis-*glycosidic linkages. *Nat. Commun.* 7:12482 doi: 10.1038/ncomms12482 (2016).

## Supplementary Material

Supplementary Information Supplementary Figures 1-118, Supplementary Tables 1-10, Supplementary Methods and Supplementary References

## Figures and Tables

**Figure 1:**
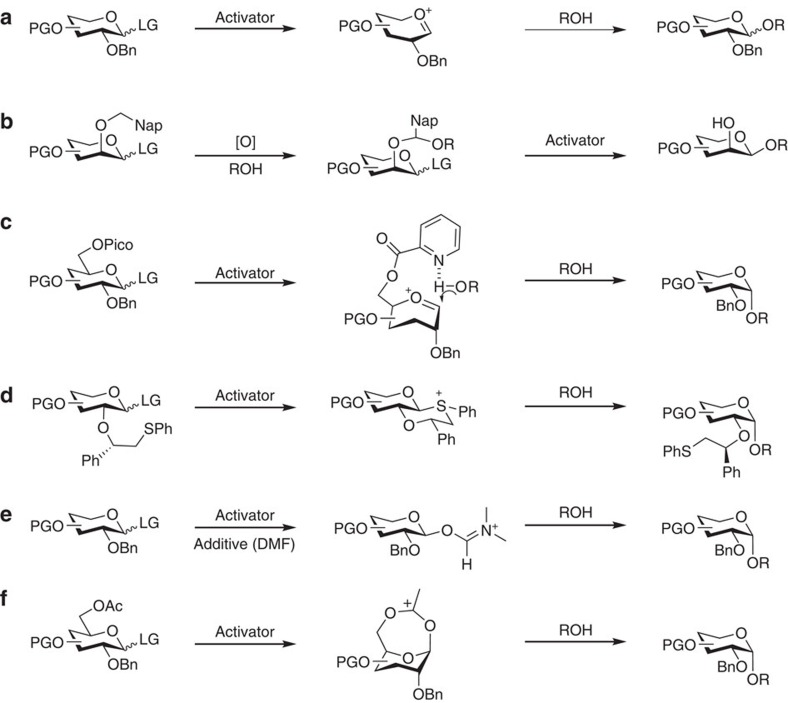
Various methods used for the formation of 1,2-*cis-*glycosides. (**a**) A non-participating protecting group at C2 results in a mixture of anomers. (**b**) Intramolecular aglycon delivery with a C2 2-naphthylmethyl (NAP)-ether produces predominantly the β-anomer. (**c**) Hydrogen-bond-mediated aglycan delivery using picolinyl and picoloyl protecting groups at the C6 position of glucose provide high selectivity. (**d**) Chiral auxiliaries ensure complete selectivity. (**e**) Additive (or solvent)-modulation results in predominant to exclusive formation of 1,2-*cis-*glycosides. (**f**) Remote participating groups result in good selectivities. Ac, acetate; Bn, benzyl; DMF, dimethylformamide; LG, leaving group; Nap, naphthyl; [O], oxidation; PG, protecting group; Pico, picoloyl; ROH, acceptor; Ph, phenyl.

**Table 1:**
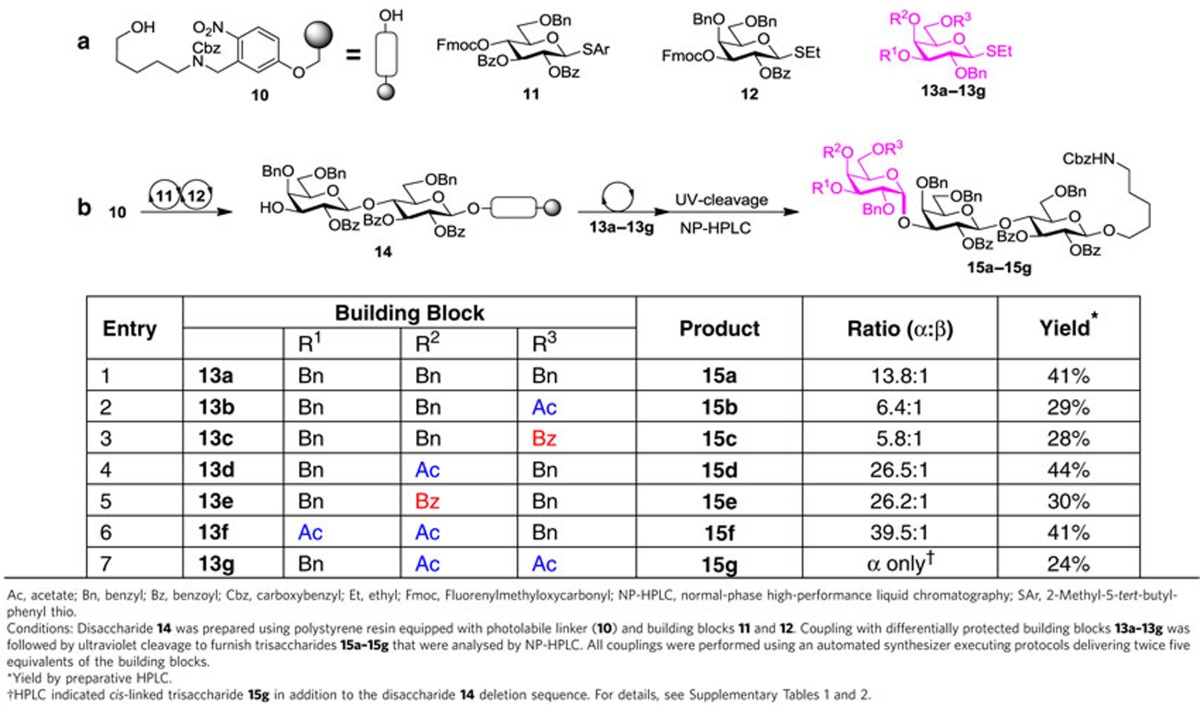
Identification of building blocks for installation of α-galactosidic linkages by automated synthesis.

**Table 2:**
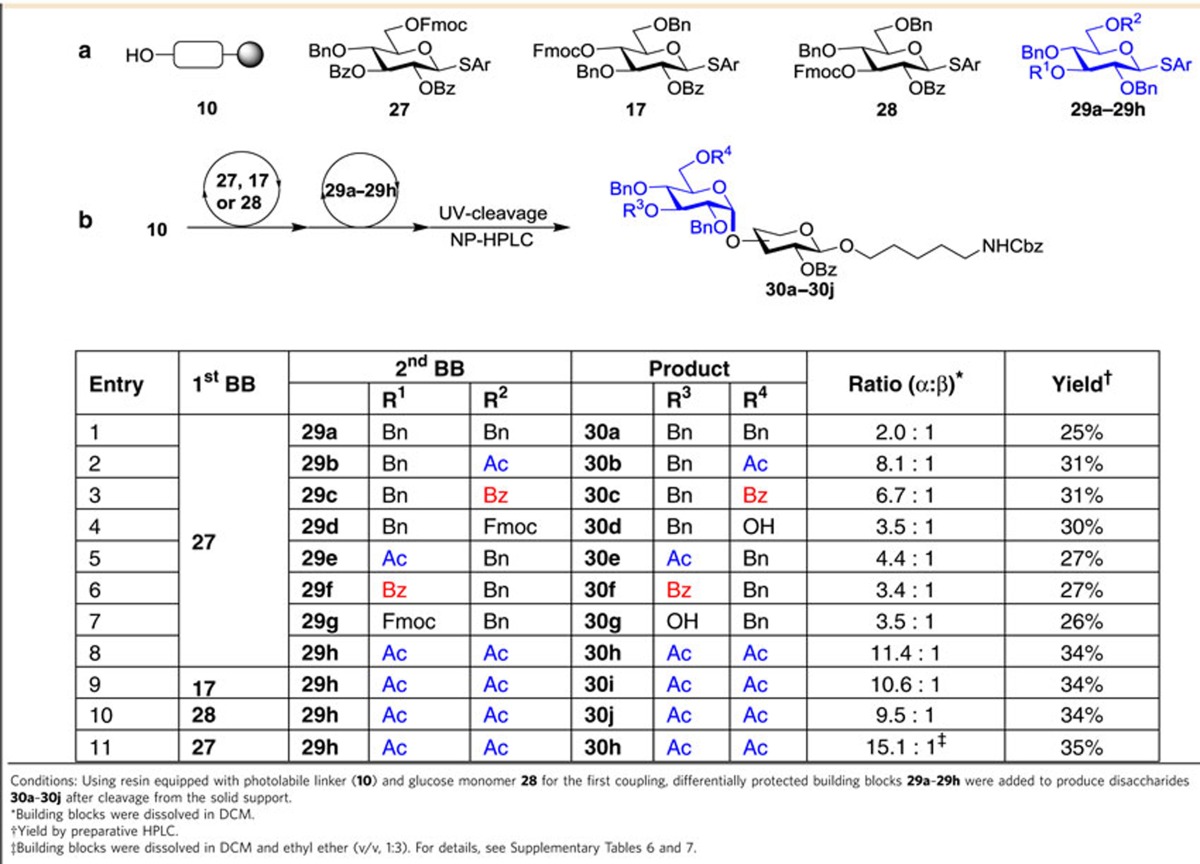
Identification of building blocks for α-glucoside installation by automated synthesis.
