# Supplementary material for: Automated assembly of oligosaccharides containing multiple cis-glycosidic linkages
Source: Nat Commun. 2016 Sep 1;7:12482. doi: 10.1038/ncomms12482 (PMC5025749; doi:10.1038/ncomms12482)
Supplement: Supplementary Information — Supplementary Figures 1-118, Supplementary Tables 1-10, Supplementary Methods and Supplementary References [file ncomms12482-s1.pdf]

^{1}H NMR, 600 MHz, D_2O

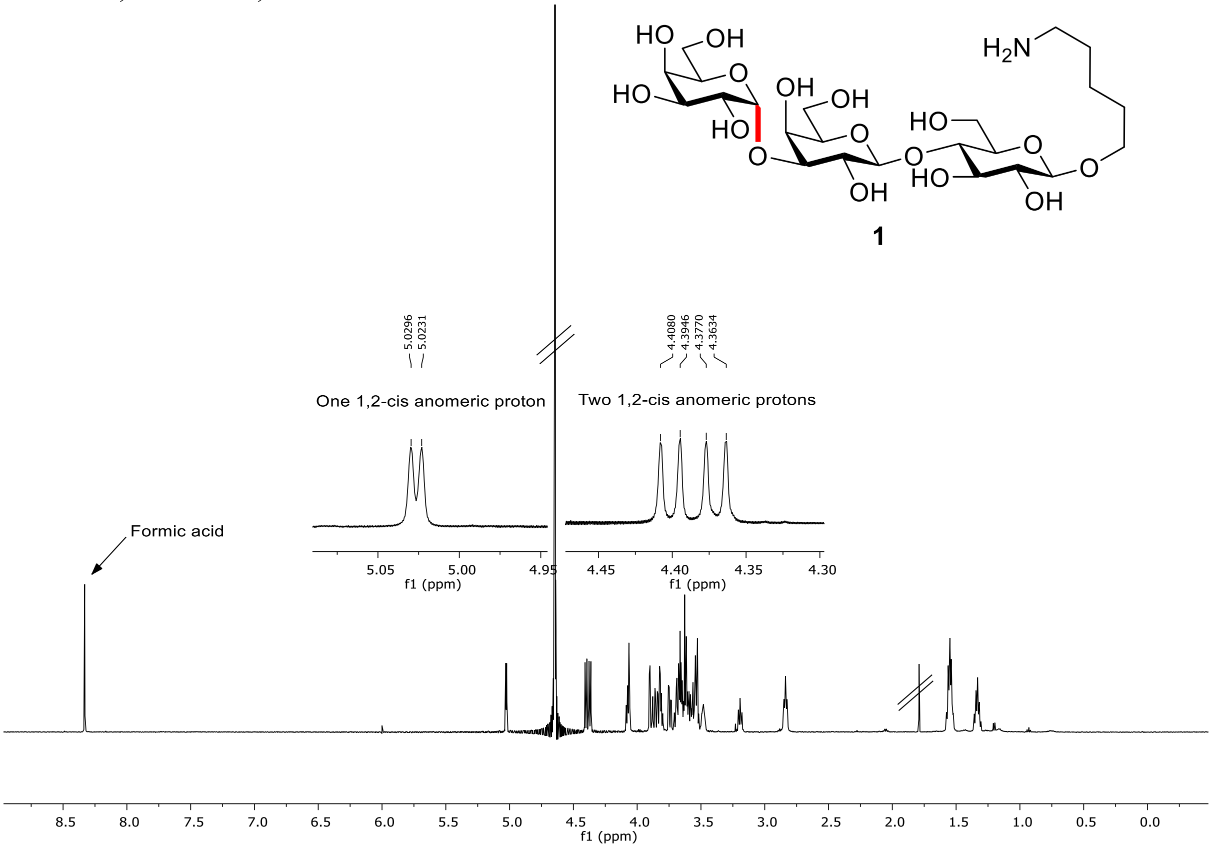

^{13}C NMR, 150 MHz, D_2O

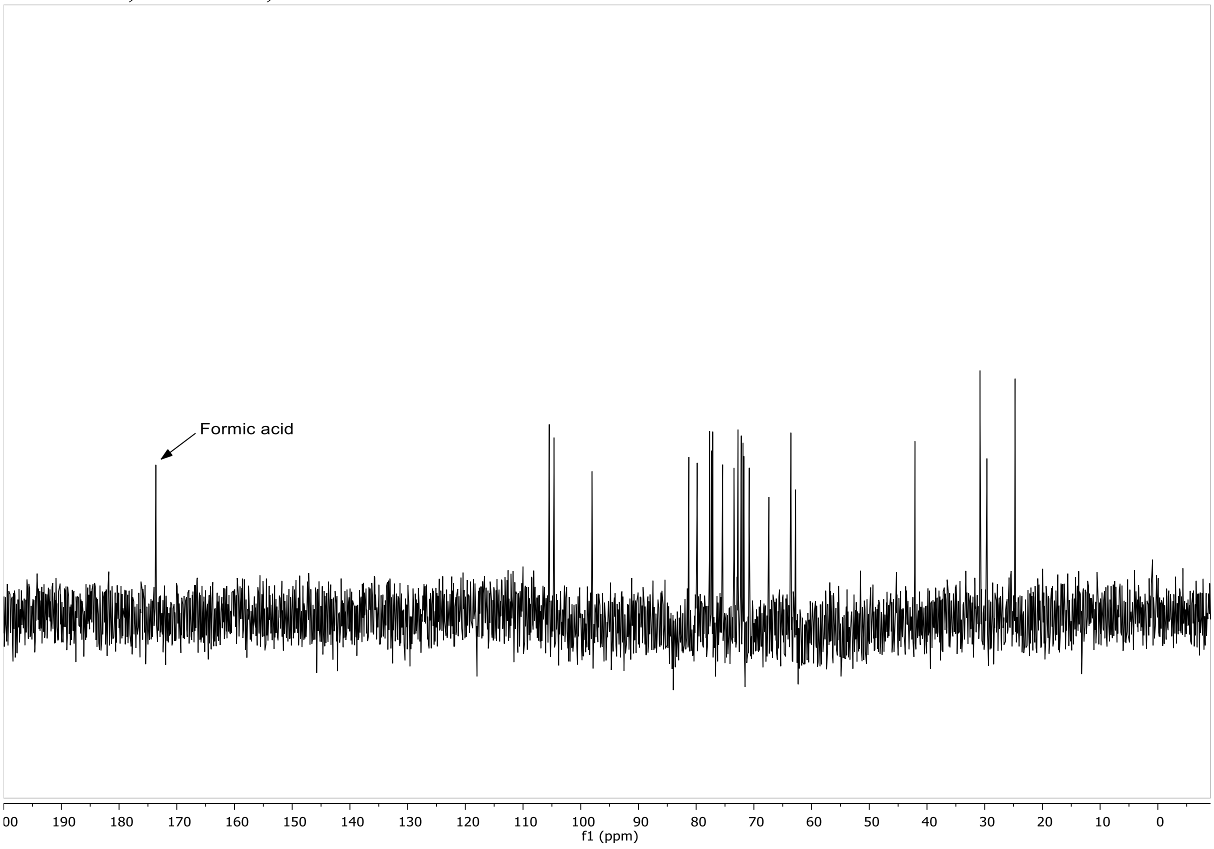

Supplementary Figure 1| 1D NMR spectra of 1

^{1}H-COSY NMR, 600 MHz, D_2O

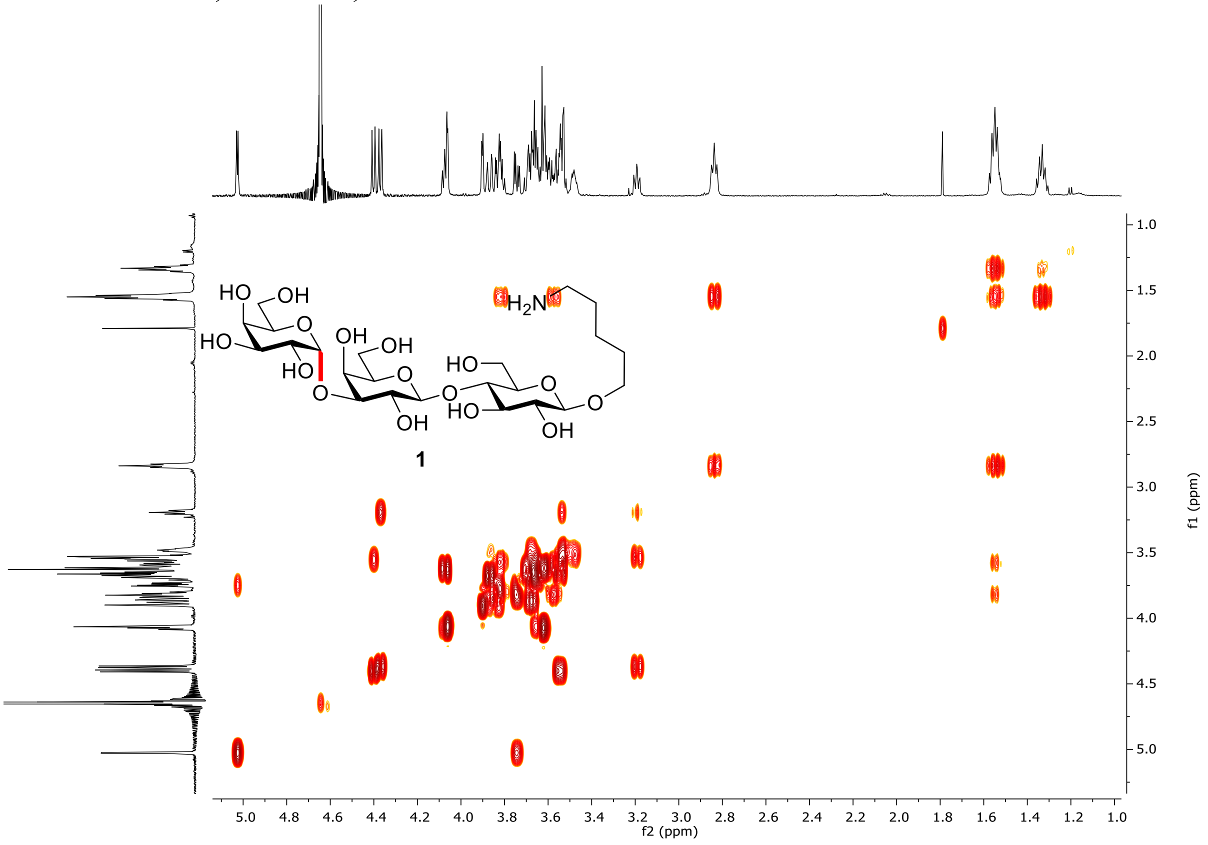

H-^{13}C-HSQC NMR, 600 MHz, D_2O

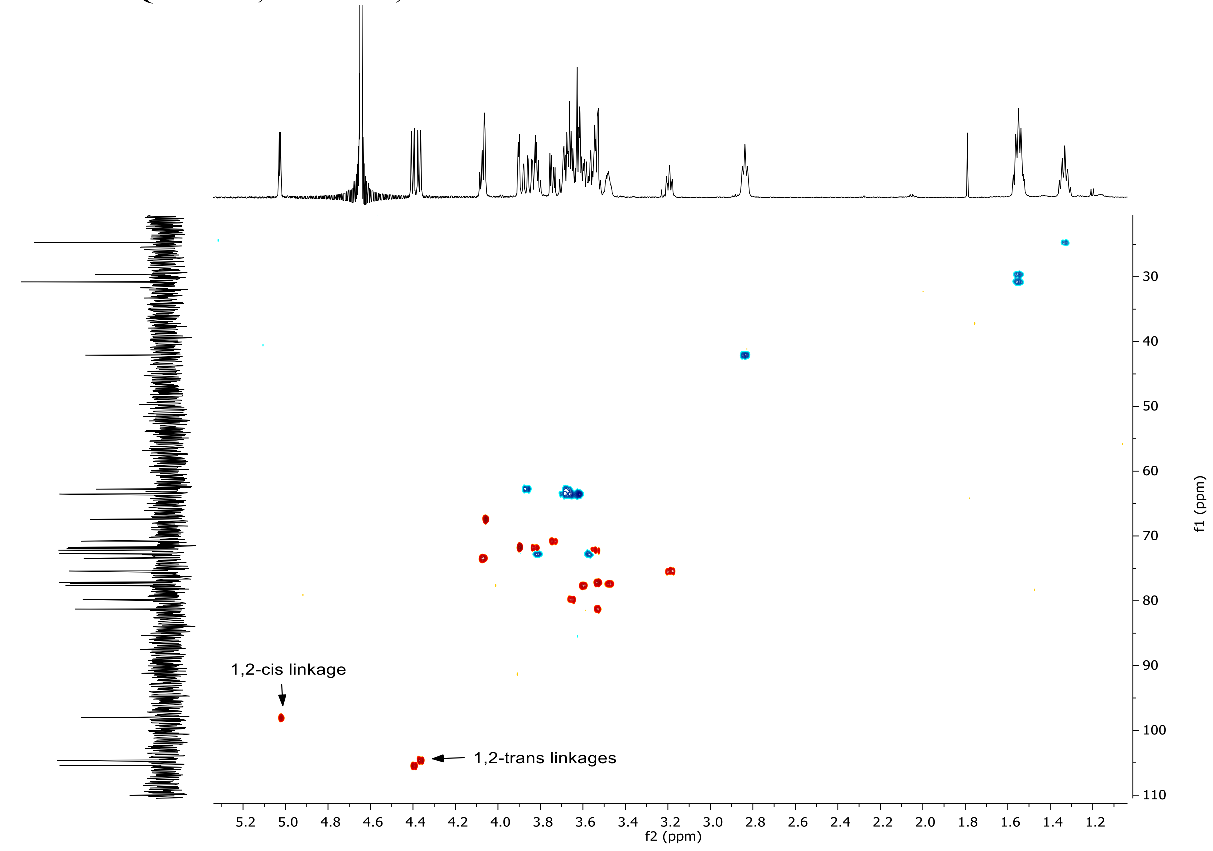

Supplementary Figure 2 | 2D NMR spectra of 1

^{1}H NMR, 600 MHz, D_2O

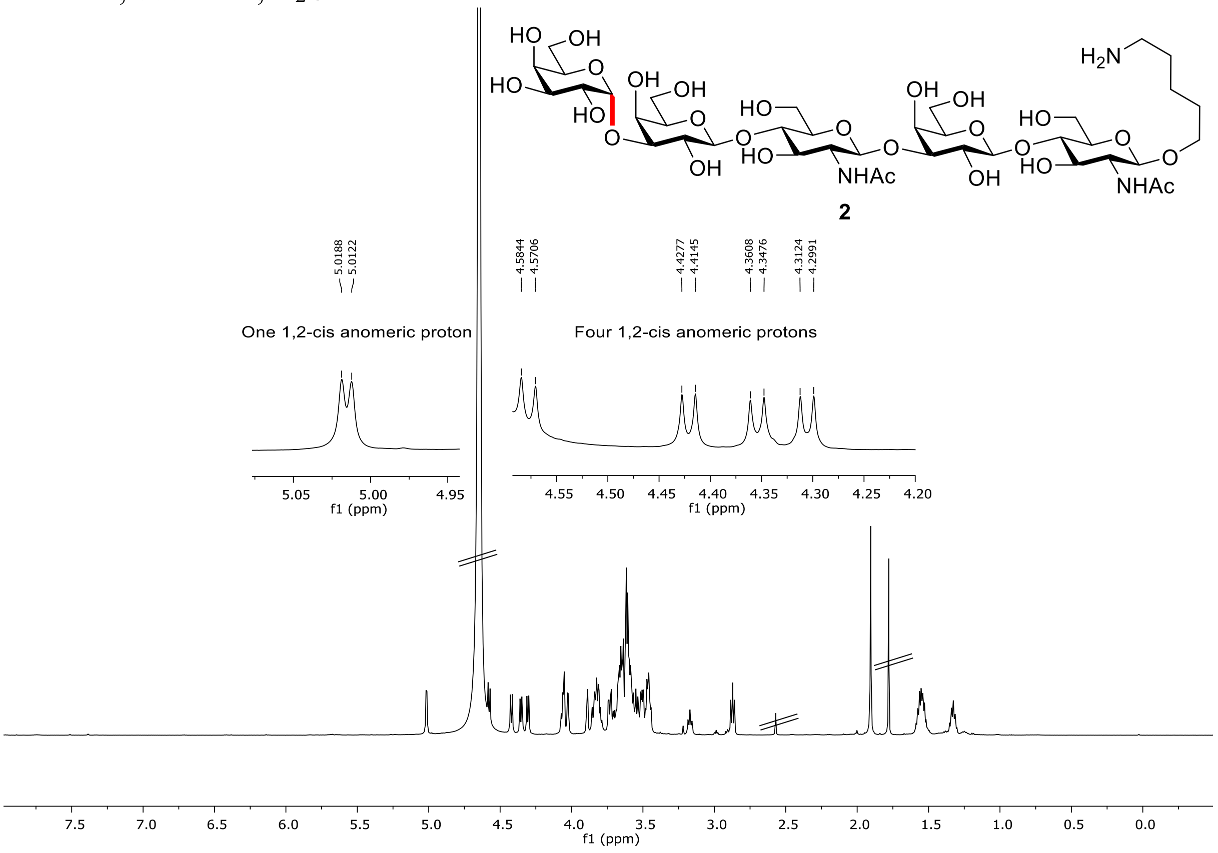

^{13}C NMR, 150 MHz, D_2O

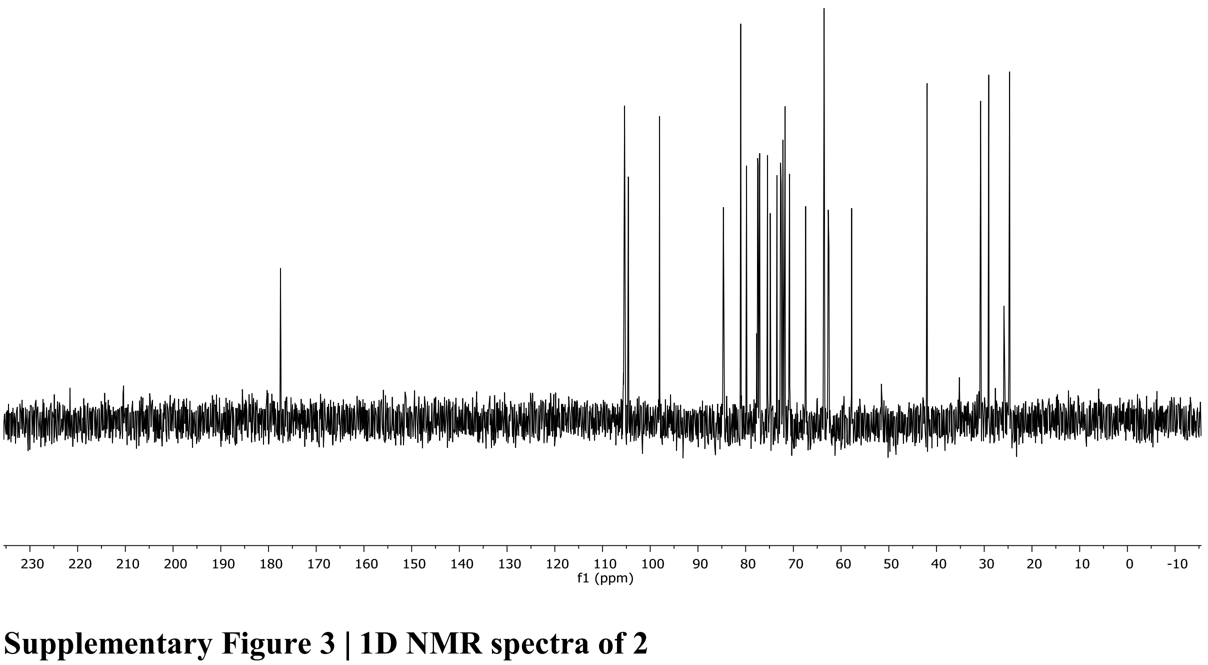

Supplementary Figure 3 | 1D NMR spectra of 2

^{1}H-COSY NMR, 600 MHz, D_2O

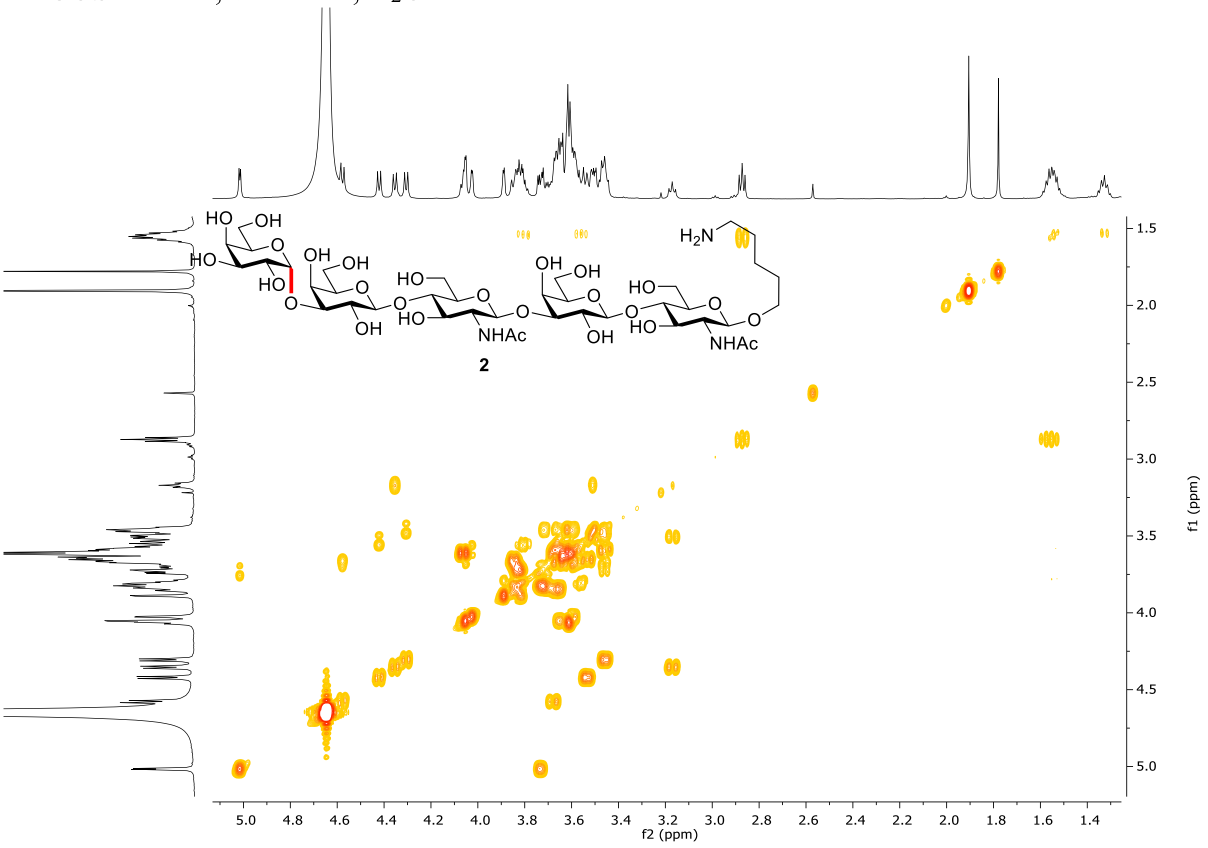

^{1}H-^{13}C-HSQC NMR, 600 MHz, D_2O

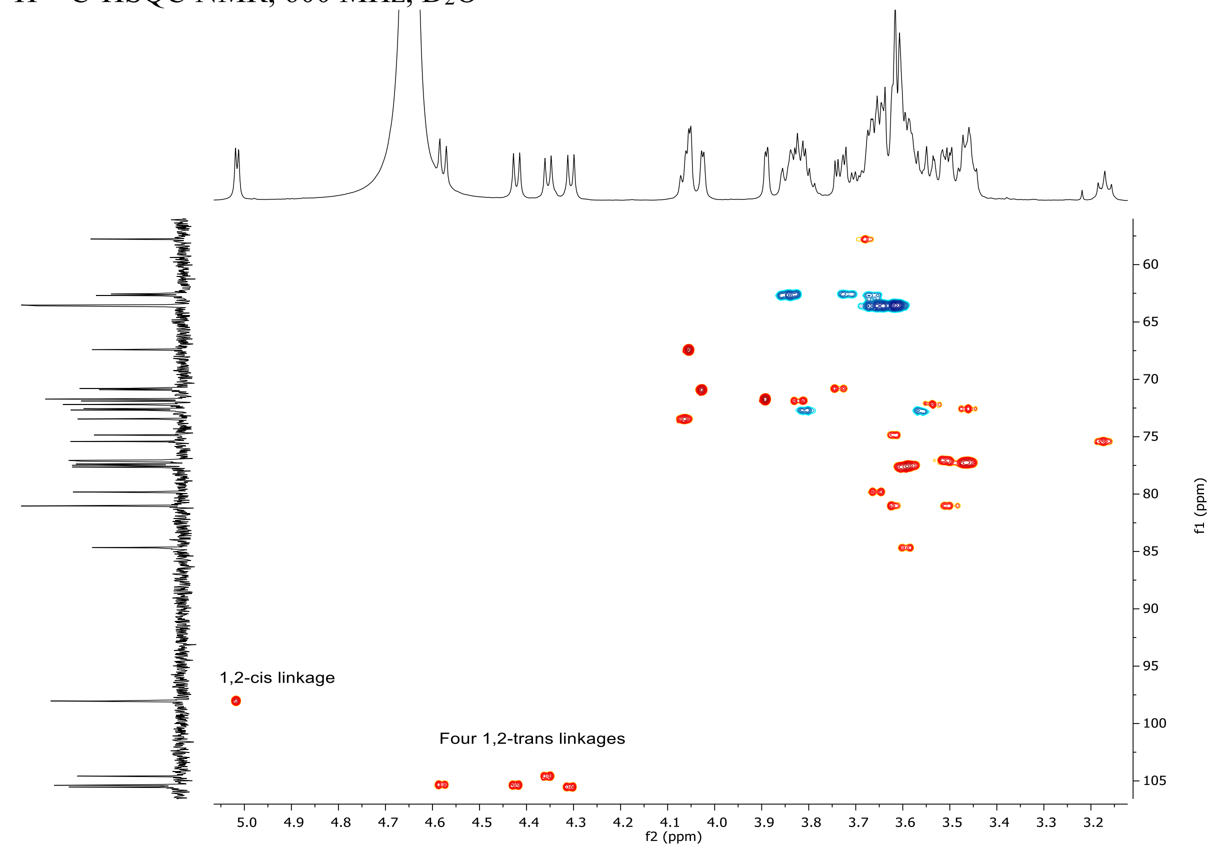

Supplementary Figure 4 | 2D NMR spectra of 2

^{1}H NMR, 600 MHz, D_2O

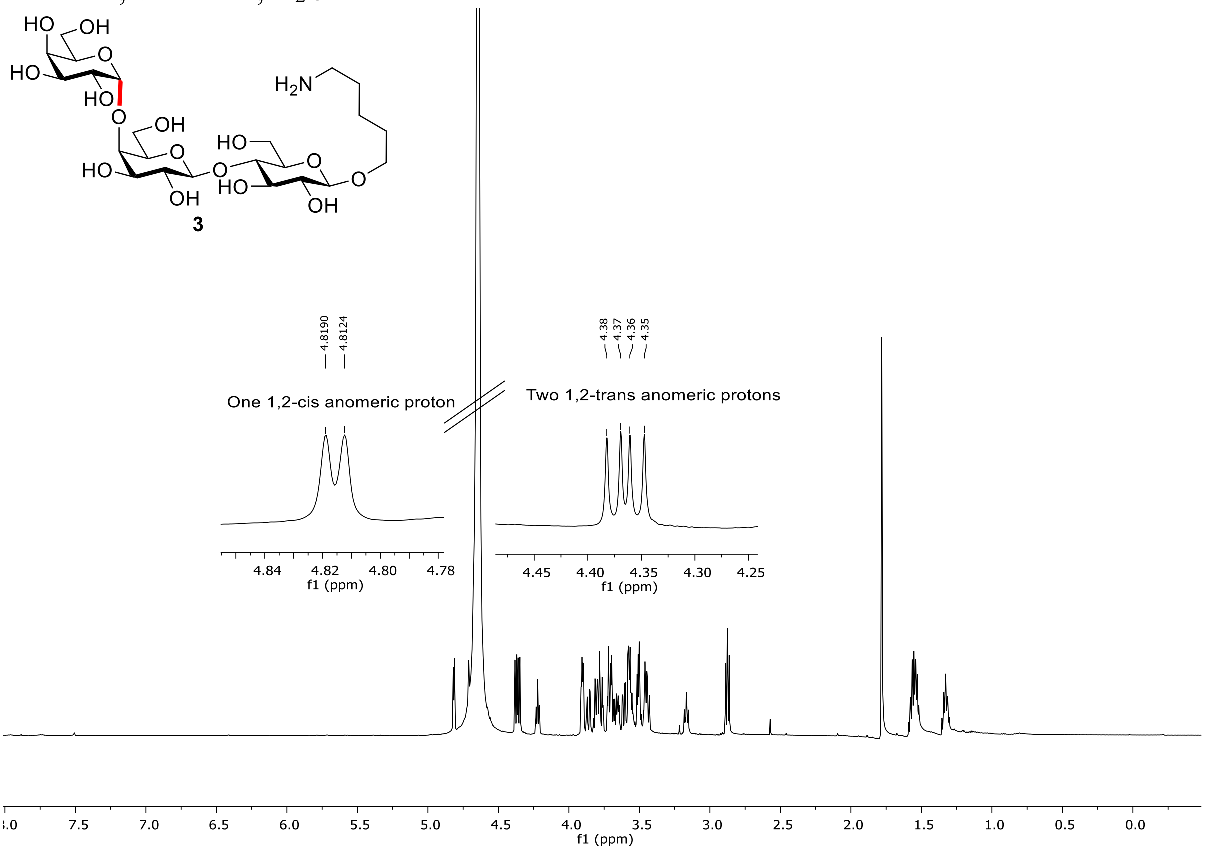

^{13}C NMR, 150 MHz, D_2O

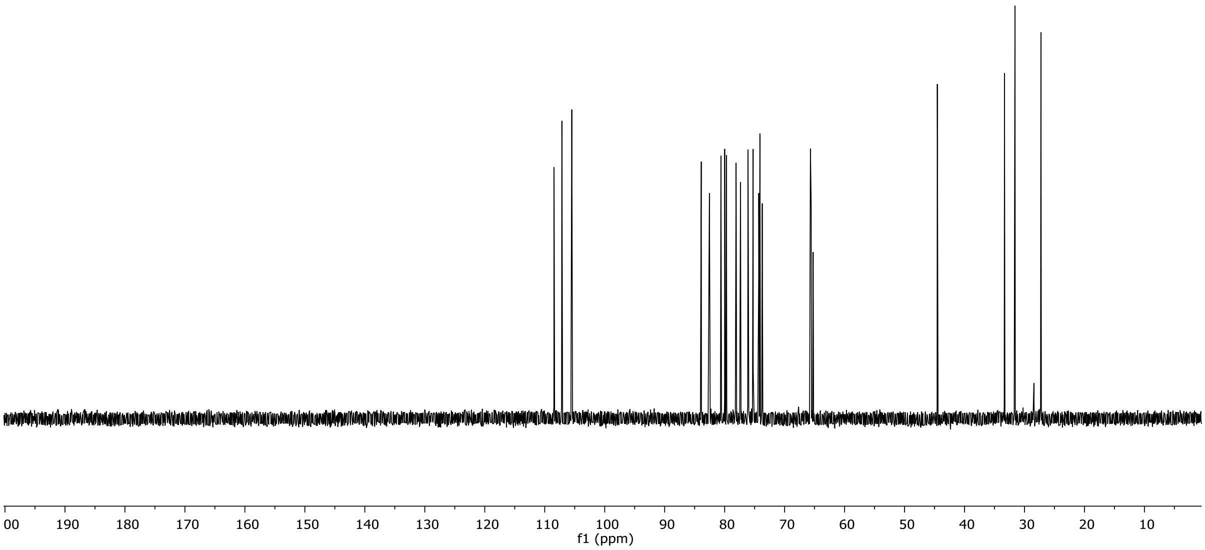

Supplementary Figure 5 | 1D NMR spectra of 3

^{1}H-COSY NMR, 600 MHz, D_2O

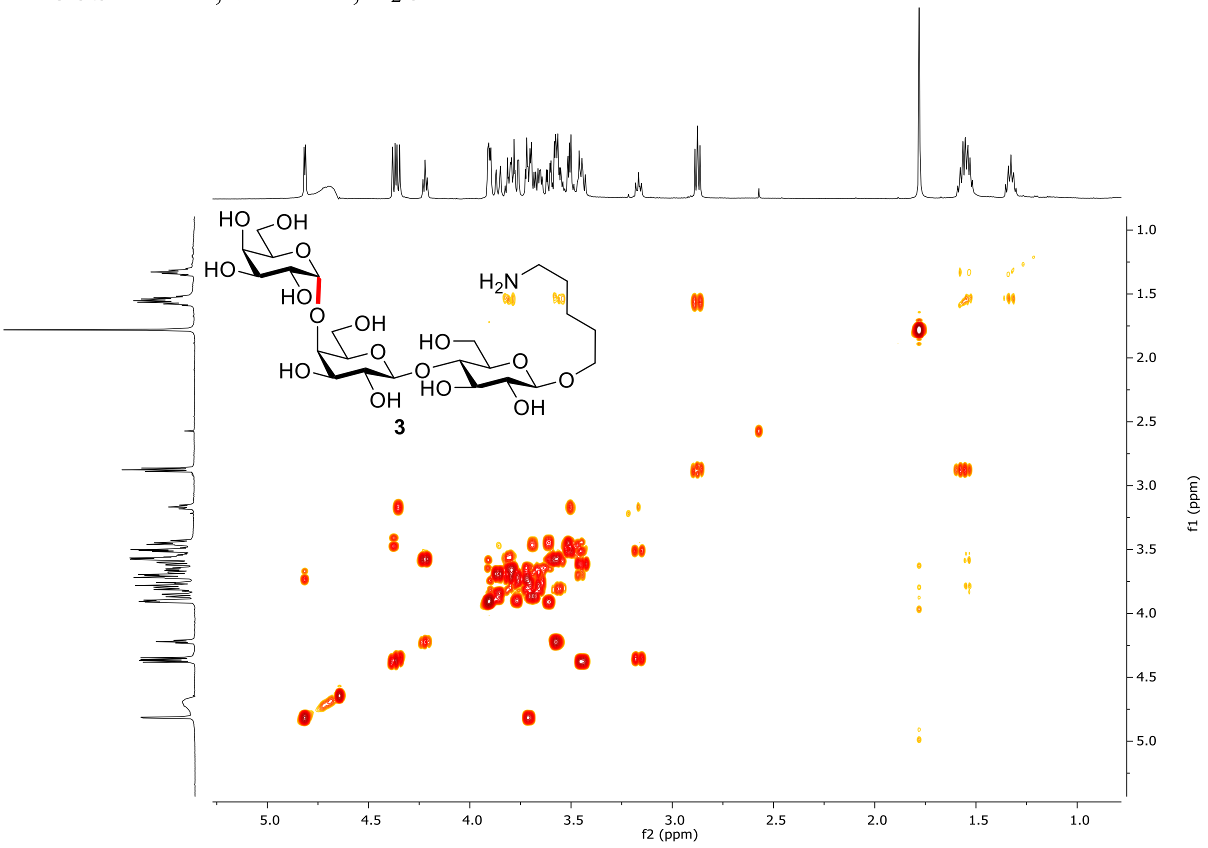

^{1}H-^{13}C-HSQC NMR, 600 MHz, D_2O

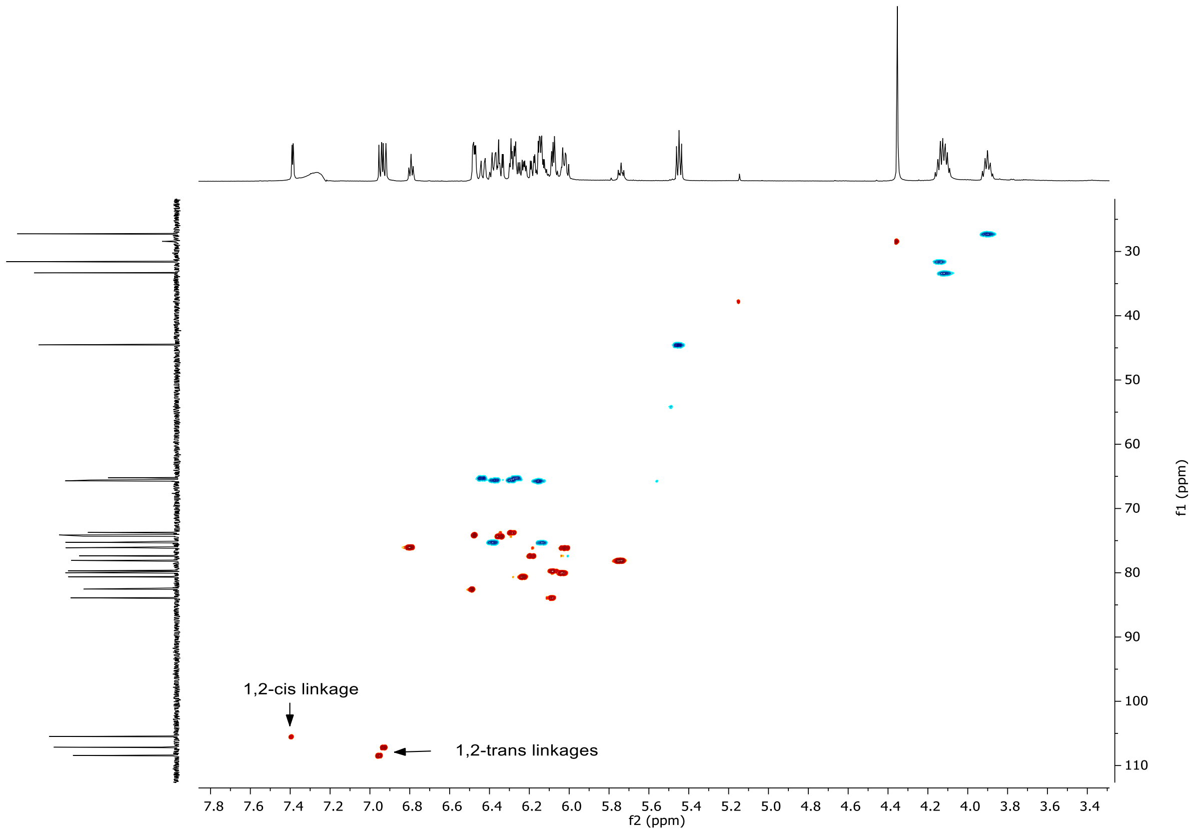

Supplementary Figure 6 | 2D NMR spectra of 3

^{1}H NMR, 500 MHz, D_2O

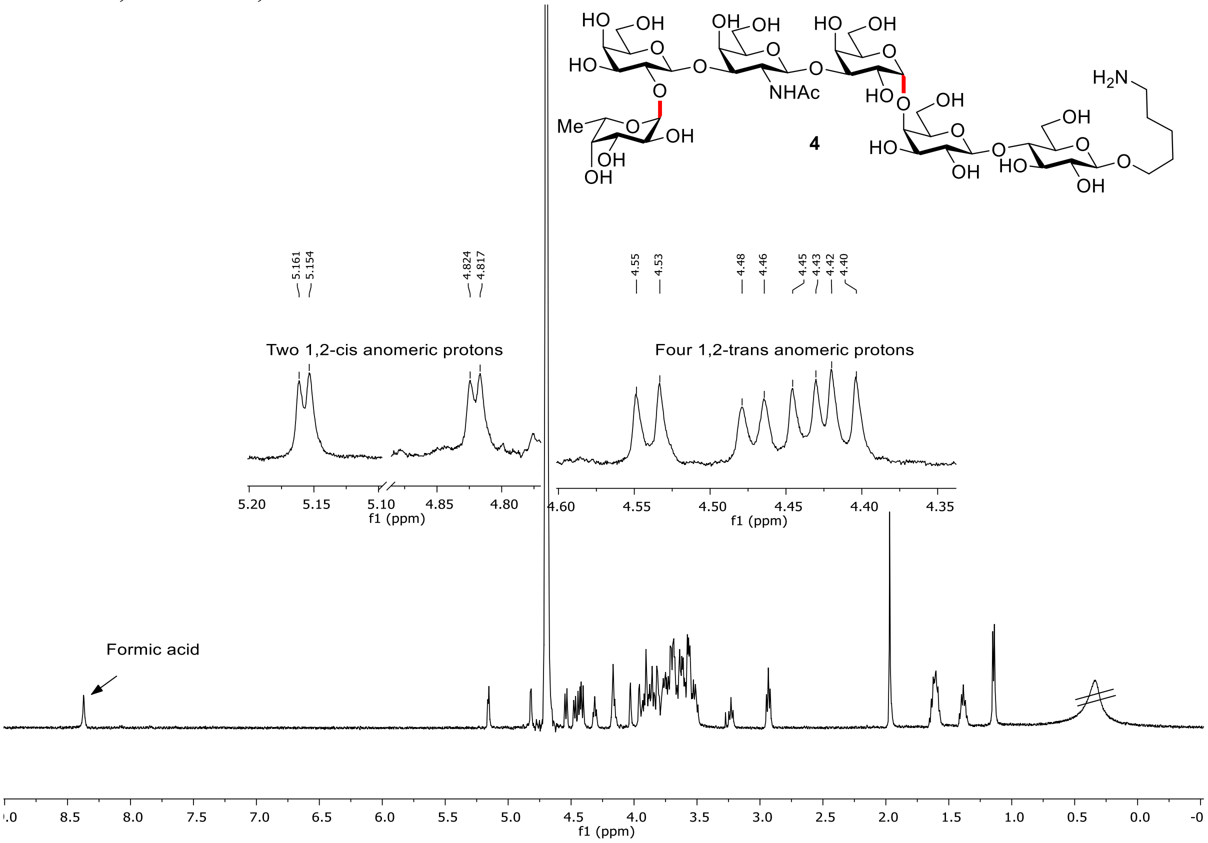

^{13}C NMR, 125 MHz, D_2O

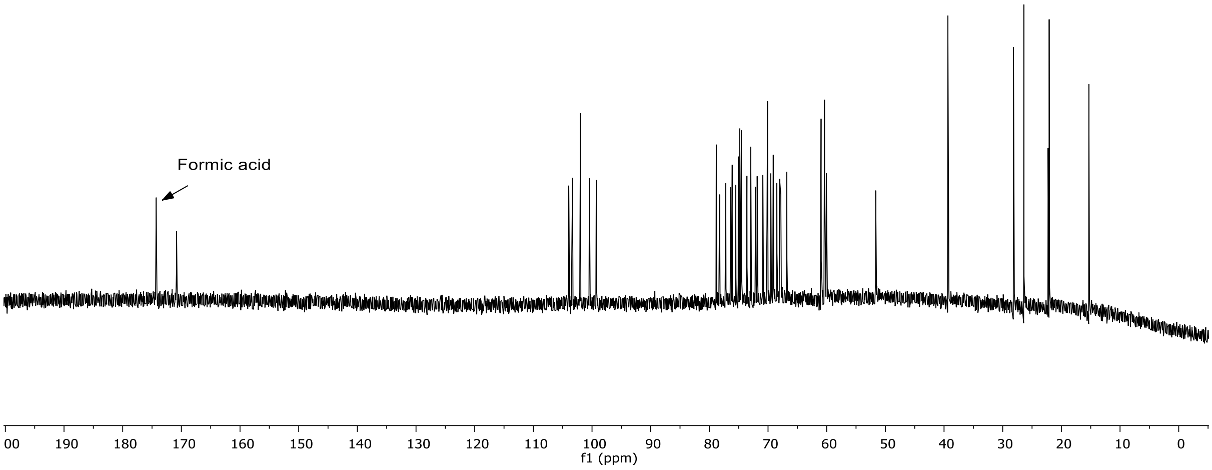

Supplementary Figure 7 | 1D NMR spectra of 4

^{1}H-COSY NMR, 500 MHz, D_2O

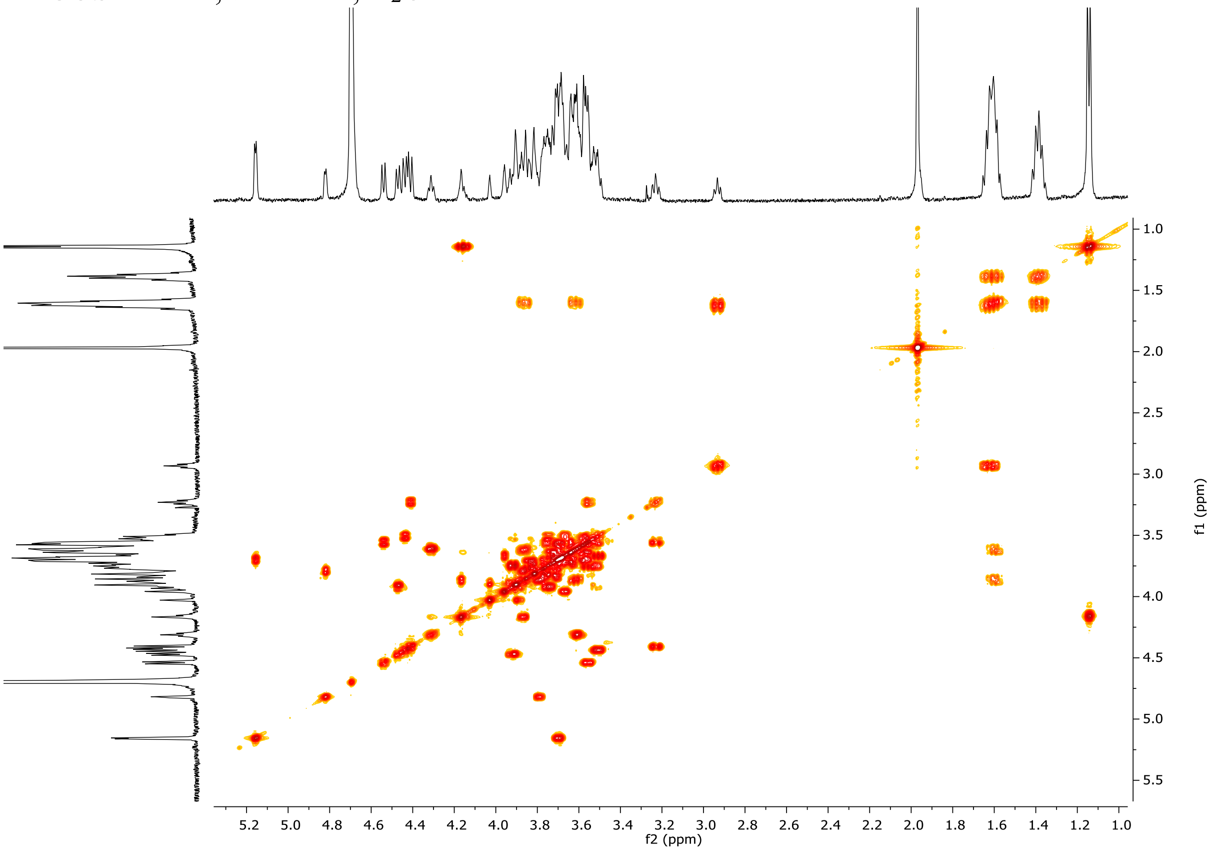

^{1}H-^{13}C-HSQC NMR, 500 MHz, D_2O

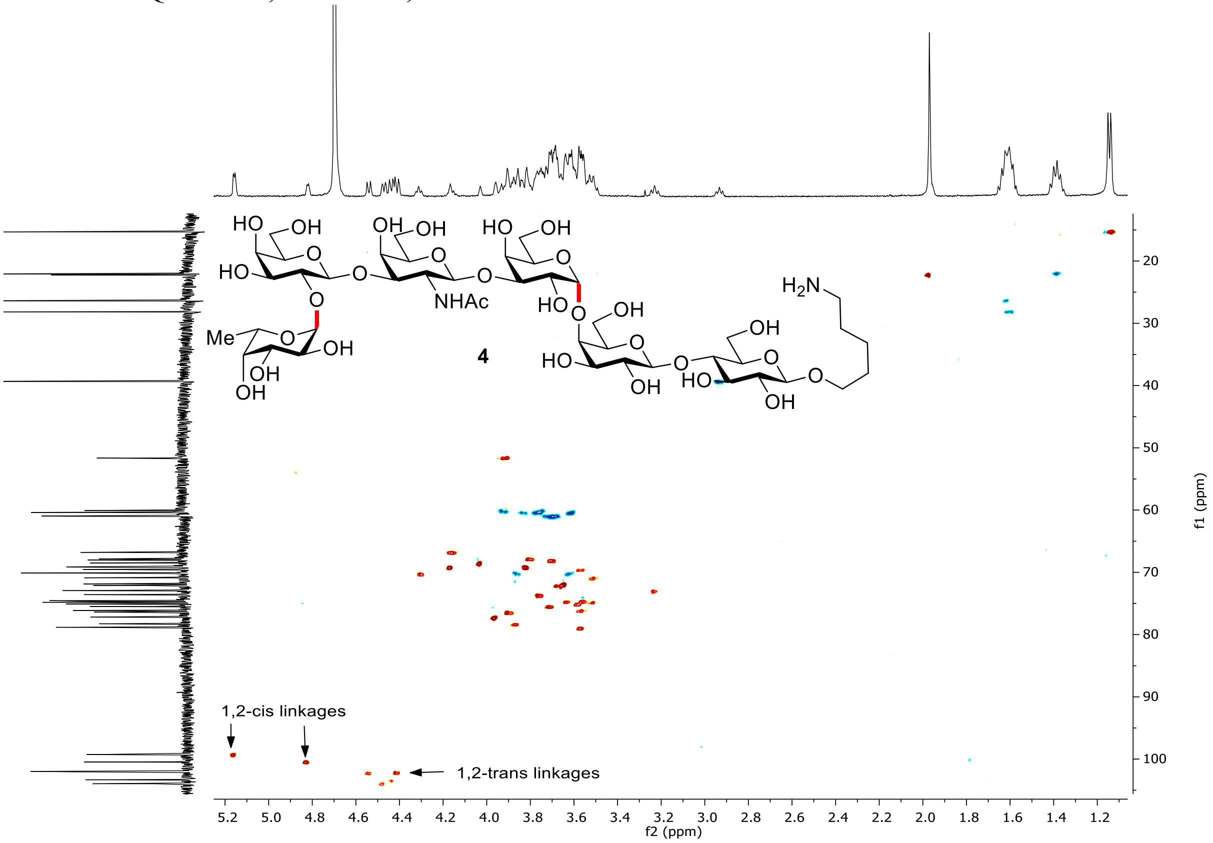

Supplementary Figure 8 | 2D NMR spectra of 4

^{1}H NMR, 700 MHz, D_2O

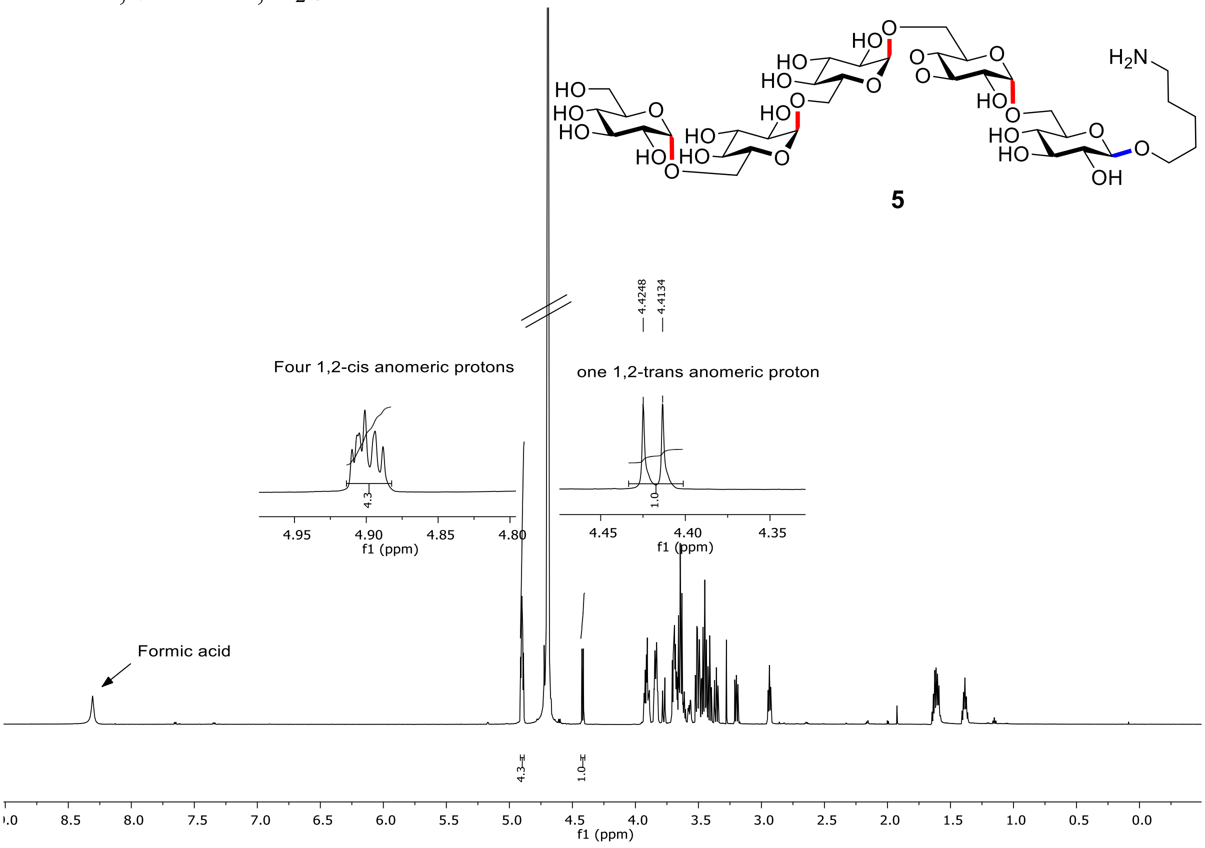

^{13}C NMR, 175 MHz, D_2O

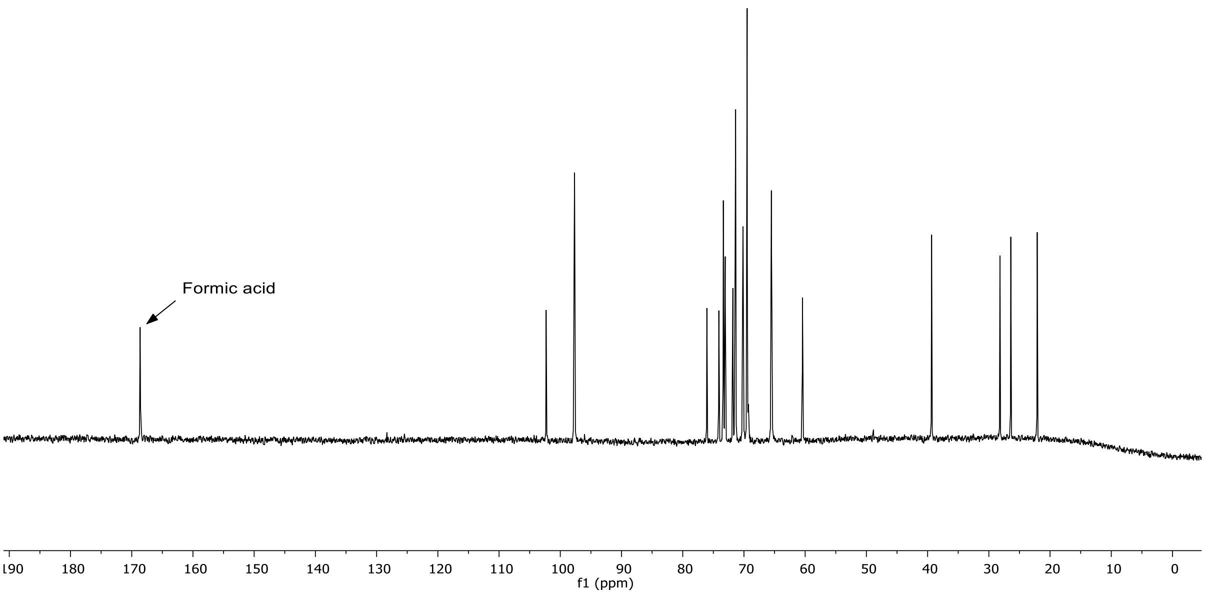

Supplementary Figure 9 | 1D NMR spectra of 5

^{1}H-COSY NMR, 700 MHz, D_2O

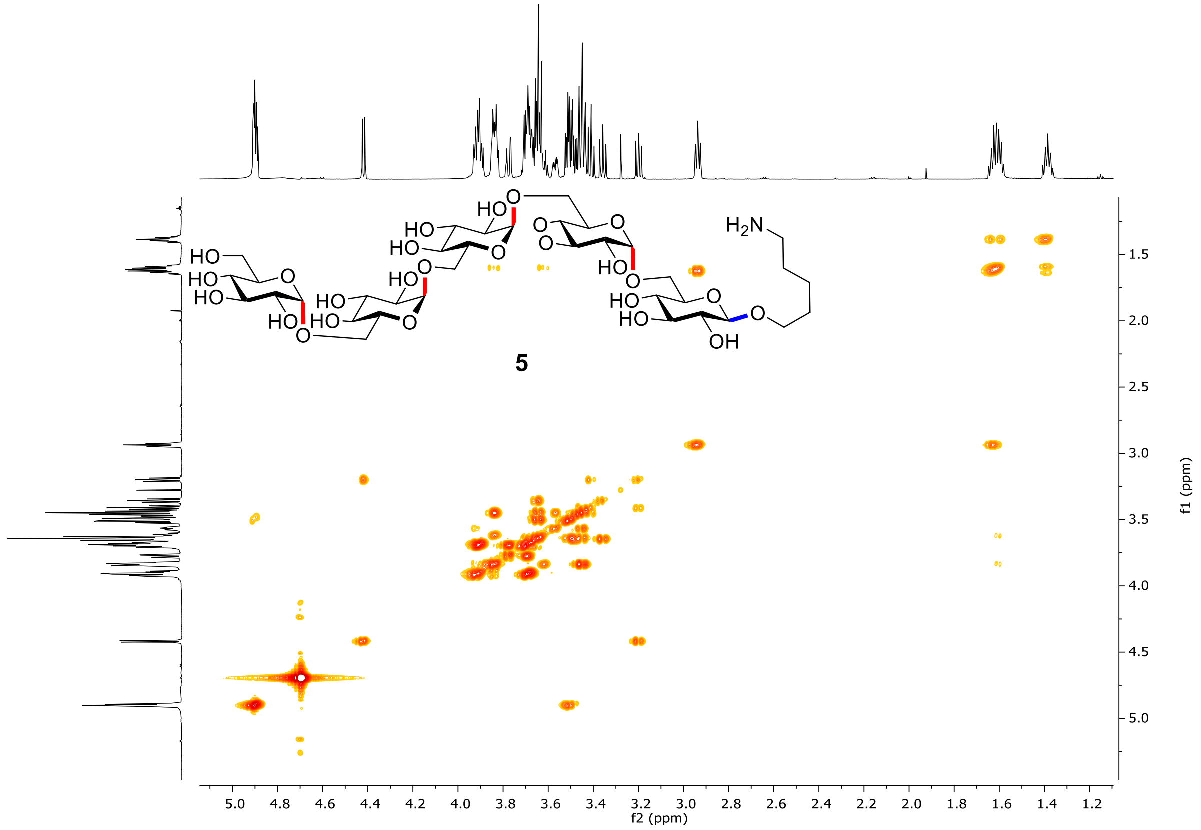

^{1}H-^{13}C-HSQC NMR, 700 MHz, D_2O

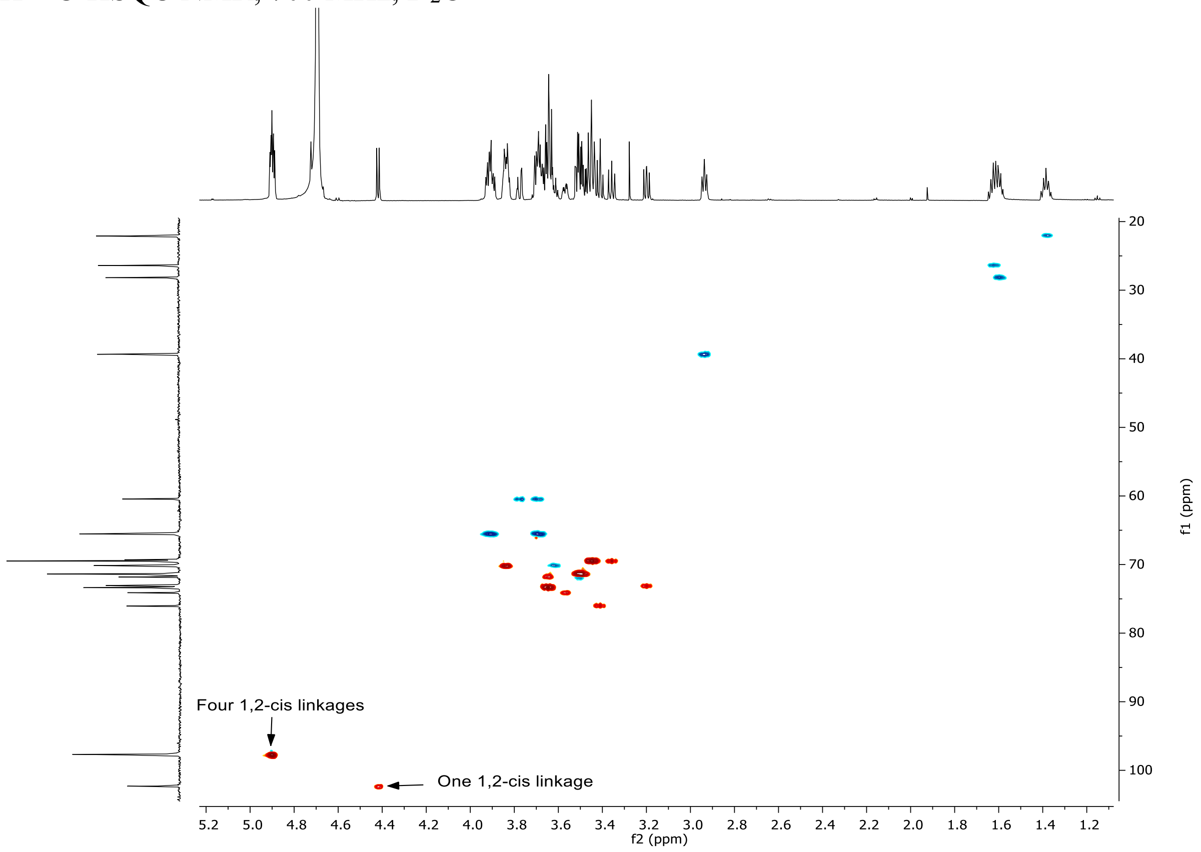

Supplementary Figure 10 | 2D NMR spectra of 5

^{1}H NMR, 700 MHz, D_2O

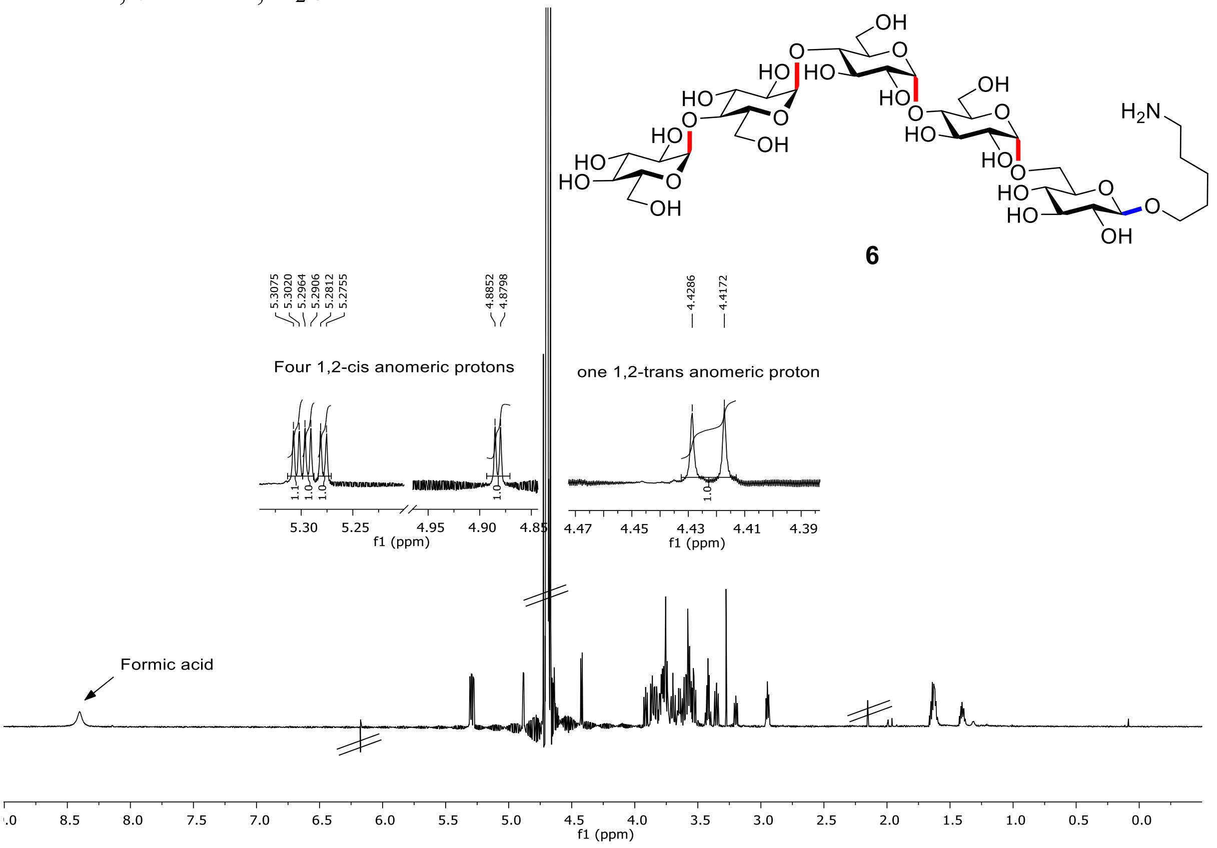

6

^{13}C NMR, 175 MHz, D_2O

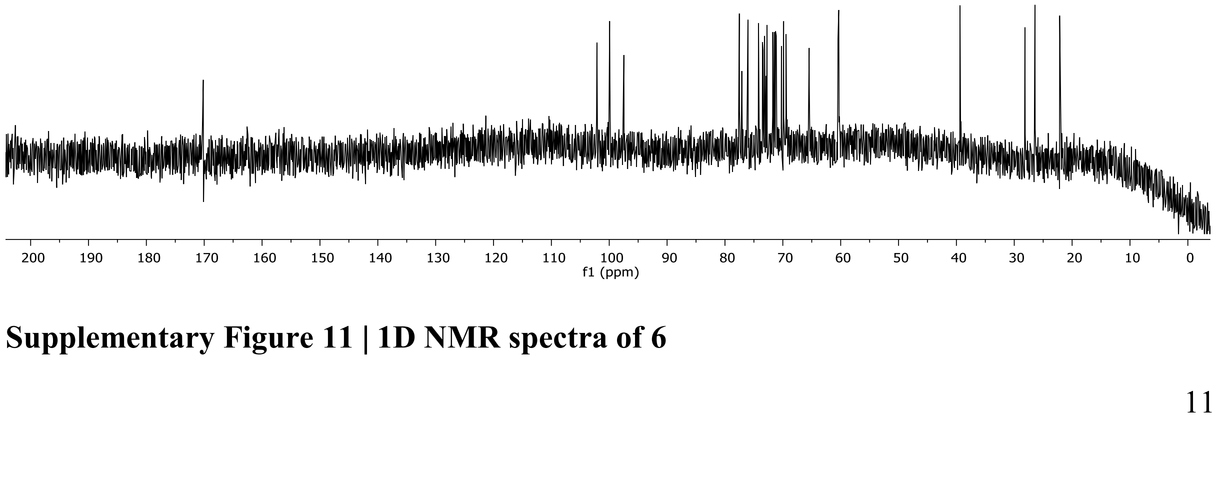

Supplementary Figure 11 | 1D NMR spectra of 6

^{1}H-COSY NMR, 700 MHz, D_2O

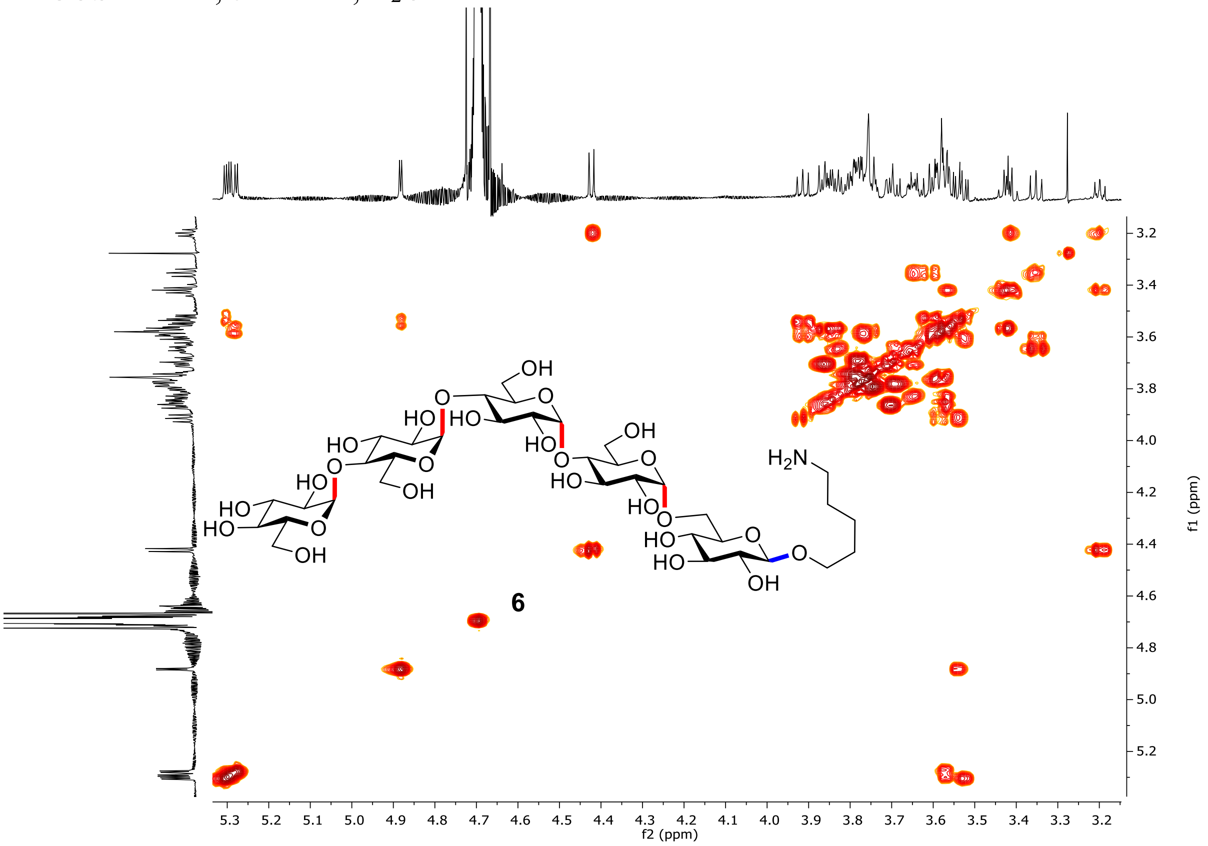

^{1}H-^{13}C-HSQC NMR, 700 MHz, D_2O

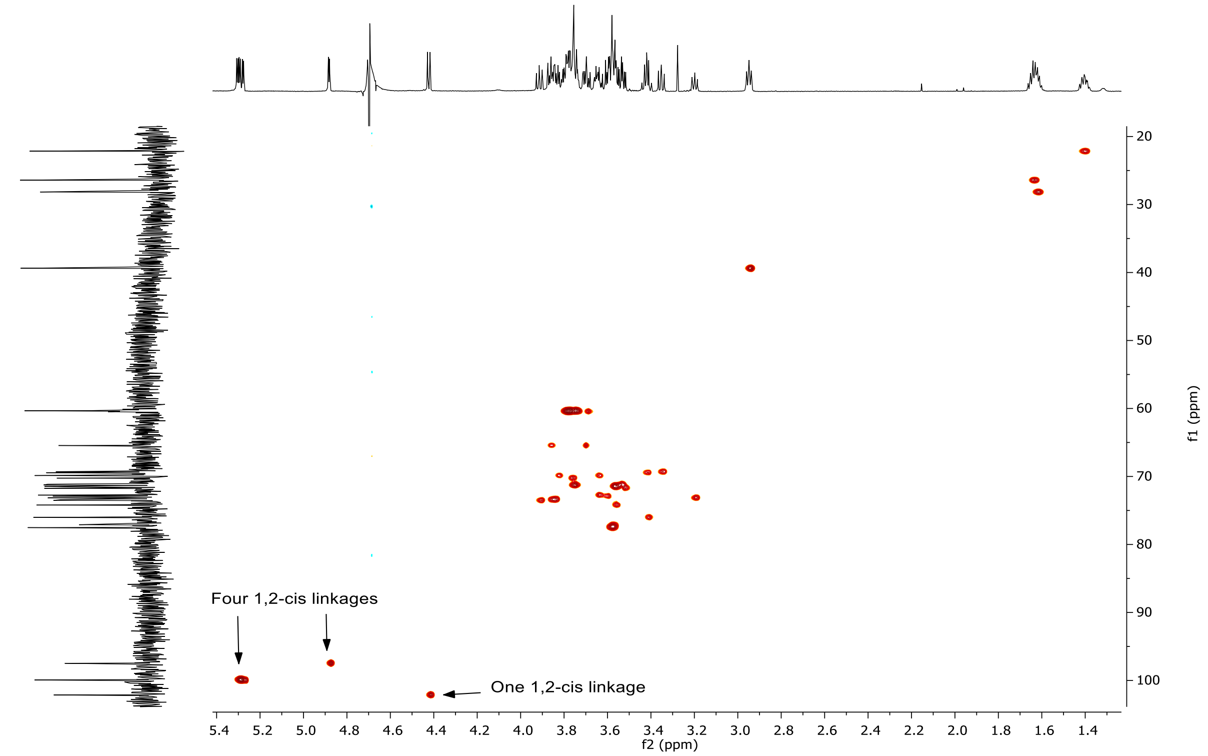

Supplementary Figure 12 | 2D NMR spectra of 6

^{1}H NMR, 700 MHz, D_2O

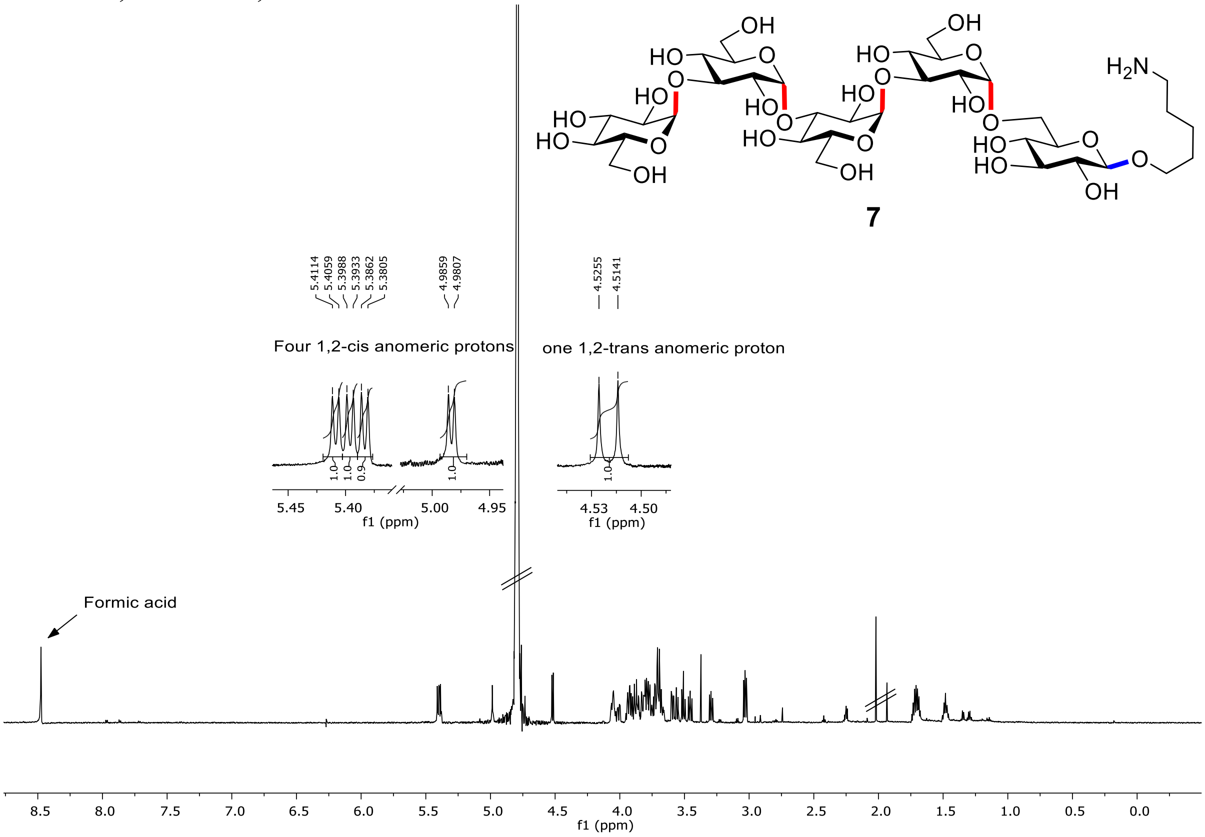

^{13}C NMR, 175 MHz, D_2O

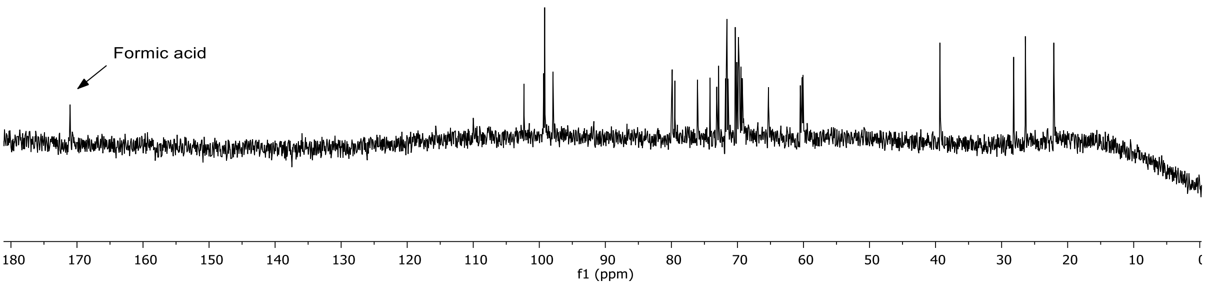

Supplementary Figure 13 | 1D NMR spectra of 7

^{1}H-COSY NMR, 700 MHz, D_2O

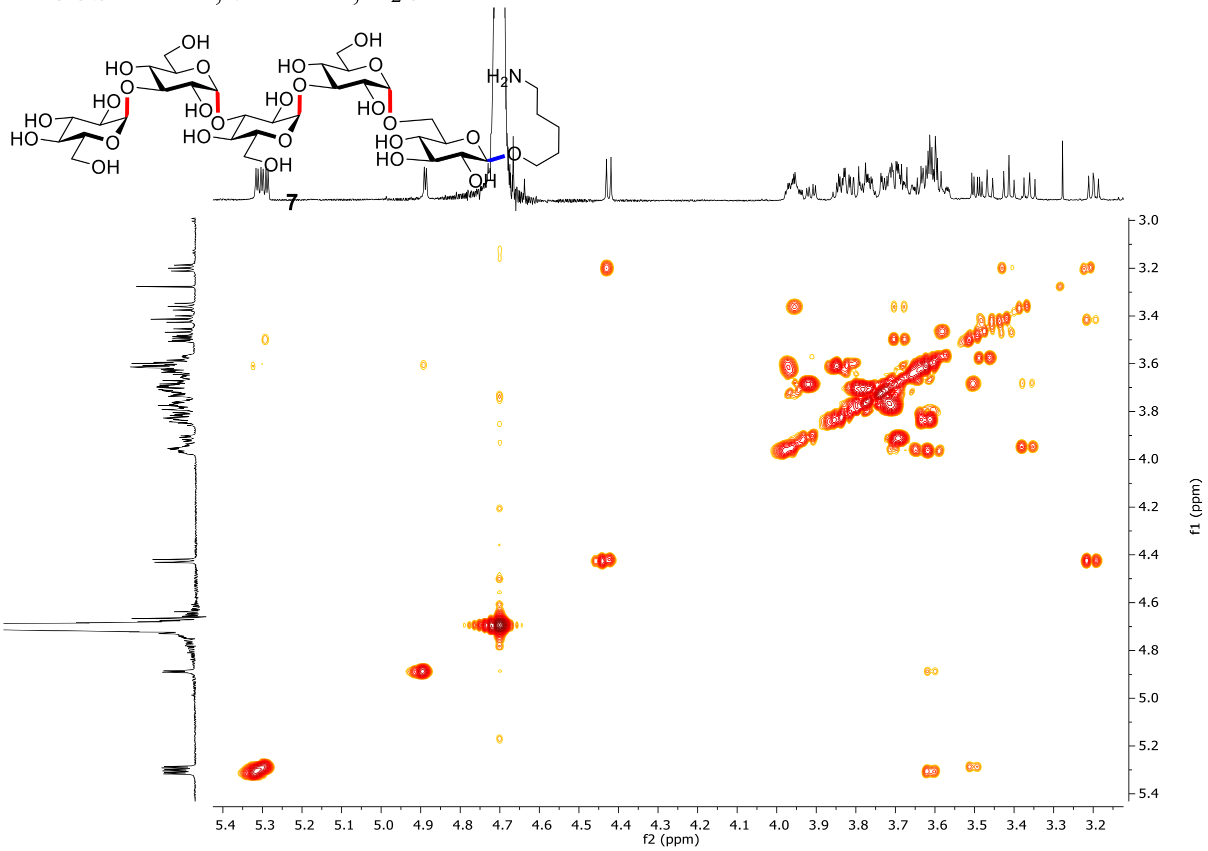

^{1}H-^{13}C-HSQC NMR, 700 MHz, D_2O

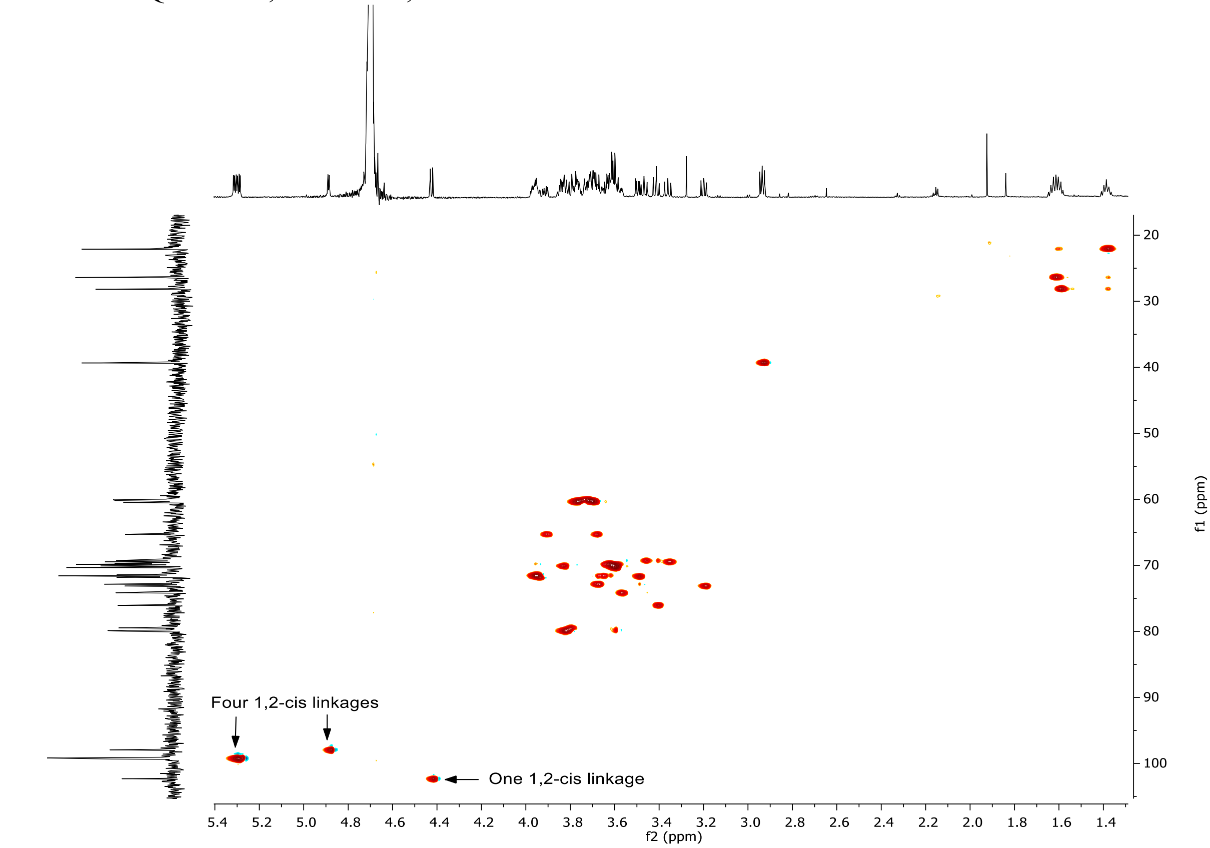

Supplementary Figure 14 | 2D NMR spectra of 7

^{1}H NMR, 700 MHz, D_2O

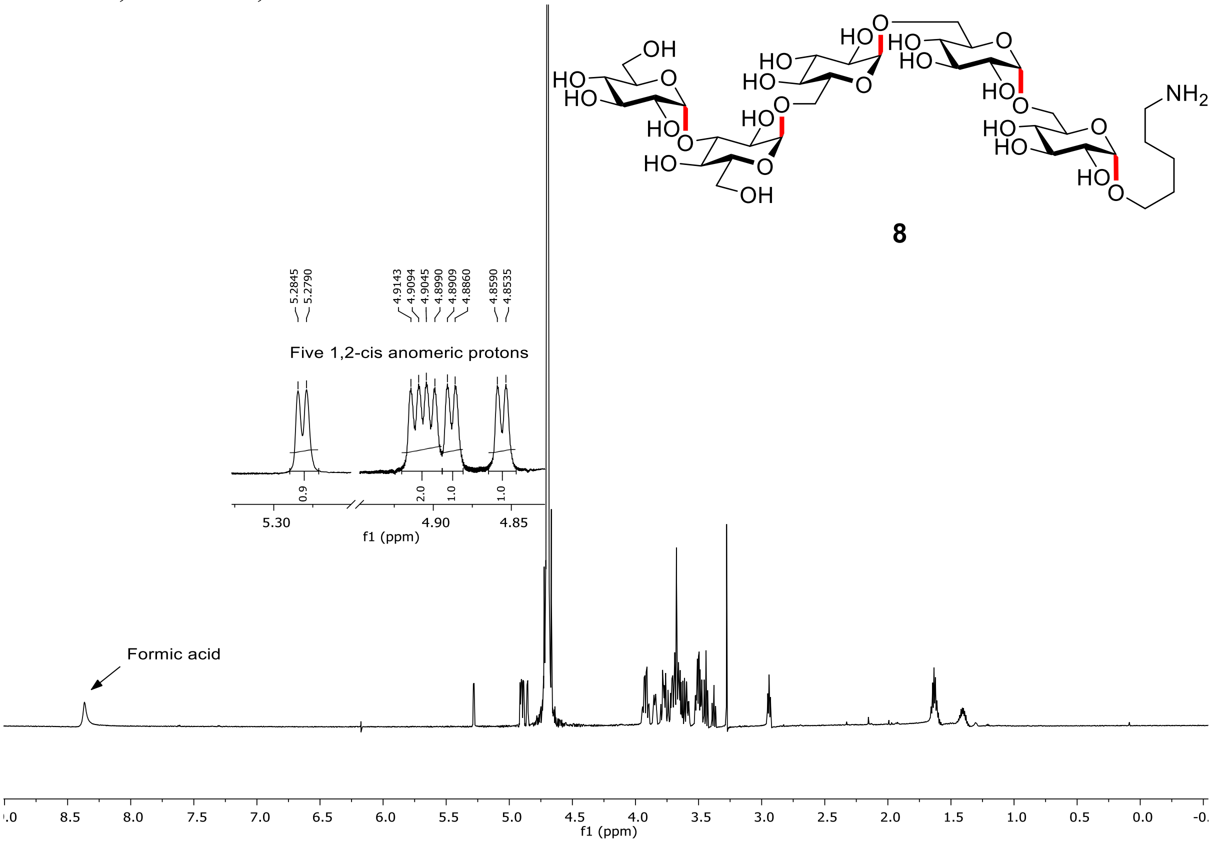

^{13}C NMR, 175 MHz, D_2O

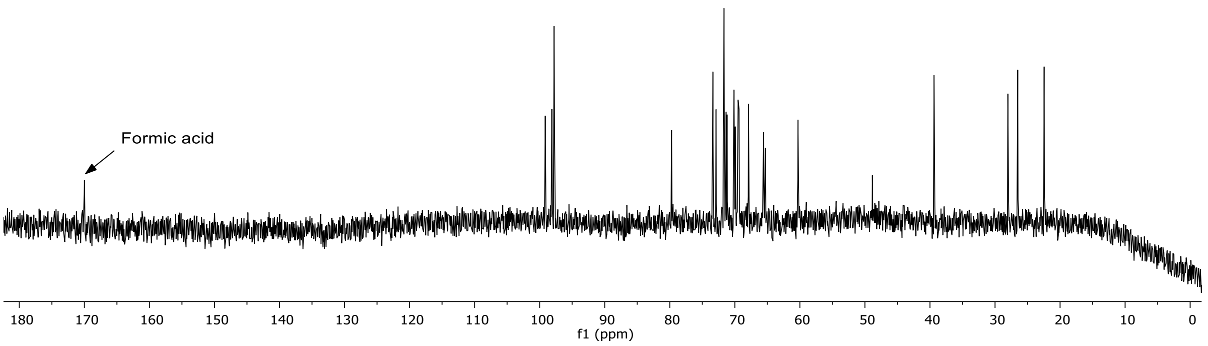

Supplementary Figure 15 | 1D NMR spectra of 8

^{1}H-COSY NMR, 700 MHz, D_2O

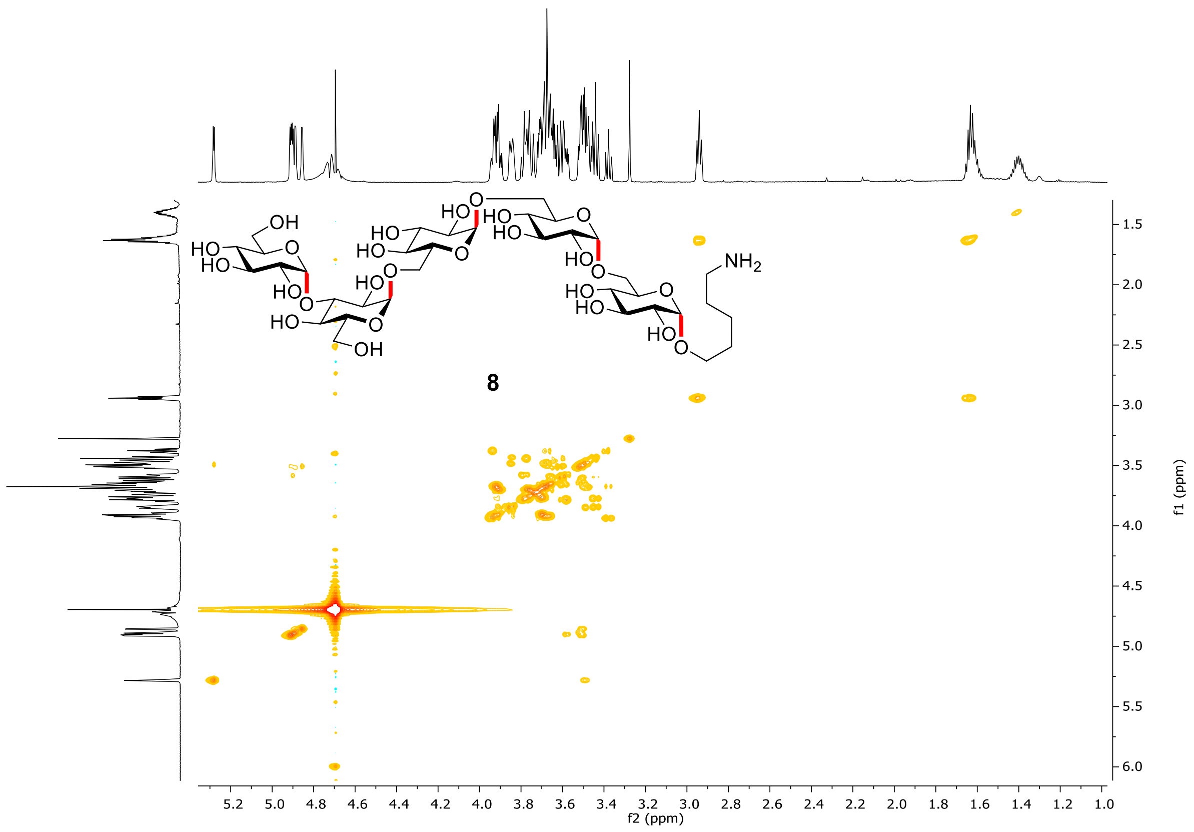

^{1}H-^{13}C-HSQC NMR, 700 MHz, D_2O

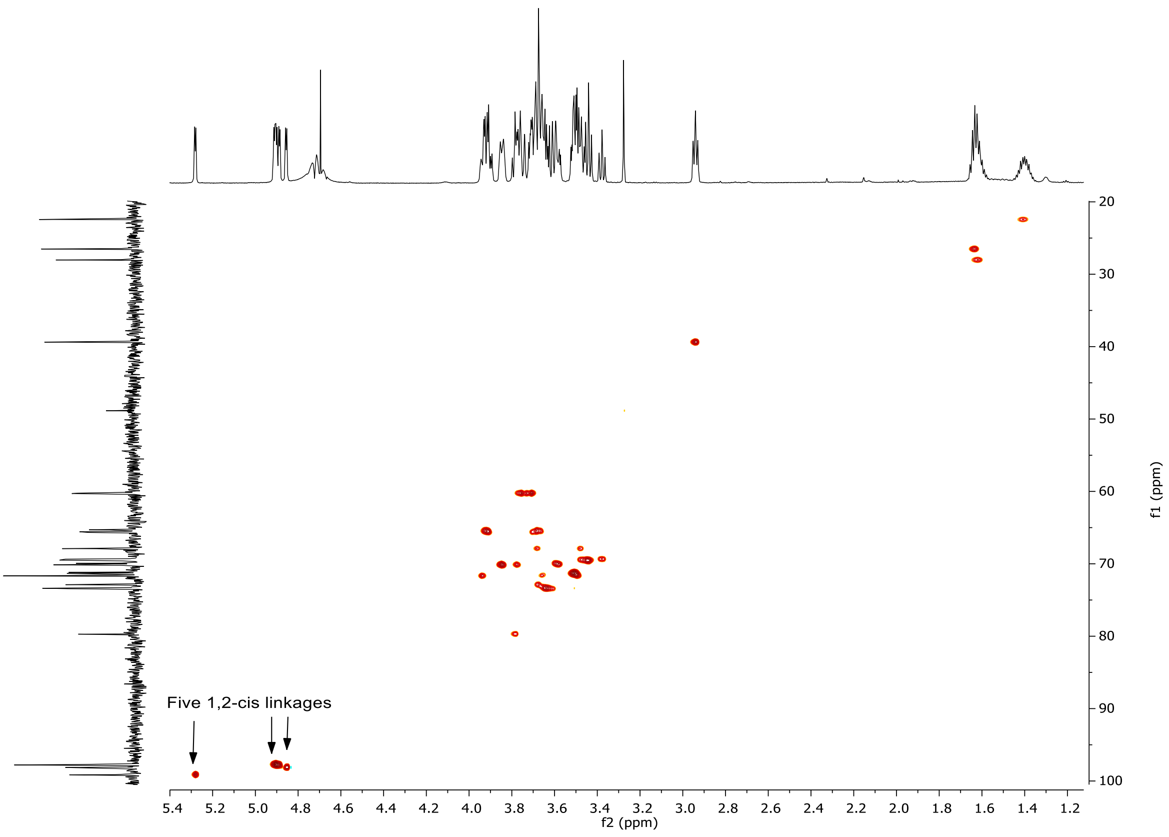

Supplementary Figure 16 | 2D NMR spectra of 8

^{1}H NMR, 700 MHz, D_2O

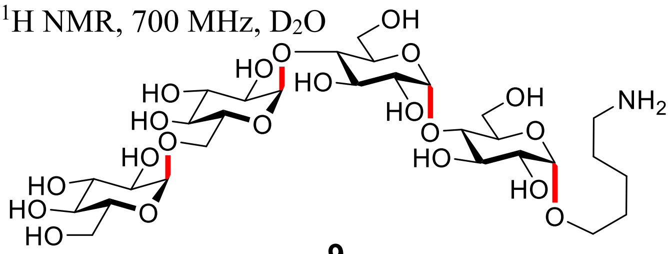

9

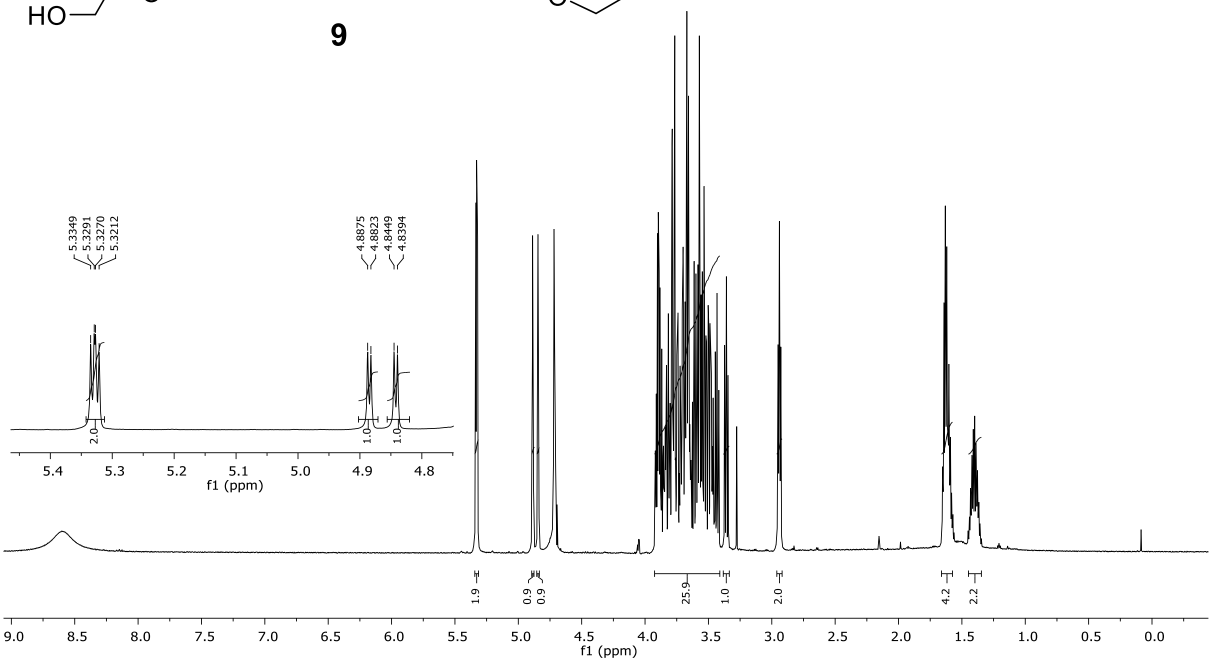

^{13}C NMR, 175 MHz, D_2O

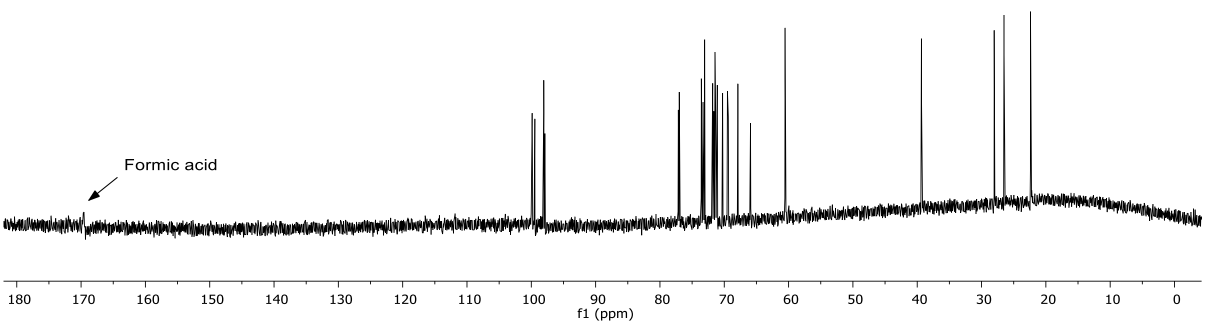

Supplementary Figure 17 | 1D NMR spectra of 9

^{1}H-COSY NMR, 700 MHz, D_2O

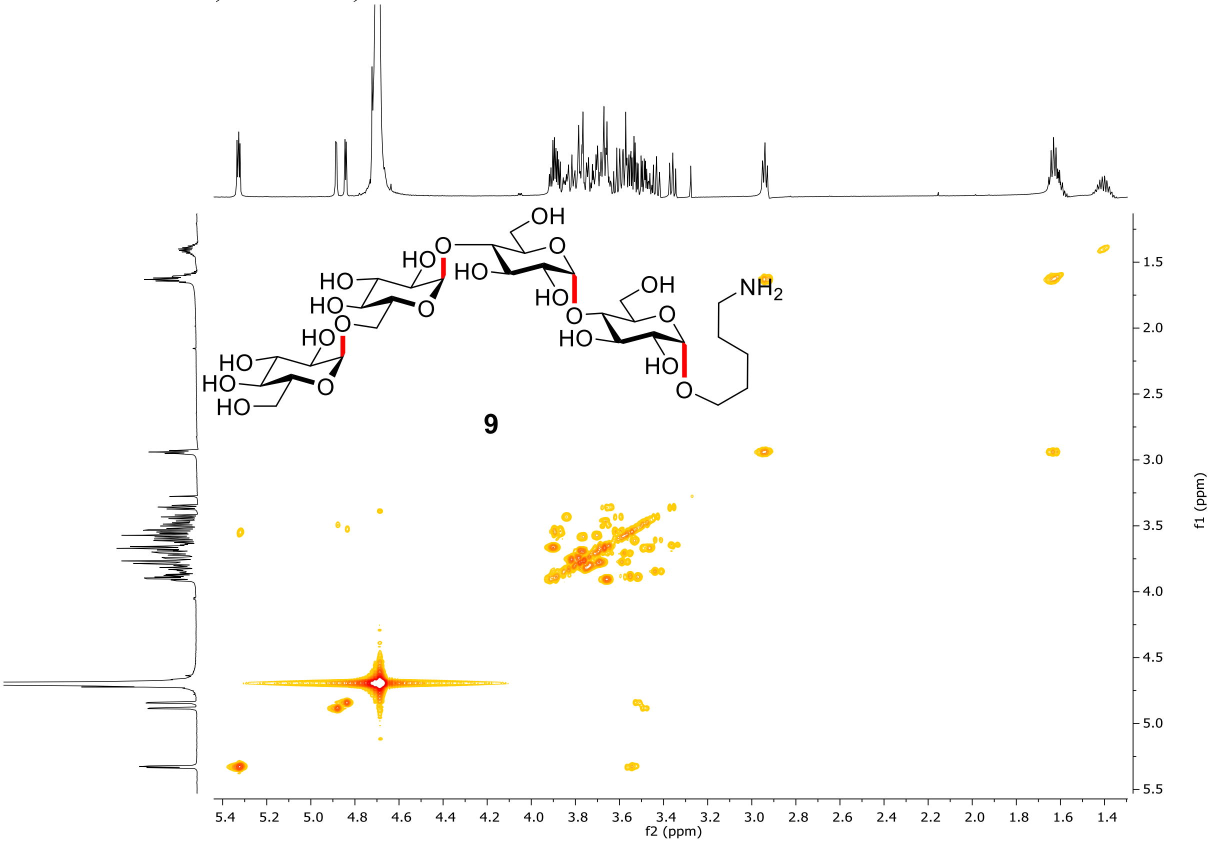

^{1}H-^{13}C-HSQC NMR, 700 MHz, D_2O

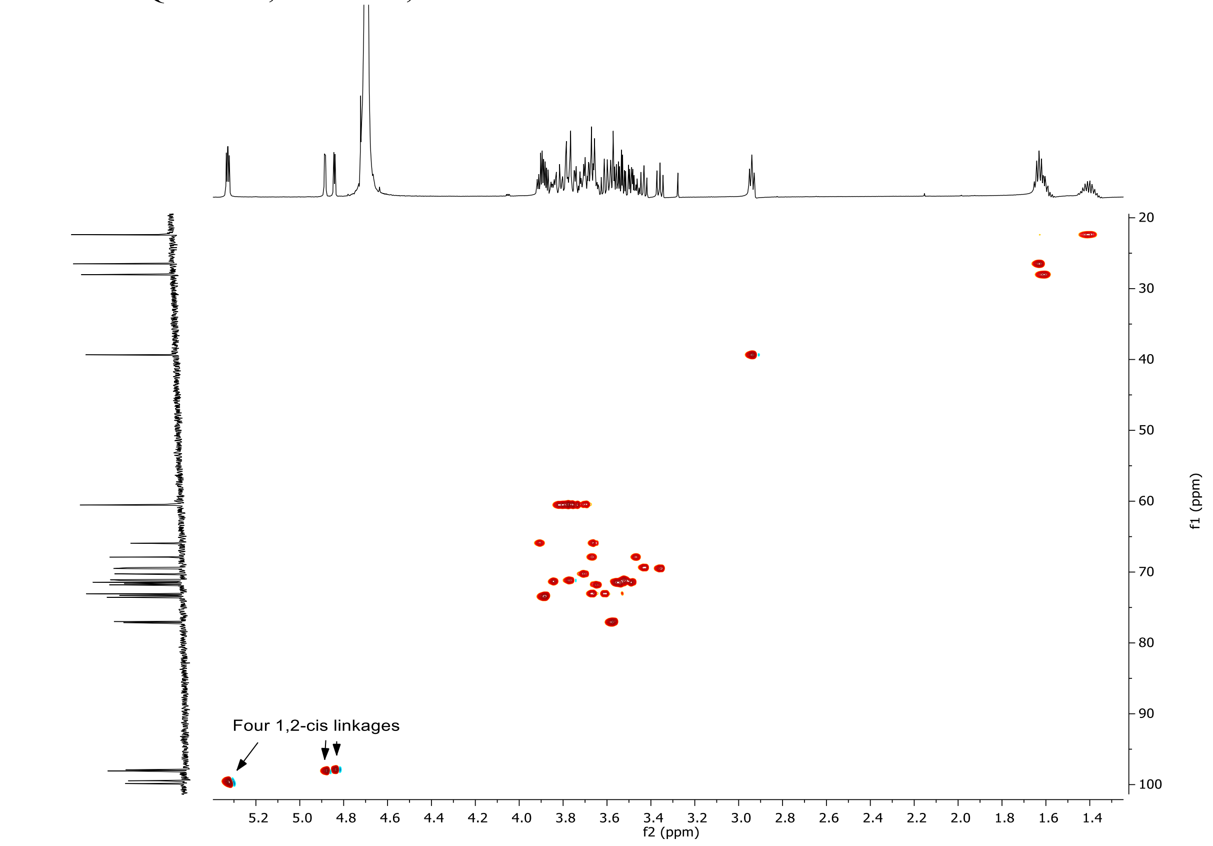

Supplementary Figure 18 | 2D NMR spectra of 9

^{1}H NMR, 400 MHz, $CDCl_3$

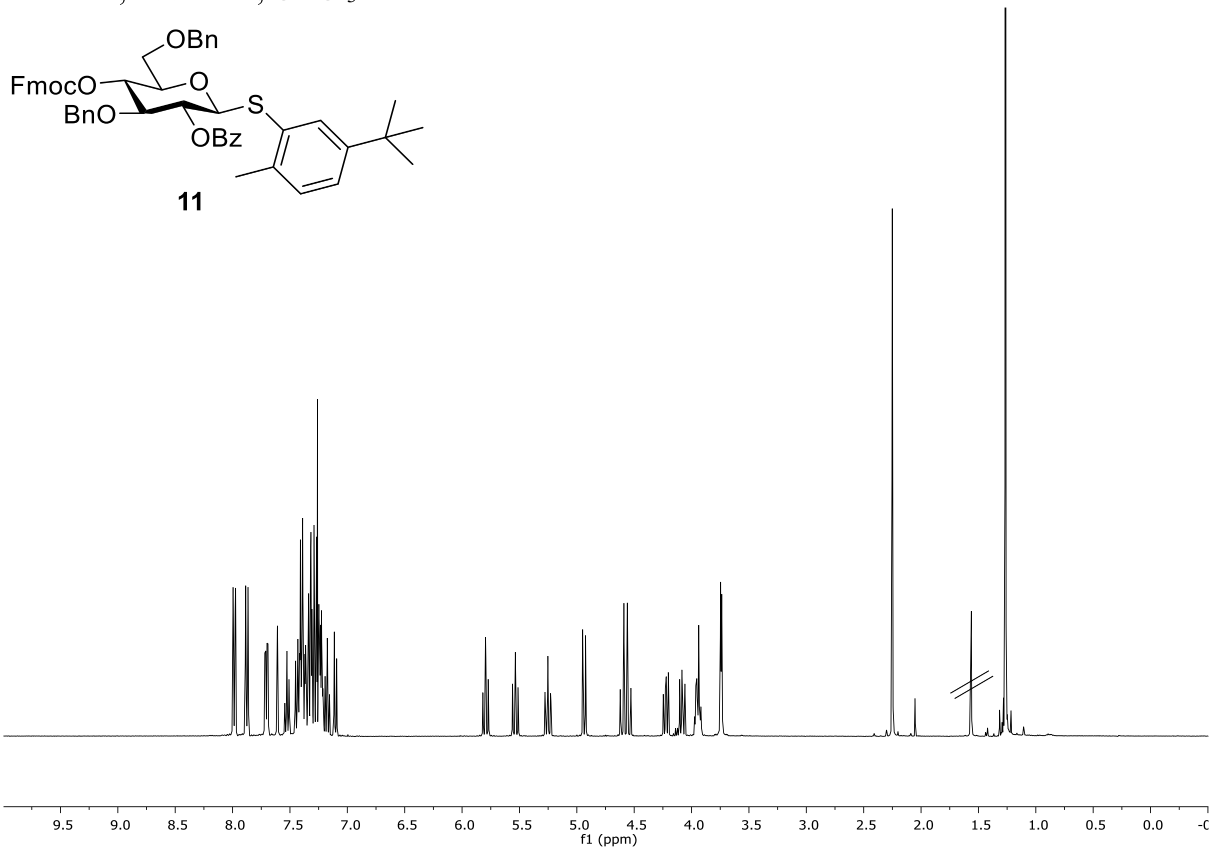

^{13}C NMR, 100 MHz, $CDCl_3$

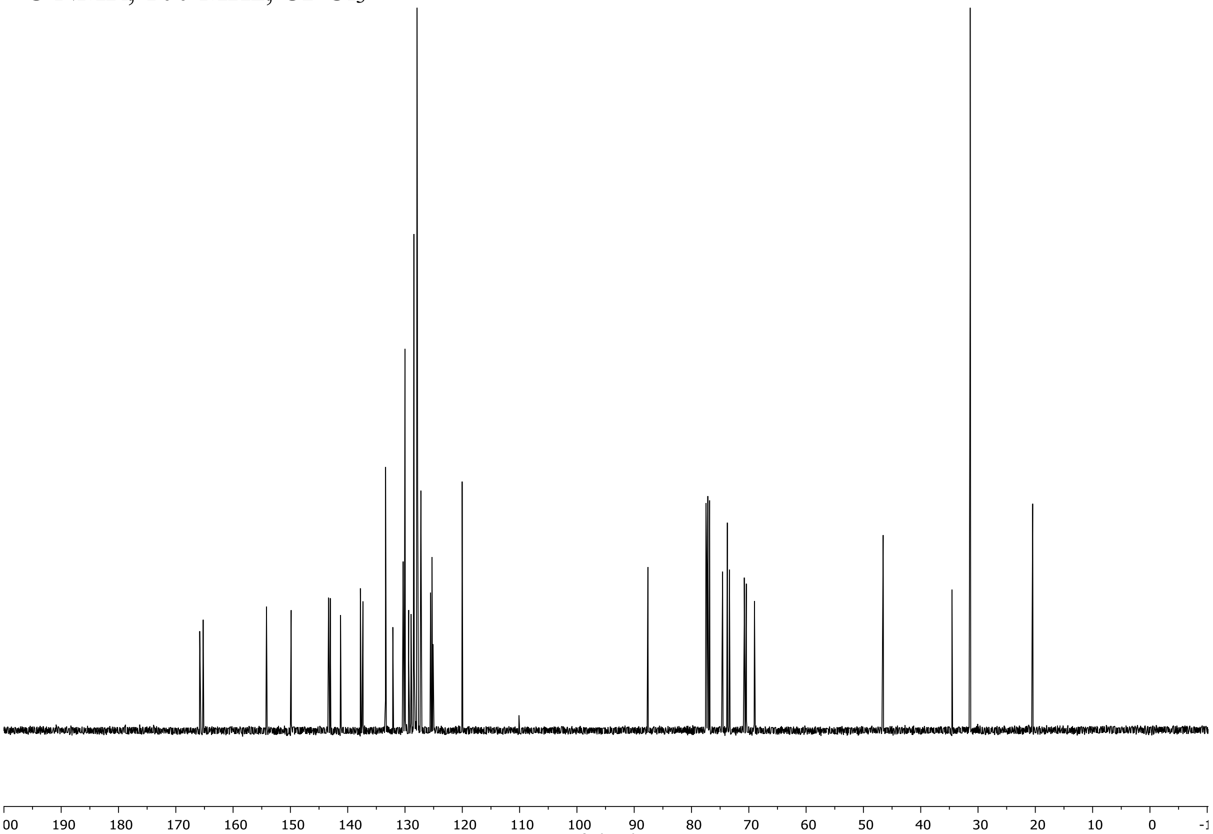

Supplementary Figure 19 | 1D NMR spectra of 11

^{1}H-COSY NMR, 400 MHz, $CDCl_3$

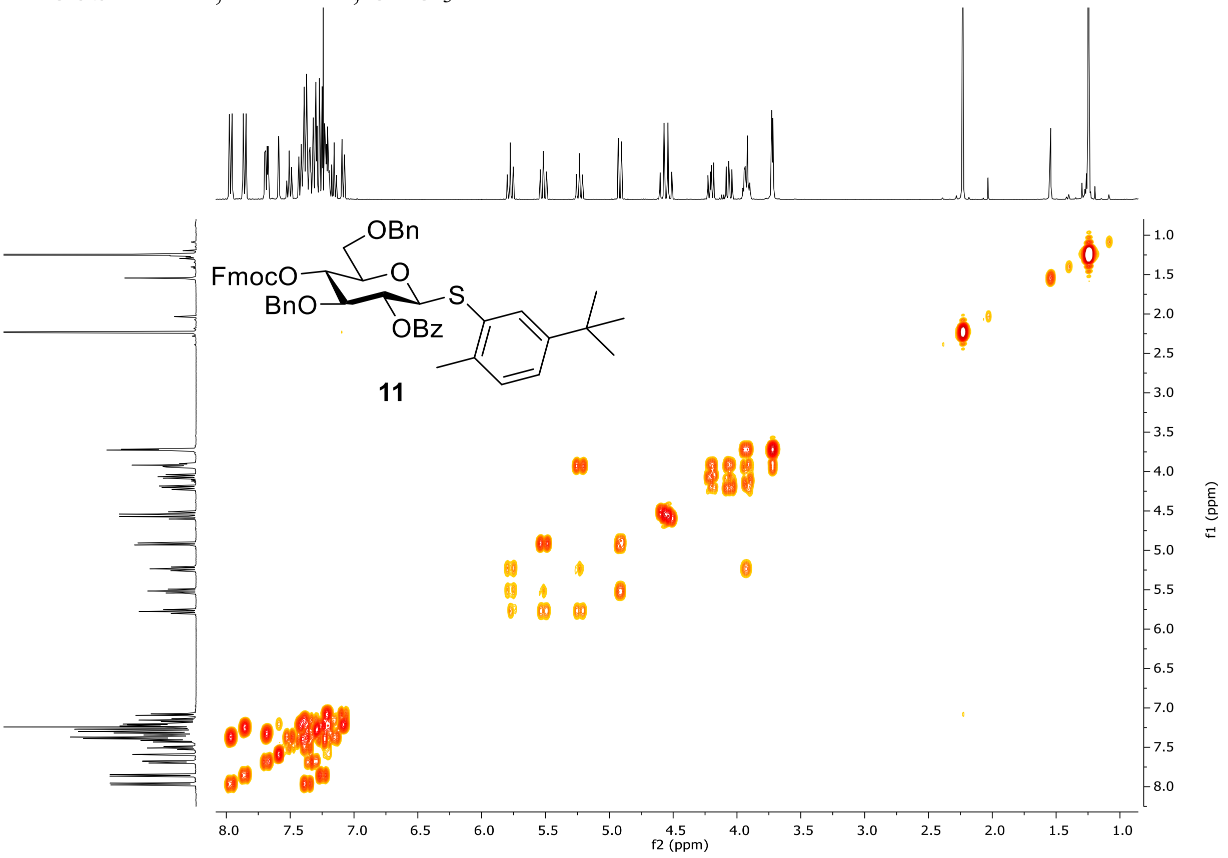

^{1}H-^{13}C-HSQC NMR, 400 MHz, $CDCl_3$

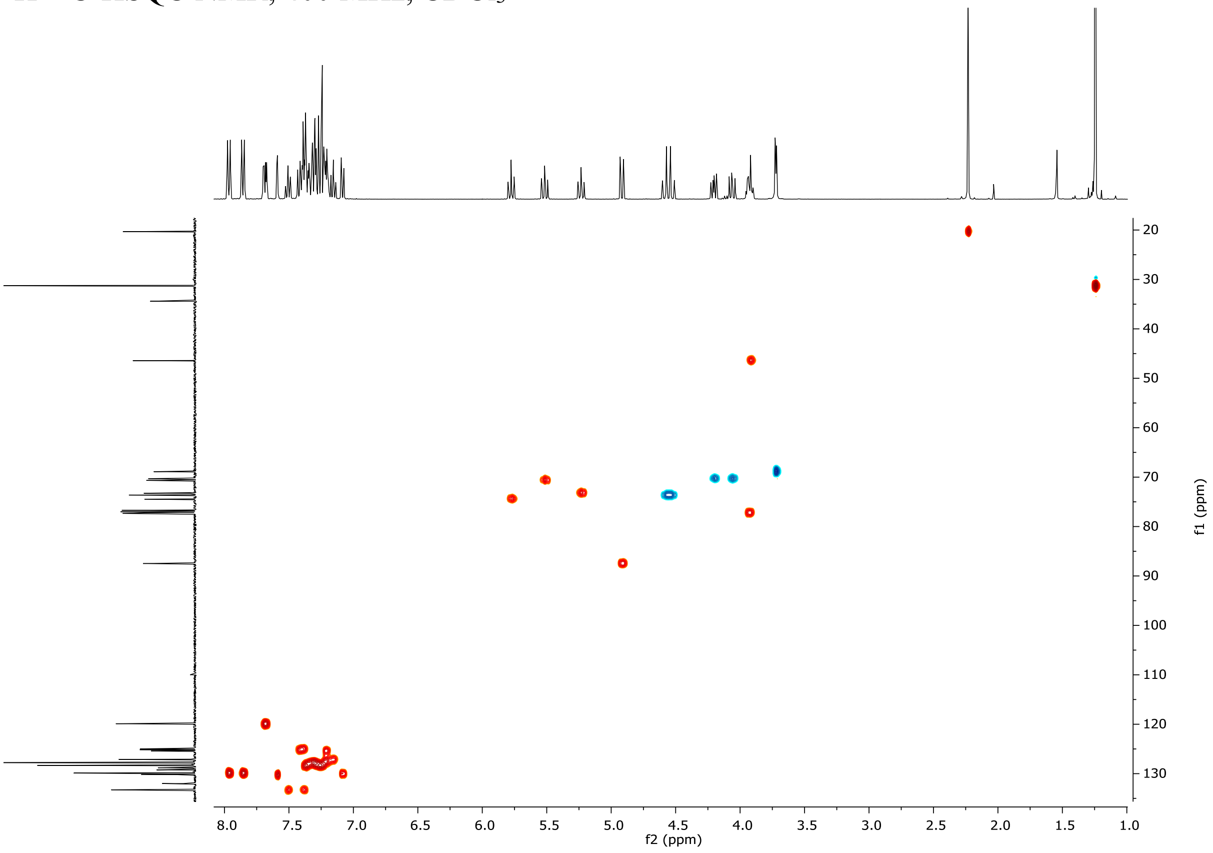

Supplementary Figure 20 | 2D NMR spectra of 11

^{1}H NMR, 400 MHz, $CDCl_3$

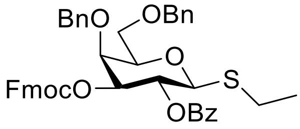

12

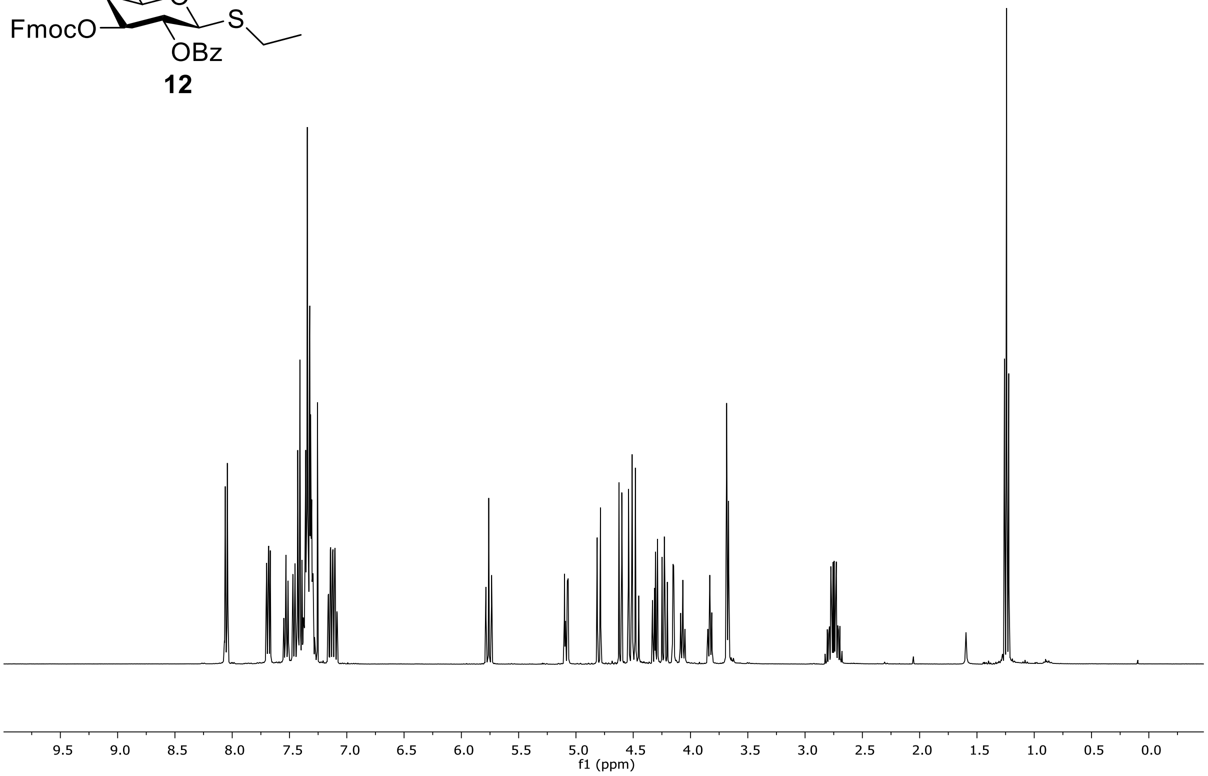

^{13}C NMR, 100 MHz, $CDCl_3$

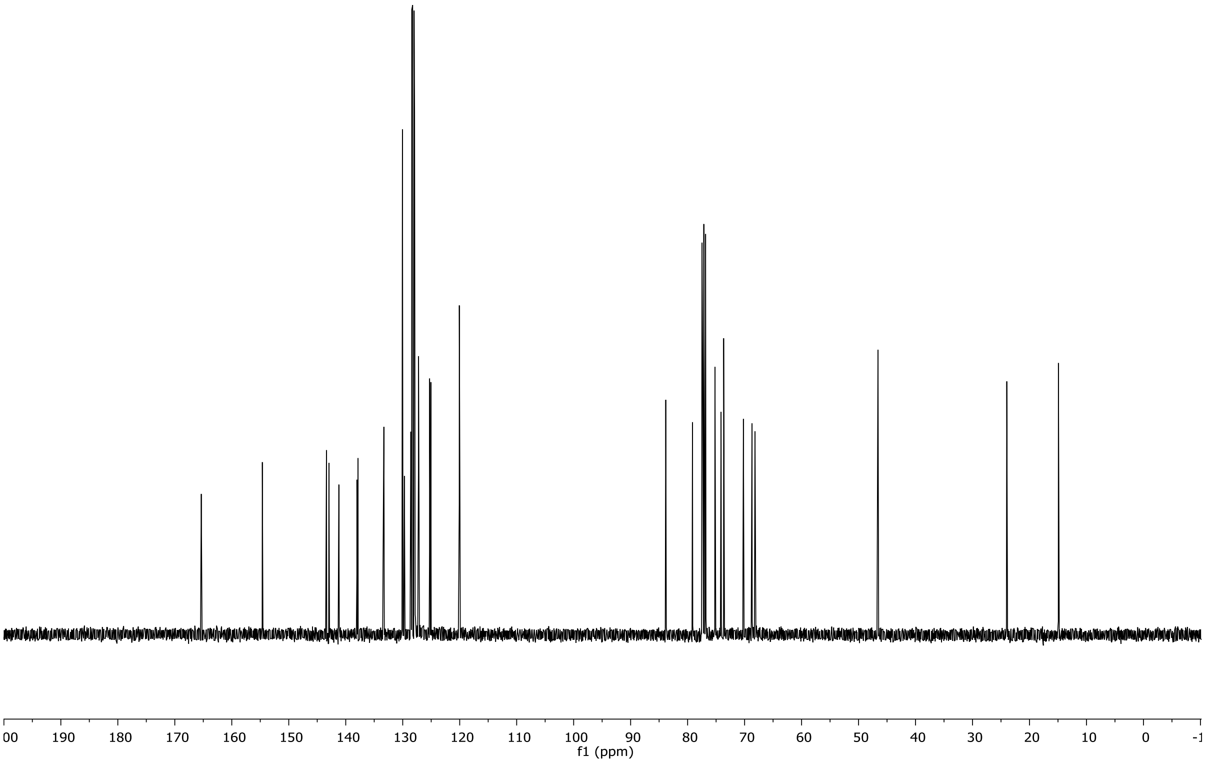

Supplementary Figure 21 | 1D NMR spectra of 12

^{1}H-COSY NMR, 400 MHz, $CDCl_3$

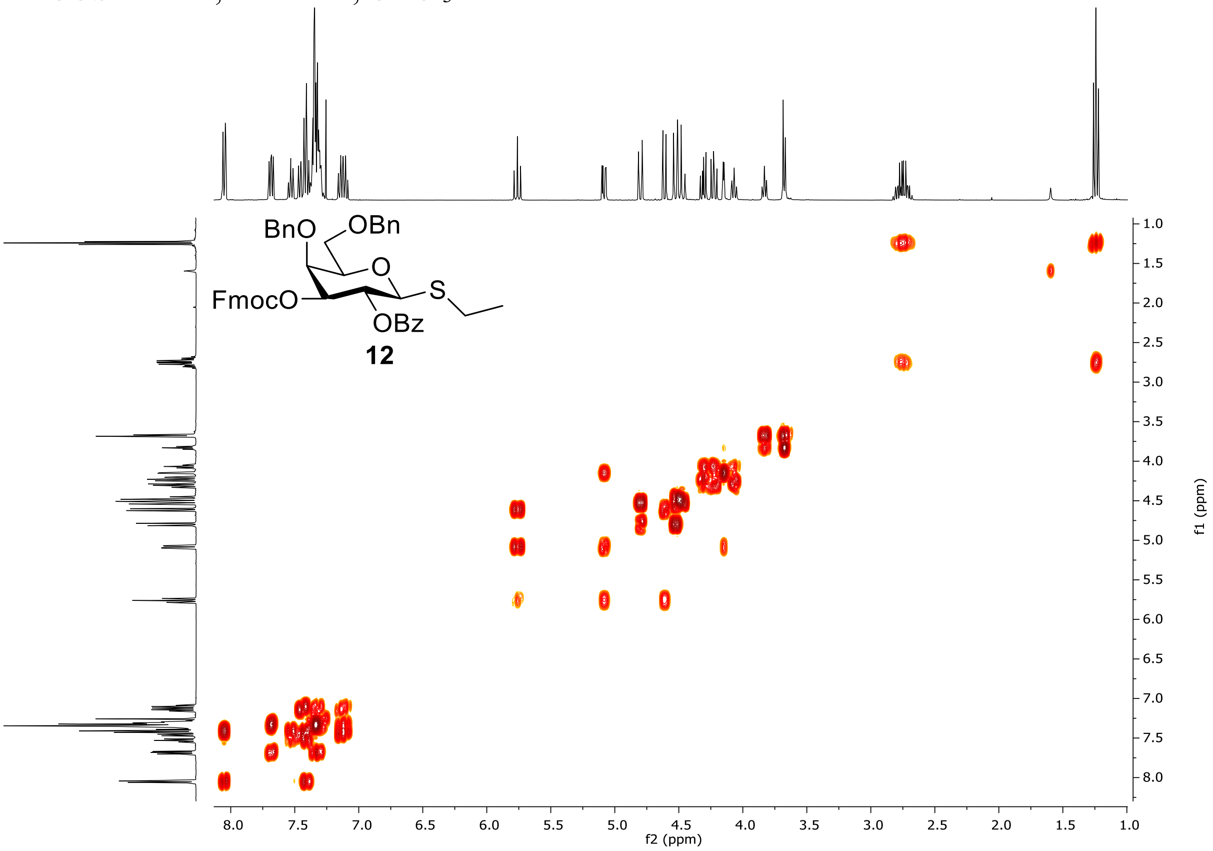

^{1}H-^{13}C-HSQC NMR, 400 MHz, $CDCl_3$

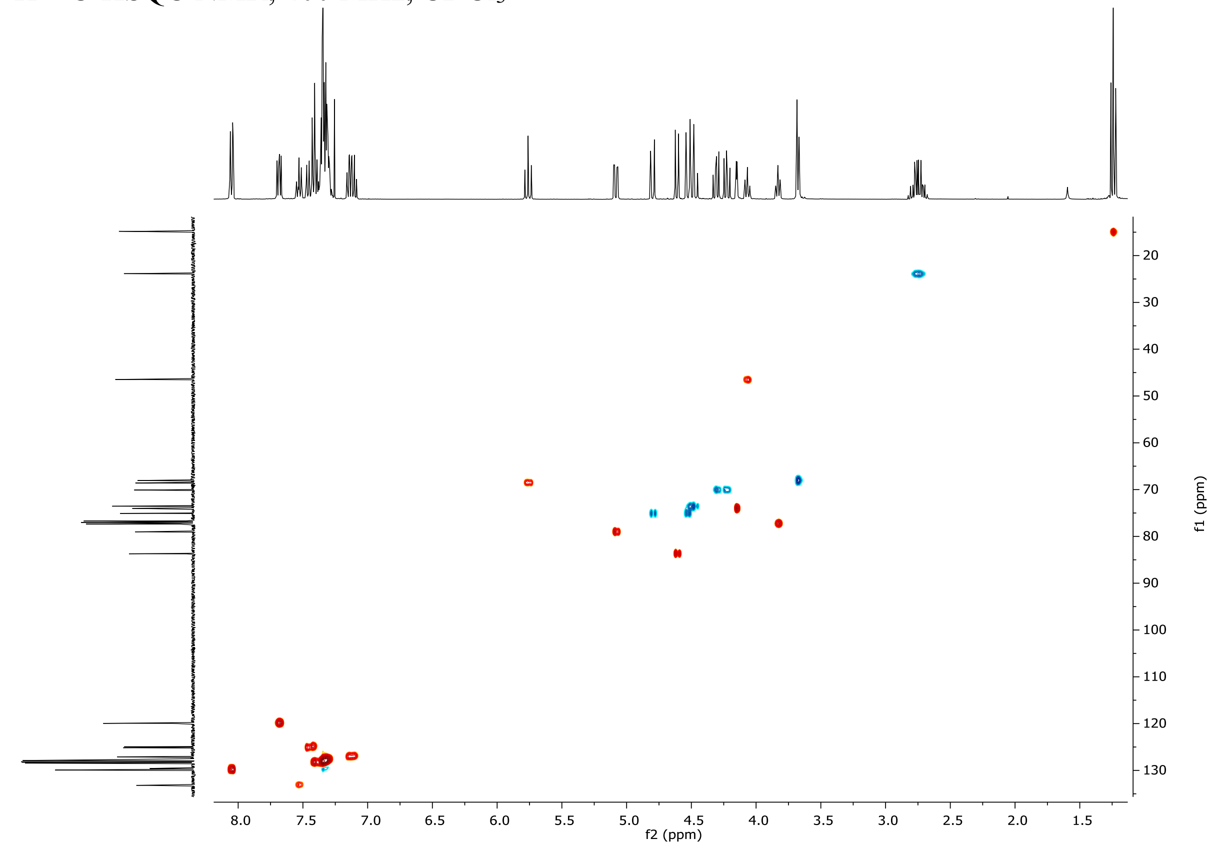

Supplementary Figure 22 | 2D NMR spectra of 12

^{1}H NMR, 400 MHz, $CDCl_3$

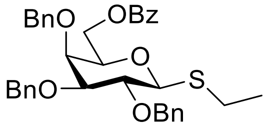

13c

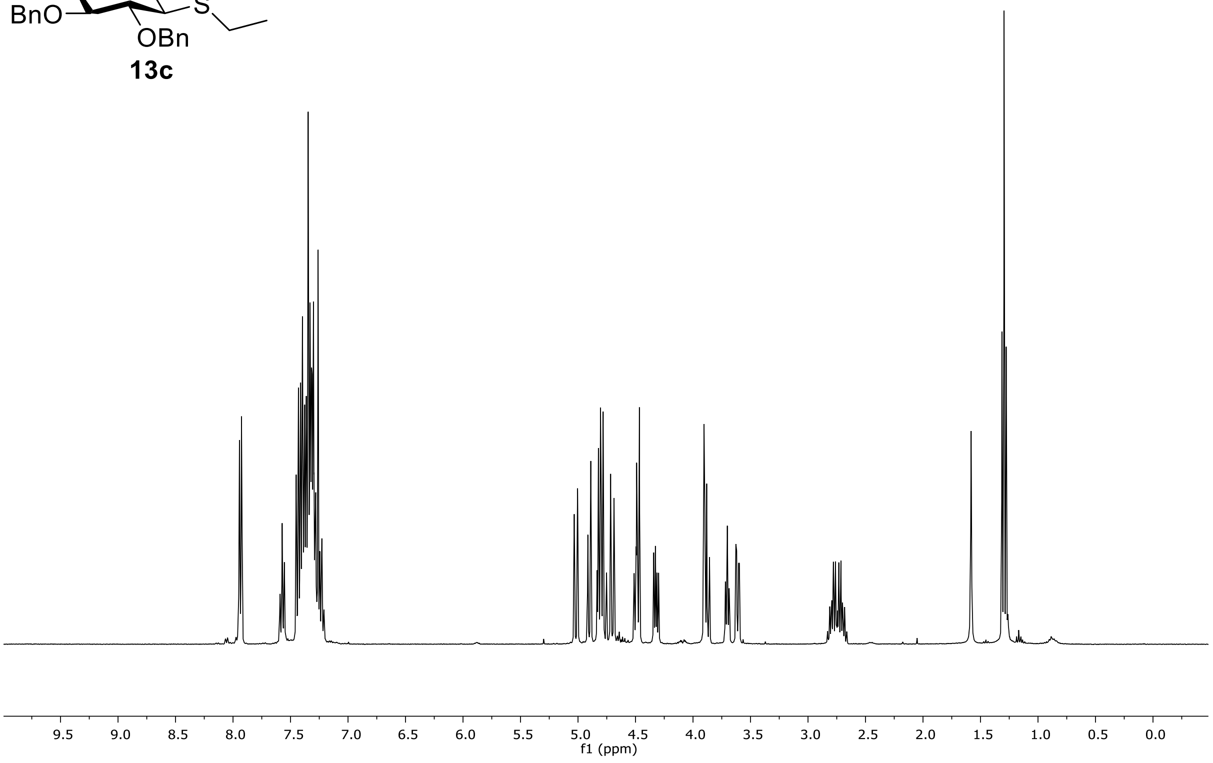

^{13}C NMR, 100 MHz, $CDCl_3$

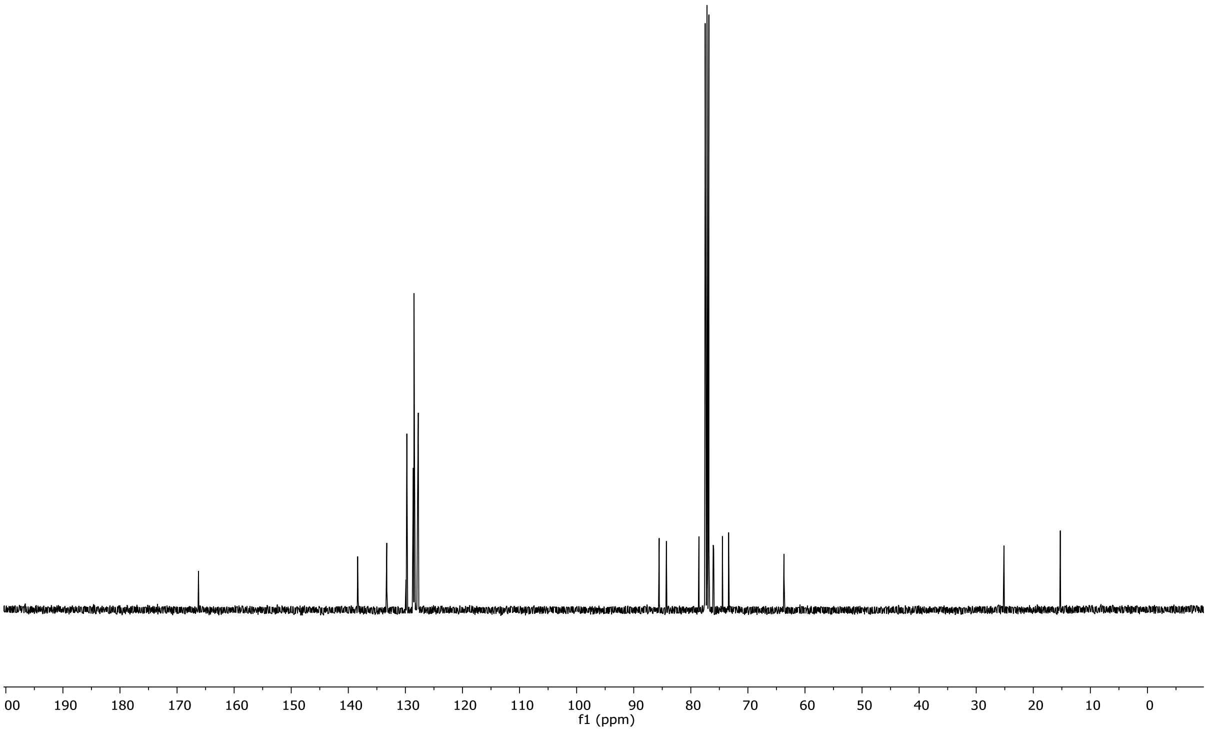

Supplementary Figure 23 | 1D NMR spectra of 13c

^{1}H-COSY NMR, 400 MHz, $CDCl_3$

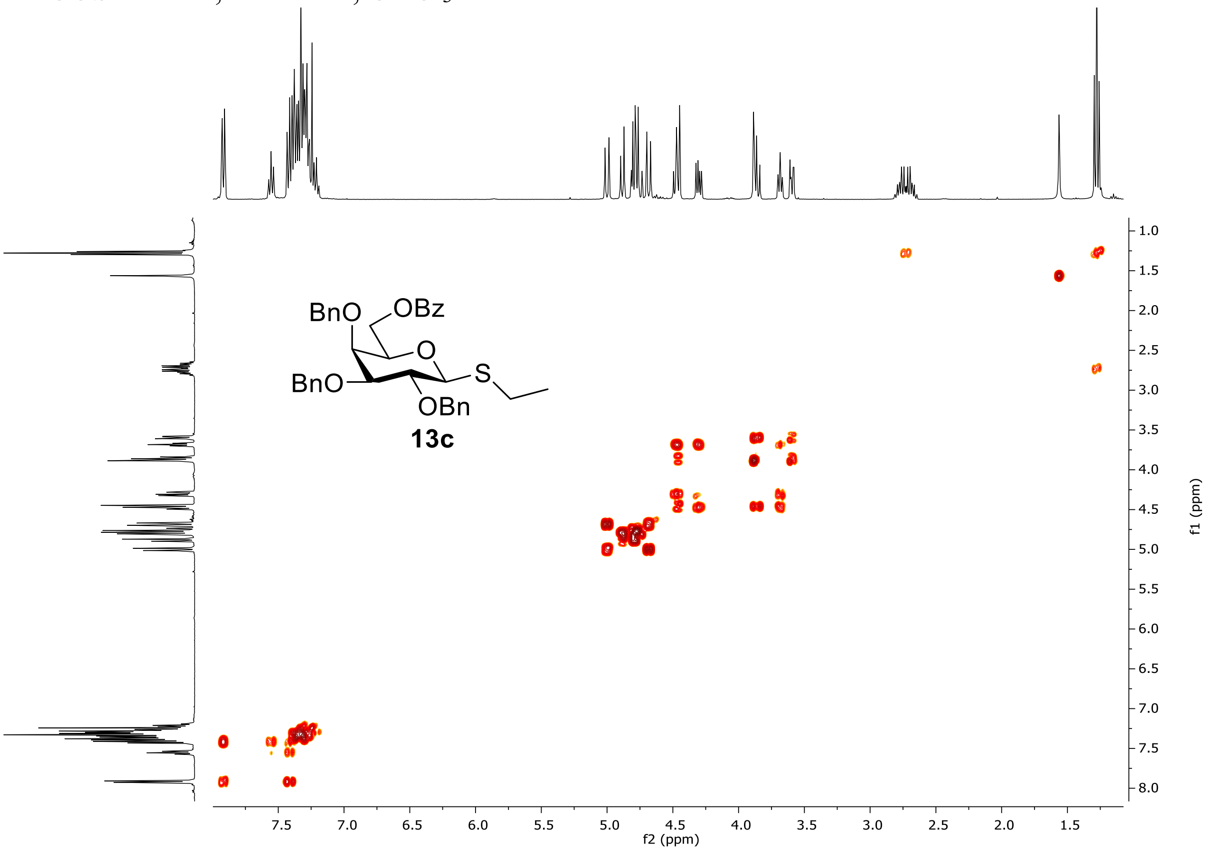

^{1}H-^{13}C-HSQC NMR, 400 MHz, $CDCl_3$

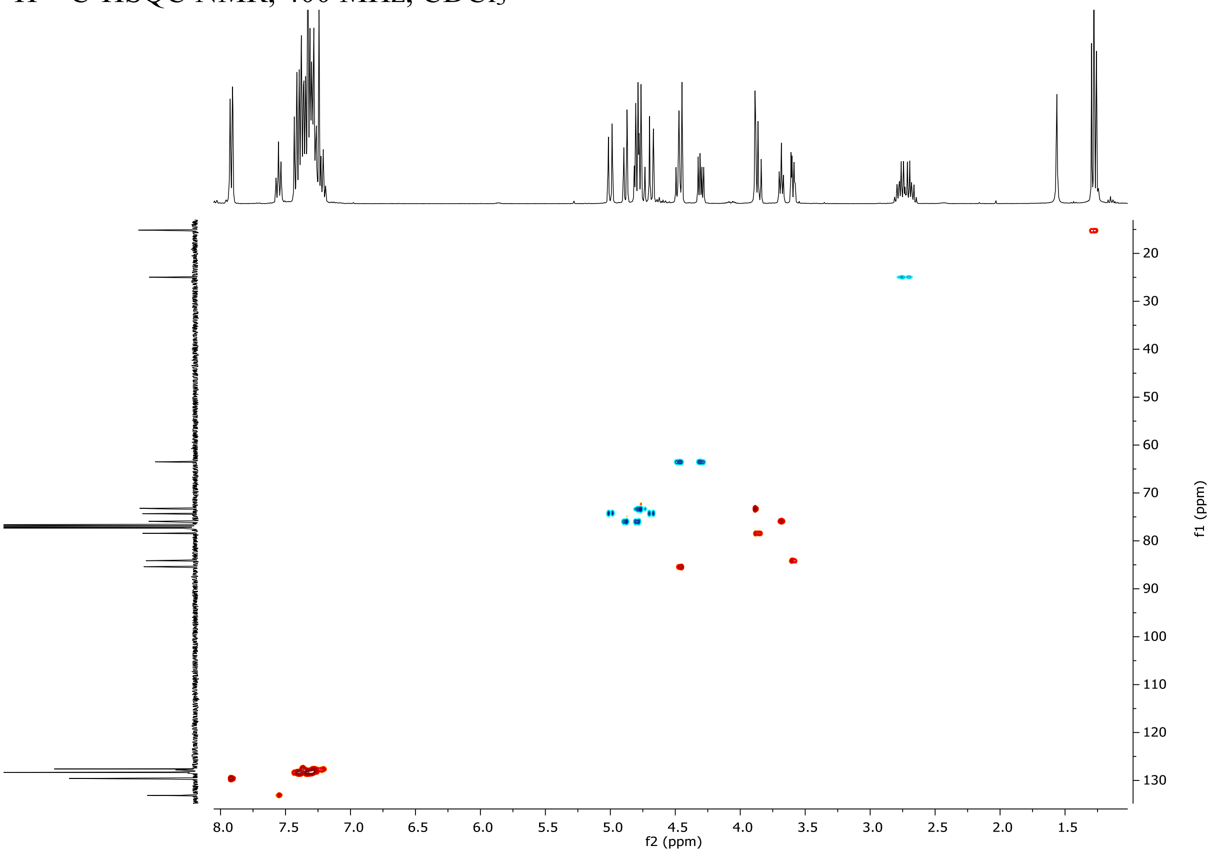

Supplementary Figure 24 | 2D NMR spectra of 13c

^{1}H NMR, 400 MHz, $CDCl_3$

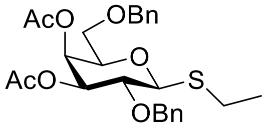

13f

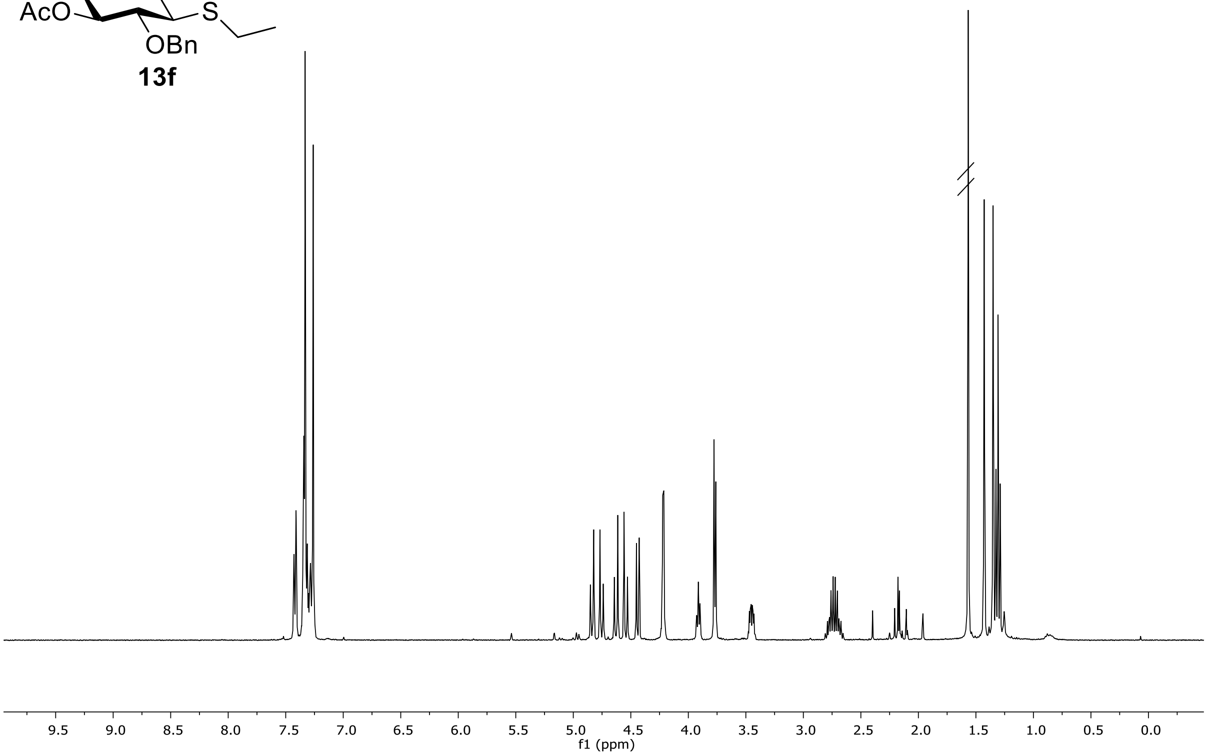

^{13}C NMR, 100 MHz, $CDCl_3$

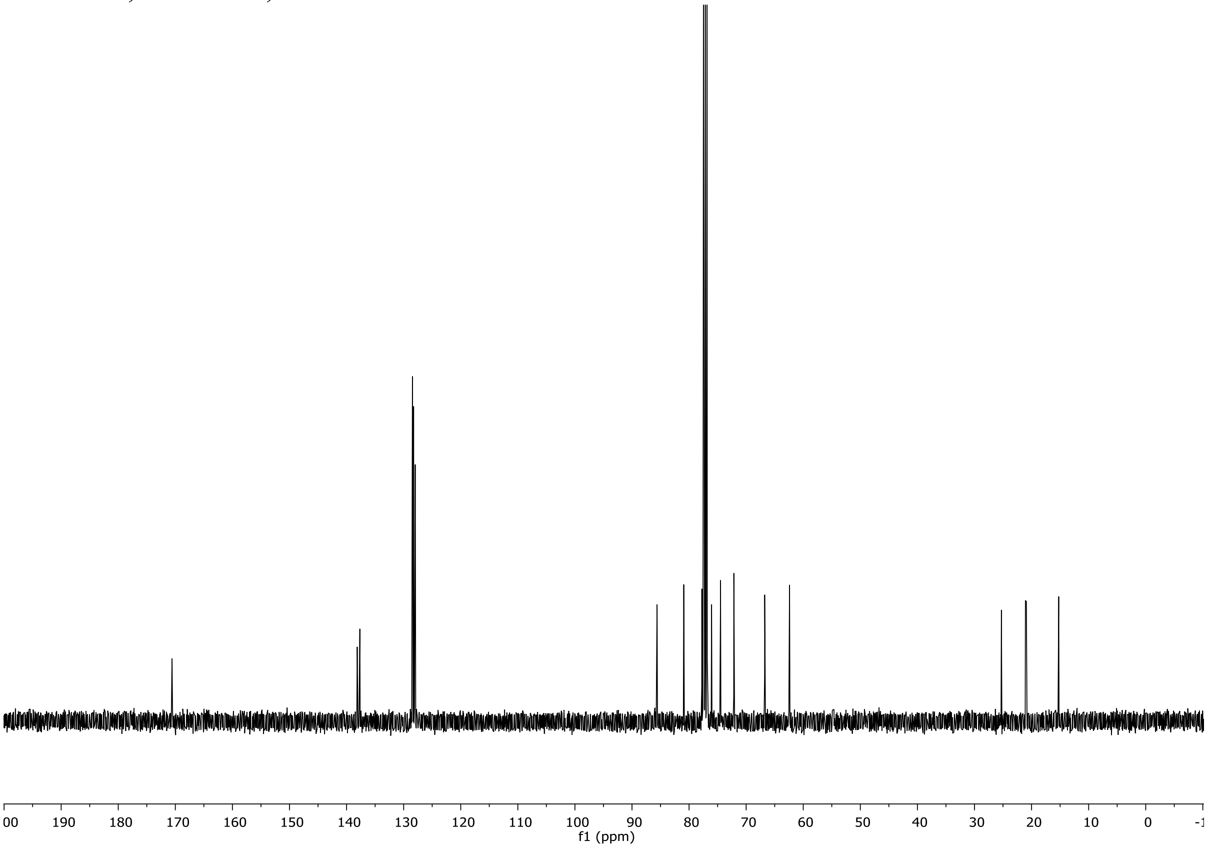

Supplementary Figure 25 | 1D NMR spectra of 13f

^{1}H-COSY NMR, 400 MHz, $CDCl_3$

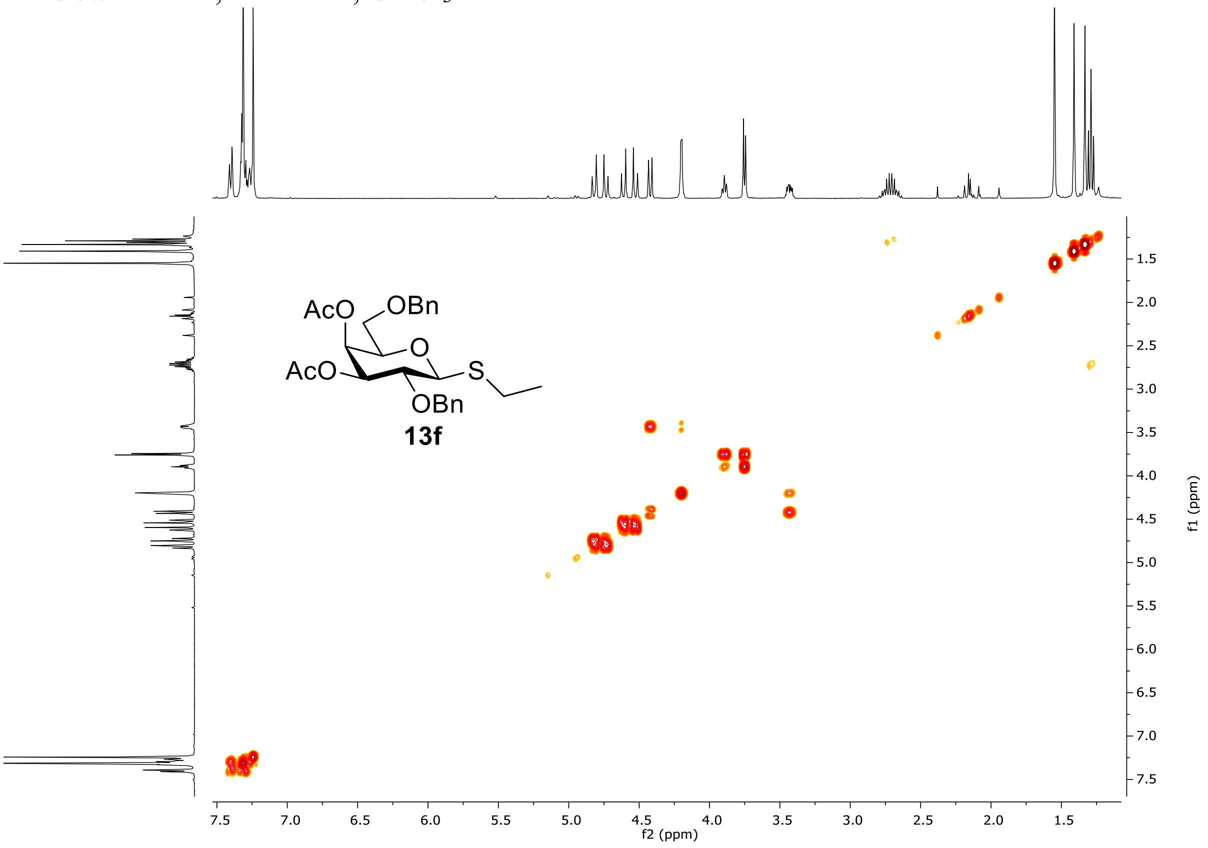

^{1}H-^{13}C-HSQC NMR, 400 MHz, $CDCl_3$

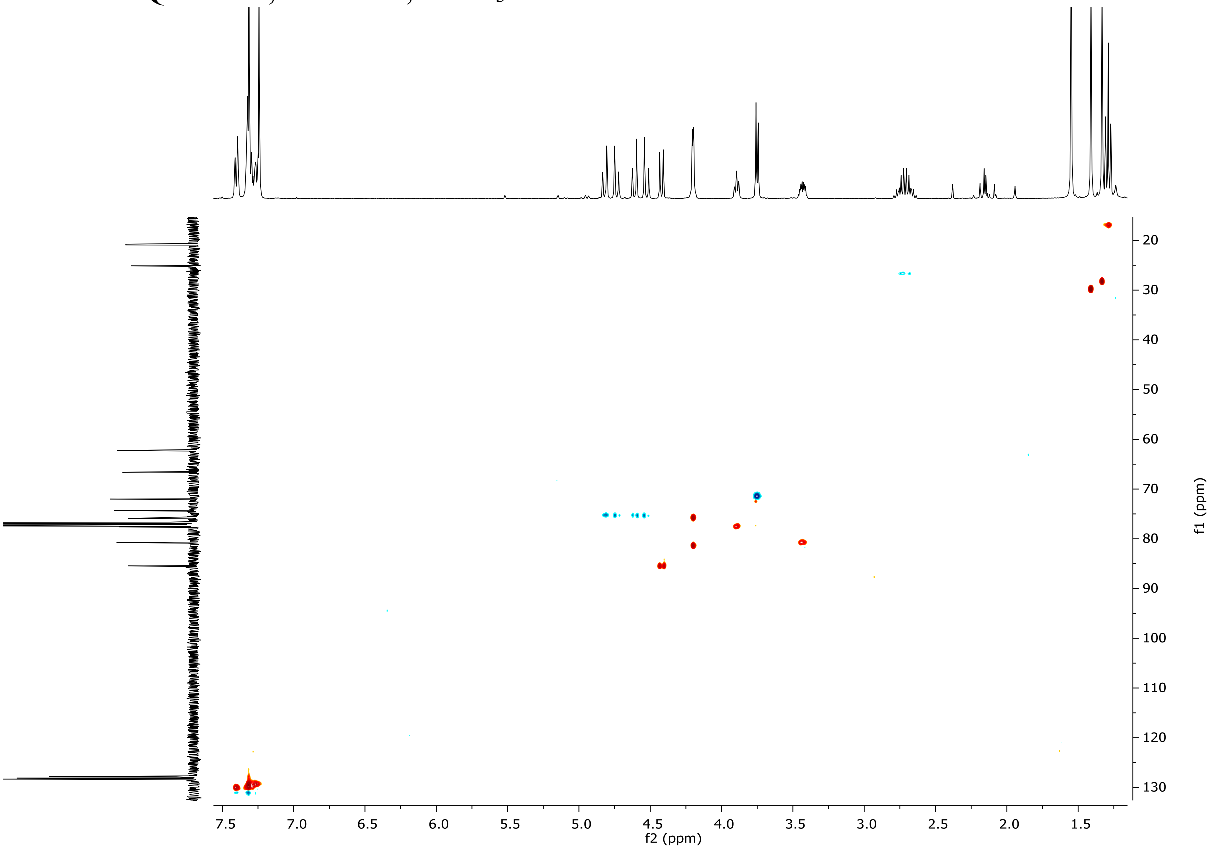

Supplementary Figure 26 | 2D NMR spectra of 13c

^{1}H NMR, 400 MHz, $CDCl_3$

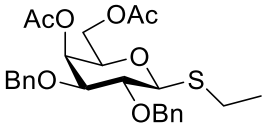

13g

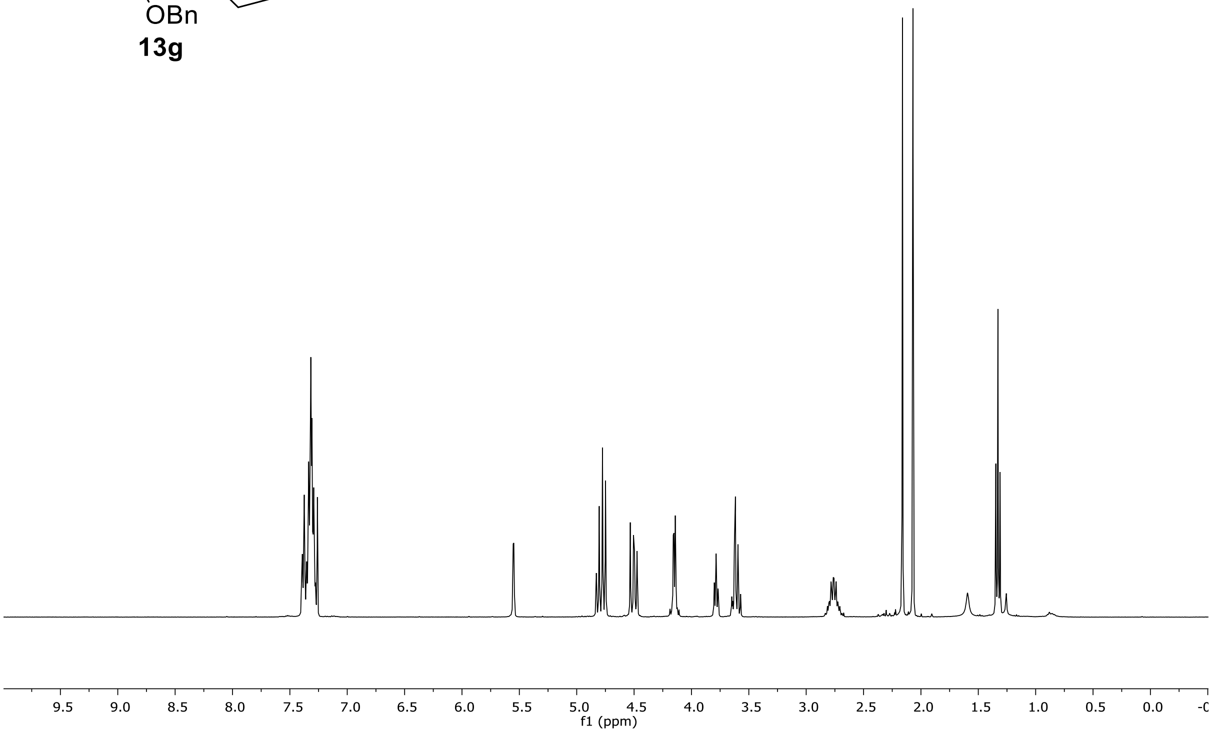

^{13}C NMR, 100 MHz, $CDCl_3$

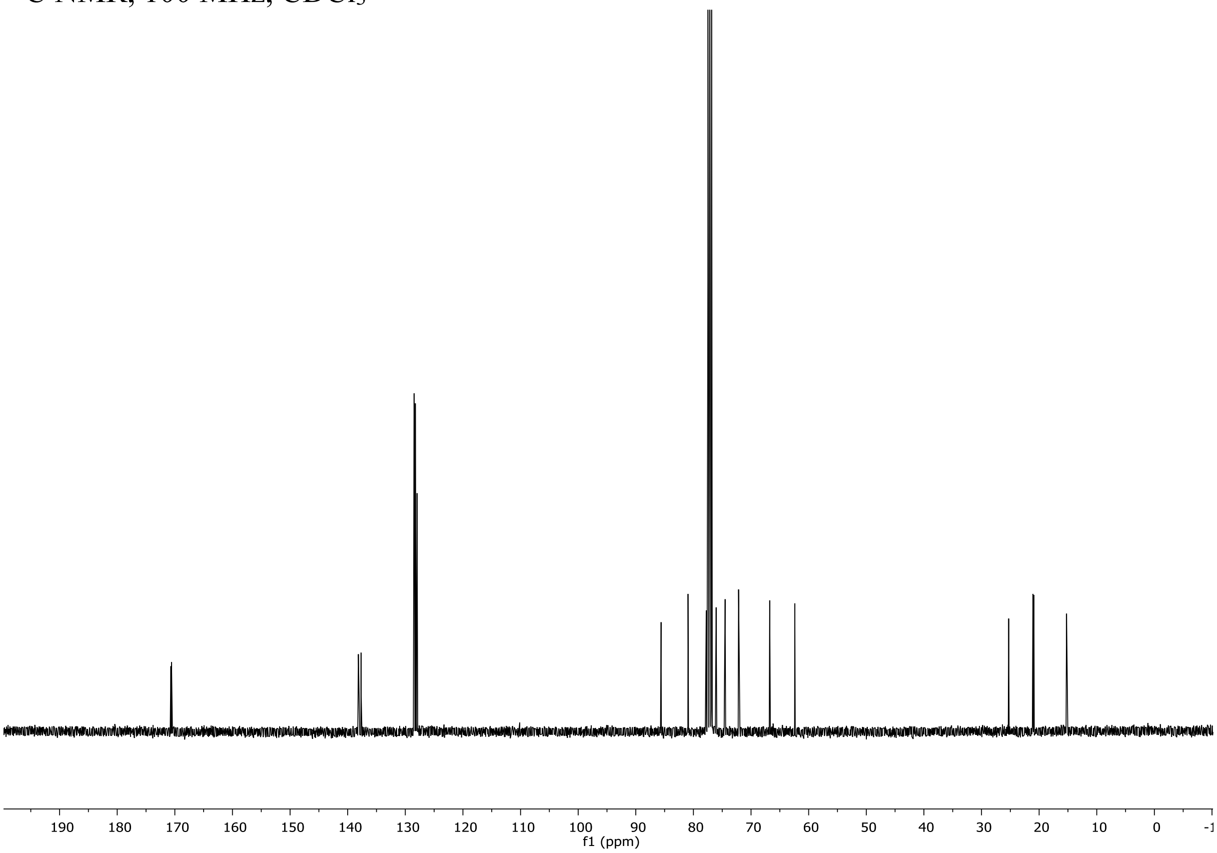

Supplementary Figure 27 | 1D NMR spectra of 13g

^{1}H-COSY NMR, 400 MHz, $CDCl_3$

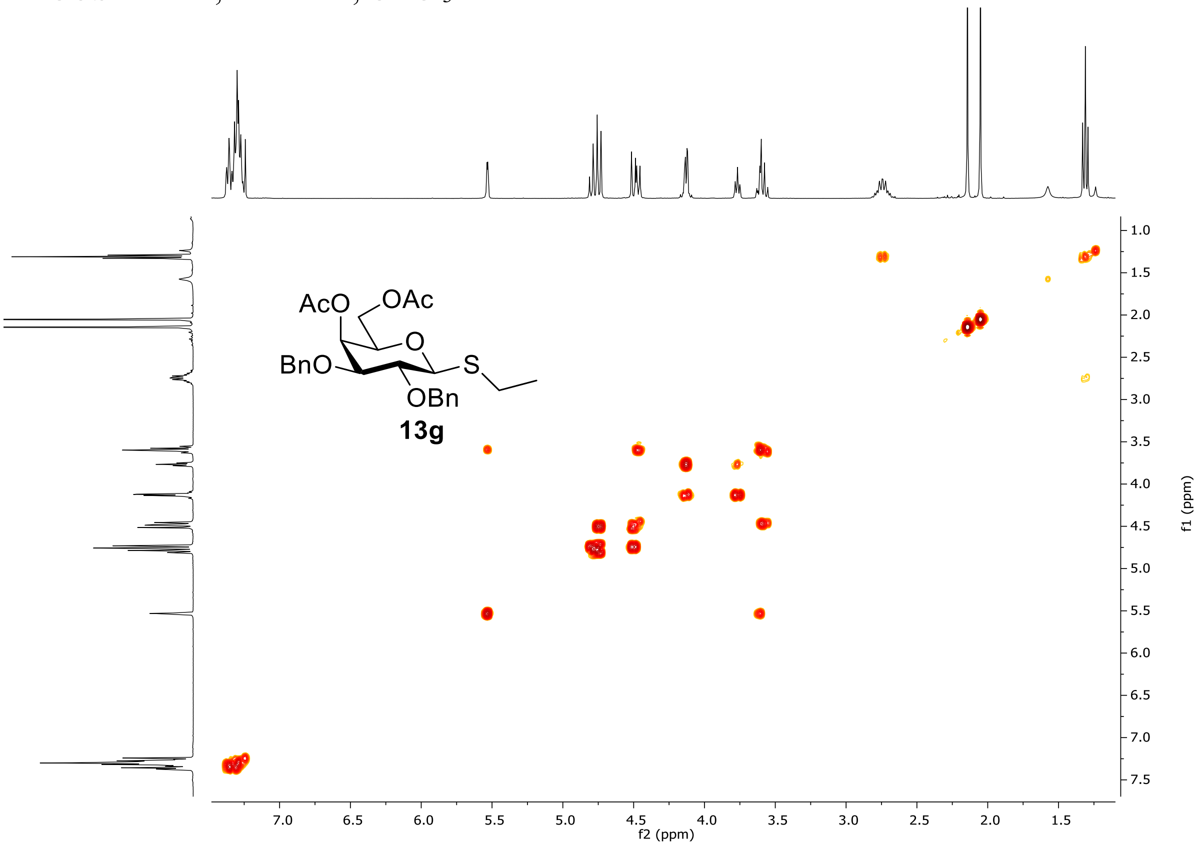

^{1}H-^{13}C-HSQC NMR, 400 MHz, $CDCl_3$

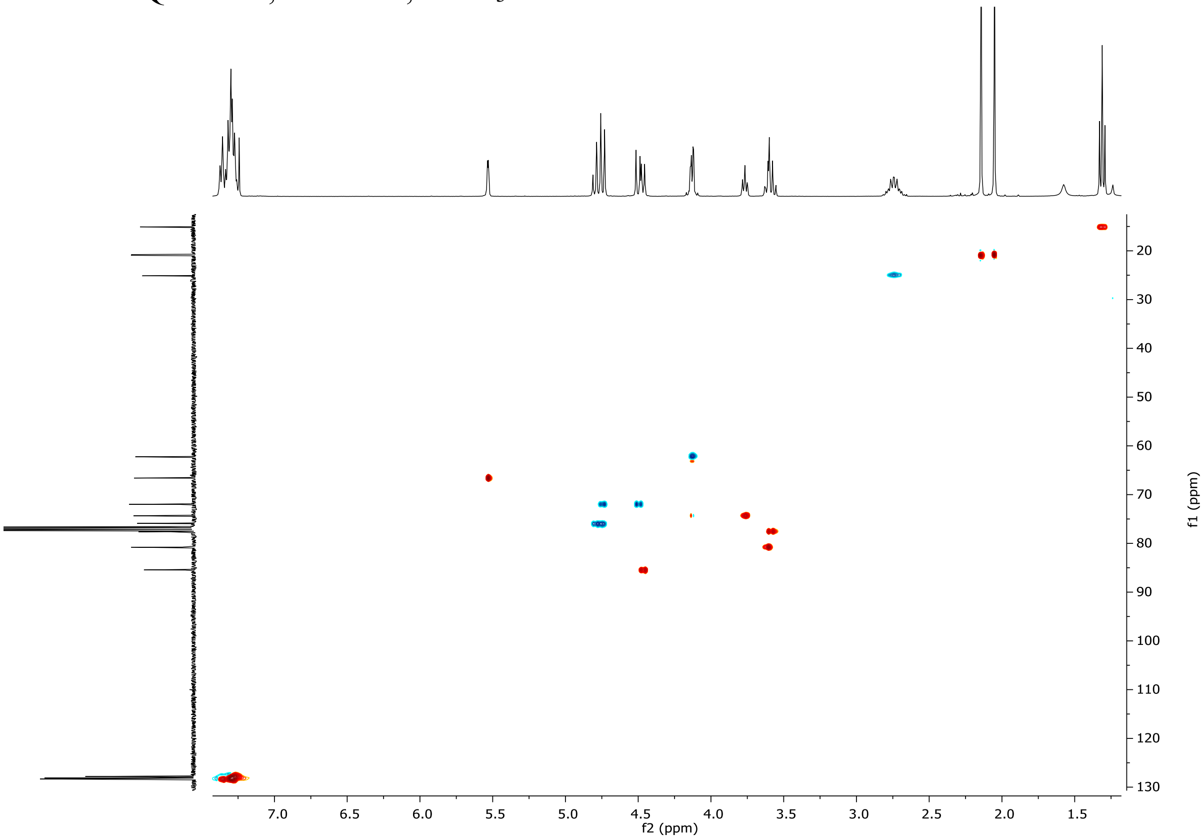

Supplementary Figure 28 | 2D NMR spectra of 13g

^{1}H NMR, 400 MHz, $CDCl_3$

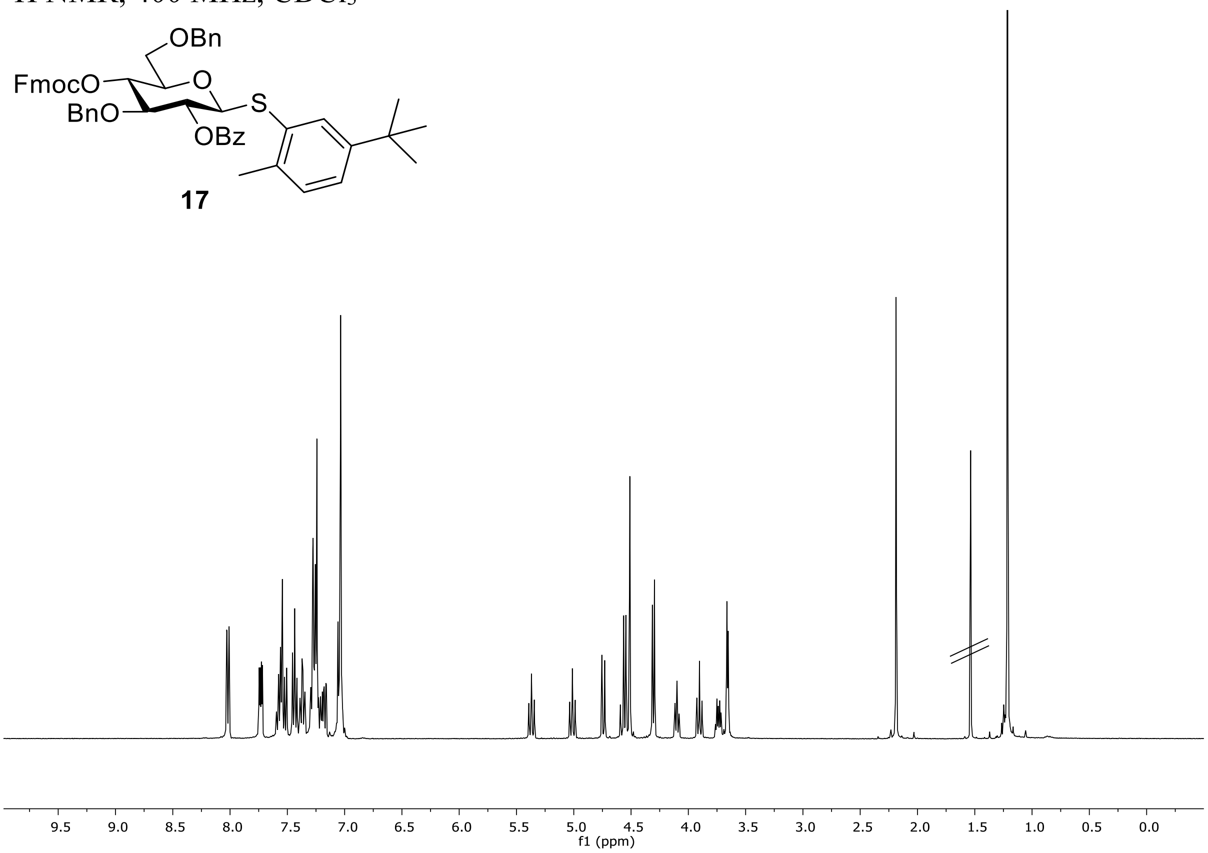

^{13}C NMR, 100 MHz, $CDCl_3$

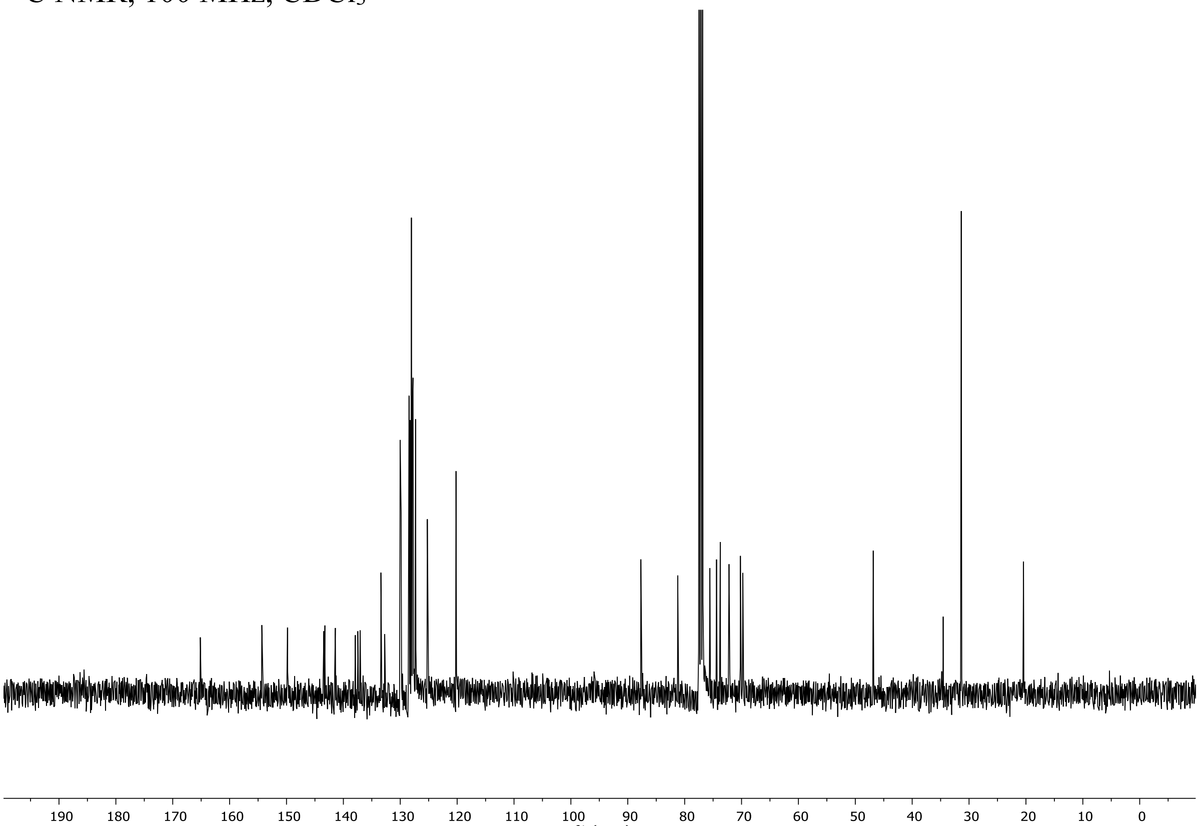

Supplementary Figure 29 | 1D NMR spectra of 17

^{1}H-COSY NMR, 400 MHz, $CDCl_3$

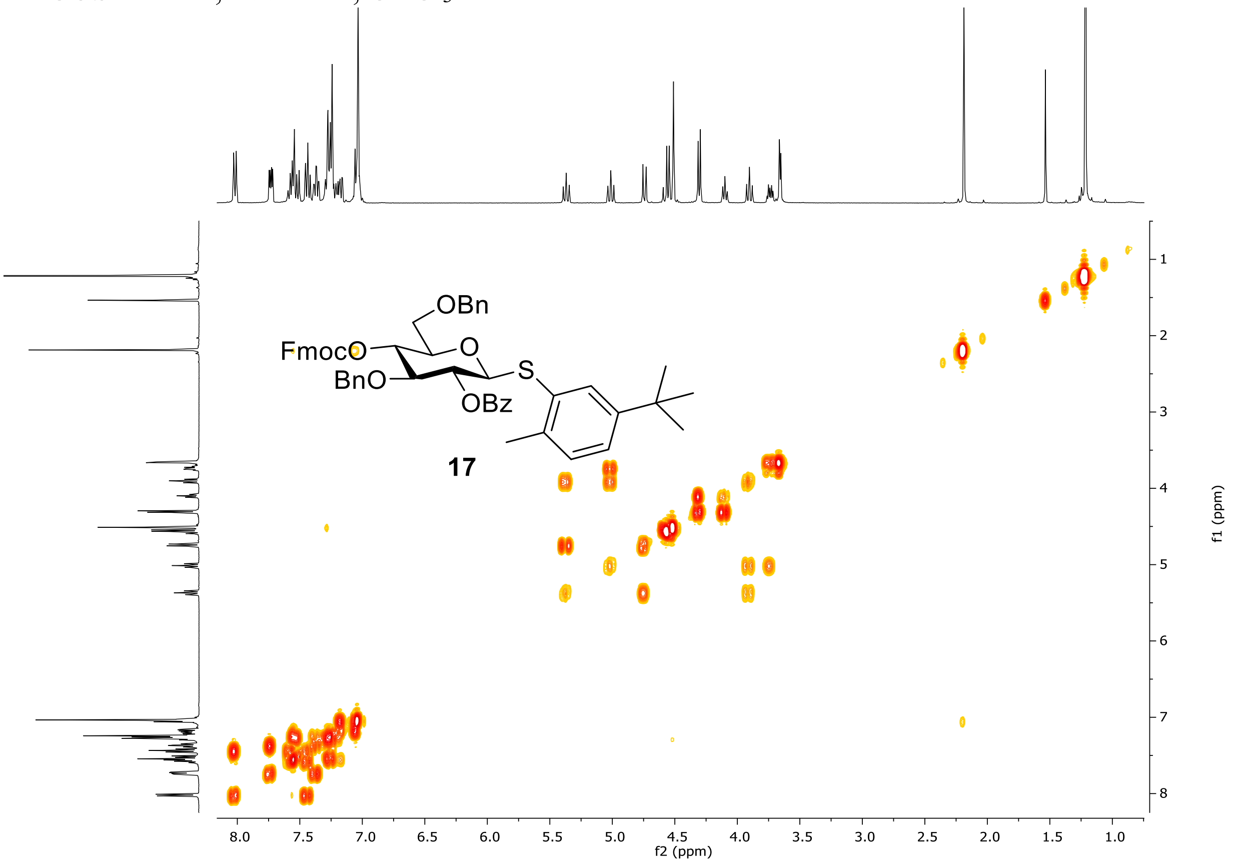

^{1}H-^{13}C-HSQC NMR, 400 MHz, $CDCl_3$

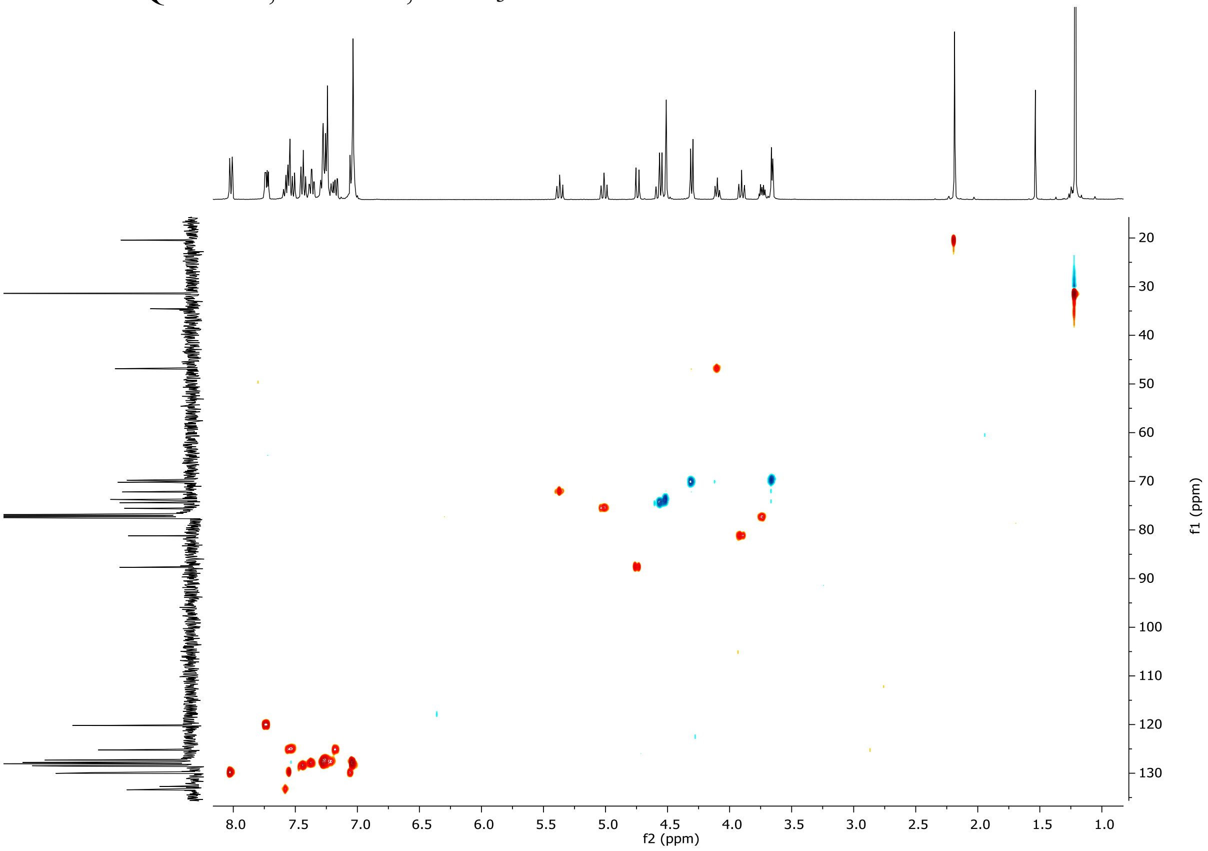

Supplementary Figure 30 | 2D NMR spectra of 17

^{1}H NMR, 400 MHz, $CDCl_3$

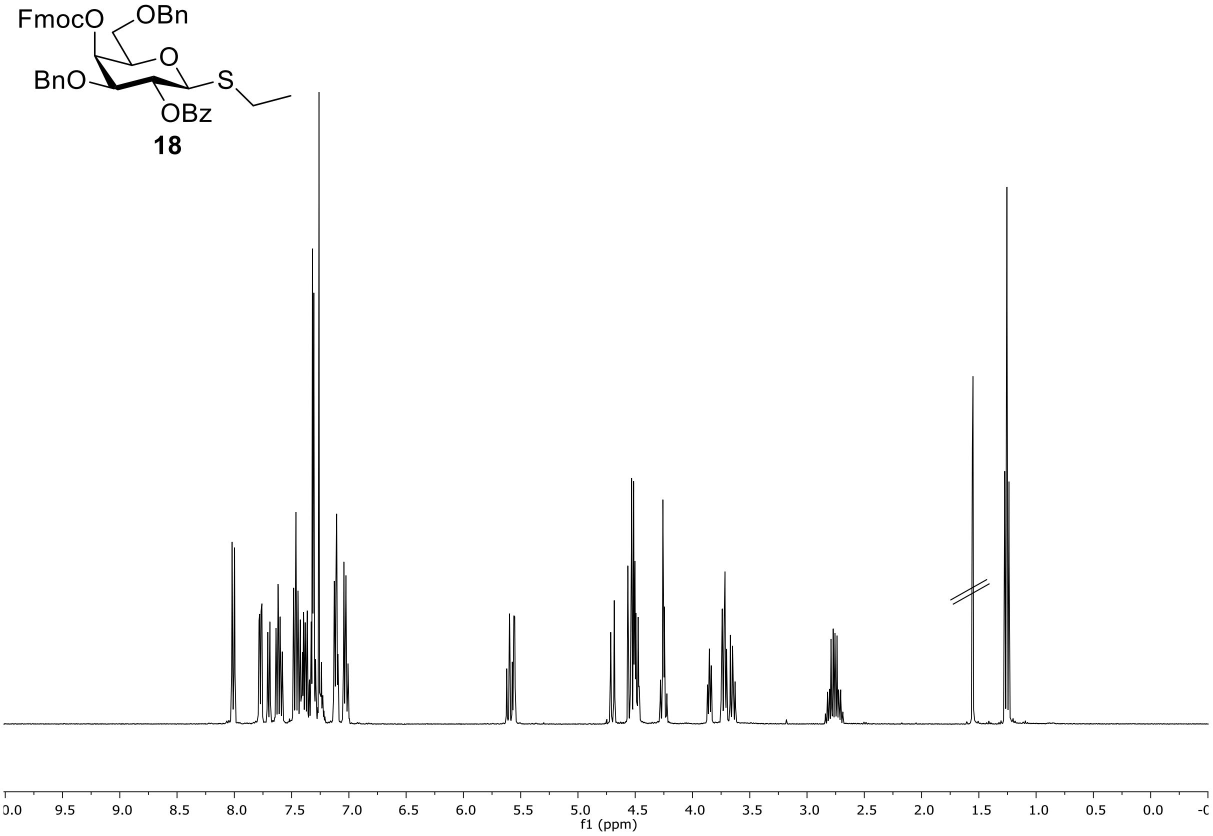

^{13}C NMR, 100 MHz, $CDCl_3$

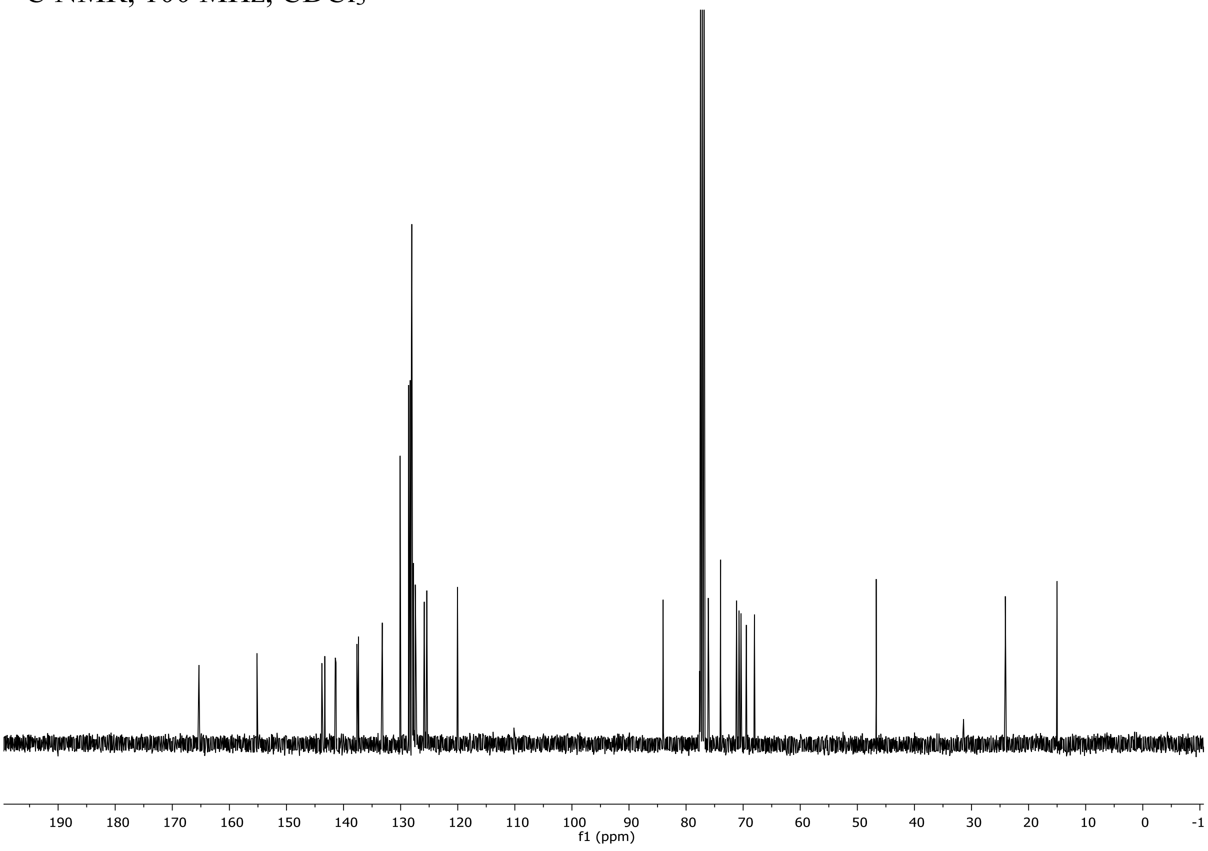

Supplementary Figure 31 | 1D NMR spectra of 18

^{1}H-COSY NMR, 400 MHz, $CDCl_3$

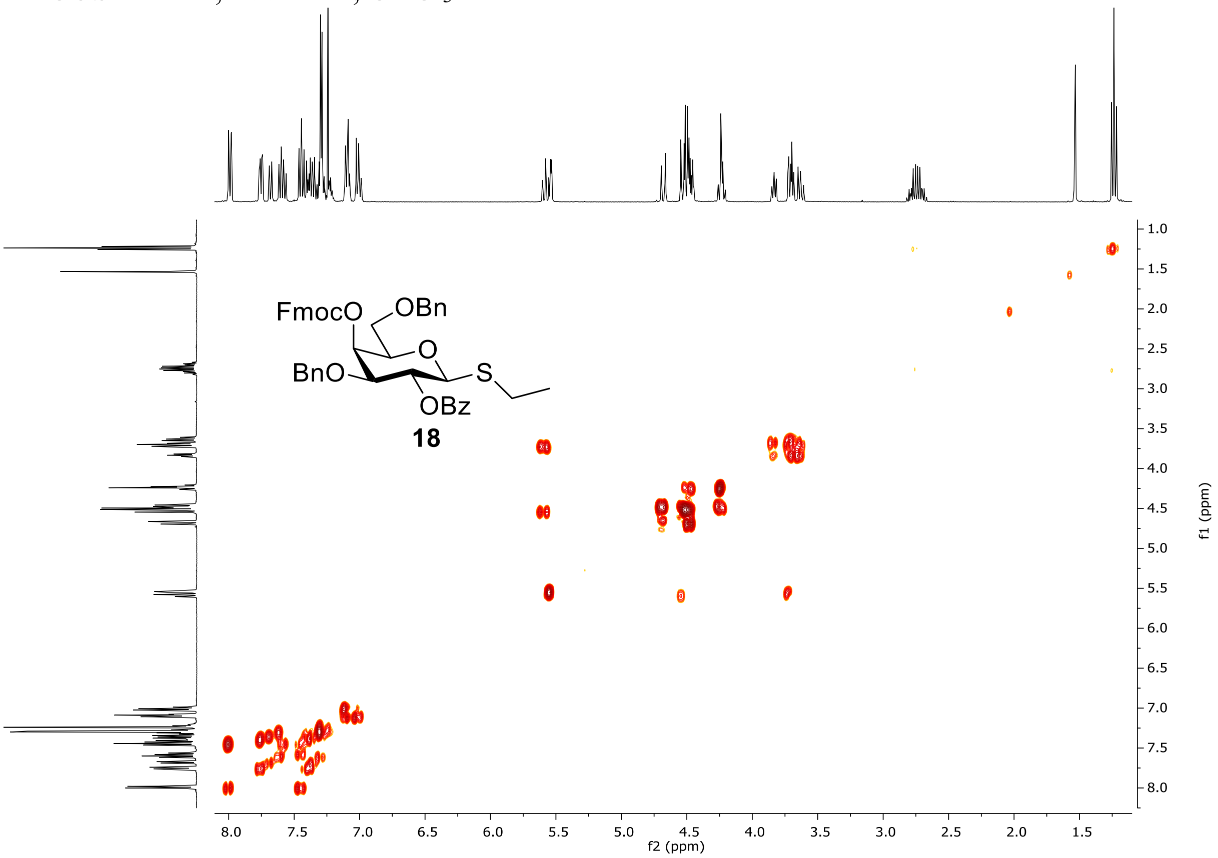

^{1}H-^{13}C-HSQC NMR, 400 MHz, $CDCl_3$

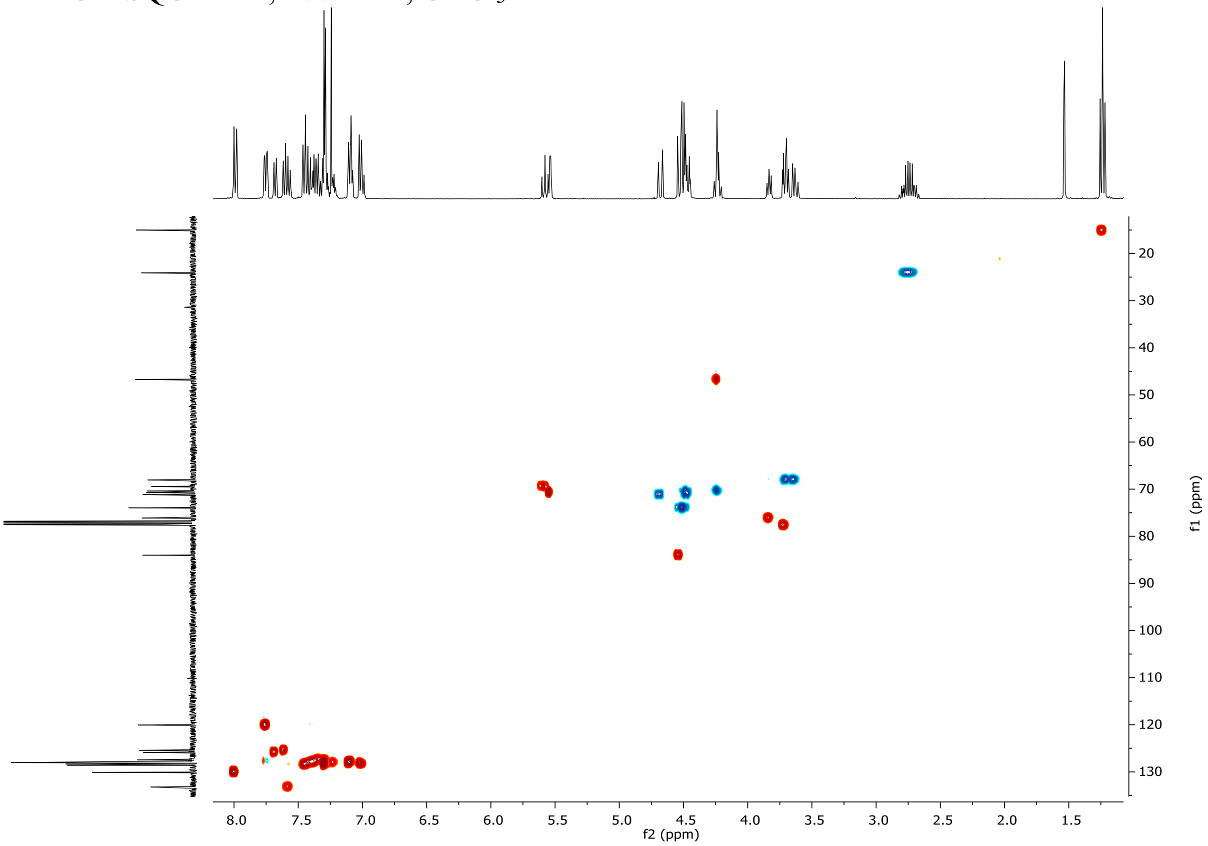

Supplementary Figure 32 | 2D NMR spectra of 18

^{1}H NMR, 400 MHz, $CDCl_3$

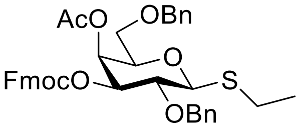

19

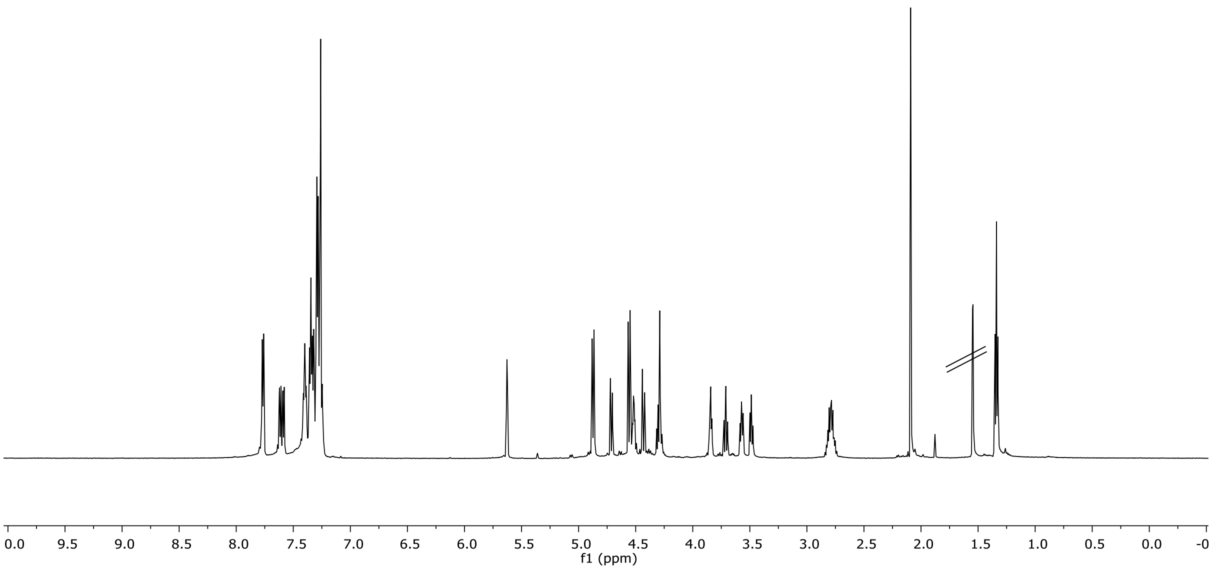

^{13}C NMR, 100 MHz, $CDCl_3$

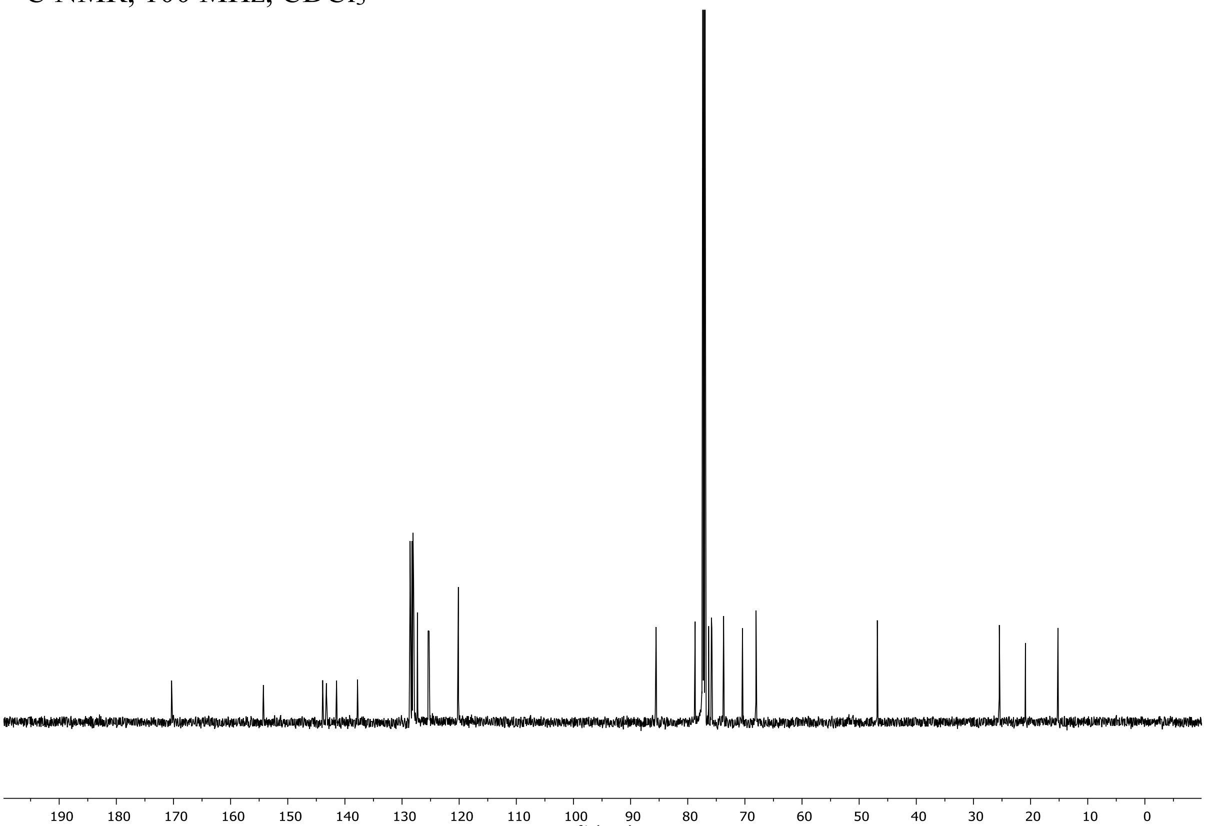

Supplementary Figure 33 | 1D NMR spectra of 19

^{1}H-COSY NMR, 400 MHz, $CDCl_3$

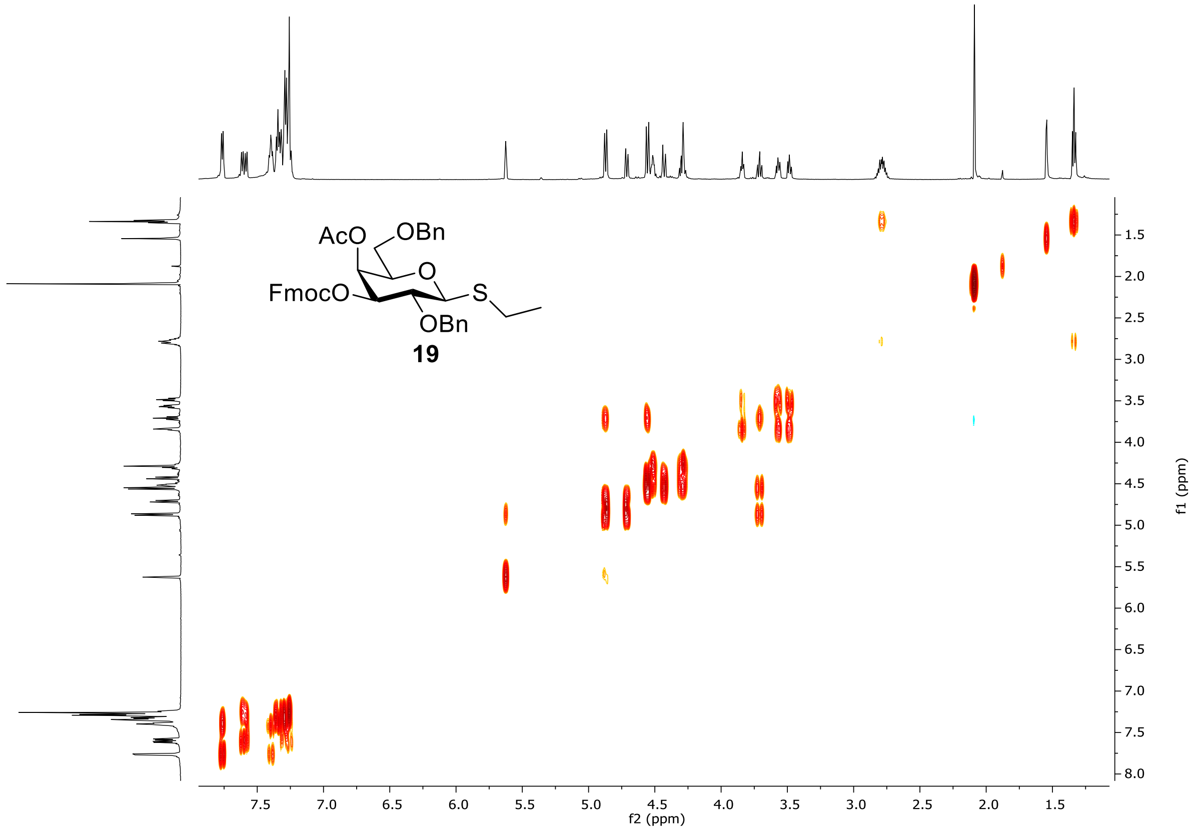

^{1}H-^{13}C-HSQC NMR, 400 MHz, $CDCl_3$

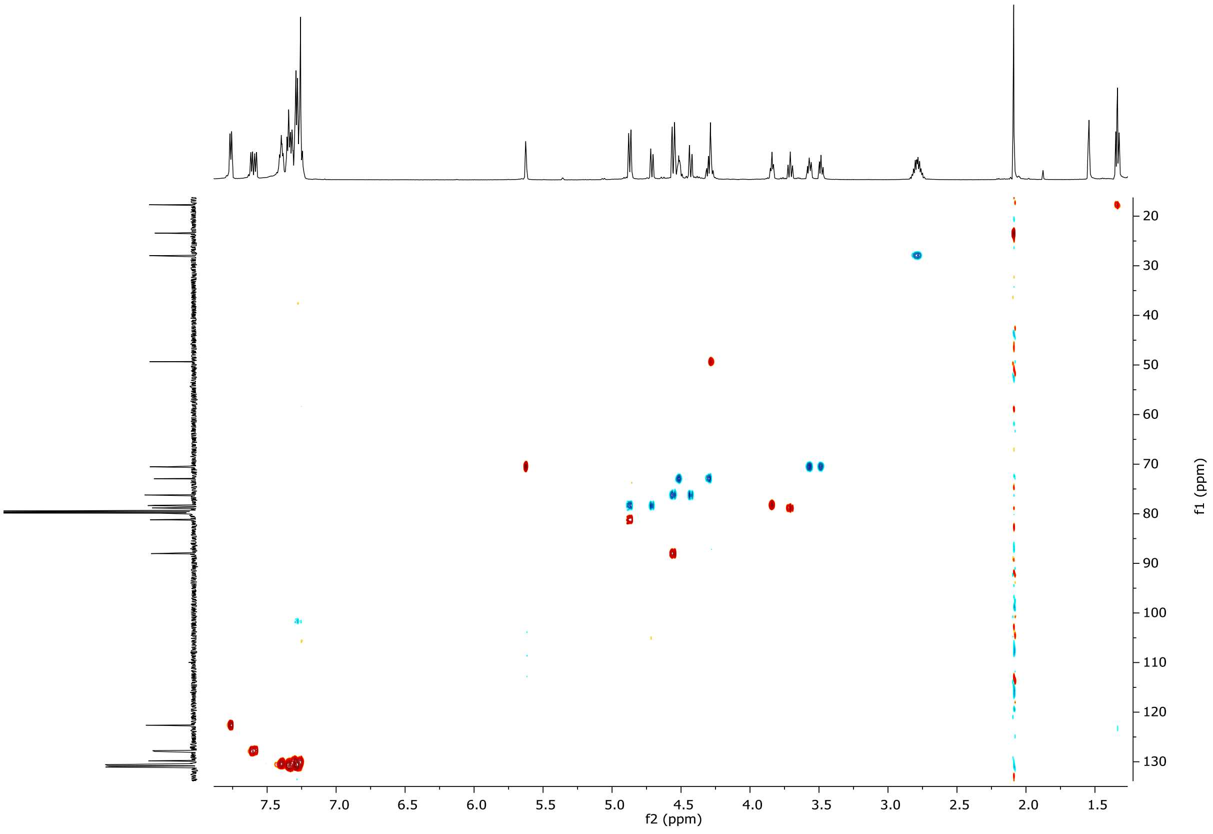

Supplementary Figure 34 | 2D NMR spectra of 19

^{1}H NMR, 400 MHz, $CDCl_3$

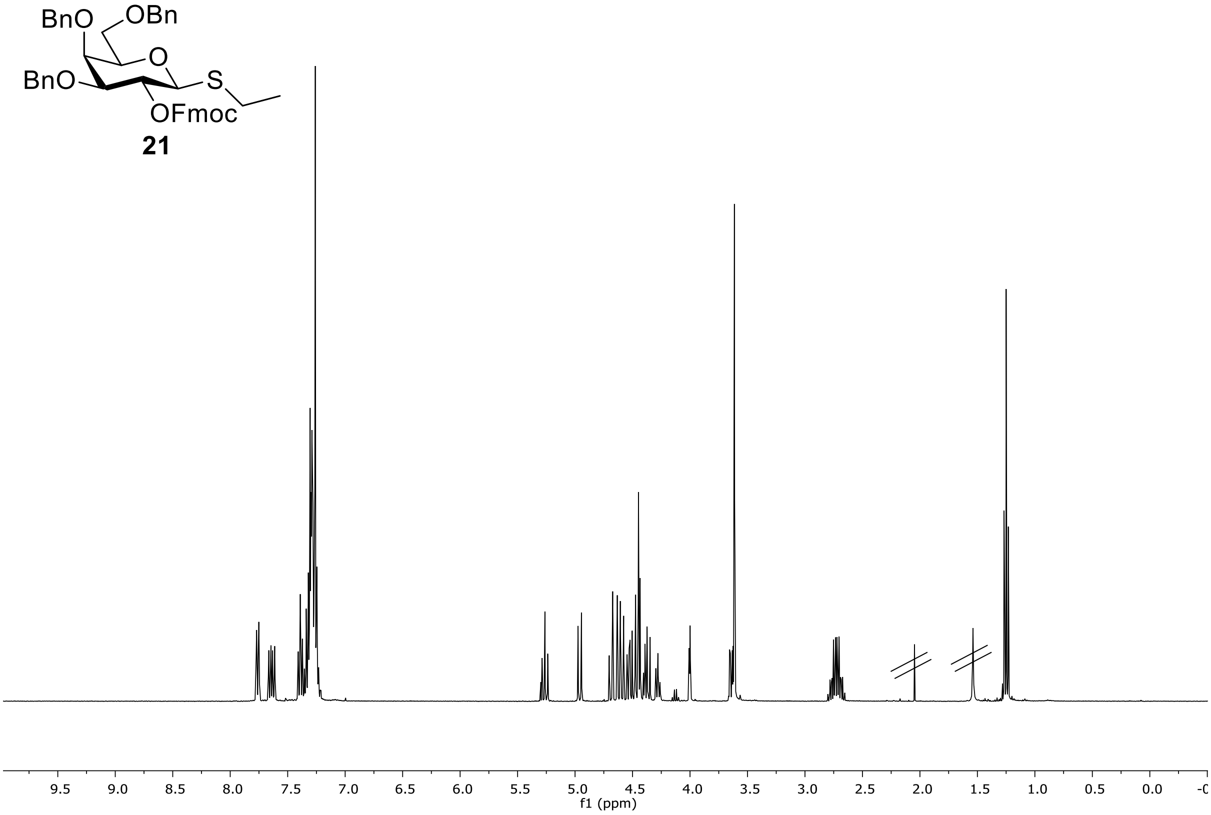

^{13}C NMR, 100 MHz, $CDCl_3$

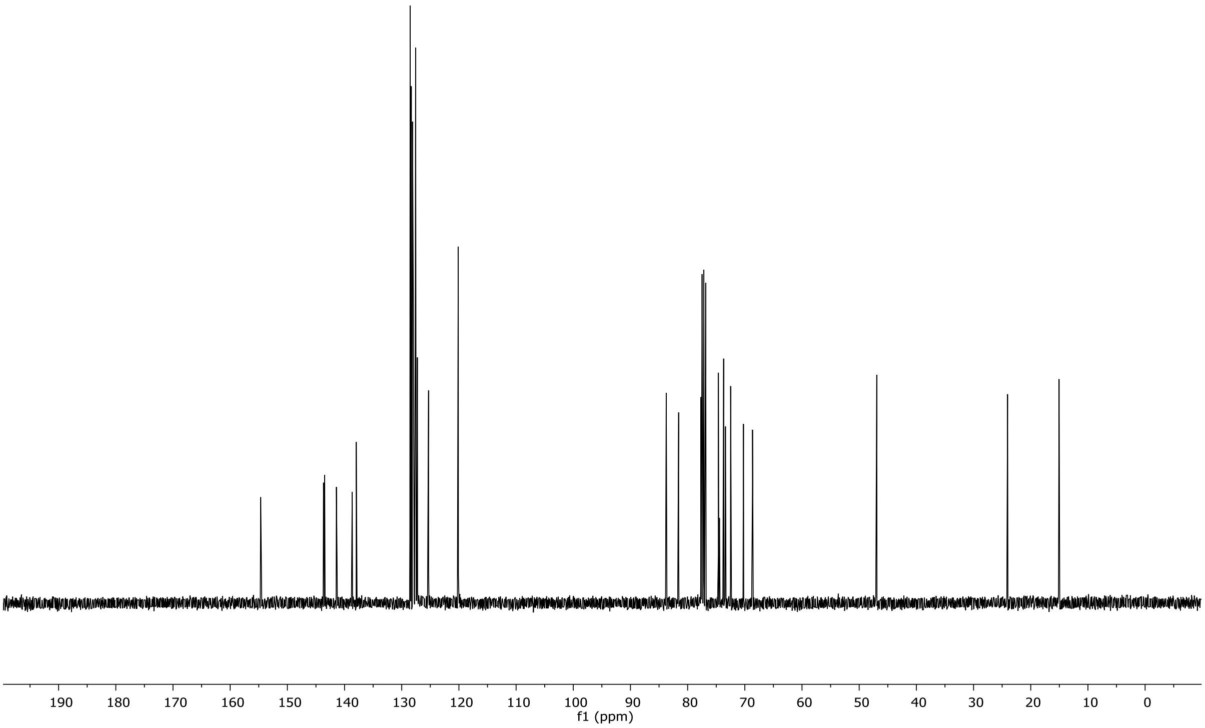

Supplementary Figure 35 | 1D NMR spectra of 21

^{1}H-COSY NMR, 400 MHz, $CDCl_3$

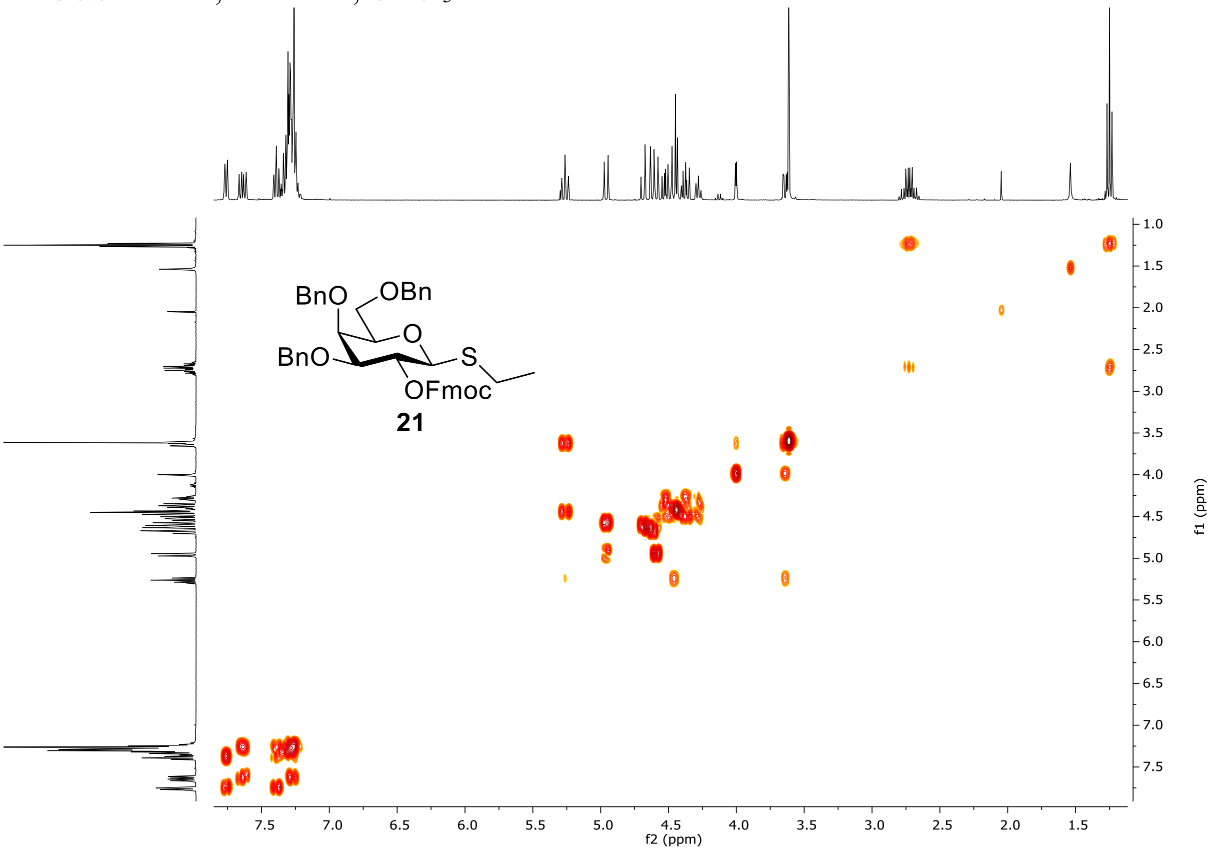

^{1}H-^{13}C-HSQC NMR, 400 MHz, $CDCl_3$

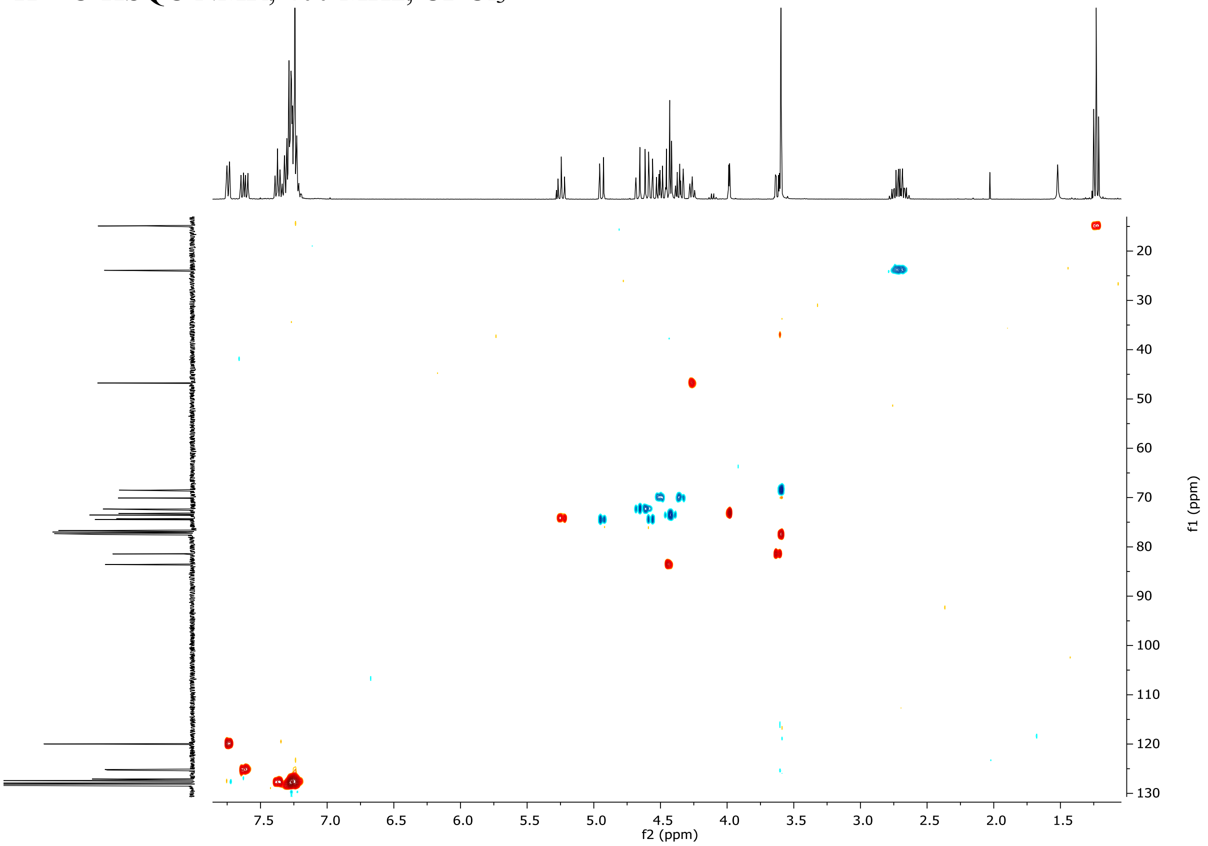

Supplementary Figure 36 | 2D NMR spectra of 21

^{1}H NMR, 400 MHz, $CDCl_3$

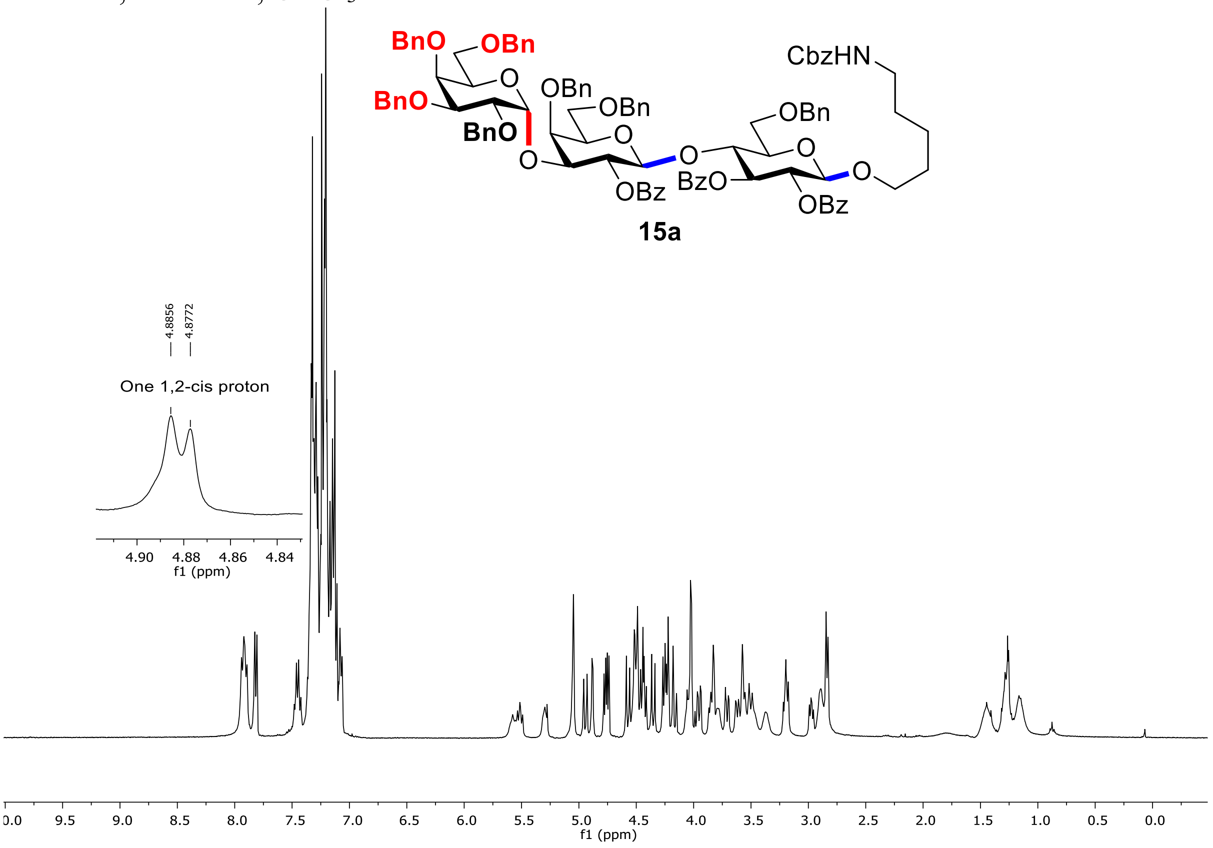

^{13}C NMR, 100 MHz, $CDCl_3$

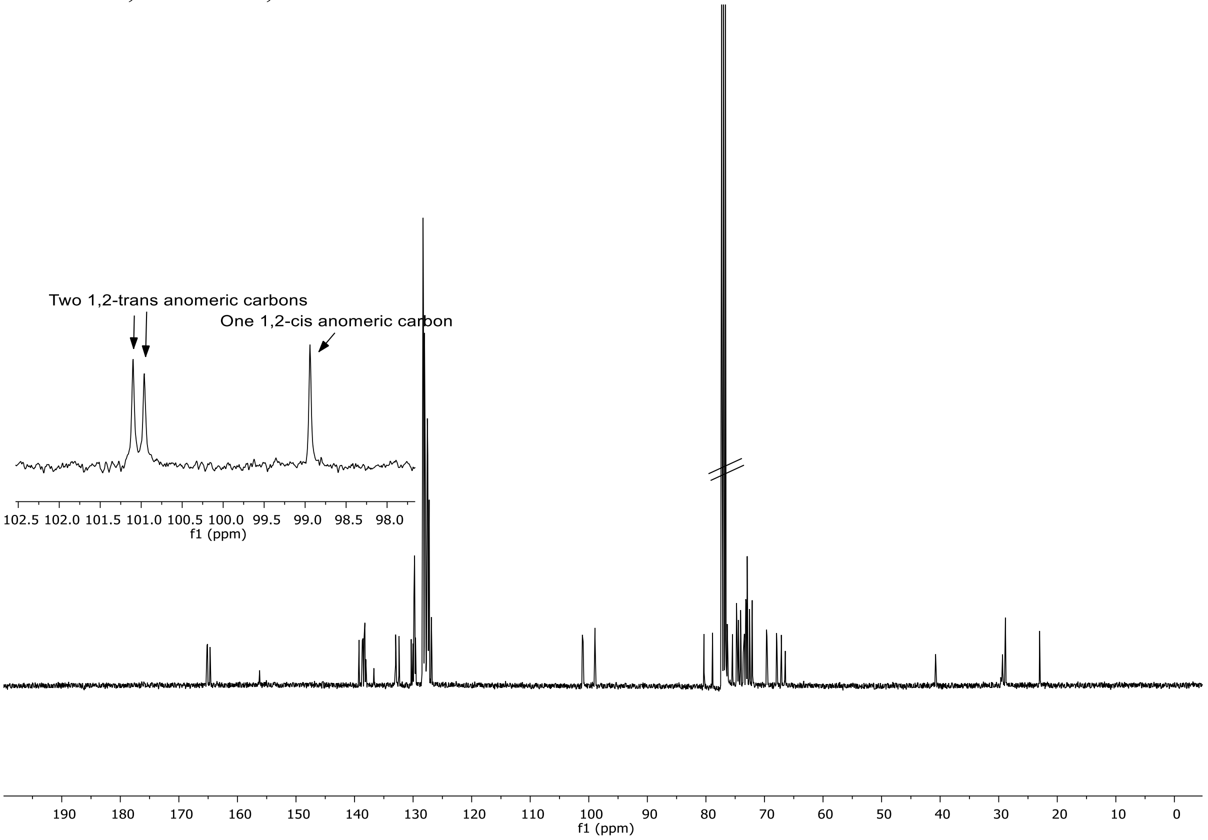

Supplementary Figure 37 | 1D NMR spectra of 15a

^{1}H-COSY NMR, 400 MHz, $CDCl_3$

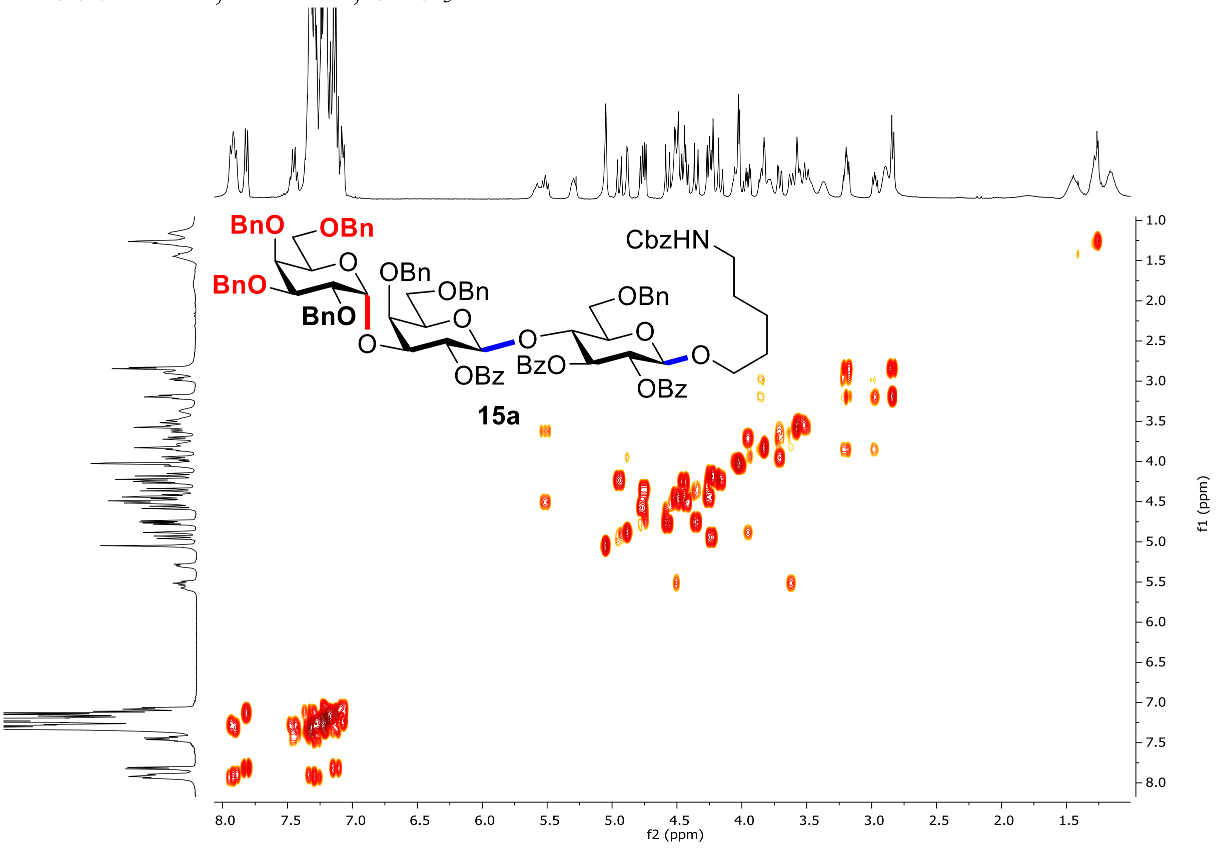

^{1}H-^{13}C-HSQC and ^{1}H-^{13}C-coupled-HSQC (zoom-in) NMR, 400 MHz, $CDCl_3$

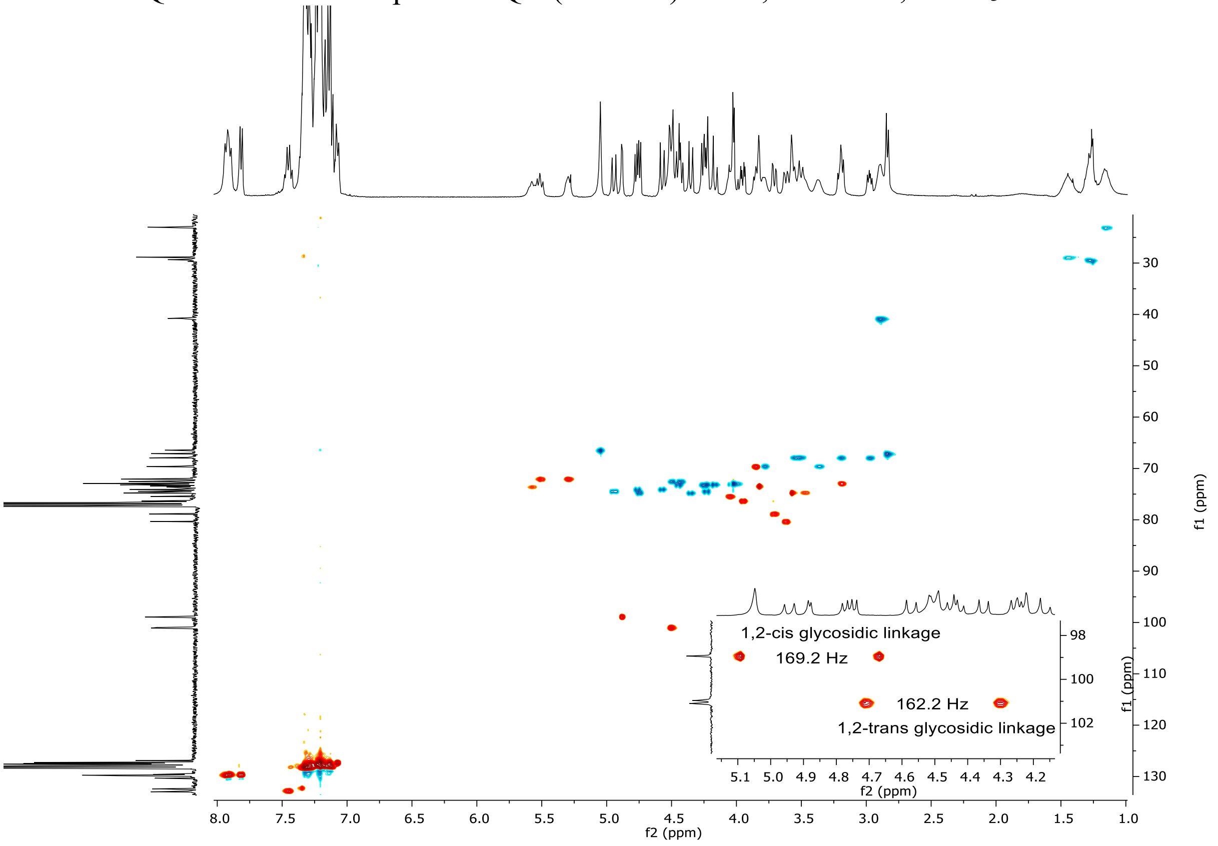

Supplementary Figure 38 | 2D NMR spectra of 15a

^{1}H NMR, 400 MHz, $CDCl_3$

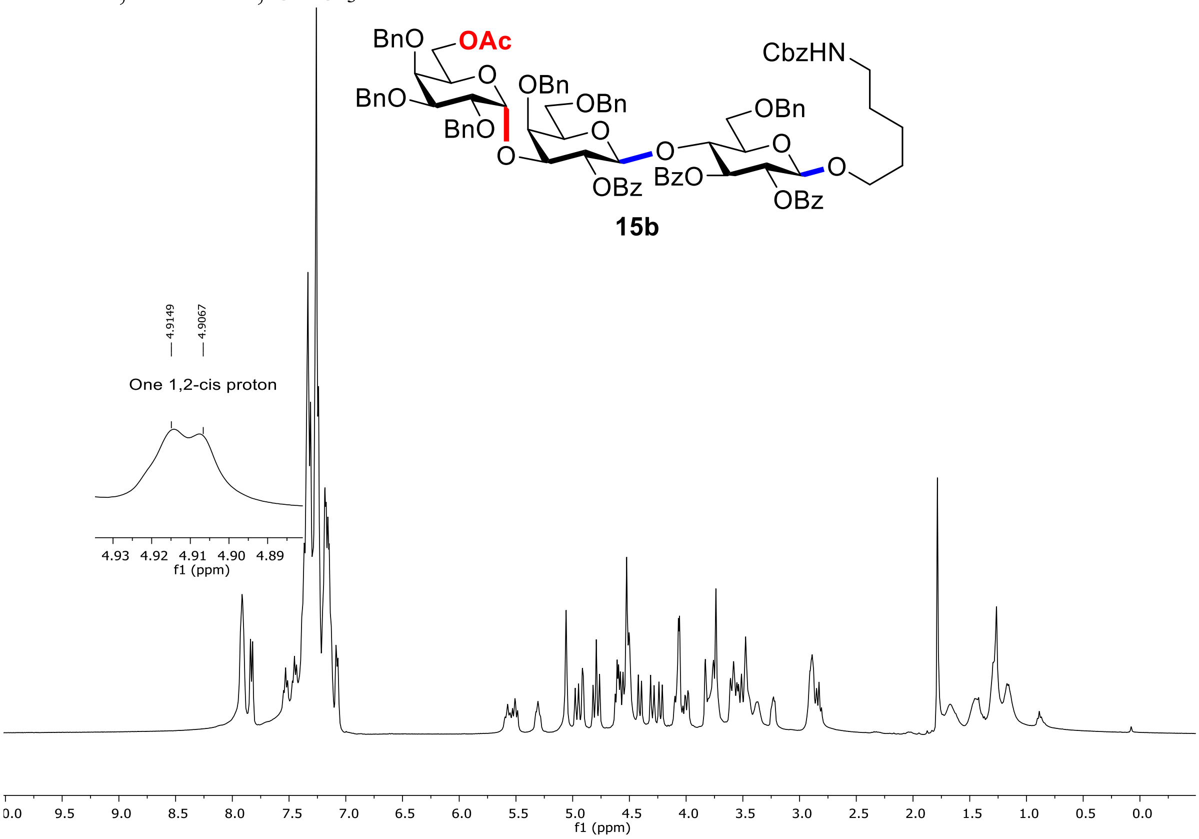

^{13}C NMR, 100 MHz, $CDCl_3$

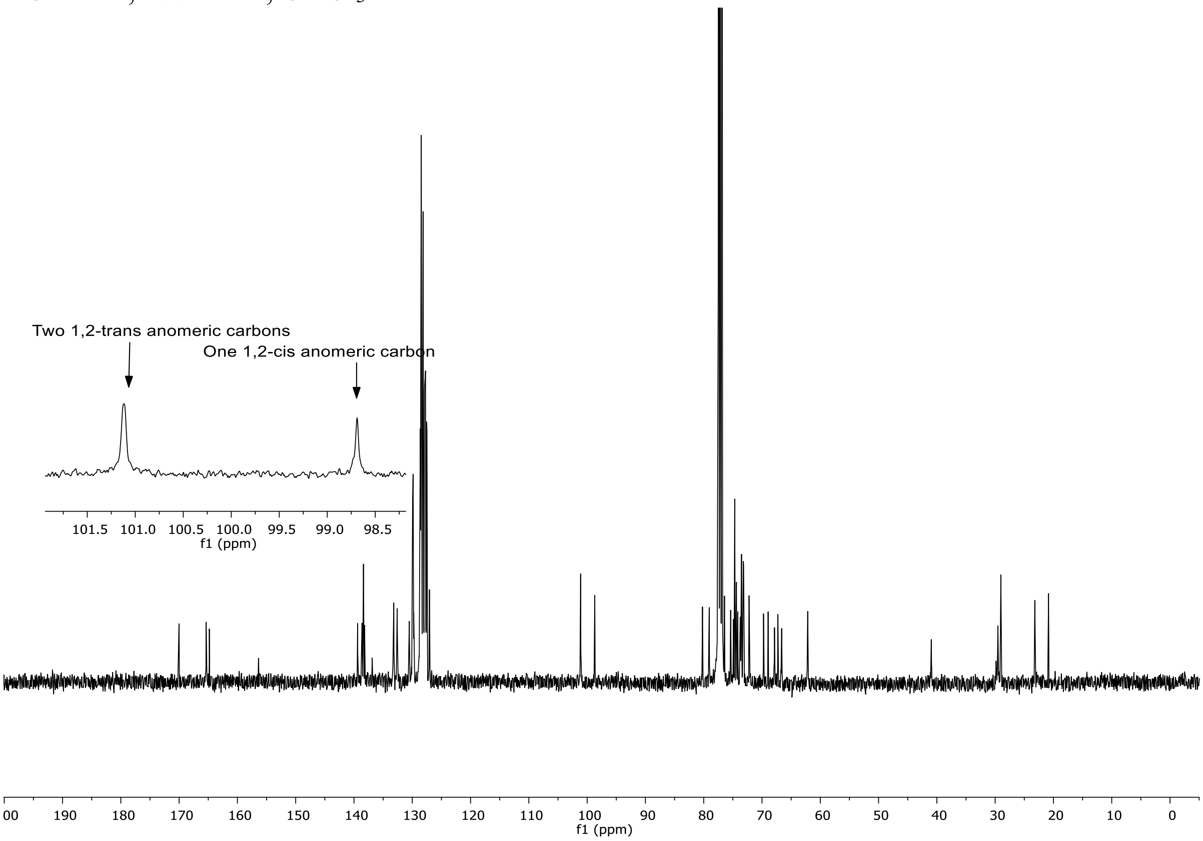

Supplementary Figure 39 | 1D NMR spectra of 15b

^{1}H-COSY NMR, 400 MHz, $CDCl_3$

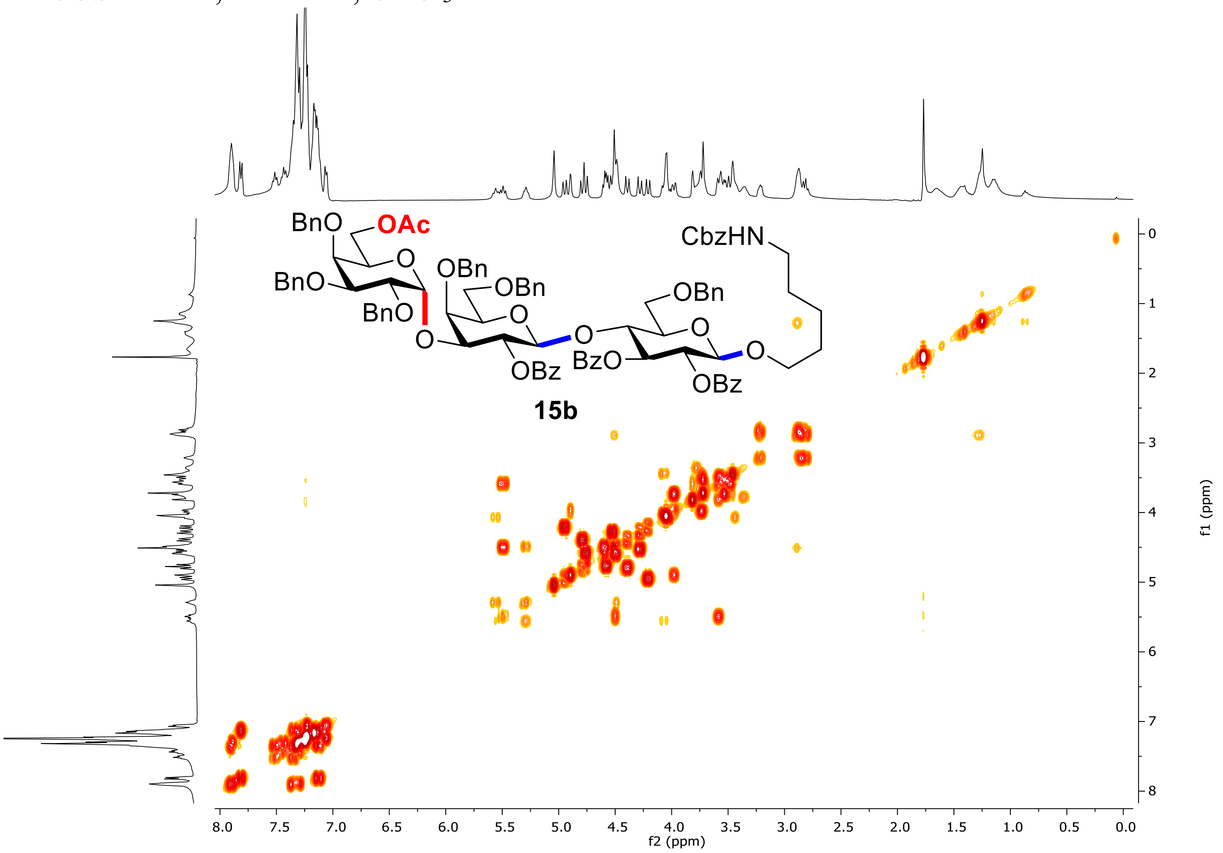

^{1}H-^{13}C-HSQC and ^{1}H-^{13}C-coupled-HSQC (zoom-in) NMR, 400 MHz, $CDCl_3$

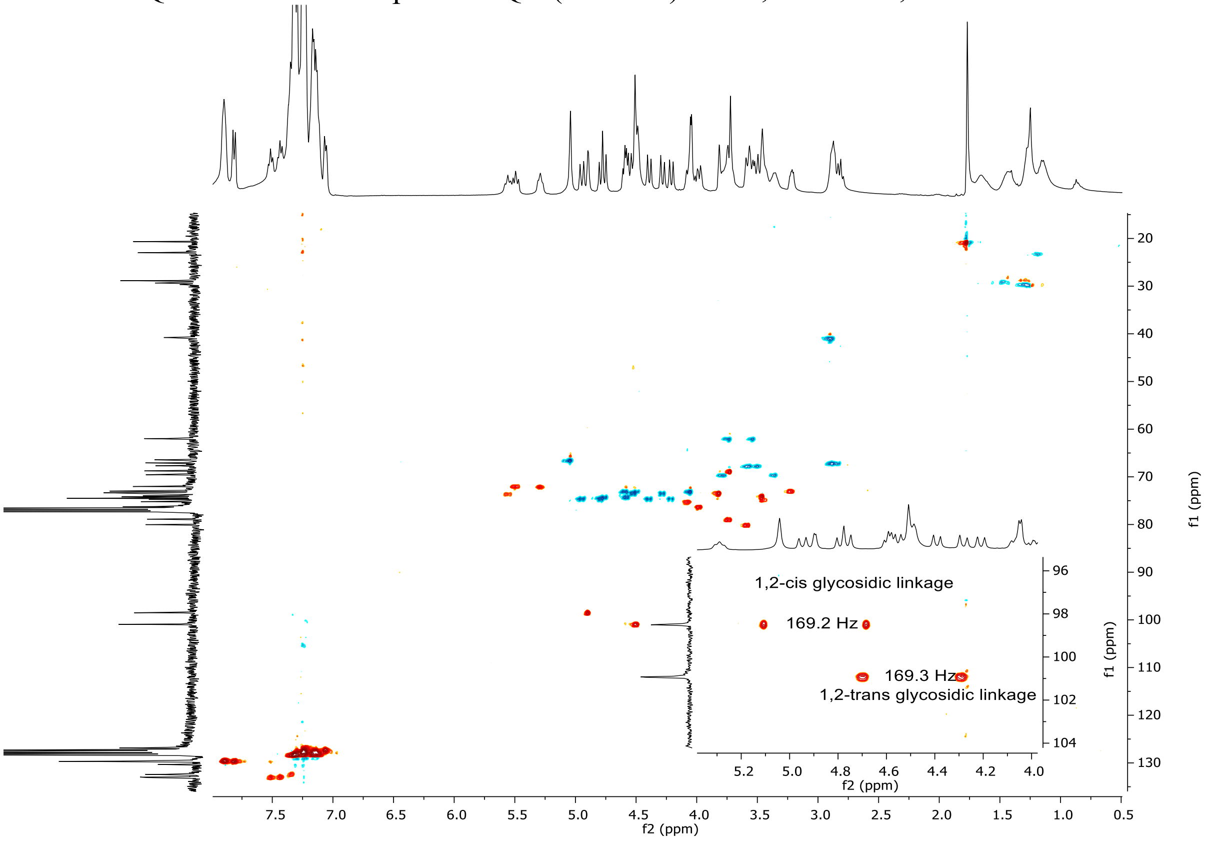

Supplementary Figure 40 | 2D NMR spectra of 15b

^{1}H NMR, 400 MHz, $CDCl_3$

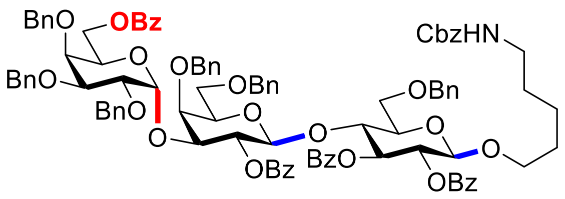

15c

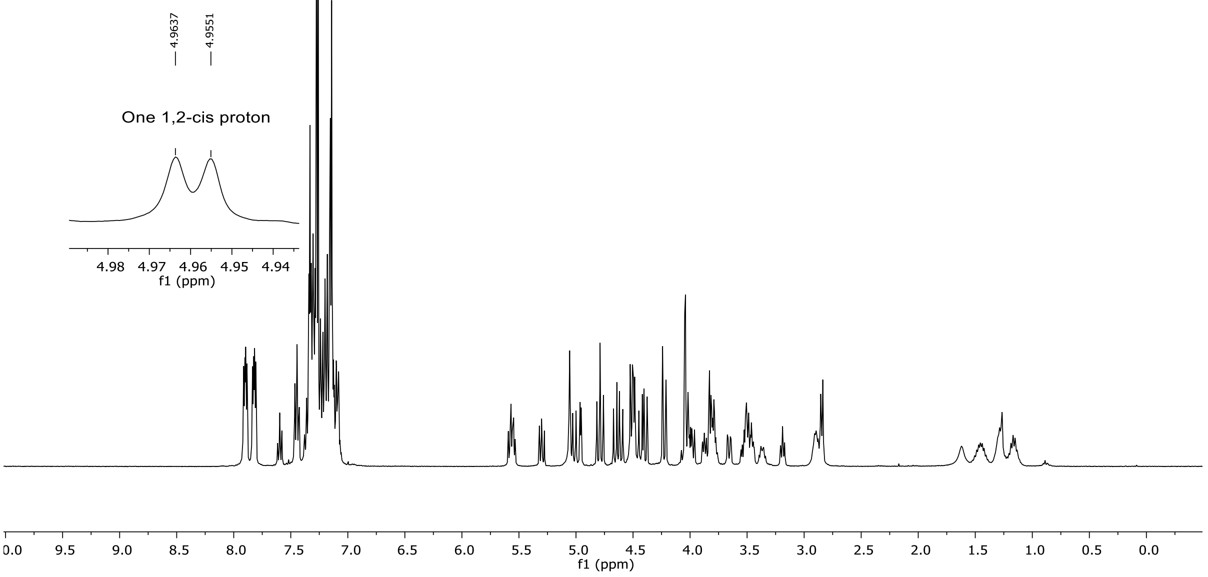

^{13}C NMR, 100 MHz, $CDCl_3$

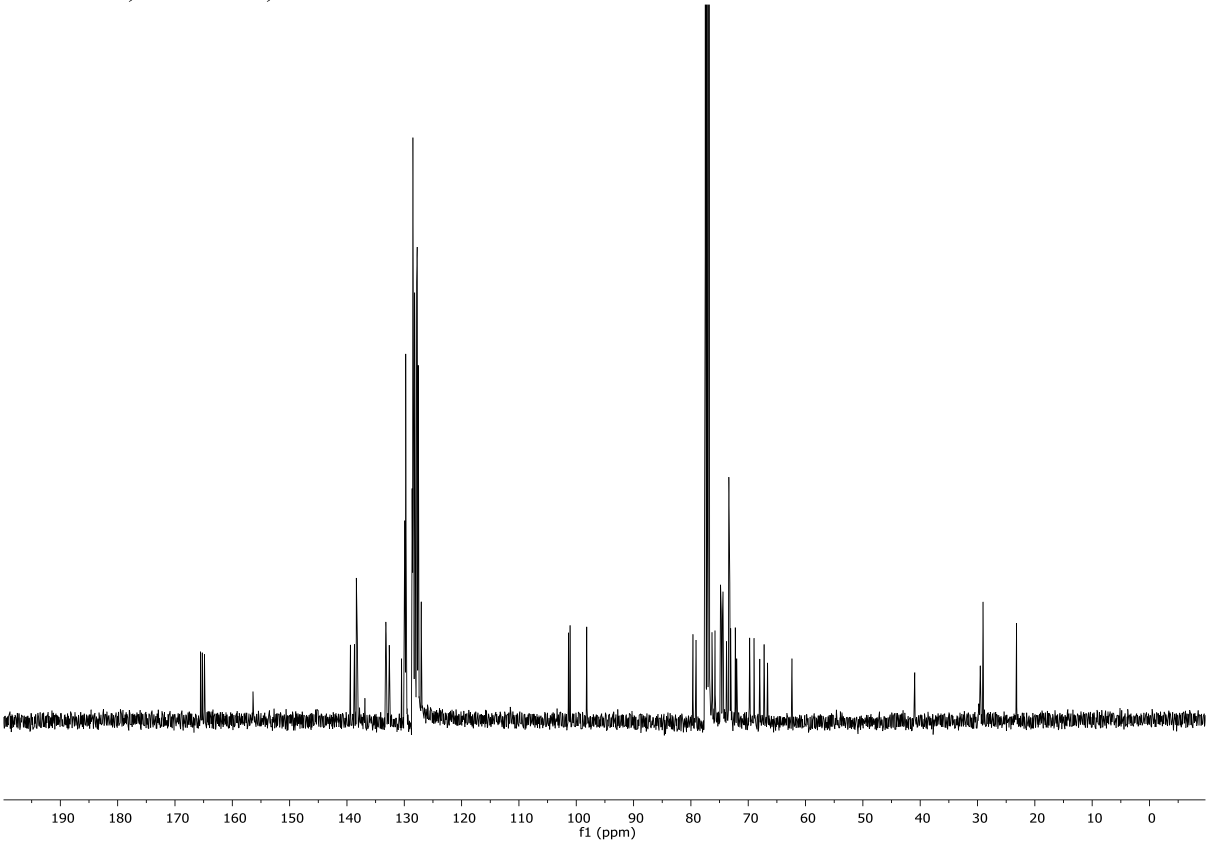

Supplementary Figure 41 | 1D NMR spectra of 15c

^{1}H-COSY NMR, 600 MHz, $CDCl_3$

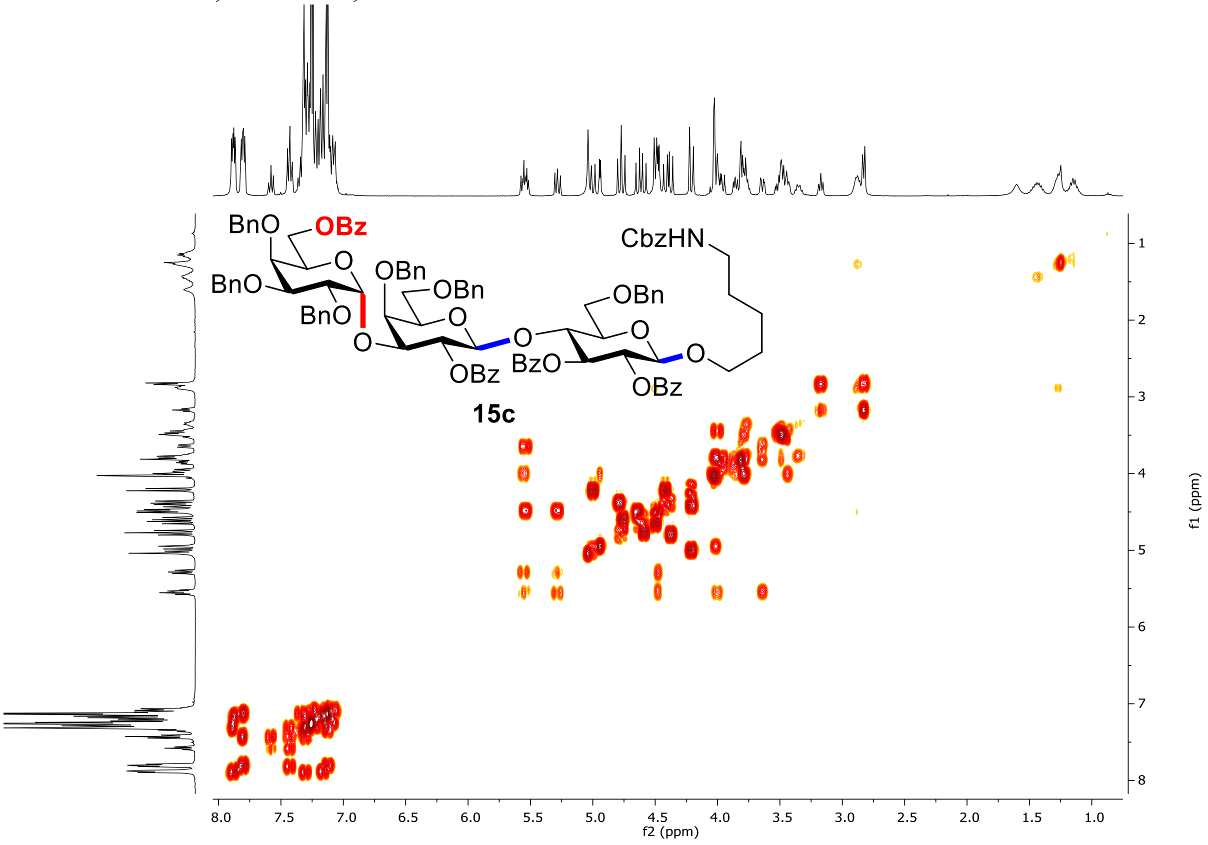

^{1}H-^{13}C-HSQC and ^{1}H-^{13}C-coupled-HSQC (zoom-in) NMR, 600 MHz, $CDCl_3$

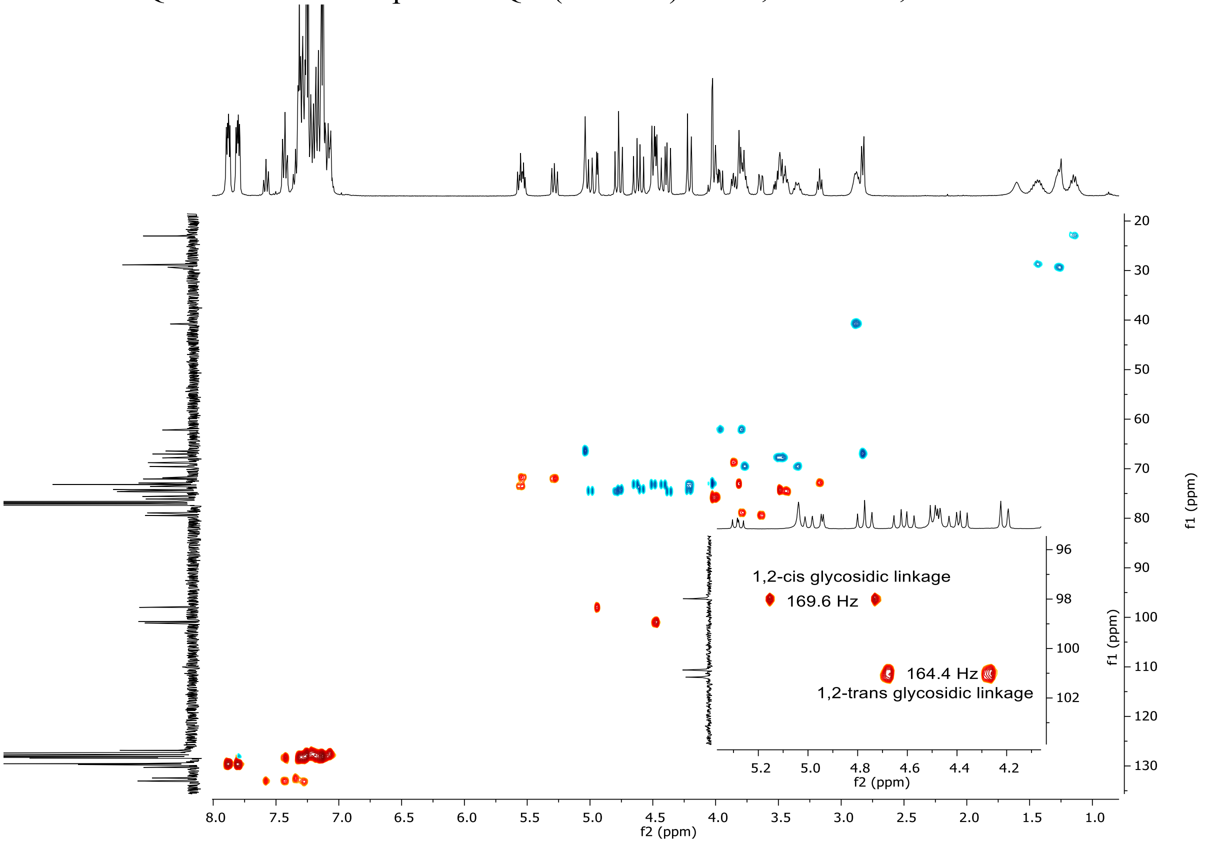

Supplementary Figure 42 | 2D NMR spectra of 15c

^{1}H NMR, 400 MHz, $CDCl_3$

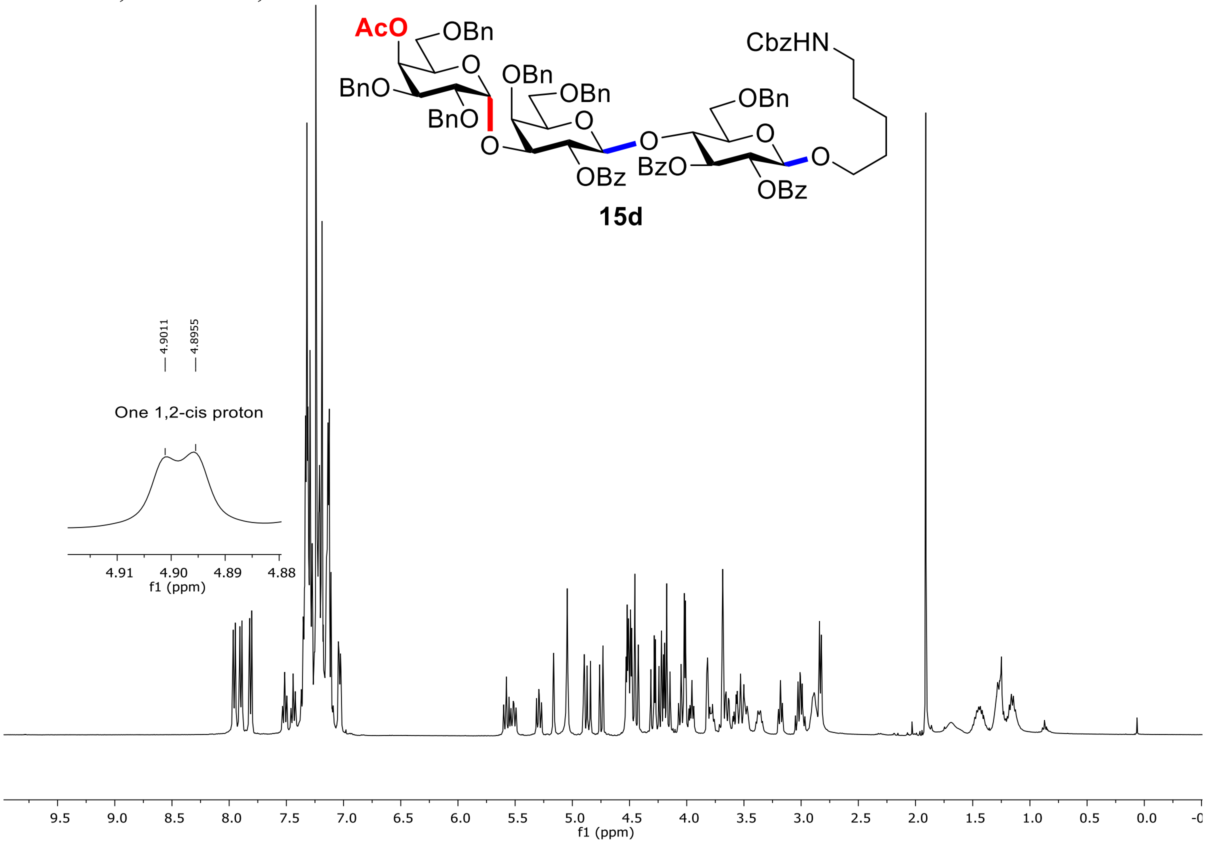

^{13}C NMR, 100 MHz, $CDCl_3$

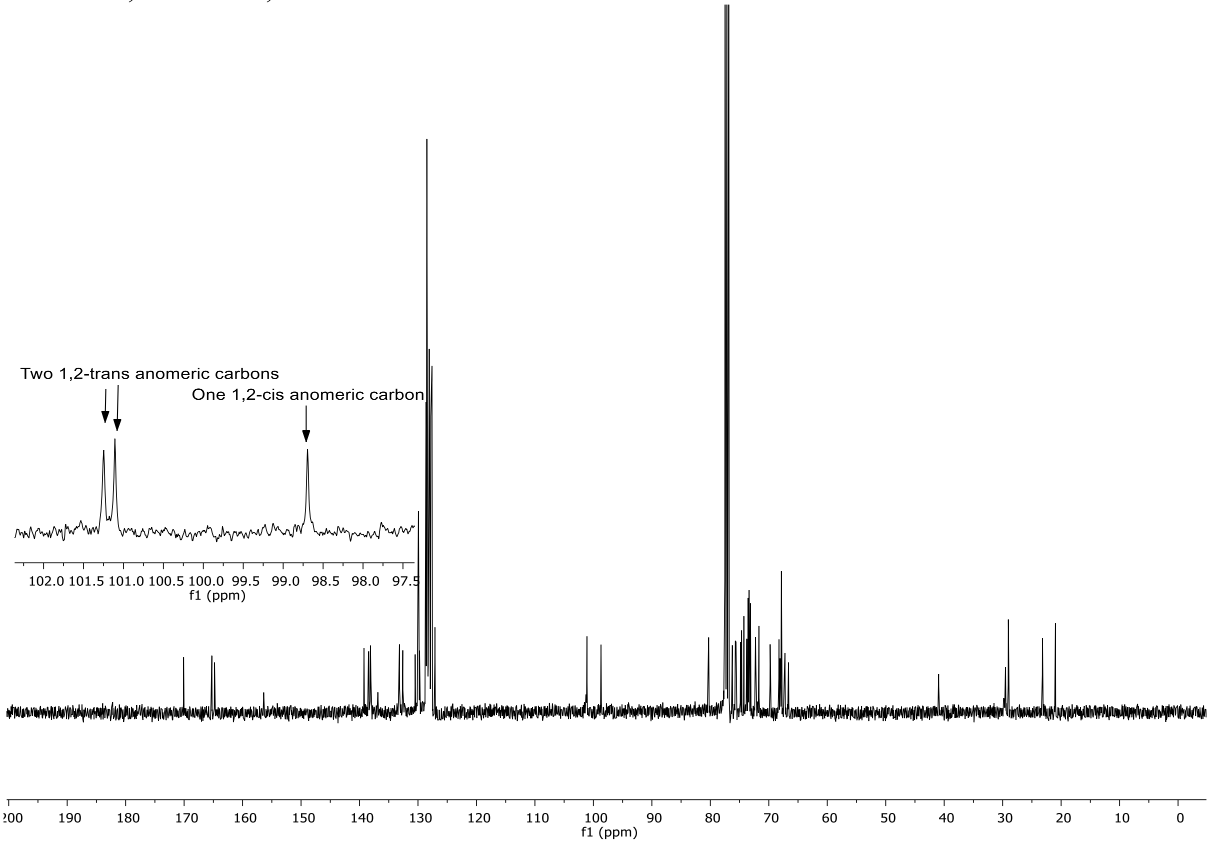

Supplementary Figure 43 | 1D NMR spectra of 15d

^{1}H-COSY NMR, 400 MHz, $CDCl_3$

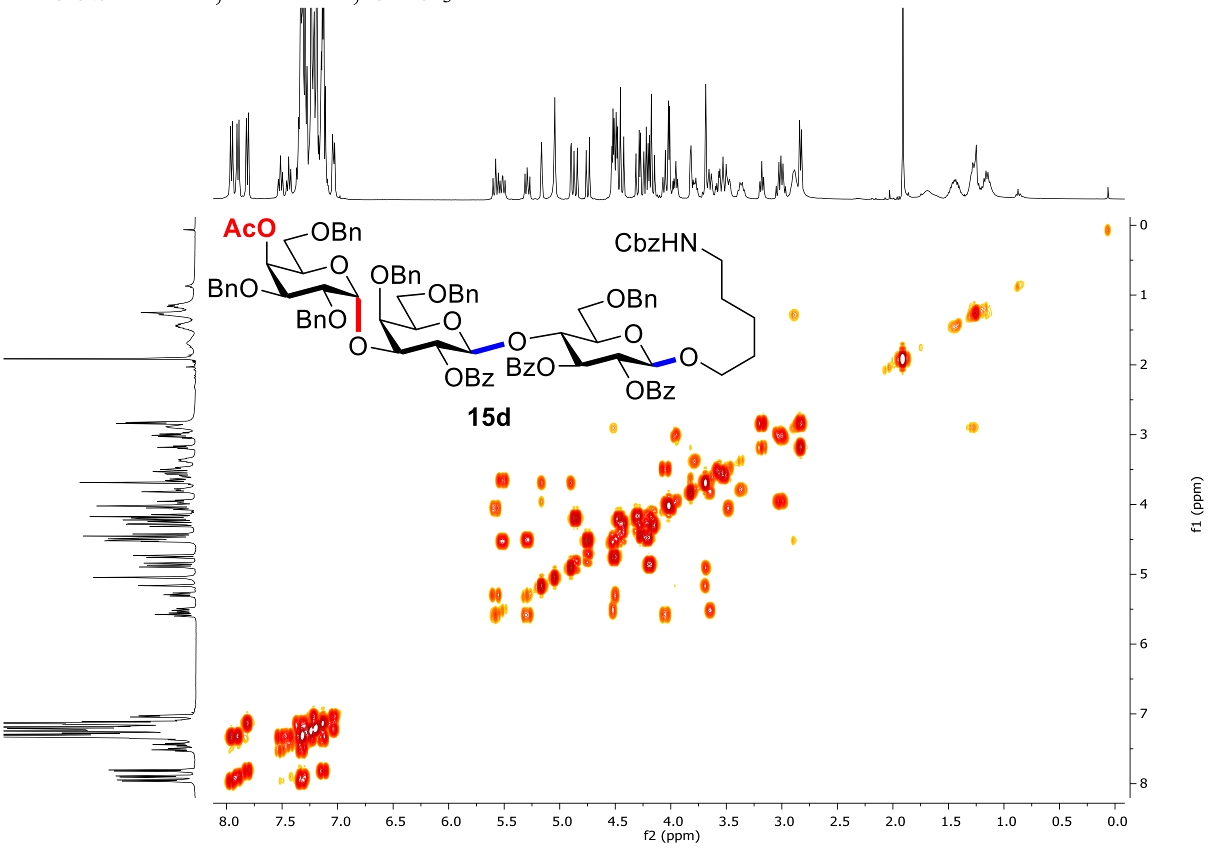

^{1}H-^{13}C-HSQC and ^{1}H-^{13}C-coupled-HSQC (zoom-in) NMR, 400 MHz, $CDCl_3$

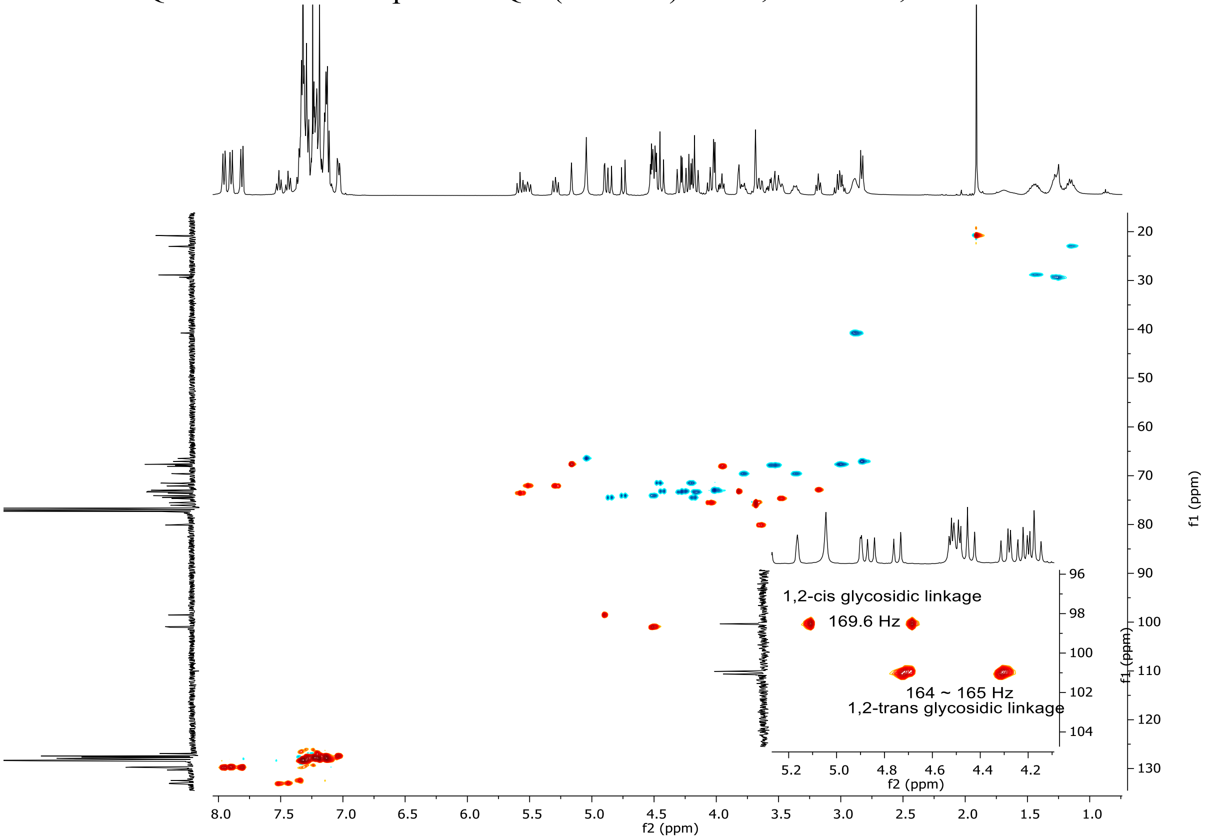

Supplementary Figure 44 | 2D NMR spectra of 15d

^{1}H NMR, 400 MHz, $CDCl_3$

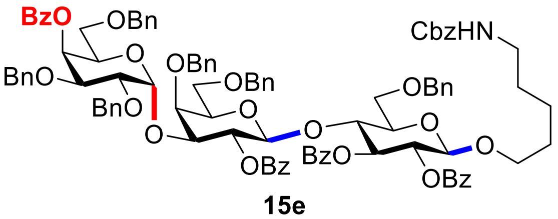

15e

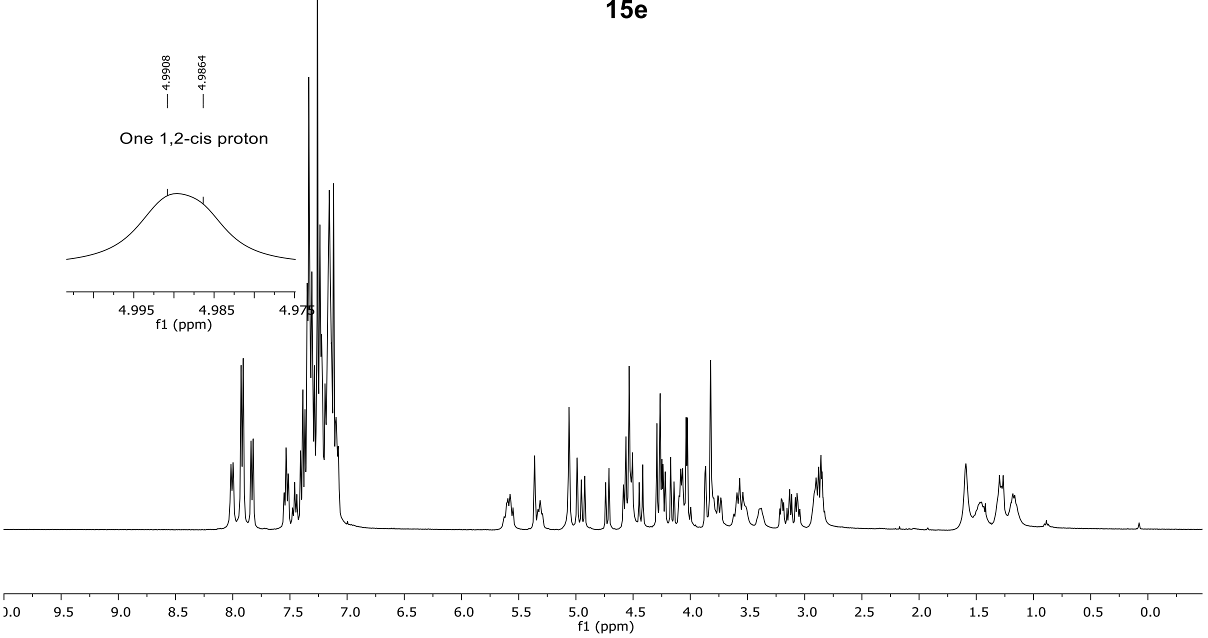

^{13}C NMR, 100 MHz, $CDCl_3$

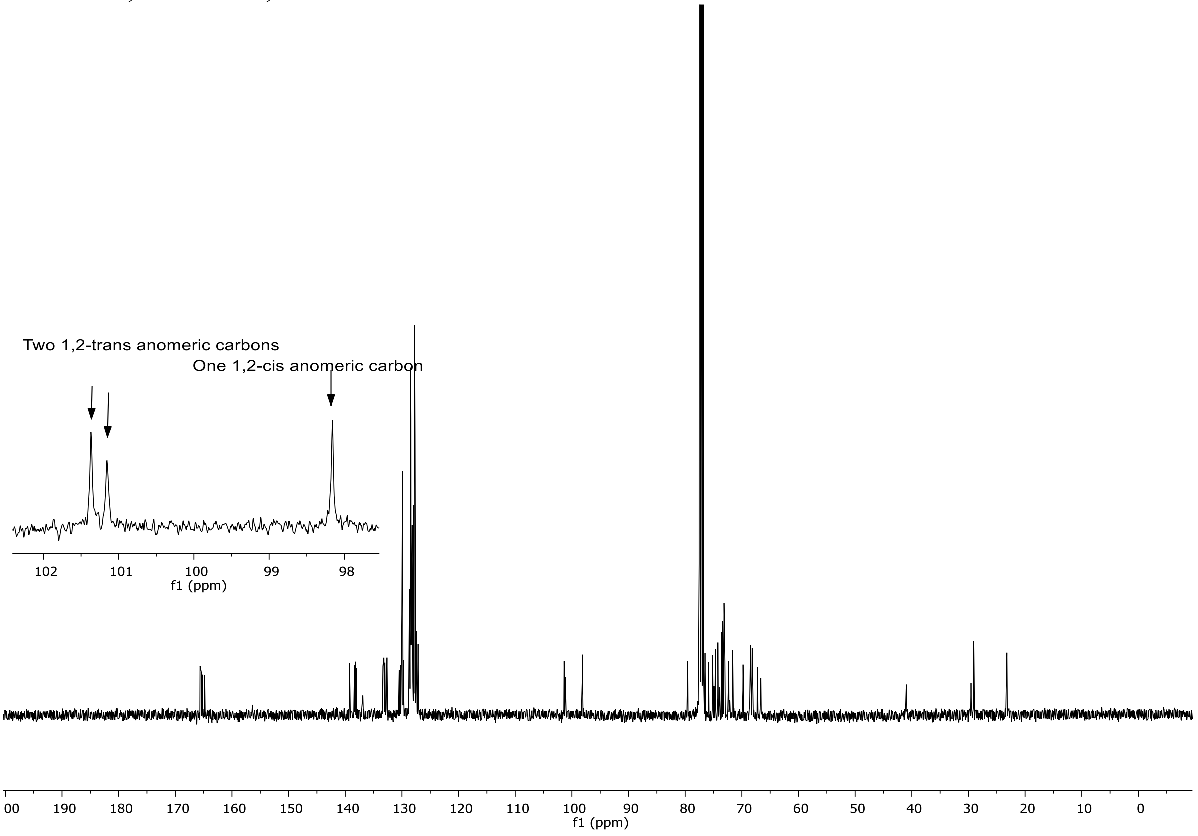

Supplementary Figure 45 | 1D NMR spectra of 15e

^{1}H-COSY NMR, 400 MHz, $CDCl_3$

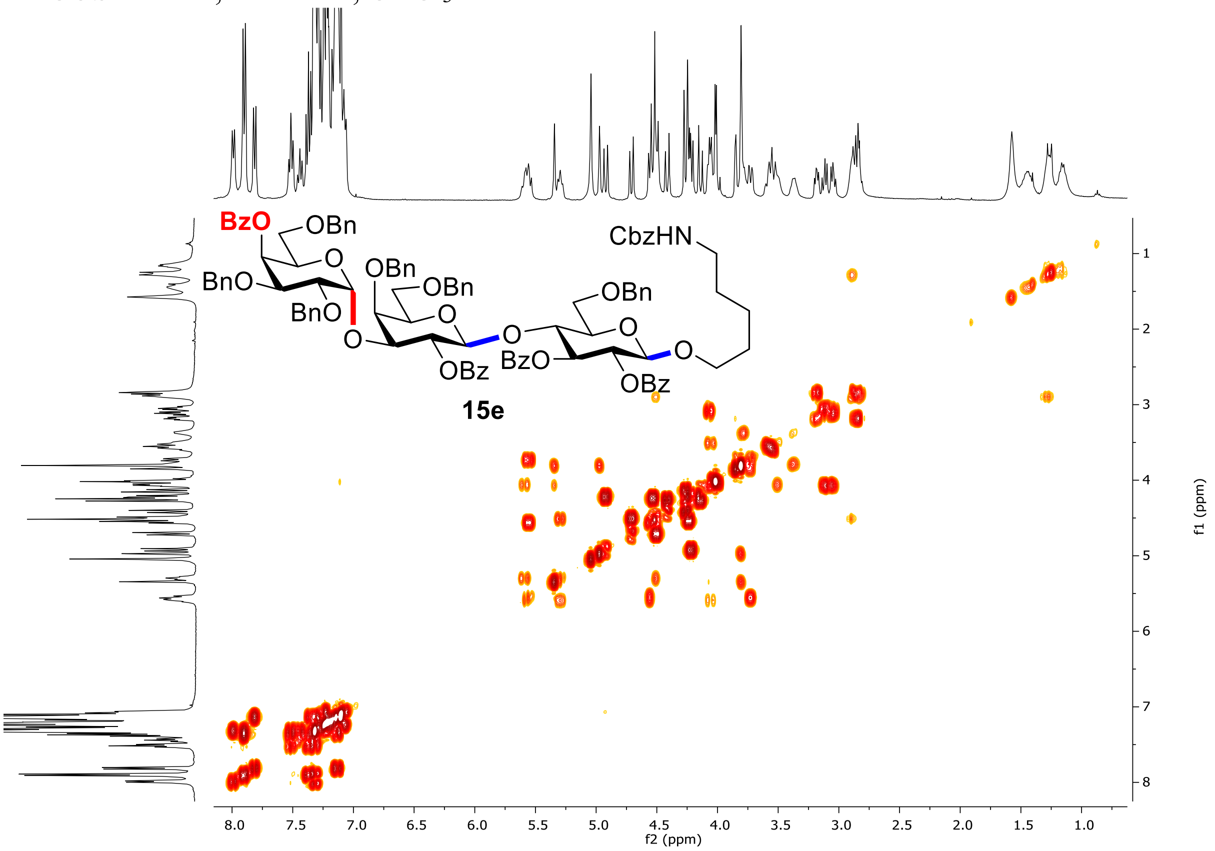

^{1}H-^{13}C-HSQC and ^{1}H-^{13}C-coupled-HSQC (zoom-in) NMR, 400 MHz, $CDCl_3$

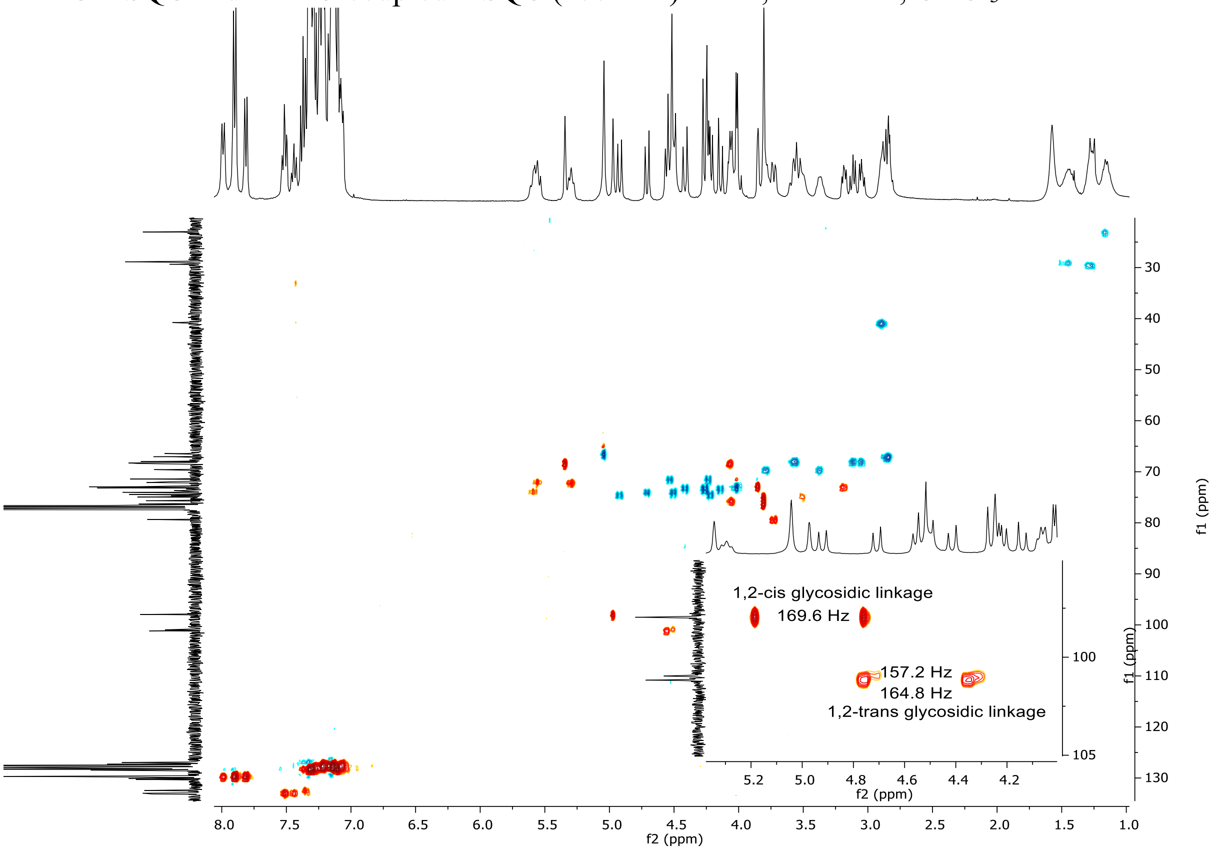

Supplementary Figure 46 | 2D NMR spectra of 15e

^{1}H NMR, 400 MHz, $CDCl_3$

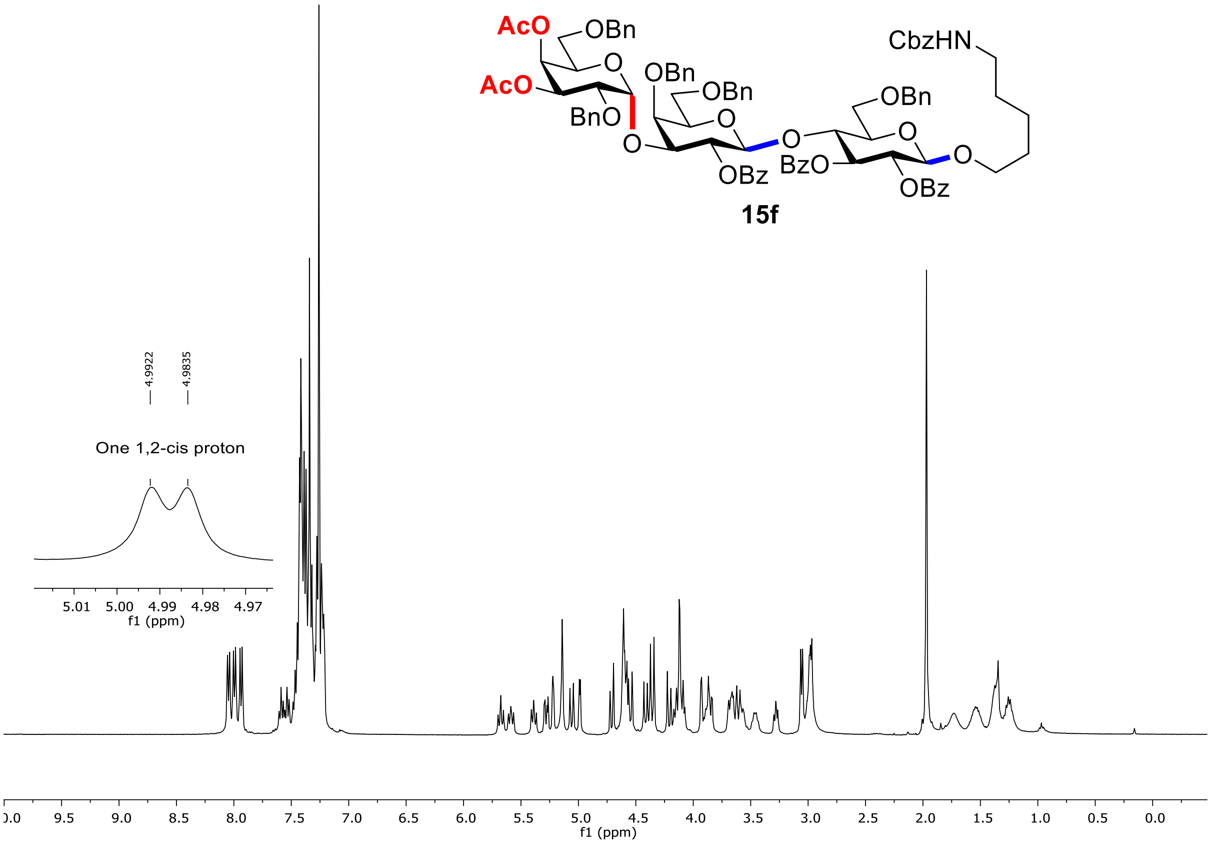

^{13}C NMR, 100 MHz, $CDCl_3$

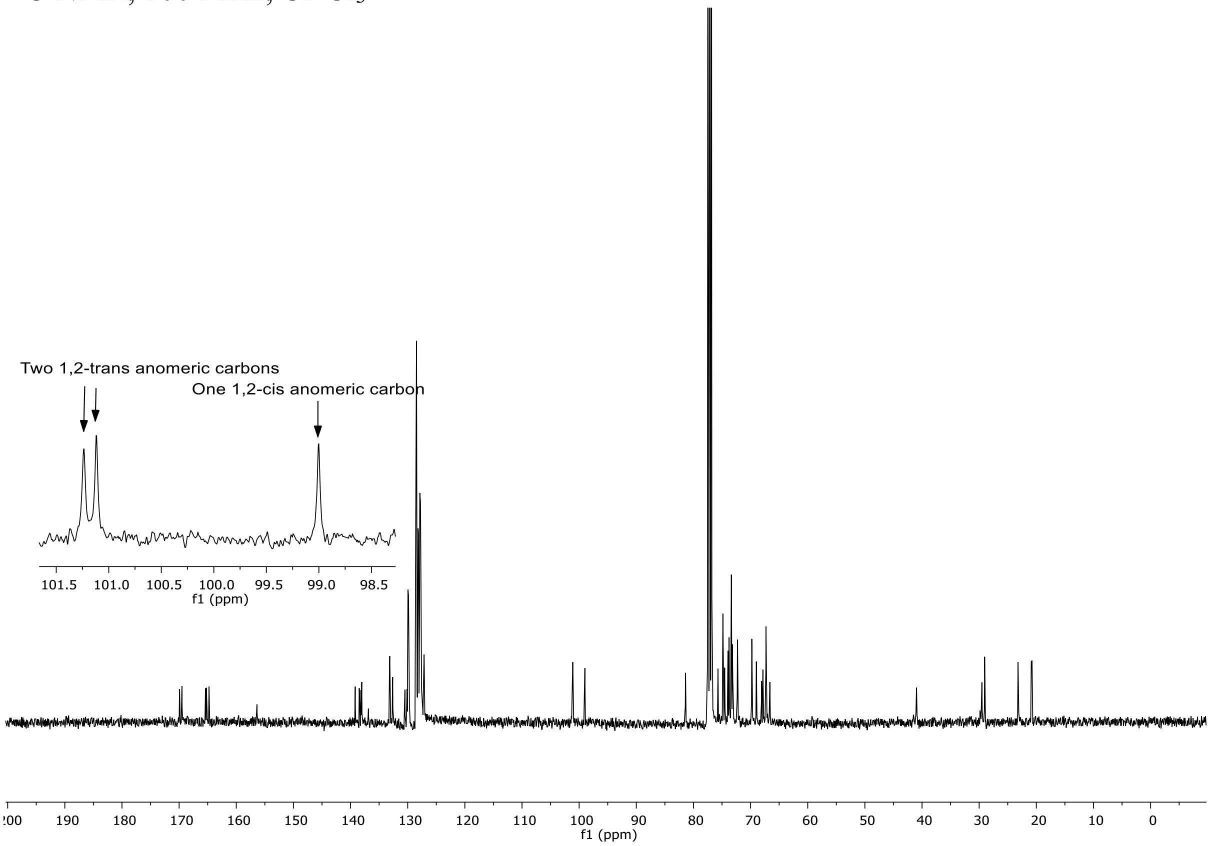

Supplementary Figure 47 | 1D NMR spectra of 15f

^{1}H-COSY NMR, 400 MHz, $CDCl_3$

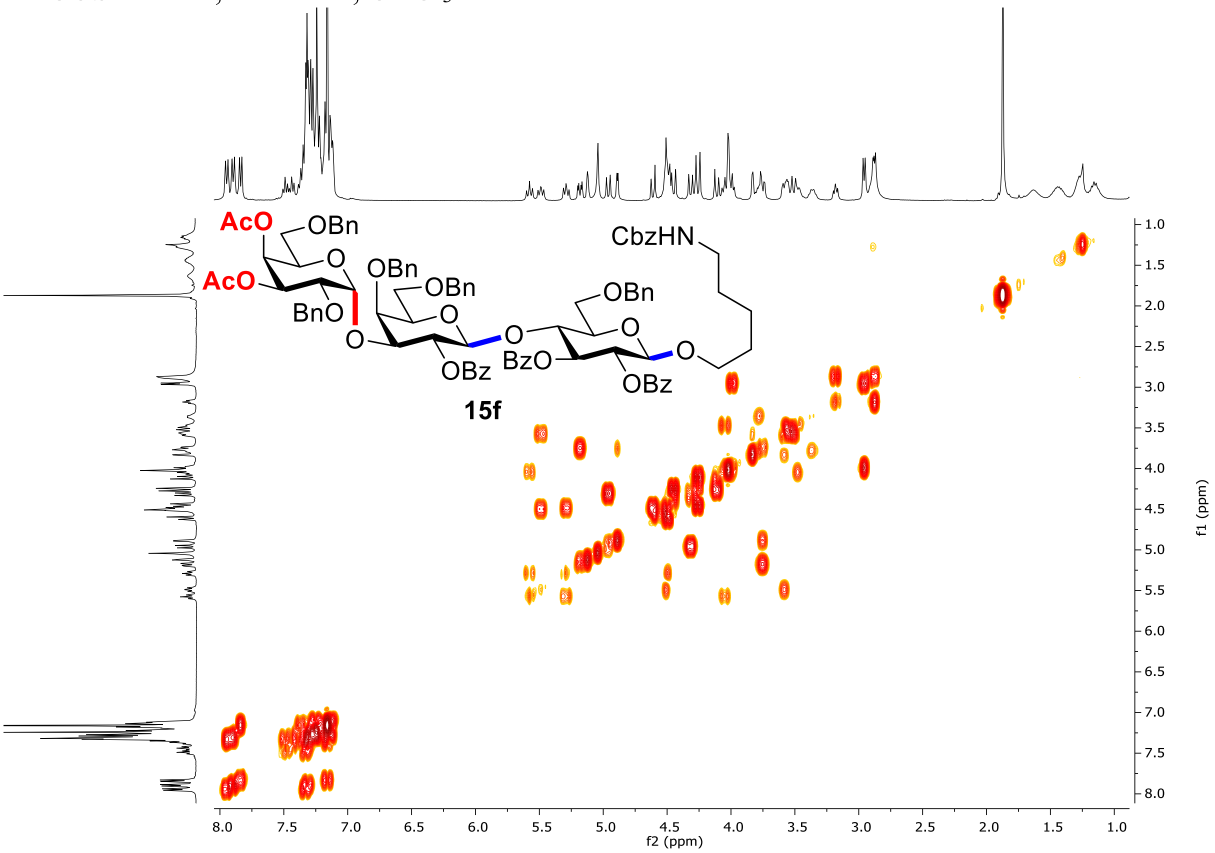

^{1}H-^{13}C-HSQC and ^{1}H-^{13}C-coupled-HSQC (zoom-in) NMR, 400 MHz, $CDCl_3$

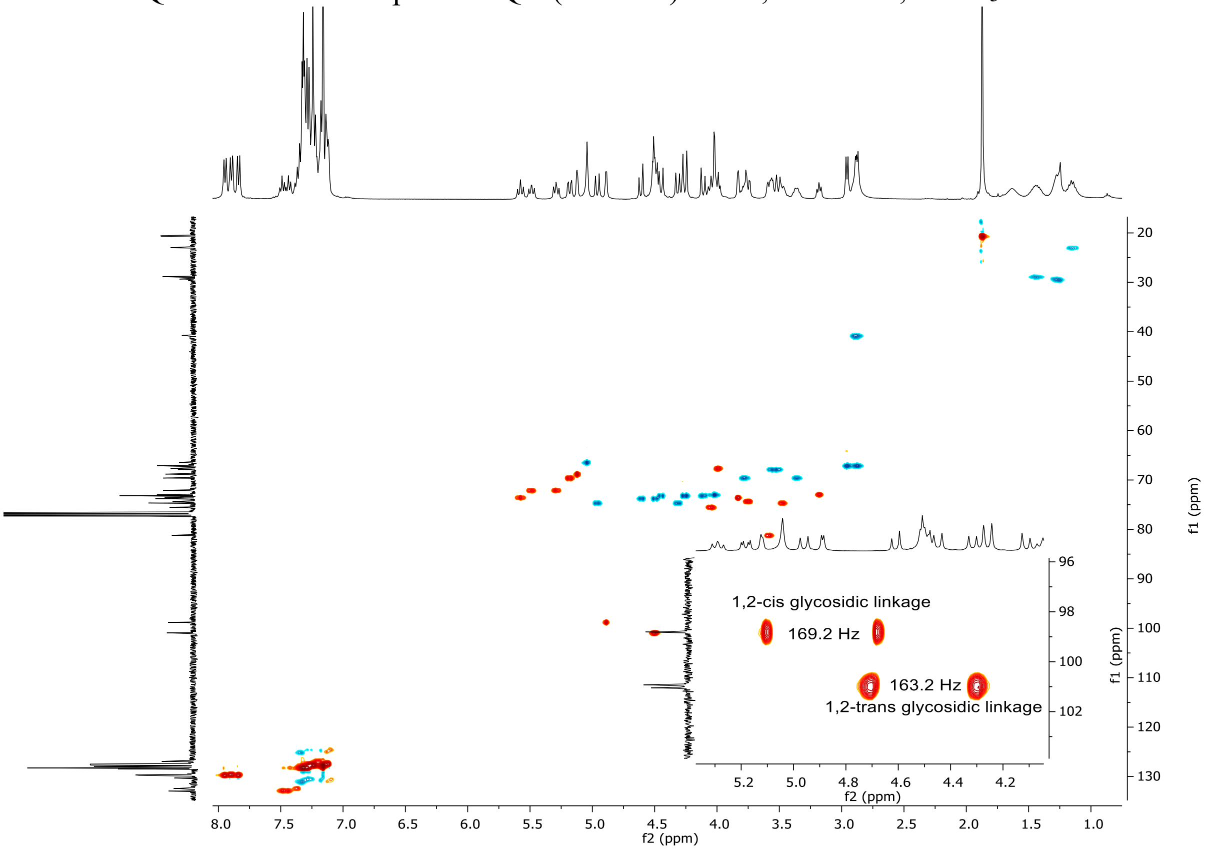

Supplementary Figure 48 | 2D NMR spectra of 15f

^{1}H NMR, 600 MHz, $CDCl_3$

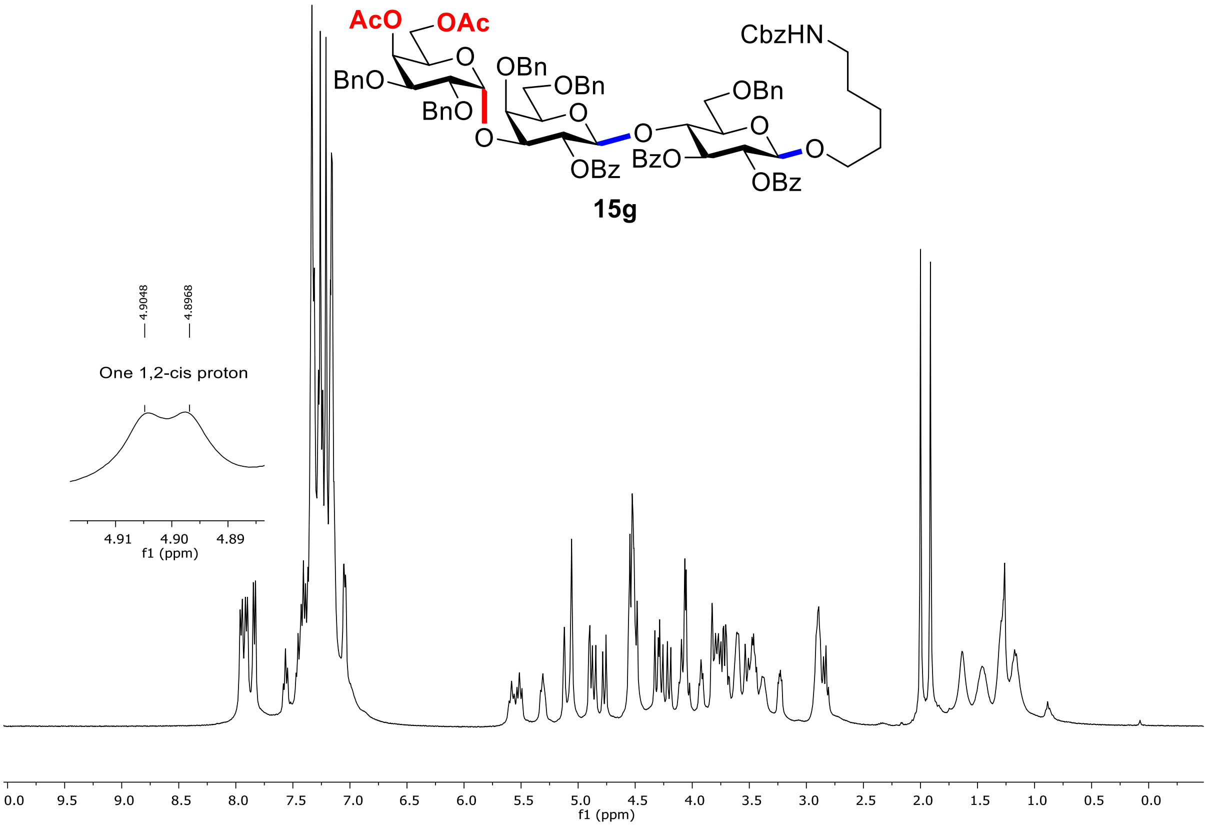

^{13}C NMR, 150 MHz, $CDCl_3$

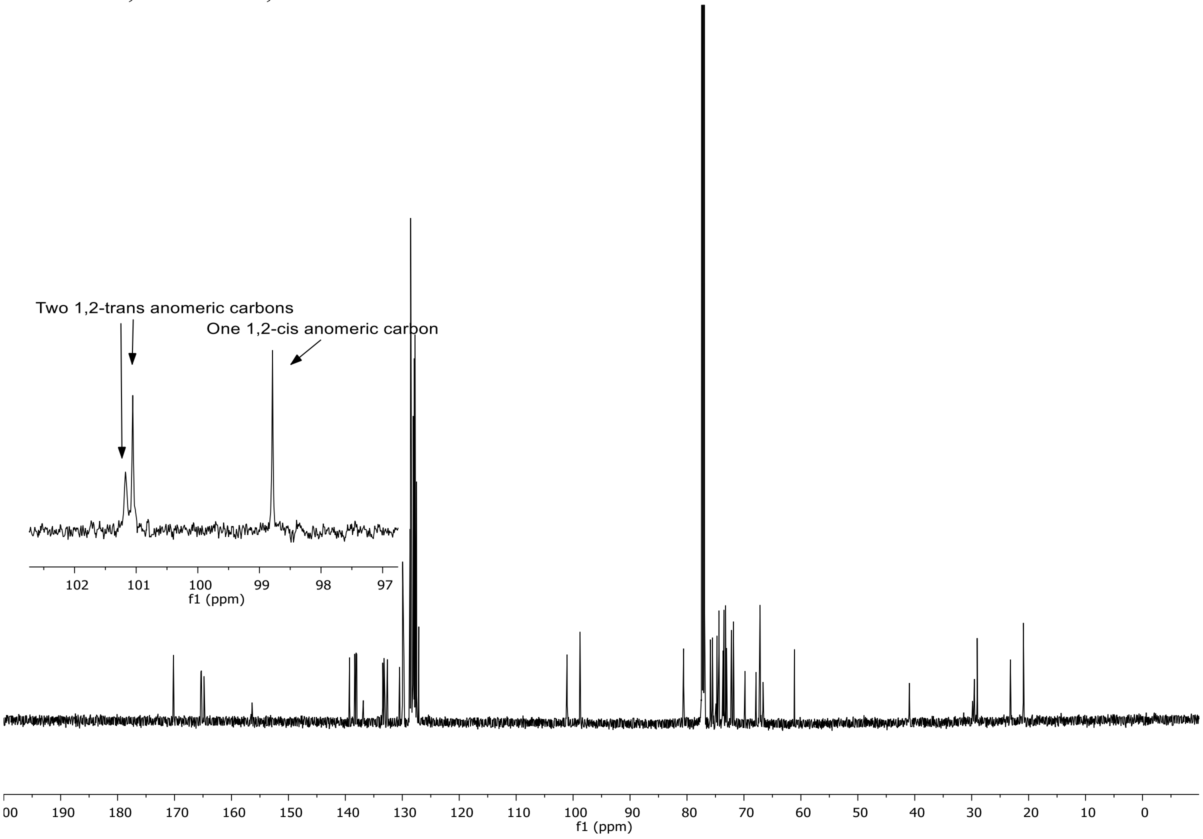

Supplementary Figure 49 | 1D NMR spectra of 15g

^{1}H-COSY NMR, 600 MHz, $CDCl_3$

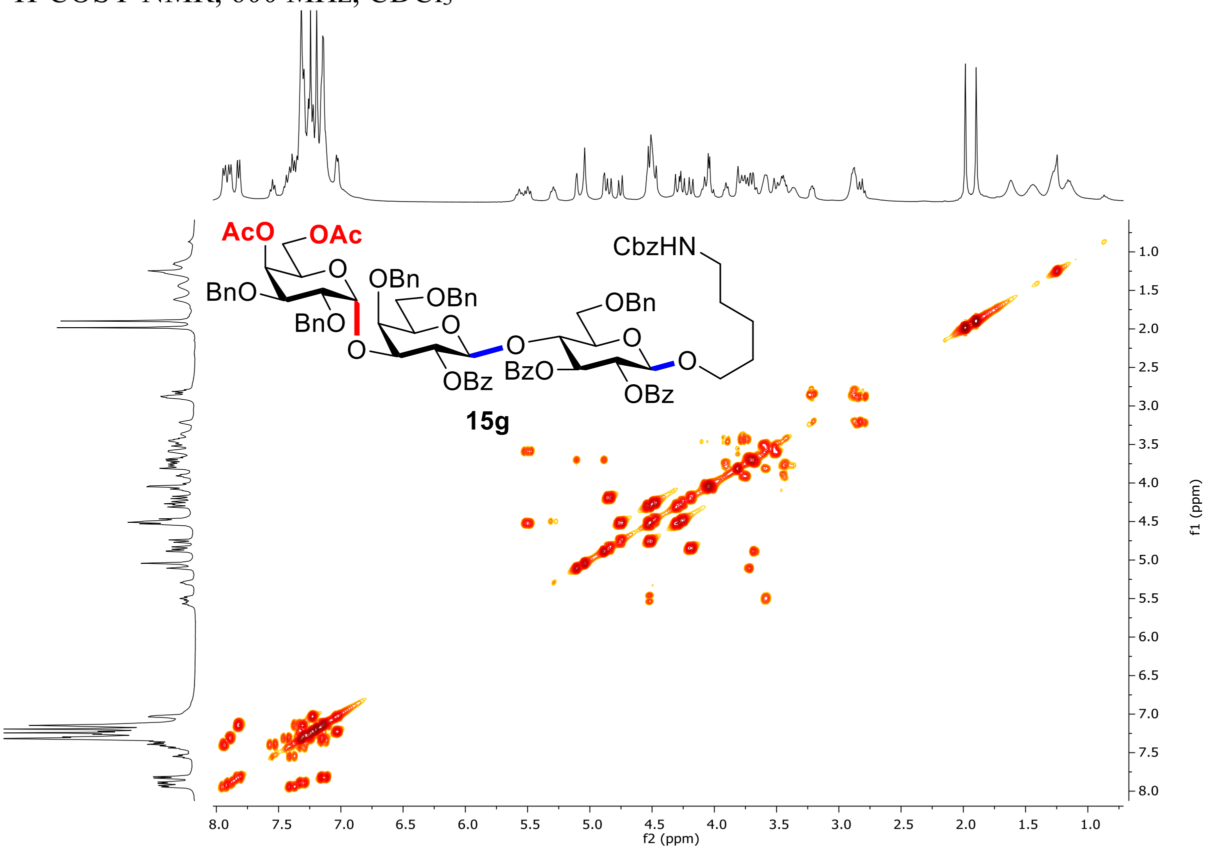

^{1}H-^{13}C-HSQC and ^{1}H-^{13}C-coupled-HSQC (zoom-in) NMR, 600 MHz, $CDCl_3$

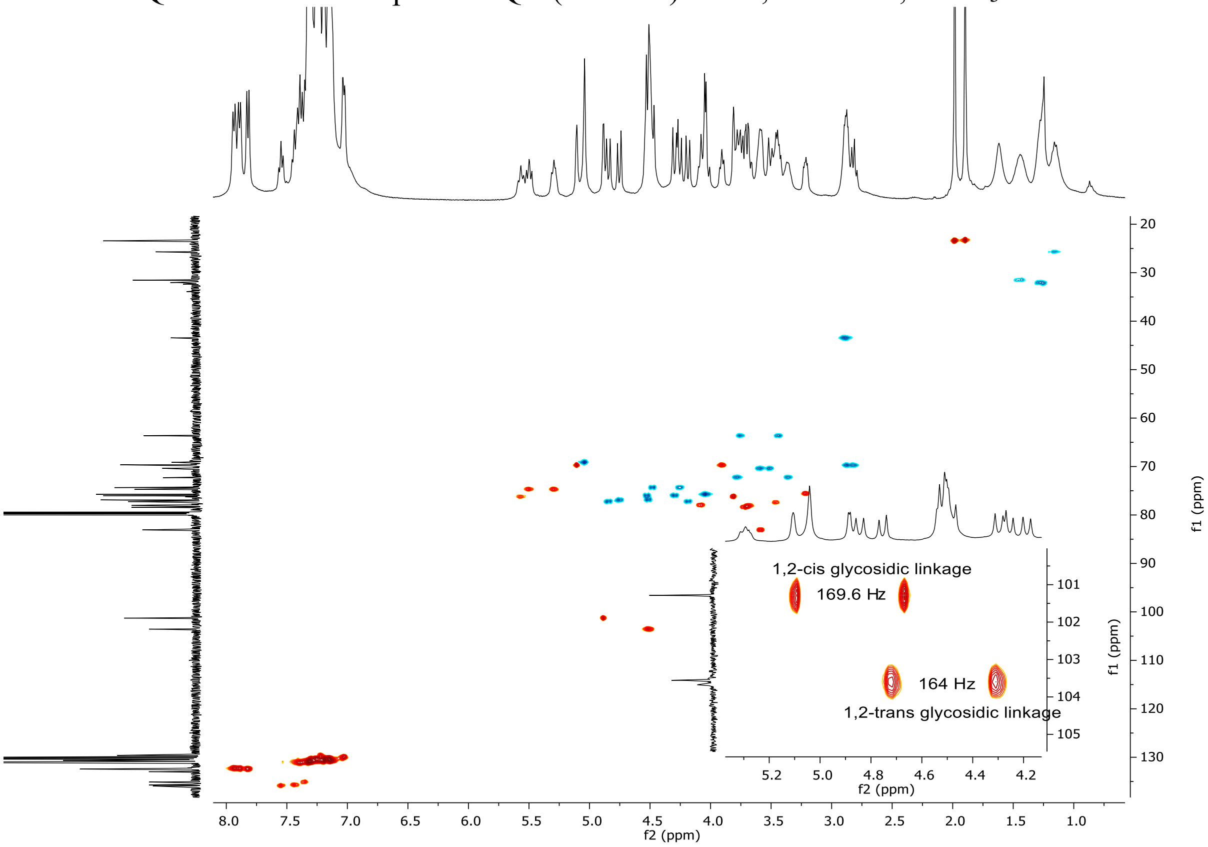

Supplementary Figure 50 | 2D NMR spectra of 15g

^{1}H NMR, 400 MHz, $CDCl_3$

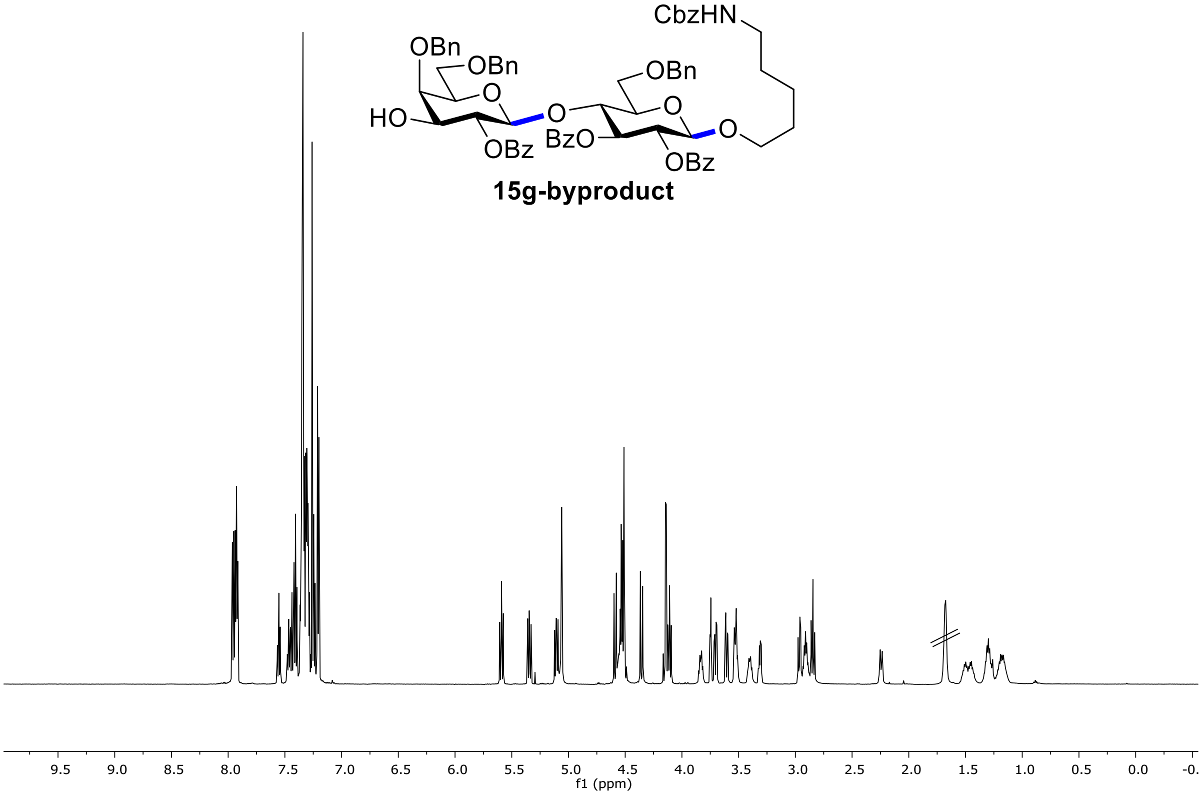

^{13}C NMR, 100 MHz, $CDCl_3$

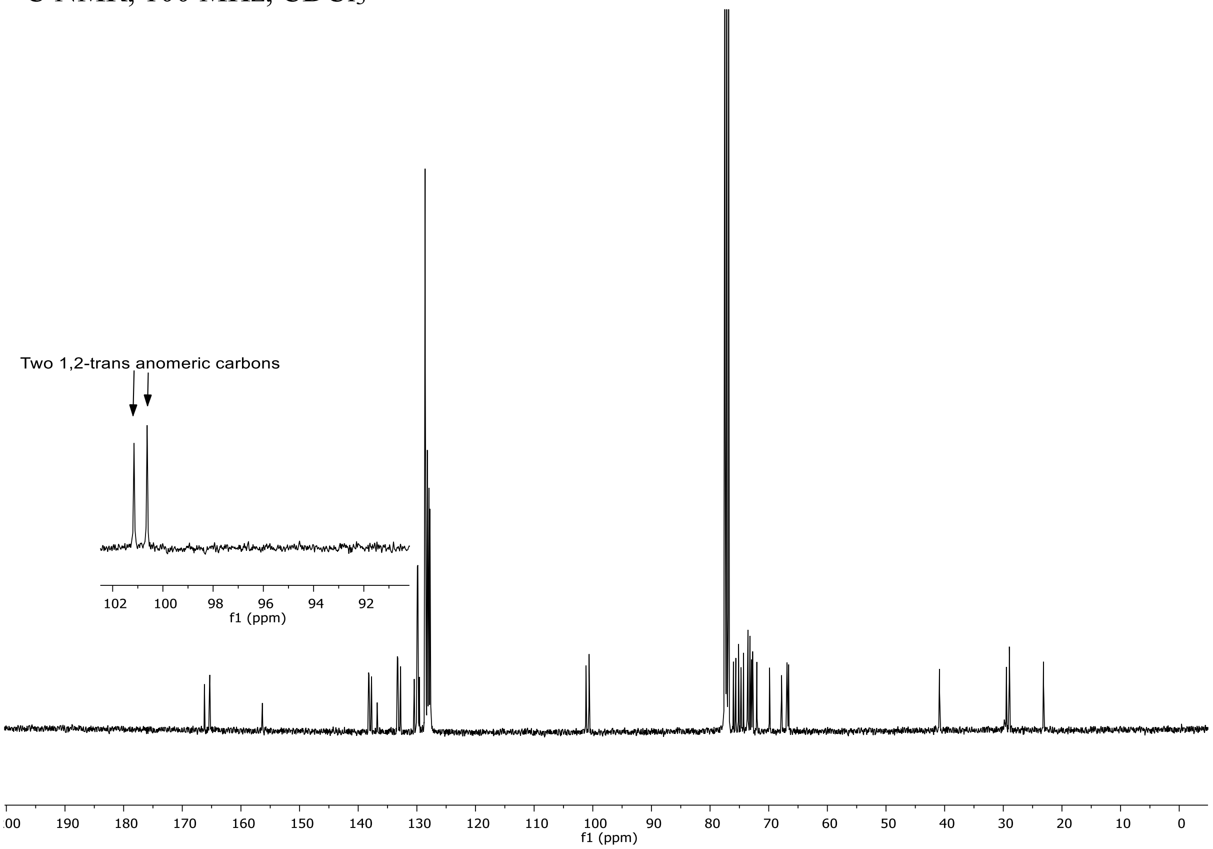

Supplementary Figure 51 | 1D NMR spectra of 15g-byproduct

^{1}H-COSY NMR, 100 MHz, $CDCl_3$

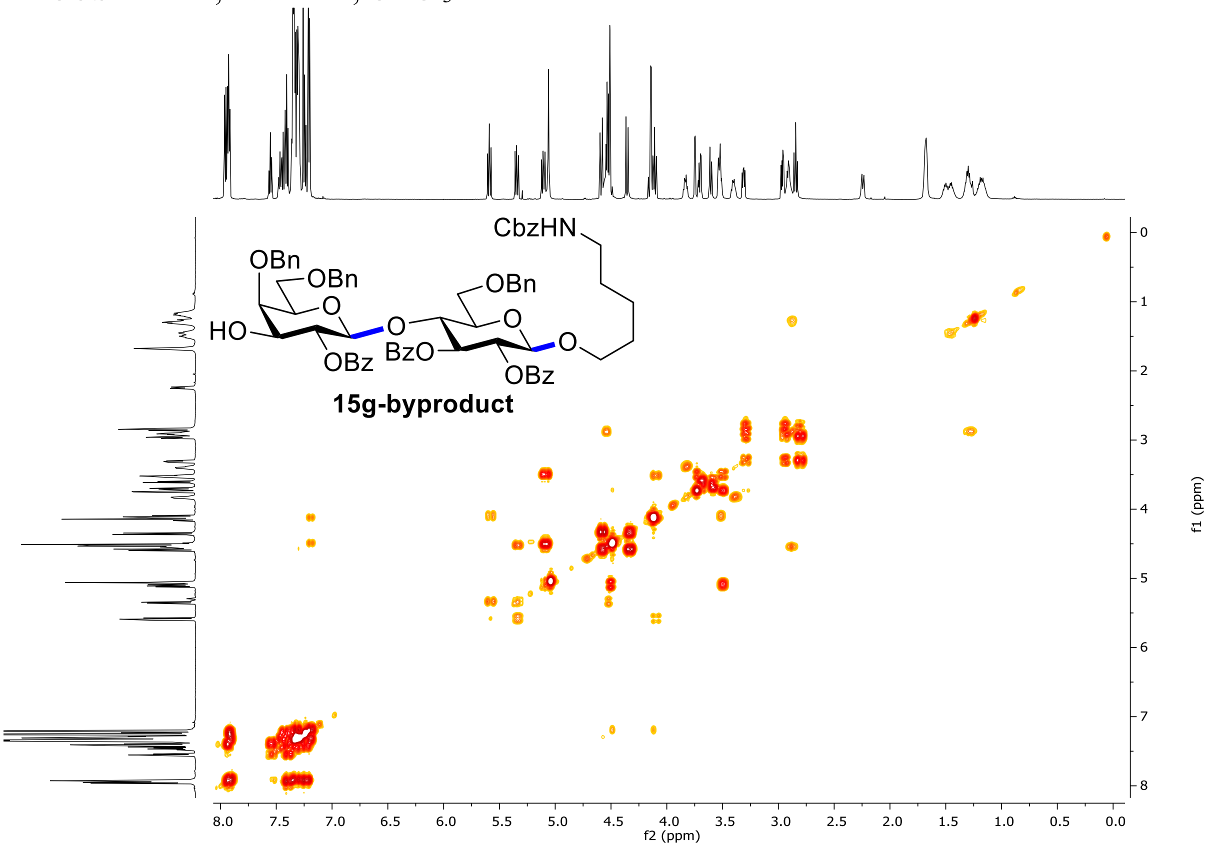

^{1}H-^{13}C-HSQC NMR, 400 MHz, $CDCl_3$

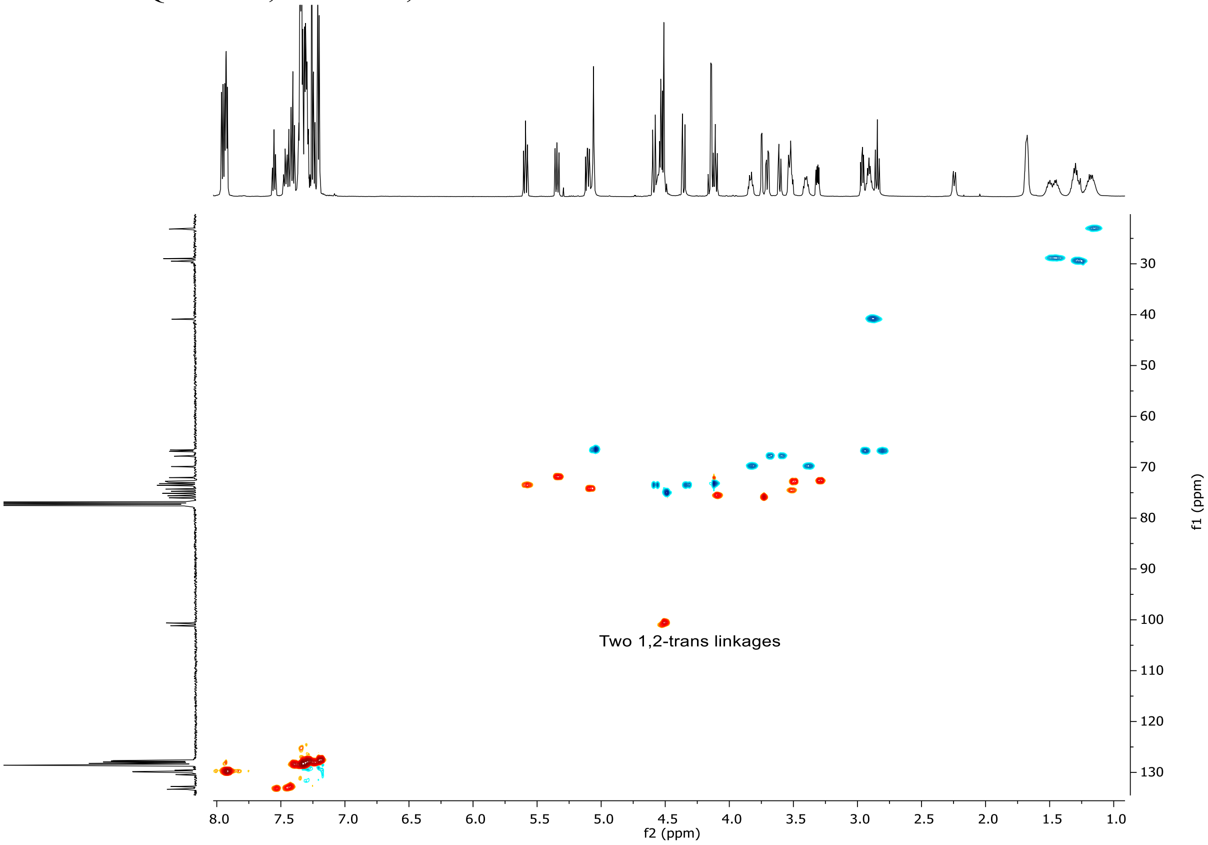

Supplementary Figure 52 | 2D NMR spectra of 15g-byproduct

^{1}H NMR, 600 MHz, $CDCl_3$

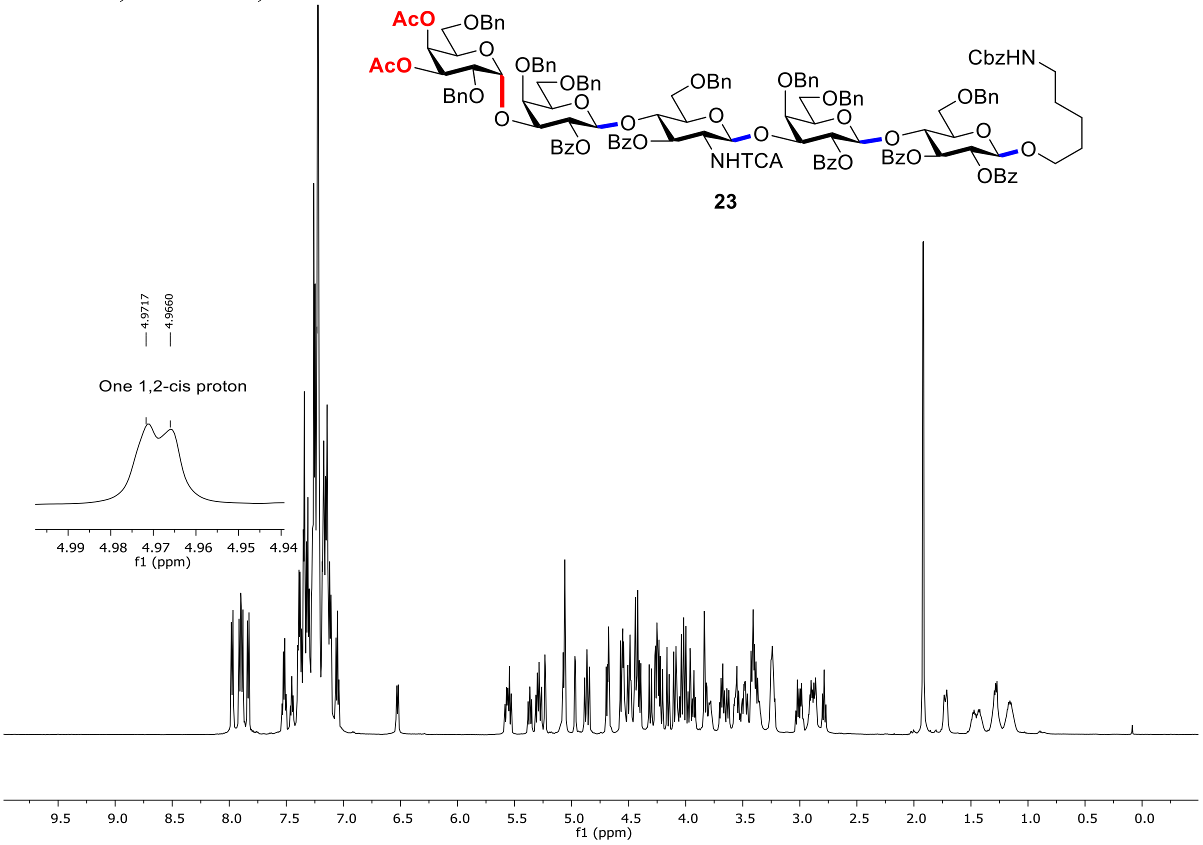

^{13}C NMR, 150 MHz, $CDCl_3$

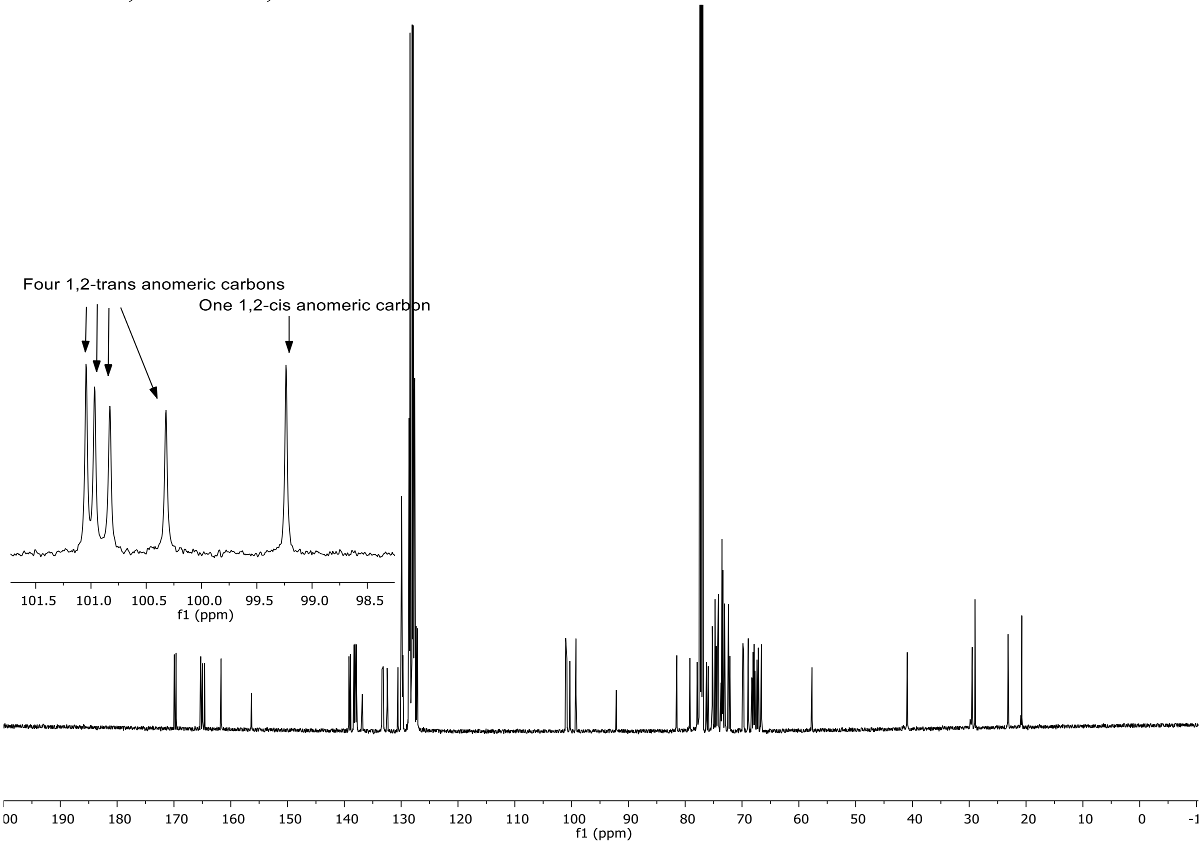

Supplementary Figure 53 | 1D NMR spectra of 23

^{1}H-COSY NMR, 600 MHz, $CDCl_3$

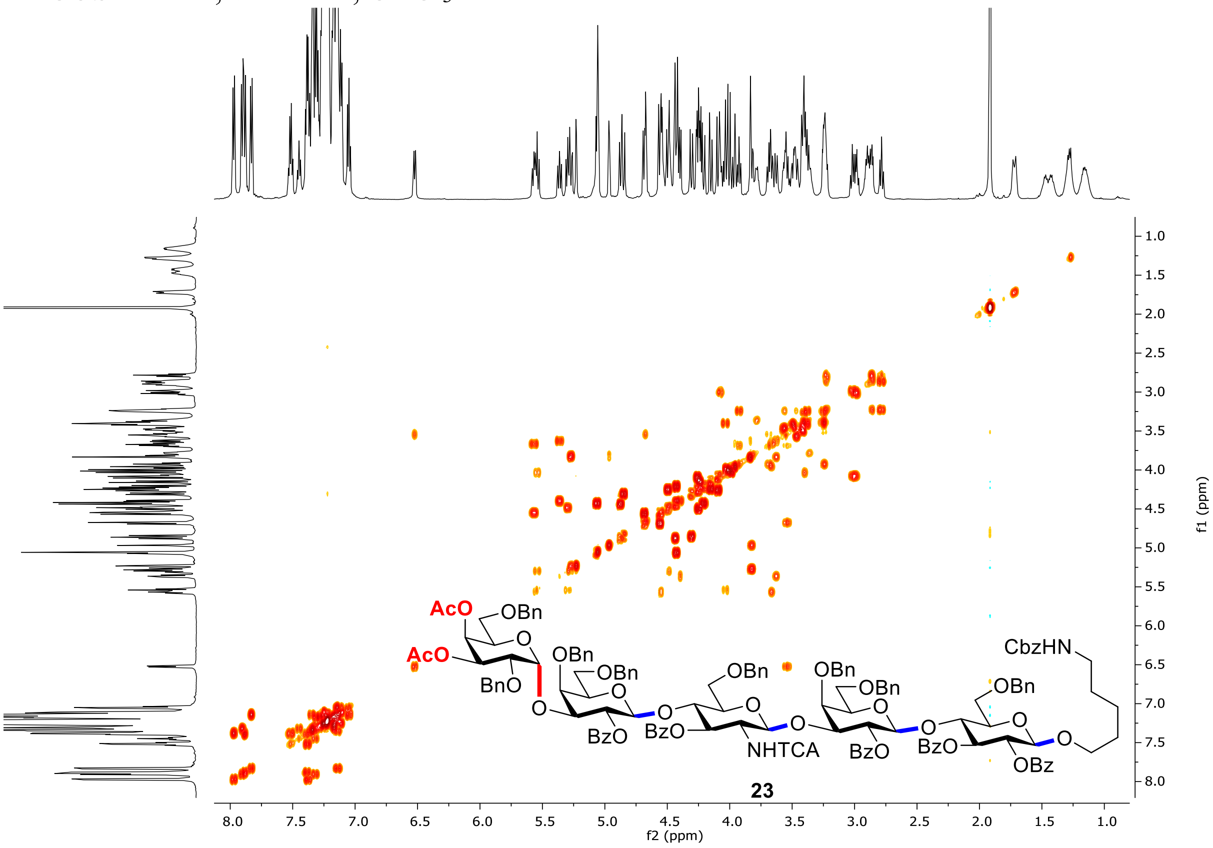

^{1}H-^{13}C-HSQC and ^{1}H-^{13}C-coupled-HSQC (zoom-in) NMR, 600 MHz, $CDCl_3$

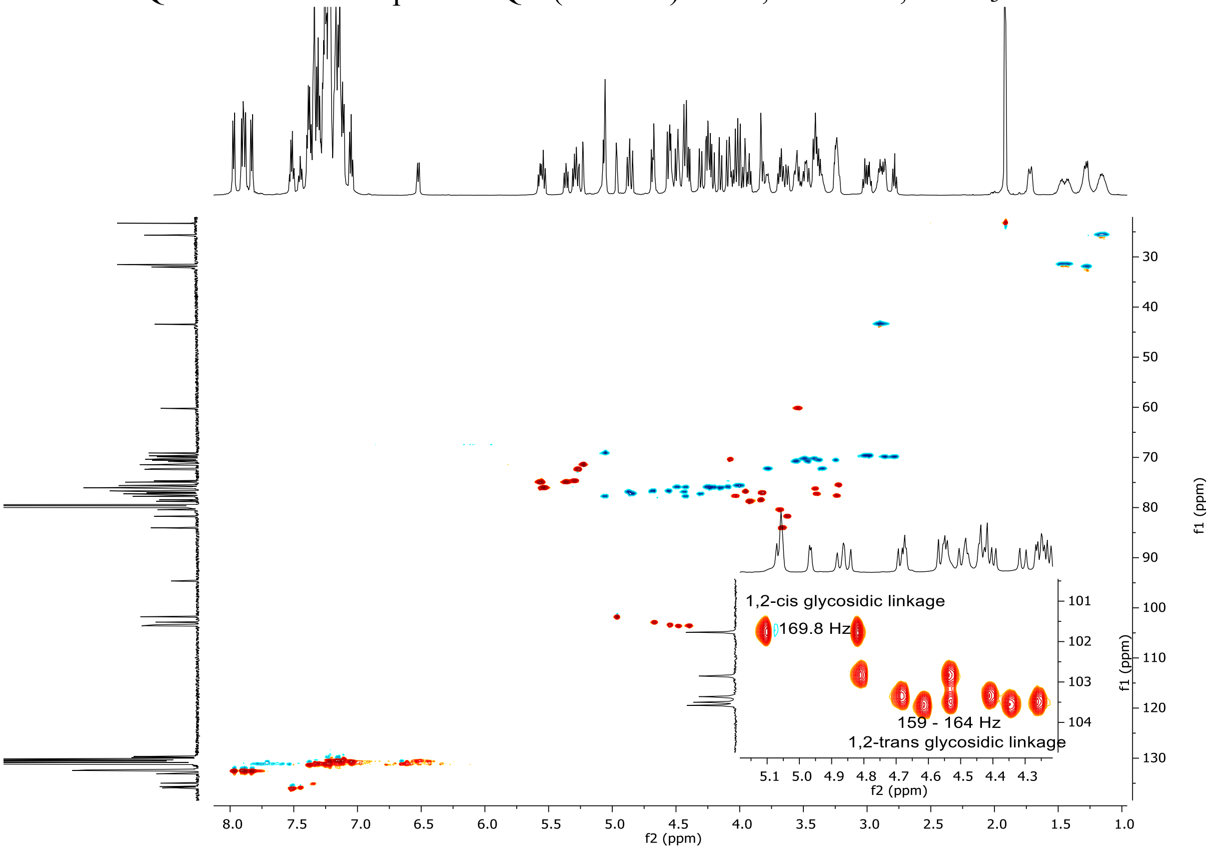

Supplementary Figure 54 | 2D NMR spectra of 23

^{1}H NMR, 600 MHz, $CDCl_3$

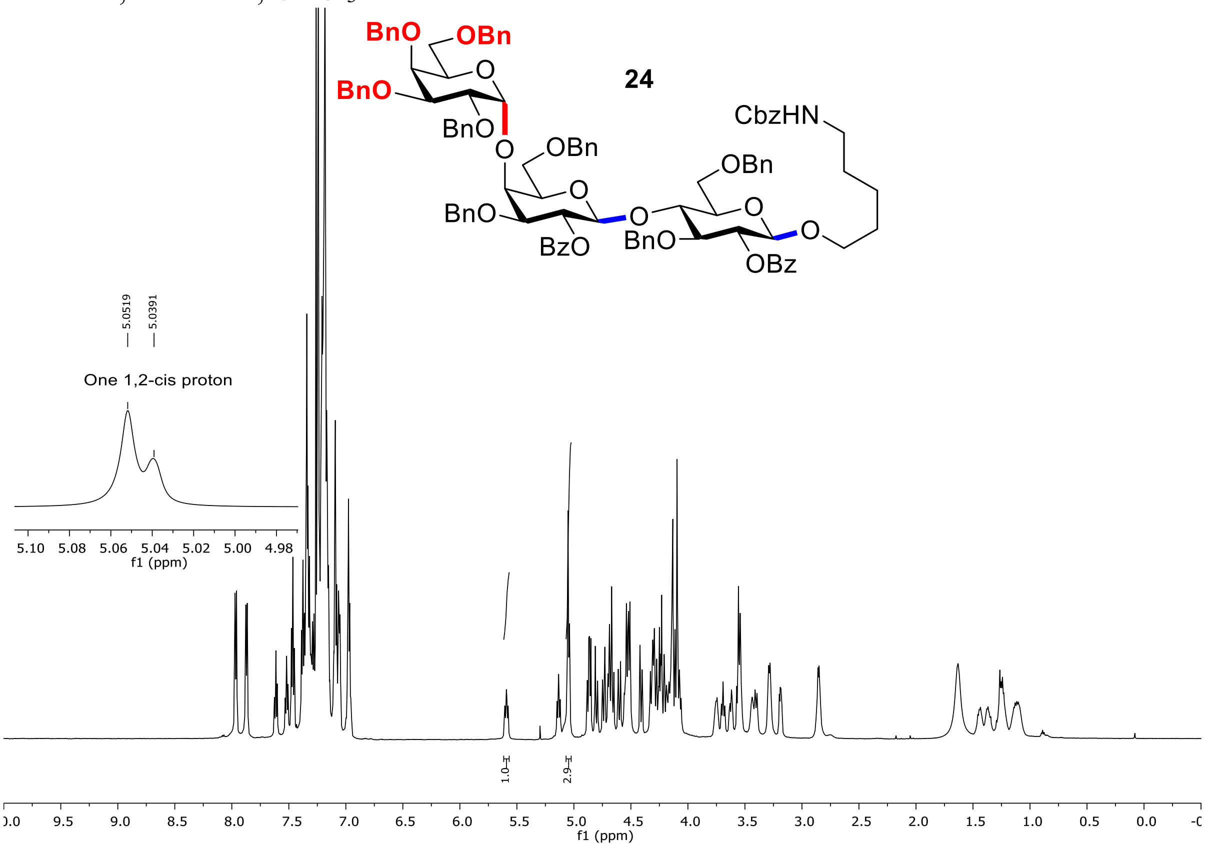

^{13}C NMR, 150 MHz, $CDCl_3$

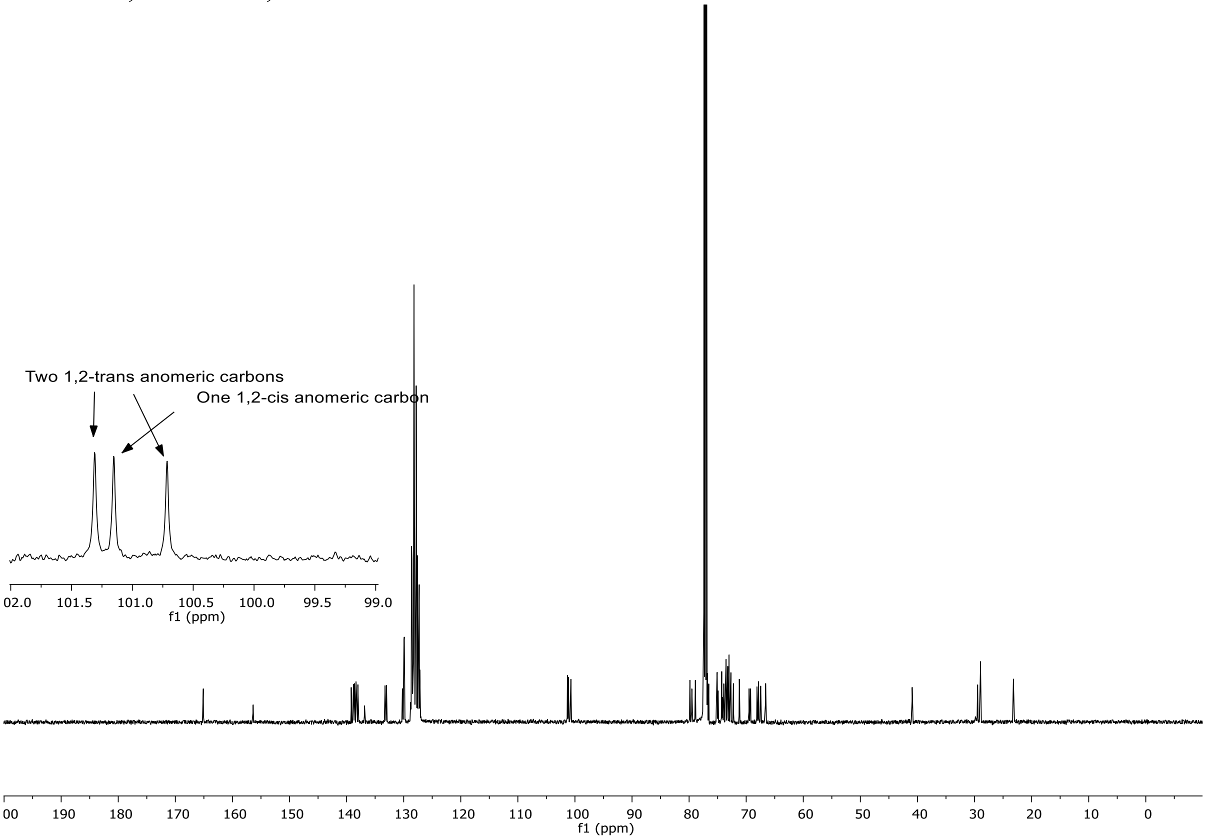

Supplementary Figure 55 | 1D NMR spectra of 24

^{1}H-COSY NMR, 600 MHz, $CDCl_3$

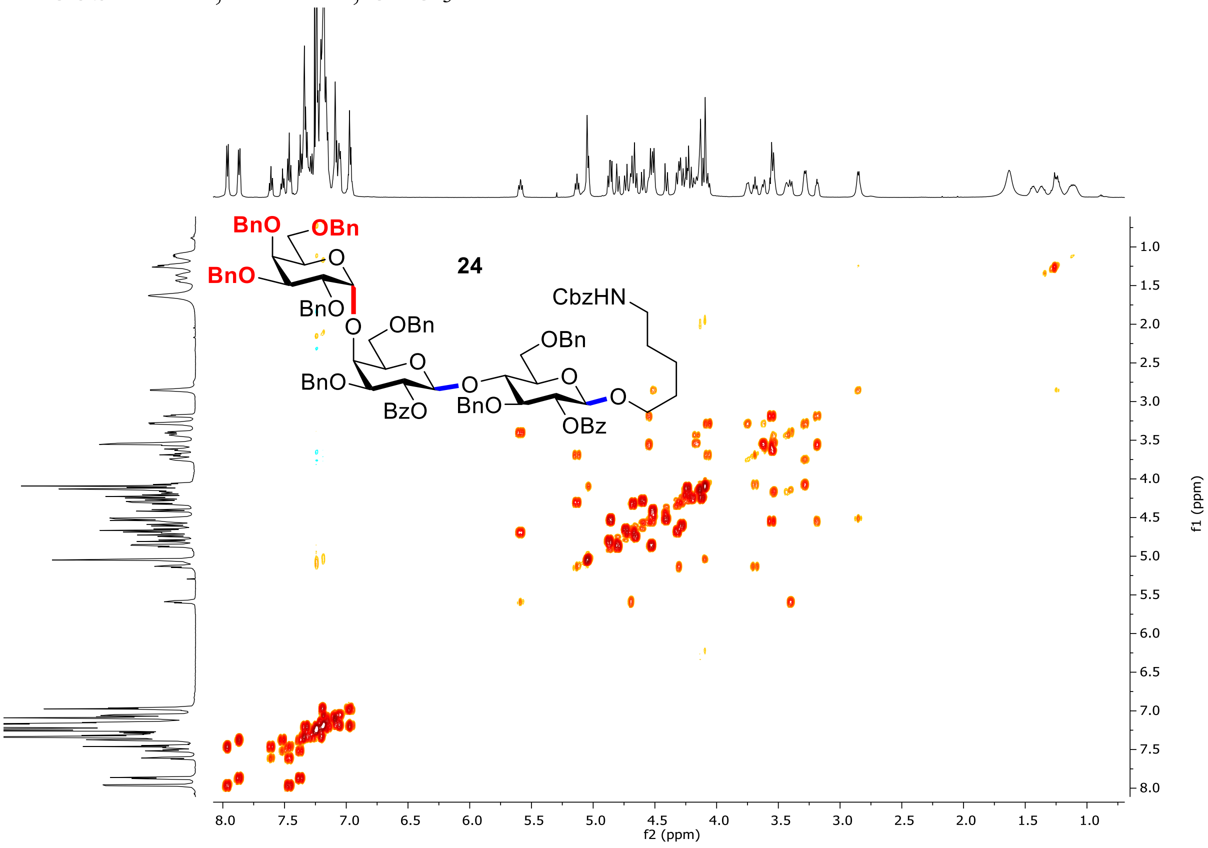

^{1}H-^{13}C-HSQC and ^{1}H-^{13}C-coupled-HSQC (zoom-in) NMR, 600 MHz, $CDCl_3$

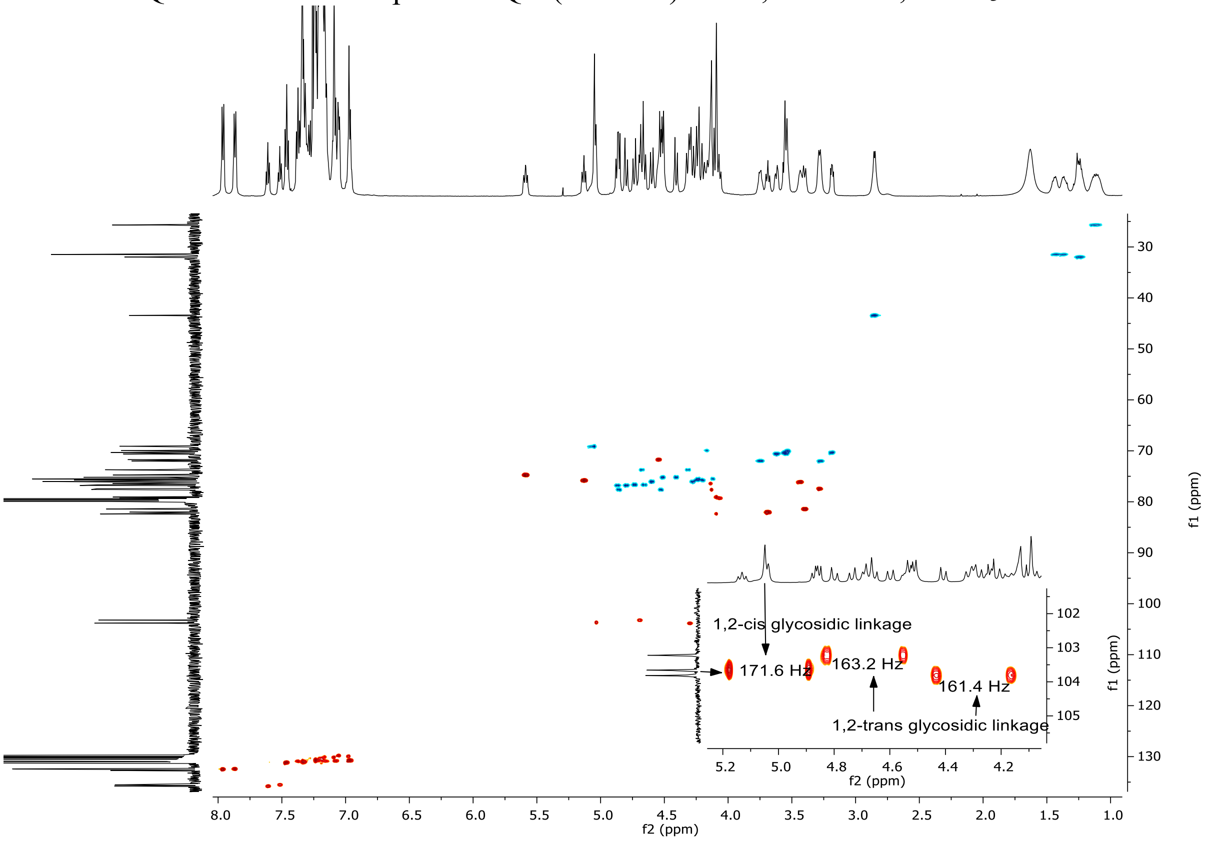

Supplementary Figure 56 | 2D NMR spectra of 24

^{1}H NMR, 600 MHz, $CDCl_3$

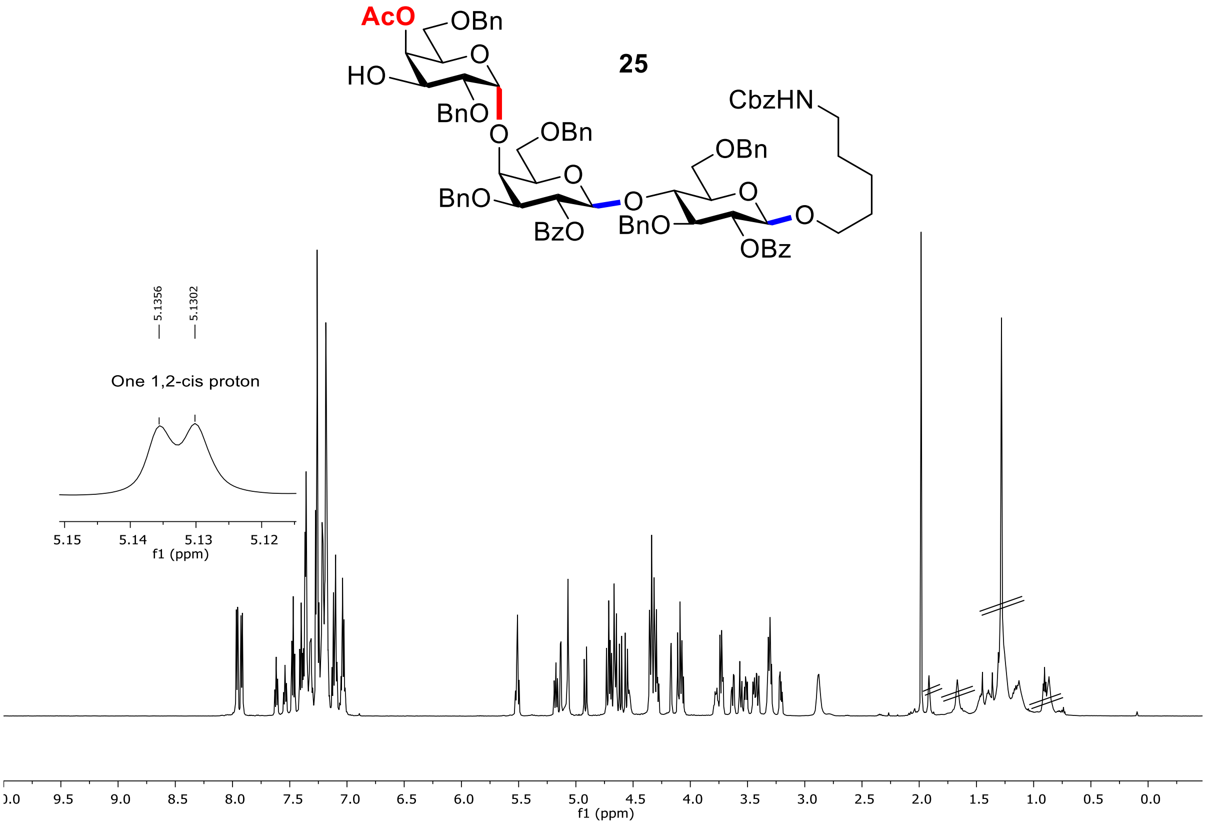

^{13}C NMR, 150 MHz, $CDCl_3$

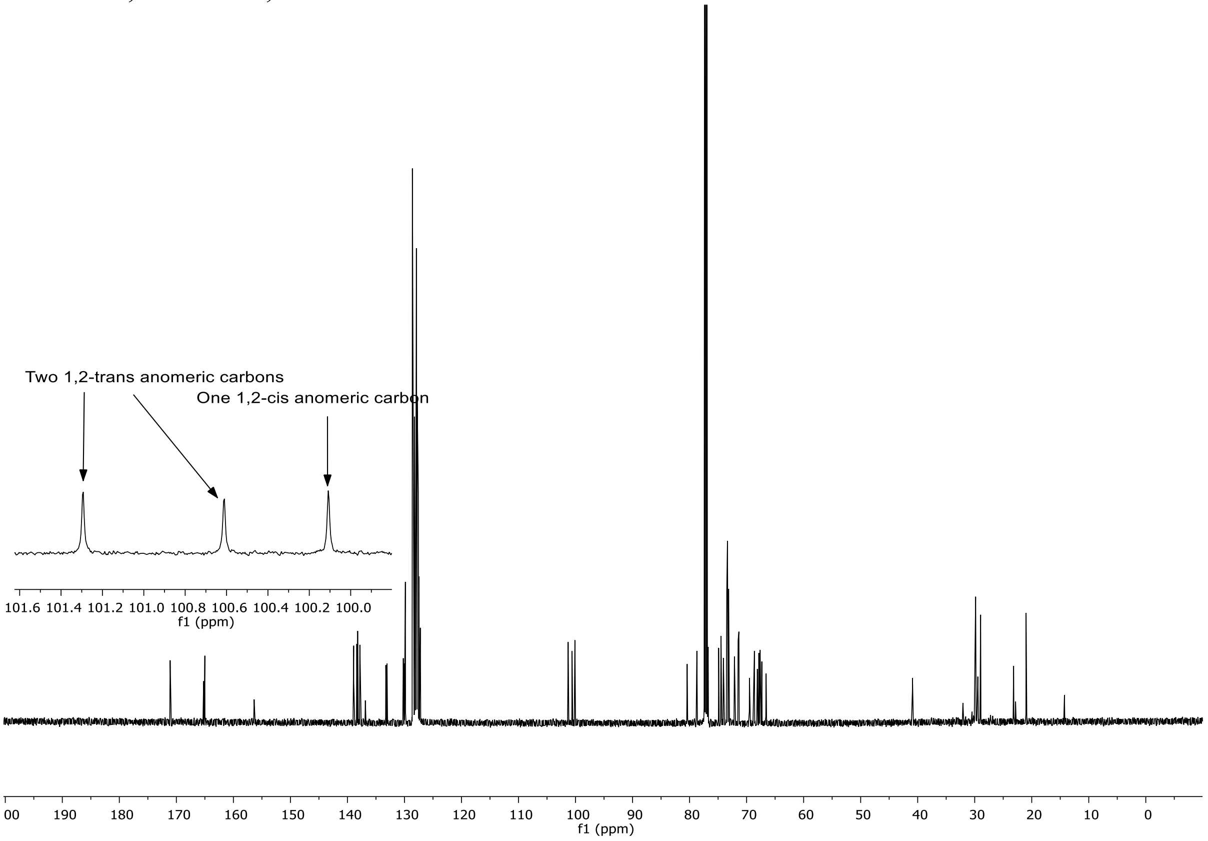

Supplementary Figure 57 | 1D NMR spectra of 25

^{1}H-COSY NMR, 600 MHz, $CDCl_3$

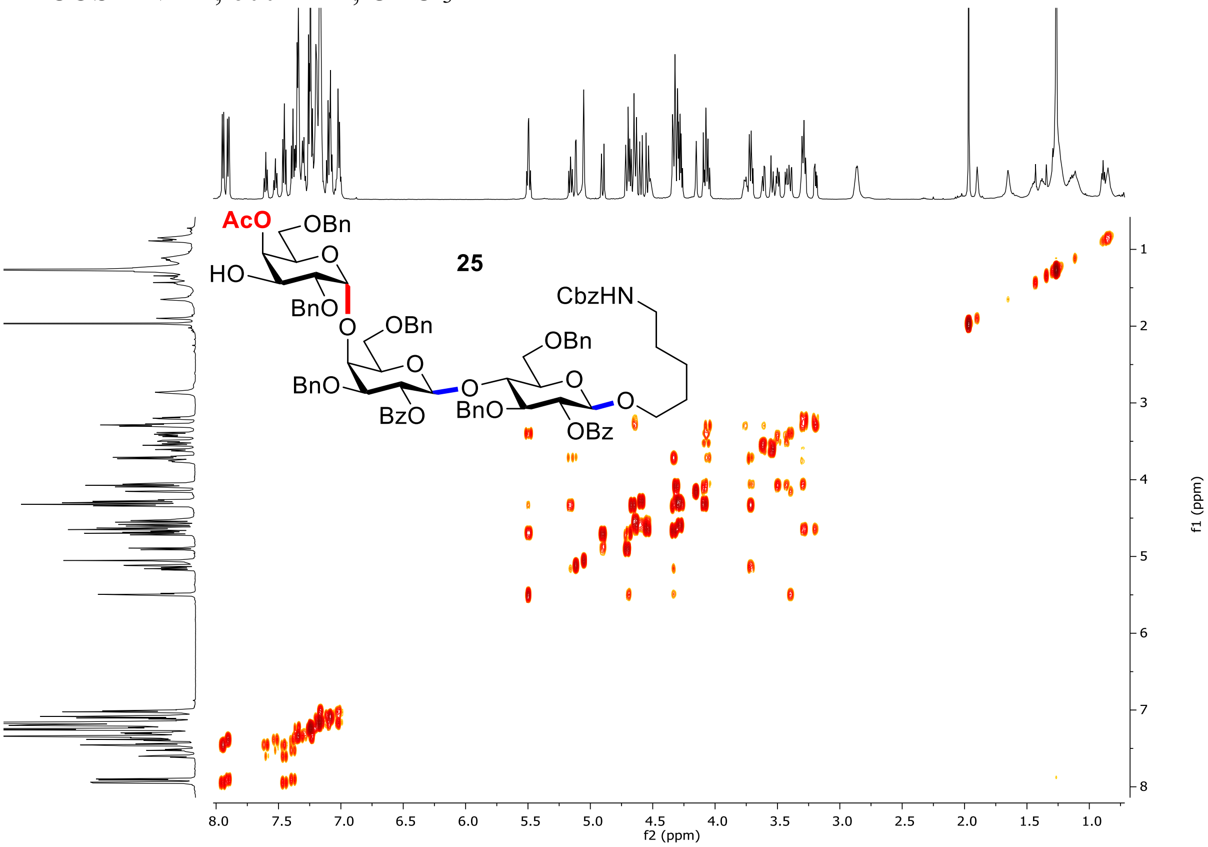

^{1}H-^{13}C-HSQC and ^{1}H-^{13}C-coupled-HSQC (zoom-in) NMR, 600 MHz, $CDCl_3$

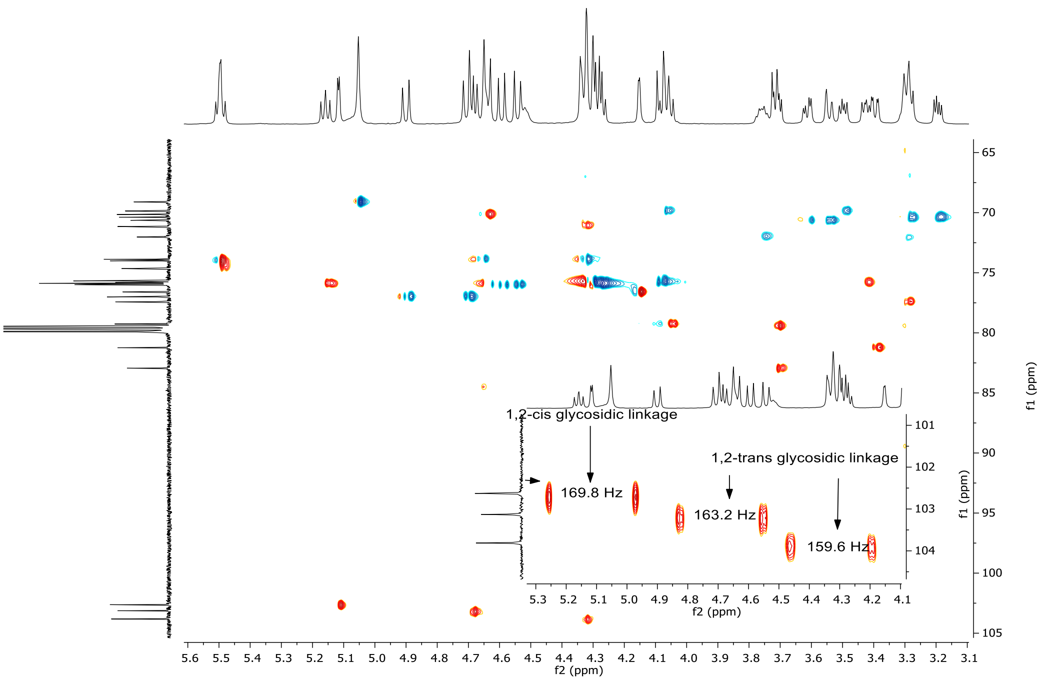

Supplementary Figure 58 | 2D NMR spectra of 25

^{1}H NMR, 600 MHz, $CDCl_3$

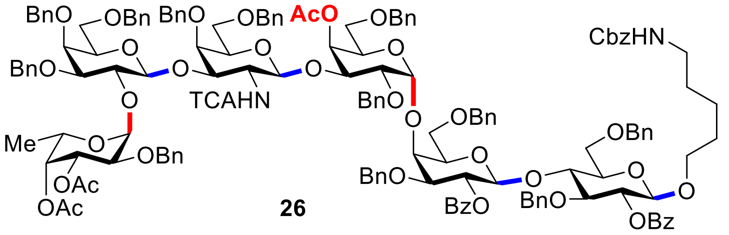

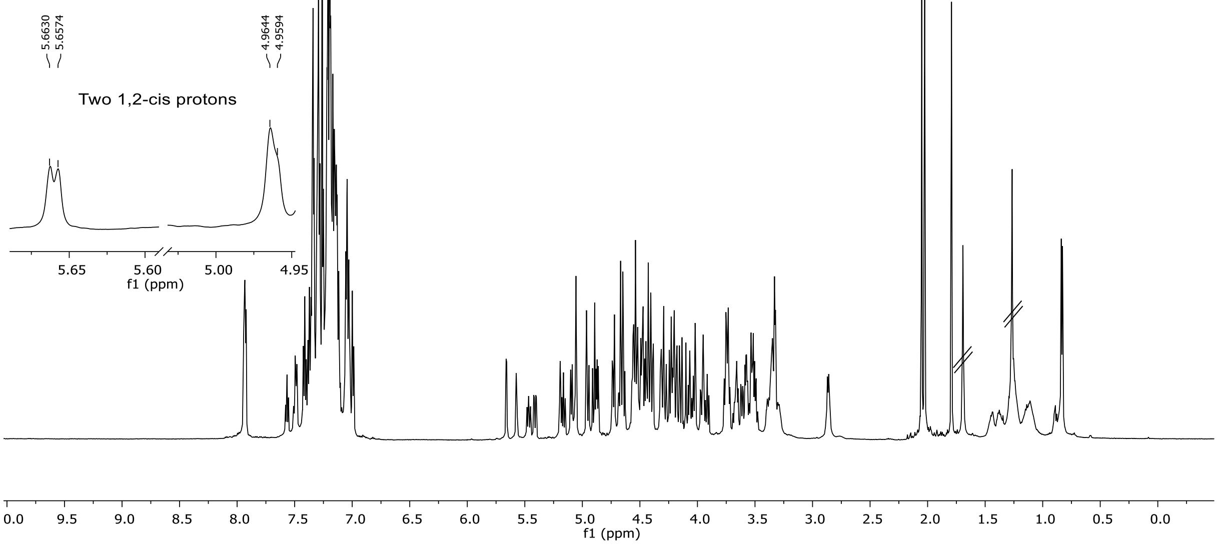

^{13}C NMR, 150 MHz, $CDCl_3$

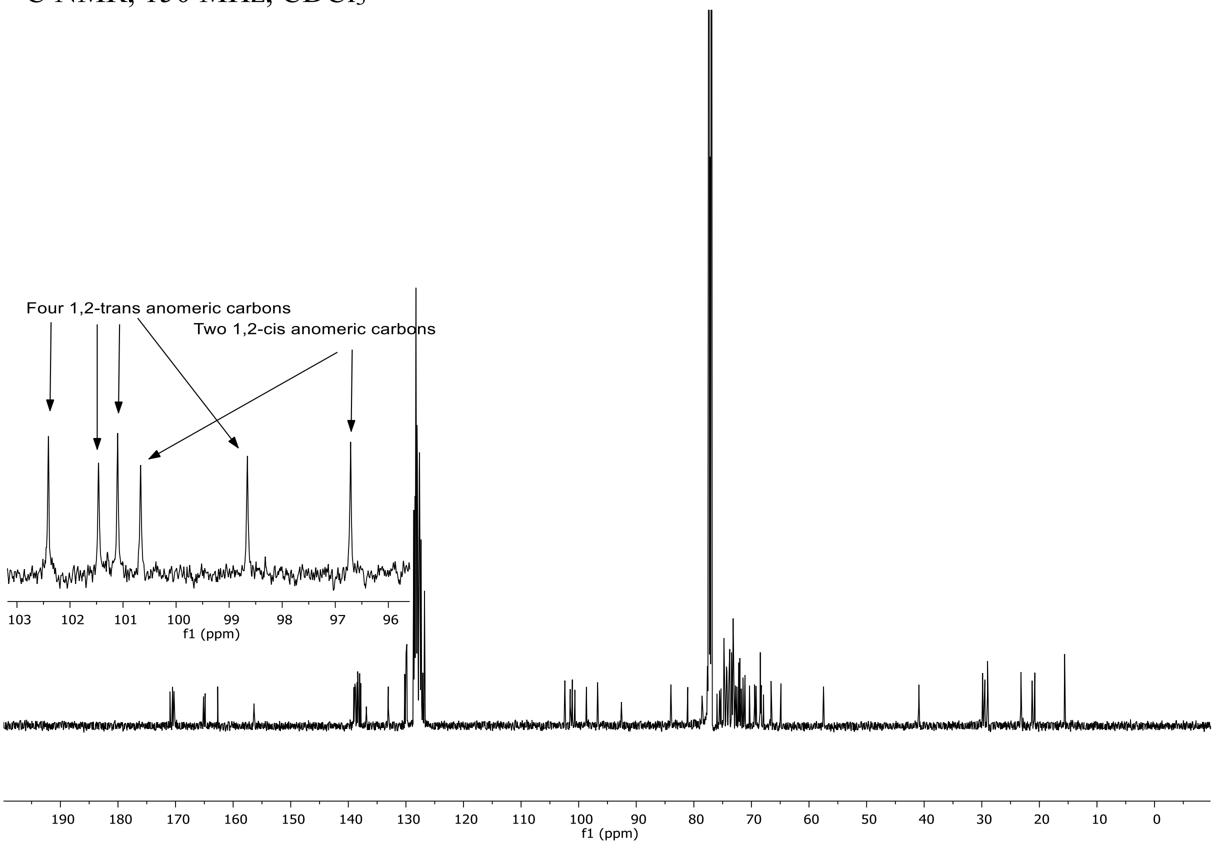

Supplementary Figure 59 | 1D NMR spectra of 26

^{1}H-COSY NMR, 600 MHz, $CDCl_3$

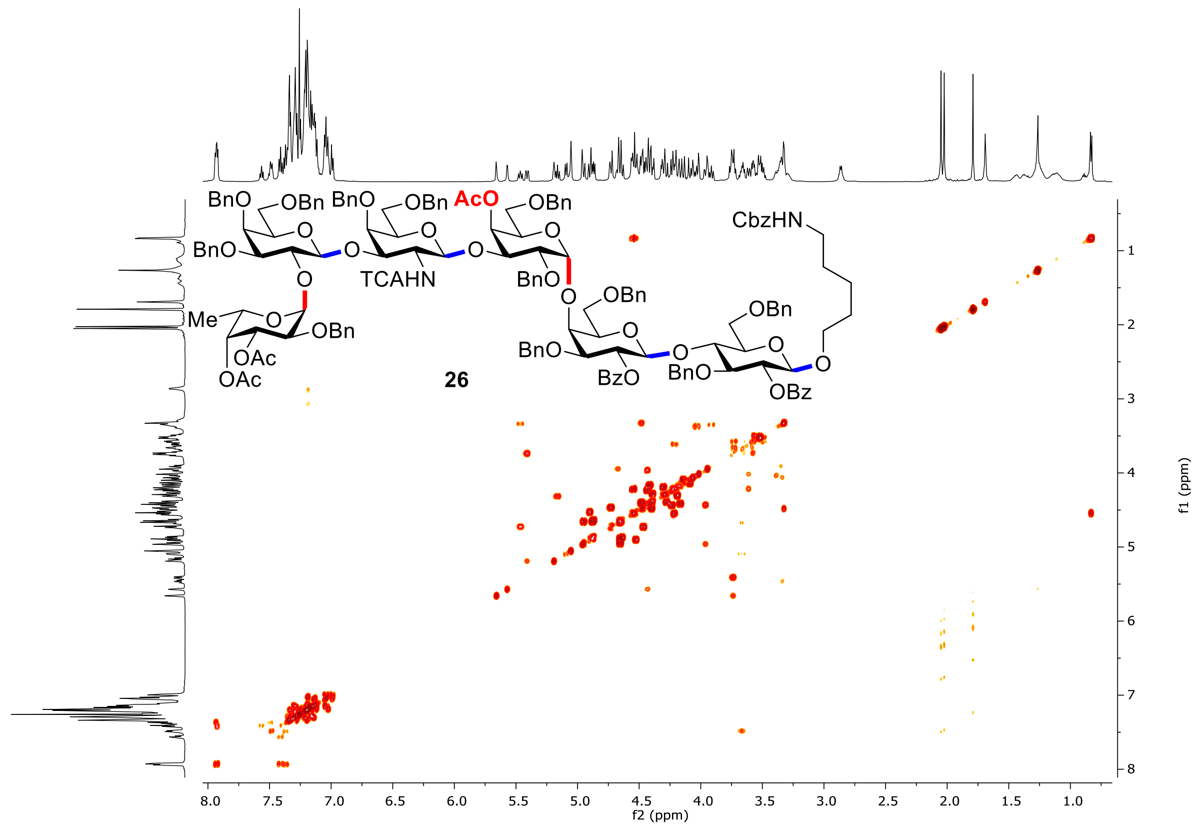

^{1}H-^{13}C-HSQC and ^{1}H-^{13}C-coupled-HSQC (zoom-in) NMR, 600 MHz, $CDCl_3$

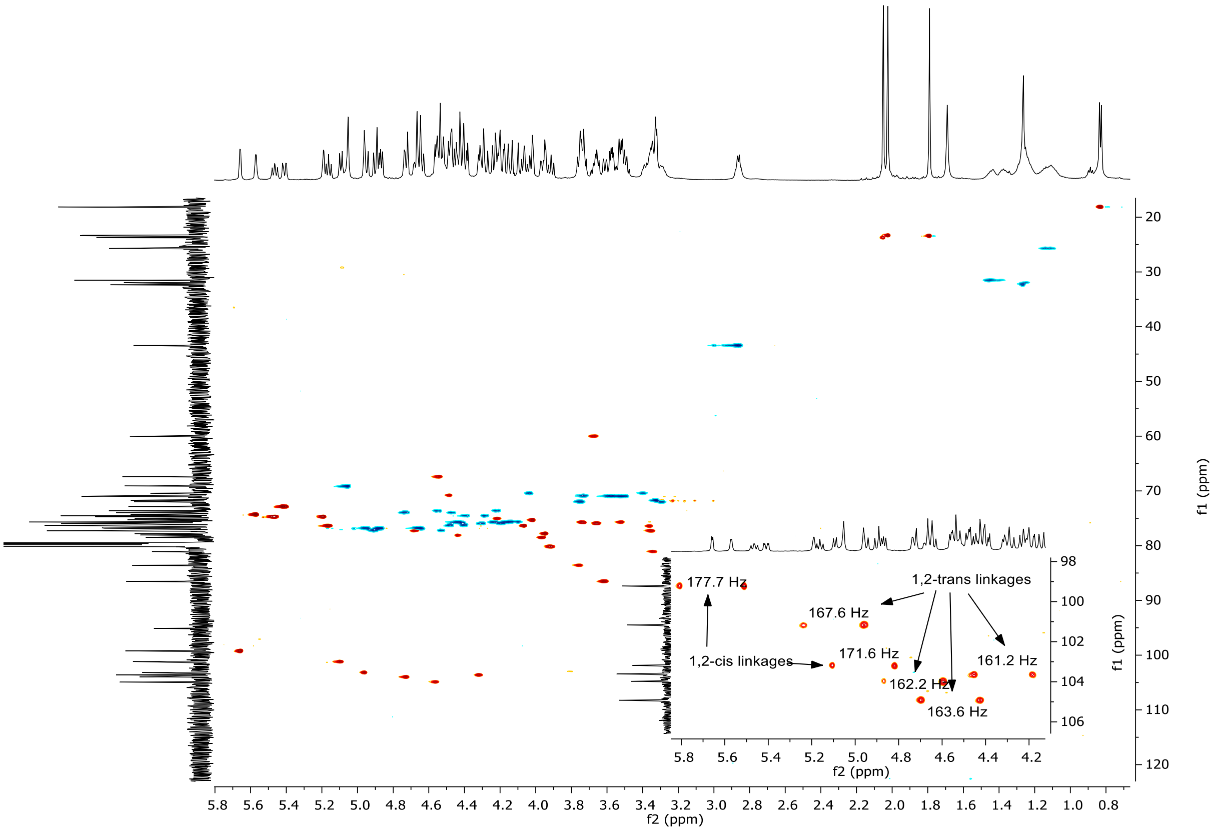

Supplementary Figure 60 | 2D NMR spectra of 26

^{1}H NMR, 400 MHz, $CDCl_3$

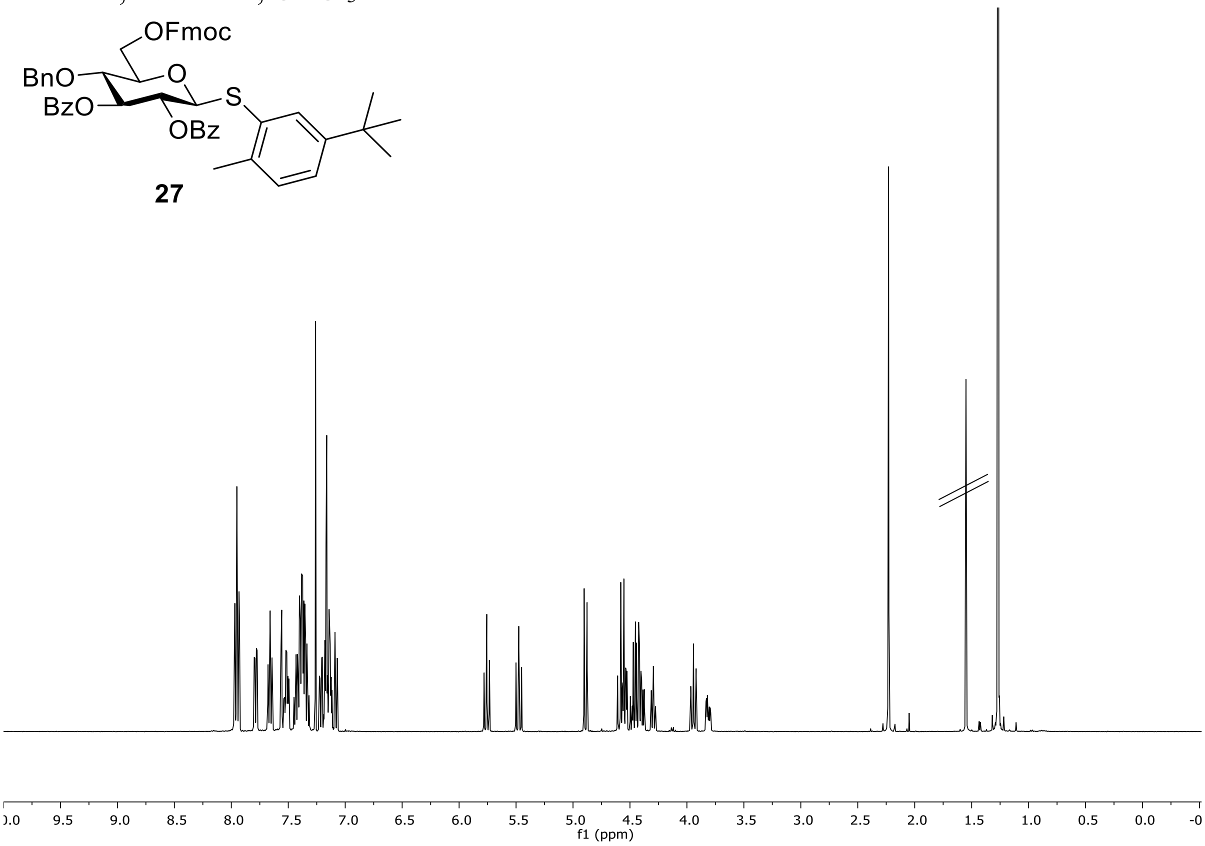

^{13}C NMR, 100 MHz, $CDCl_3$

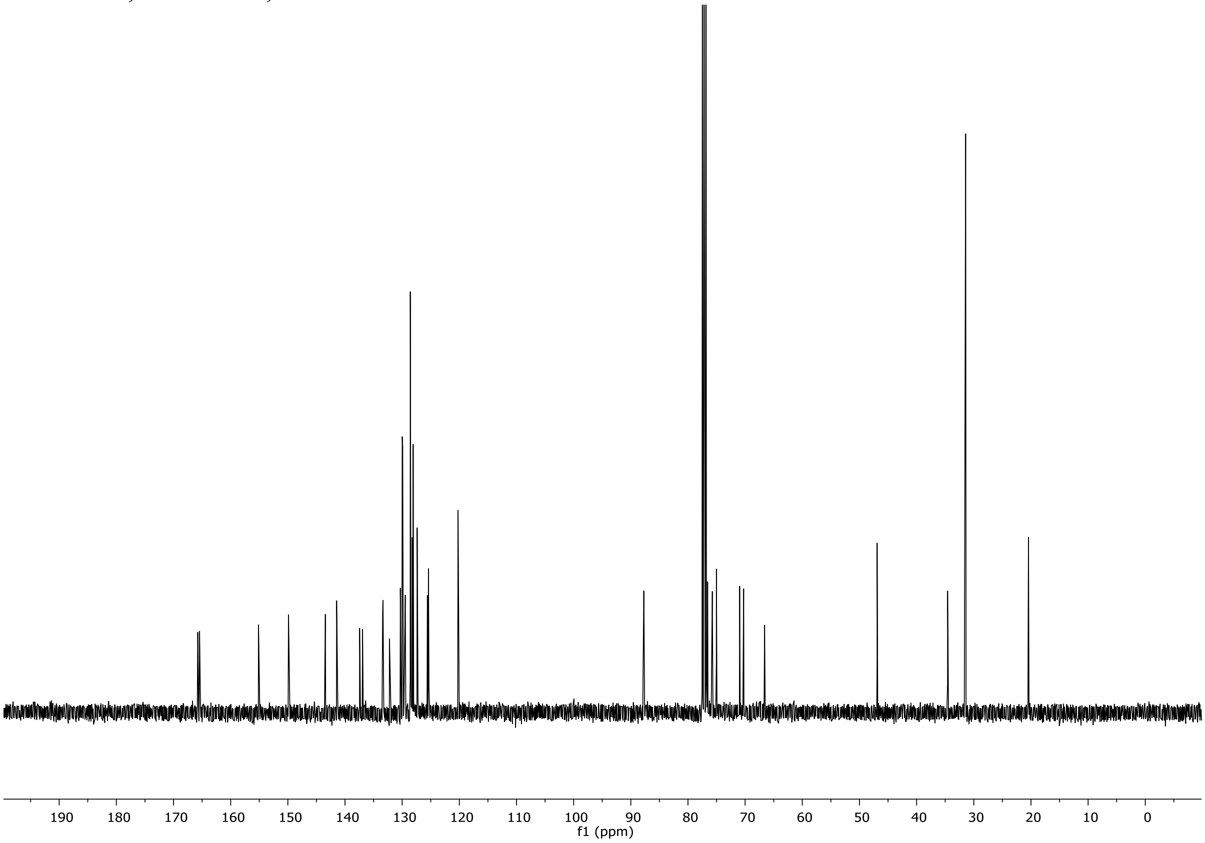

Supplementary Figure 61 | 1D NMR spectra of 27

^{1}H-COSY NMR, 400 MHz, $CDCl_3$

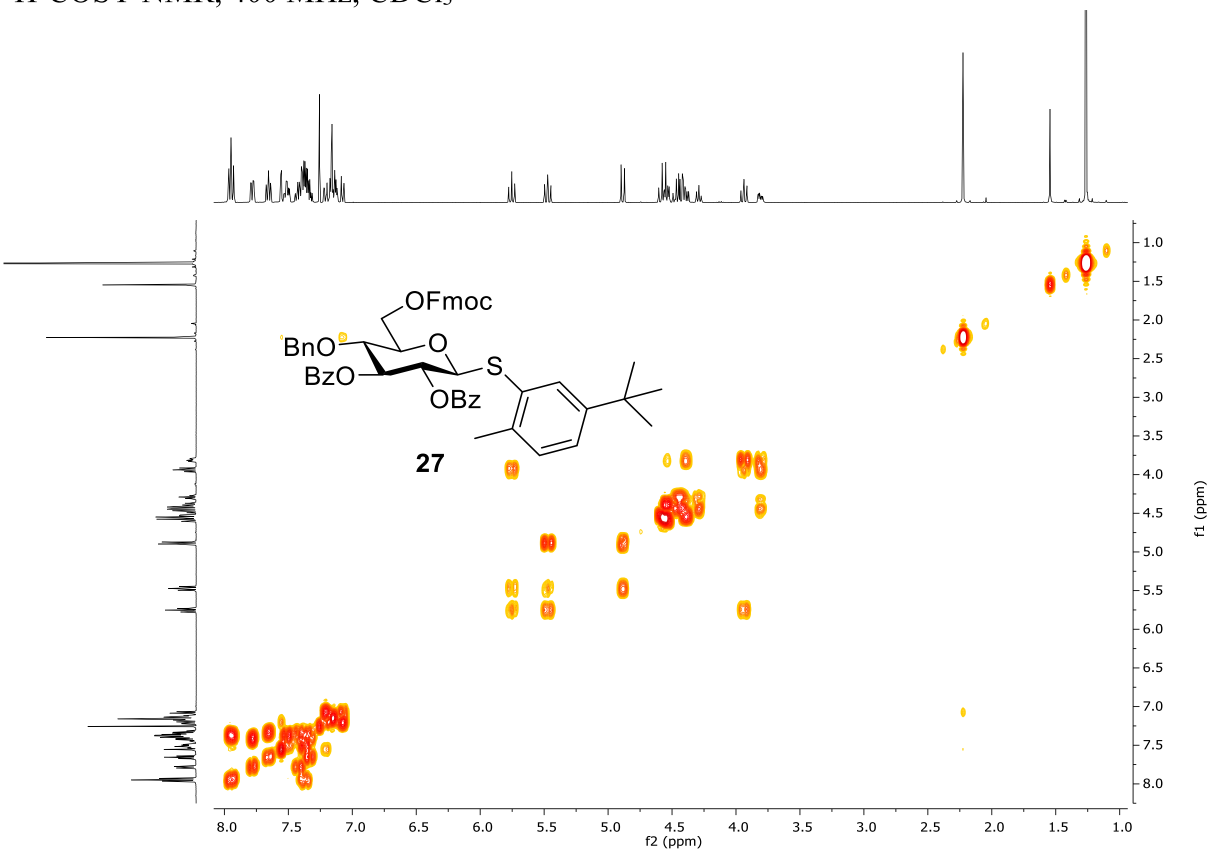

^{1}H-^{13}C-HSQC NMR, 400 MHz, $CDCl_3$

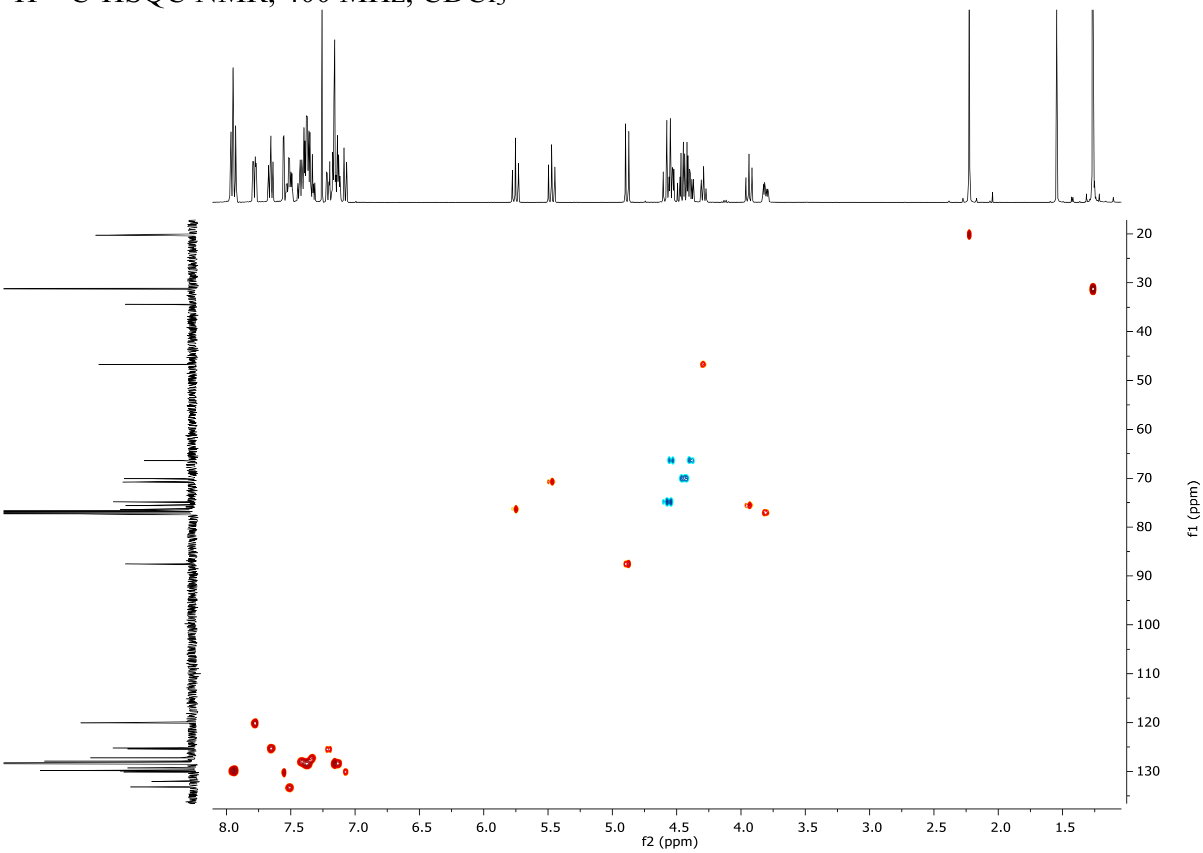

Supplementary Figure 62 | 2D NMR spectra of 27

^{1}H NMR, 400 MHz, $CDCl_3$

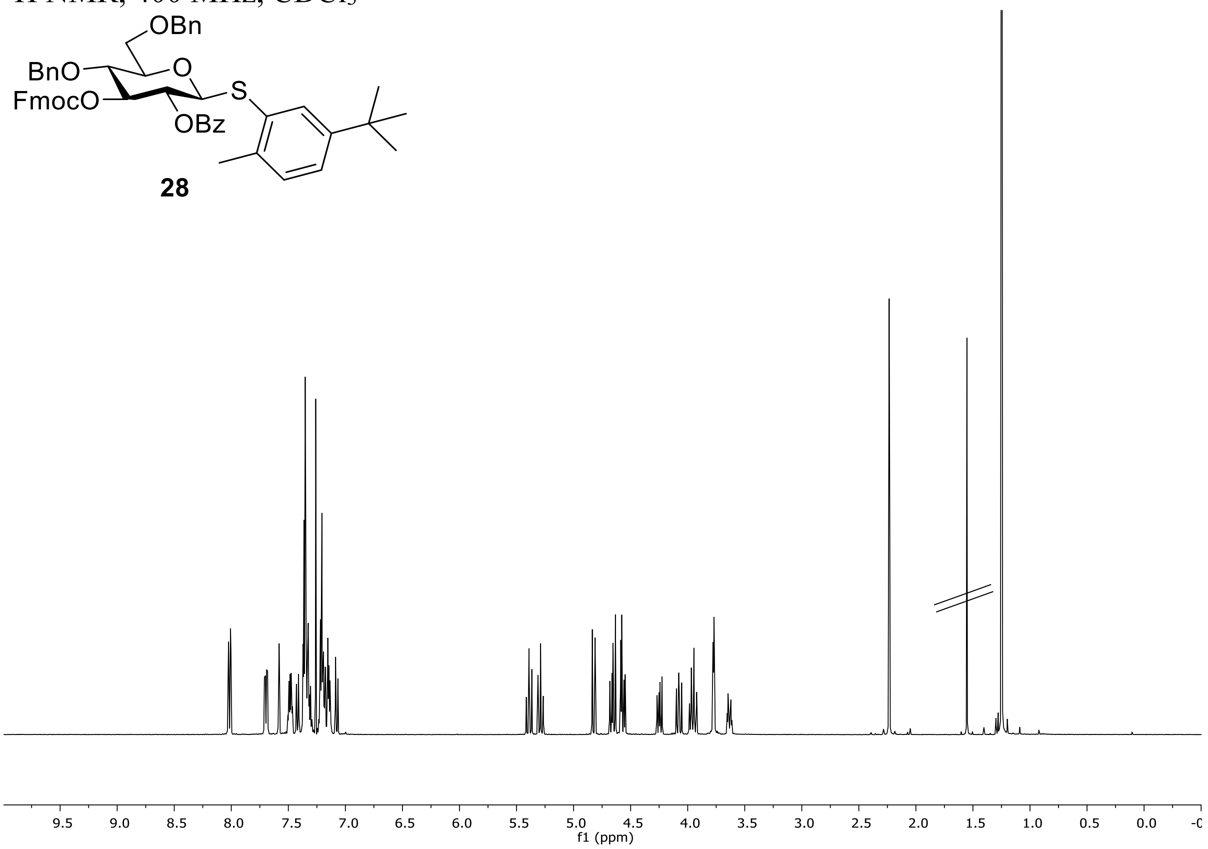

^{13}C NMR, 100 MHz, $CDCl_3$

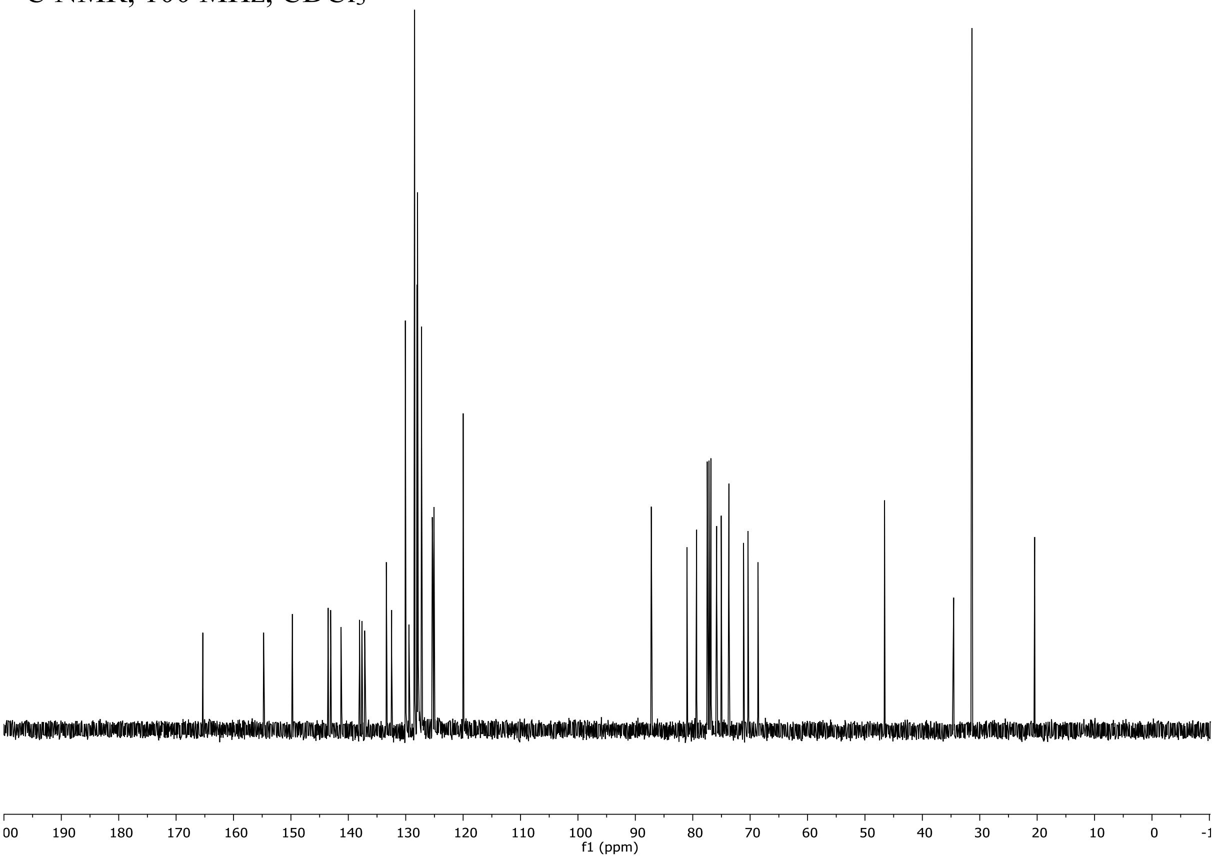

Supplementary Figure 63 | 1D NMR spectra of 28

^{1}H-COSY NMR, 400 MHz, $CDCl_3$

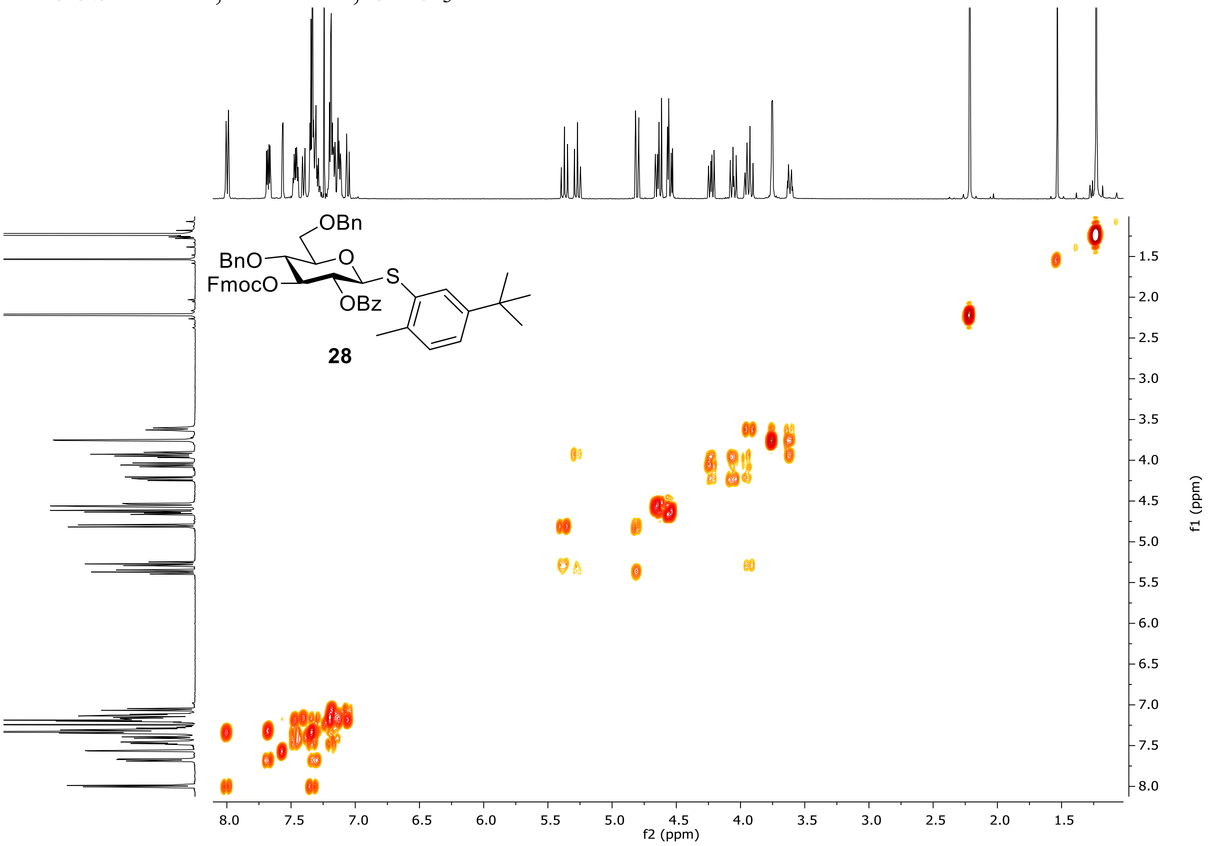

^{1}H-^{13}C-HSQC NMR, 400 MHz, $CDCl_3$

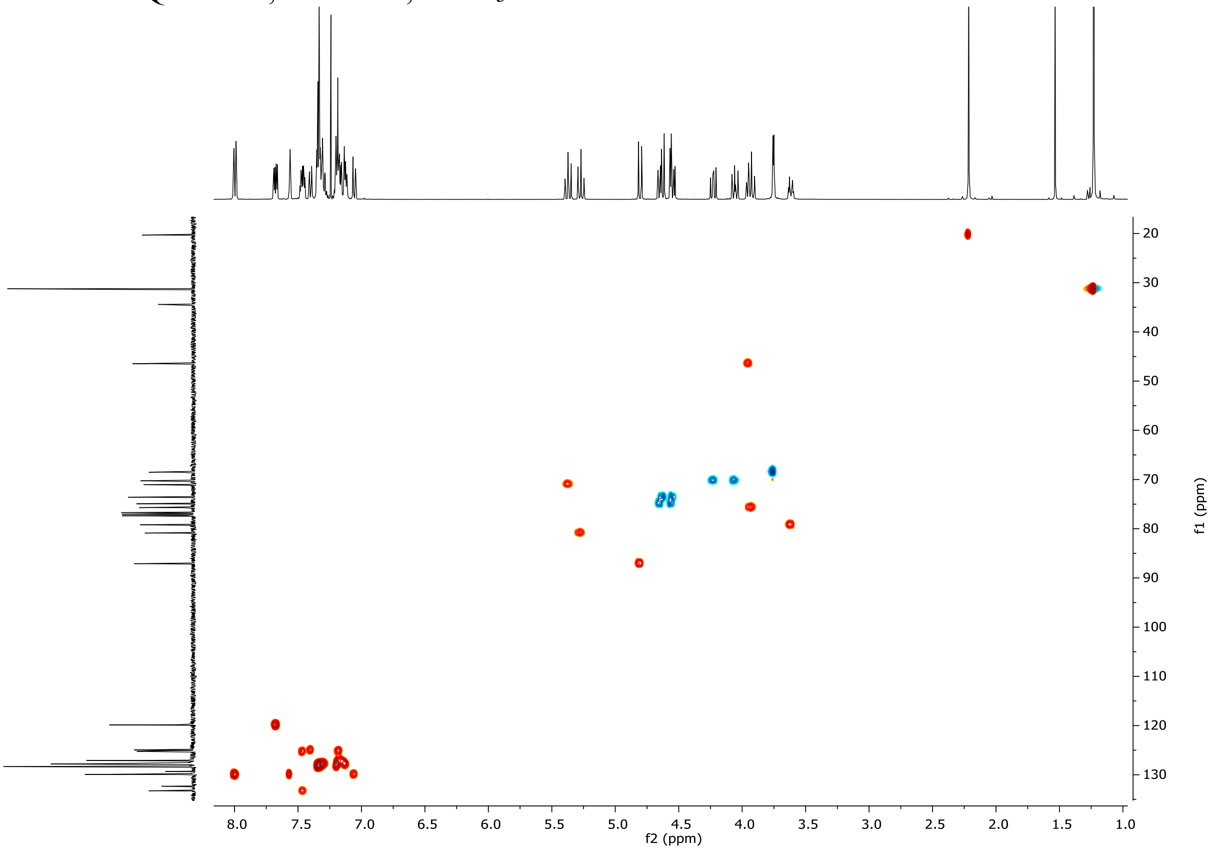

Supplementary Figure 64 | 2D NMR spectra of 28

^{1}H NMR, 400 MHz, $CDCl_3$

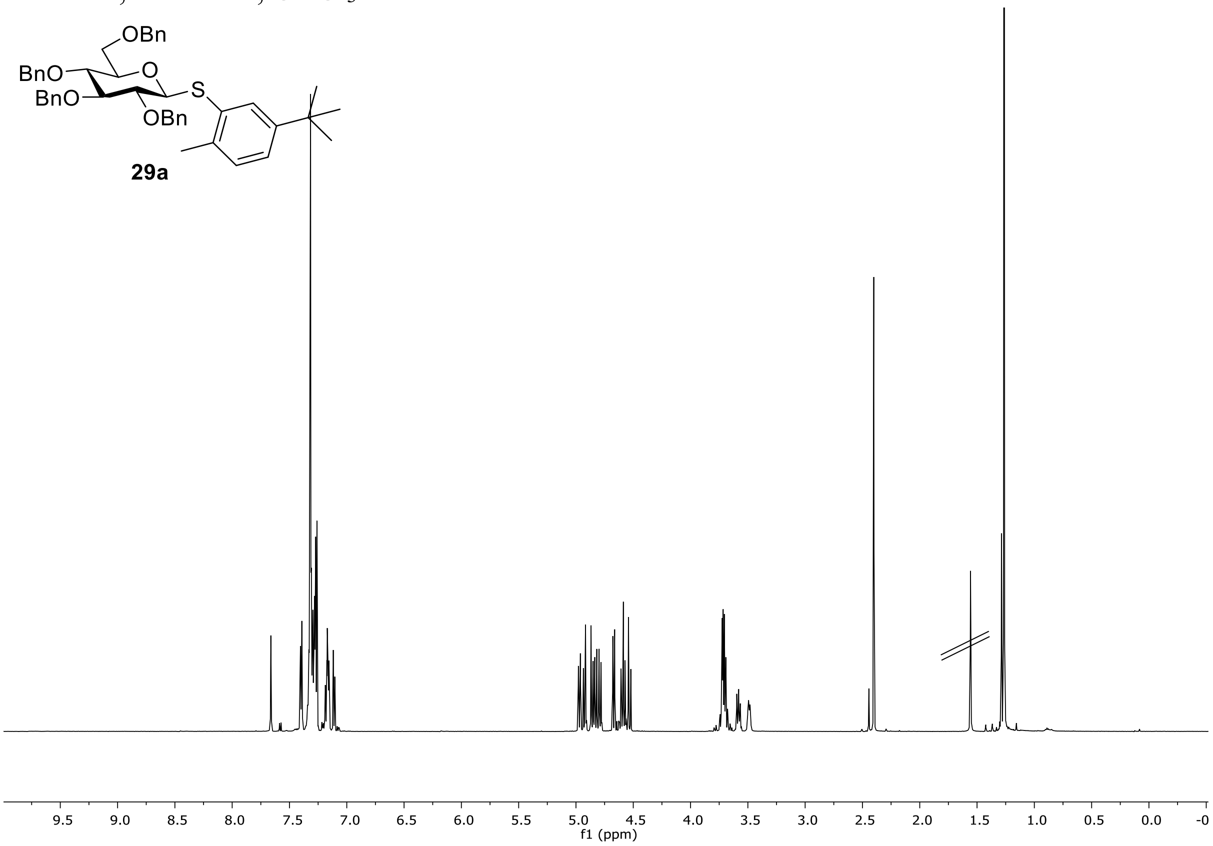

^{13}C NMR, 100 MHz, $CDCl_3$

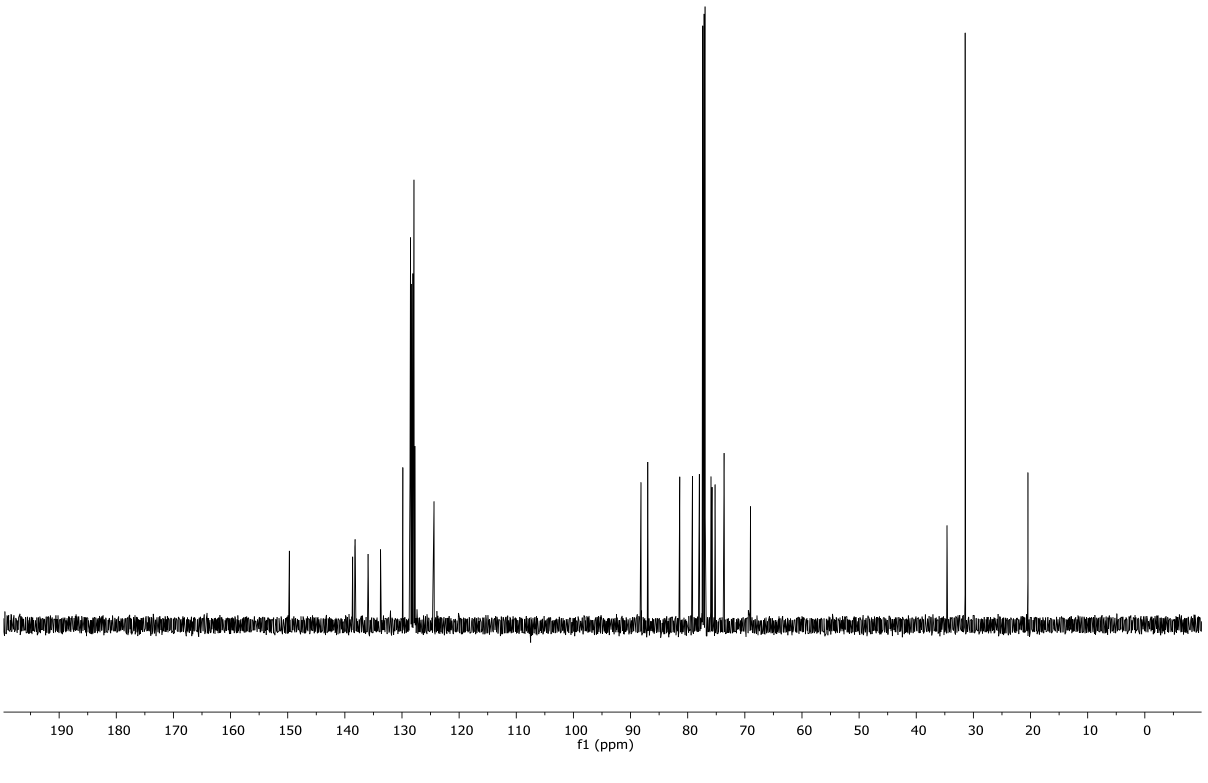

Supplementary Figure 65 | 1D NMR spectra of 29a

^{1}H-COSY NMR, 400 MHz, $CDCl_3$

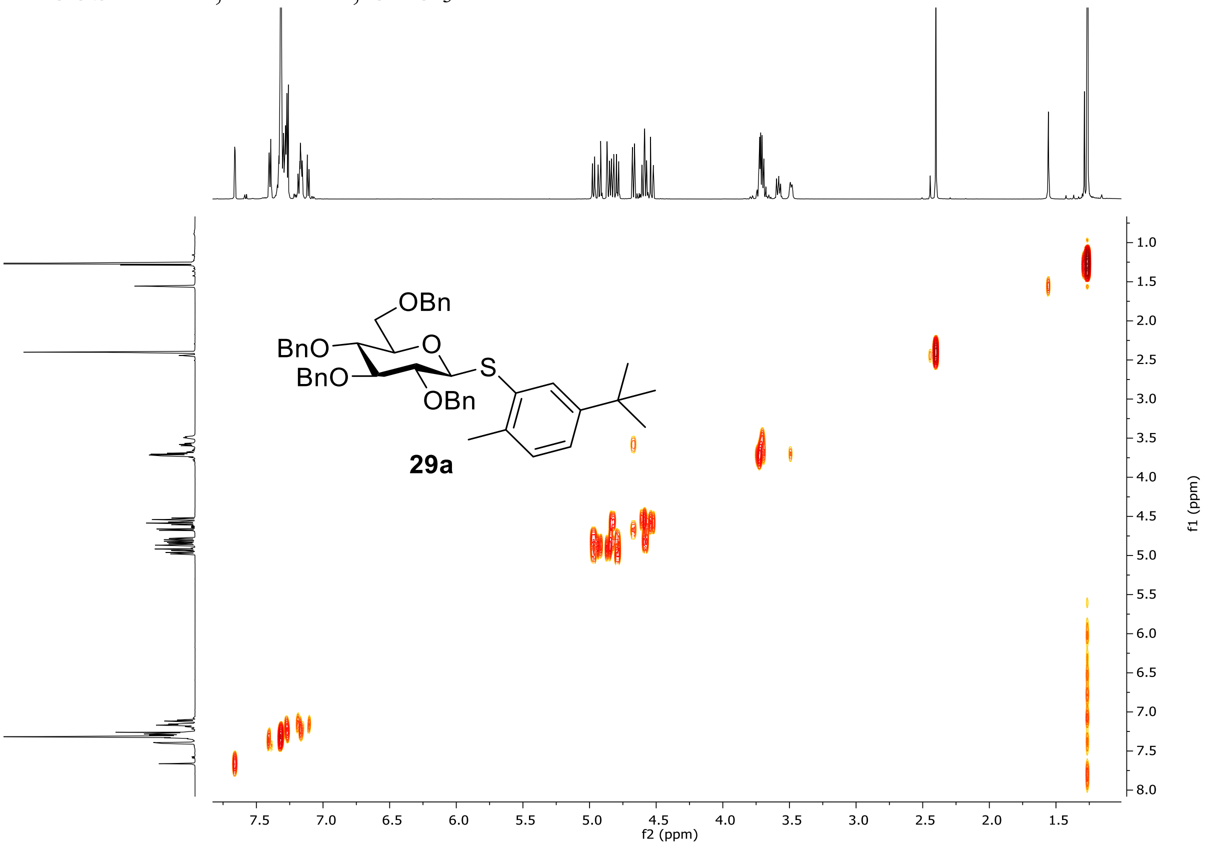

^{1}H-^{13}C-HSQC NMR, 400 MHz, $CDCl_3$

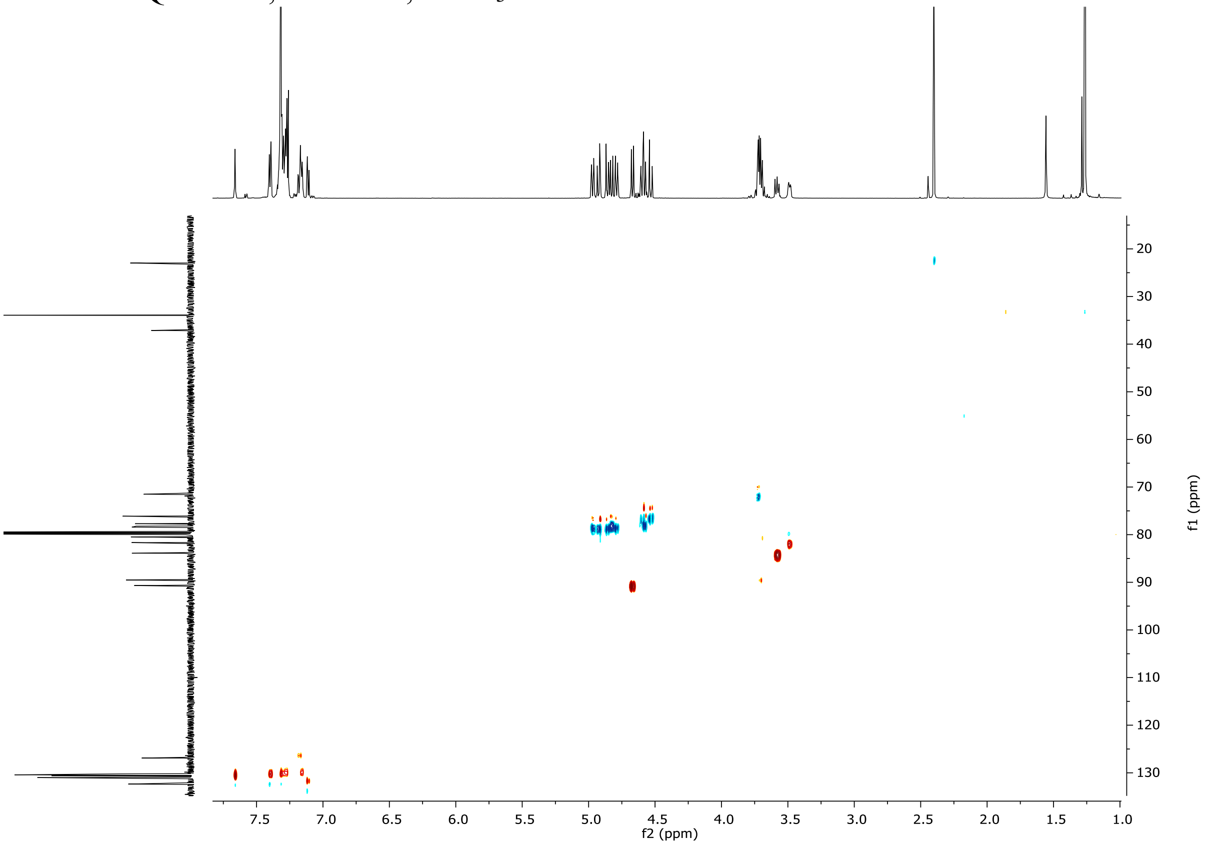

Supplementary Figure 66 | 2D NMR spectra of 29a

^{1}H NMR, 400 MHz, $CDCl_3$

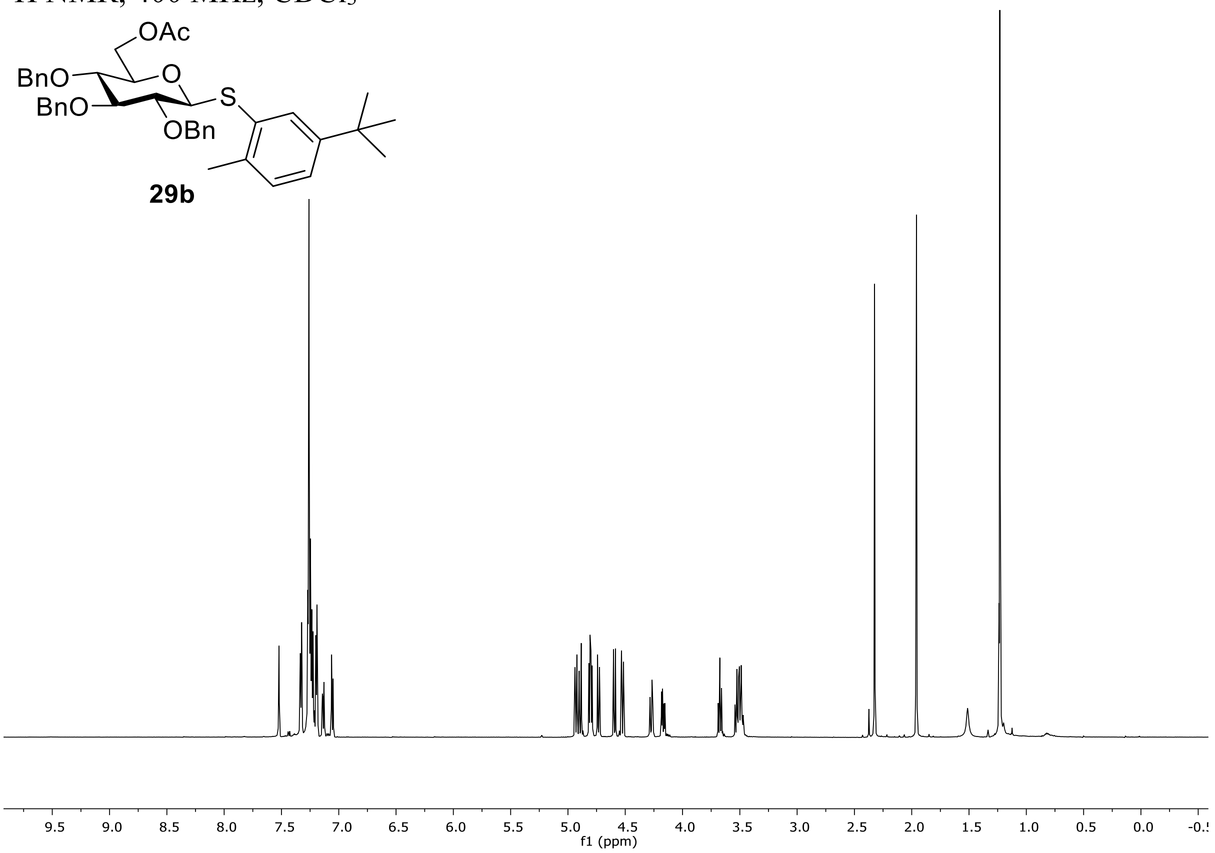

^{13}C NMR, 100 MHz, $CDCl_3$

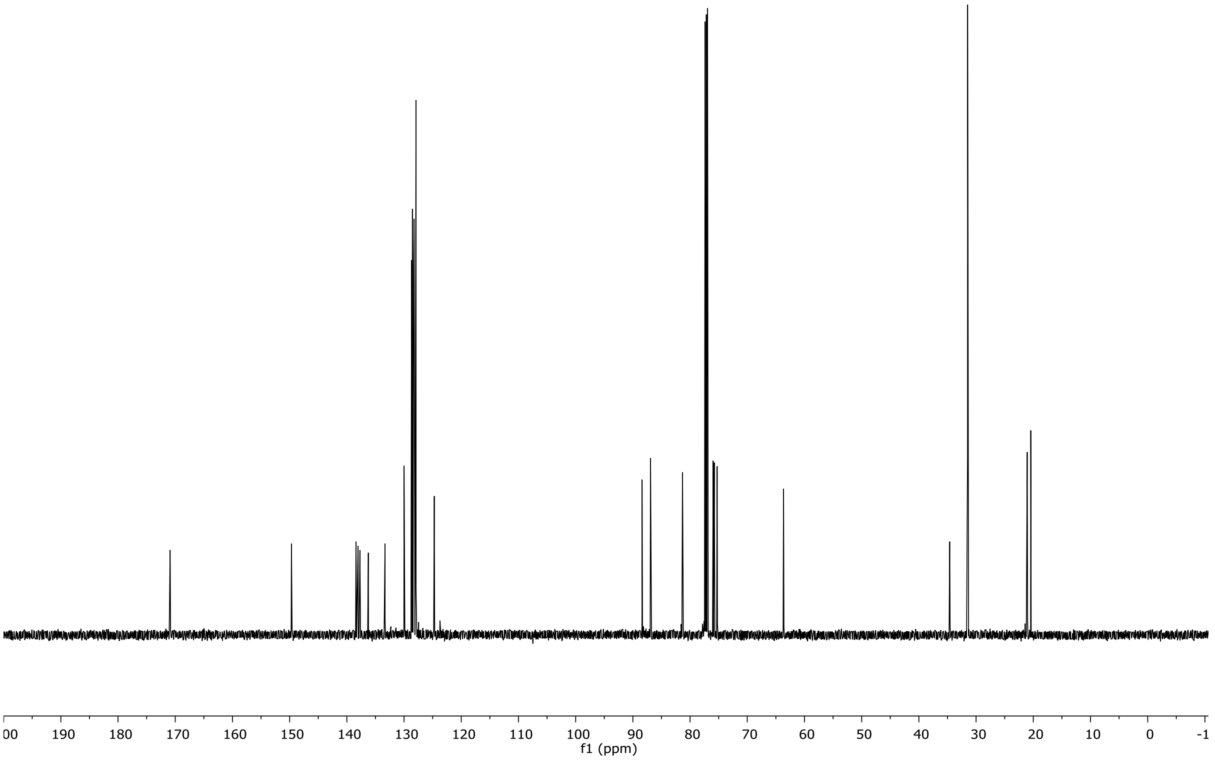

Supplementary Figure 67 | 1D NMR spectra of 29b

^{1}H-COSY NMR, 400 MHz, $CDCl_3$

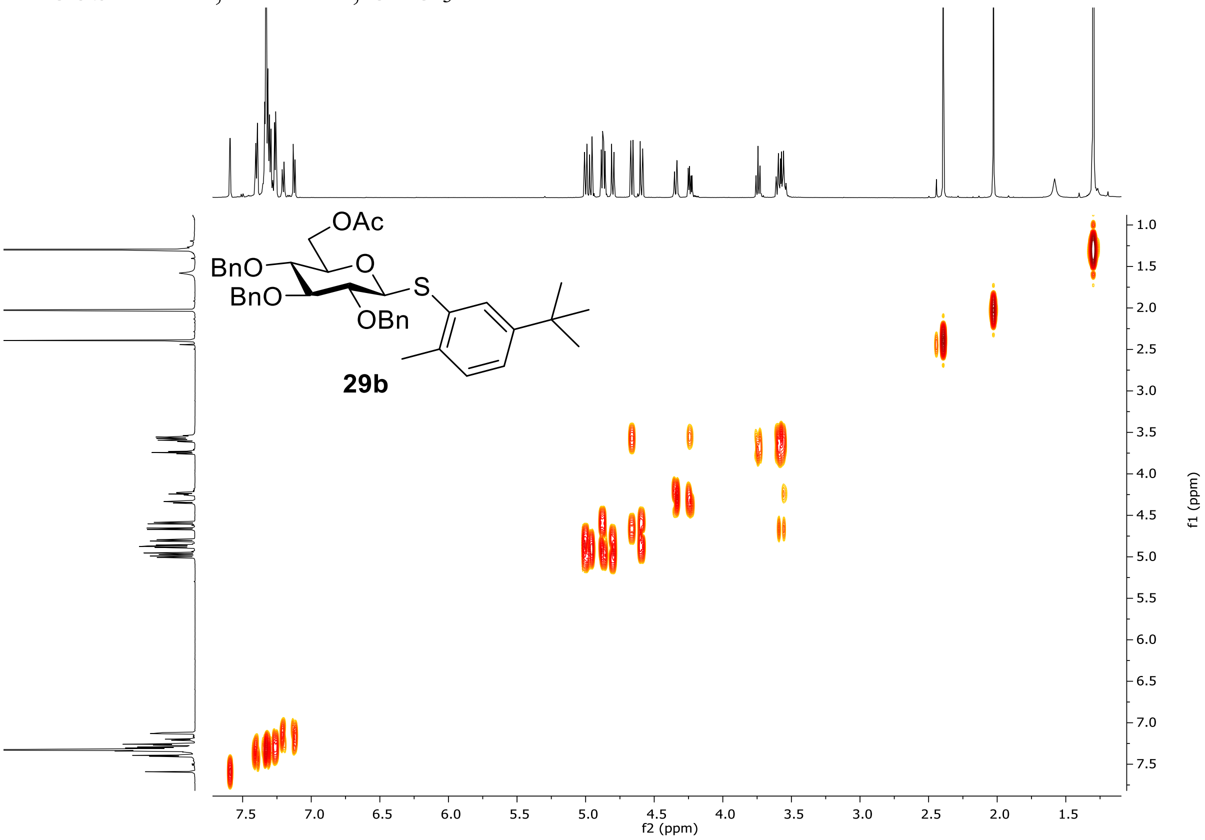

^{1}H-^{13}C-HSQC NMR, 400 MHz, $CDCl_3$

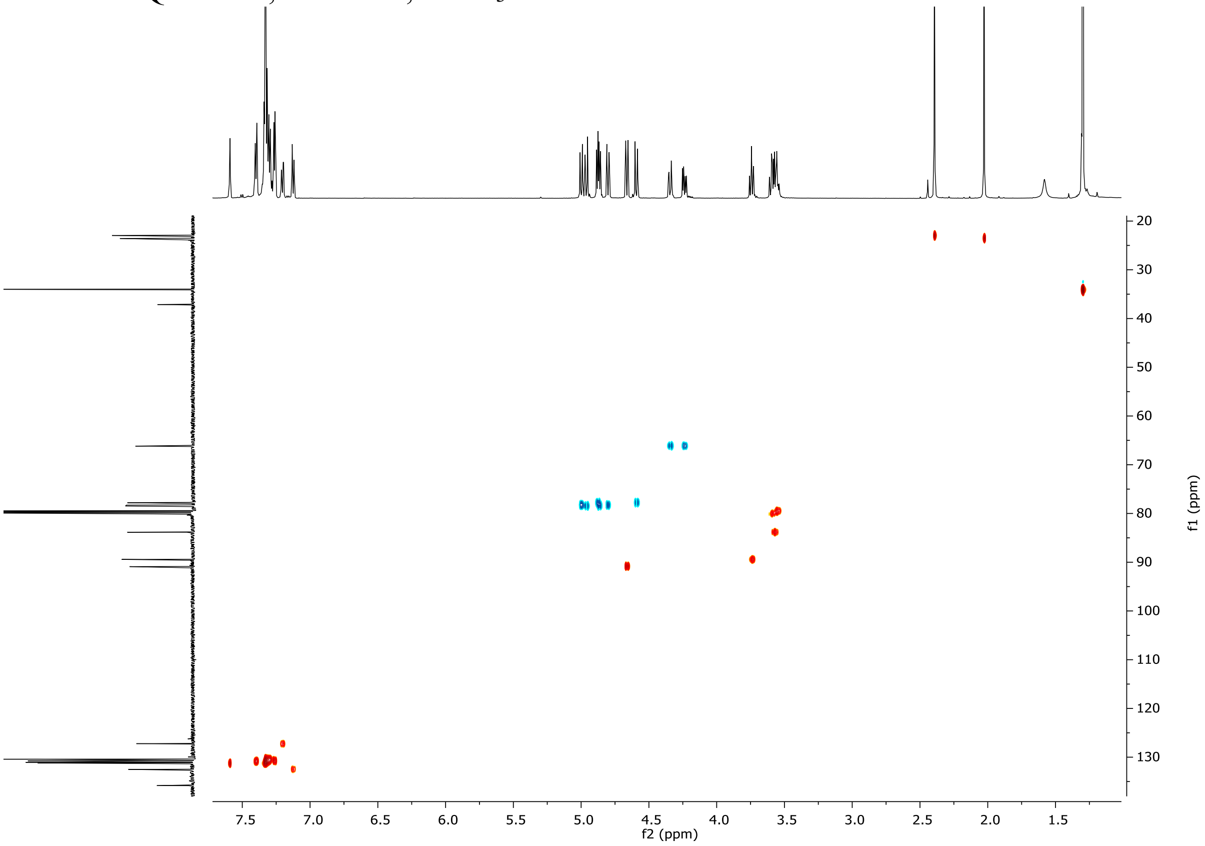

Supplementary Figure 68 | 2D NMR spectra of 29b

^{1}H NMR, 400 MHz, $CDCl_3$

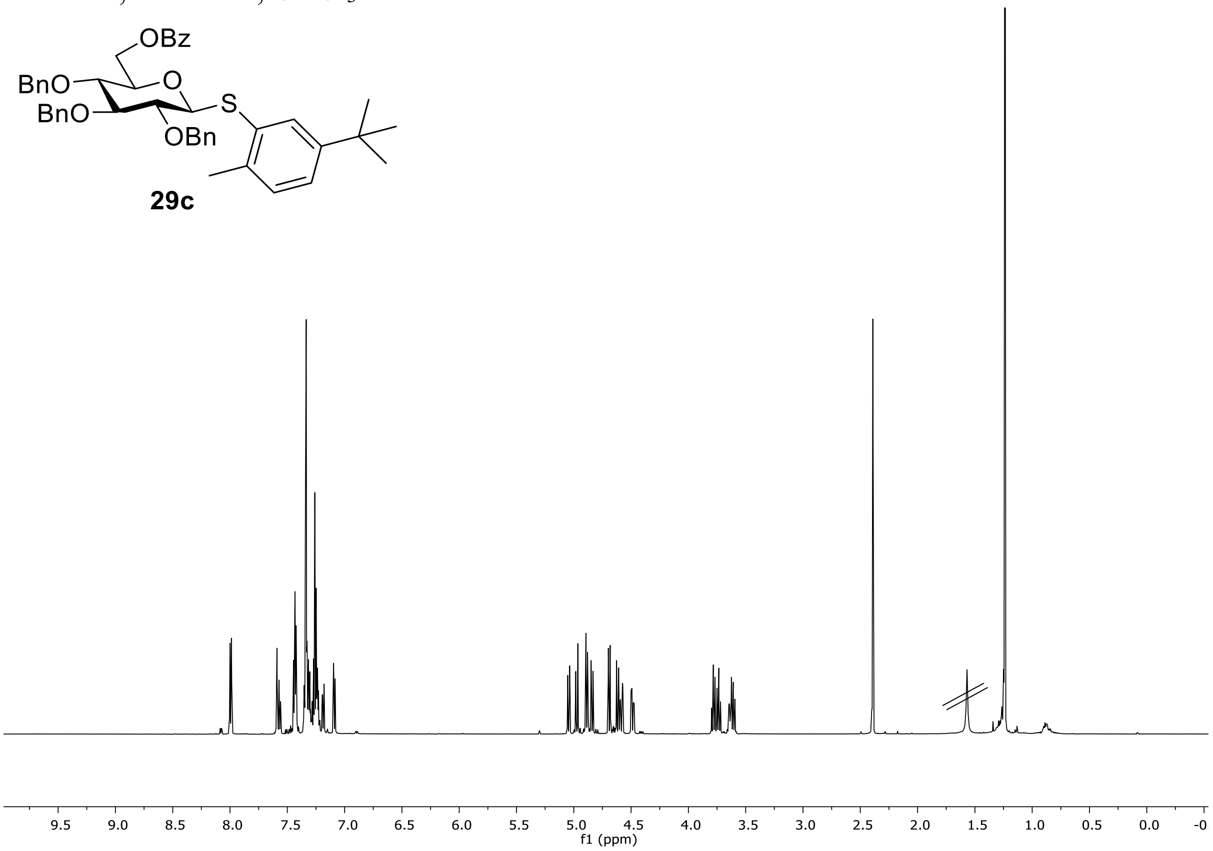

^{13}C NMR, 100 MHz, $CDCl_3$

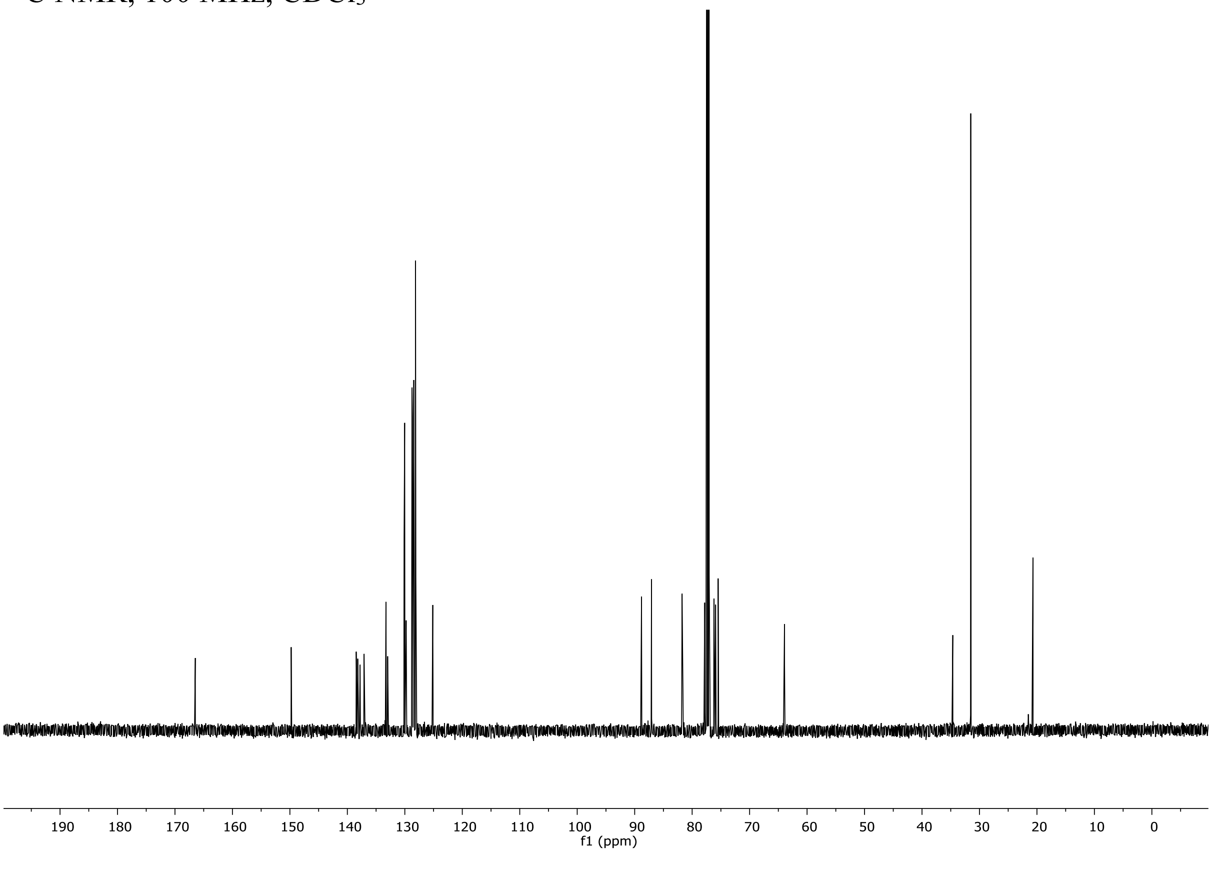

Supplementary Figure 69 | 1D NMR spectra of 29c

^{1}H-COSY NMR, 400 MHz, $CDCl_3$

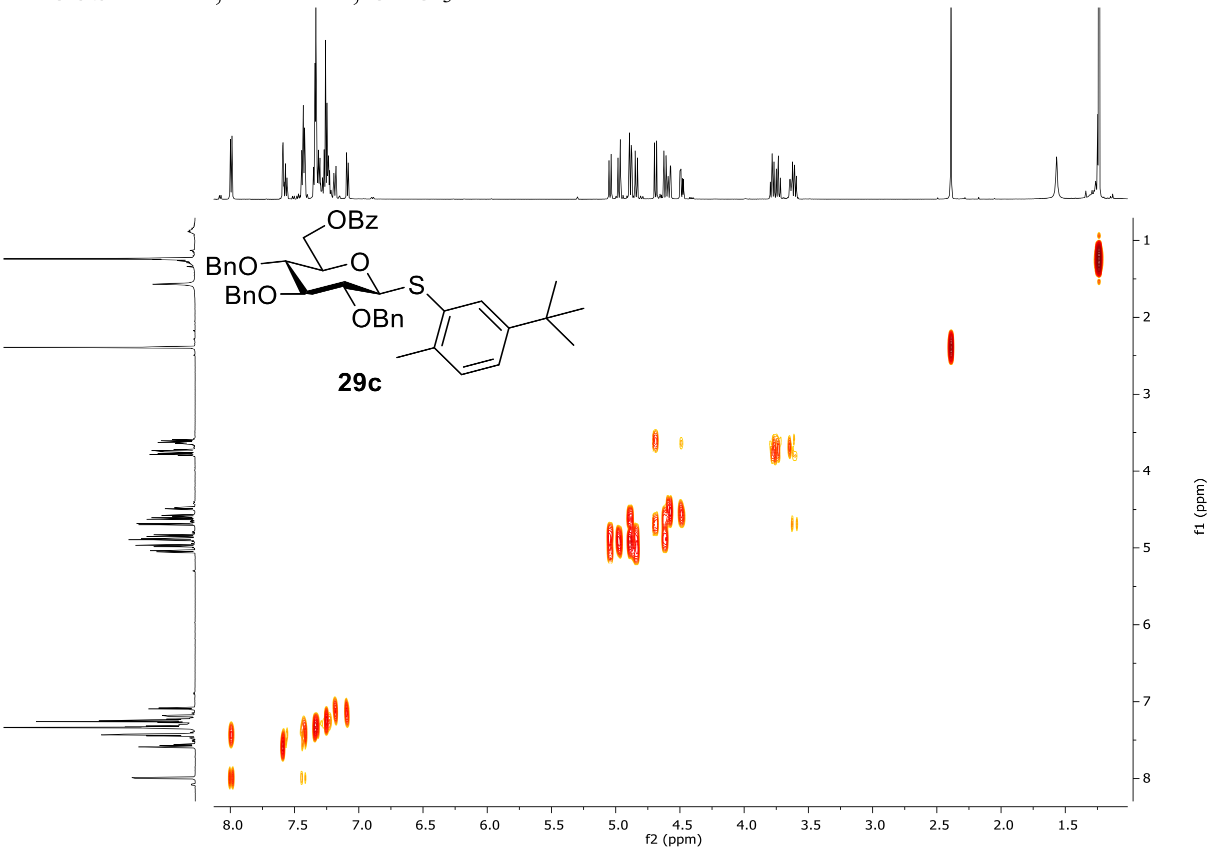

^{1}H-^{13}C-HSQC NMR, 400 MHz, $CDCl_3$

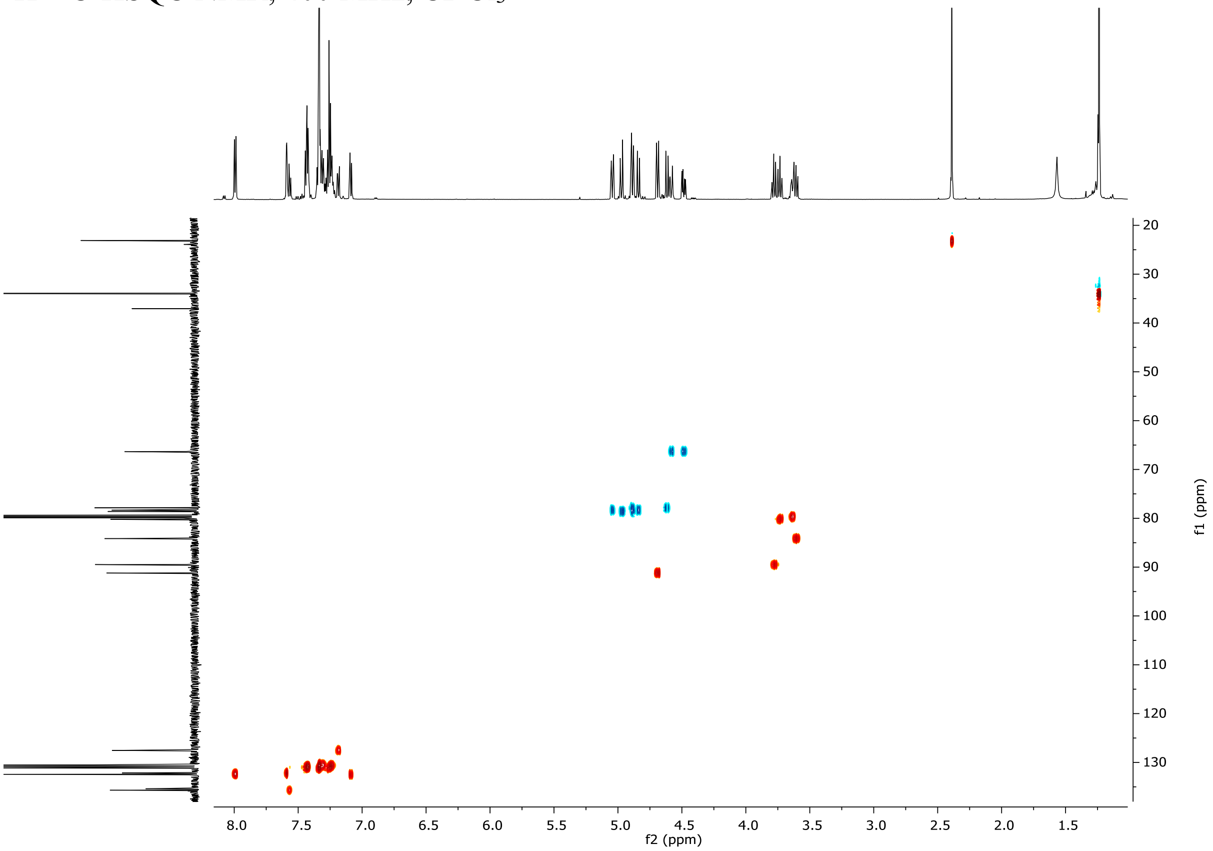

Supplementary Figure 70 | 2D NMR spectra of 29c

^{1}H NMR, 400 MHz, $CDCl_3$

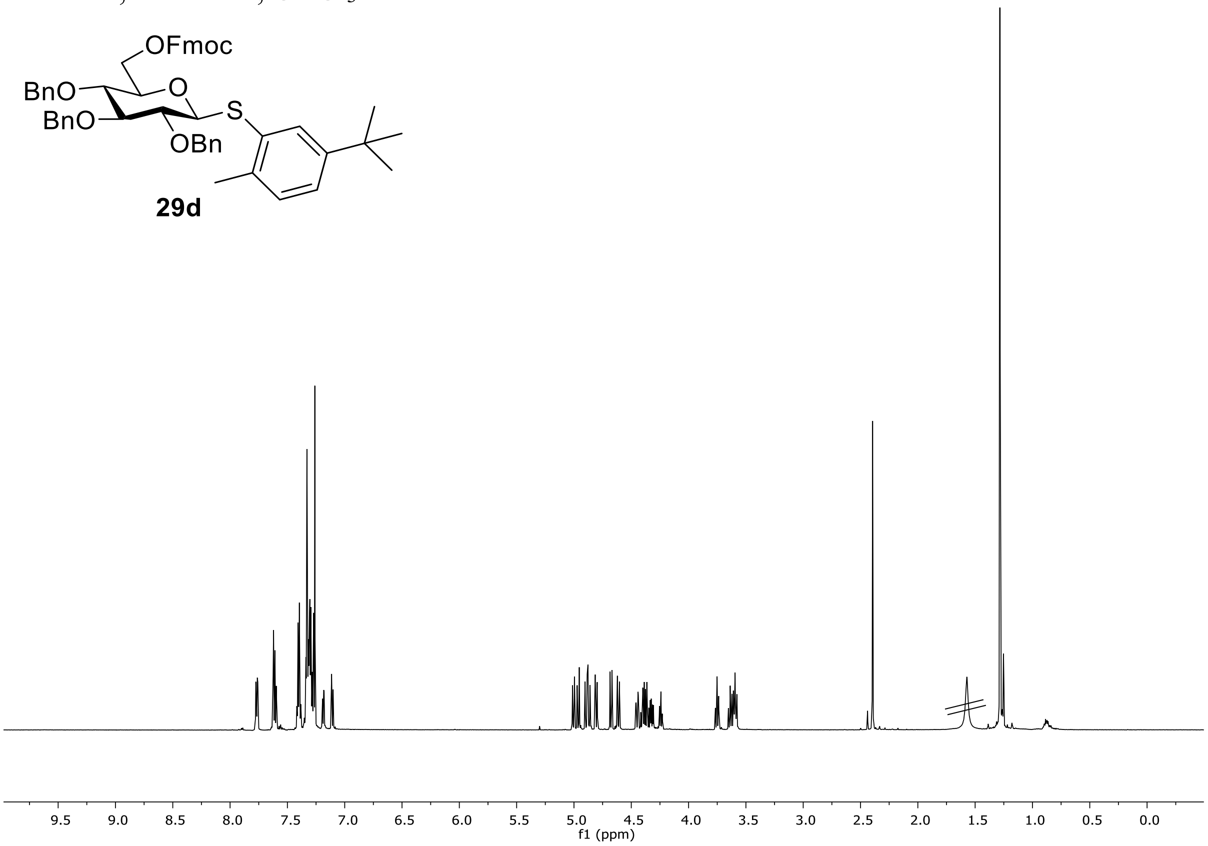

^{13}C NMR, 100 MHz, $CDCl_3$

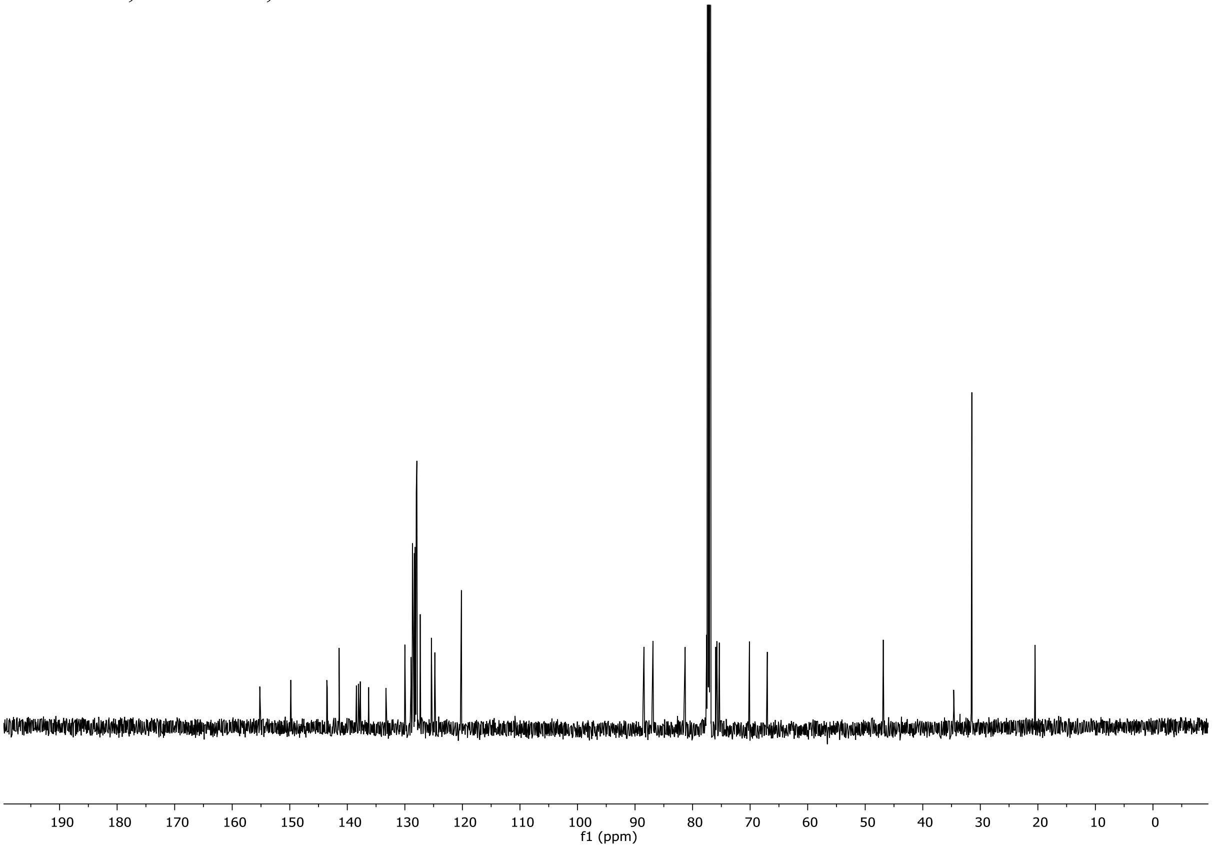

Supplementary Figure 71 | 1D NMR spectra of 29d

^{1}H-COSY NMR, 400 MHz, $CDCl_3$

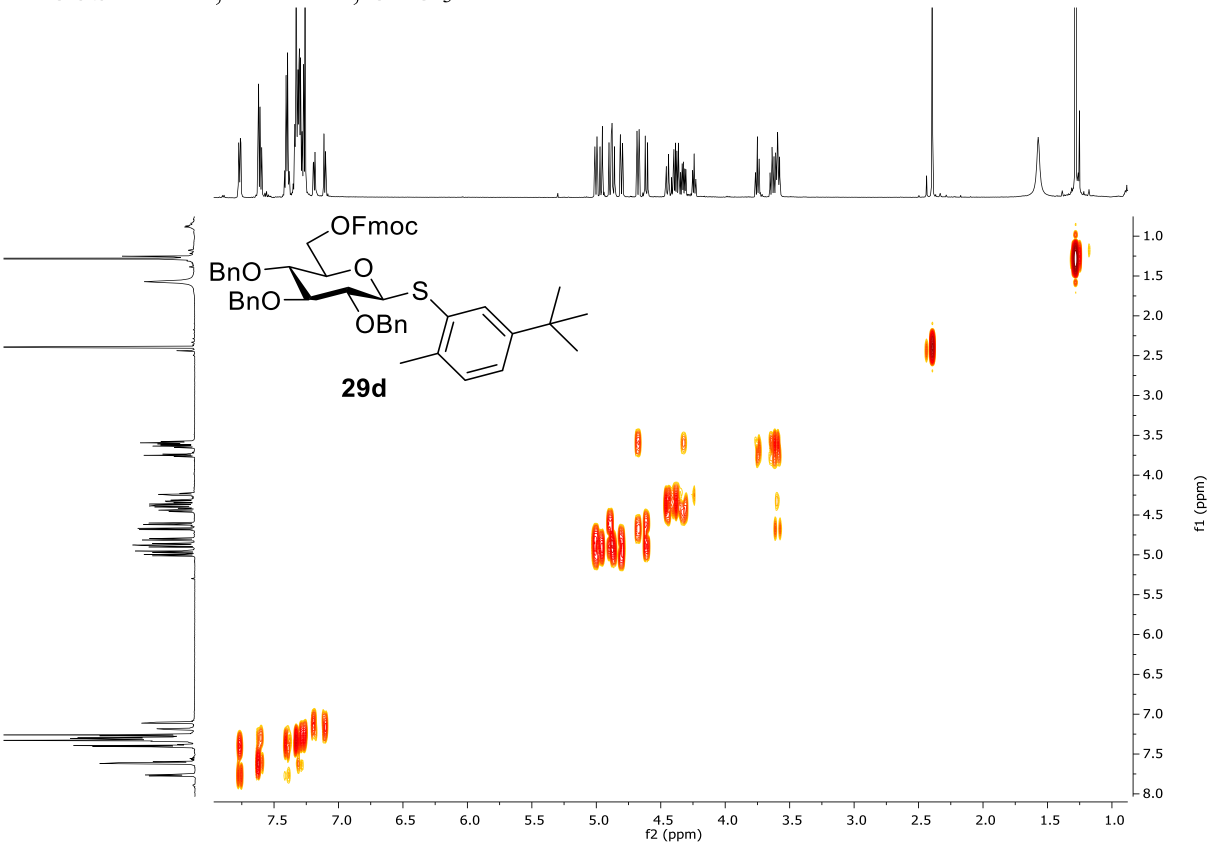

^{1}H-^{13}C-HSQC NMR, 400 MHz, $CDCl_3$

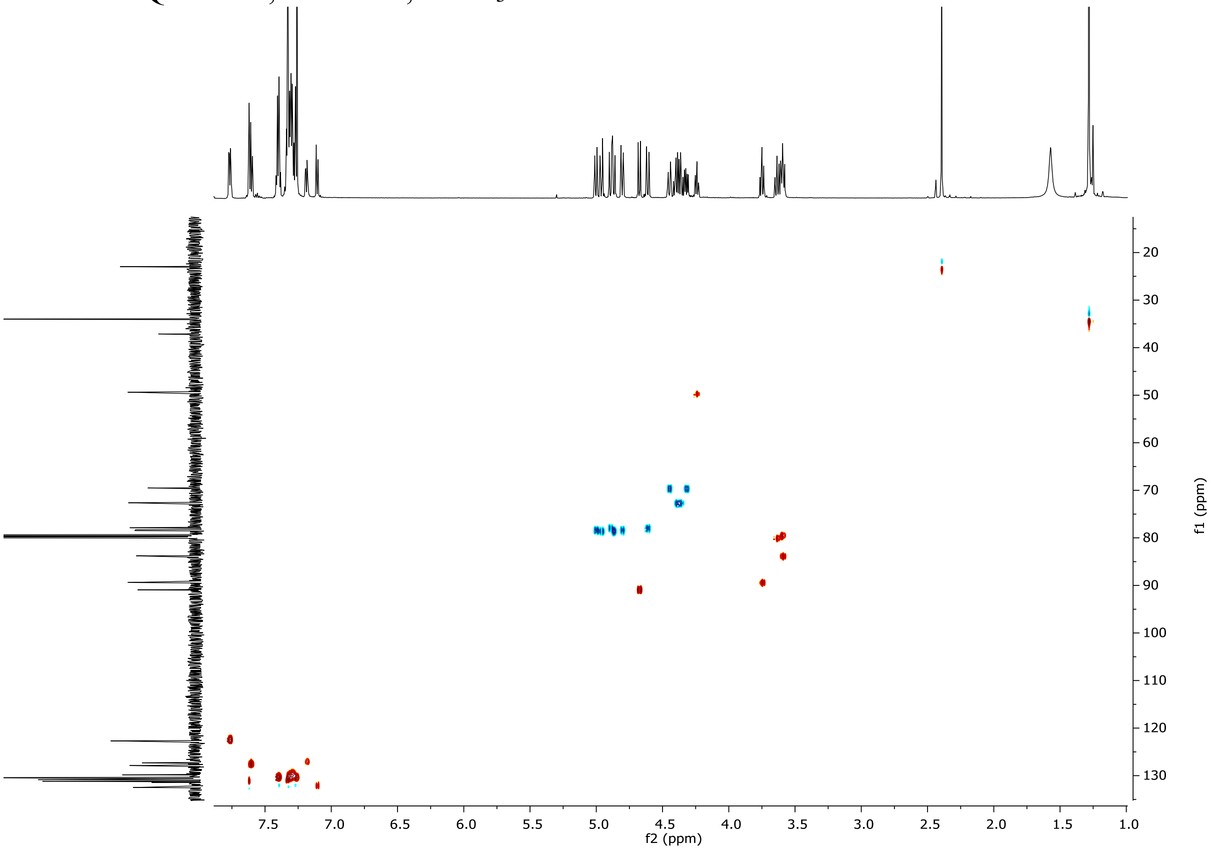

Supplementary Figure 72 | 2D NMR spectra of 29d

^{1}H NMR, 400 MHz, $CDCl_3$

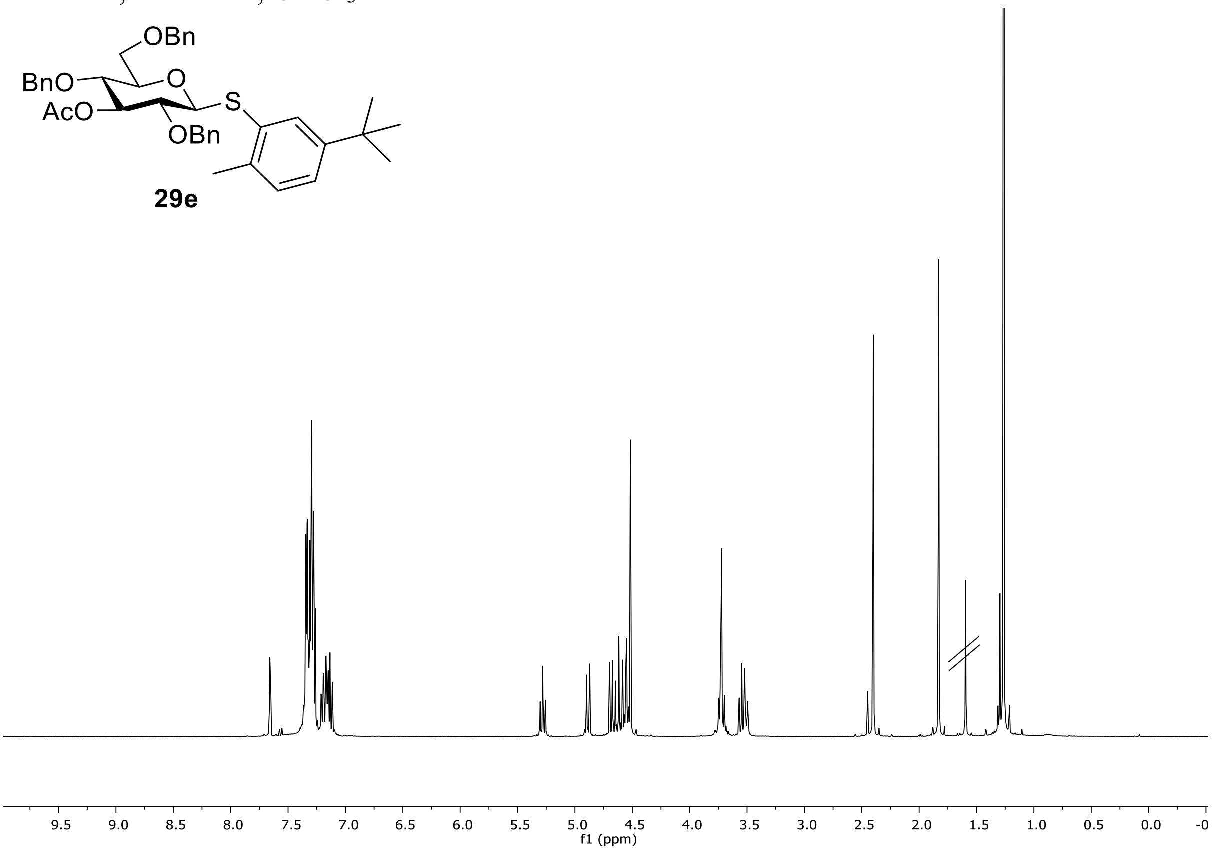

^{13}C NMR, 100 MHz, $CDCl_3$

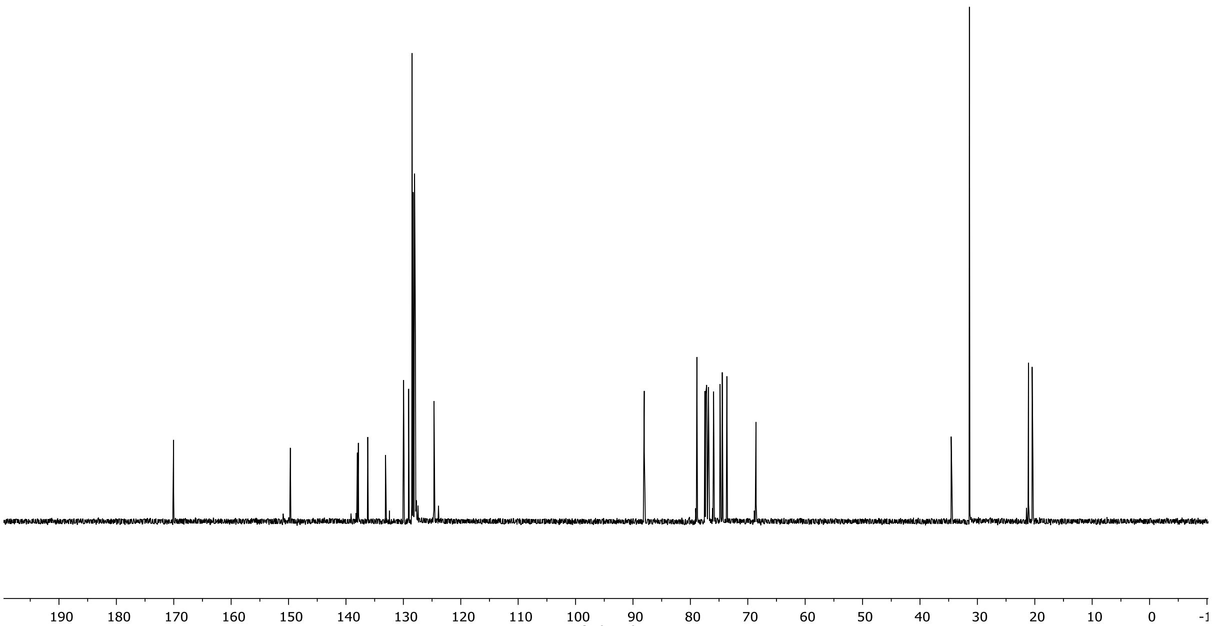

Supplementary Figure 73 | 1D NMR spectra of 29e

^{1}H-COSY NMR, 400 MHz, $CDCl_3$

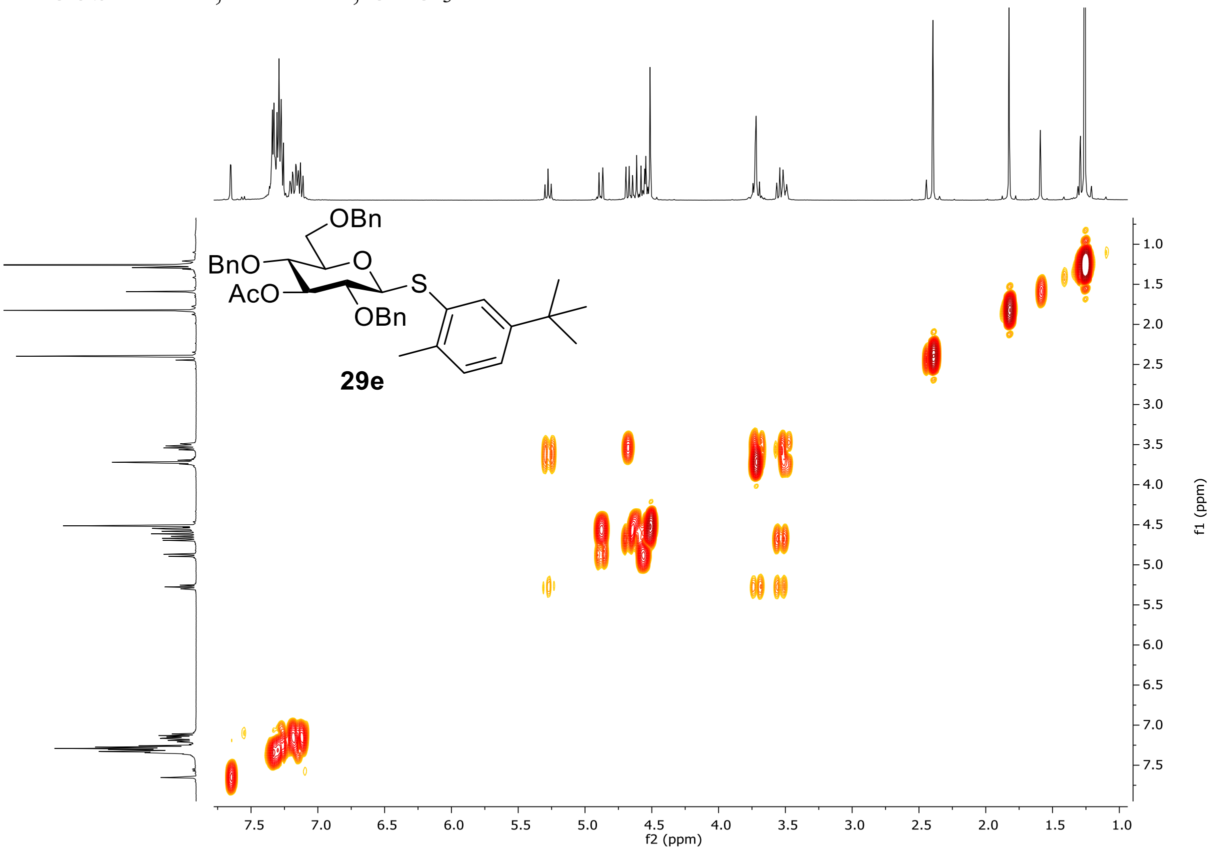

^{1}H-^{13}C-HSQC NMR, 400 MHz, $CDCl_3$

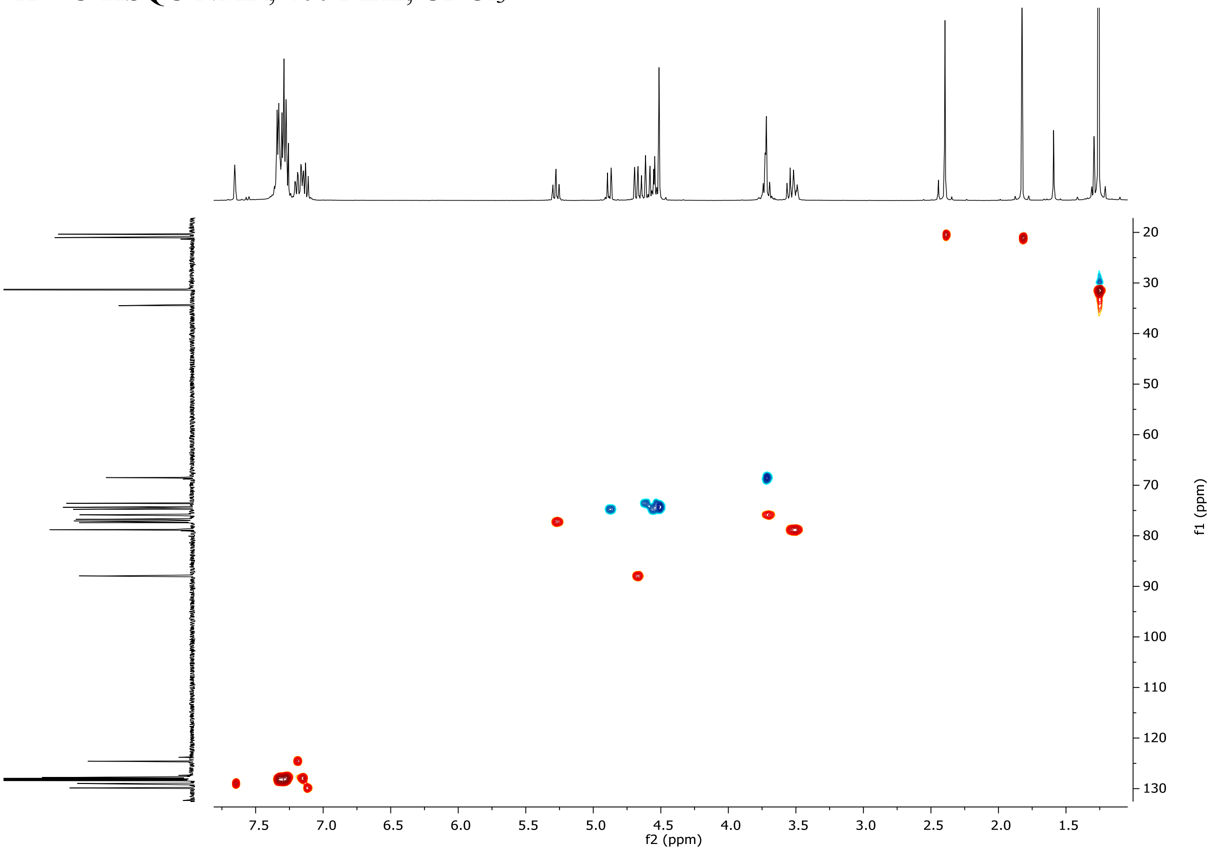

Supplementary Figure 74 | 2D NMR spectra of 29e

^{1}H NMR, 400 MHz, $CDCl_3$

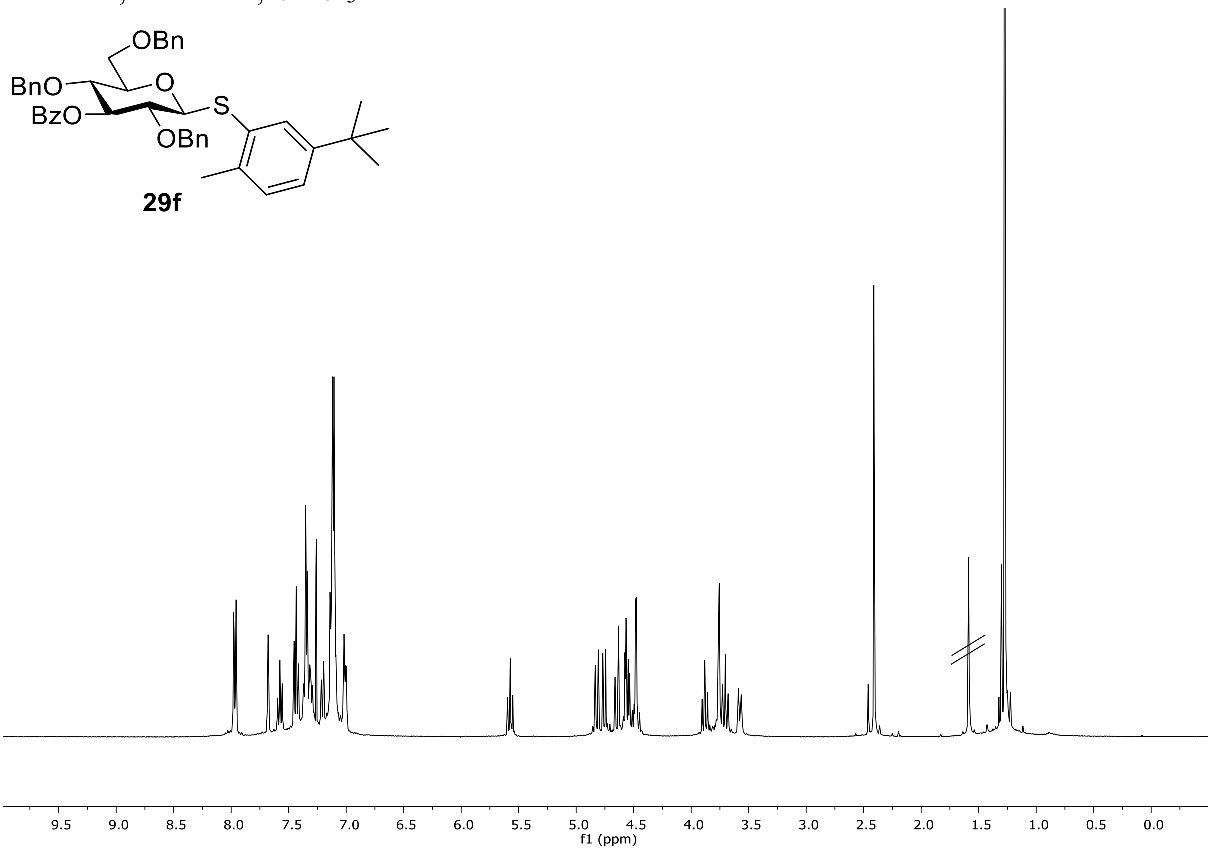

^{13}C NMR, 100 MHz, $CDCl_3$

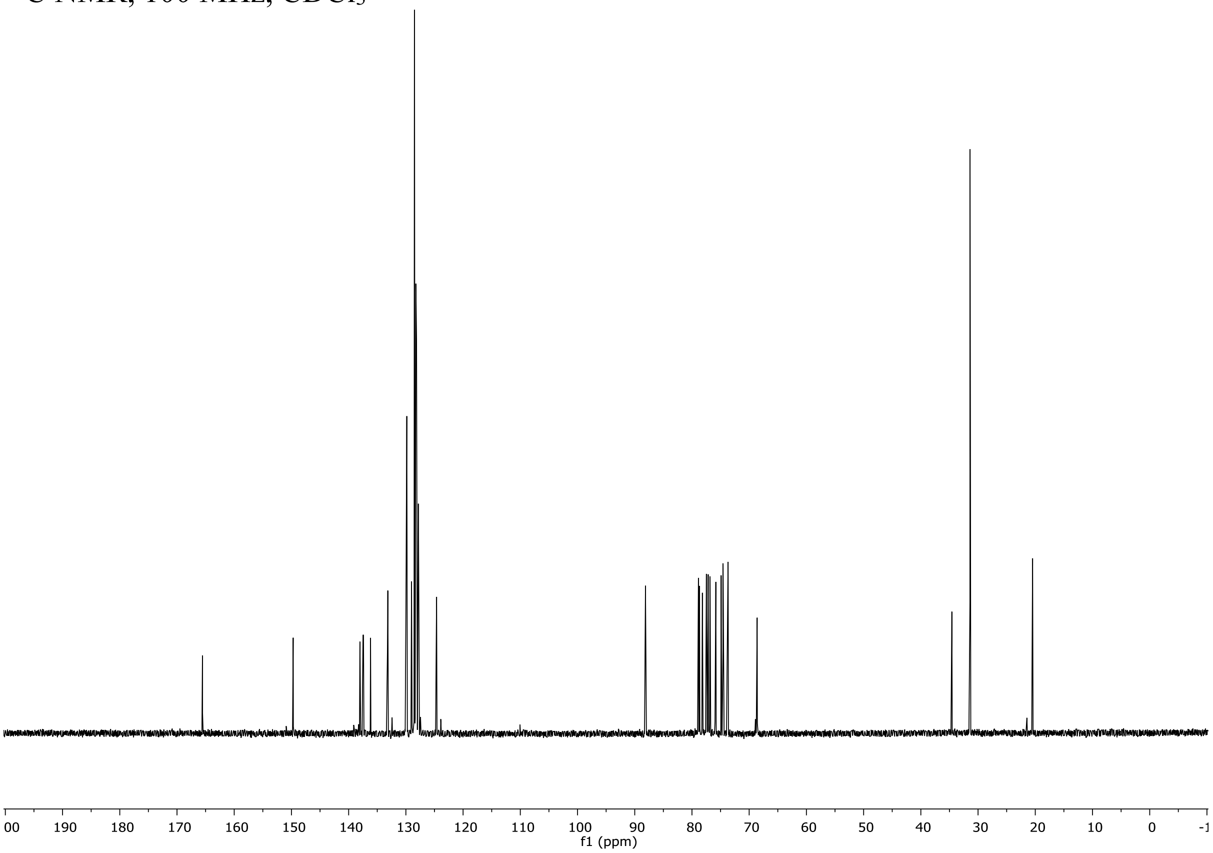

Supplementary Figure 75 | 1D NMR spectra of 29f

^{1}H-COSY NMR, 400 MHz, $CDCl_3$

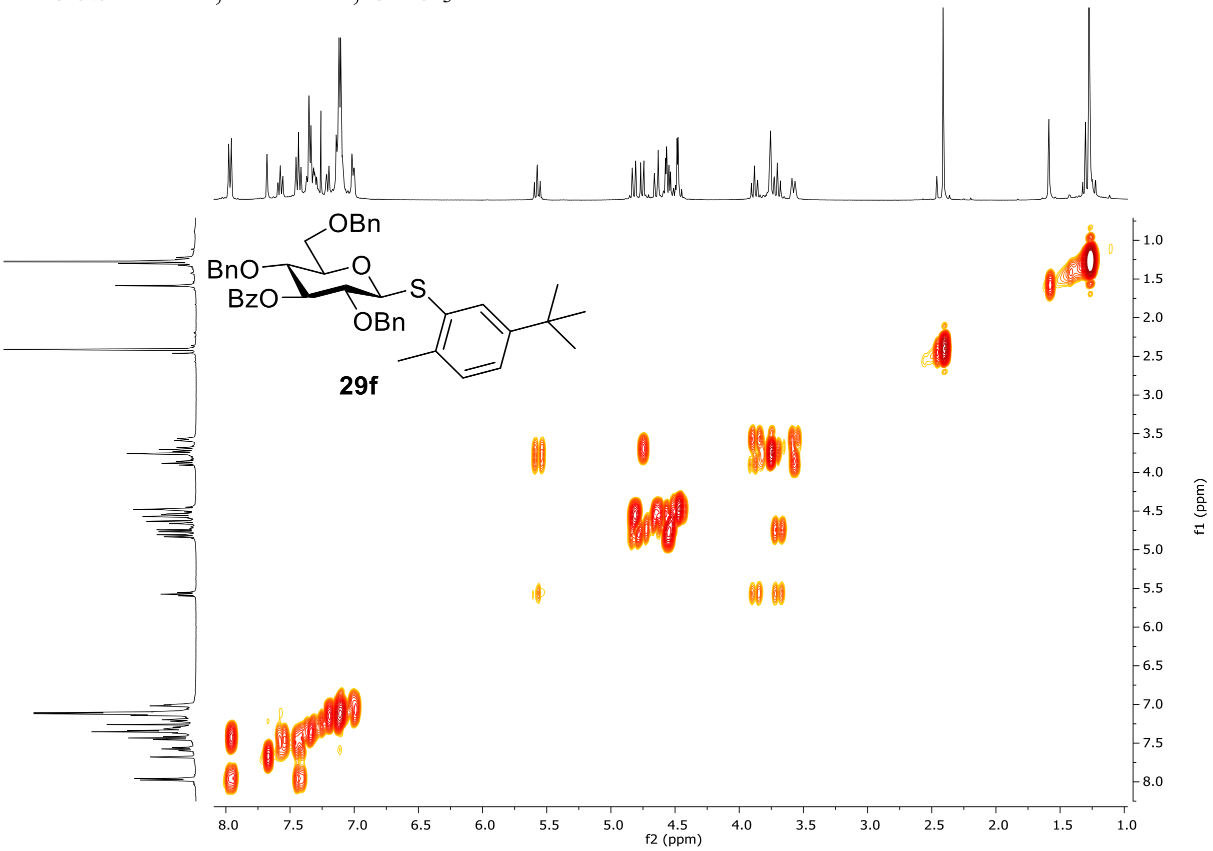

^{1}H-^{13}C-HSQC NMR, 400 MHz, $CDCl_3$

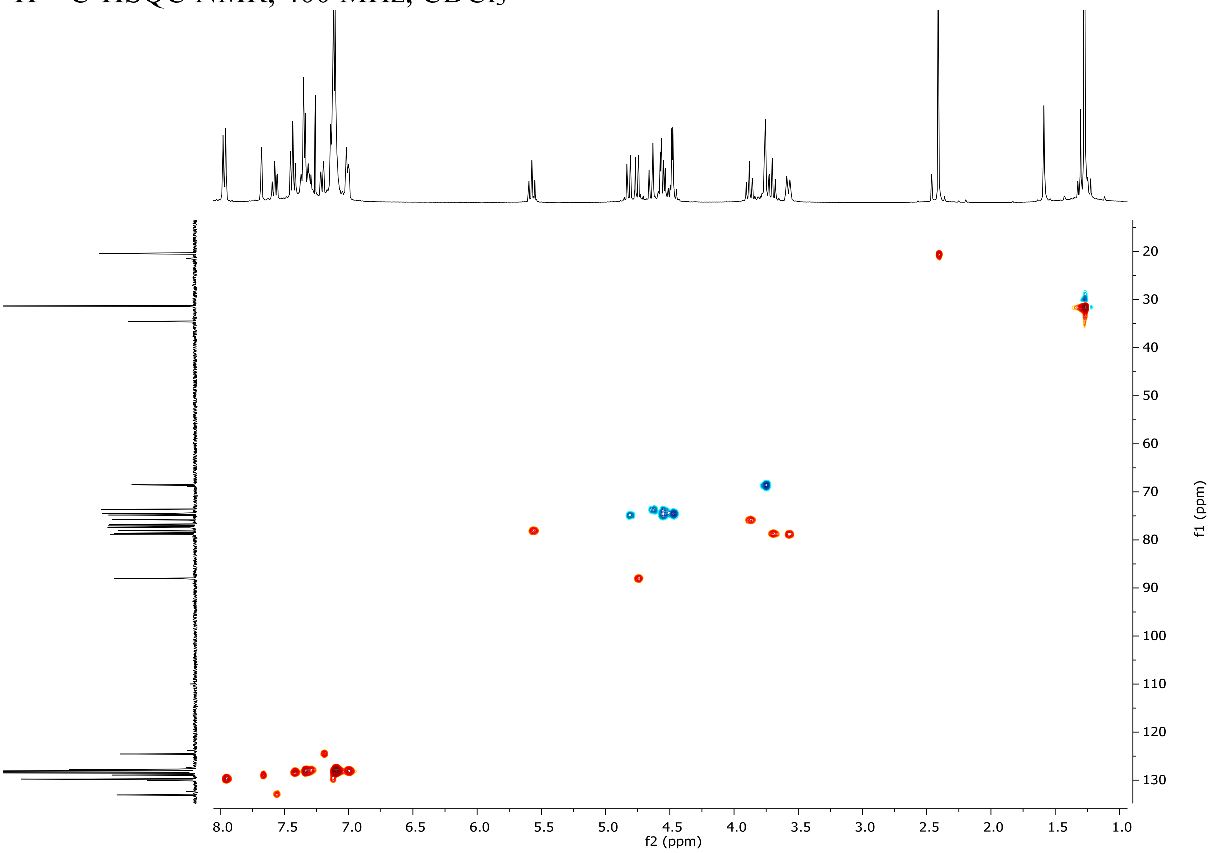

Supplementary Figure 76 | 2D NMR spectra of 29f

^{1}H NMR, 400 MHz, $CDCl_3$

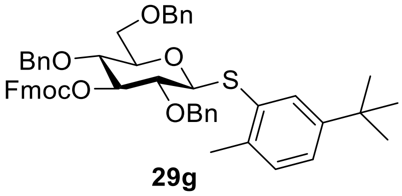

29g

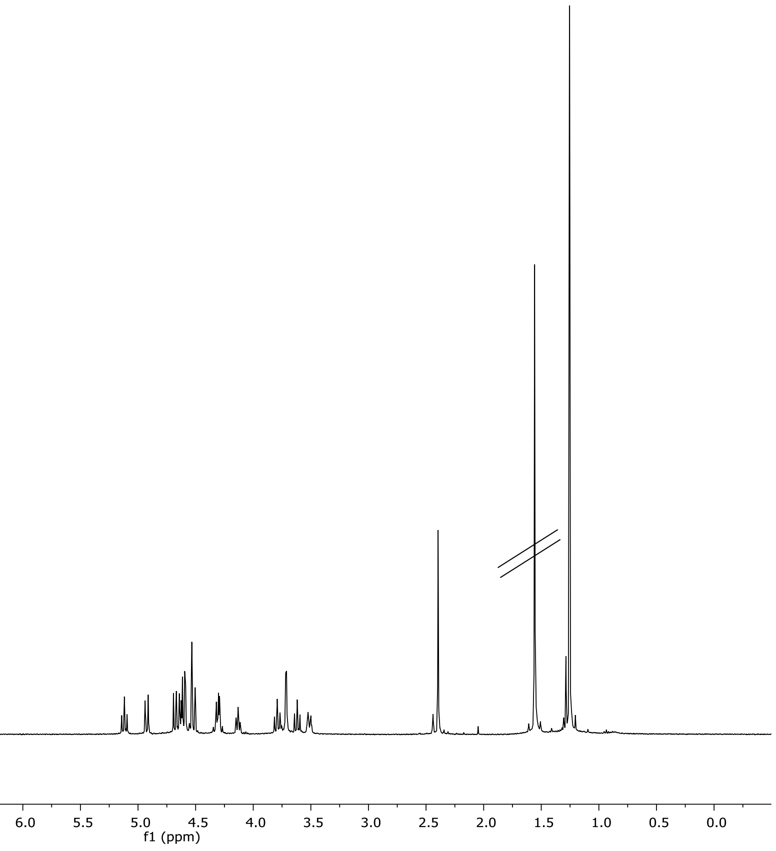

^{13}C NMR, 100 MHz, $CDCl_3$

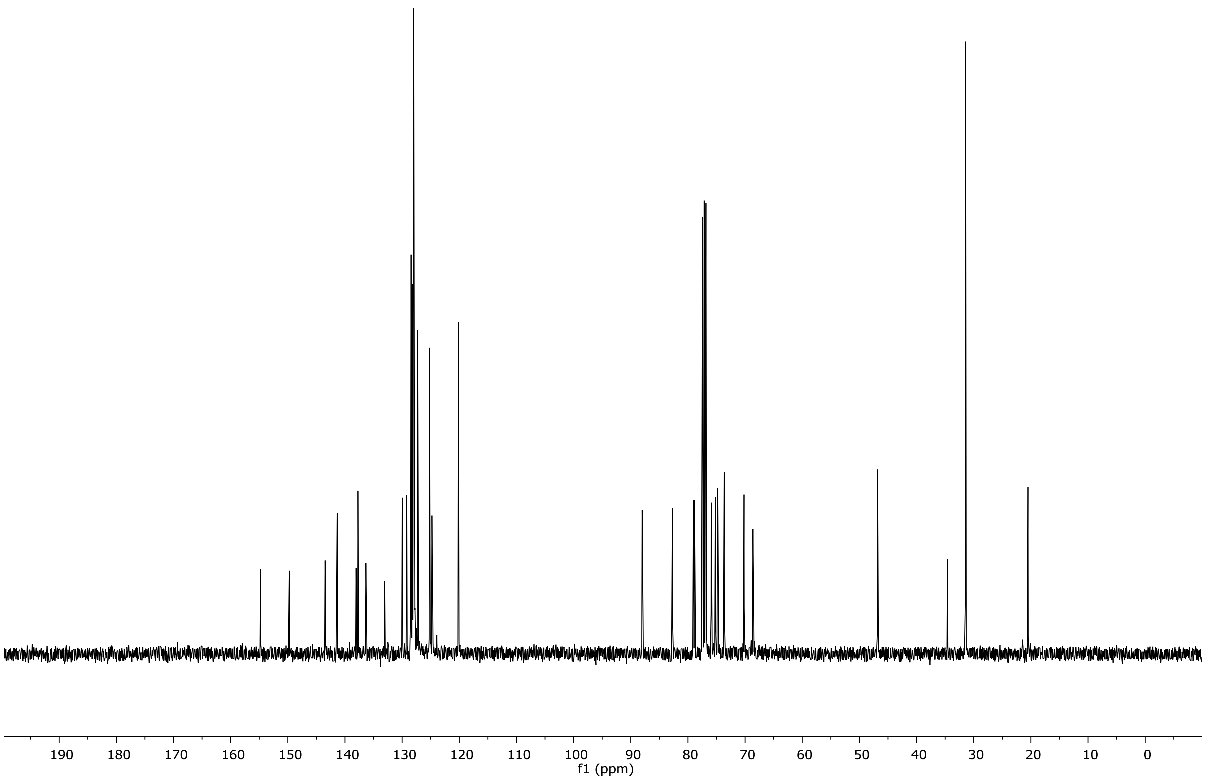

Supplementary Figure 77 | 1D NMR spectra of 29g

^{1}H-COSY NMR, 400 MHz, $CDCl_3$

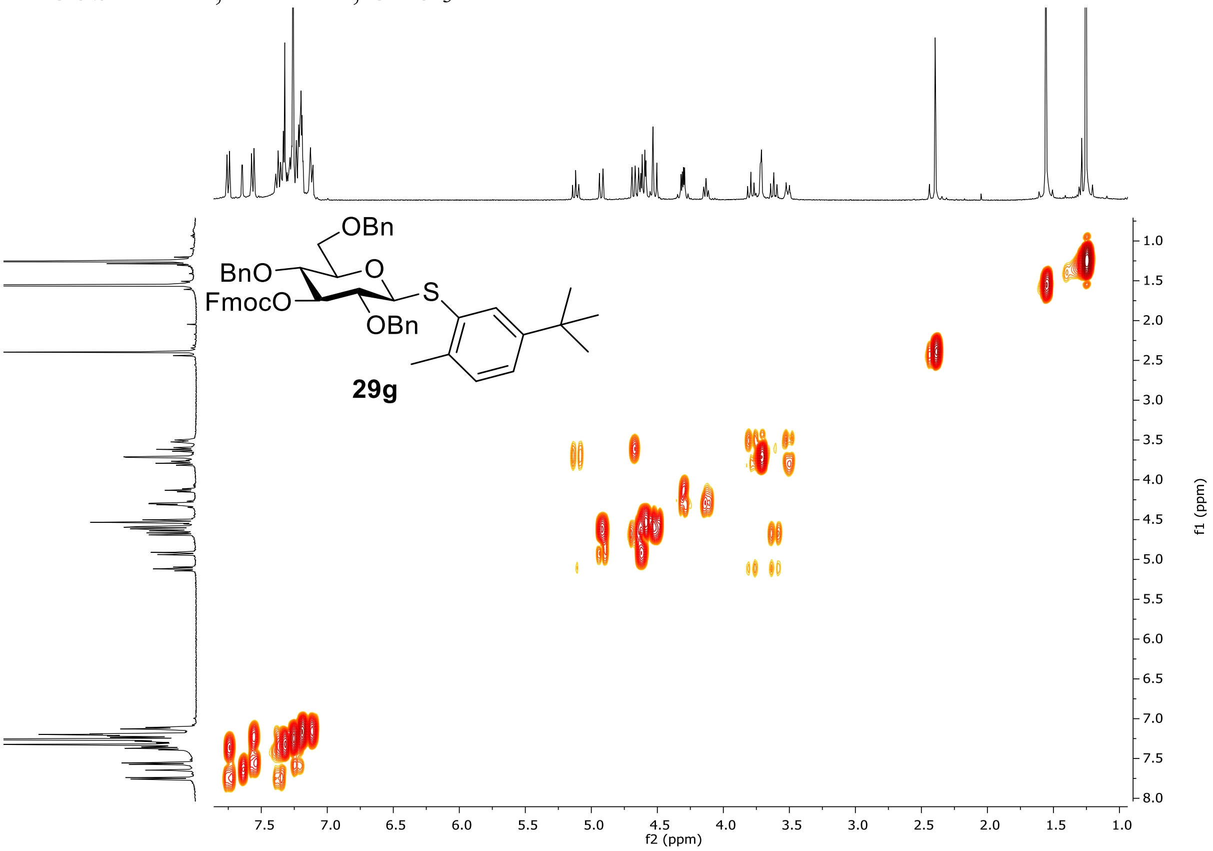

^{1}H-^{13}C-HSQC NMR, 400 MHz, $CDCl_3$

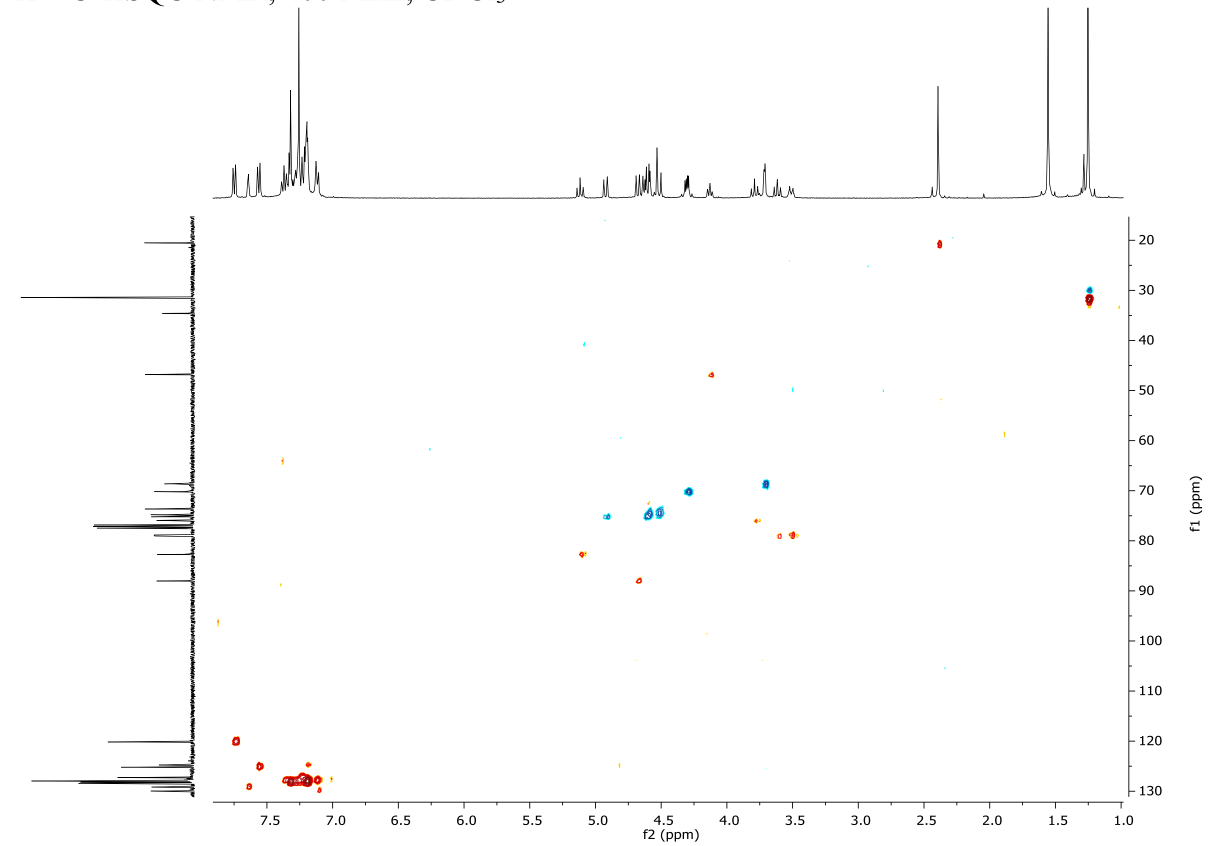

Supplementary Figure 78 | 2D NMR spectra of 29g

^{1}H NMR, 600 MHz, $CDCl_3$

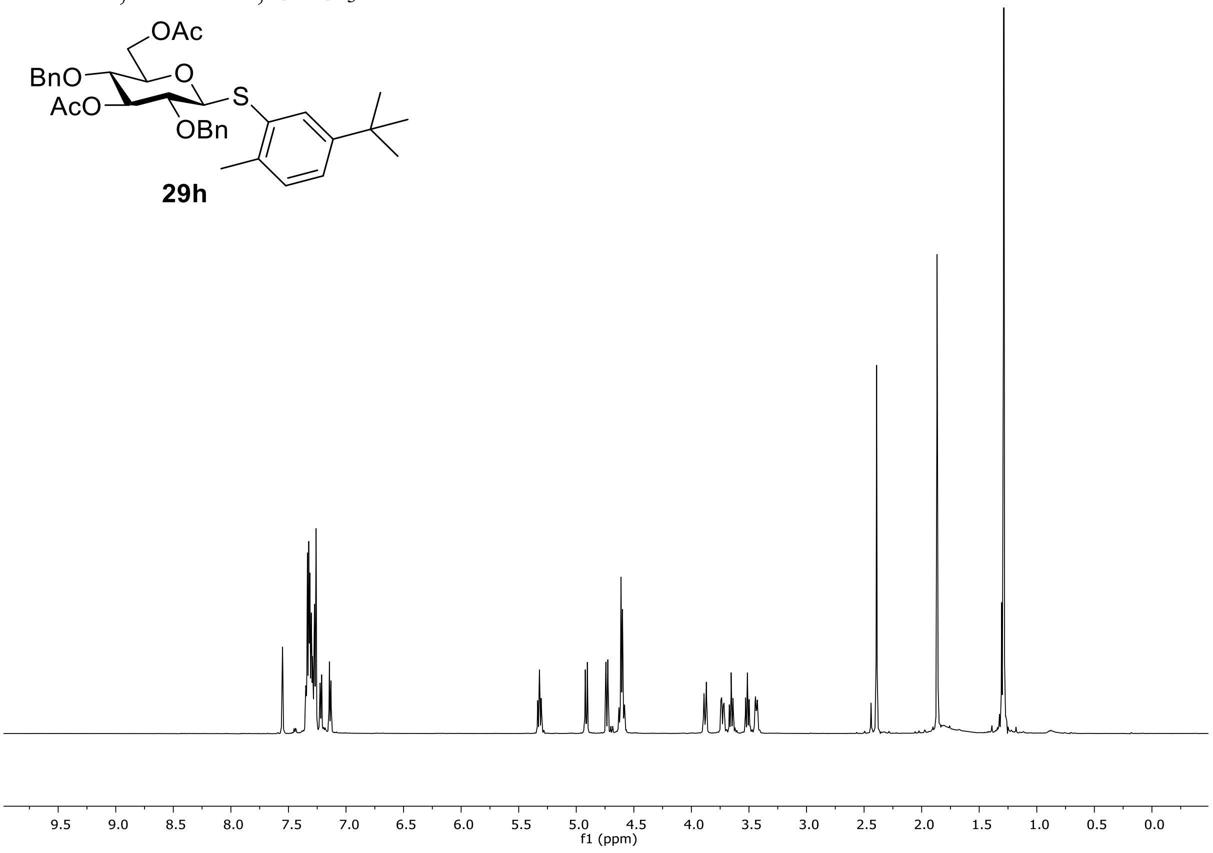

^{13}C NMR, 150 MHz, $CDCl_3$

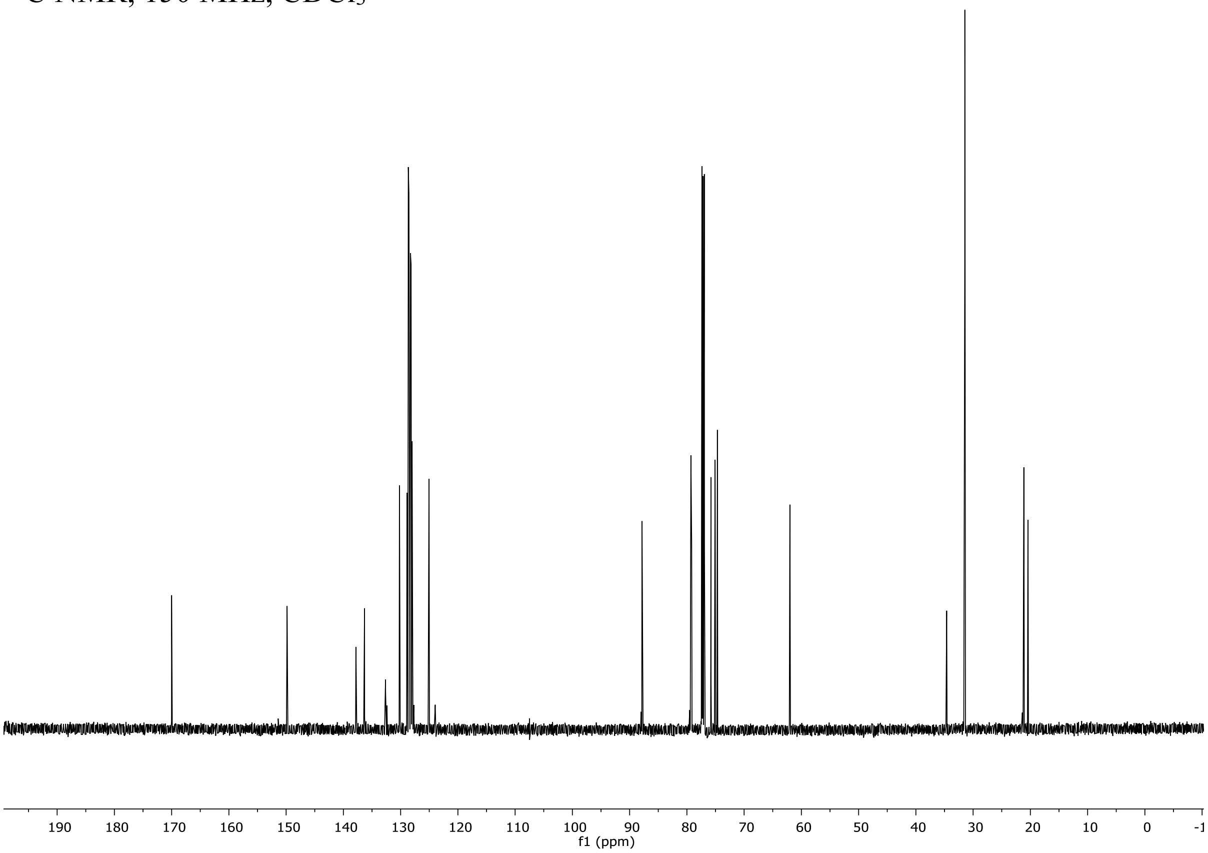

Supplementary Figure 79 | 1D NMR spectra of 29h

^{1}H-COSY NMR, 600 MHz, $CDCl_3$

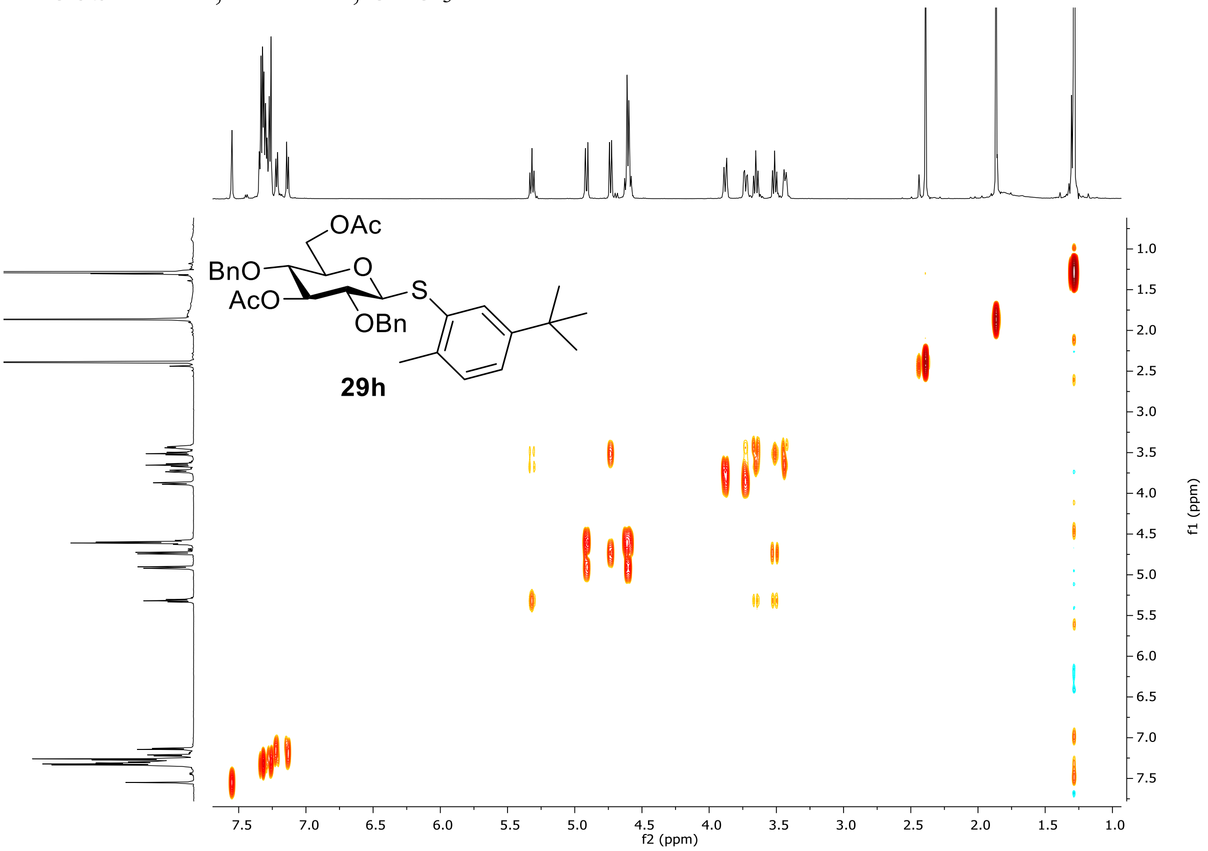

^{1}H-^{13}C-HSQC NMR, 600 MHz, $CDCl_3$

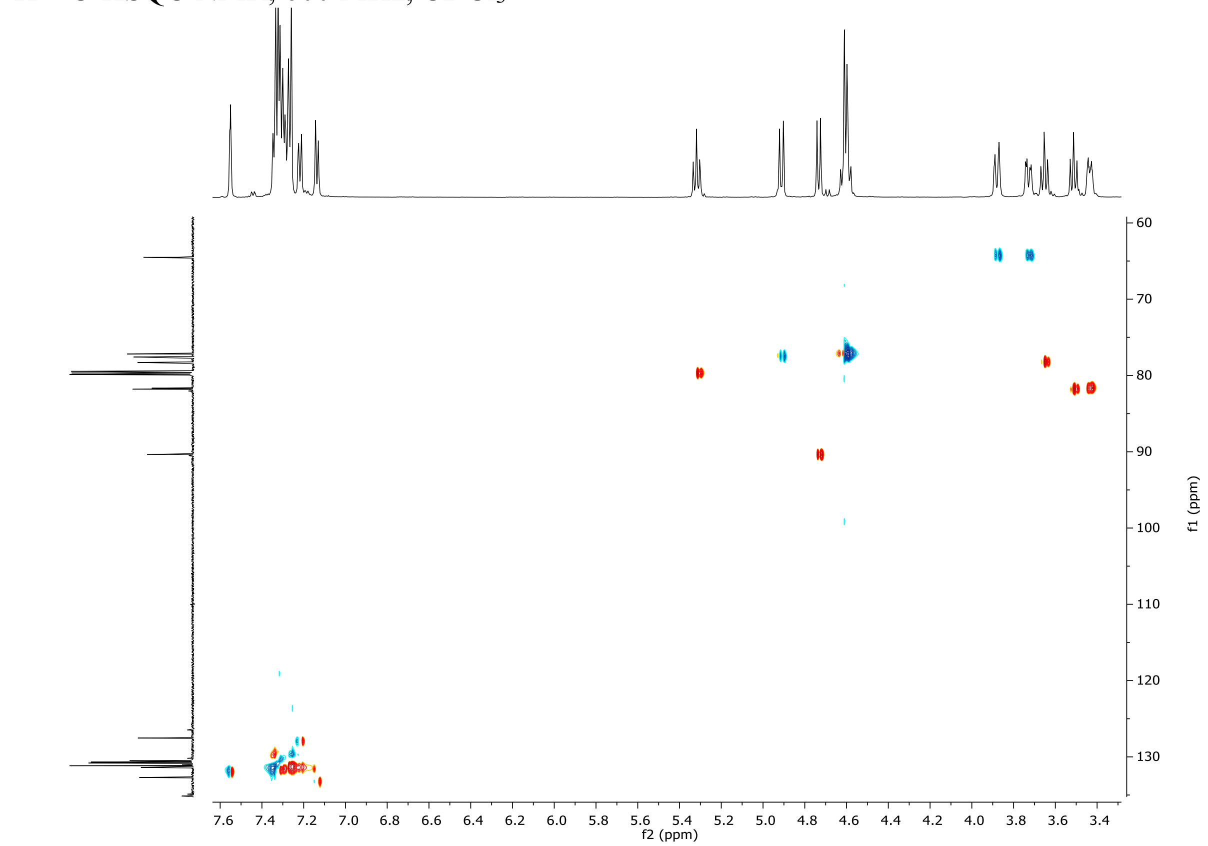

Supplementary Figure 80 | 2D NMR spectra of 29h

^{1}H NMR, 400 MHz, $CDCl_3$

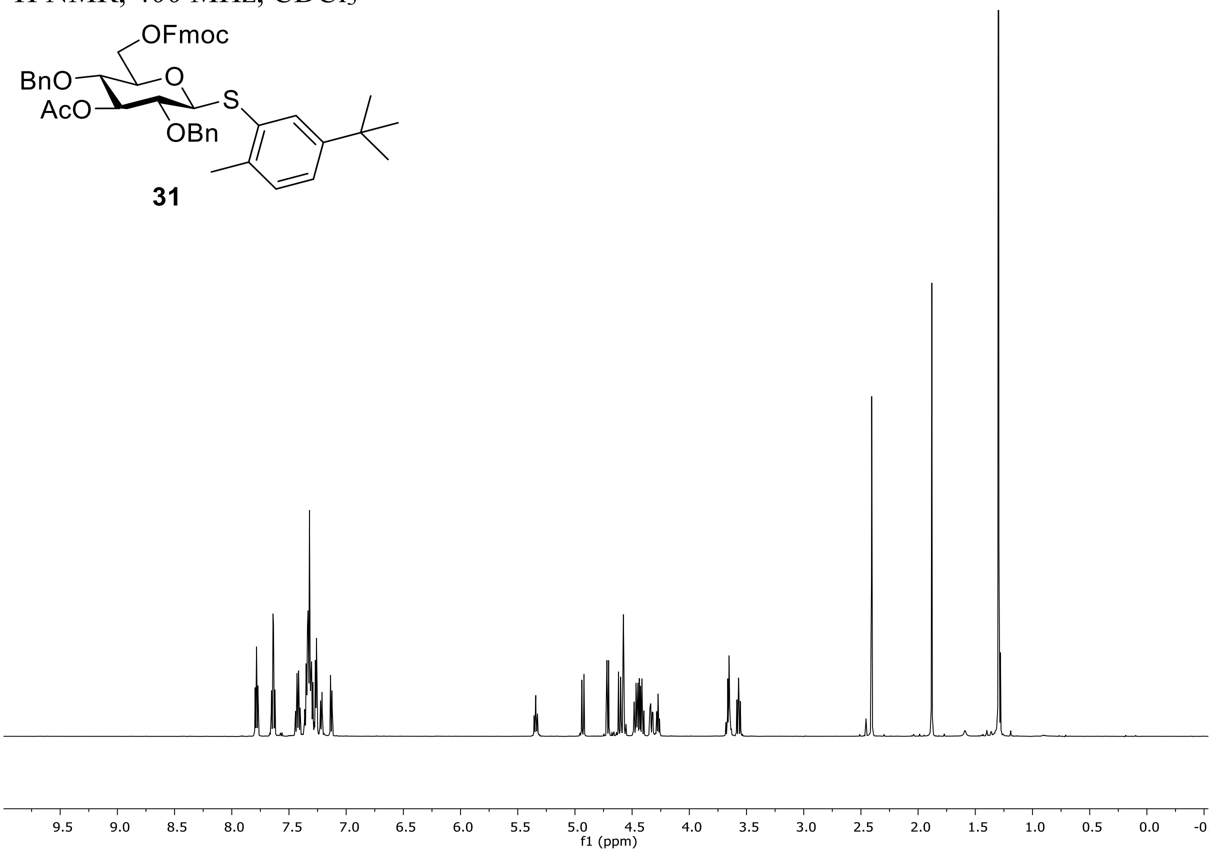

31

^{13}C NMR, 100 MHz, $CDCl_3$

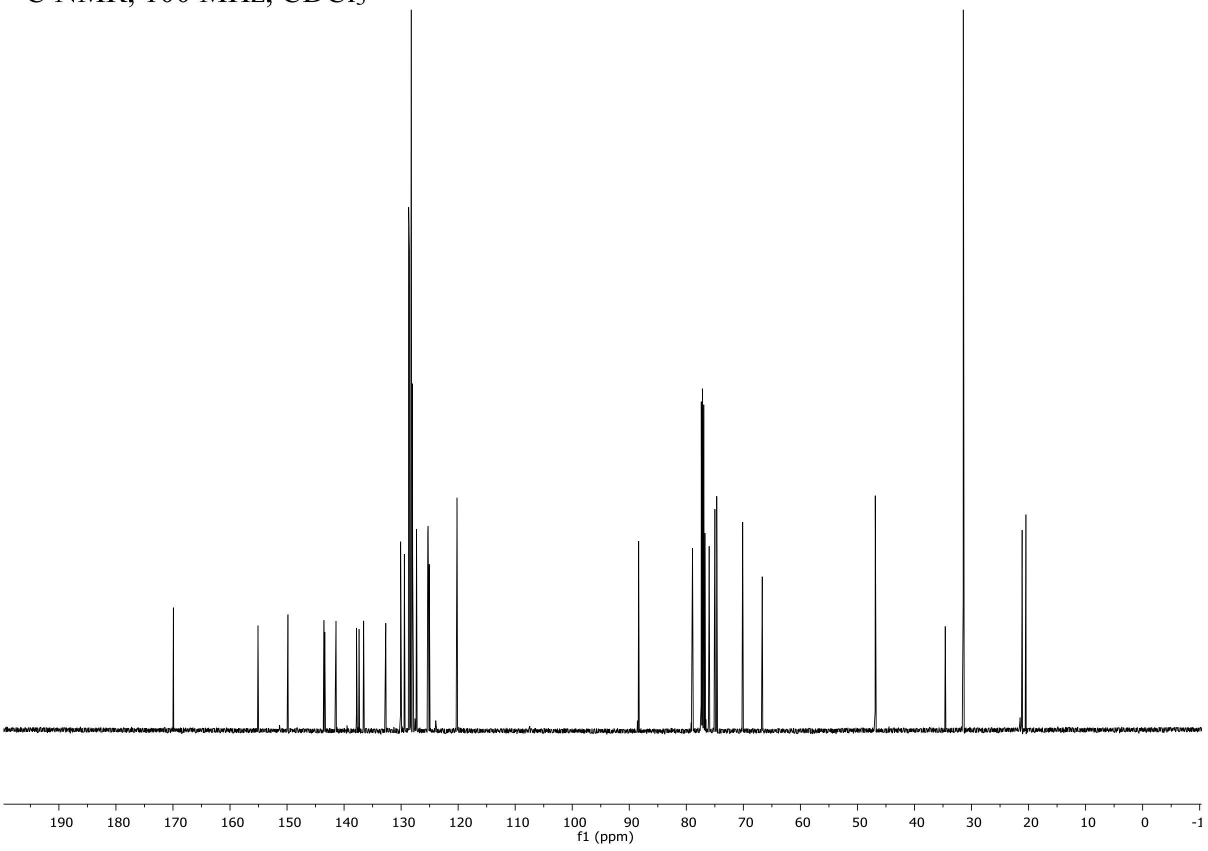

Supplementary Figure 81 | 1D NMR spectra of 31

^{1}H-COSY NMR, 400 MHz, $CDCl_3$

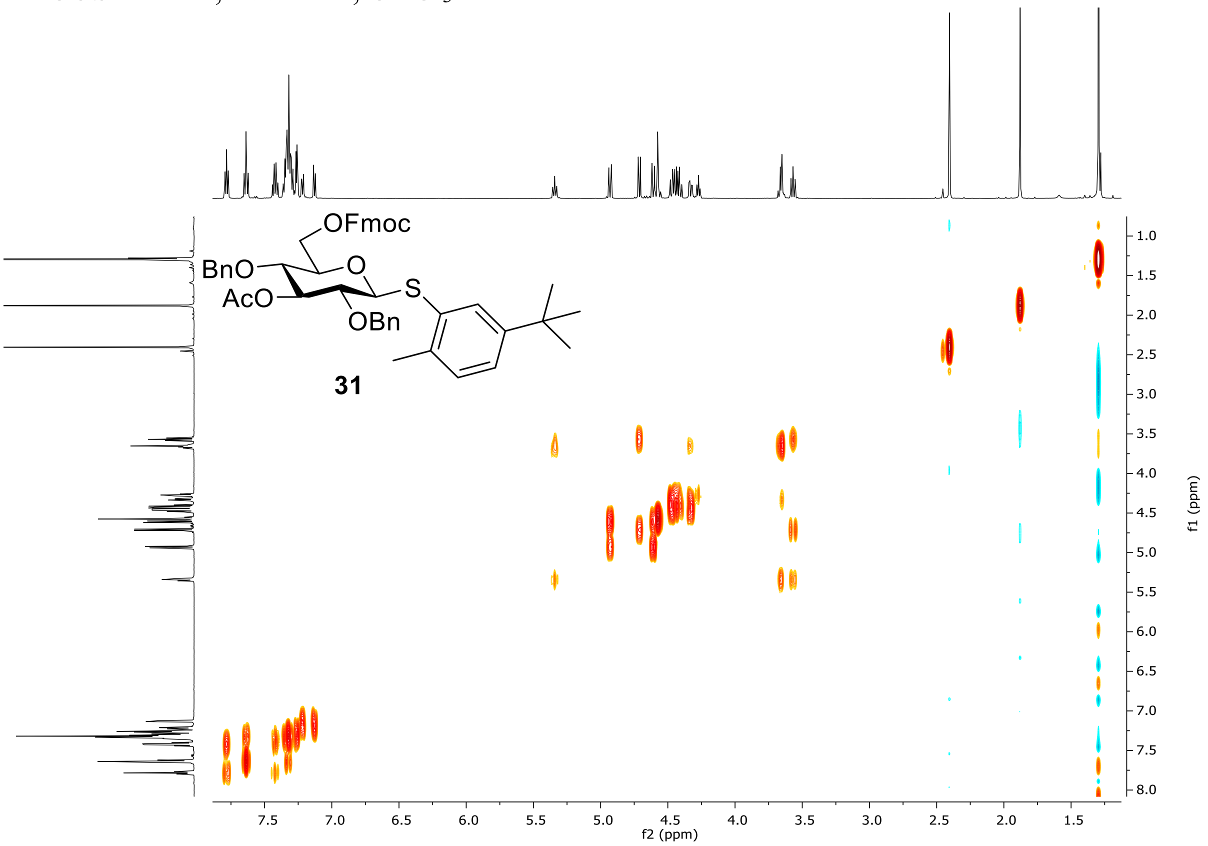

^{1}H-^{13}C-HSQC NMR, 400 MHz, $CDCl_3$

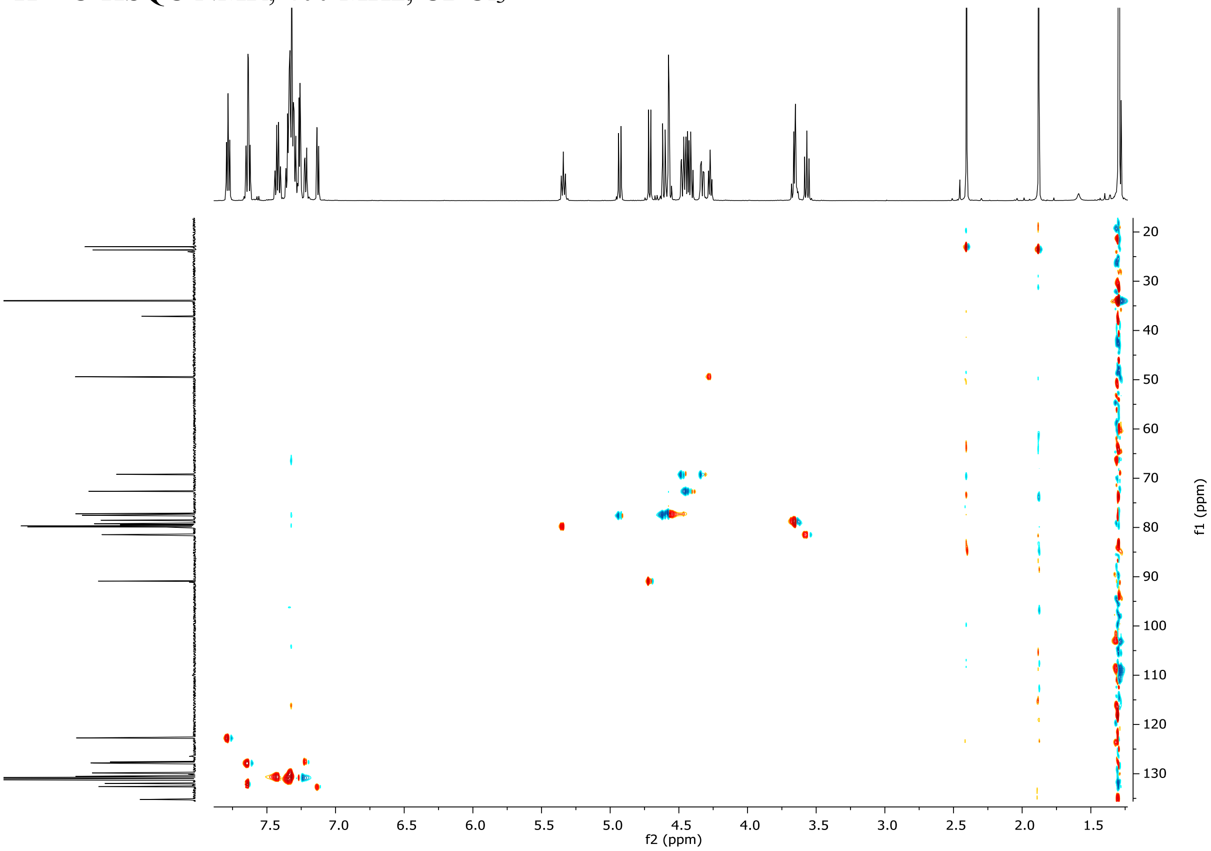

Supplementary Figure 82 | 2D NMR spectra of 31

^{1}H NMR, 400 MHz, $CDCl_3$

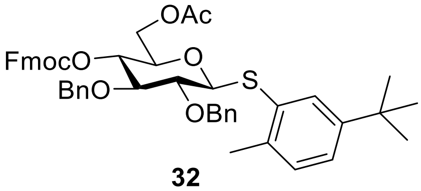

32

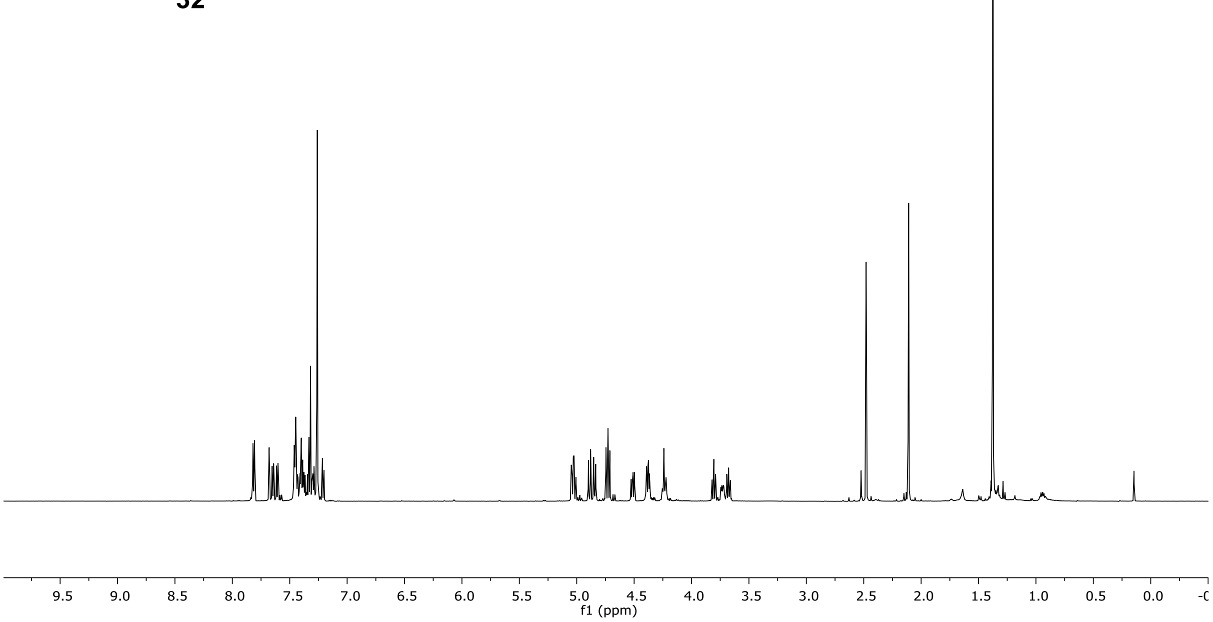

^{13}C NMR, 100 MHz, $CDCl_3$

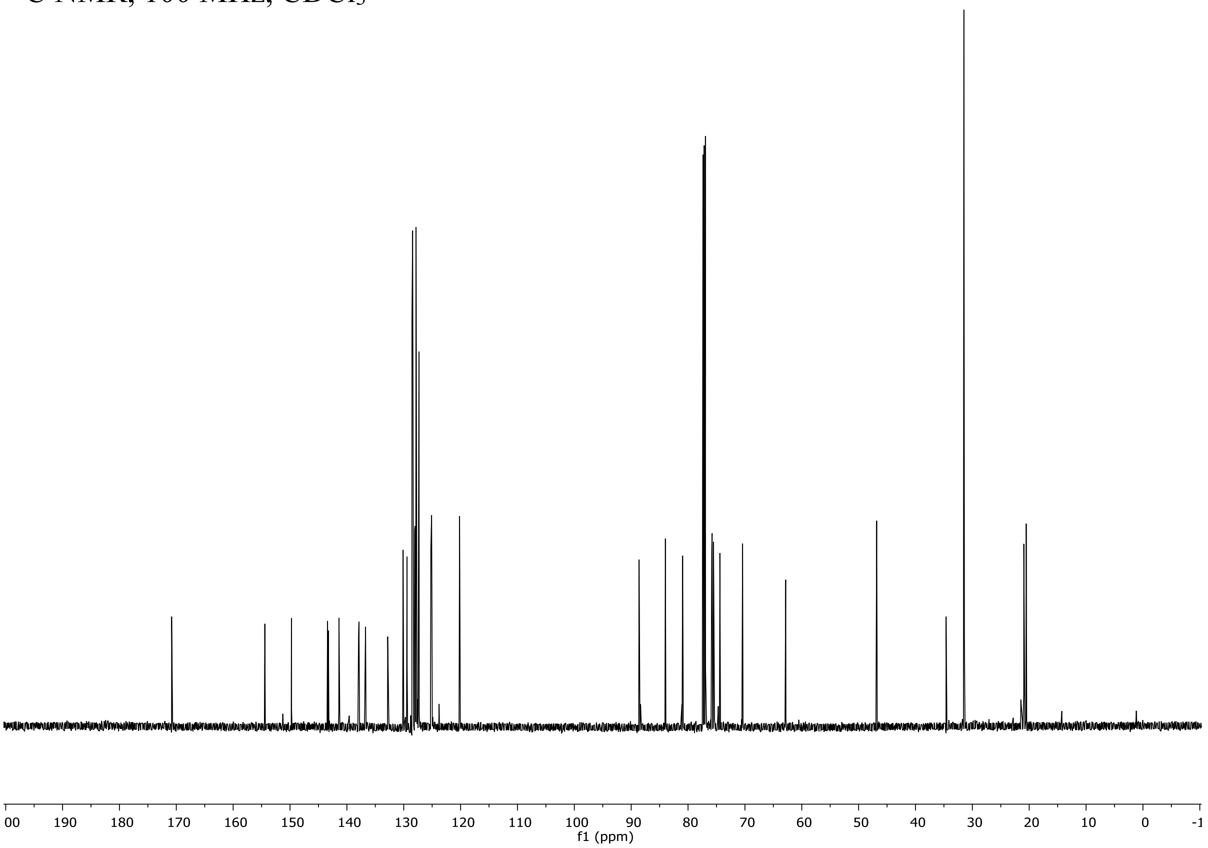

Supplementary Figure 83 | 1D NMR spectra of 32

^{1}H-COSY NMR, 400 MHz, $CDCl_3$

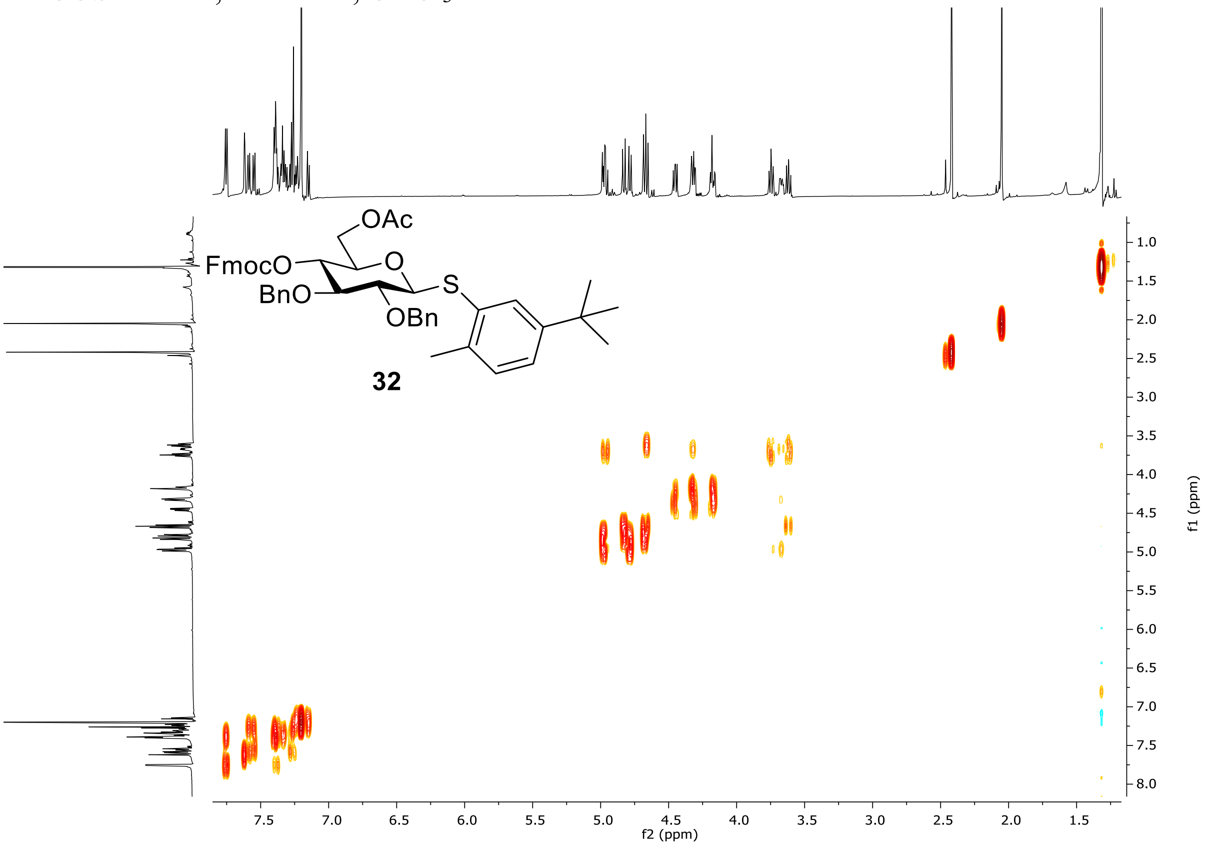

^{1}H-^{13}C-HSQC NMR, 400 MHz, $CDCl_3$

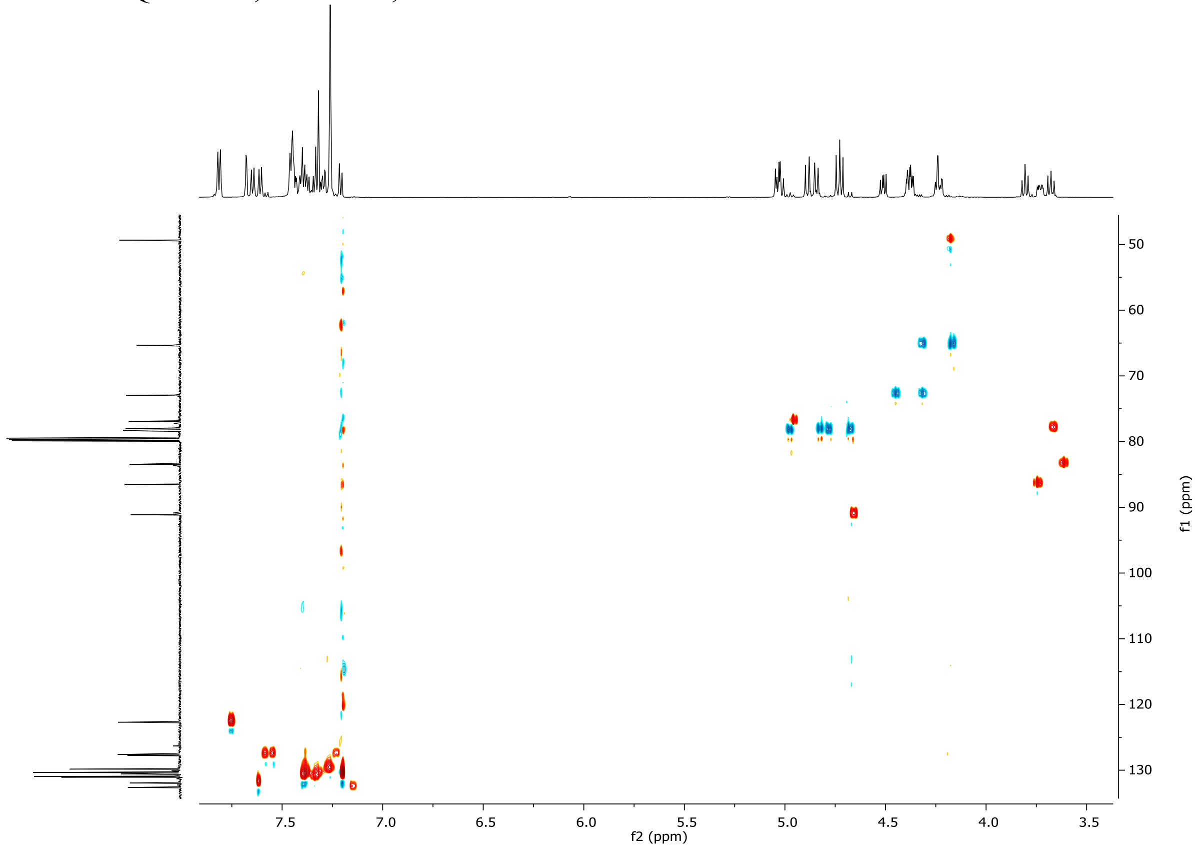

Supplementary Figure 84 | 2D NMR spectra of 32

^{1}H NMR, 400 MHz, $CDCl_3$

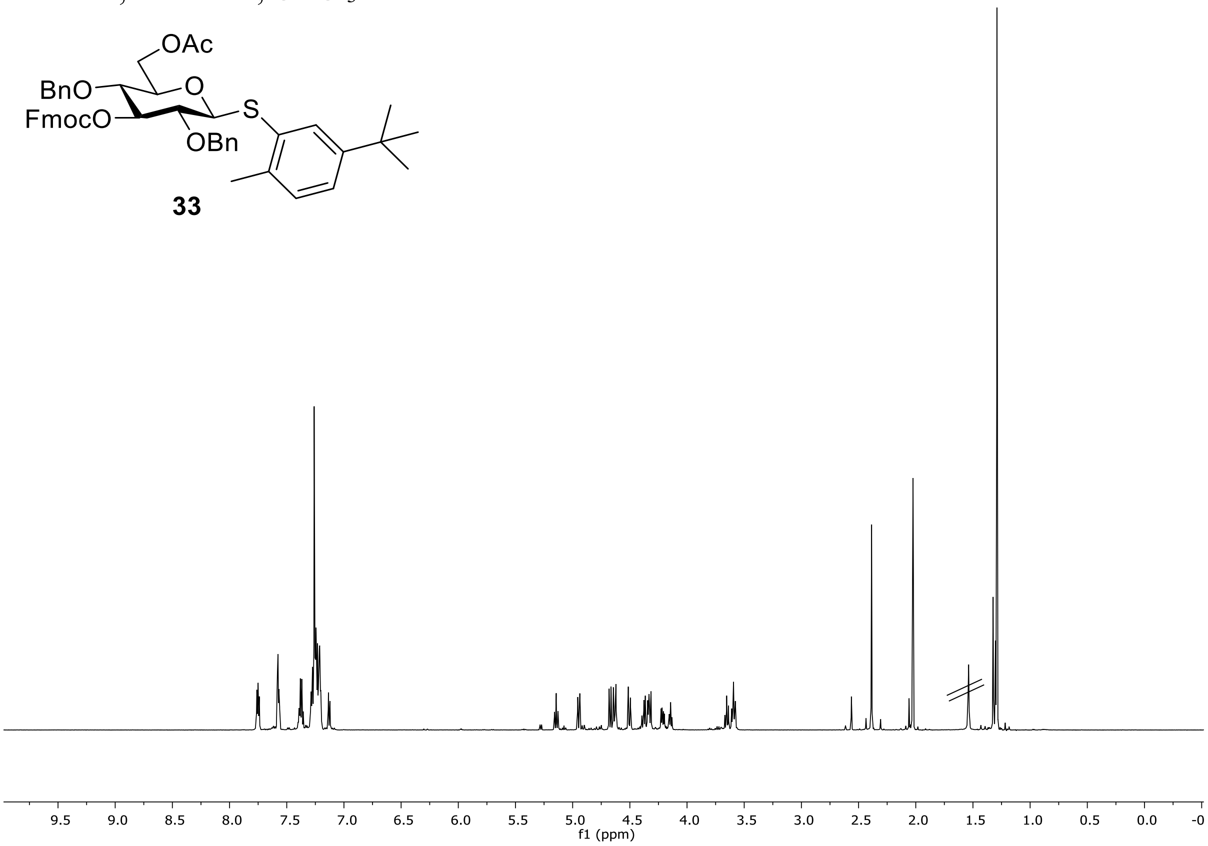

^{13}C NMR, 100 MHz, $CDCl_3$

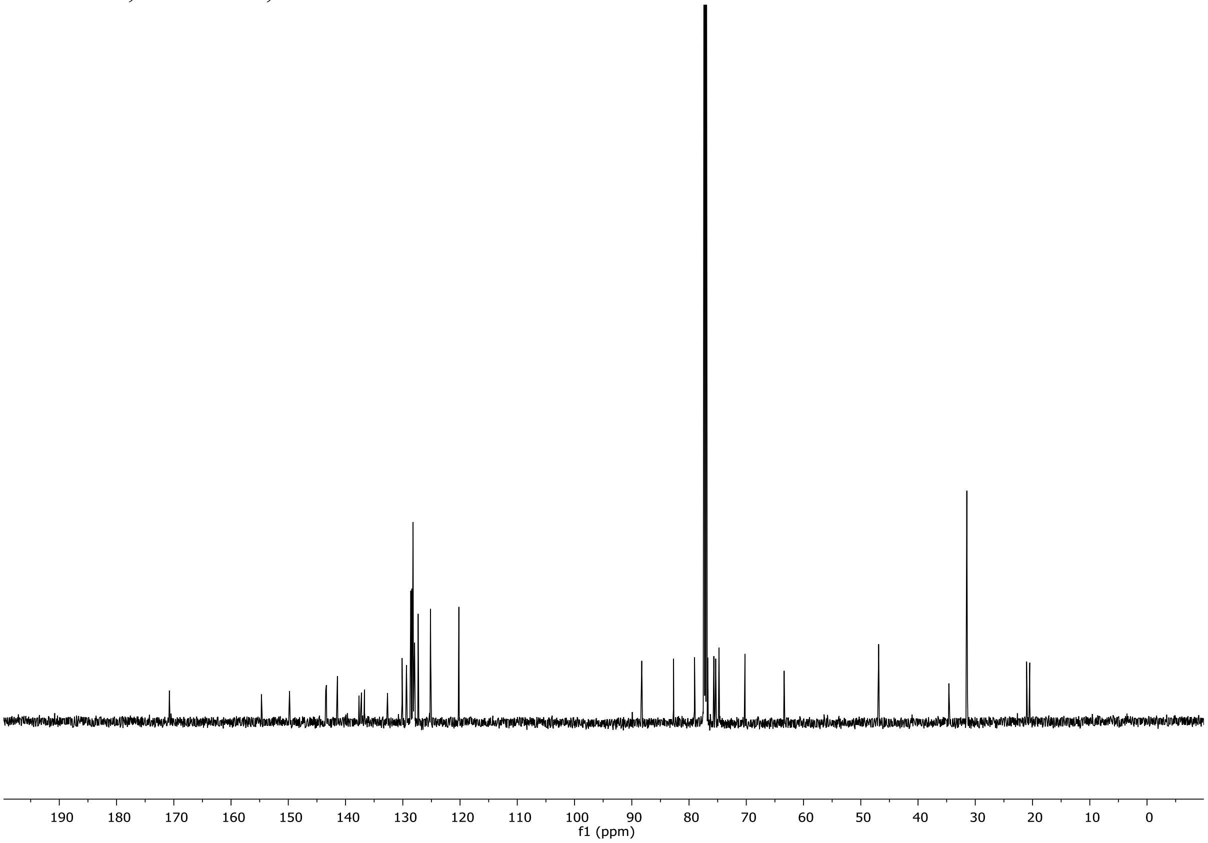

Supplementary Figure 85 | 1D NMR spectra of 33

^{1}H-COSY NMR, 400 MHz, $CDCl_3$

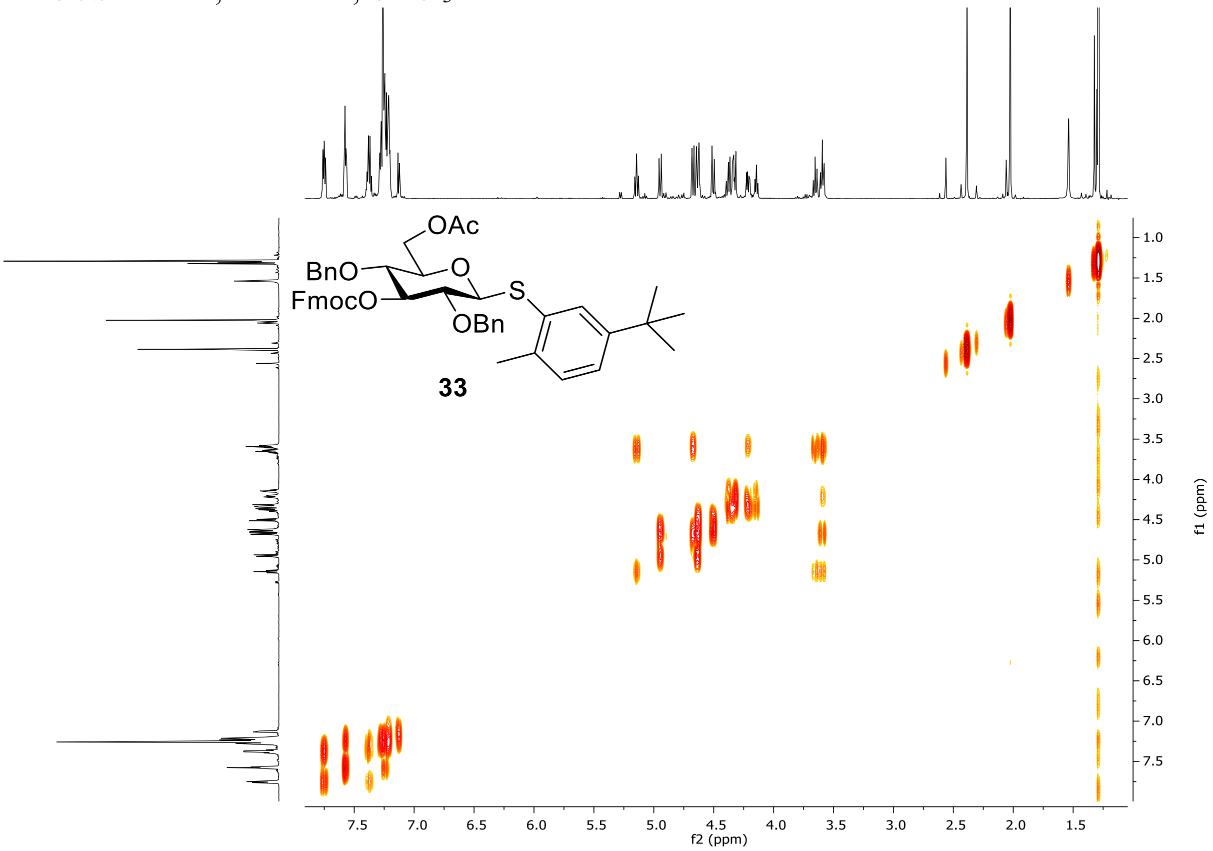

^{1}H-^{13}C-HSQC NMR, 400 MHz, $CDCl_3$

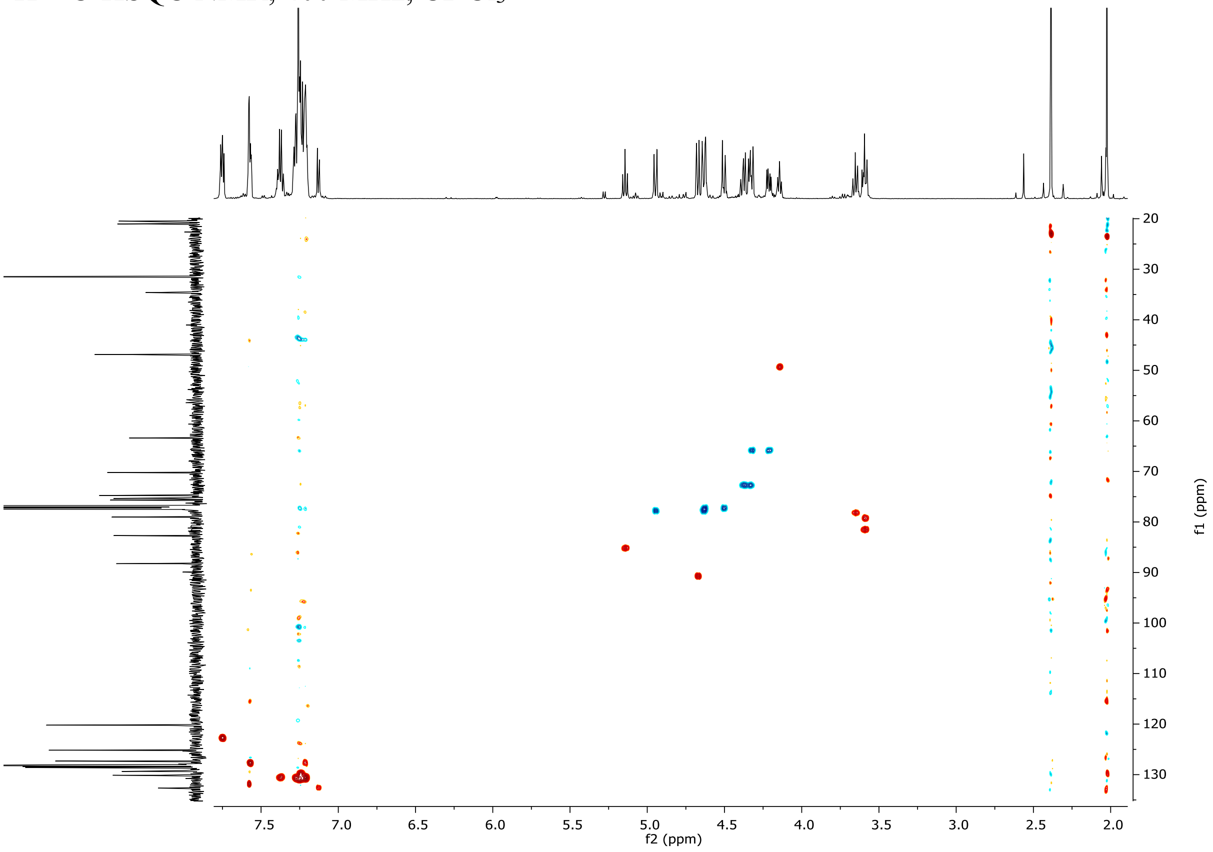

Supplementary Figure 86 | 2D NMR spectra of 33

^{1}H NMR, 600 MHz, $CDCl_3$

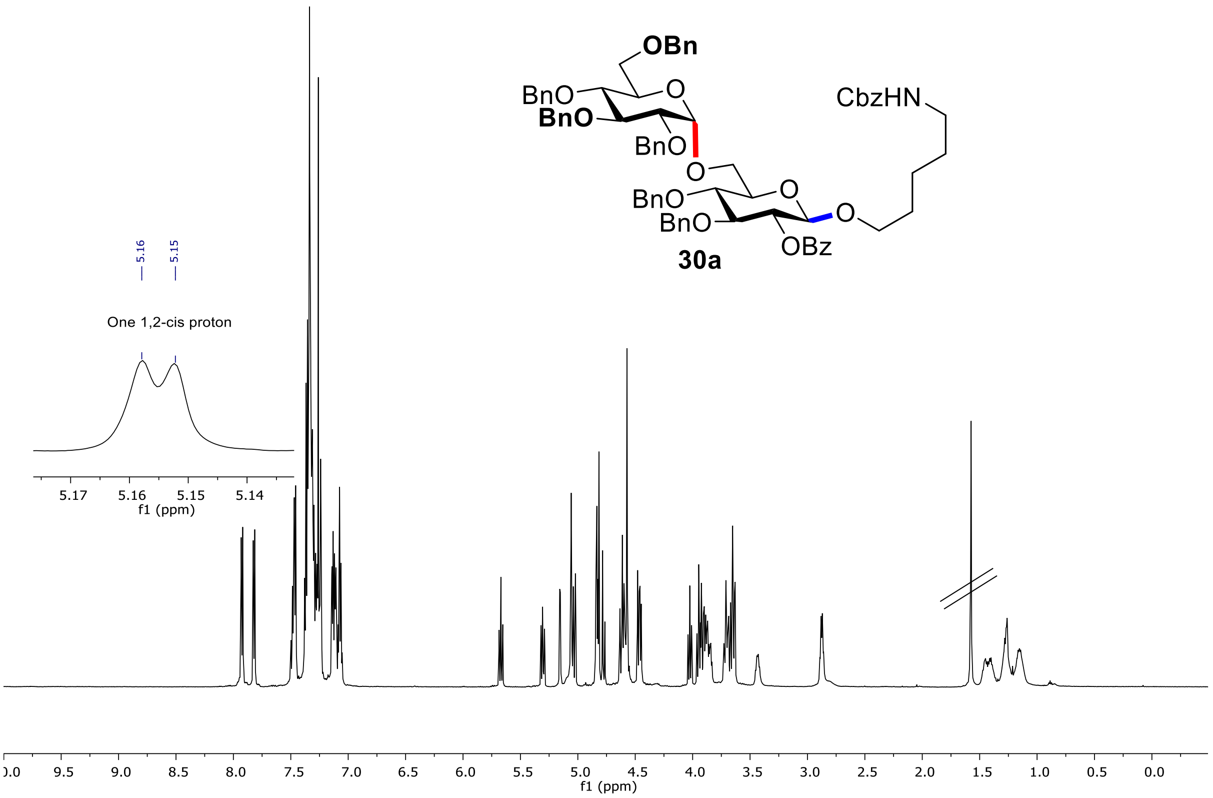

^{13}C NMR, 150 MHz, $CDCl_3$

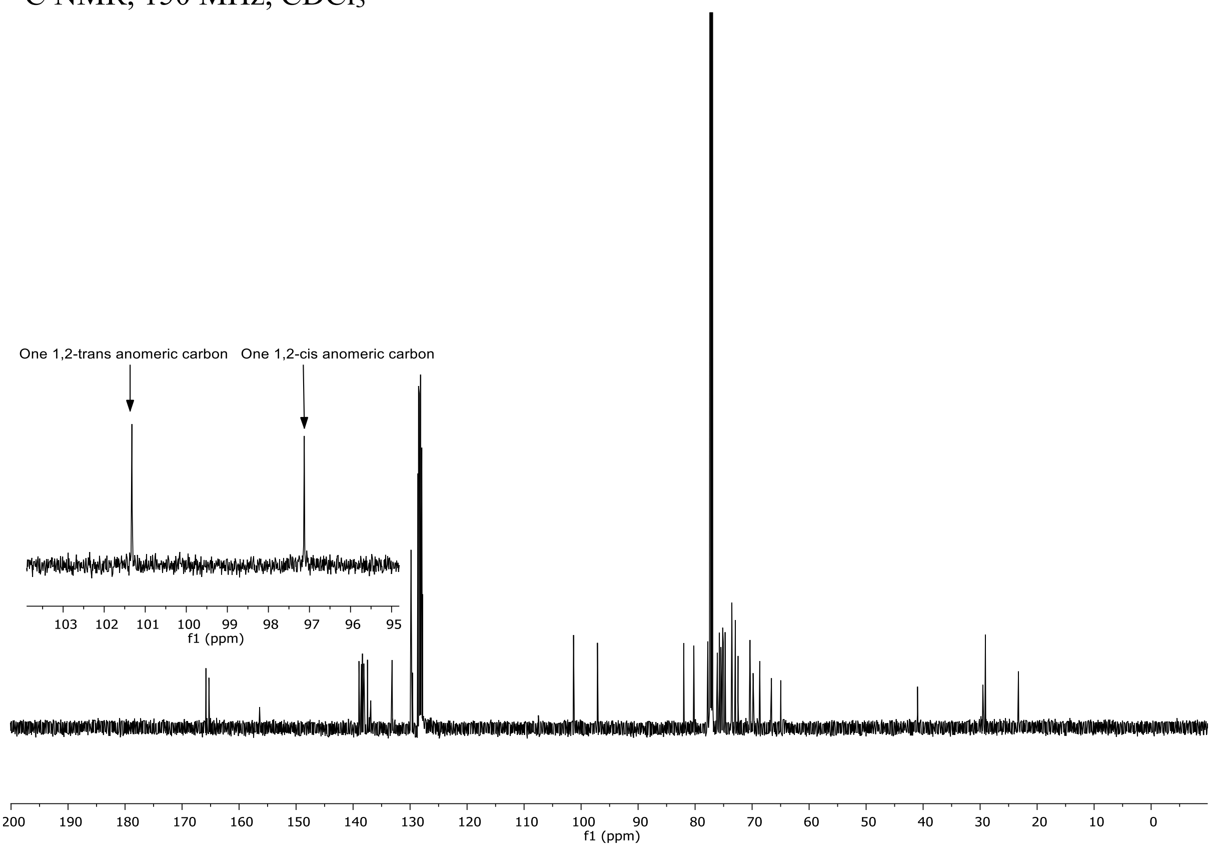

Supplementary Figure 87 | 1D NMR spectra of 30a

^{1}H-COSY NMR, 600 MHz, $CDCl_3$

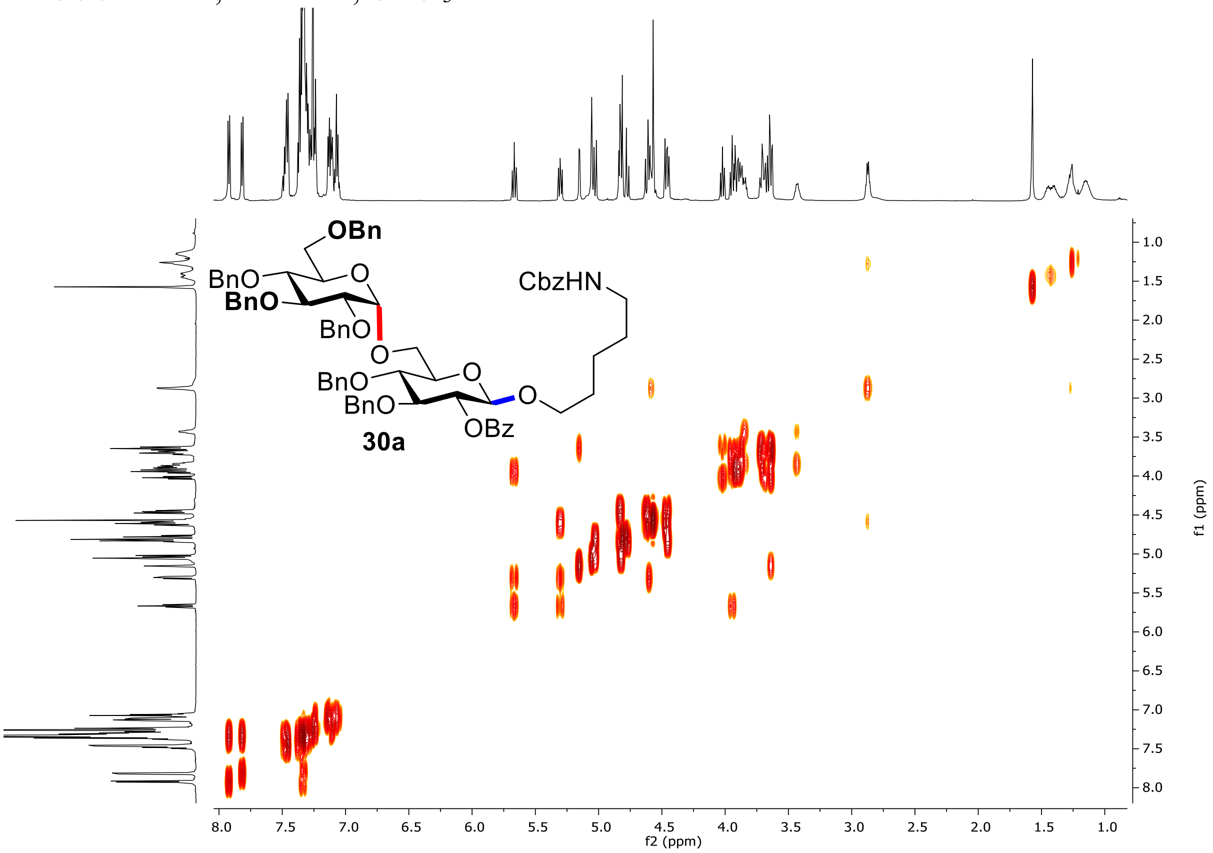

^{1}H-^{13}C-HSQC and ^{1}H-^{13}C-coupled-HSQC (zoom-in) NMR, 600 MHz, $CDCl_3$

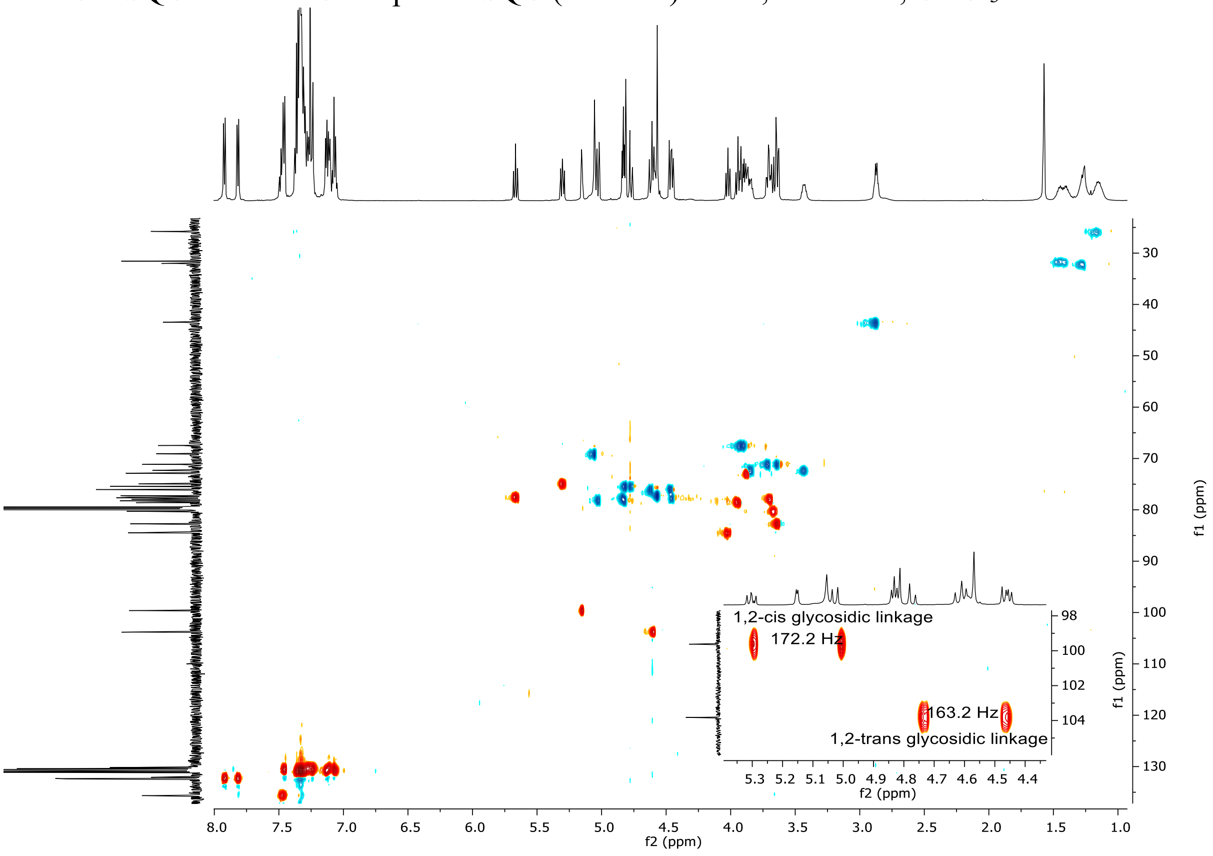

Supplementary Figure 88 | 2D NMR spectra of 30a

^{1}H NMR, 600 MHz, $CDCl_3$

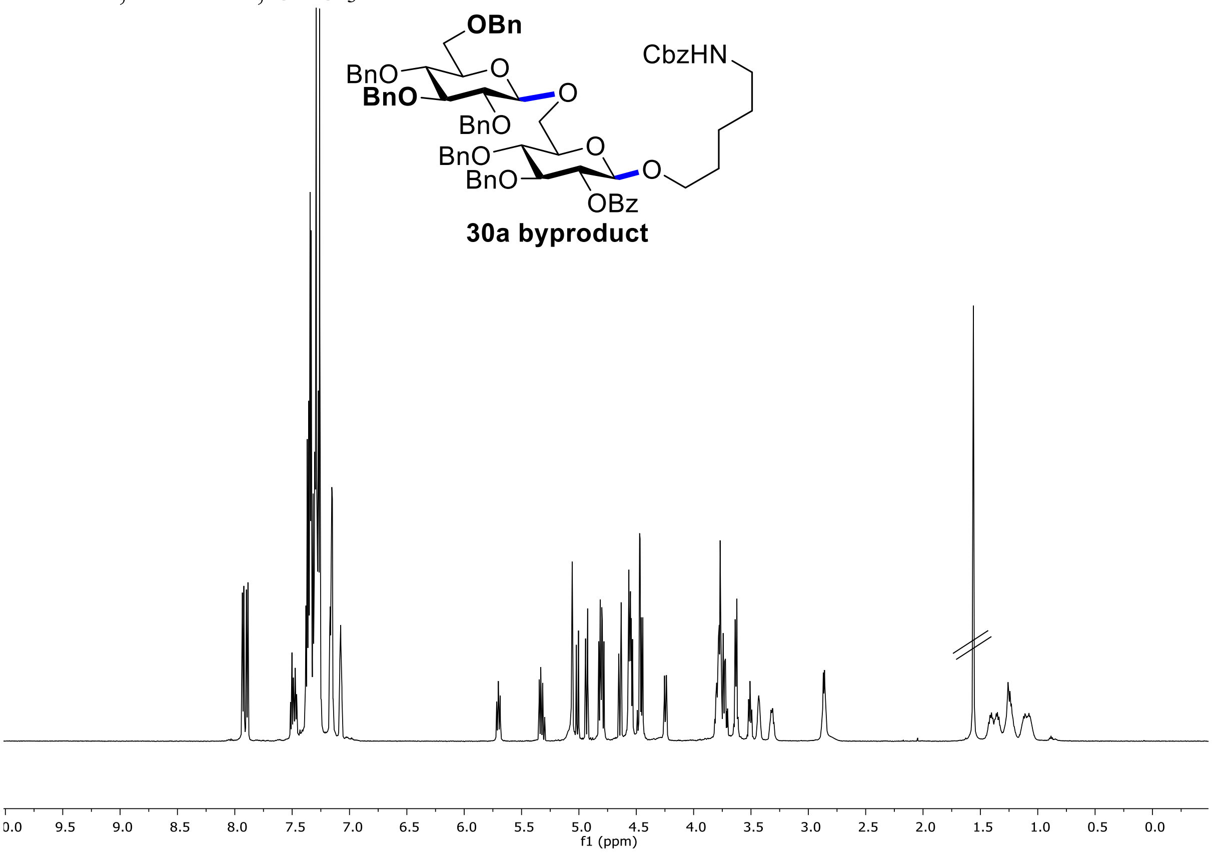

^{13}C NMR, 150 MHz, $CDCl_3$

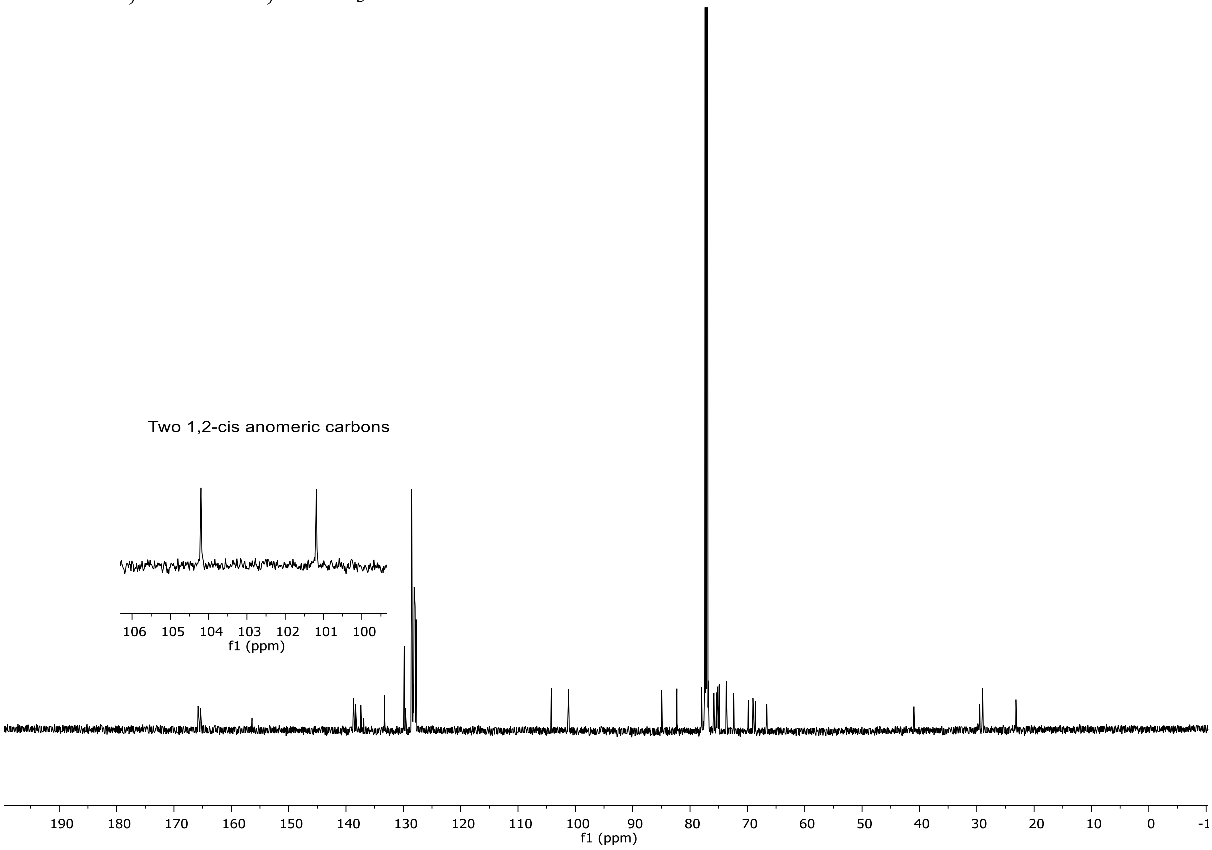

Supplementary Figure 89 | 1D NMR spectra of 30a byproduct

^{1}H-COSY NMR, 600 MHz, CDCl$_3$

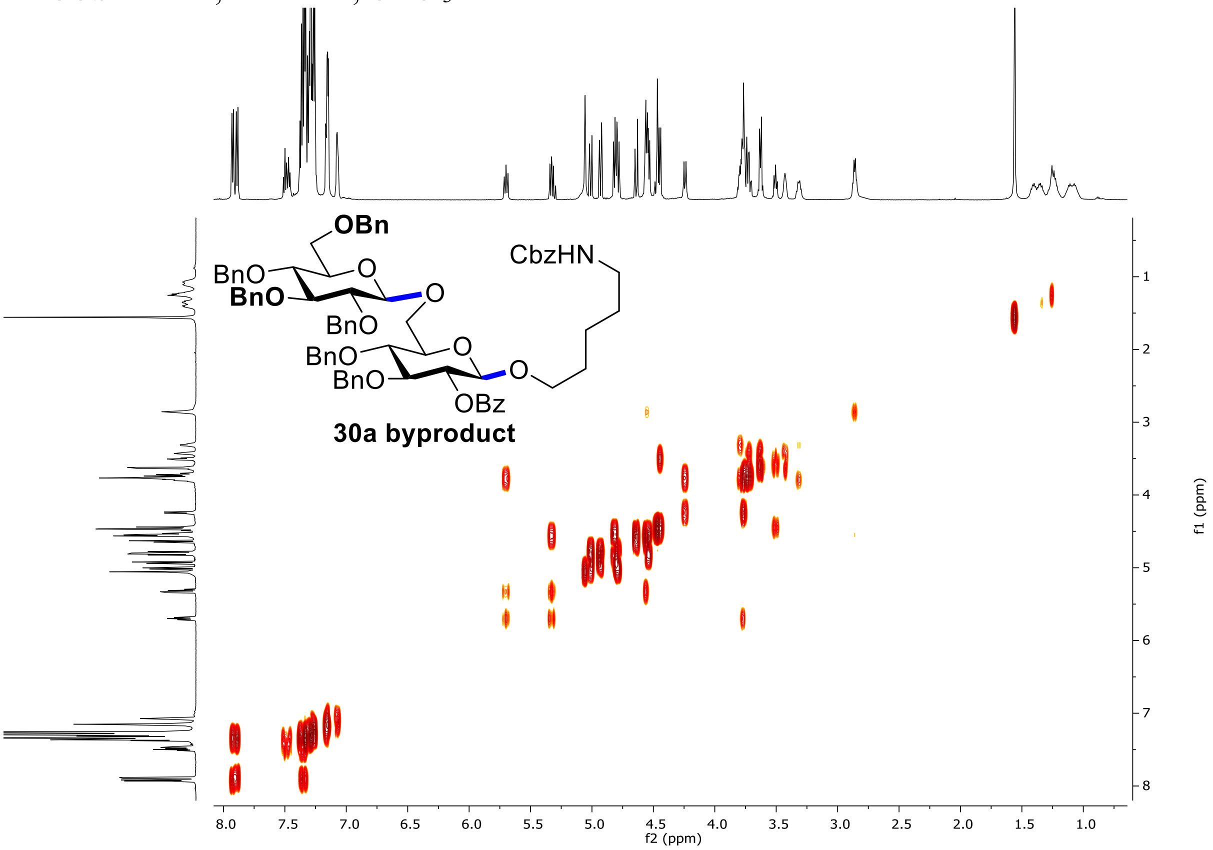

^{1}H-^{13}C-HSQC and ^{1}H-^{13}C-coupled-HSQC (zoom-in) NMR, 600 MHz, CDCl$_3$

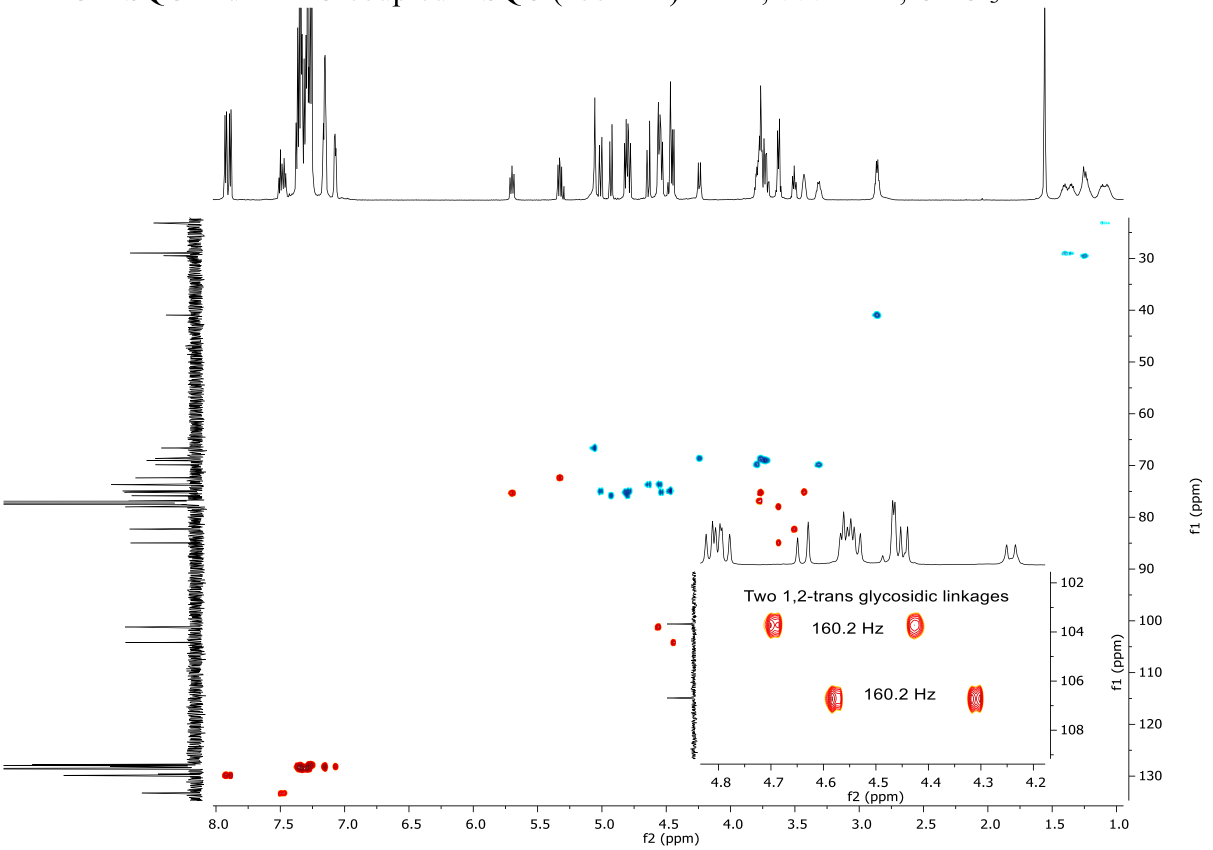

Supplementary Figure 90 | 2D NMR spectra of 30a byproduct

^{1}H NMR, 600 MHz, $CDCl_3$

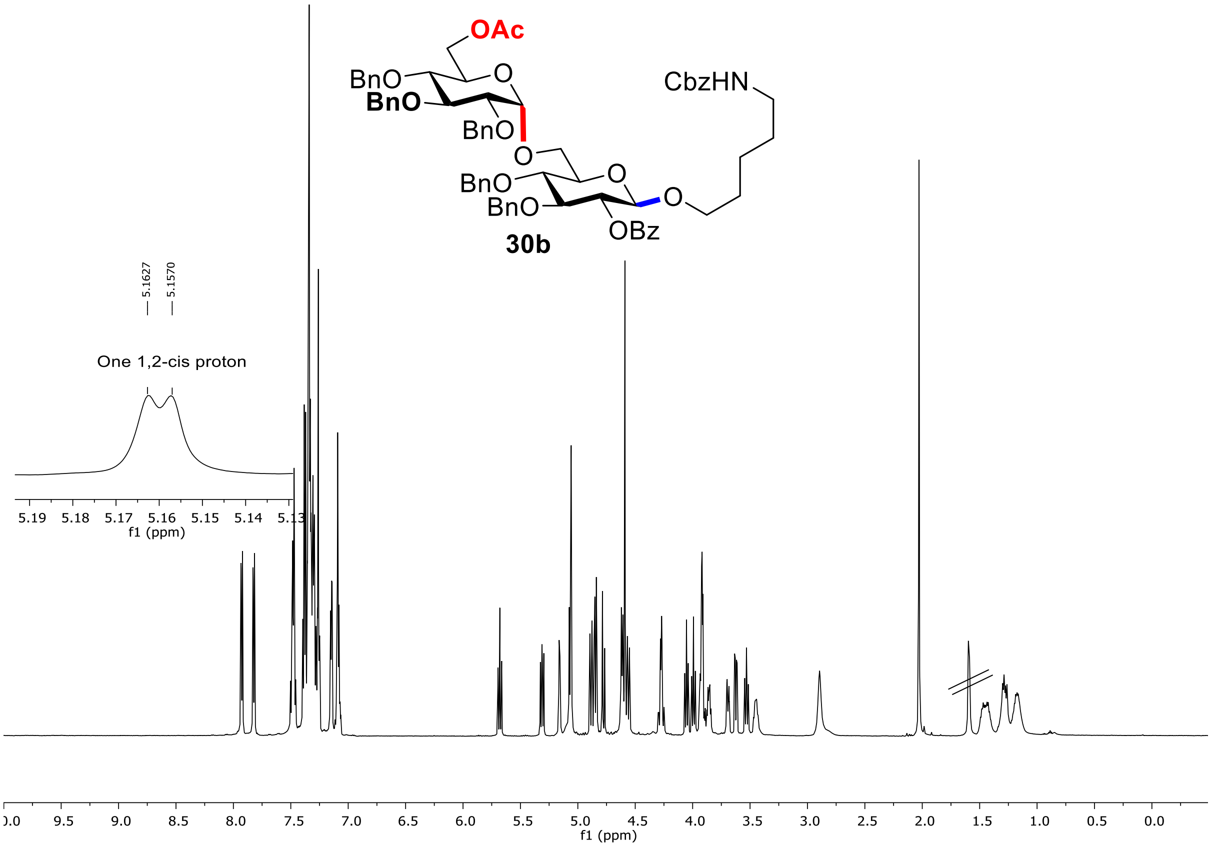

^{13}C NMR, 150 MHz, $CDCl_3$

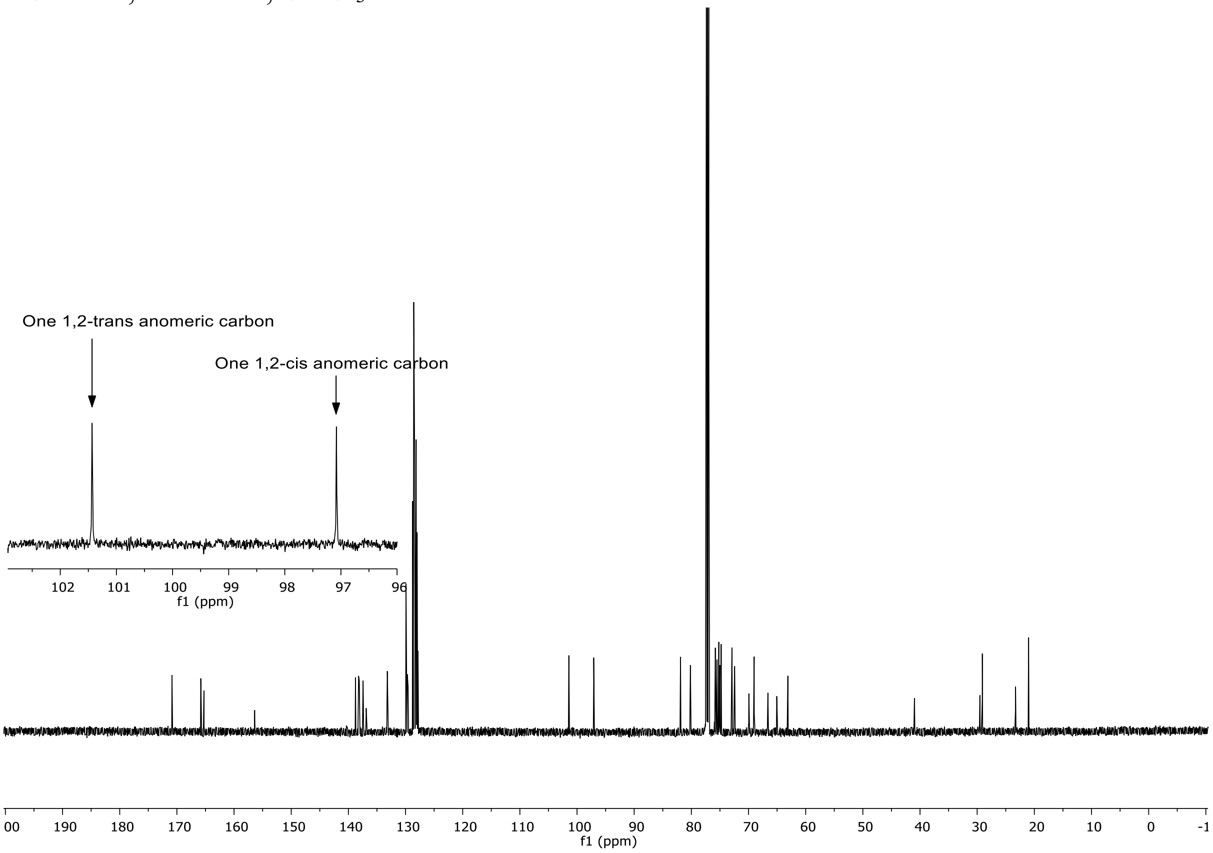

Supplementary Figure 91 | 1D NMR spectra of 30b

^{1}H-COSY NMR, 600 MHz, $CDCl_3$

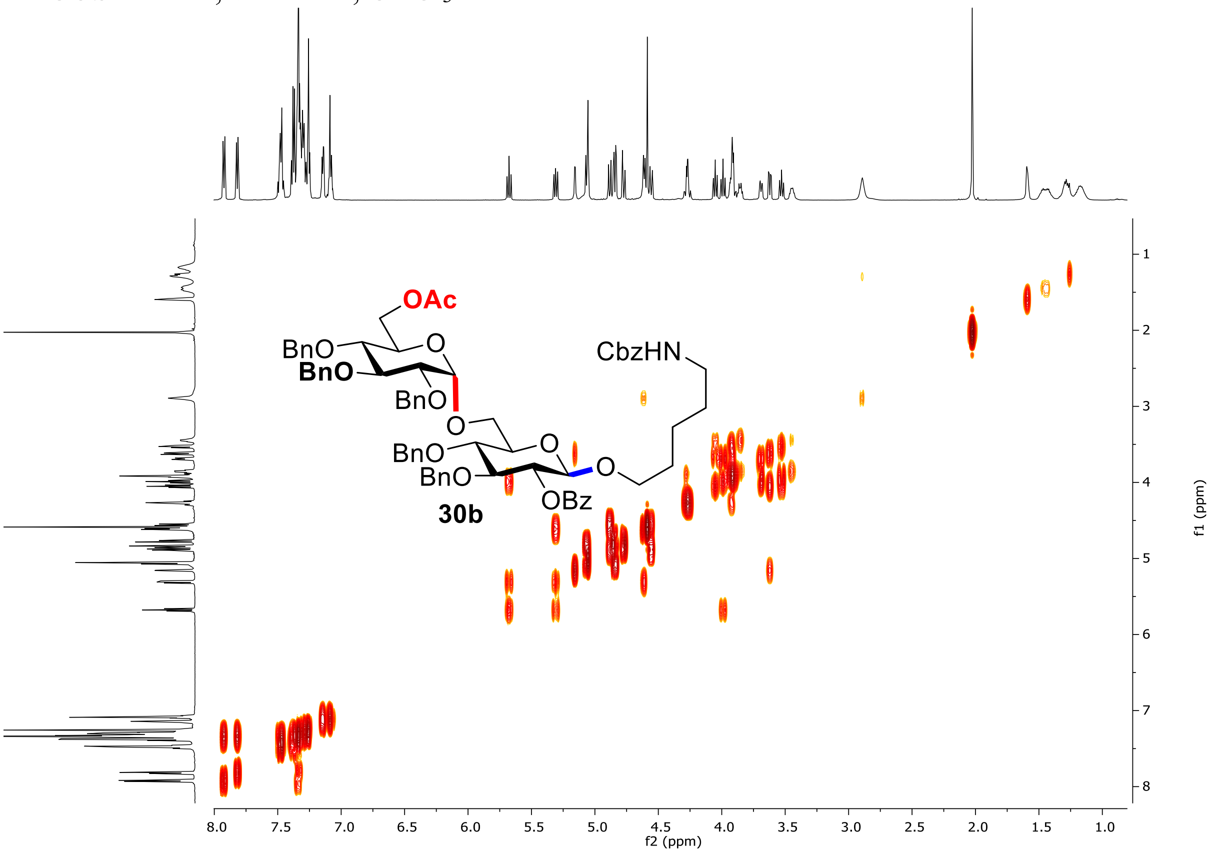

^{1}H-^{13}C-HSQC and ^{1}H-^{13}C-coupled-HSQC (zoom-in) NMR, 600 MHz, $CDCl_3$

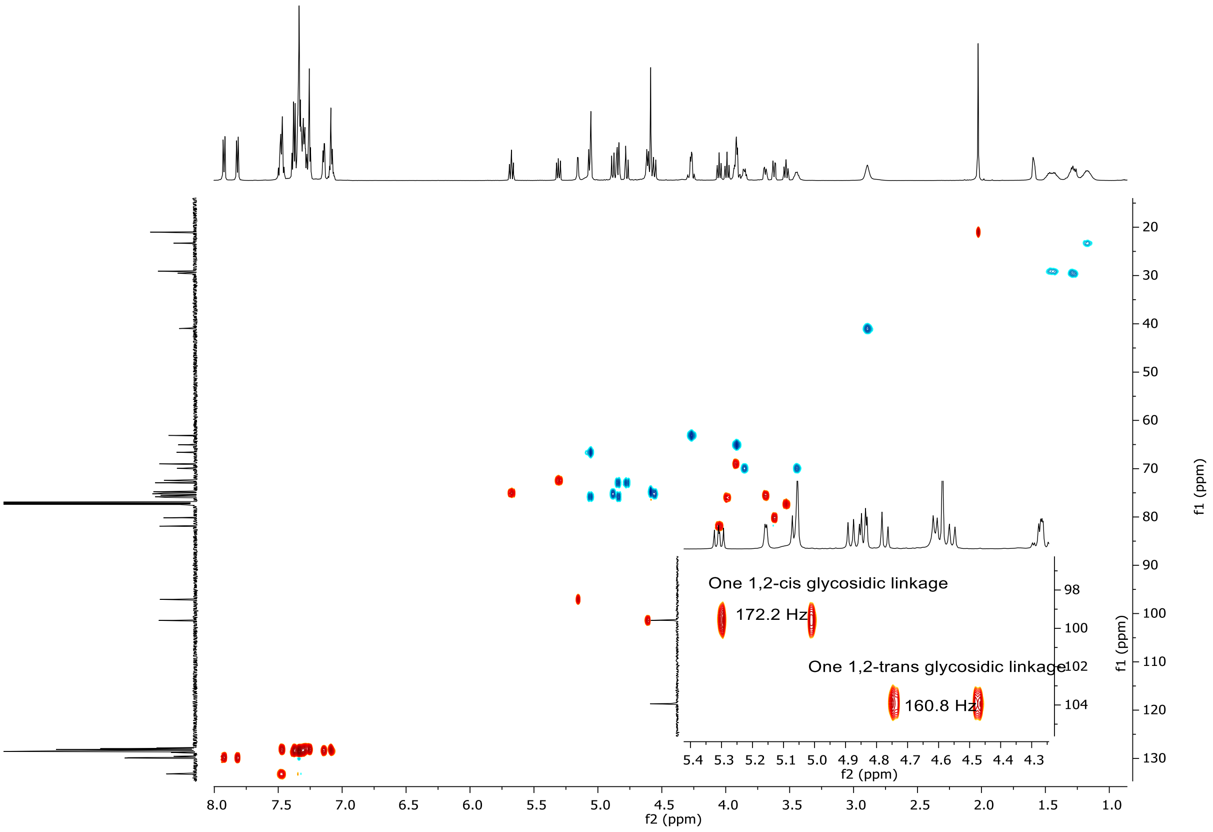

Supplementary Figure 92 | 2D NMR spectra of 30b

^{1}H NMR, 600 MHz, $CDCl_3$

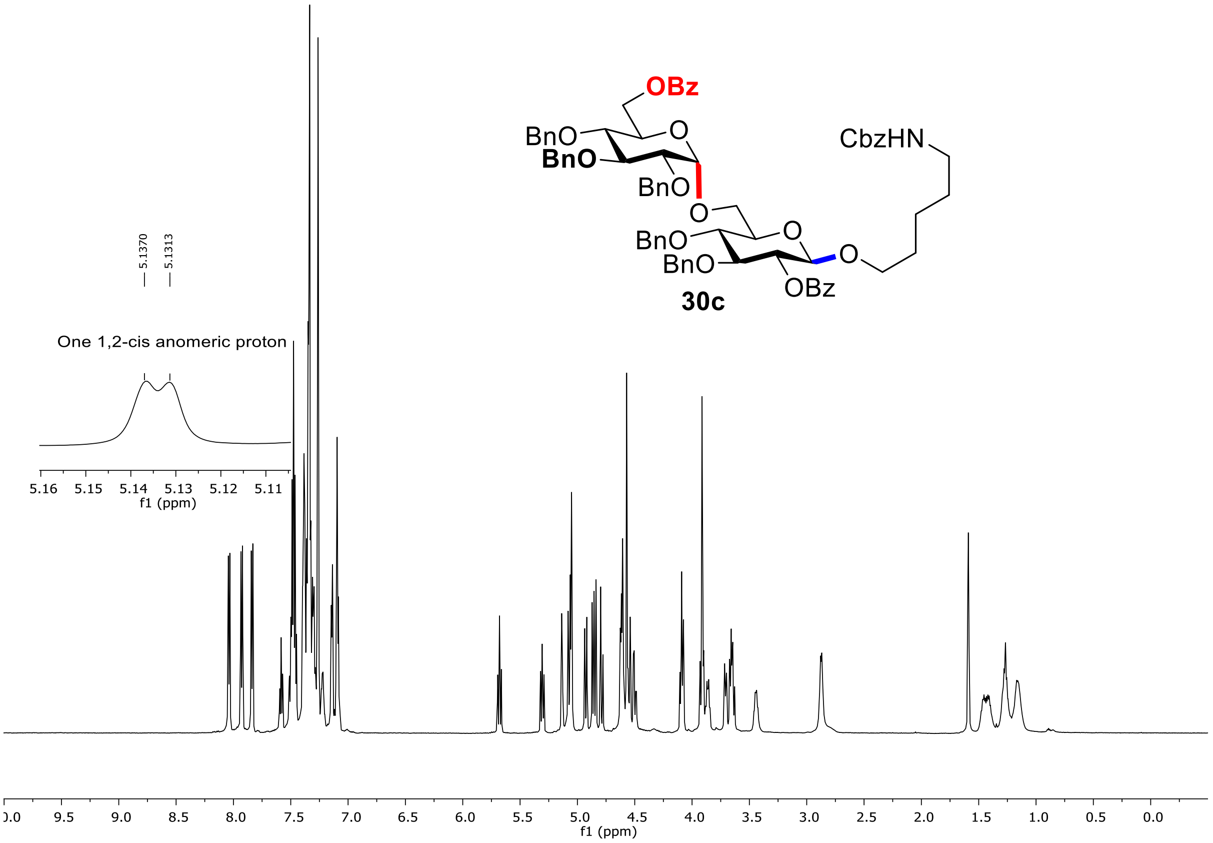

^{13}C NMR, 150 MHz, $CDCl_3$

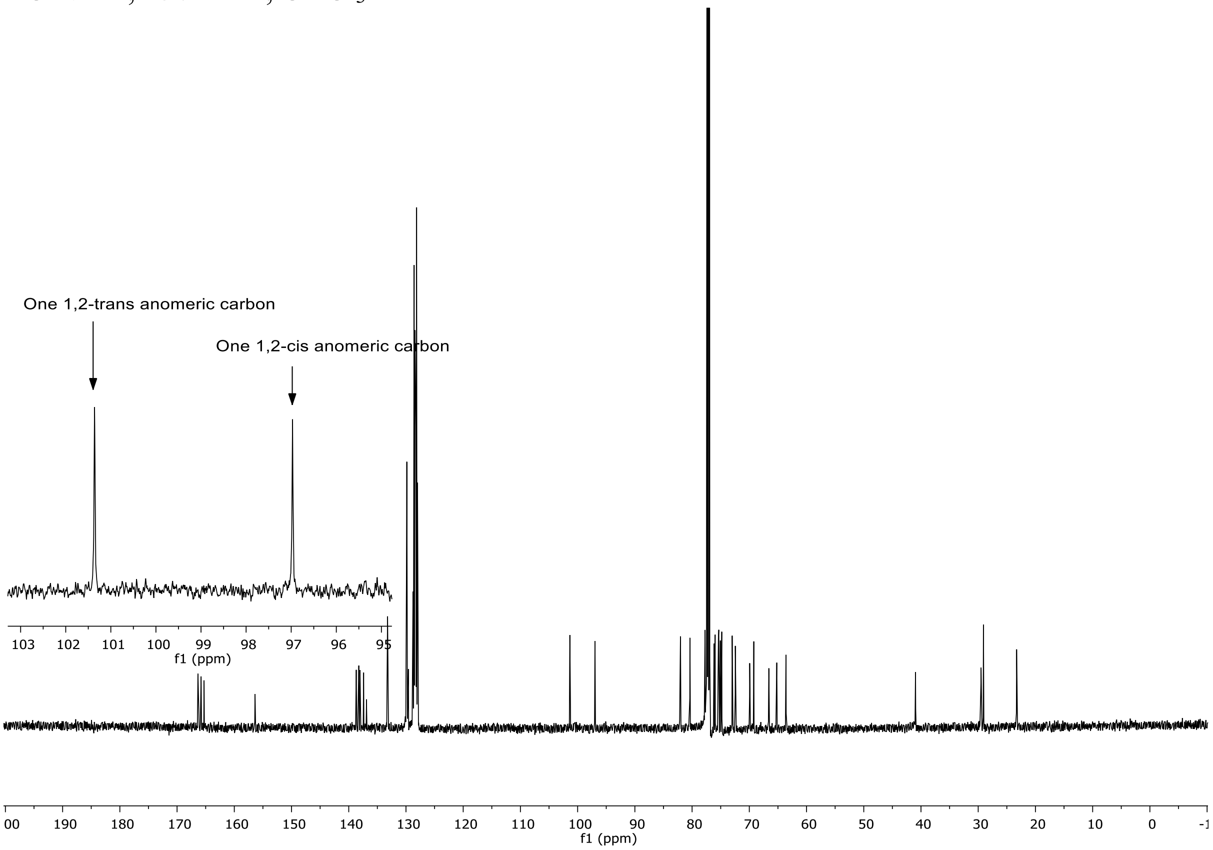

Supplementary Figure 93 | 1D NMR spectra of 30c

^{1}H-COSY NMR, 600 MHz, $CDCl_3$

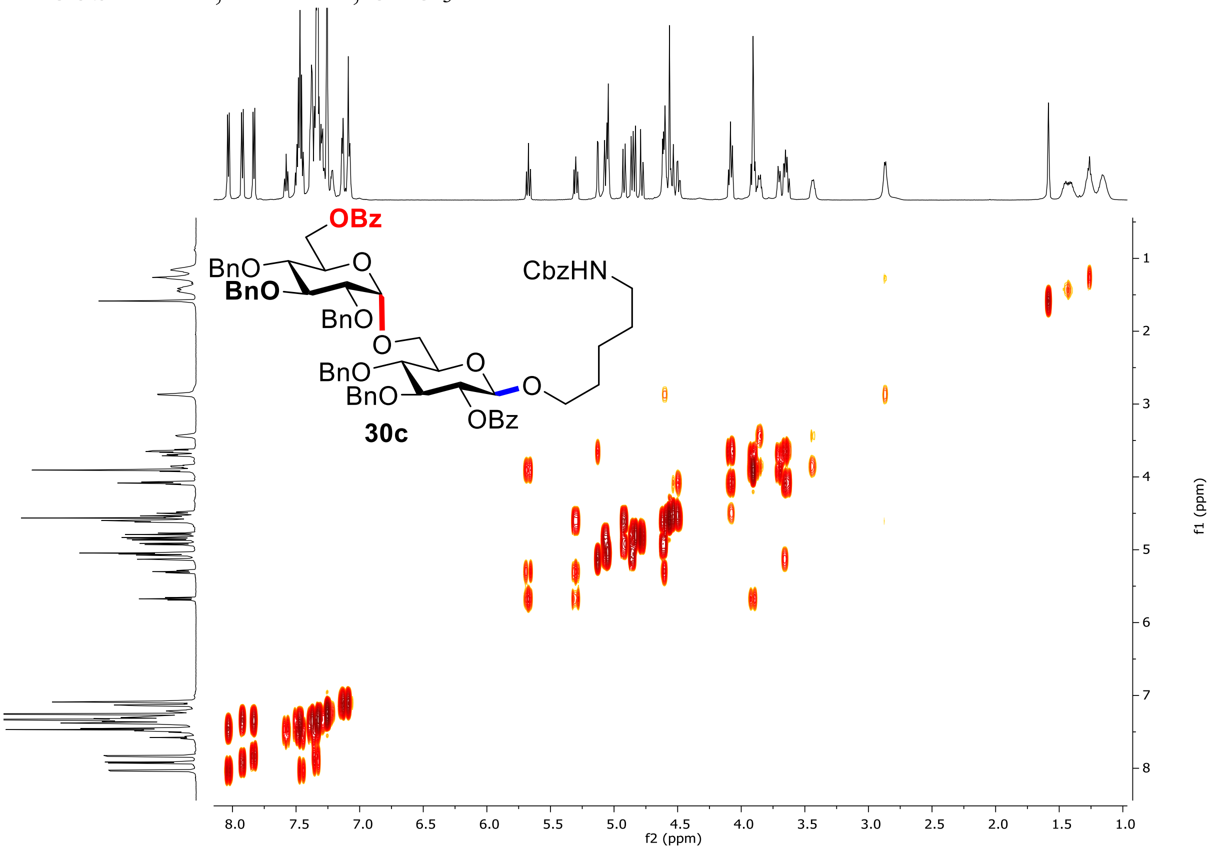

^{1}H-^{13}C-HSQC and ^{1}H-^{13}C-coupled-HSQC (zoom-in) NMR, 600 MHz, $CDCl_3$

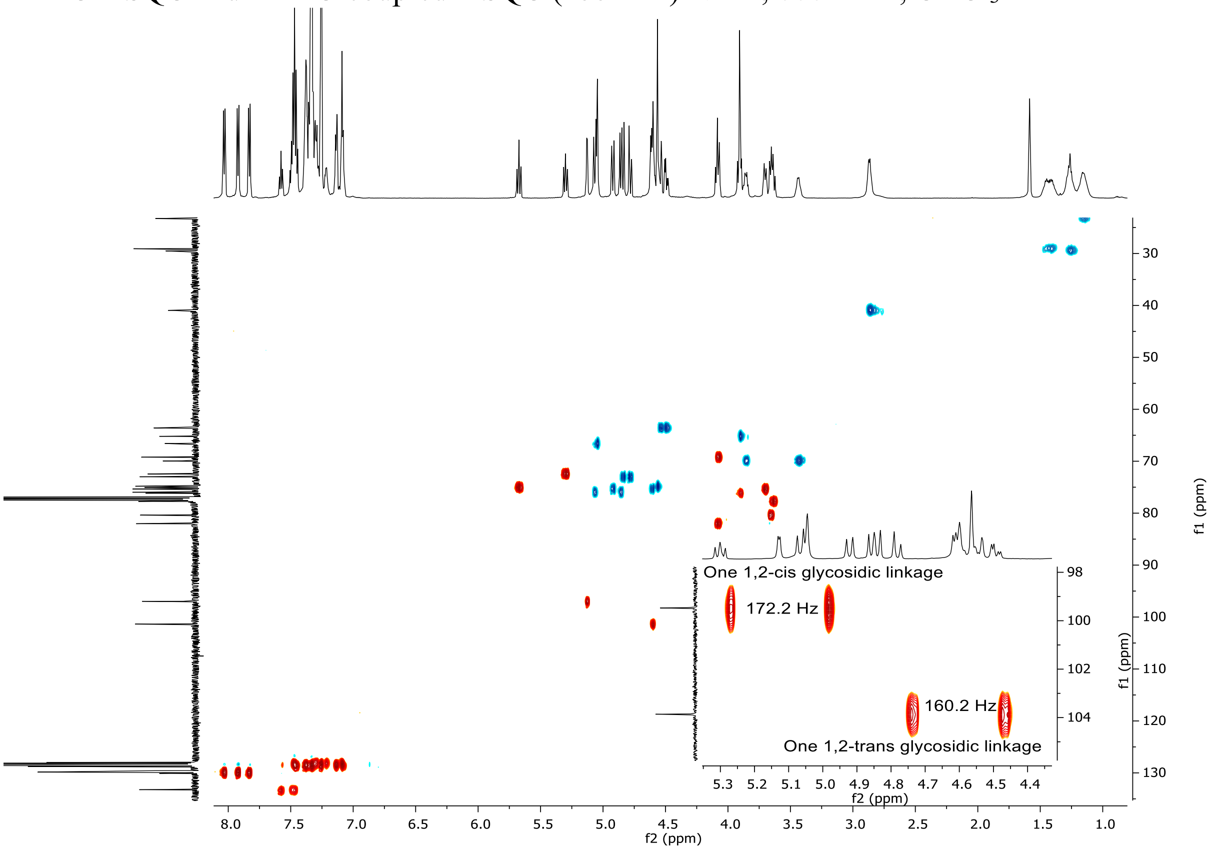

Supplementary Figure 94 | 2D NMR spectra of 30c

^{1}H NMR, 600 MHz, $CDCl_3$

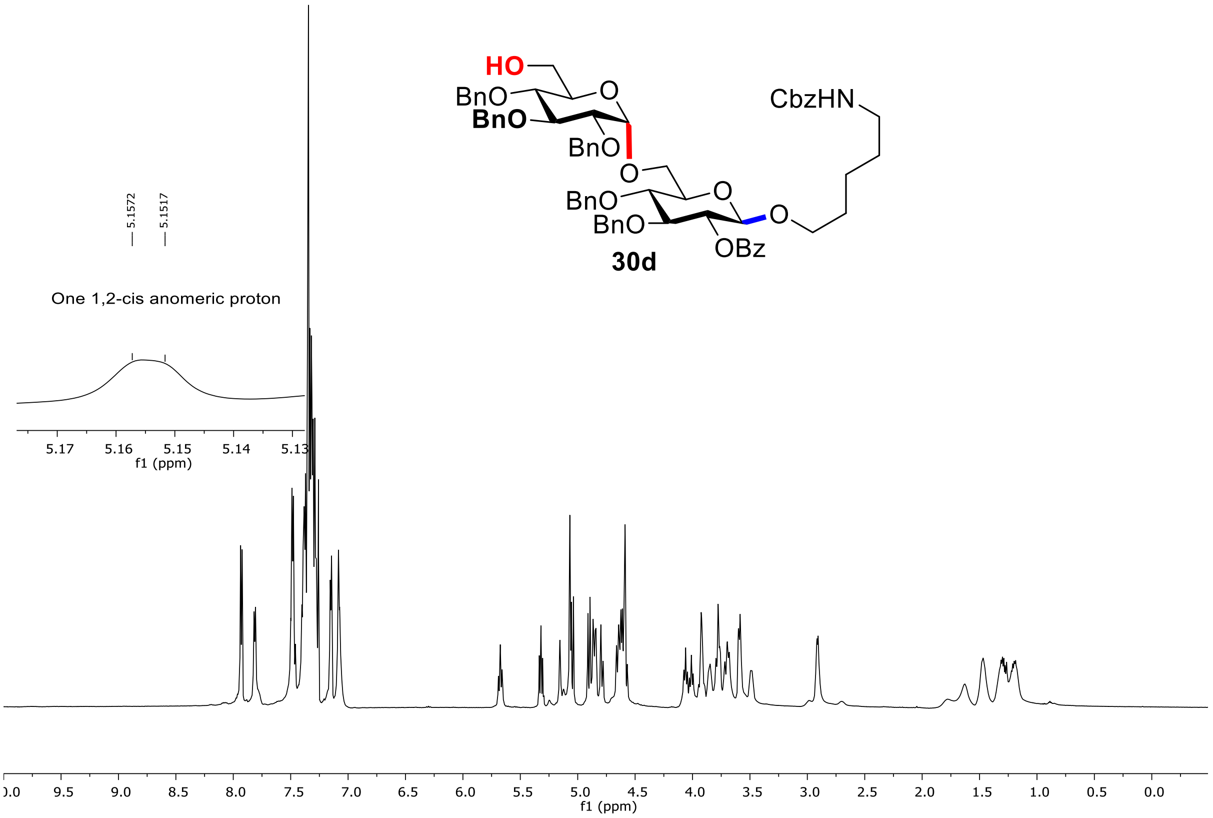

^{13}C NMR, 150 MHz, $CDCl_3$

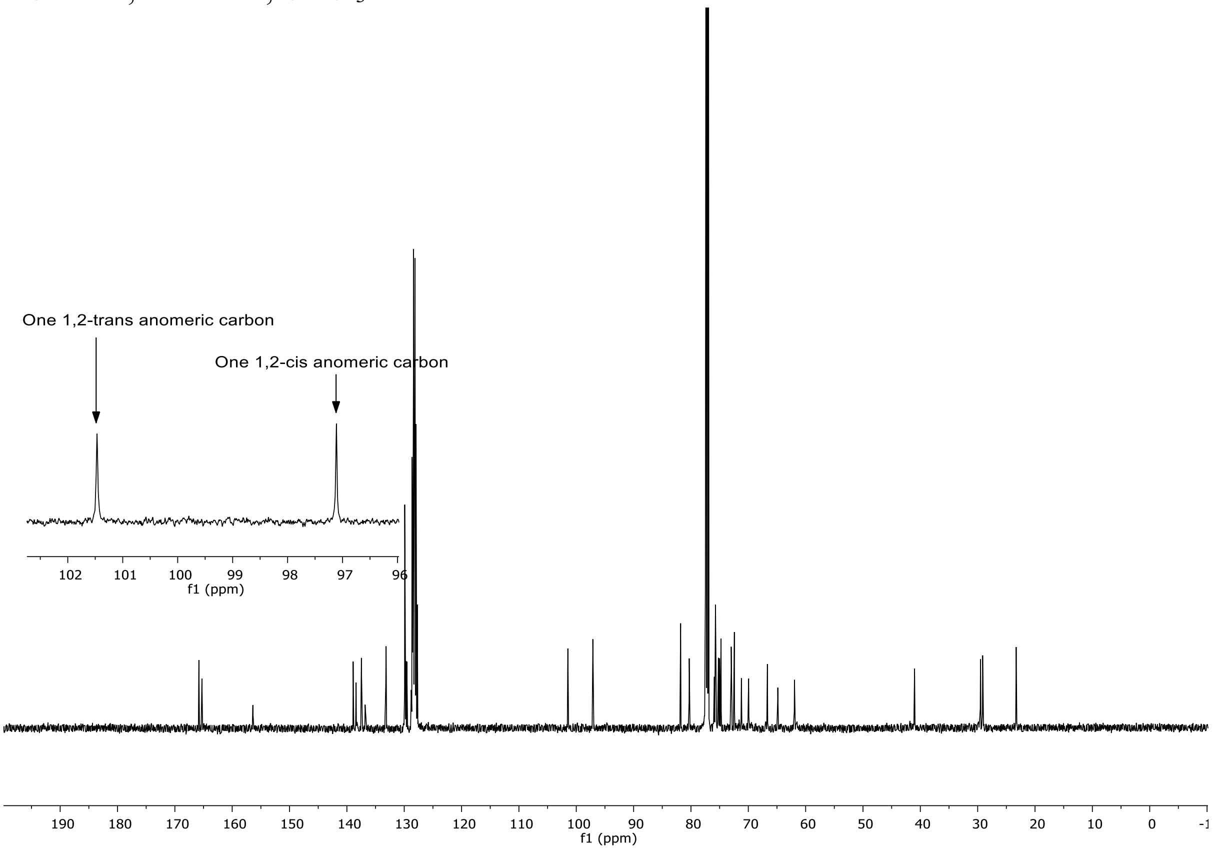

Supplementary Figure 95 | 1D NMR spectra of 30d

^{1}H-COSY NMR, 600 MHz, $CDCl_3$

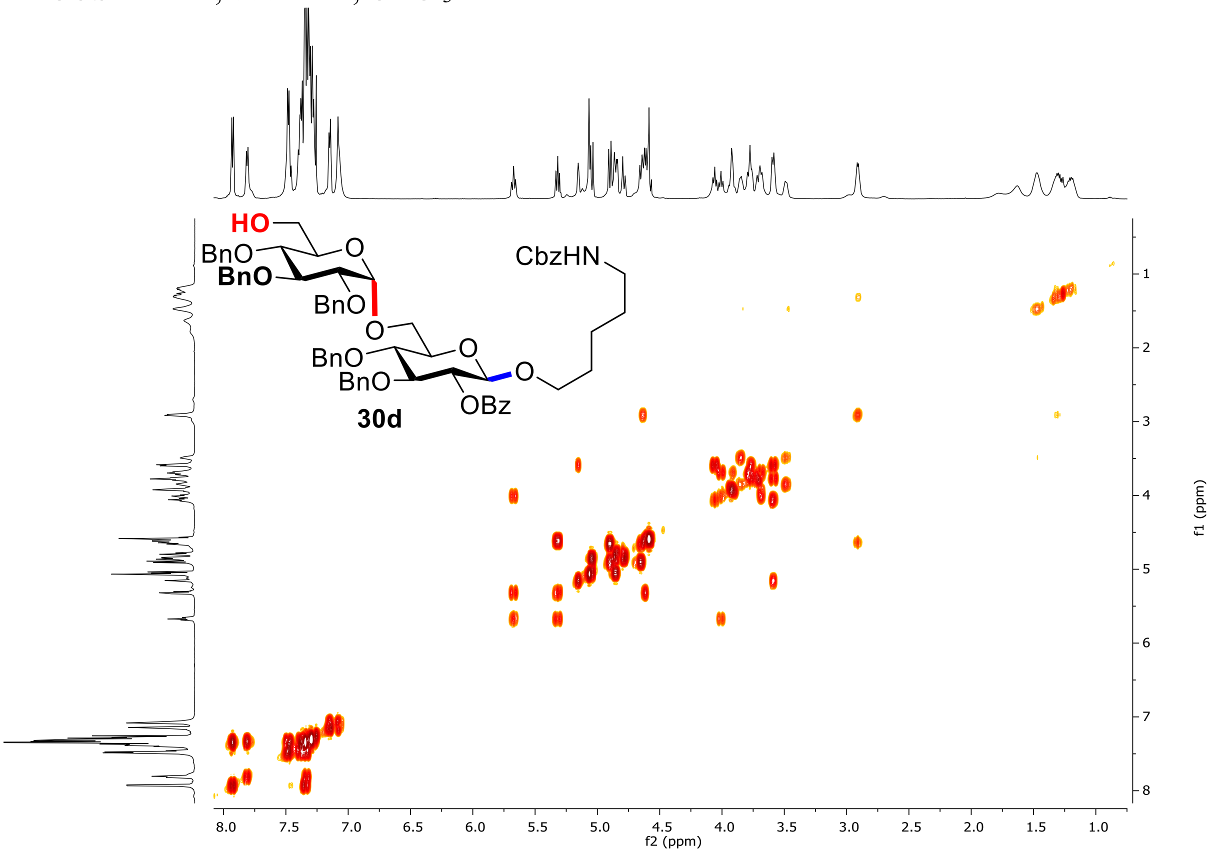

^{1}H-^{13}C-HSQC and ^{1}H-^{13}C-coupled-HSQC (zoom-in) NMR, 600 MHz, $CDCl_3$

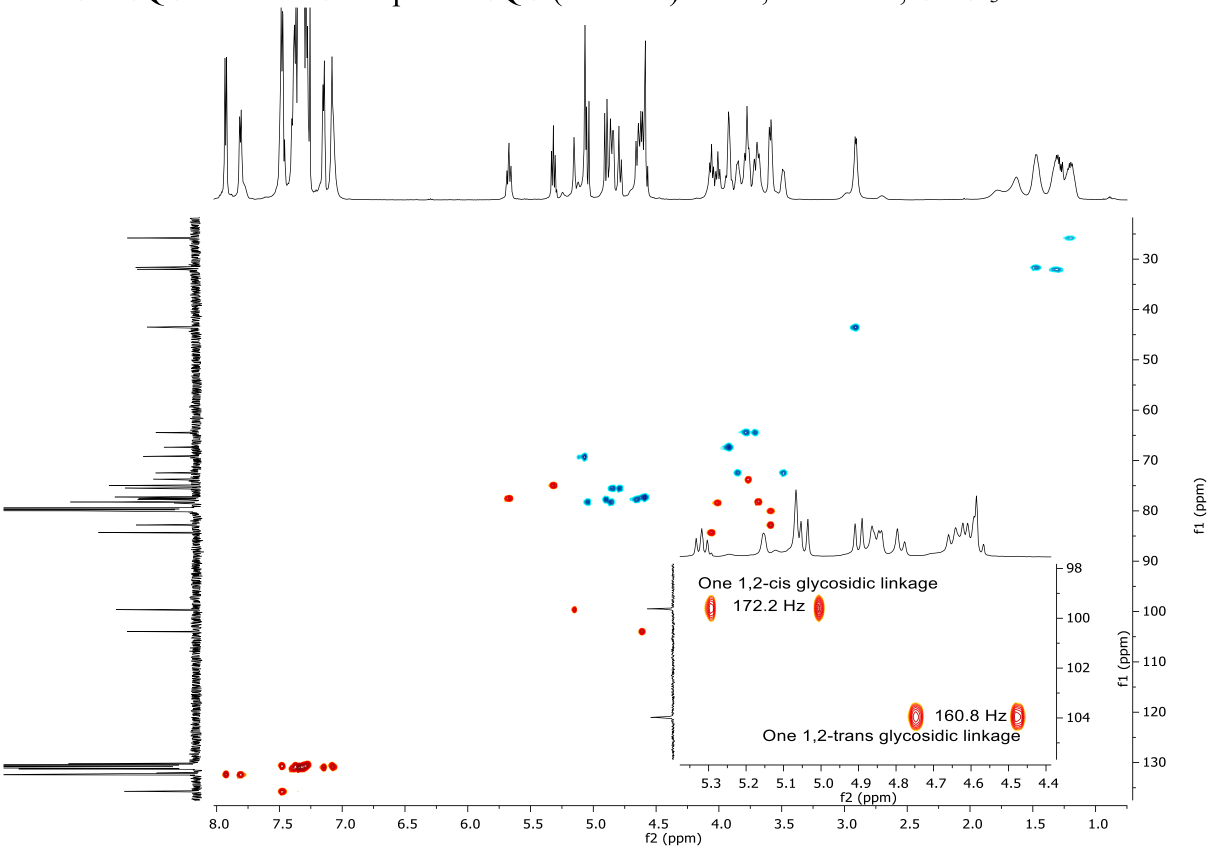

Supplementary Figure 96 | 2D NMR spectra of 30d

^{1}H NMR, 600 MHz, $CDCl_3$

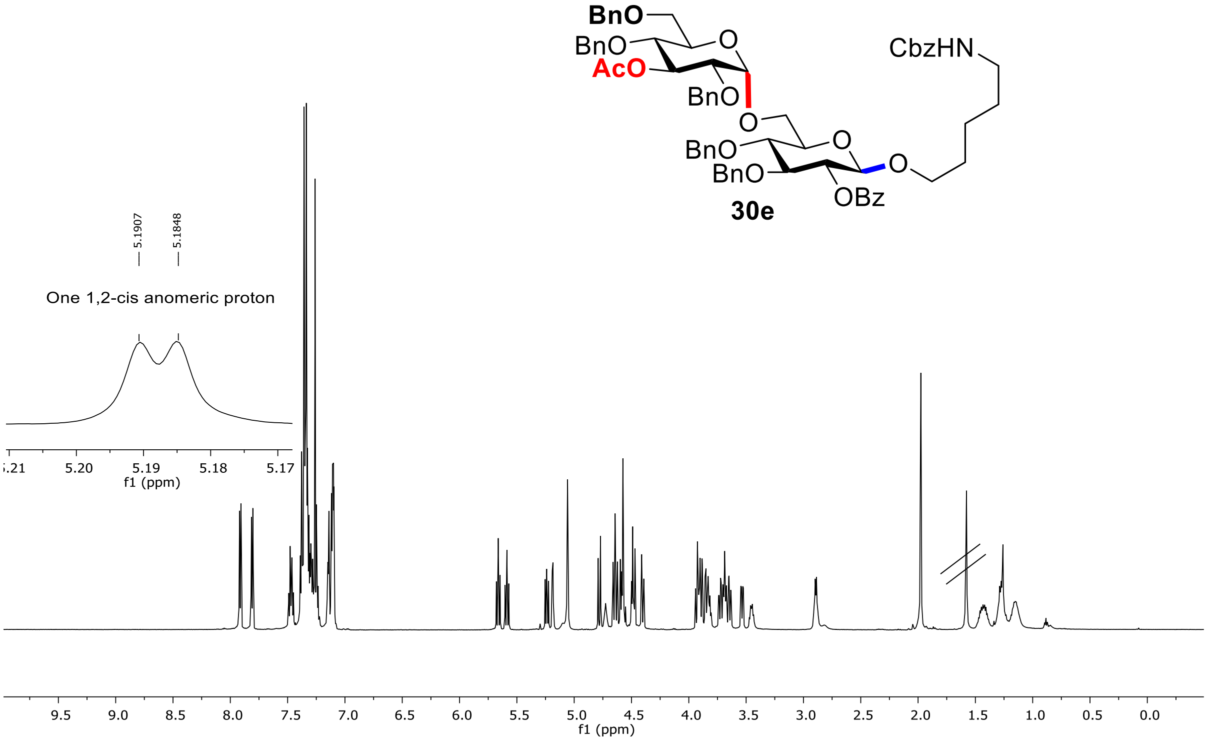

^{13}C NMR, 150 MHz, $CDCl_3$

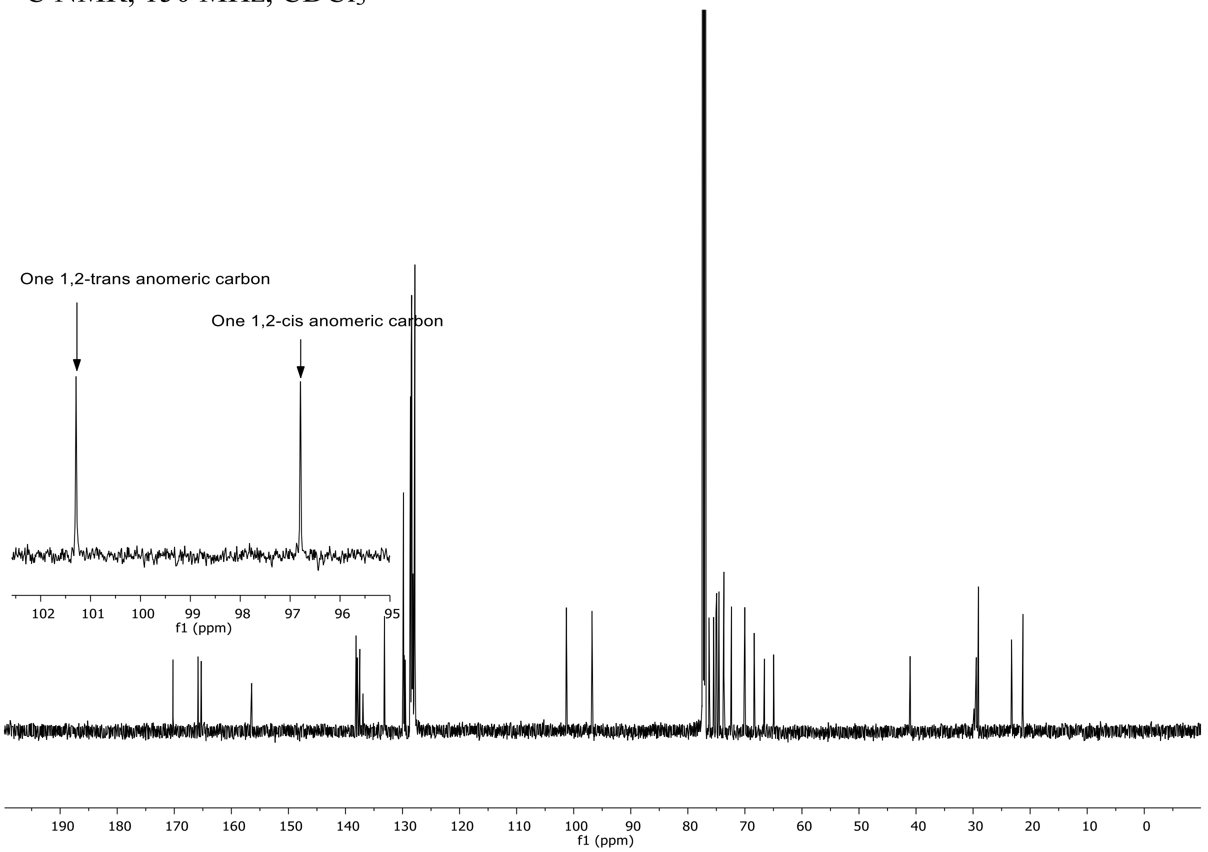

Supplementary Figure 97 | 1D NMR spectra of 30e

^{1}H-COSY NMR, 600 MHz, $CDCl_3$

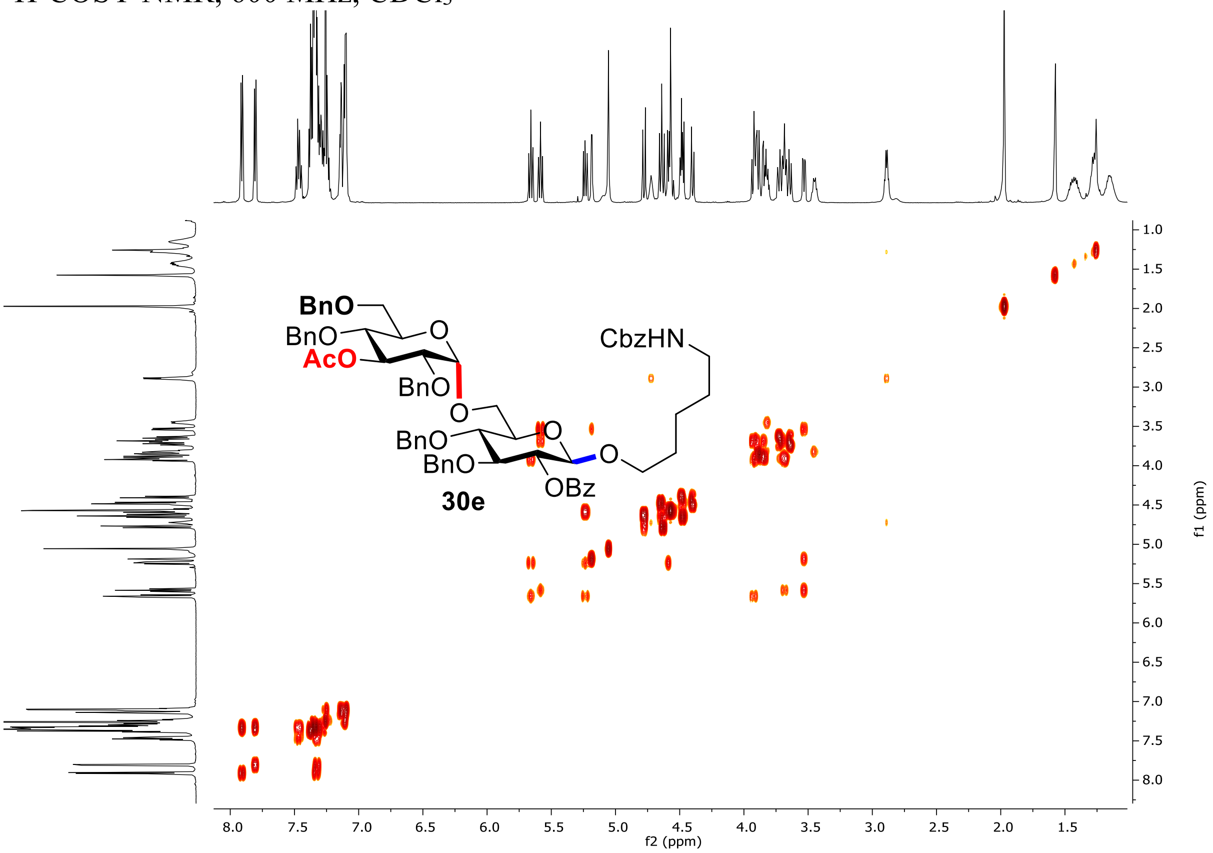

^{1}H-^{13}C-HSQC and ^{1}H-^{13}C-coupled-HSQC (zoom-in) NMR, 600 MHz, $CDCl_3$

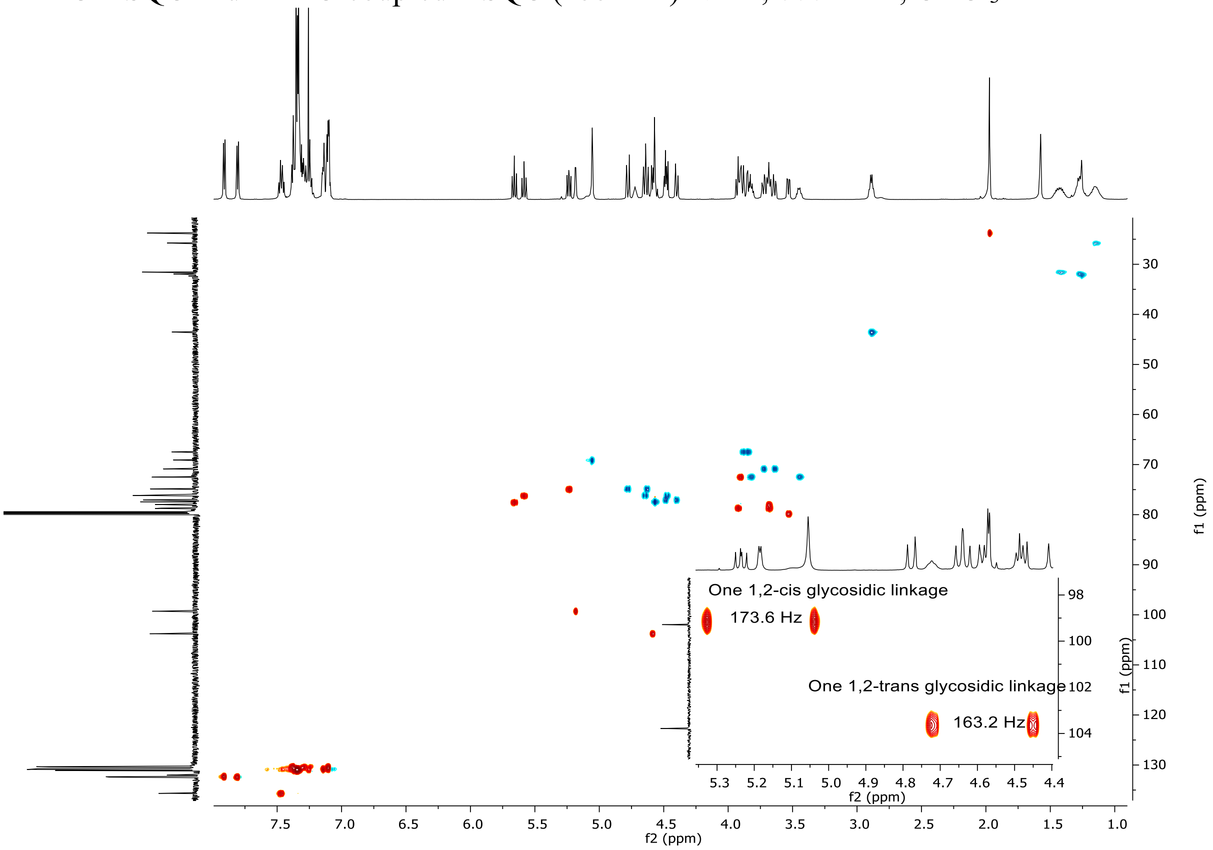

Supplementary Figure 98 | 2D NMR spectra of 30e

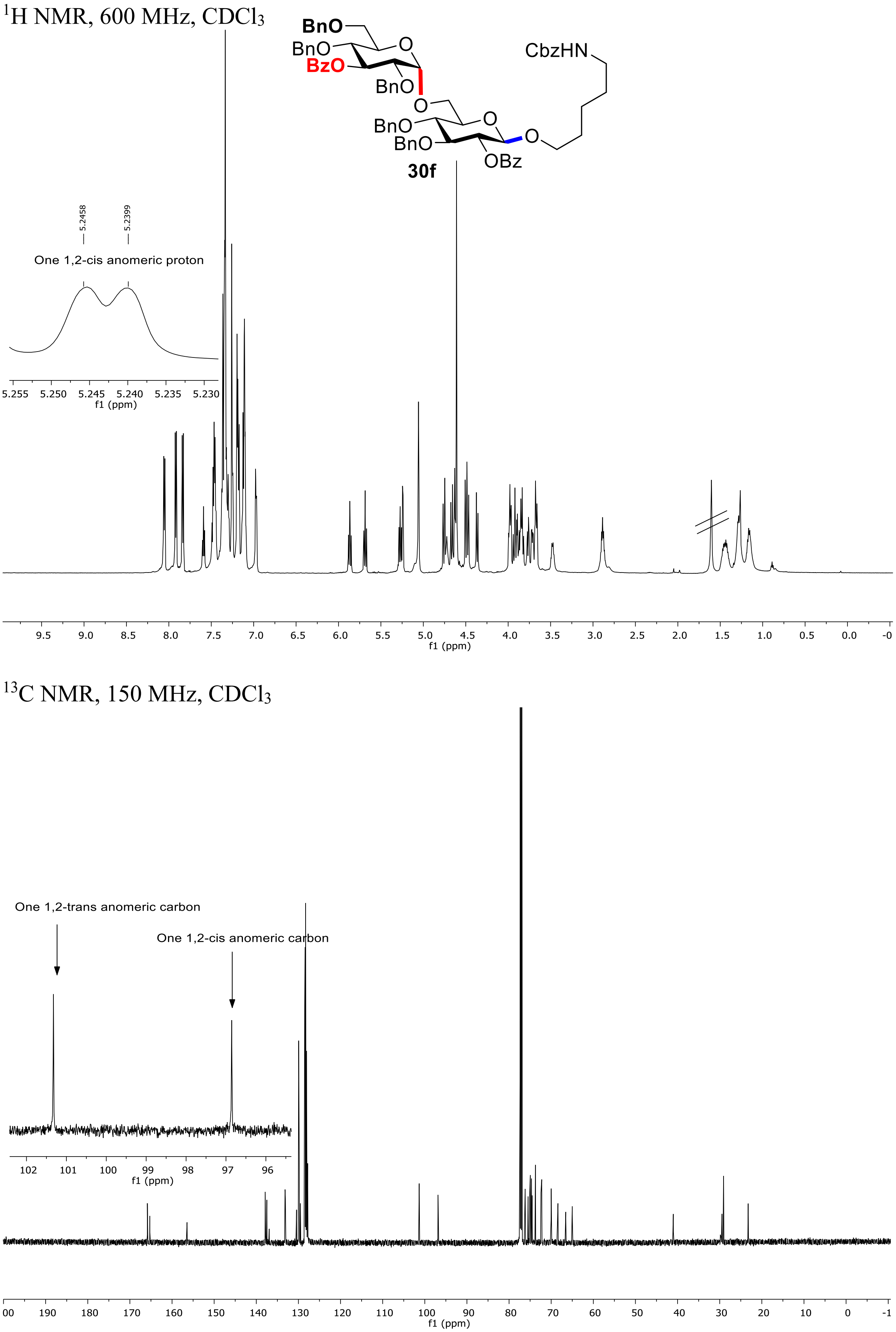

Supplementary Figure 99 | NMR spectra of 30f

^{1}H-COSY NMR, 600 MHz, $CDCl_3$

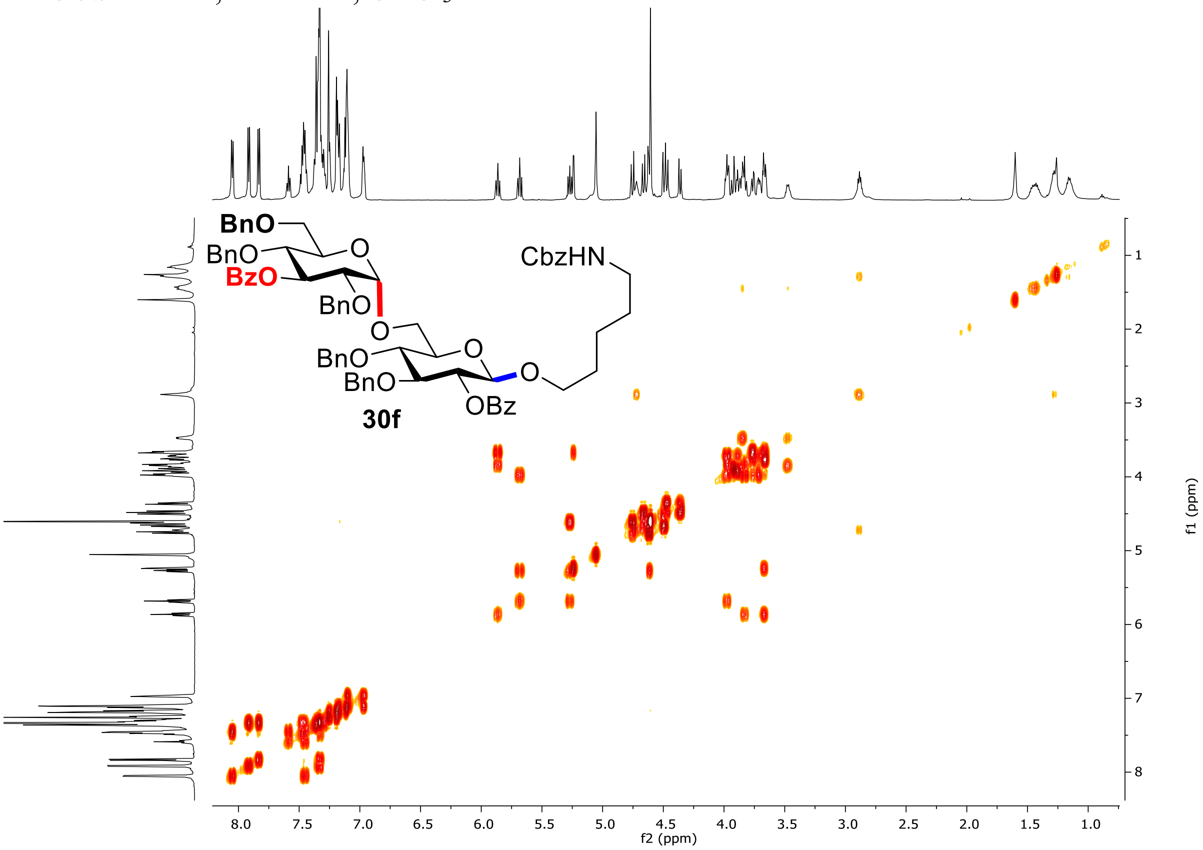

^{1}H-^{13}C-HSQC and ^{1}H-^{13}C-coupled-HSQC (zoom-in) NMR, 600 MHz, $CDCl_3$

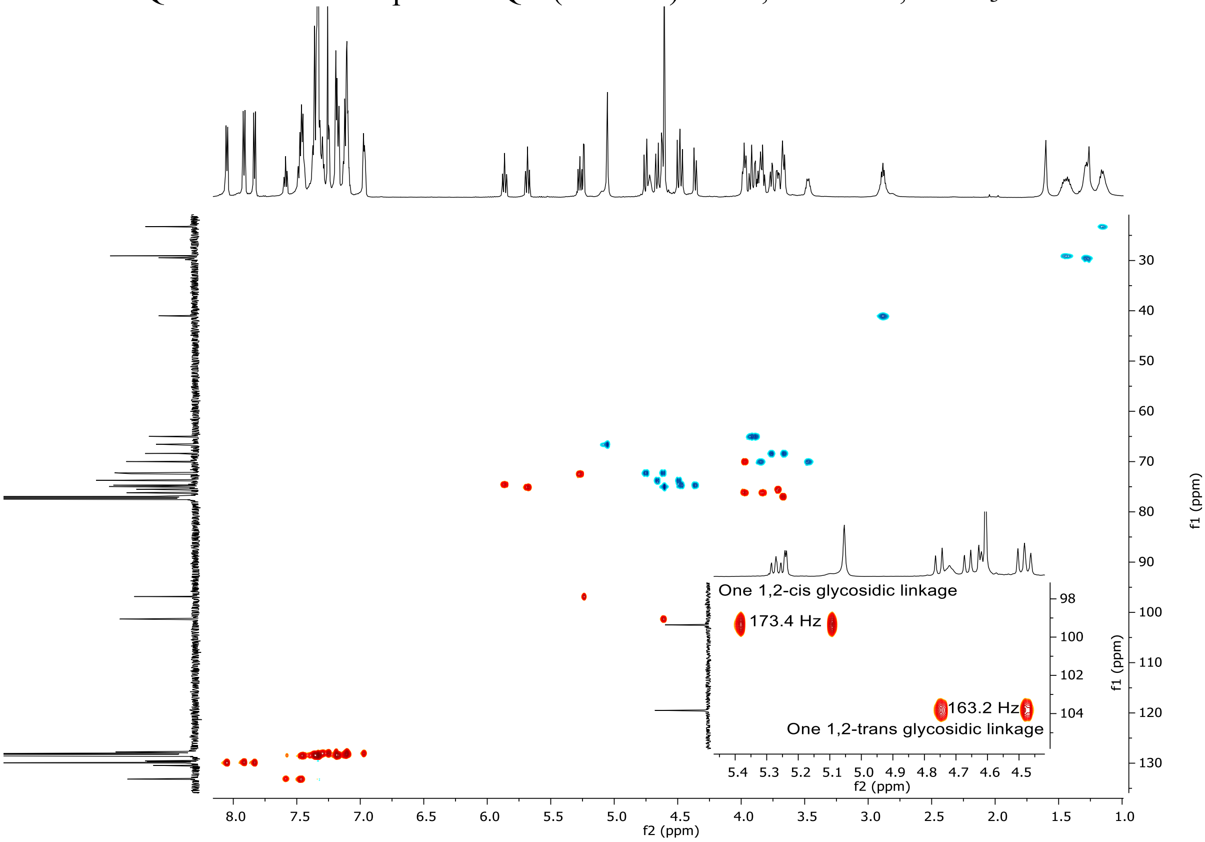

Supplementary Figure 100 | 2D NMR spectra of 30f

^{1}H NMR, 600 MHz, CDCl$_3$

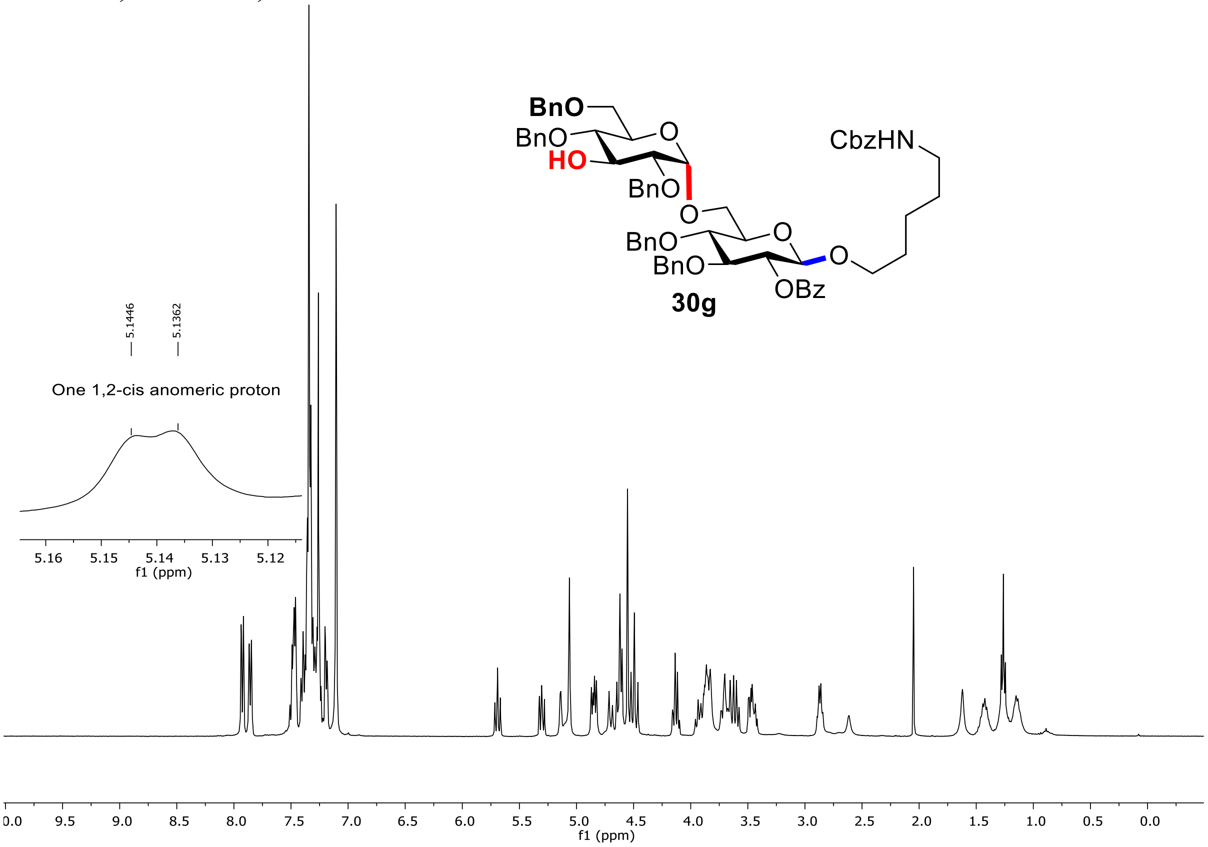

^{13}C NMR, 150 MHz, CDCl$_3$

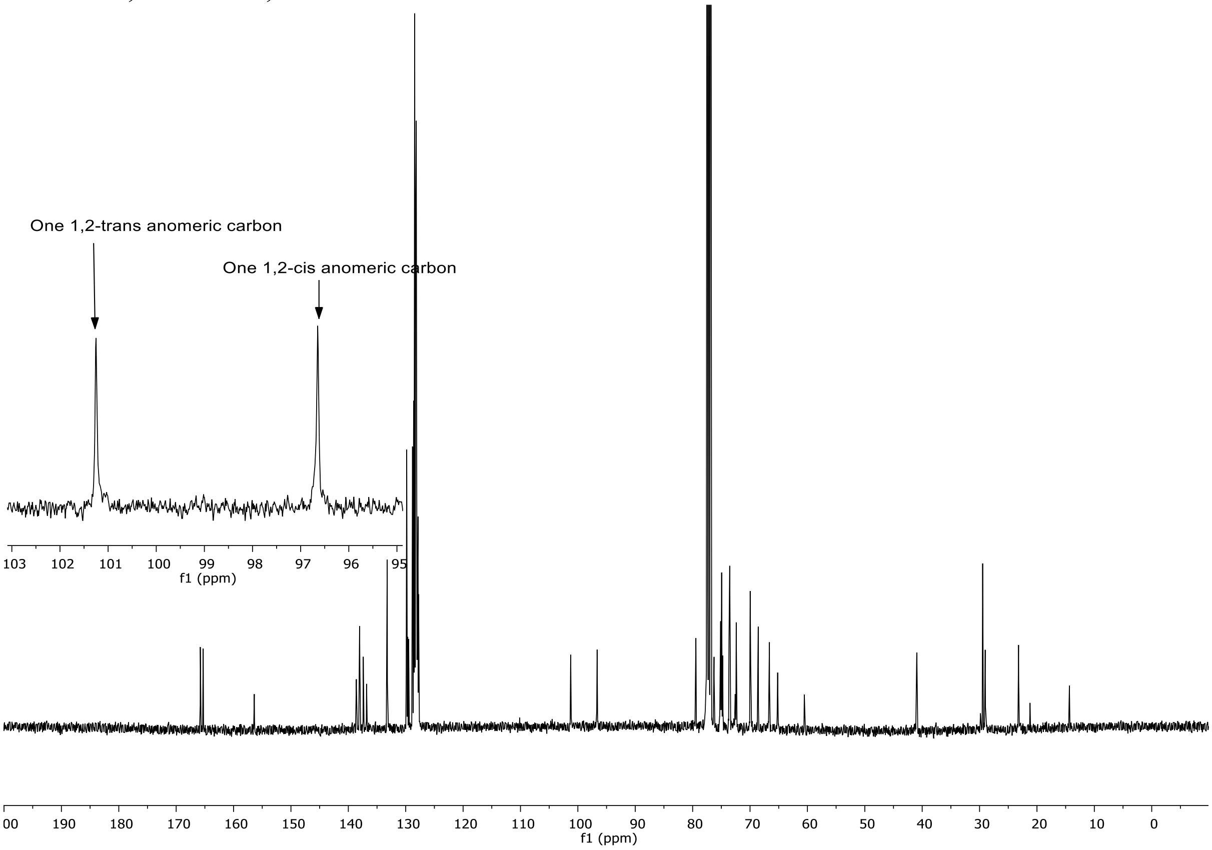

Supplementary Figure 101 | 1D NMR spectra of 30g

^{1}H-COSY NMR, 600 MHz, $CDCl_3$

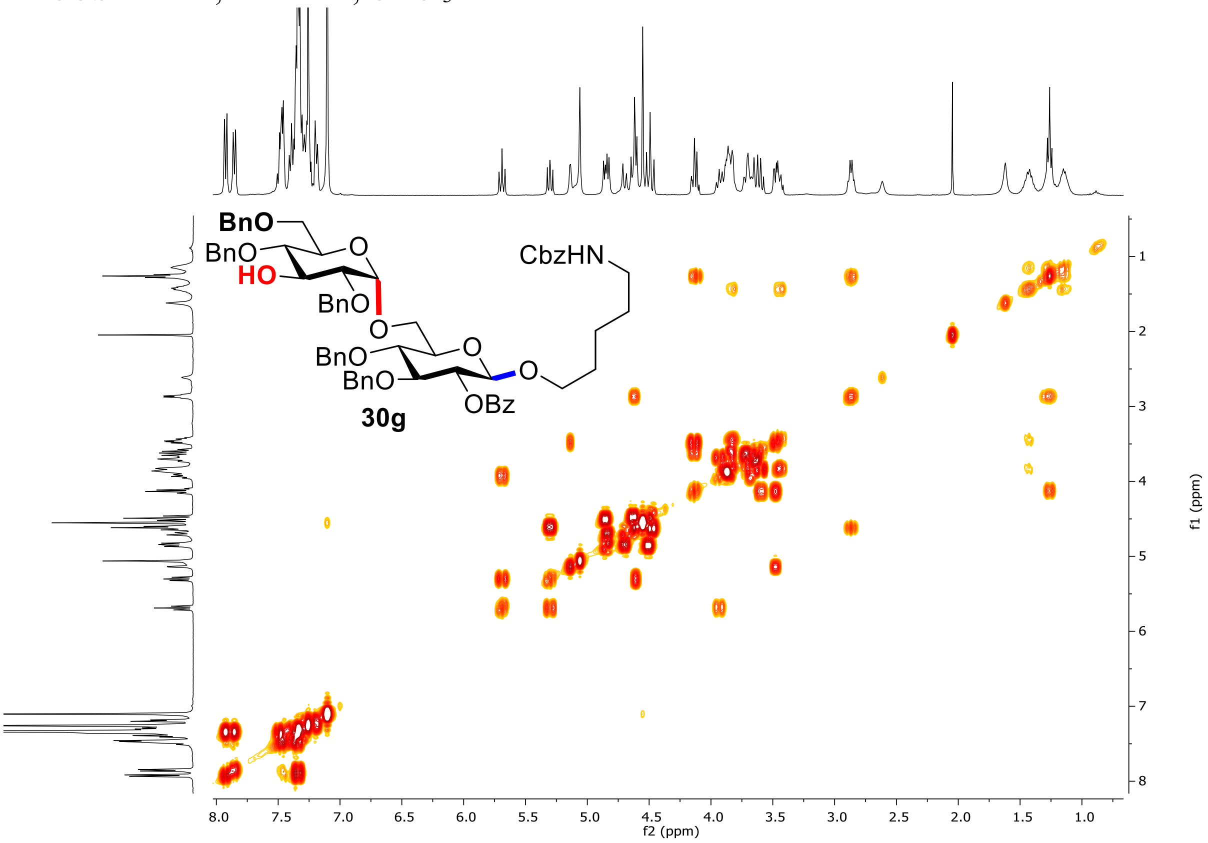

^{1}H-^{13}C-HSQC and ^{1}H-^{13}C-coupled-HSQC (zoom-in) NMR, 600 MHz, $CDCl_3$

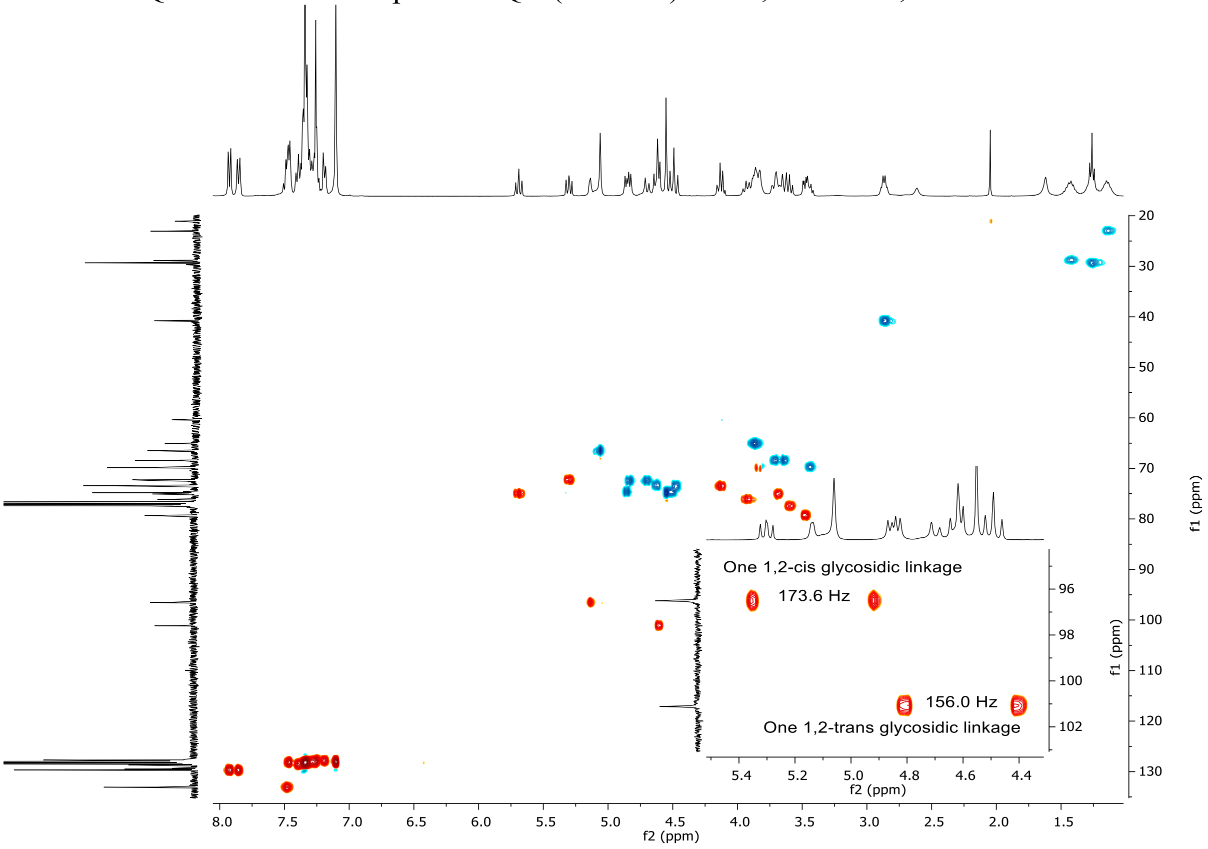

Supplementary Figure 102 | 2D NMR spectra of 30g

^{1}H NMR, 600 MHz, $CDCl_3$

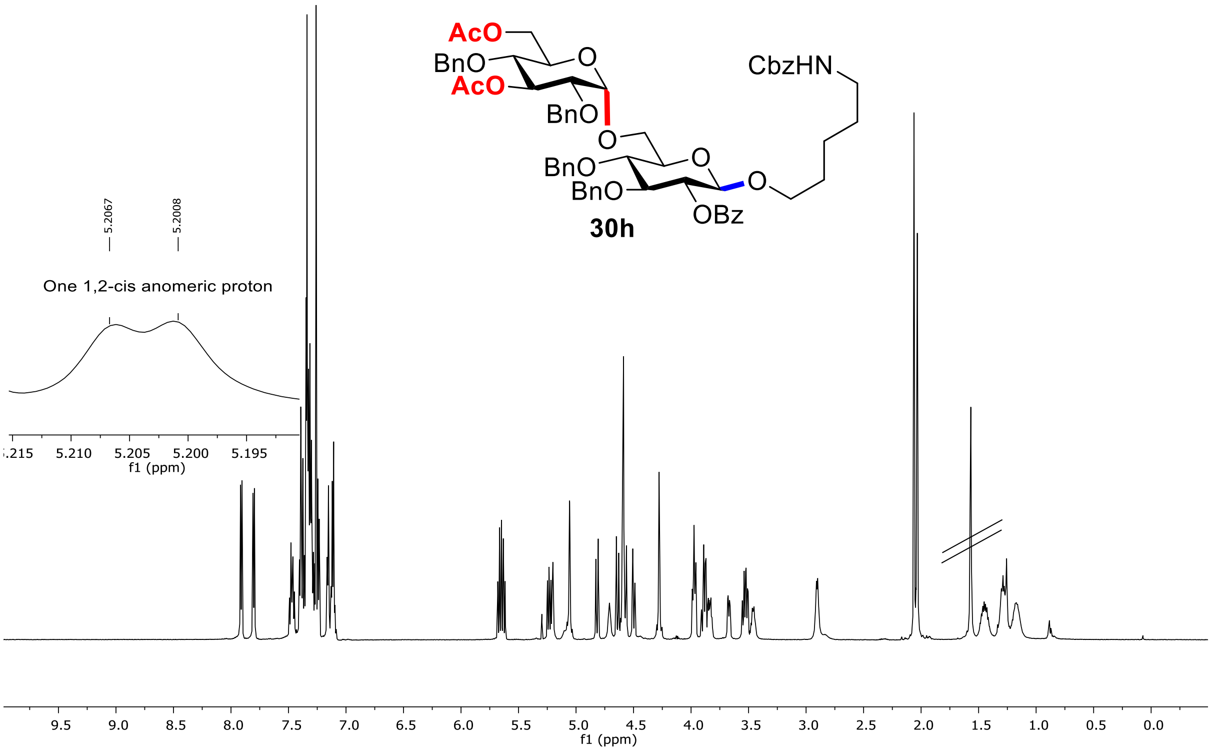

^{13}C NMR, 150 MHz, $CDCl_3$

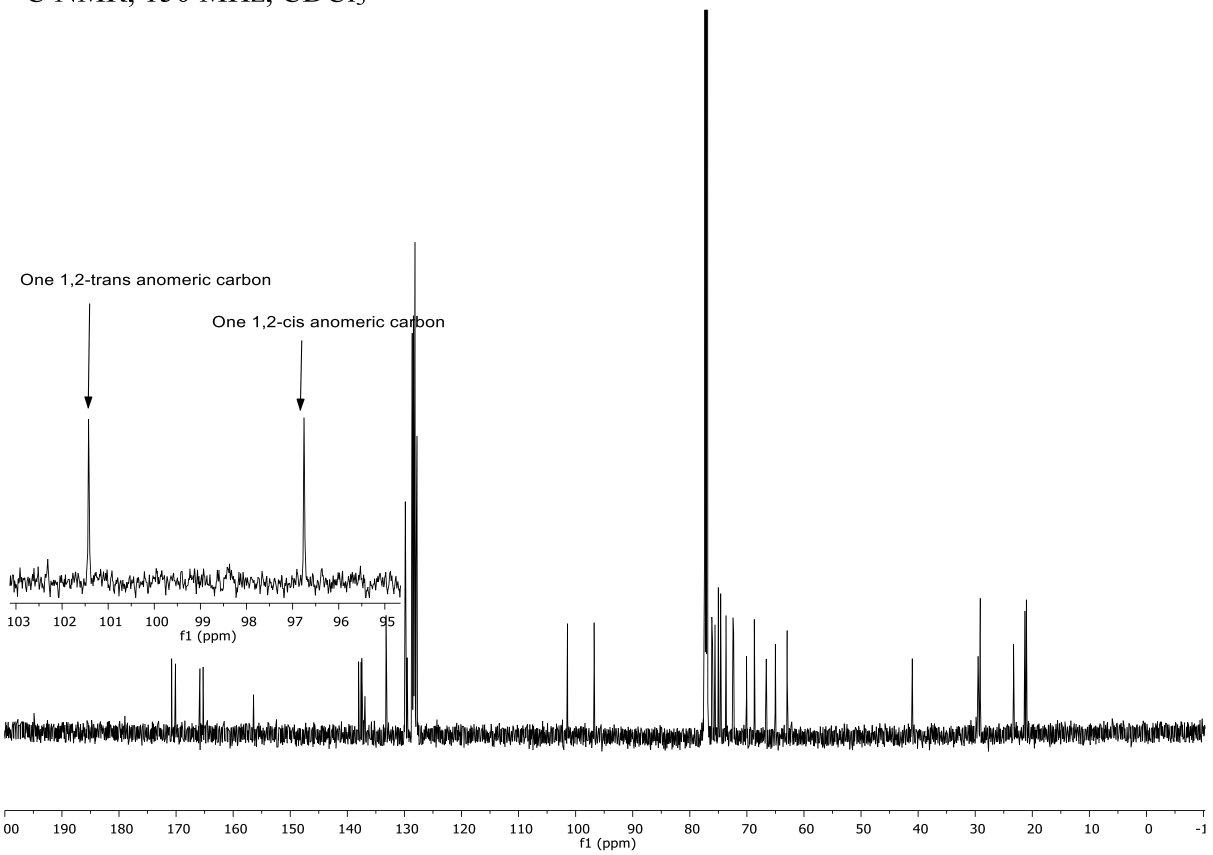

Supplementary Figure 103 | 1D NMR spectra of 30h

^{1}H-COSY NMR, 600 MHz, $CDCl_3$

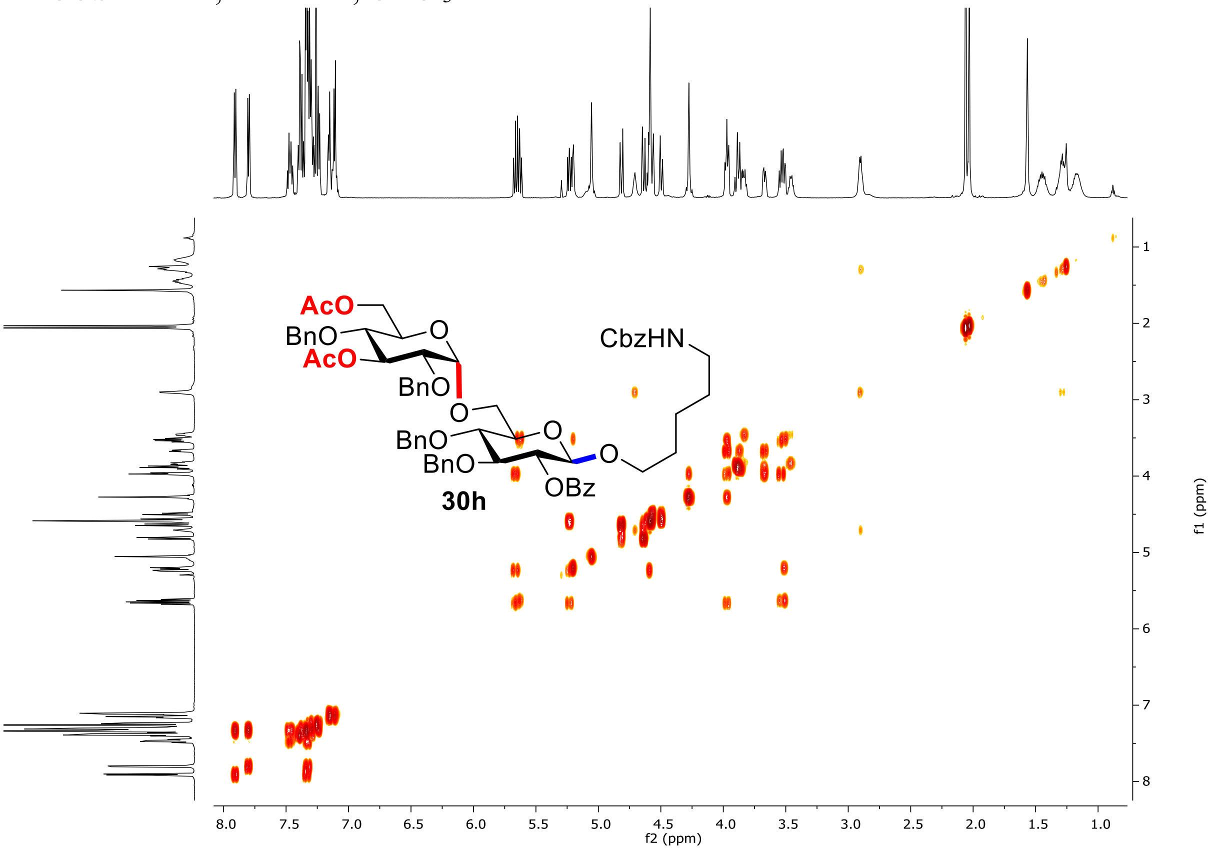

^{1}H-^{13}C-HSQC and ^{1}H-^{13}C-coupled-HSQC (zoom-in) NMR, 600 MHz, $CDCl_3$

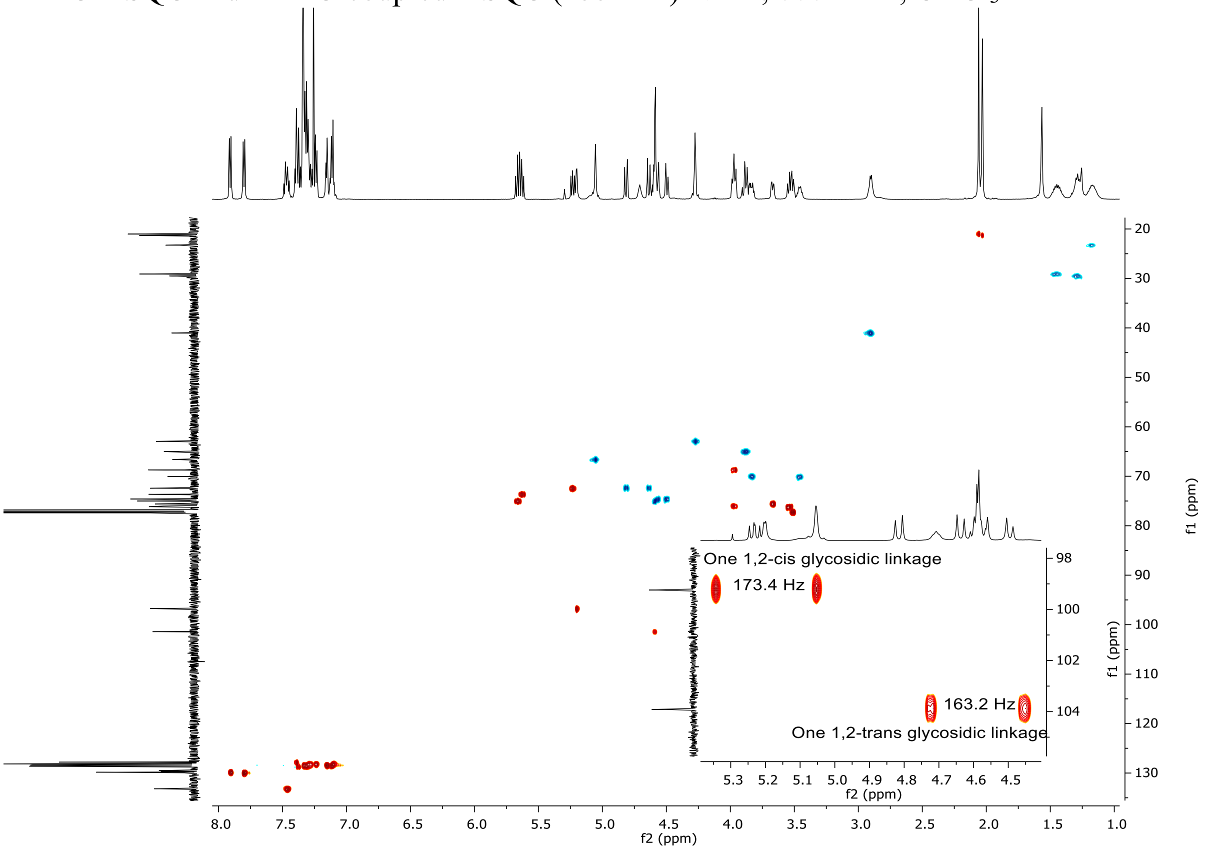

Supplementary Figure 104 | 2D NMR spectra of 30h

^{1}H NMR, 400 MHz, $CDCl_3$

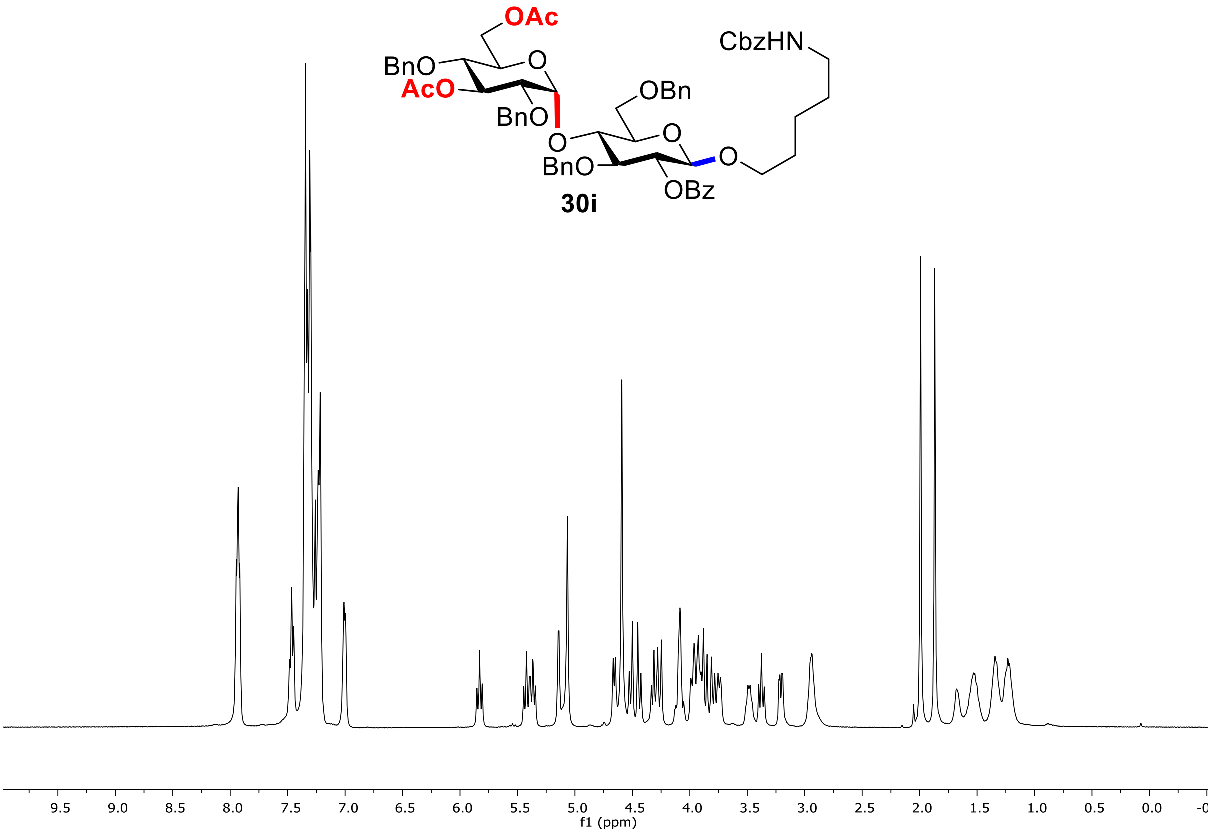

^{13}C NMR, 100 MHz, $CDCl_3$

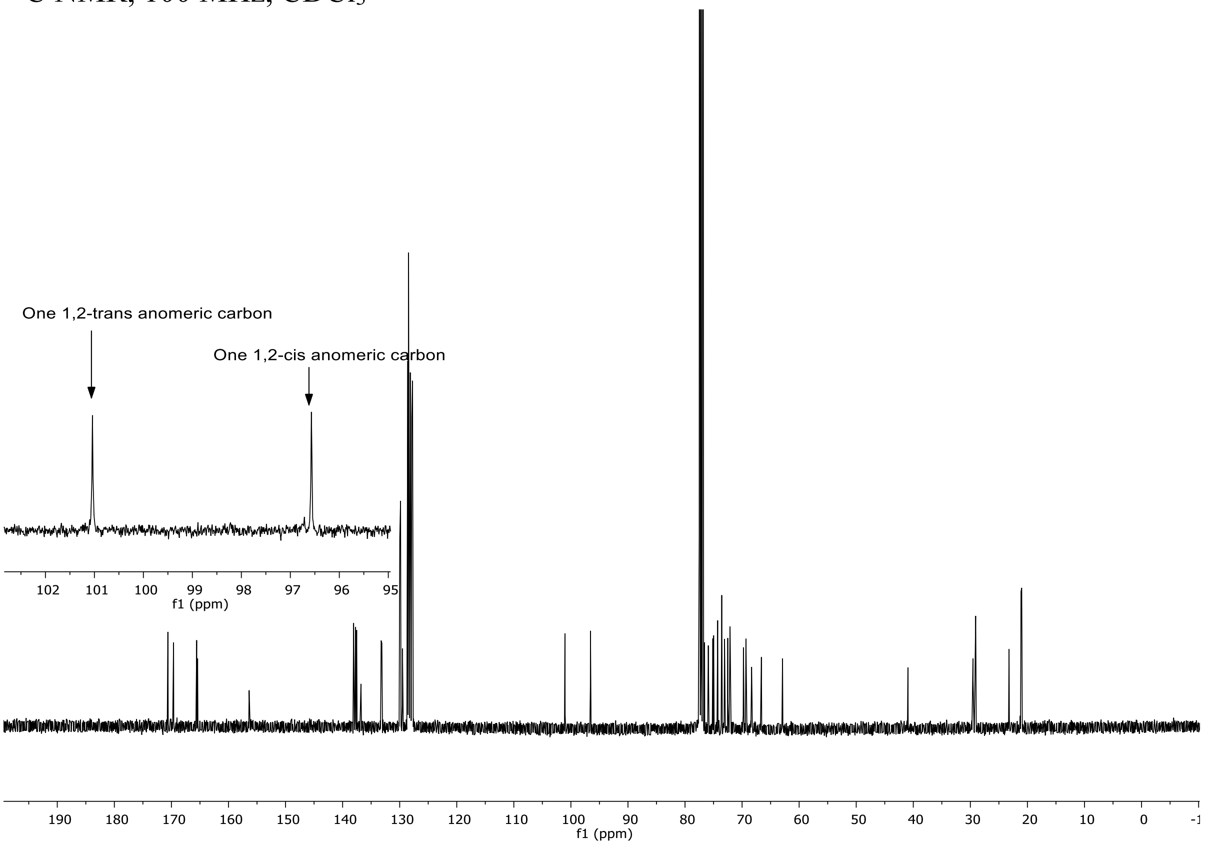

Supplementary Figure 105 | 1D NMR spectra of 30i

^{1}H-COSY NMR, 400 MHz, $CDCl_3$

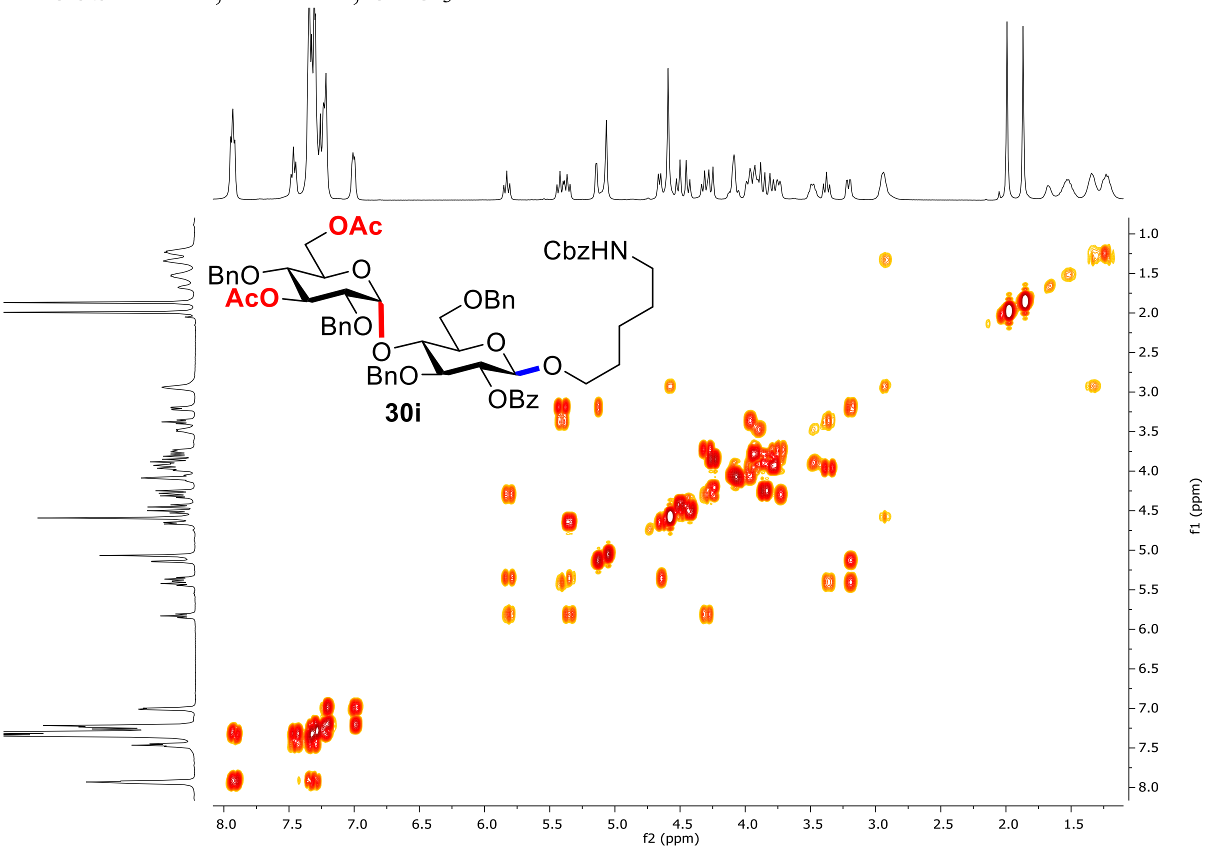

^{1}H-^{13}C-HSQC and ^{1}H-^{13}C-coupled-HSQC (zoom-in) NMR, 400 MHz, $CDCl_3$

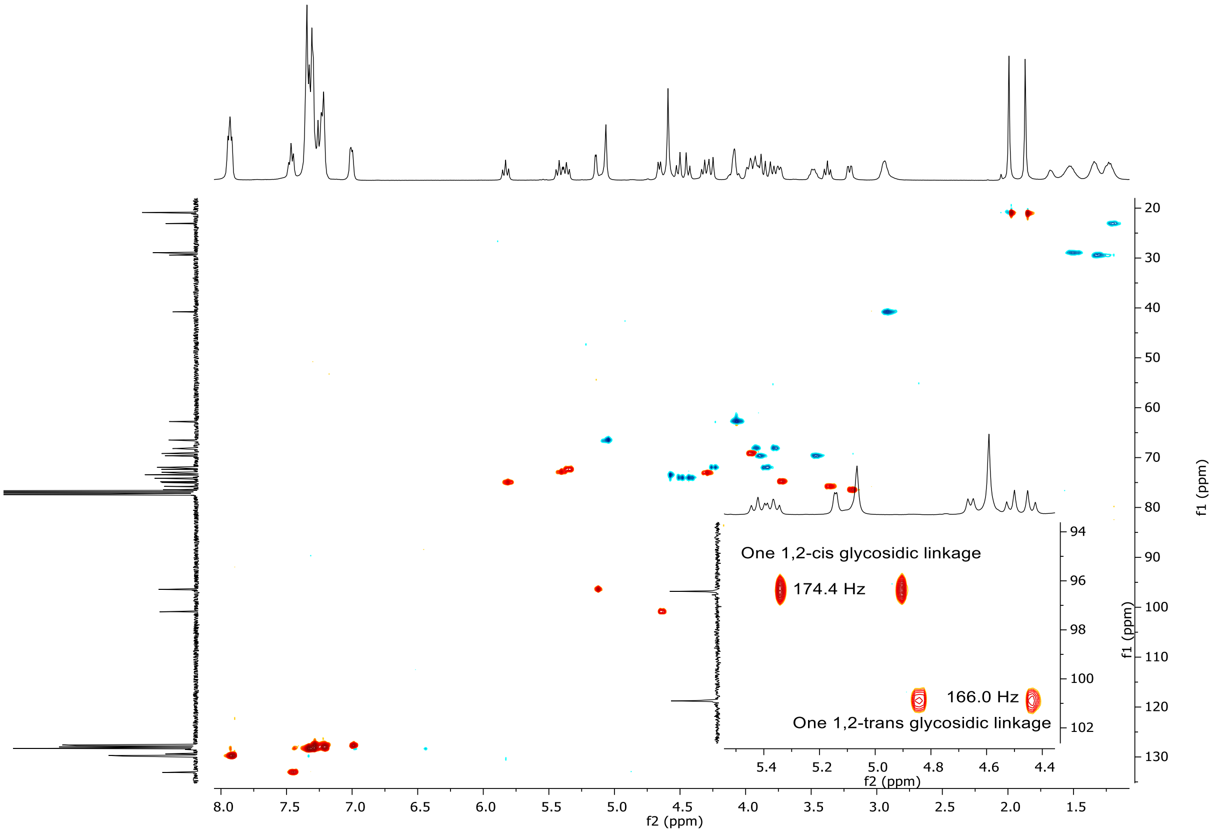

Supplementary Figure 106 | 2D NMR spectra of 30i

^{1}H NMR, 400 MHz, $CDCl_3$

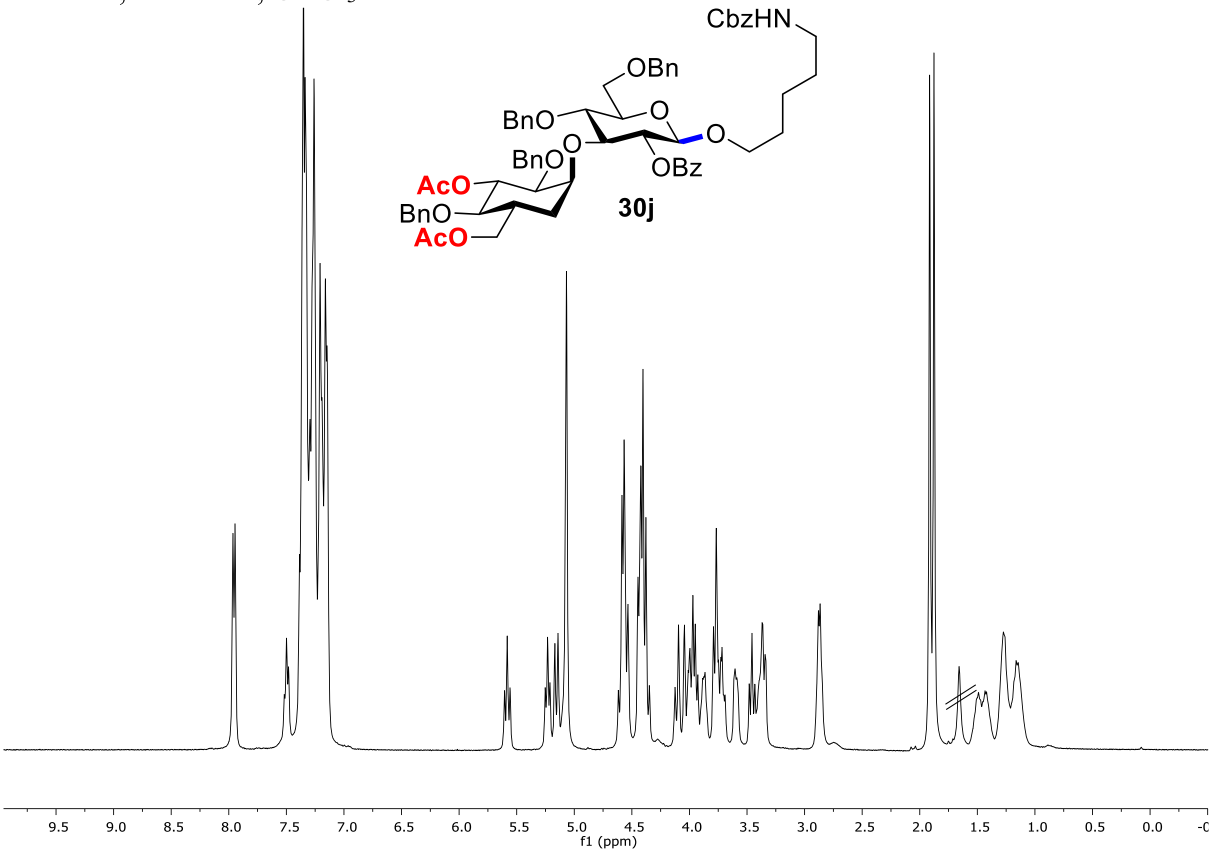

^{13}C NMR, 100 MHz, $CDCl_3$

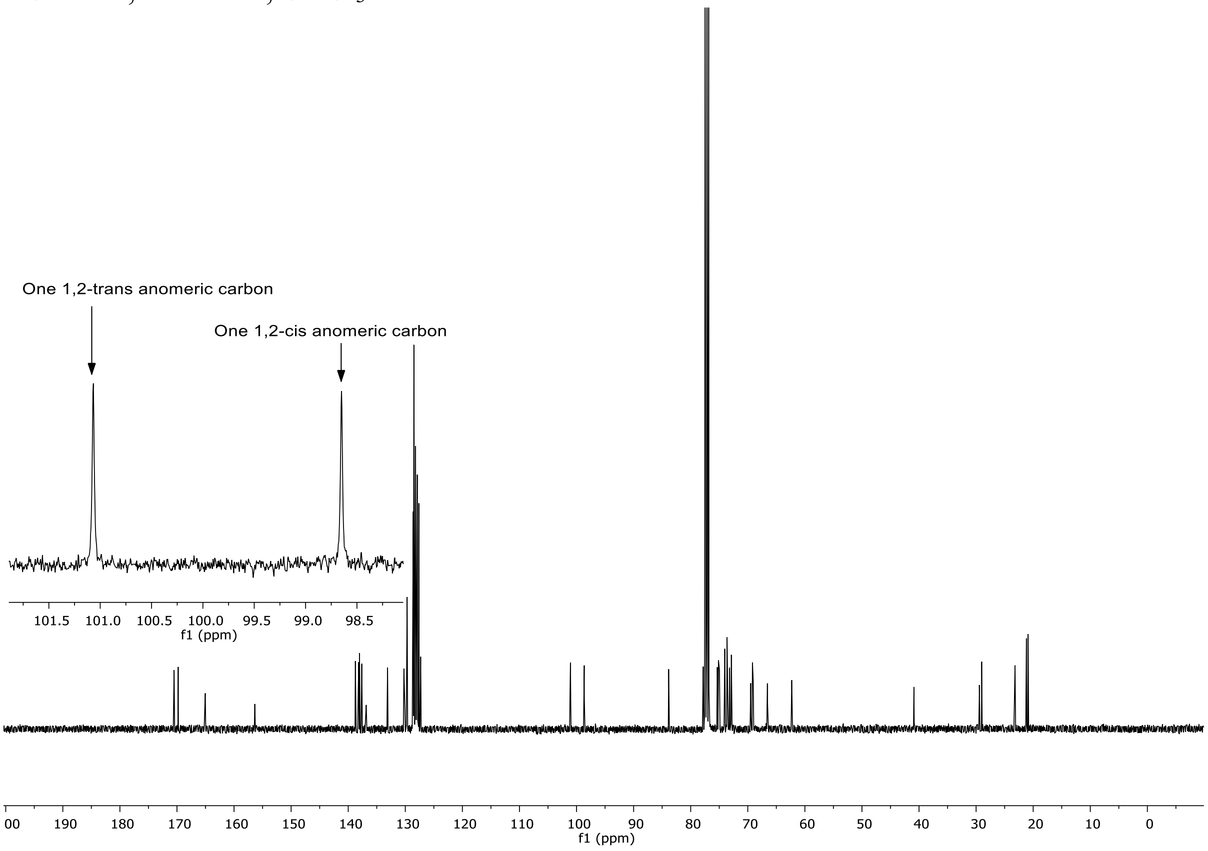

Supplementary Figure 107 | 1D NMR spectra of 30j

^{1}H-COSY NMR, 600 MHz, CDCl$_3$

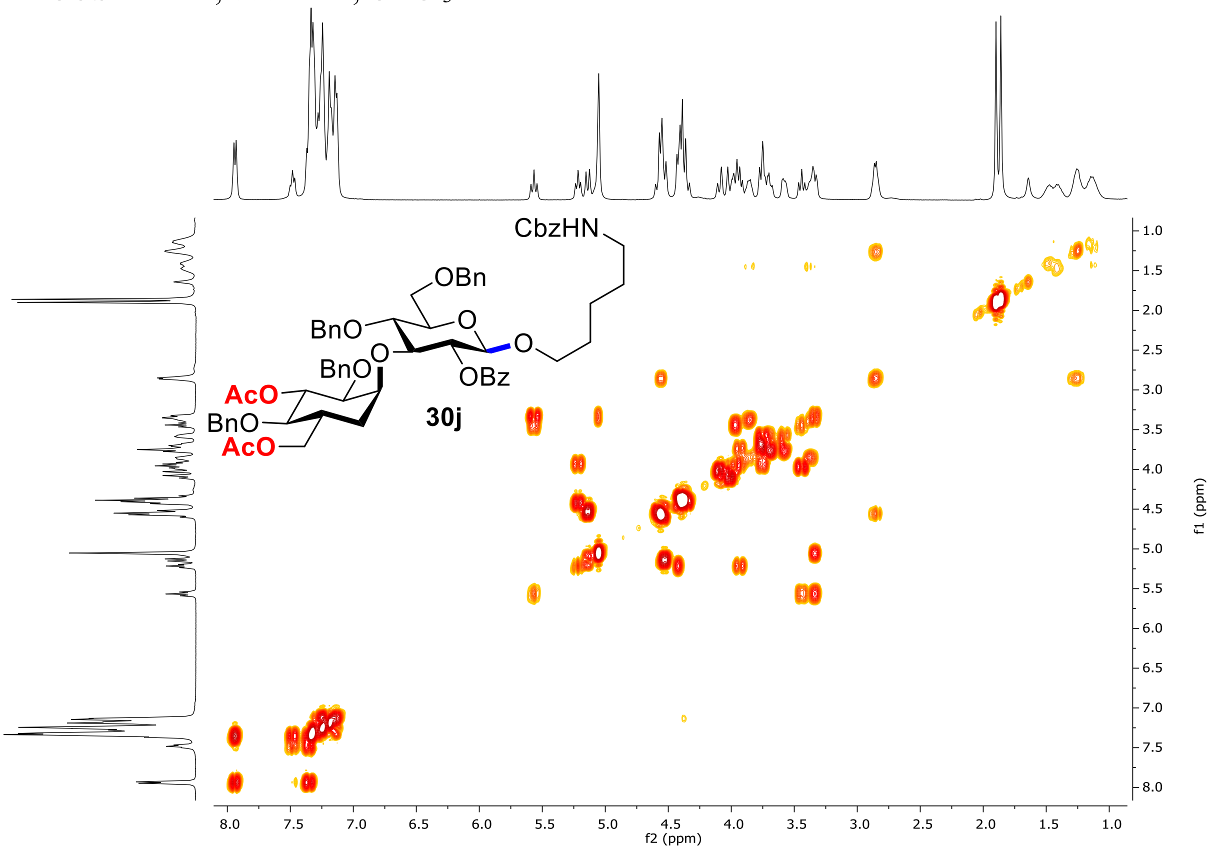

^{1}H-^{13}C-HSQC and ^{1}H-^{13}C-coupled-HSQC (zoom-in) NMR, 600 MHz, CDCl$_3$

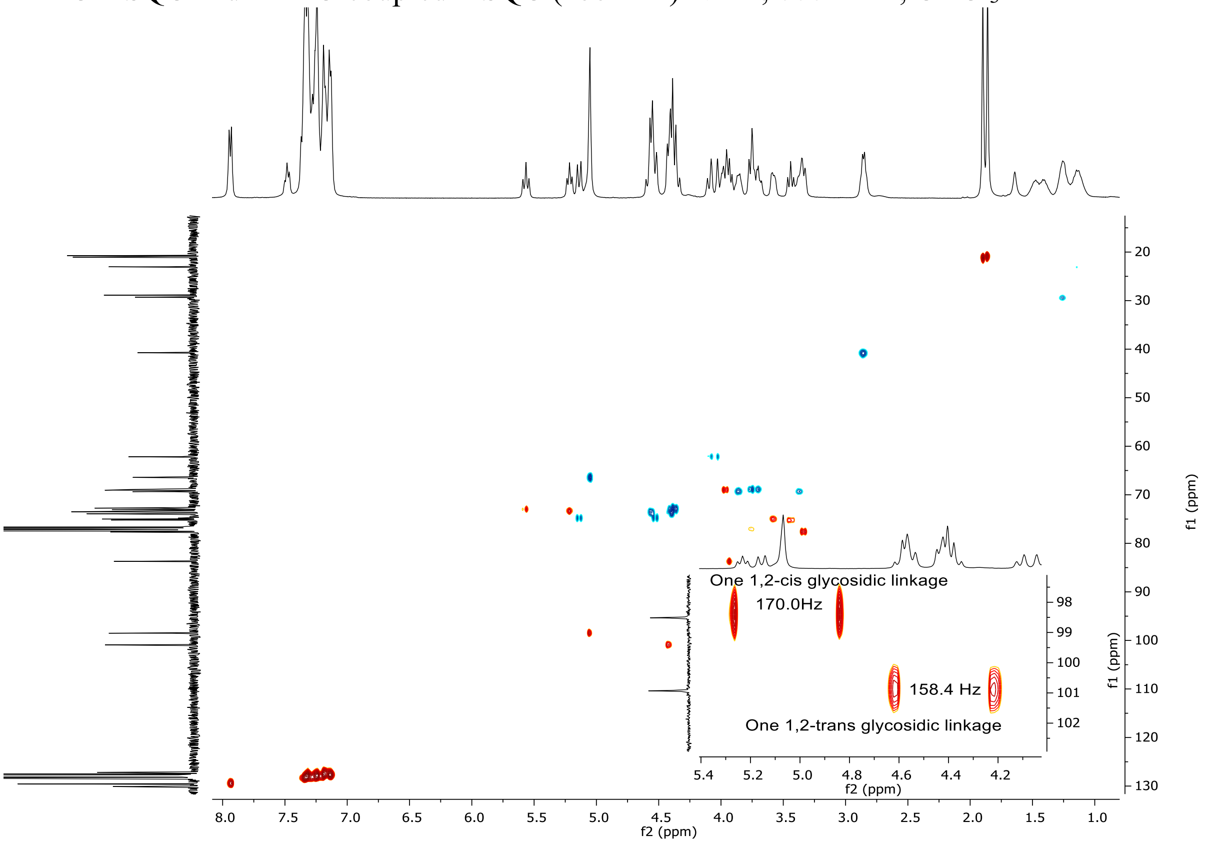

Supplementary Figure 108 | 2D NMR spectra of 30j

^{1}H NMR, 600 MHz, $CDCl_3$

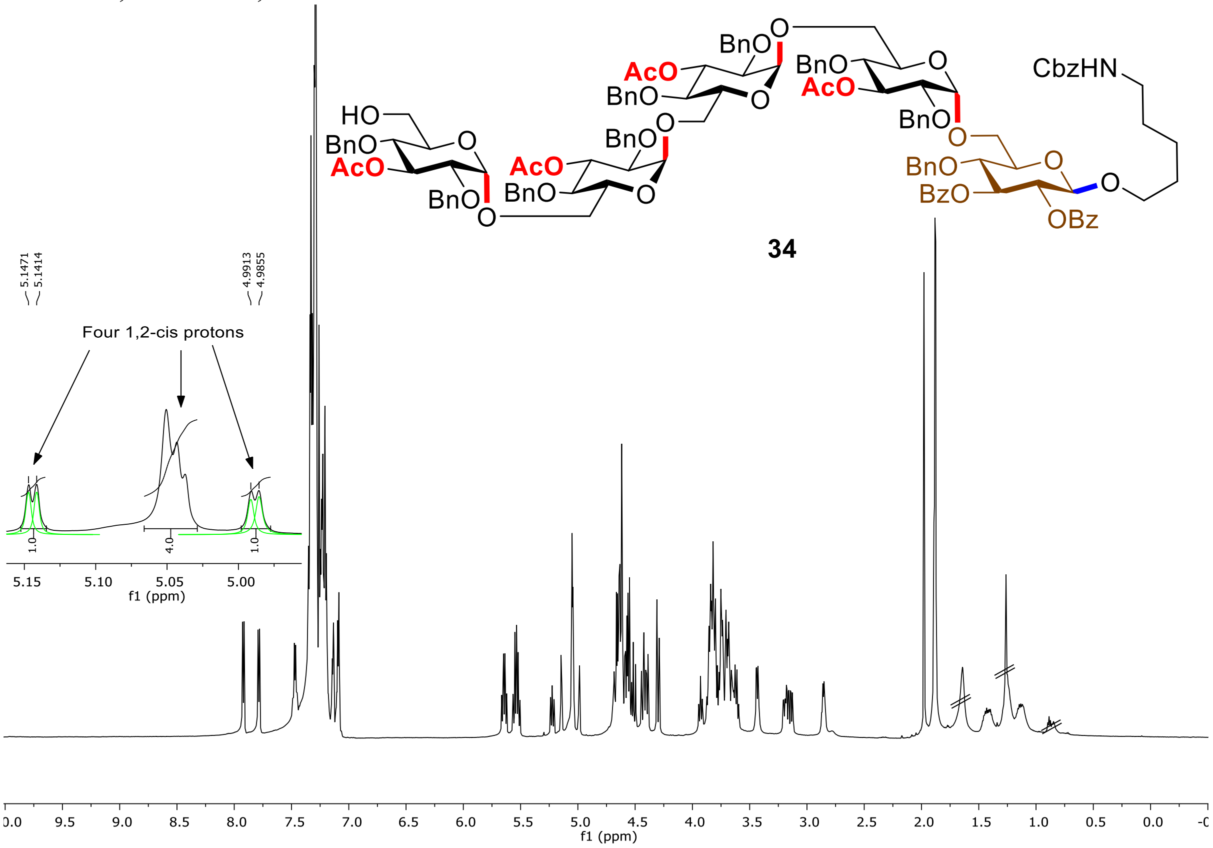

^{13}C NMR, 150 MHz, $CDCl_3$

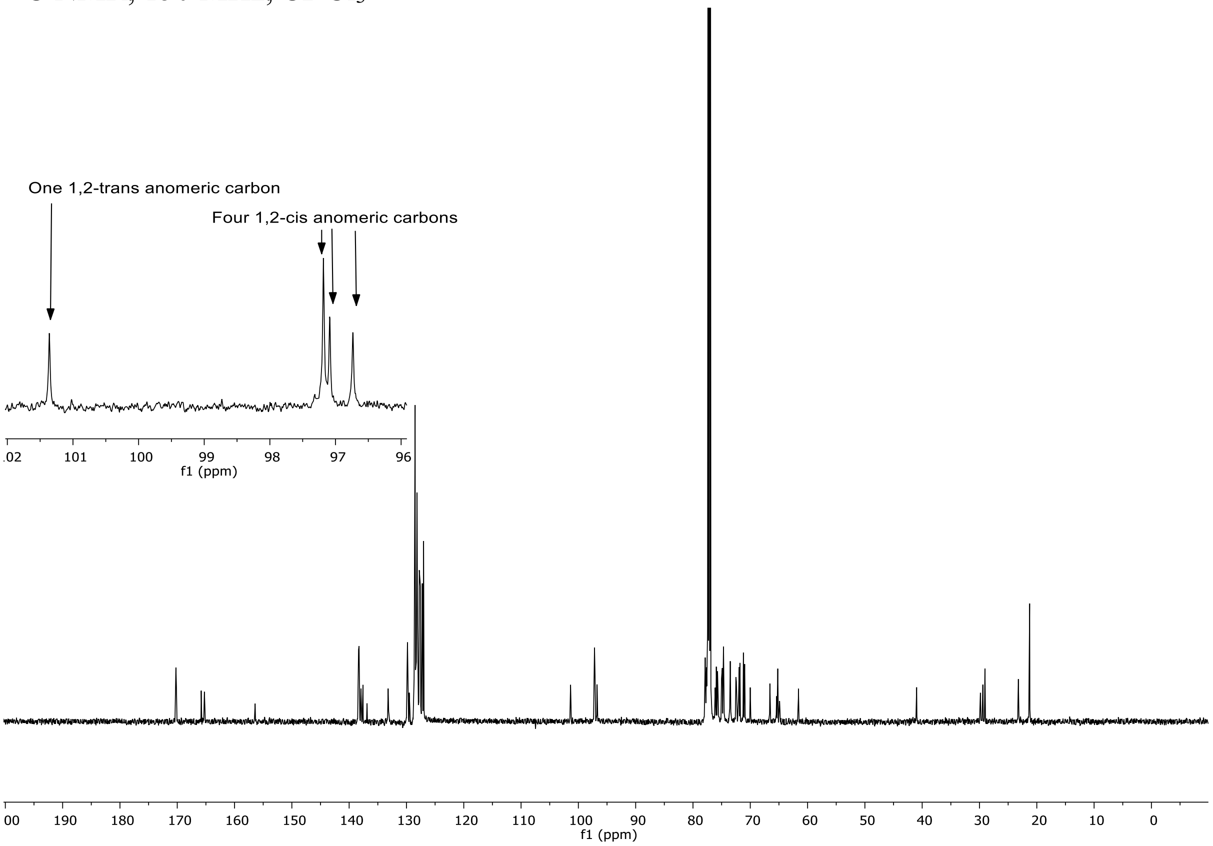

Supplementary Figure 109 | 1D NMR spectra of 34

^{1}H-COSY NMR, 600 MHz, $CDCl_3$

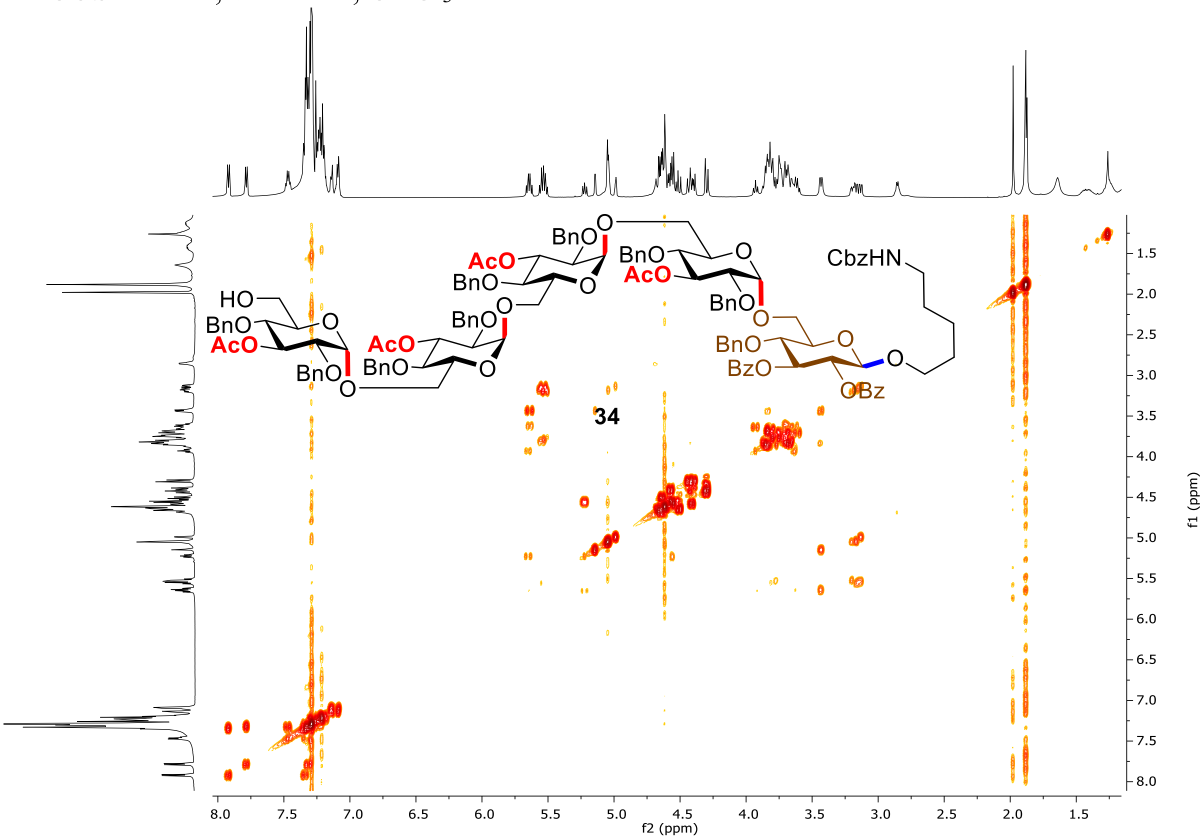

^{1}H-^{13}C-HSQC and ^{1}H-^{13}C-coupled-HSQC (zoom-in) NMR, 600 MHz, $CDCl_3$

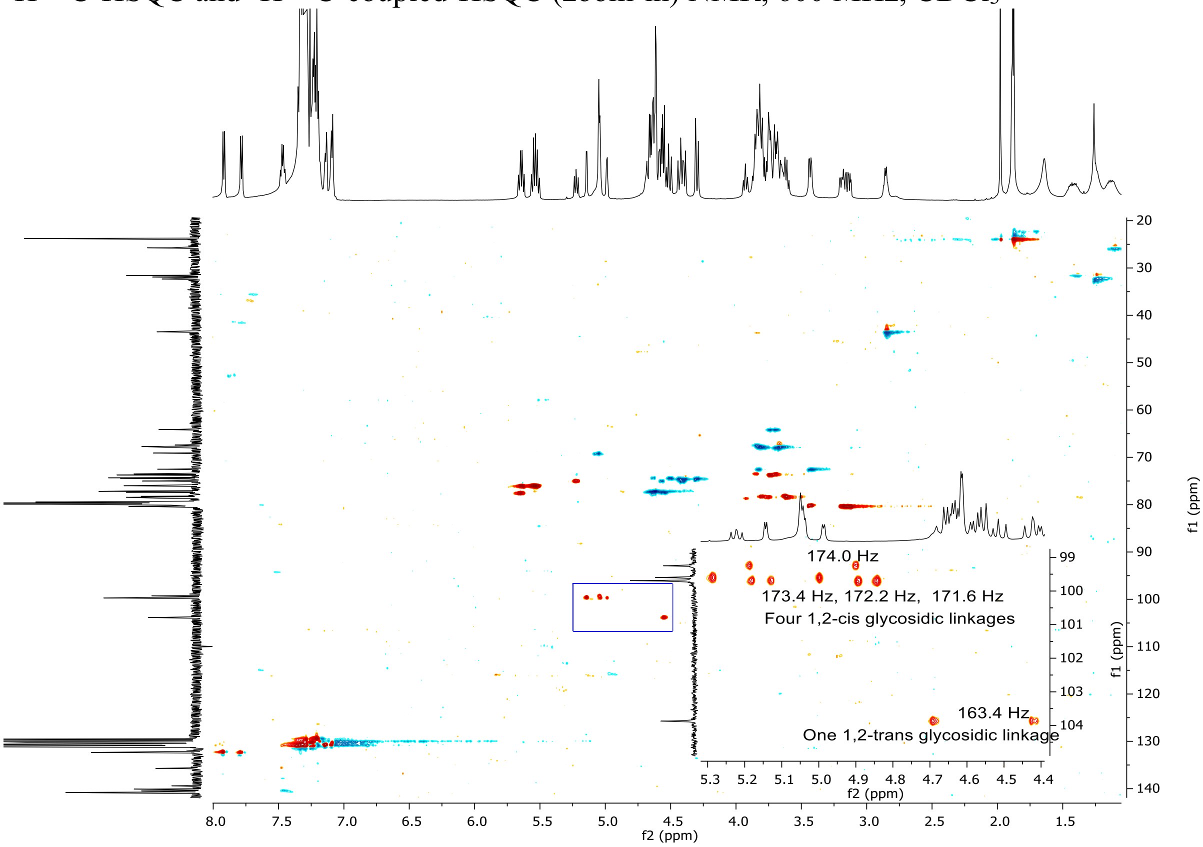

Supplementary Figure 110 | 2D NMR spectra of 34

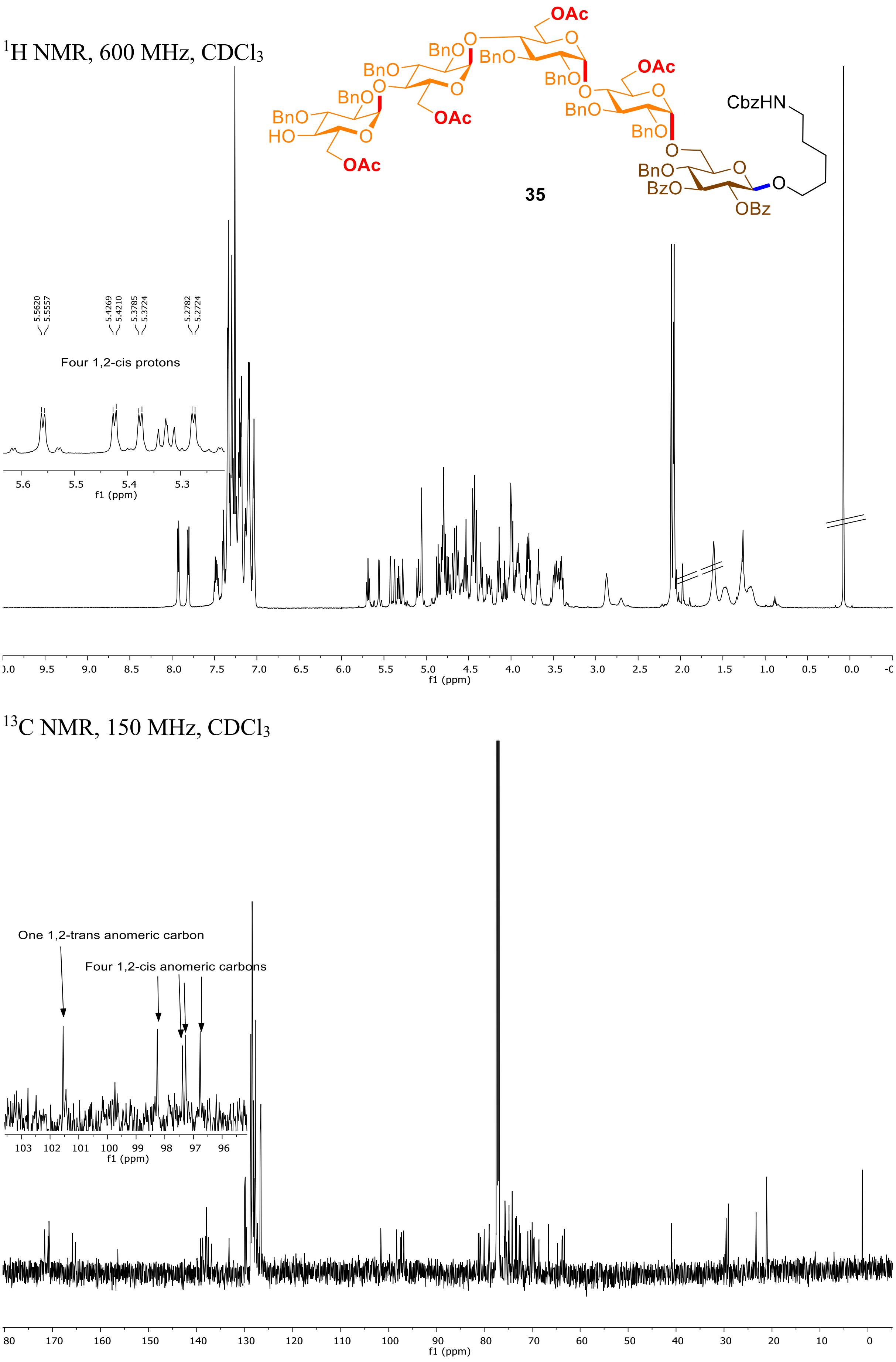

Supplementary Figure 111 | 1D NMR spectra of 35

^{1}H-COSY NMR, 600 MHz, $CDCl_3$

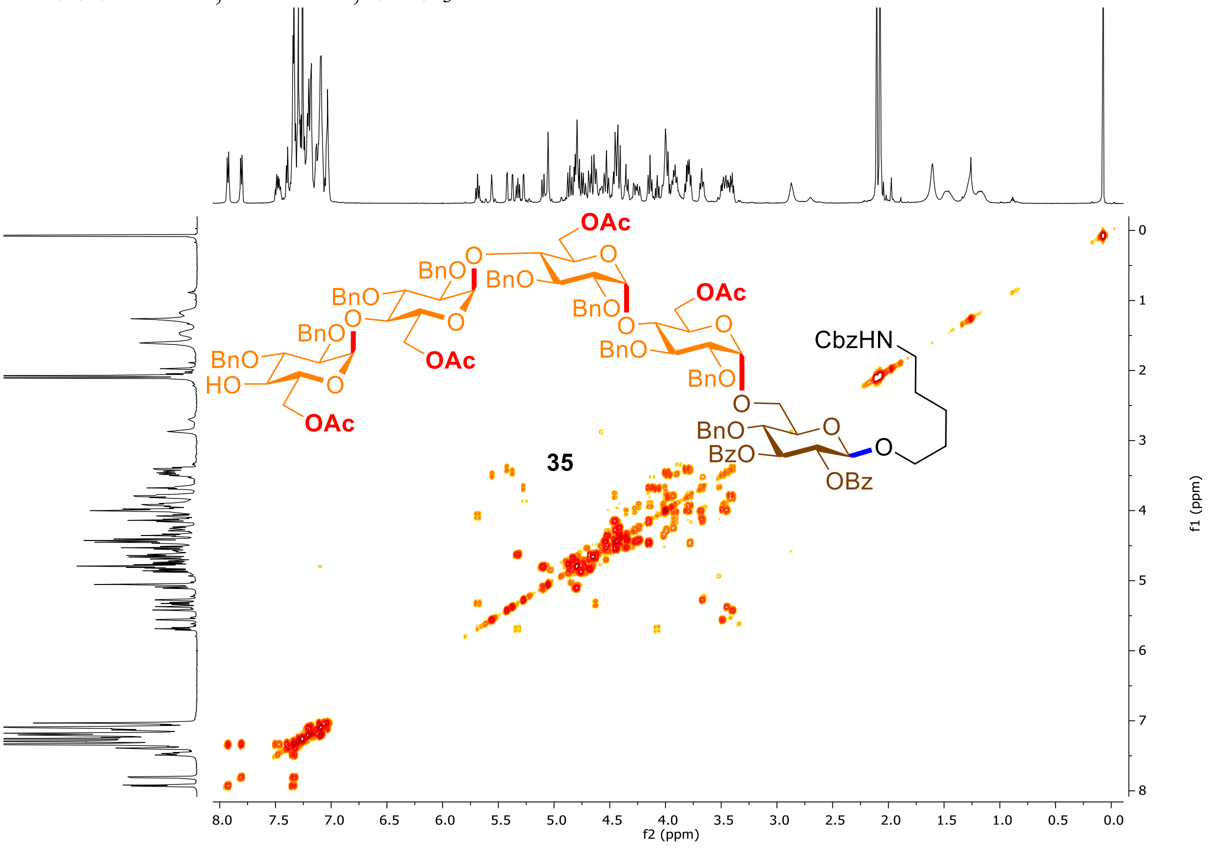

^{1}H-^{13}C-HSQC and ^{1}H-^{13}C-coupled-HSQC (zoom-in) NMR, 600 MHz, $CDCl_3$

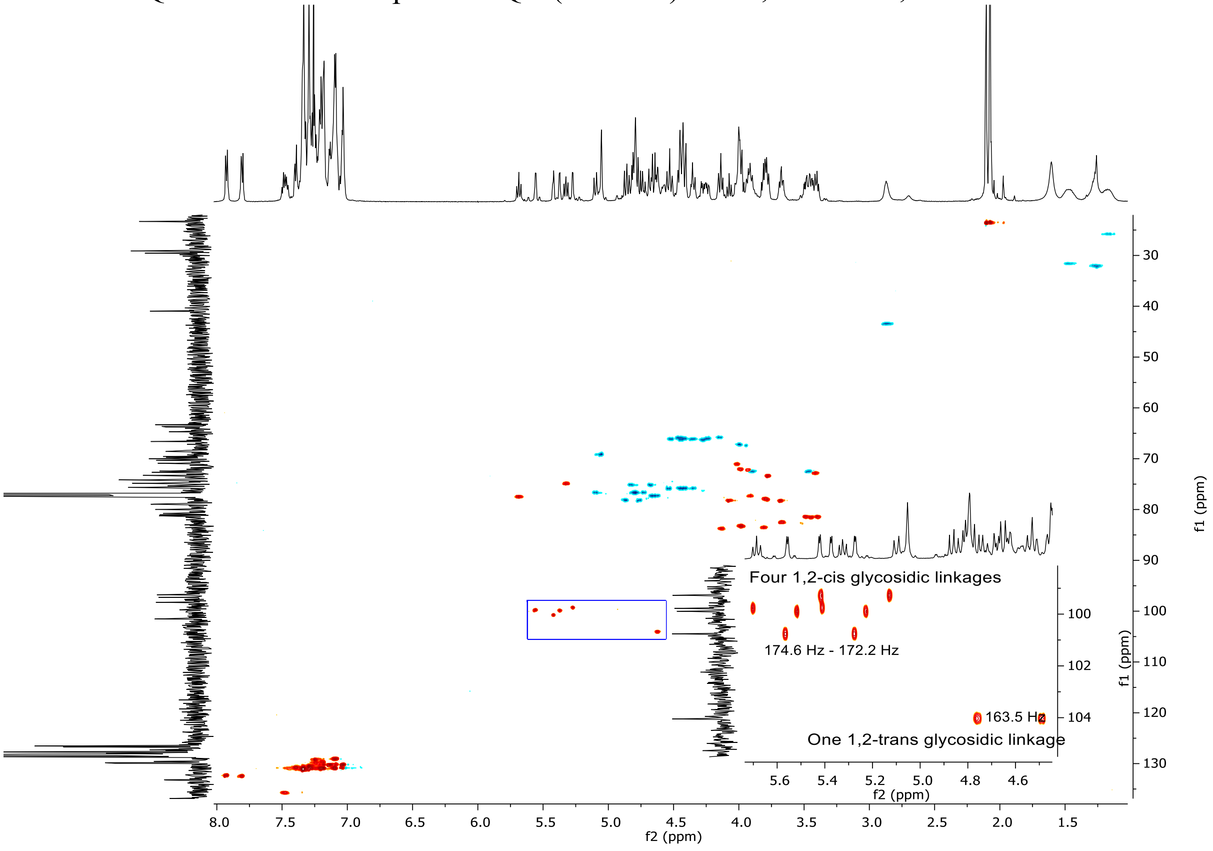

Supplementary Figure 112 | 2D NMR spectra of 35

^{1}H NMR, 600 MHz, $CDCl_3$

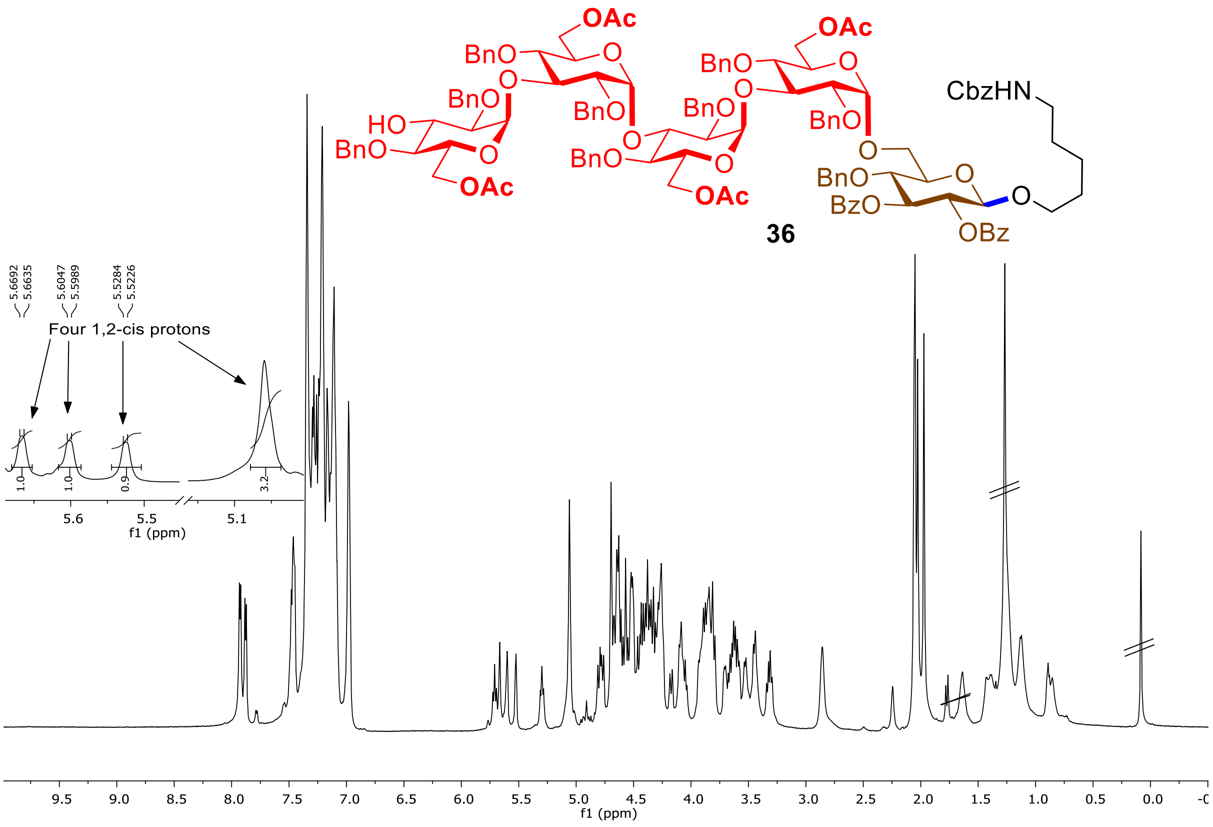

^{13}C NMR, 150 MHz, $CDCl_3$

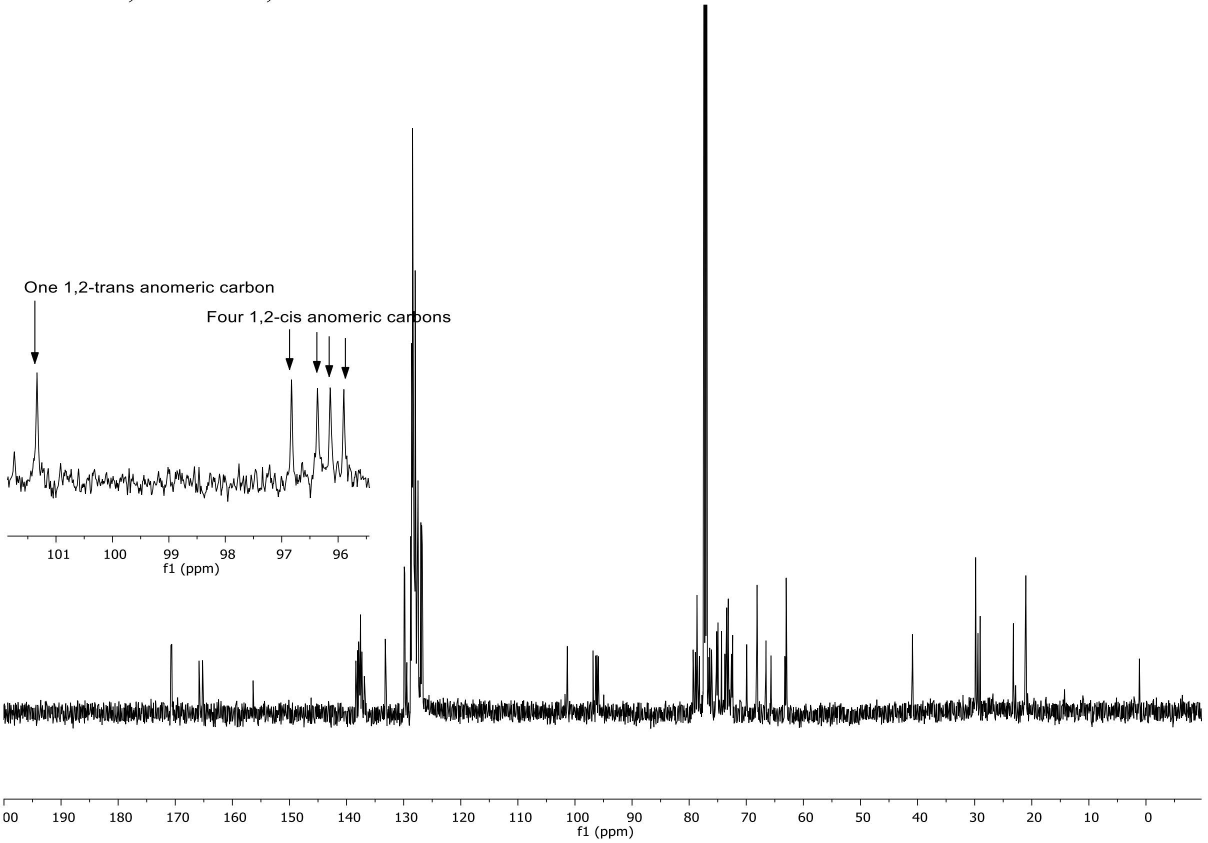

Supplementary Figure 113 | 1D NMR spectra of 36

^{1}H-COSY NMR, 600 MHz, $CDCl_3$

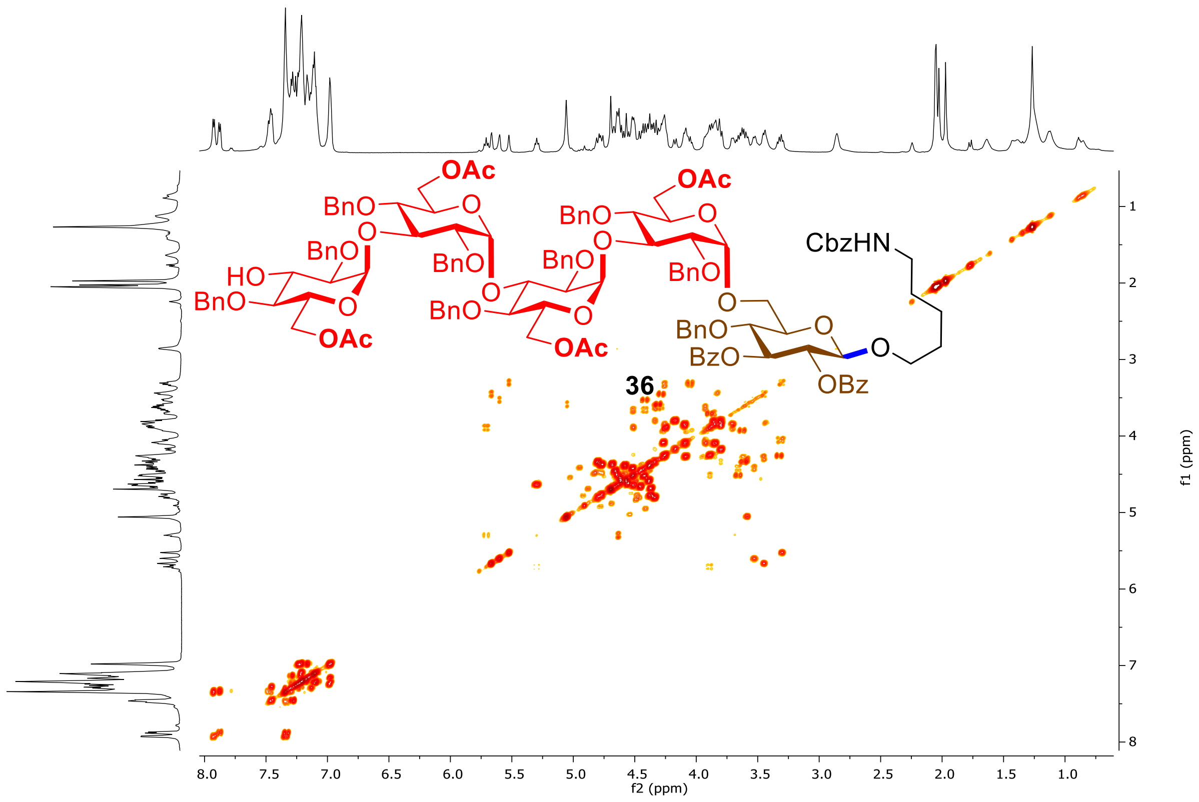

^{1}H-^{13}C-HSQC and ^{1}H-^{13}C-coupled-HSQC (zoom-in) NMR, 600 MHz, $CDCl_3$

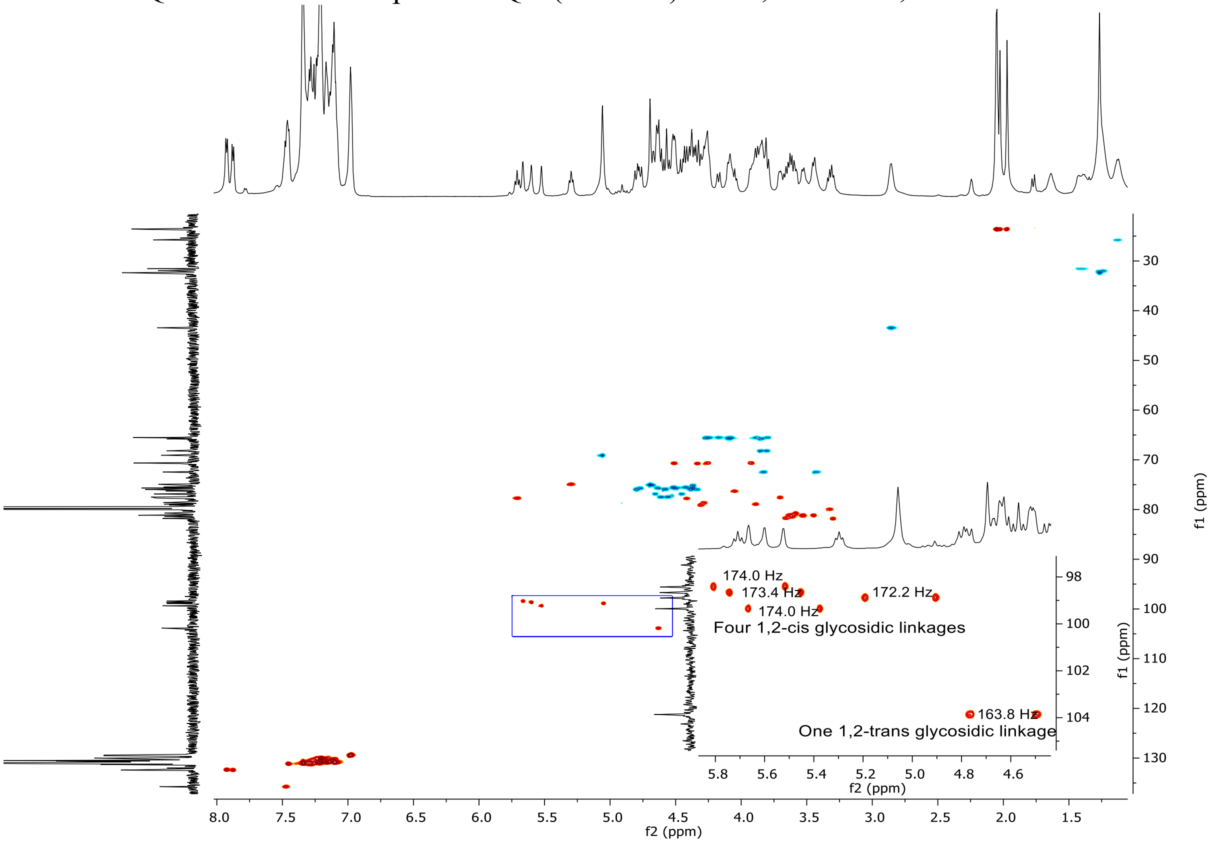

Supplementary Figure 114 | 2D NMR spectra of 36

^{1}H NMR, 600 MHz, $CDCl_3$

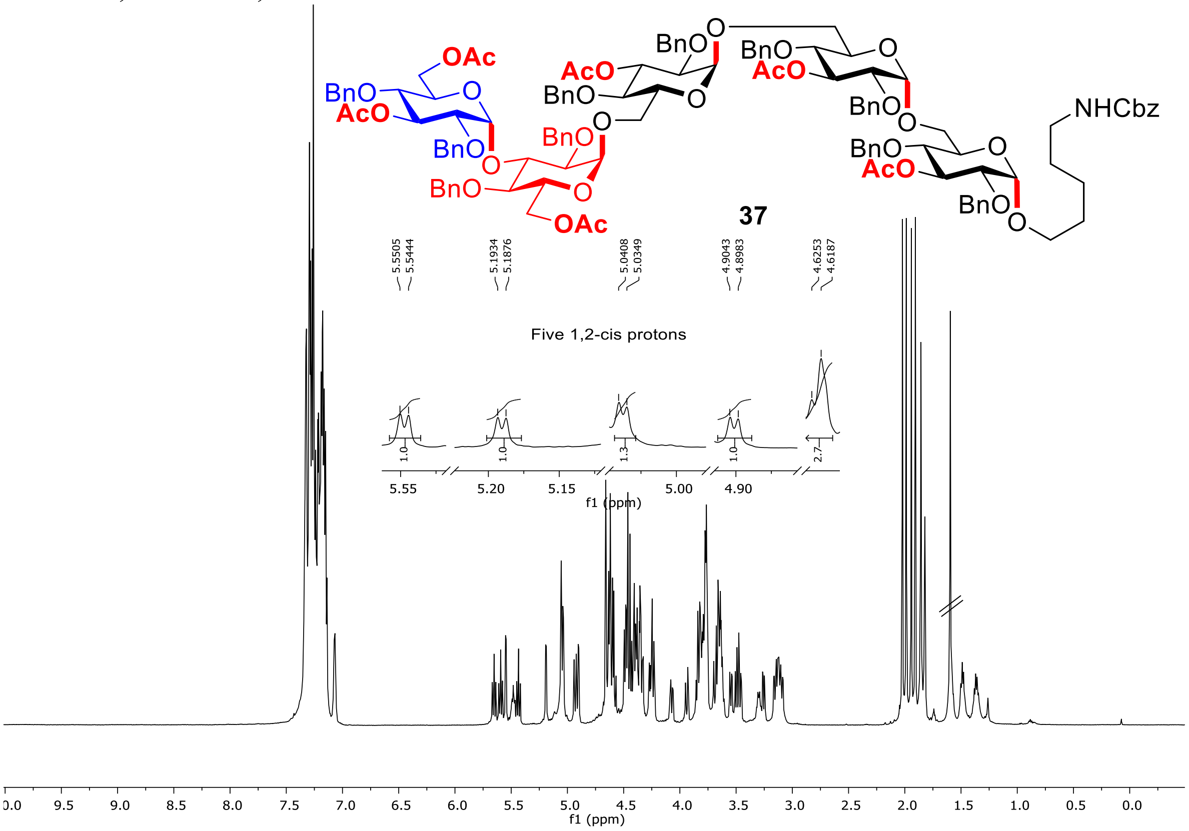

^{13}C NMR, 150 MHz, $CDCl_3$

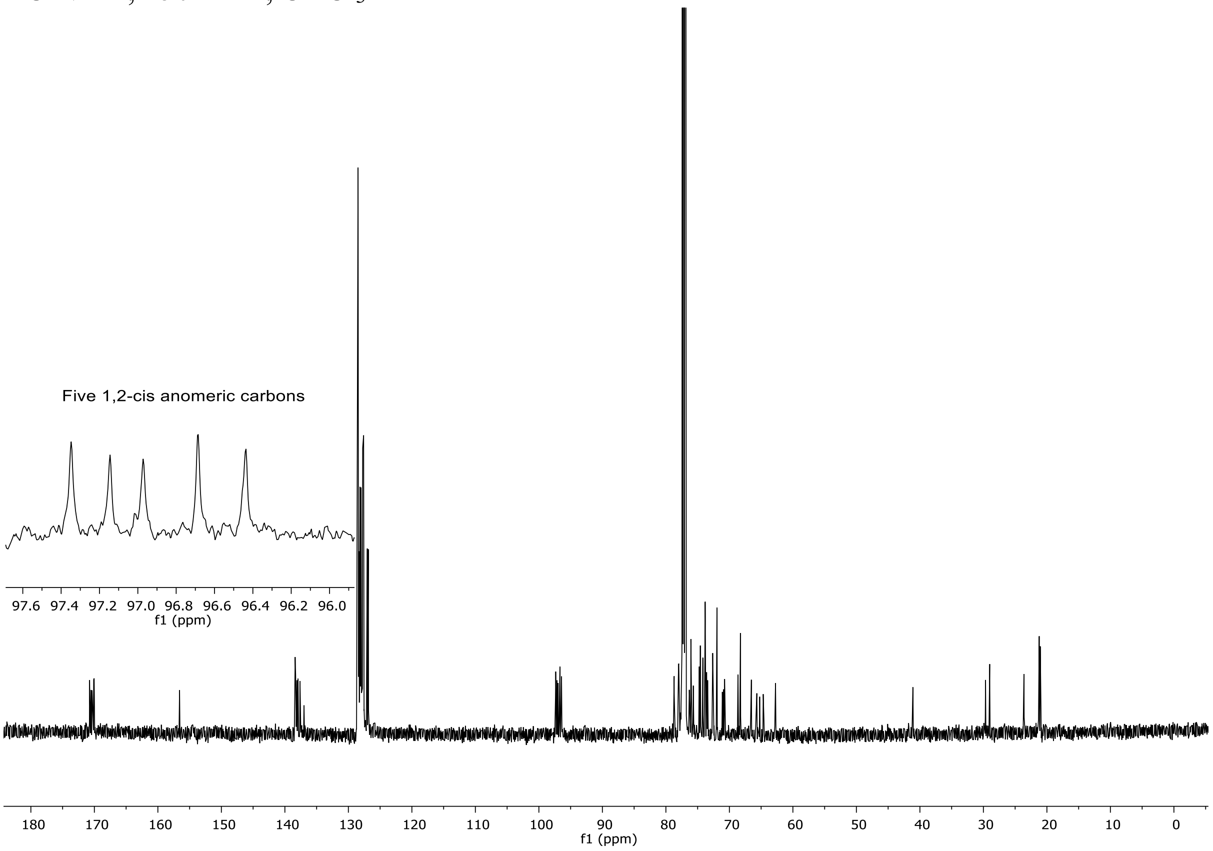

Supplementary Figure 115 | 1D NMR spectra of 37

^{1}H-COSY NMR, 600 MHz, $CDCl_3$

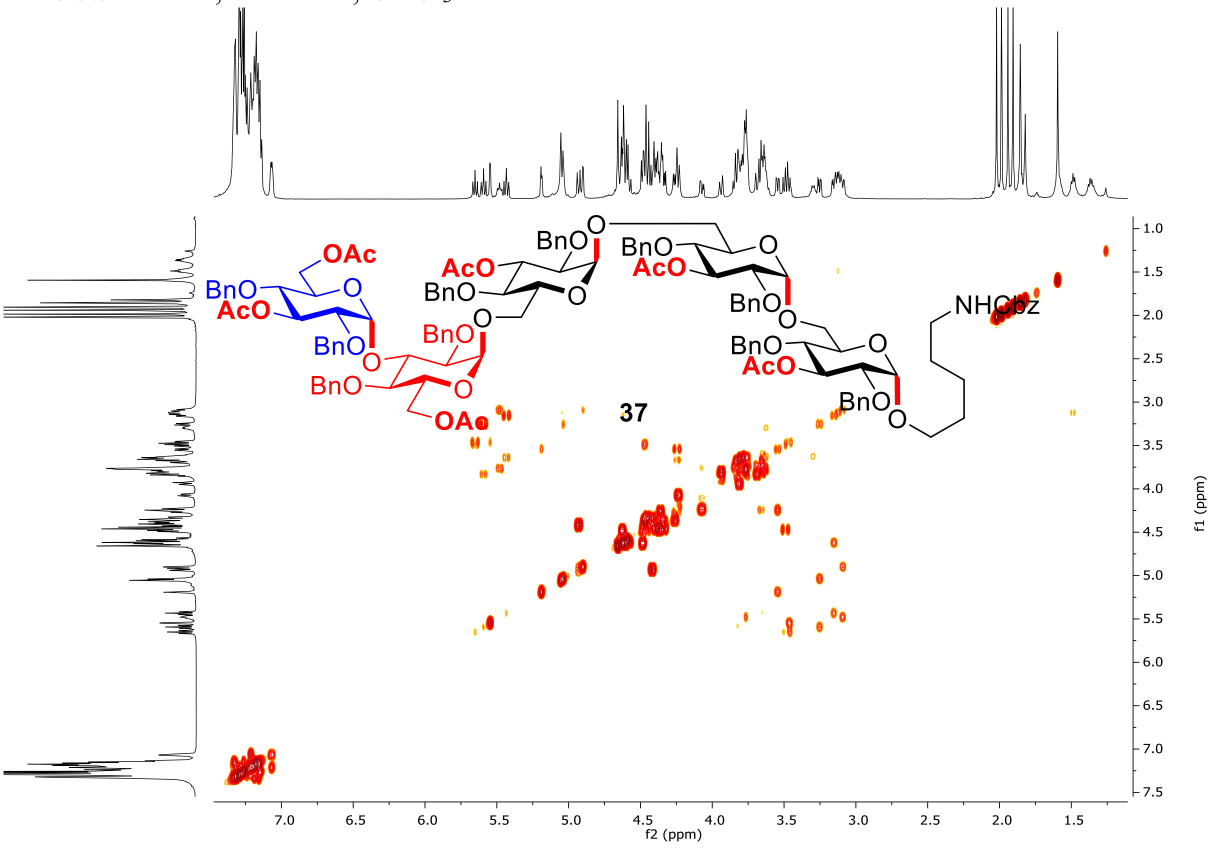

^{1}H-^{13}C-HSQC and ^{1}H-^{13}C-coupled-HSQC (zoom-in) NMR, 600 MHz, $CDCl_3$

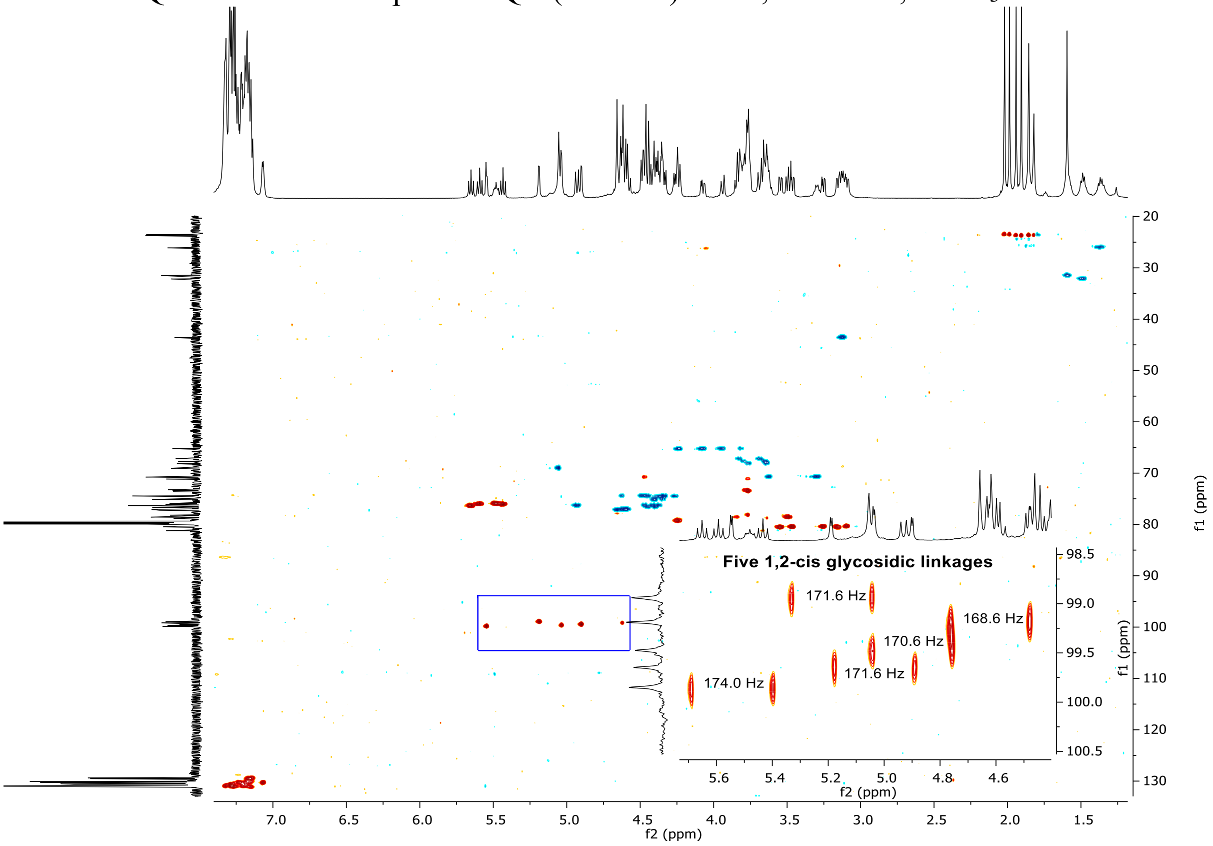

Supplementary Figure 116 | 2D NMR spectra of 37

^{1}H NMR, 600 MHz, $CDCl_3$

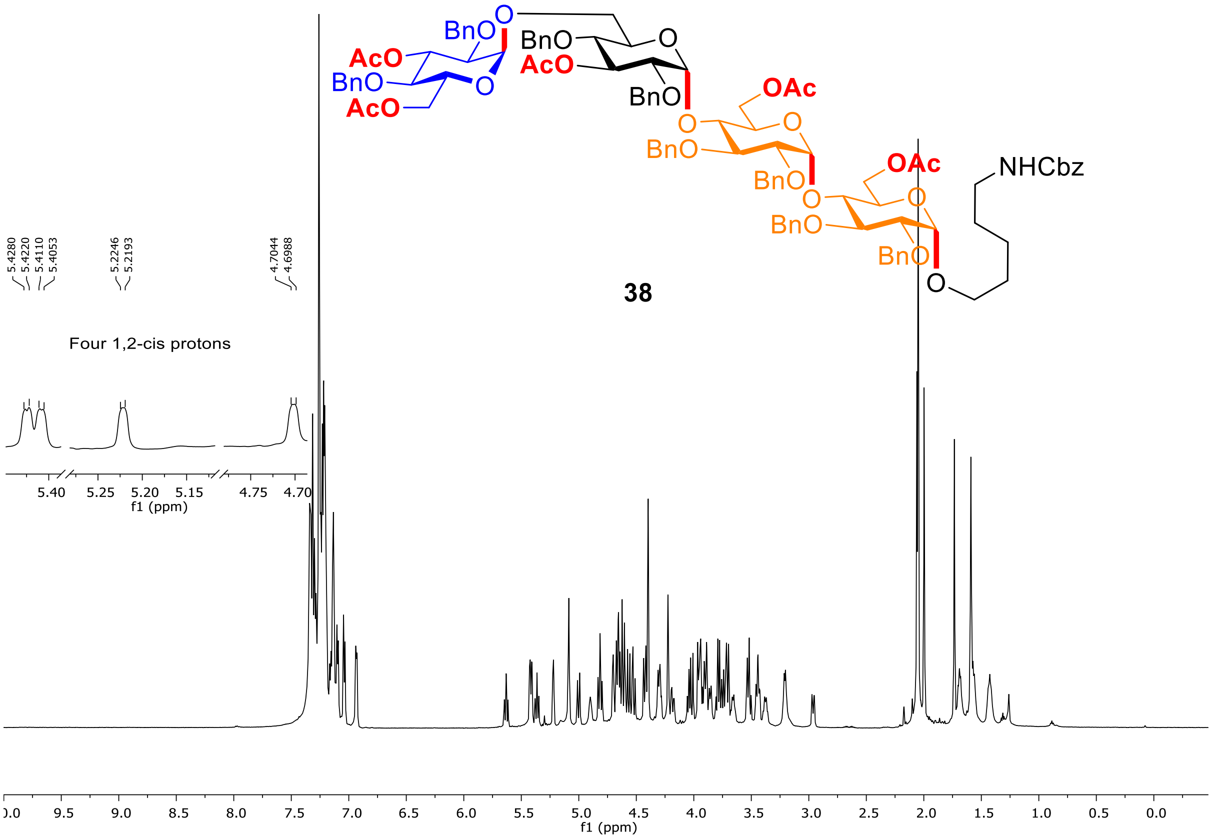

^{13}C NMR, 150 MHz, $CDCl_3$

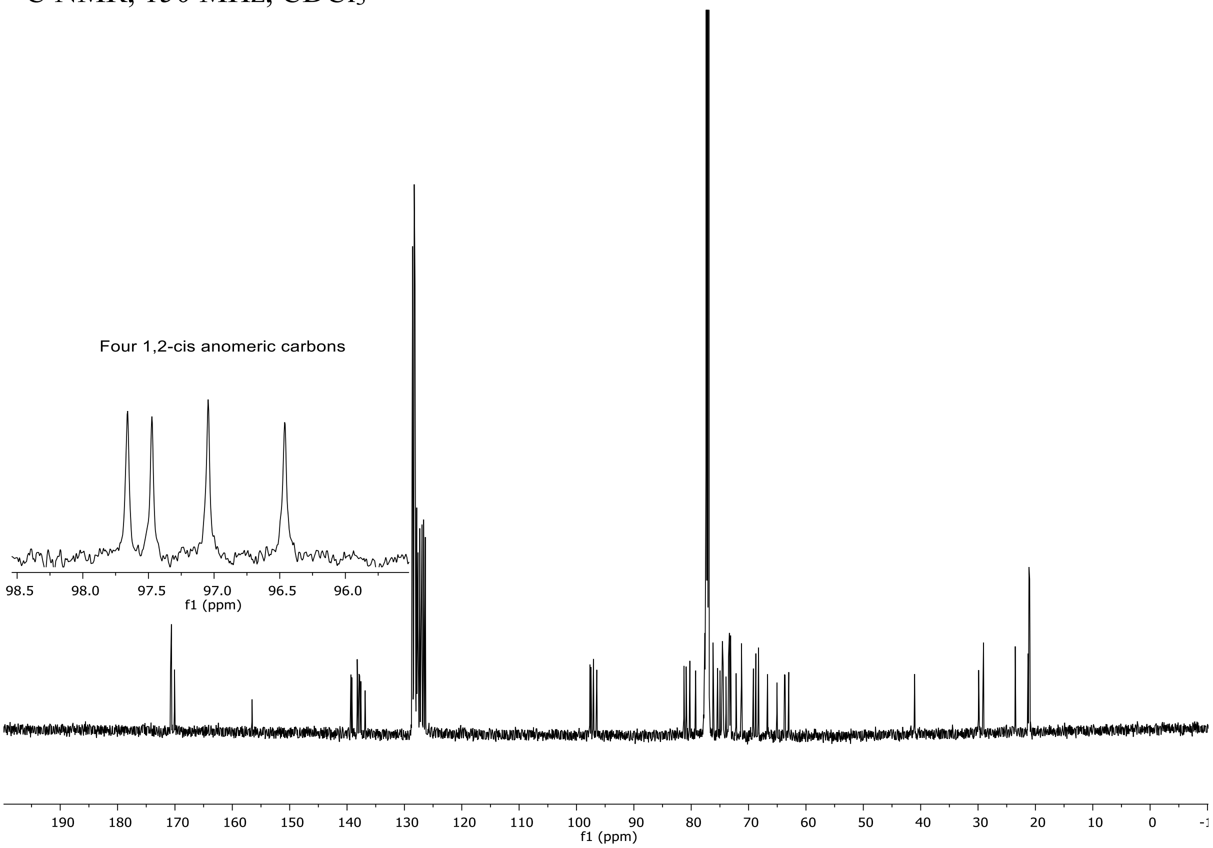

Supplementary Figure 117 | 1D NMR spectra of 38

^{1}H-COSY NMR, 600 MHz, $CDCl_3$

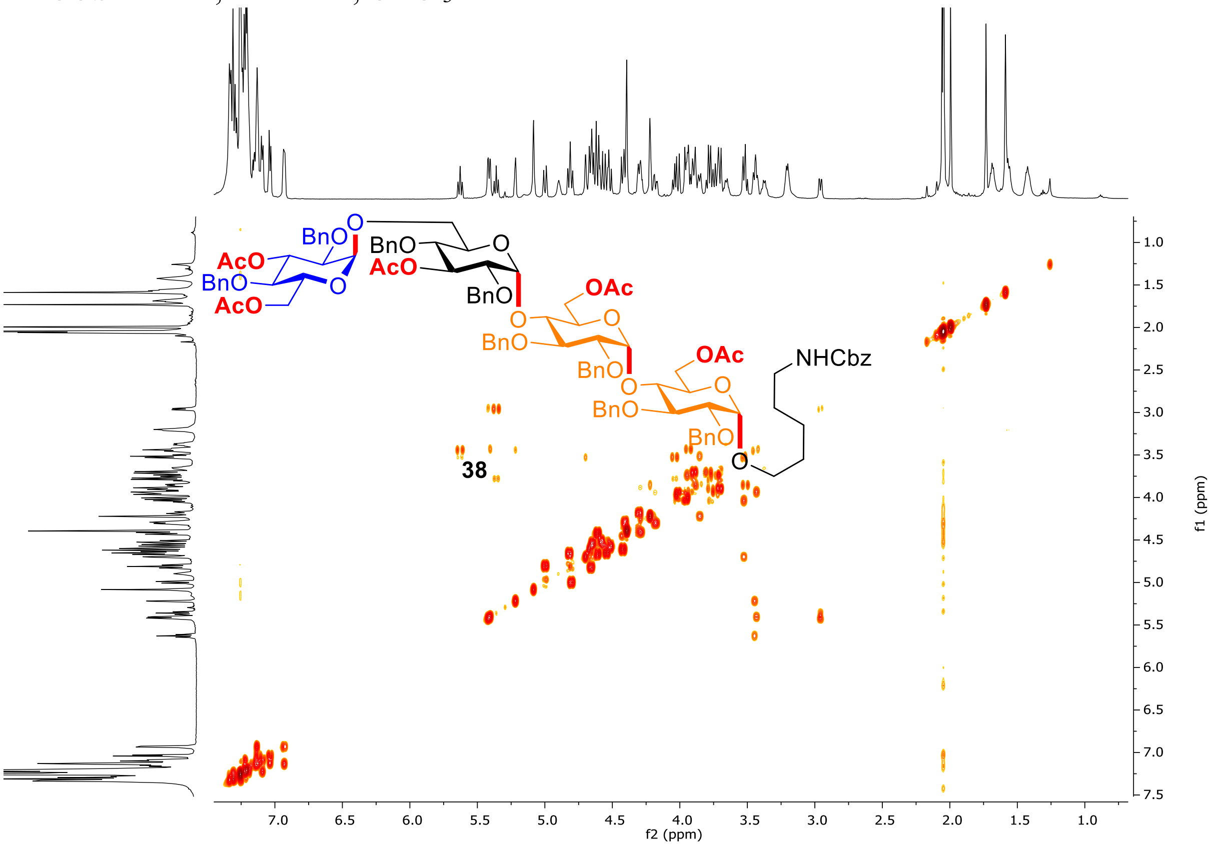

^{1}H-^{13}C-HSQC and ^{1}H-^{13}C-coupled-HSQC (zoom-in) NMR, 600 MHz, $CDCl_3$

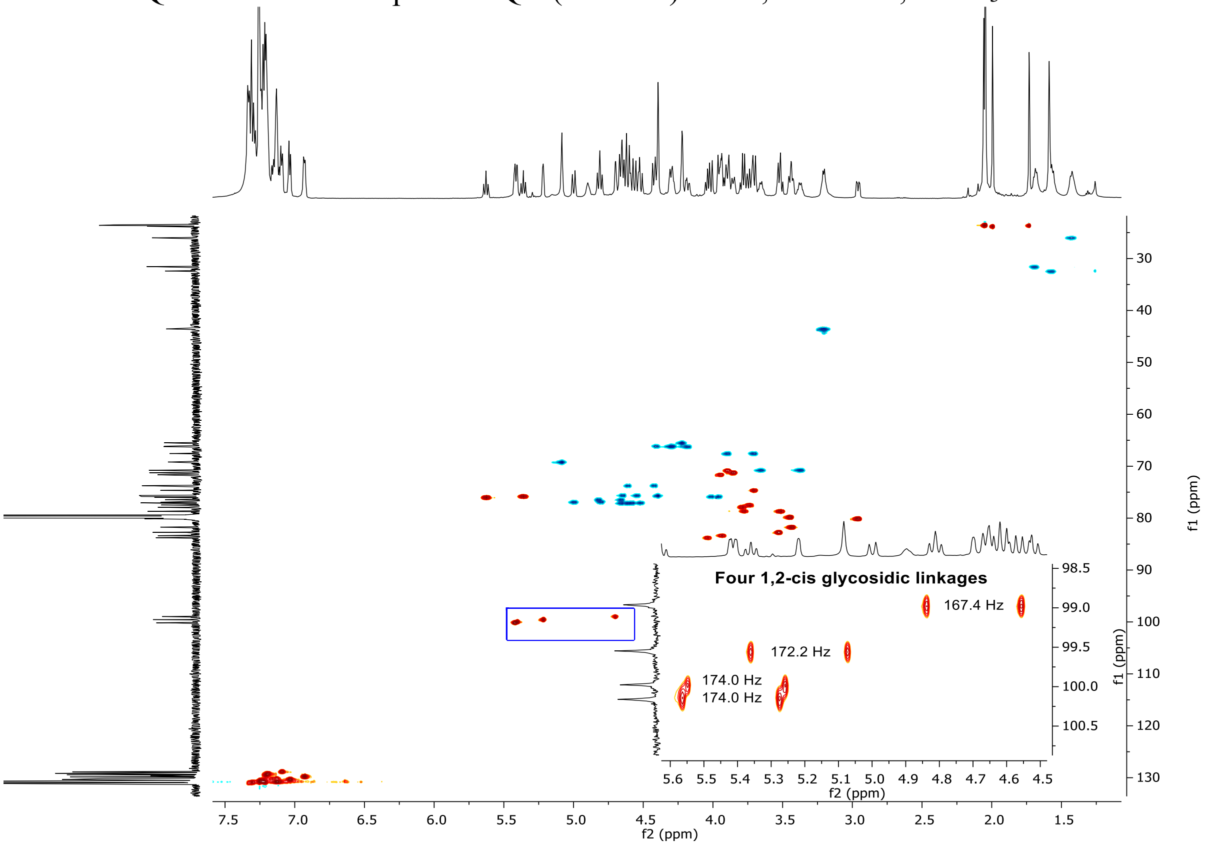

Supplementary Figure 118 | 2D NMR spectra of 38

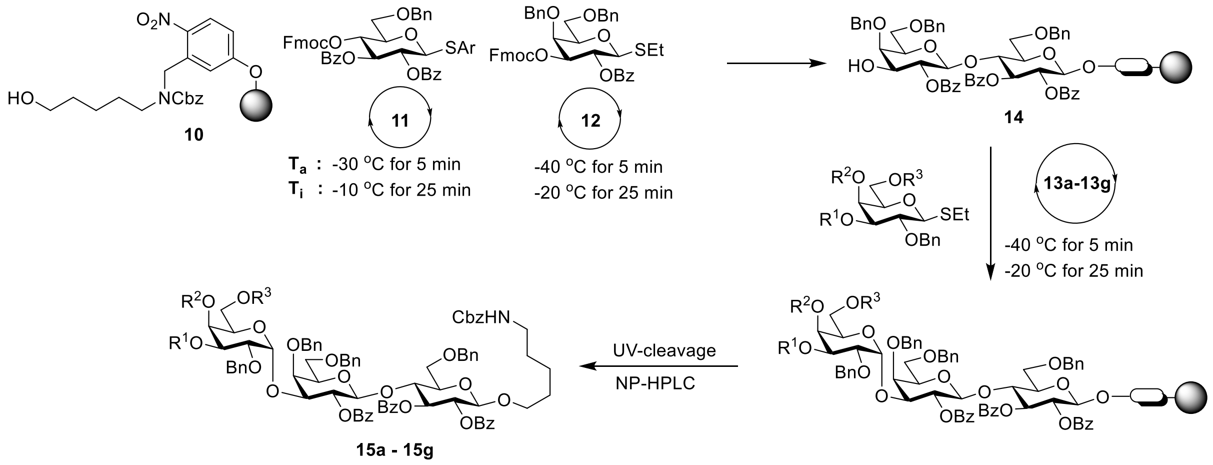

Sequence	Module	Details	Condition
I	1	2.5 eq. of TMSOTf solution	-20 °C, 1 min
	2	5 eq. building block **11**	$\mathbf{T_a}$ = -30 °C, $\mathbf{t_1}$ = 5 min
		5 eq. of NIS Solution	$\mathbf{T_i}$ = -10 °C, $\mathbf{t_2}$ = 25 min
	3	Fmoc Removal	r.t., 5 min
II	1	2.5 eq. of TMSOTf solution	-20 °C, 1 min
	2	5 eq. building block **12**	$\mathbf{T_a}$ = -40 °C, $\mathbf{t_1}$ = 5 min
		5 eq. of NIS Solution	$\mathbf{T_i}$ = -20 °C, $\mathbf{t_2}$ = 25 min
	3	Fmoc Removal	r.t., 5 min
III	1	2.5 eq. of TMSOTf solution	-20 °C, 1 min
	2	5 eq. building block **13a-13g**	$\mathbf{T_a}$ = -40 °C, $\mathbf{t_1}$ = 5 min
		5 eq. of NIS Solution	$\mathbf{T_i}$ = -20 °C, $\mathbf{t_2}$ = 25 min

Supplementary Table 1 | Modules of automated syntheses of trisaccharides 15a – 15g.

Entry	BB 2	Compound	Amounts	Yield
1	**13a**	**15a**	16 mg	41%
2	**13b**	**15b**	11 mg	29%
3	**13c**	**15c**	11 mg	28%
4	**13d**	**15d**	17 mg	44%
5	**13e**	**15e**	12 mg	30%
6	**13f**	**15f**	15 mg	41%
7	**13g**	**15g**	9 mg	24%

Supplementary Table 2 | Yields of trisaccharides 15a – 15g.

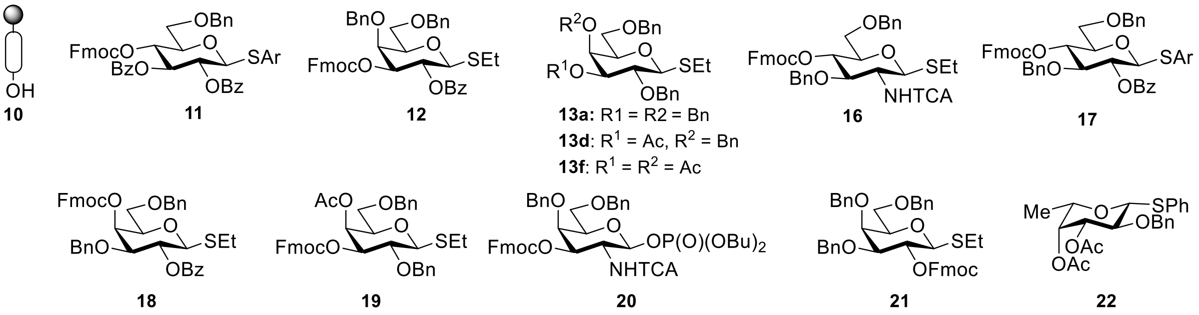

Sequence	Module	Details	Conditions
	1	2.5 eq. of TMSOTf solution	-20 °C, 1 min
I	2	5 eq. BB **11, 16**, or **17**	$\mathbf{T_a}$ = -30 °C, $\mathbf{t_1}$ = 5 min
		5.5 eq. of NIS Solution	$\mathbf{T_i}$ = -10 °C, $\mathbf{t_2}$ = 25 min
	3	Fmoc Removal	r.t., 5 min
	1	2.5 eq. of TMSOTf solution	-20 °C, 1 min
II	2	5 eq. BB **12, 18**, or **19**	$\mathbf{T_a}$ = -40 °C, $\mathbf{t_1}$ = 5 min
		5.5 eq. of NIS Solution	$\mathbf{T_i}$ = -20 °C, $\mathbf{t_2}$ = 25 min
	3	Fmoc Removal	r.t., 5 min
	1	2.5 eq. of TMSOTf solution	-20 °C, 1 min
III	2	5 eq. BB **13a-13g** or **22**	$\mathbf{T_a}$ = -40 °C, $\mathbf{t_1}$ = 5 min
		5.5 eq. of NIS Solution	$\mathbf{T_i}$ = -20 °C, $\mathbf{t_2}$ = 25 min
	1	2.5 eq. of TMSOTf solution	-20 °C, 1 min
IV	2	5 eq. BB **20**,	$\mathbf{T_a}$ = -30 °C, $\mathbf{t_1}$ = 5 min
		5.5 eq. of TMSOTf solution	$\mathbf{T_i}$ = -10 °C, $\mathbf{t_2}$ = 25 min
	3	Fmoc Removal	r.t., 5 min
	1	2.5 eq. of TMSOTf solution	-20 °C, 1 min
V	2	5 eq. BB **21**,	$\mathbf{T_a}$ = -40 °C, $\mathbf{t_1}$ = 5 min
		5.5 eq. of NIS Solution	$\mathbf{T_i}$ = -20 °C, $\mathbf{t_2}$ = 25 min
	3	Fmoc Removal	**30 °C**, 5 min

Supplementary Table 3 | Sequences of the glycosylation cycle with the corresponding monomers.

Compound		On-Resin Steps	Amounts	Yield
**23**	Alpha-Gal pentasaccharide	10	20 mg	33%
**24**	Gb-3	6	15 mg	35%
**25**	Gb-3	7	17 mg	41%
**26**	Globo-H	12	18 mg	33%

Supplementary Table 4. Yields of oligosaccharides **23-26.**

Compound	Amounts	Yield
**1**	2.5 mg	48%
**2**	4.1 mg	48%
**3**	3.3 mg	51%
**4**	2.9 mg	42%

Supplementary Table 5. Yields of oligosaccharides **1–4.**

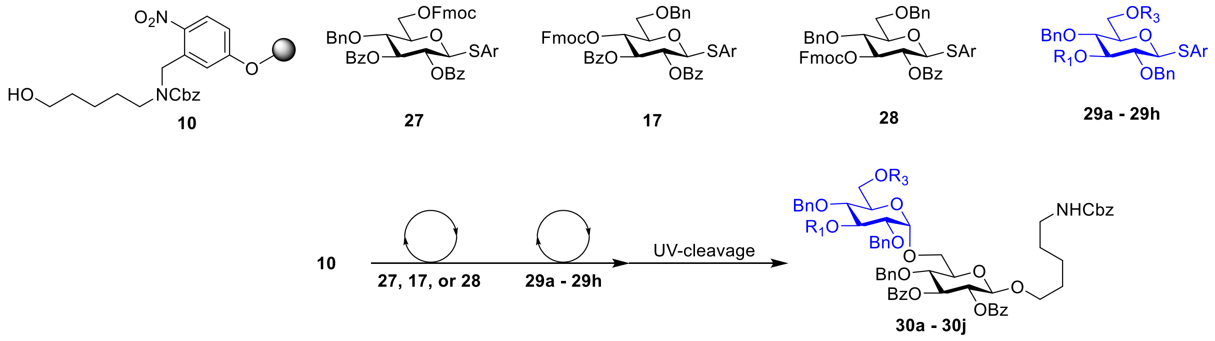

Sequence	Module	Details	Condition
I	1	2.5 eq. of TMSOTf solution	- 20 °C, 1 min.
	2	5 eq. building block **27, 17, or 28**	**T_a** = -30 °C, **t_1** = 5 min
		5.5 eq. of NIS Solution	**T_i** = -10 °C, **t_2** = 25 min
	3	Fmoc Removal	r.t., 5 min
II	1	2.5 eq. of TMSOTf solution	- 20 °C, 1 min.
	2	5 eq. building block **29a** – **29h**	**T_a** = -30 °C, **t_1** = 5 min
		5.5 eq. of NIS Solution	**T_i** = -10 °C, **t_2** = 25 min
	3	Fmoc Removal	r.t., 5 min

Supplementary Table 6. Modules of automated synthesis of disaccharides **30a – 30j.**

Entry	1st BB	2nd BB			Product					
			**R_1**	**R_2**		**R_3**	**R_4**	Amounts	Yield	Ratio (α : β)[a]
1	27	**29a**	Bn	Bn	**30a**	Bn	Bn	7.5 mg	37% (α:β = 25%:12%)	2.0 : 1
2		**29b**	Bn	Ac	**30b**	Bn	Ac	9.2 mg	31%	8.1 : 1
3		**29c**	Bn	Bz	**30c**	Bn	Bz	9.4 mg	31%	6.7 : 1
4		**29d**	Bn	Fmoc	**30d**	Bn	OH	8.4 mg	30%	3.5 : 1
5		**29e**	Ac	Bn	**30e**	Ac	Bn	8.2 mg	27%	4.4 : 1
6		**29f**	Bz	Bn	**30f**	Bz	Bn	8.6 mg	27%	3.4 : 1
7		**29g**	Fmoc	Bn	**30g**	OH	Bn	7.6 mg	26%	3.5 : 1
8		**29h**	Ac	Ac	**30h**	Ac	Ac	9.6 mg	34%	11.4 : 1
9	**17**	**29h**	Ac	Ac	**30i**	Ac	Ac	9.5 mg	34%	10.6 : 1
10	**28**	**29h**	Ac	Ac	**30j**	Ac	Ac	9.4 mg	34%	9.5 : 1
11	**27**	**29h**	Ac	Ac	**30h**	Ac	Ac	9.8 mg	35%	15.1 : 1[b]

Supplementary Table 7. Yields of disaccharides **30a – 30j.**

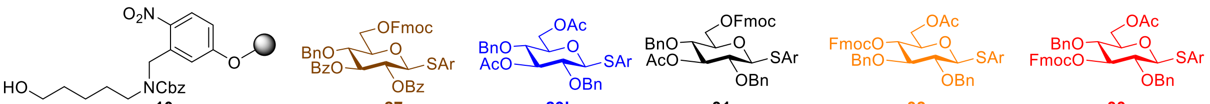

10 27 29h 31 32 33

Sequence	Module	Details	Conditions
I	1	2.5 eq. of TMSOTf solution	-20 °C, 1 min
	2	5 eq. building block **27**, 5.5 eq. of NIS Solution	$\mathbf{T_a}$ = -30 °C, $\mathbf{t_1}$ = 5 min<br>$\mathbf{T_i}$ = -10 °C, $\mathbf{t_2}$ = 25 min
	3	Fmoc Removal	r.t., 5 min
II	1	2.5 eq. of TMSOTf solution	-20 °C, 1 min
	2	5 eq. building block **31**, 5.5 eq. of NIS Solution	$\mathbf{T_a}$ = -30 °C, $\mathbf{t_1}$ = 5 min<br>$\mathbf{T_i}$ = -10 °C, $\mathbf{t_2}$ = 25 min
	3	Fmoc Removal	r.t., 5 min
III	1	2.5 eq. of TMSOTf solution	-20 °C, 1 min
	2	5 eq. building block **32**, 5.5 eq. of NIS Solution	$\mathbf{T_a}$ = -30 °C, $\mathbf{t_1}$ = 5 min<br>$\mathbf{T_i}$ = -10 °C, $\mathbf{t_2}$ = 50 min
	3	Fmoc Removal	r.t., 5 min
IV	1	2.5 eq. of TMSOTf solution	-20 °C, 1 min
	2	5 eq. building block **33**, 5.5 eq. of NIS Solution	$\mathbf{T_a}$ = -30 °C, $\mathbf{t_1}$ = 5 min<br>$\mathbf{T_i}$ = -10 °C, $\mathbf{t_2}$ = 50 min
	3	Fmoc Removal	r.t., 5 min
V	1	2.5 eq. of TMSOTf solution	-20 °C, 1 min
	2	5 eq. building block **29h**, 5.5 eq. of NIS Solution	$\mathbf{T_a}$ = -30 °C, $\mathbf{t_1}$ = 5 min<br>$\mathbf{T_i}$ = -10 °C, $\mathbf{t_2}$ = 25 min

Supplementary Table 8. Sequences of the glycosylation cycle with the corresponding monomers.

Compound	On-Resin Steps	Amounts	Yield
**34**	11	12.9 mg	23%
**35**	11	5.3 mg	9%
**36**	10	6.9 mg	12%
**37**	10	9.6 mg	17%
**38**	8	9.0 mg	20%

Supplementary Table 9. Yields of α-glucans **34 - 38.**

Compound	Amounts	Yield
**5**	2.6 mg	45%
**6**	0.8 mg	38%
**7**	0.9 mg	31%
**8**	1.9 mg	47%
**9**	1.2 mg	33%

Supplementary Table 10. Yields of α-glucans **5-9.**

Supplementary Methods

General Information

All chemicals used were reagent grade and used as supplied, except where noted. Prior to use, molecular sieves were activated by heating under high vacuum. All reactions were performed in oven-dried glassware under an argon atmosphere, unless noted otherwise. *N*,*N*-Dimethylformamide (DMF), dichloromethane (DCM), toluene and tetrahydrofuran (THF) were purified in a Cycle-Tainer Solvent Delivery System, unless noted otherwise. Analytical thin layer chromatography (TLC) was performed on Merck silica gel 60 F254 plates (0.25 mm). Compounds were visualized by UV-irradiation, or by dipping the plate either in a cerium sulfate ammonium molybdate (CAM) solution or a 1:1 mixture of H_2SO_4 (2 N) and resorcine monomethylether (0.2%) in ethanol. Flash column chromatography was carried out using forced flow of the indicated solvent on Fluka Kieselgel 60 (230-400 mesh). All automated glycosylations were performed on a prototype automated oligosaccharide synthesizer using anhydrous solvents of the Cycle-Tainer Solvent Delivery System. LCMS chromatograms were recorded on an Agilent 1100 Series spectrometer. Preparative HPLC purifications were performed on an Agilent 1200 Series. Loading determination of functionalized resins was obtained using a Shimadzu UV-MINI-1240 UV spectrometer. ^{1}H and ^{13}C NMR spectra were recorded a Varian Mercury 400 (400 MHz), 600 (600MHz) or Bruker DRX700 (700 MHz) spectrometers in $CDCl_3$ or D_2O with chemical shifts referenced to internal standards ($CDCl_3$: 7.26 ppm ^{1}H, 77.16 ppm ^{13}C; CD_3OD: 4.87 or 3.13 ppm ^{1}H, 49.0 ppm ^{13}C) unless stated otherwise. Splitting patterns are indicated as s, singlet; d, doublet; t, triplet; q, quartet; m, multiplet; brs, broad singlet for 1H-NMR data. NMR chemical shifts (δ) are reported in ppm and coupling constants (*J*) are reported in Hz. High resolution mass spectral (HRMS) analyses were performed by the MS-service in the Department of Organic Chemistry at Free University Berlin using an Agilent 6210 ESI-TOF (Agilent Technologies, Santa Clara, CA, USA). IR spectra were recorded on a Perkin-Elmer 1600 FTIR spectrometer. Optical rotations were measured with a UniPol L 1000 polarimeter (Schmidt & Haensch, Berlin, Germany), with concentrations expressed in g per 100 mL. MALDI-TOF spectra were recorded on a Bruker Daltonics Autoflex Speed, using a 2,4,6-trihydroxyacetophenone (THAP) matrix.

Pre-Automation

Photolabile linker bound resin

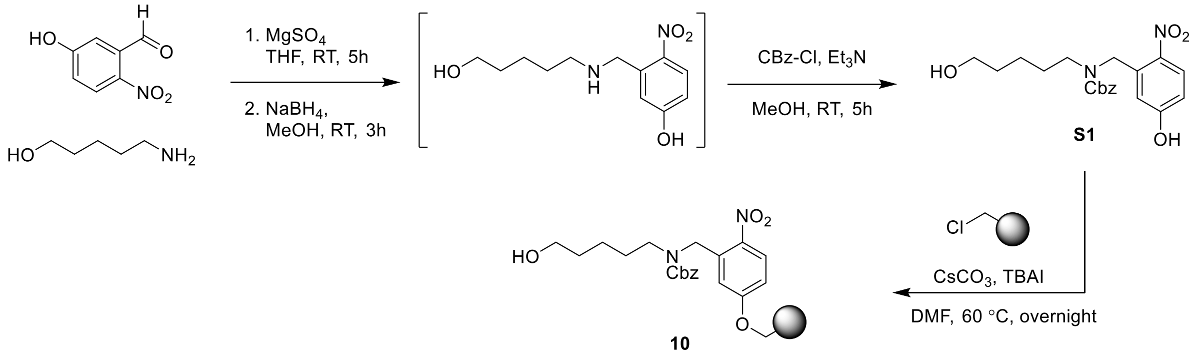

The known resin **10** (loading 0.392 mmol/g) was prepared according to literature[1,2].

Building Blocks

2-Methyl-5-tert-butylphenyl 2,3-di-*O*-benzoyl-6-*O*-benzyl-4-*O*-fluorenylmethoxycarbonyl-1-thio-β-D-glucopyranoside 11[1].

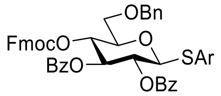

11

Ethyl 2-*O*-benzoyl-4,6-di-*O*-benzyl-3-*O*-fluorenylmethoxycarbonyl-1-thio-β-D-galactopyranoside 12[1]

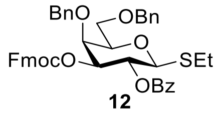

12

Known compounds **11** and **12**, please reference[1] for details.

Ethyl 2,3,4,6-tetra-*O*-benzyl-1-thio-β-D-galactopyranoside 13a[3]

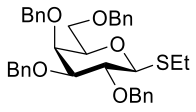

13a

Known compound, please reference[3] for details.

Ethyl 6-*O*-acetyl-2,3,4-tri-*O*-benzyl-1-thio-β-D-galactopyranoside 13b[4]

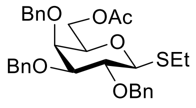

13b

Known compound, please reference[4] for details.

Ethyl 6-*O*-benzoyl-2,3,4-tri-*O*-benzyl-1-thio-β-D-galactopyranoside 13c

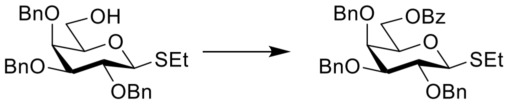

S2 **13c**

To a solution of compound **S2**[5] (0.21 g, 0.43 mmol) in anhydrous DCM (2.1mL, 0.2 M) were added were added benzoic anhydride (0.192 g, 0.849 mmol), triethylamine (0.12 mL, 0.849 mmol), and a catalytic amount of DMAP (10.4 mg, 0.085 mmol) at 0°C. After the mixture was stirred overnight at room temperature, the mixture was quenched with saturated aqueous $NaHCO_3$, and dried over $MgSO_4$. The combined organic phase was dried over $MgSO_4$, and evaporated *in vacuo*. The crude product was purified by column chromatography on silica gel (hexane/ethyl acetate = 9:1 to 7:3) to afford **13c** (0.224 g, 0.374 mmol, 88%). R_f = 0.21 (hexane/ethyl acetate/DCM, 9:1:0.5); $[\alpha]_D^{20}$ = -22.26 (c = 2.30, CDCl3); IR (thin film): υ = 2869, 1724, 1453, 1267, 1096, 1069 cm-1; ^{1}H NMR (400 MHz, $CDCl_3$) δ 7.93 (dd, *J* = 8.2, 1.1 Hz, 2H), 7.57 (dd, *J* = 10.6, 4.3 Hz, 1H), 7.50 – 6.98 (m, 17H), 5.02 (d, *J* = 11.6 Hz, 1H, C*H*HPh), 4.90 (d, *J* = 10.2 Hz, 1H, C*H*HPh), 4.84 – 4.75 (m, 3H, 3 x C*H*HPh), 4.70 (d, *J* = 11.7 Hz, 1H, C*H*HPh), 4.52 – 4.45 (m, 2H, **H-1**, H-6), 4.32 (dd, *J* = 11.2, 6.0 Hz, 1H, H-6), 3.90 – 3.86 (m, 2H, H-2, H-4), 3.70 (t, *J* = 6.4 Hz, 1H, H-5), 3.61 (dd, *J* = 9.3, 2.7 Hz, 1H, H-3), 2.86 – 2.64 (m, 2H), 1.29 (t, *J* = 7.4 Hz, 3H). ^{13}C NMR (100 MHz, $CDCl_3$) δ 166.25 (Bz), 138.36, 138.34, 138.29, 133.26, 129.90, 129.75, 128.61, 128.59, 128.48, 128.45, 128.41, 127.93, 127.90, 127.86, 127.74 (Ar), 85.54 (C-1), 84.28 (C-3), 78.58 (C-2), 76.08 (CH_2Ph), 75.99 (C-5), 74.47 (CH_2Ph), 73.50 (C-4), 73.37 (CH_2Ph), 63.68 (C-6), 25.13, 15.27. MS ESI+-HRMS m/z [M+Na]+ calcd for $C_{36}H_{38}O_6SNa$ 621.2281, found 621.2292.

Ethyl 4-*O*-acetyl-2,3,6-tri-*O*-benzyl-1-thio-β-D-galactopyranoside 13d[6]

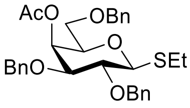

13d

Ethyl 4-*O*-benzoyl-2,3,6-tri-*O*-benzyl-1-thio-β-D-galactopyranoside 13e[6]

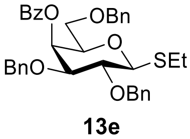

13e

Known compounds **13d** and **13e**, please reference[6] for details.

Ethyl 3,4-di-*O*-acetyl-2,6-di-*O*-benzyl-1-thio-β-D-galactopyranoside 13f[6]

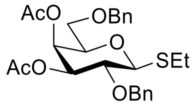

13f

Known compound, please reference[7] for details.

Ethyl 4,6-di-*O*-acetyl-2,3-di-*O*-benzyl-1-thio-β-D-galactopyranoside 13g

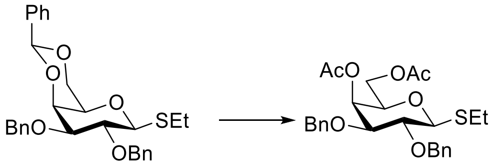

To a solution of compound **S3**[5] (0.502 g, 1.102 mmol) in DCM (100 ml) and water (1.1 mL) was added TFA (1.6 mL) and the mixture was stirred at 0 °C for 4 h. After the mixture was quenched with saturated aqueous $NaHCO_3$, and diluted with DCM, combined organic phase was dried over $MgSO_4$, and evaporated *in vacuo*. Without further purification, the crude was used in the next step. To a solution of the crude mixture in DCM (5.1 ml, 0.2 M) were added acetic anhydride (0.48 ml, 5.08 mmol), triethylamine (1.42 ml, 1.03 mmol), and DMAP (0.025 g, 0.203 mmol) in the ice bath, and the mixture was stirred overnight at room temperature. After the mixture was quenched with saturated aqueous $NaHCO_3$, and diluted with DCM, organic phase was separated and aqueous phase was extracted two more times with DCM. Combined organic phase was dried over $MgSO_4$, and evaporated *in vacuo*. The crude product was purified by column chromatography on silica gel (hexane:ethyl acetate:DCM = 8/1/0.5 to 7/3/0.5) to give **13g** (0.416 g, 0.851 mmol, 84%) over two steps. R_f = 0.22(hexane/ethyl acetate/DCM, 7:3); $[\alpha]_D^{20}$ = 28.56 (c = 3.10, $CDCl_3$); IR (thin film): υ = 2869, 1752, 1452, 1251 cm-1; ^{1}H NMR (400 MHz, $CDCl_3$) δ 7.41 – 7.25 (m, 10H), 5.55 (d, *J* = 2.3 Hz, 1H, H-4)), 4.79 (dd, *J* = 21.8, 10.4 Hz, 3H, C*H*HPh, CH_2Ph), 4.52 (d, *J* = 11.1 Hz, 1H, C*H*HPh), 4.48 (d, *J* = 8.9 Hz, 1H, H-1) 4.19 – 4.10 (m, 2H, H-6), 3.78 (t, *J* = 6.5 Hz, 1H, H-5), 3.66 – 3.55 (m, 2H, H-2, H-3), 2.76 (qd, *J* = 12.7, 7.1 Hz, 2H), 2.16 (s, 3H, Ac), 2.07 (s, 3H, Ac), 1.33 (t, *J* = 7.4 Hz, 3H). ^{13}C NMR (100 MHz, $CDCl_3$) δ 170.69 (Ac), 170.58 (Ac), 138.14, 137.69, 128.54, 128.48, 128.46, 128.26, 127.97 (Ar), 85.61 (C-1), 80.93 (C-3), 77.77 (C-2), 76.06 (CH_2Ph), 74.50 (C-5), 72.16 (CH_2Ph), 66.76 (C-4), 62.40 (C-6), 25.29, 21.08 (Ac), 20.91 (Ac), 15.24. MS ESI+-HRMS m/z [M+Na]+ calcd for $C_{26}H_{32}O_7SNa$ 511.1761, found 511.1765.

Ethyl 3,6-di-*O*-benzyl-4-*O*-fluorenylmethoxycarbonyl-2-deoxy-2-trichloroacetamino-1-thio-β-D-glucopyranoside 16[3]

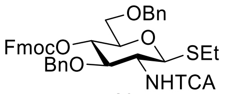

16

Known compound, please reference[3] for details.

2-Methyl-5-turt-butylphenyl-2-O-benzoyl -3, 6-di-O-benzyl-1-thio-β-D-glucopyranose 17[2]

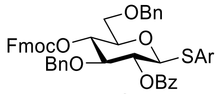

17

Known compound, please reference[1] for details

Ethyl 2-O-benzoyl-4,6-di-O-benzyl-4-O-fluorenylmethoxycarbonyl-1-thio-β-D-galactopyranoside 18[2]

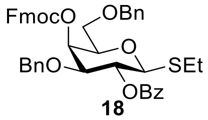

18

Ethyl 4-*O*-acetyl-2,6-di-*O*-benzyl-3-*O*-fluorenylmethoxycarbonyl-1-thio-β-D-galactopyranoside 19[2]

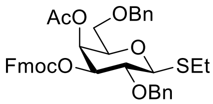

19

Known compounds **18** and **19**, please reference[1] for details.

Phenyl 4,6-di-*O*-benzyl-3-*O*-fluorenylmethoxycarbonyl-2-deoxy-2-trichloroacetamino-1-thio-β-D-galactopyranoside 20[8]

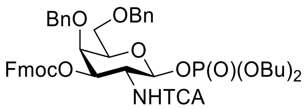

20

Known compound, please reference[8] for details.

Ethyl 3,4,6-tri-O-benzyl-2-O-fluorenylmethoxycarbonyl-1-thio-β-D-galactopyranoside 21

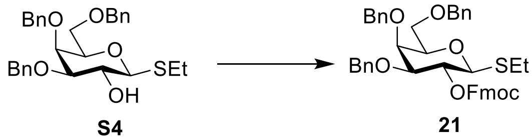

To a solution of compound **S4**[9] in anhydrous DCM (15 mL, 0.3 M) were added 9-fluorenylmethylchloroformate (2.3 g, 8.8 mmol) and pyridine (1.1 mL, 13.2 mmol,) at 0 °C. The reaction was stirred at r.t. overnight afterwhich, the mixture was diluted with DCM and quenched with 1M aqueous HCl. The organic phase was separated and aqueous phase was extracted twice with DCM. The combined organic layer was dried over $MgSO_4$ and the solvent was evaporated *in vacuo*. The crude product was purified by column chromatography on silica gel (hexane/ethyl acetate/DCM = 8:1:1 to 8:2:1) to afford **21** (2.7 g, 3.77 mmol, 86%). Rf = 0.17 (hexane/ethyl acetate /DCM = 9:1:0.5); $[\alpha]_D^{20}$ = -12.26 (c = 1.30, $CHCl_3$); IR (thin film): υ = 2869, 1752, 1452, 1251 cm-1; ^{1}H NMR (CDCl3) 8.03 (d, J = 7.5 Hz, 2H), 7.78 (d, J = 7.5 Hz, 2H), 7.59 (t, J = 7.7 Hz, 3H), 7.49 – 7.39 (m, 4H), 7.38 – 7.26 (m, 7H), 7.23 – 7.14 (m, 5H), 5.71 (t, J = 9.7 Hz, 1H, H-2), 5.04 (d, J = 11.7 Hz, 1H, C*H*HPh), 4.68 (d, J = 12.2 Hz, 1H, C*H*HPh), 4.67 (d, J = 11.7 Hz, 1H, C*H*HPh), 4.56 (d, J = 12.2 Hz, 1H, C*H*HPh), 4.52 (d, J = 9.9 Hz, 1H, 4.44 – 4.34 (m, 3H, H-6, CH2 of Fmoc), 4.25 (t, J = 7.4 Hz, 1H, CH of Fmoc), 4.18 (dd, J = 11.1, 5.8 Hz, 1H, H-6), 3.97 (d, J = 1.9 Hz, 1H, H-4), 3.72 (dd, J = 13.2, 4.1 Hz, 2H, H-3, H-5), 2.82 – 2.64 (m, 2H), 1.22 (t, J = 7.4 Hz, 3H). ^{13}C NMR (100 MHz, cdcl3) 154.66 (Fmoc), 143.68, 143.47, 141.42, 141.40, 138.66, 137.95, 137.95, 128.56, 128.53, 128.32, 128.11, 128.04, 127.96, 127.94, 127.92, 127.85, 127.63, 127.57, 127.29, 127.26, 125.43, 125.31, 120.12 (Ar), 83.74 (C-1), 81.58 (C-3), 77.67 (C-5), 74.62 (CH_2Ph), 74.44 (C-2), 73.71 (CH_2Ph), 73.39 (C-4), 72.48 (CH_2Ph), 70.24 (CH_2, Fmoc), 68.65 (C-6), 46.93 (CH, Fmoc), 24.05, 15.05. MS ESI+-HRMS m/z [M+Na]+ calcd for C44H44O7SNa 739.2705, found 739.2699.

Phenyl 3,4-di-*O*-acetyl-2-*O*-benzyl-1-thio-β-L-fucopyranoside 22[10]

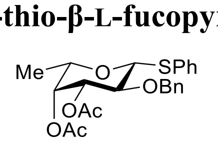

22

Known compound, please reference[10] for details.

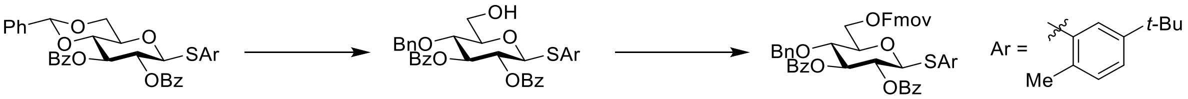

S5 S6 27

2-Methyl-5-tert-butylphenyl 2,3-di-*O*-benzoyl-4-*O*-benzyl-1-thio-β-D-glucopyranoside S4

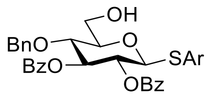

S6

Compound **S5**[1] (4.7 g, 7.36 mmol) was co-evaporated with toluene and dissolved under an Ar atmosphere in DCM (41 mL, 0.18 M). To a solution of compound **S5** (10.0 g, 15.7 mmol) was added triethylsilane (7.05 mL, 44.1 mmol) and trifluoroacetic anhydride (0.520 mL, 3.68 mmol) at 0 °C. After 30 min, trifluoroacetic acid (2.83 mL, 36.8 mmol) was added slowly, and the reaction mixture was allowed to warm up to room temperature gradually. After 5 h, the mixture was quenched with saturated aqueous $NaHCO_3$, and then diluted with DCM. The organic layer was separated and aqueous phase was extracted twice with DCM. The organic layer was dried over $MgSO_4$ and the solvent was evaporated *in vacuo*. The crude product was purified by column chromatography on silica gel (hexane/ethyl acetate /DCM = 9:1:0.5 to 7:3:0.5) to afford **S6** (4.44 g, 6.94 mmol, 94%). R_f = 0.21 (hexane/ethyl acetate /DCM = 8:2:0.5). $[\alpha]_D^{25}$ = 93.96 (c = 3.50, $CHCl_3$). IR (thin film): υ = 3458, 2961, 1729, 1274 cm^{-1}; ^1H NMR (400 MHz, $CDCl_3$) δ 8.07 – 7.82 (m, 4H), 7.59 (d, J = 2.0 Hz, 1H), 7.51 (ddd, J = 8.7, 2.5, 1.3 Hz, 2H), 7.40 – 7.27 (m, 8H), 7.22 (dd, J = 8.0, 2.1 Hz, 1H), 7.09 (d, J = 8.0 Hz, 1H), 5.63 – 5.21 (m, 2H, H-2, H-3), 4.88 (d, J = 9.7 Hz, 1H, H-1), 4.62 (q, J = 12.0 Hz, 2H, CH_2Ph), 4.00 (td, J = 9.3, 3.1 Hz, 1H, H-4), 3.86 (d, J = 4.6 Hz, 2H, H-6), 3.71 (dt, J = 9.3, 4.5 Hz, 1H, H-5), 3.23 (br, J = 3.4 Hz, 1H, OH), 2.23 (s, 3H, Me), 1.26 (s, 9H, *t*-Bu). ^{13}C NMR (100 MHz, $CDCl_3$) δ 167.29 (OBz), 165.41 (OBz), 149.79, 137.71, 137.22, 133.56, 133.38, 132.33, 130.20, 130.10, 130.06, 129.95, 129.48, 129.17, 128.62, 128.54, 128.50, 128.01, 127.99, 125.42 (Ar), 87.42 (C-1), 78.74 (C-5), 77.98 (C-3), 73.94 (CH_2Ph), 71.07 (C-4), 70.40 (C-2), 70.18 (C-6), 34.56 (Cq, *t*-Bu), 31.39 (Me, t-Bu), 20.45 (Me). MS ESI-HRMS m/z $[M+Na]^+$ calcd for $C_{38}H_{40}O_7S$ 663,2387, found 663.2392.

(2-Methyl-5-tert-butylphenyl) 2,3-di-*O*-benzoyl-4-*O*-benzyl-6-*O*-fluorenylmethoxycarbonyl-1-thio-β-D-glucopyranoside 27

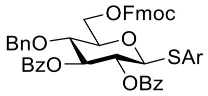

27

To a solution of compound **S6** (4.45 g, 6.94 mmol) was added 9-fluorenylmethyl chloroformate (3.59 g, 13.88 mmol) and pyridine (1.68 mL, 20.82 mmol) at 0 °C, and stirred at room temperature for overnight. After the mixture was quenched with 1M aqueous HCl, it was diluted with DCM. The organic layer was separated and aqueous phase was extracted twice with DCM. The combined organic layer was dried over $MgSO_4$ and the solvent was evaporated *in vacuo*. The crude product was purified by column chromatography on silica gel (hexane/ethyl acetate /DCM = 9:0.5:0.5 to 9:1:0.5) to afford **27** (5.74 g, 6.65 mmol, 96%). R_f = 0.18 (hexane/ethyl acetate /DCM = 9:1:0.5). $[\alpha]_D^{25}$ = 3.09 (c = 3.05, $CHCl_3$). IR (thin film): υ = 2960, 1755, 1732, 1278, 1249 cm^{-1}; ^1H NMR (400 MHz, $CDCl_3$) δ 7.98 (d, J = 8.4 Hz, 2H), 7.88 (d, J = 8.4 Hz, 2H), 7.71 (dd, J = 7.6, 3.3 Hz, 2H), 7.61 (d, J = 1.7 Hz, 1H), 7.56 – 7.50 (m, 1H), 7.46 – 7.20 (m, 16H), 7.17 (t, J = 7.5 Hz, 1H), 7.10 (d, J = 8.0 Hz, 1H), 5.80 (t, J = 9.5 Hz, 1H, H-3), 5.54 (t, J = 9.8 Hz, 1H, H-3), 5.25 (t, J = 9.8 Hz, 1H, H-4), 4.94 (d, J = 10.1 Hz, 1H, H-1), 4.58 (q, J = 12.2 Hz, 2H, CH_2Ph), 4.22 (dd, J = 10.4, 7.3 Hz, 1H, C*H*HPh, Fmoc), 4.08 (dd, J = 10.4, 7.8 Hz, 3H, H-6, C*H*HPh, Fmoc), 4.00 – 3.85 (m, 2H, H-5, CH, Fmoc), 3.74 (d, J = 3.9 Hz, 2H, H-6), 2.25 (s, 3H, Me), 1.26 (d, J = 0.8 Hz, 9H, *t*-Bu). ^{13}C NMR (100 MHz, $CDCl_3$) δ 165.82 (OBz), 165.22 (OBz), 154.17, 149.86, 143.34, 143.04, 141.25, 141.20, 137.79, 137.34, 133.39, 132.13, 130.32, 130.09, 130.00, 129.98, 129.35, 128.93, 128.49, 128.46, 128.40, 127.90, 127.79, 127.23, 127.23, 125.54, 125.28, 125.11, 120.02 (Ar), 87.61 (C-1), 77.34 (C-5), 74.58 (C-3), 73.75 (CH_2Ph), 73.38 (C-4), 70.79 (C-2), 70.43 (CH_2 of Fmoc), 69.02 (C-6), 46.55 (CH of Fmoc), 34.54 (Cq, *t*-Bu), 31.37 (Me, *t*-Bu), 20.46 (Me). MS ESI-HRMS m/z $[M+Na]^+$ calcd for $C_{53}H_{50}O_9S$ 885.3068, found 885.3094.

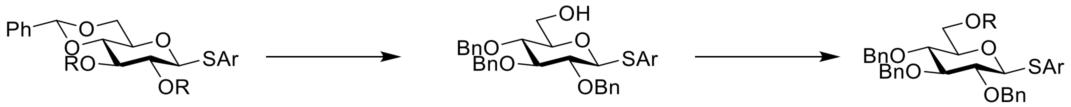

S7: R = H
S8: R = Bn
S9
29a: R = Bn
29b: R = Ac
29c: R = Bz
29d: R = Fmoc

2-Methyl-5-tert-butylphenyl 2,3-di-*O*-benzyl-4,6-*O*-benzylidene-1-thio-β-D-glucopyranoside S8

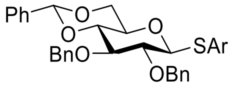

S8

To a solution of compound **S7**[2] (1.3 g, 3.02 mmol) in anhydrous DMF (15 mL, 0.2 M) was added BnBr (1.08 g, 9.06 mmol), NaH (0.604 g, 15.1 mmol) at 0 °C, and mixture was stirred for 2 h at room temperature. Then the mixture was quenched with saturated aqueous NH_4Cl, it was diluted with DCM. The organic layer was separated and aqueous phase was extracted twice with DCM. The combined organic layer was dried over $MgSO_4$ and the solvent was evaporated *in vacuo*. The crude product was purified by column chromatography on silica gel (hexane/ethyl acetate = 9:1 to 7:3) to afford **S8** (1.77 g, 2.90 mmol, 96%). R_f: 0.35 (hexane/ethyl acetate/DCM = 8:2:0.5). $[\alpha]_D^{25}$ = -2.75 (*c* = 3.33, $CHCl_3$). IR (thin film): υ = 2962, 1454, 1089 cm^{-1}; ^1H NMR (400 MHz, $CDCl_3$) δ 7.65 – 7.11 (m, 13H), 5.62 (s, 1H, C*H*HPh), 5.01 – 4.80 (m, 4H, 2 X CH_2Ph), 4.77 (d, *J* = 9.9 Hz, 1H, H-1), 4.36 (dd, *J* = 10.5, 4.8 Hz, 1H, H-6), 3.97 – 3.81 (m, 2H, H-3, H-6), 3.78 (t, *J* = 9.2 Hz, 1H, H-4), 3.60 (t, *J* = 9.0 Hz, 1H, H-2), 3.49 (td, *J* = 9.3, 4.9 Hz, 1H, H-5), 2.41 (s, 3H, Me), 1.30 (s, 9H, t-Bu). ^{13}C NMR (100 MHz, $CDCl_3$) δ 149.67, 138.49, 138.13, 137.36, 136.27, 132.89, 130.07, 129.12, 129.08, 128.51, 128.50, 128.40, 128.36, 128.22, 127.98, 127.88, 126.11, 124.88 (Ar), 101.28 (CHPh), 88.63 (C-1), 83.17 (C-3), 81.63 (C-4), 81.01 (C-2), 76.19 (CH_2Ph), 75.43 (CH_2Ph), 70.21 (C-5), 68.89 (C-6), 34.62 (Cq, t-Bu), 31.44 (Me, t-Bu), 20.46 (Me). MS ESI-HRMS m/z $[M+Na]^+$ calcd for $C_{38}H_{42}O_5S$ 633.2645, found 633.2644.

(2-Methyl-5-tert-butylphenyl) 2,3,4-tri-*O*-benzyl-1-thio-β-D-glucopyranoside S9

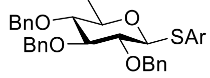

S9

Compound **S8** (1.81 g, 2.95 mmol) was co-evaporated with toluene and dissolved under an Ar atmosphere in DCM (78 mL, 0.2 M). To a solution of compound **S8** were added 1 M solution of BH_3 in THF (14.7 mL, 14.7 mmol), and TMSOTf (0.266 mL, 1.47 mmol) at 0 °C. After the

mixture was stirred for 5 h at 0 °C, the mixture was quenched with saturated aqueous $NaHCO_3$, and then diluted with DCM. The organic layer was separated and aqueous phase was extracted twice with DCM. The combined organic layer was dried over $MgSO_4$ and the solvent was evaporated *in vacuo*. The crude product was purified by column chromatography on silica gel (hexane/Ethyl acetate/DCM = 9:1:0.5 to 7:3:0.5) to afford **S9** (1.64 g, 2.68 mmol, 91%). R_f : 0.41 (hexane/Ethyl acetate/DCM = 7:3:0.5). $[\alpha]_D^{25}$ = 16.17 (*c* = 3.33, $CHCl_3$). IR (thin film): υ = 3471, 2961, 1071 cm^{-1}; ^1H NMR (400 MHz, $CDCl_3$) δ 7.54 (s, 2H), 7.43 – 7.23 (m, 15H), 7.20 (d, *J* = 8.0 Hz, 1H), 7.12 (d, *J* = 7.9 Hz, 1H), 4.89 (ddd, *J* = 38.1, 21.9, 10.2 Hz, 5H, 2 x CH_2Ph, C*H*HPh), 4.70 (d, *J* = 9.9 Hz, 1H, H-1), 4.66 (d, *J* = 10.8 Hz, 1H, C*H*HPh), 3.86 (d, *J* = 11.7 Hz, 1H, H-6), 3.77 – 3.66 (m, 2H, H-3, H-6), 3.62 (t, *J* = 9.4 Hz, 1H, H-4), 3.54 (t, *J* = 9.3 Hz, 1H, H-2), 3.43 – 3.33 (m, 1H, H-5), 2.39 (s, 3H, Me), 1.28 (s, 9H, *t*-Bu). ^{13}C NMR (100 MHz, $CDCl_3$) δ 149.62, 138.33, 137.88, 137.82, 135.83, 132.95, 129.94, 128.50, 128.44, 128.38, 128.23, 128.20, 128.04, 127.95, 127.86, 127.77, 127.71, 124.59 (Ar), 87.64 (C-1), 86.60 (C-3), 81.39 (C-2), 79.13 (C-5), 77.54 (C-4), 75.80 (CH_2Ph), 75.67 (CH_2Ph), 75.10 (CH_2Ph), 62.18 (C-6), 34.45 (Cq, *t*-Bu), 31.29 (Me, *t*-Bu), 20.24 (Me). MS ESI-HRMS m/z $[M+Na]^+$ calcd for $C_{38}H_{44}O_5S$ 635.2802, found 635.2795.

(2-Methyl-5-tert-butylphenyl) 2,3,4,6-tetra-*O*-benzyl-3-*O*-fluorenylmethoxycarbonyl-1-thio-β-D-glucopyranoside 29a

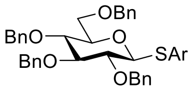

29a

Compound **S9** (0.2 g, 0.33 mmol), benzyl bromide (0.097 mL, 0.82 mmol), and sodium hydride (0.039 g, 0.979 mmol) were dissolved in anhydrous DMF (1.6 mL, 0.2 M) and stirred for 2 h at 0 °C. The mixture was quenched with saturated aqueous NH_4Cl, and then diluted with DCM. The organic layer was separated and aqueous phase was extracted twice with DCM. The combined organic layer was dried over $MgSO_4$ and the solvent was evaporated *in vacuo*. The crude product was purified by column chromatography on silica gel (hexane/Ethyl acetate/DCM = 9:0.5:0.5 to 9.0:1.0:0.5) to afford **29a** (0.22 g, 0.31 mmol, 96%). R_f = 0.48 (hexane/Ethyl acetate/DCM = 8:2:0.5). $[\alpha]_D^{25}$ = +9.43 (*c* = 3.50, $CHCl_3$). IR (thin film): υ = 2961, 1905, 1454, 1361, 1067 cm^{-1}; ^1H NMR (600 MHz, $CDCl_3$) 7.66 (d, *J* = 1.9 Hz, 1H), 7.45 – 7.23 (m, 18H), 7.19 – 7.05 (m, 4H), 4.97 (d, *J* = 10.3 Hz, 1H, C*H*HPh), 4.93 (d, *J* = 11.0 Hz, 1H, C*H*HPh), 4.86 (d, *J* = 11.0 Hz, 1H, C*H*HPh), 4.83 (d, *J* = 10.7 Hz, 1H, C*H*HPh), 4.79 (d, *J* = 10.3 Hz, 1H, C*H*HPh), 4.67 (d, *J* = 9.9 Hz, 1H, H-1), 4.59 (dd, *J* = 11.3, 9.8 Hz, 2H,

CH_2Ph), 4.53 (d, *J* = 12.3 Hz, 1H, C*H*HPh), 3.74 – 3.69 (m, 4H, H-3, H-4, H-6), 3.58 (dd, *J* = 9.7, 8.6 Hz 1H, H-2), 3.49 (ddd, *J* = 9.3, 3.9, 2.2 Hz, 1H, H-5), 2.40 (s, 3H), 1.26 (s, 9H, *t*-Bu). ^{13}C NMR (150 MHz, $CDCl_3$) δ 149.71, 138.64, 138.26, 138.19, 138.18, 135.92, 133.75, 129.86, 128.60, 128.57, 128.55, 128.51, 128.48, 128.37, 128.11, 128.04, 127.95, 127.91, 127.83, 127.80, 127.71, 124.39 (Ar), 88.18 (C-1), 86.99 (C-3), 81.39 (C-2), 79.17 (C-5), 77.96 (C-4), 75.92 (Bn), 75.71 (Bn), 75.20 (Bn), 73.61 (Bn), 69.01 (C-6), 34.61 (Cq, *t*-Bu), 31.43 (Me, *t*-Bu), 20.46 (Me). MS ESI-HRMS m/z $[M+Na]^+$ calcd for $C_{45}H_{50}O_5SNa$ 725.3277, found 725.3240

(2-Methyl-5-tert-butylphenyl) 3-*O*-Acetyl-2,4,6-tri-*O*-benzyl-1-thio-β-D-glucopyranoside 29b

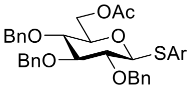

29b

To a solution of compound **S9** (0.2 g, 0.33 mmol) in anhydrous DCM (1.6 mL, 0.2 M) were added acetice anhydride (0.062 mL, 0.65 mmol), triethylamine (Et_3N) (0.18 mL, 1.31 mmol), and a catalytic amount of DMAP (8 mg, 0.065 mmol) at 0 °C and the mixture was stirred for 2 h at room temperature. After the mixture was quenched with saturated aqueous $NaHCO_3$, it was diluted with DCM. The organic layer was separated and aqueous phase was extracted twice with DCM. The combined organic layer was dried over $MgSO_4$ and the solvent was evaporated *in vacuo*. The crude product was purified by column chromatography on silica gel (hexane/Ethyl acetate/DCM = 9:1:0.5 to 7:3:0.5) to afford **29b** (0.2 g, 0.31 mmol, 94%). R_f = 0.27 (hexane/Ethyl acetate/DCM = 8:2:0.5). $[\alpha]_D^{25}$ = 23.49 (*c* = 3.07, $CHCl_3$). IR (thin film): υ = 2961, 1743, 1236, 1067 cm^{-1}; ^1H NMR (600 MHz, $CDCl_3$) δ 7.59 (d, *J* = 1.8 Hz, 1H), 7.40 (d, *J* = 7.0 Hz, 2H), 7.36 – 7.24 (m, 10H), 7.20 (dd, *J* = 8.0, 1.9 Hz, 1H), 7.13 (d, *J* = 8.0 Hz, 1H), 5.00 (d, *J* = 10.2 Hz, 1H, C*H*HPh), 4.96 (d, *J* = 10.9 Hz, 1H, C*H*HPh), 4.88 (d, *J* = 10.8, 1H, C*H*HPh), 4.87 (d, *J* = 10.9, 1H, C*H*HPh), 4.80 (d, *J* = 10.2 Hz, 1H, C*H*HPh), 4.66 (d, *J* = 9.9 Hz, 1H, H-1), 4.59 (d, *J* = 10.8 Hz, 1H, C*H*HPh), 4.34 (dd, *J* = 11.9, 1.5 Hz, 1H, H-6), 4.24 (dd, *J* = 12.0, 5.0 Hz, 1H, H-6), 3.74 (t, *J* = 8.7 Hz, 1H, H-3), 3.65 – 3.49 (m, 3H, H-2, H-4, H-5), 2.39 (s, 3H, Me), 2.03 (s, 3H, Ac), 1.30 (s, 9H, *t*-Bu). ^{13}C NMR (150 MHz, $CDCl_3$) δ 170.90 (Ac), 149.69, 138.40, 138.04, 137.72, 136.25, 133.33, 130.00, 128.69, 128.67, 128.63, 128.54, 128.34, 128.24, 128.18, 128.02, 127.91, 124.71 (Ar), 88.38 (C-1), 86.91 (C-3), 81.32 (C-2), 76.96 (C-5), 75.96 (CH_2Ph), 75.77 (CH_2Ph), 75.27 (CH_2Ph), 63.66 (C-6), 34.61 (Cq, t-Bu),

31.48 (Me, t-Bu), 21.06 (Ac), 20.44 (Me). MS ESI-HRMS m/z $[M+Na]^+$ calcd for $C_{40}H_{46}O_6S$ 677.2907, found 677.2930

(2-Methyl-5-tert-butylphenyl) 3-*O*-Benzoyl-2,4,6-tri-*O*-benzyl-1-thio-β-D-glucopyranoside 29c

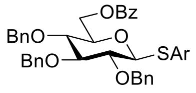

29c

To a solution of compound **S9** (0.2 g, 0.33 mmol) in anhydrous DCM (1.6 mL, 0.2 M) were added benzoic anhydride (0.15 g, 0.65 mmol), triethylamine (Et_3N, 0.18 mL, 1.31 mmol), and a catalytic amount of DMAP (8 mg, 0.065 mmol) at 0 °C and the mixture was stirred for 2 h at room temperature. After the mixture was quenched with saturated aqueous $NaHCO_3$, it was diluted with DCM. The organic layer was separated and aqueous phase was extracted twice with DCM. The combined organic layer was dried over $MgSO_4$ and the solvent was evaporated *in vacuo*. The crude product was purified by column chromatography on silica gel (hexane/Ethyl acetate/DCM = 9:1:0.5 to 7:3:0.5) to afford **29c** (0.23 g, 0.32 mmol, 98%). R_f = 0.31 (hexane/Ethyl acetate/DCM = 8:2:0.5). $[\alpha]_D^{25}$ = 18.84 (*c* = 2.98, $CHCl_3$). IR (thin film): υ = 2961, 1722, 1272, 1068 cm^{-1}; ^1H NMR (600 MHz, $CDCl_3$) δ 7.99 (dd, *J* = 8.2, 1.1 Hz, 2H), 7.62 – 7.53 (m, 2H), 7.43 (m, 4H), 7.38 – 7.21 (m, 13H), 7.19 (dd, *J* = 7.9, 2.0 Hz, 1H), 7.09 (d, *J* = 7.9 Hz, 1H), 5.04 (d, *J* = 10.2 Hz, 1H, C*H*HPh), 4.97 (d, *J* = 10.8 Hz, 1H, C*H*HPh), 4.89 (d, *J* = 10.7 Hz, 1H, C*H*HPh), 4.88 (d, *J* = 10.8 Hz, 1H, C*H*HPh), 4.84 (d, *J* = 10.2 Hz, 1H, C*H*HPh), 4.69 (d, *J* = 9.9 Hz, 1H, H-1), 4.62 (d, *J* = 10.7 Hz, 1H, C*H*HPh), 4.58 (dd, *J* = 12.0, 1.9 Hz, 1H, H-6), 4.49 (dd, *J* = 12.0, 4.5 Hz, 1H, H-6), 3.78 (t, *J* = 8.8 Hz, 1H, H-3), 3.74 (t, *J* = 9.2 Hz, 1H, H-4), 3.65 – 3.63 (m, 1H, H-5), 3.61 (dd, *J* = 9.6, 8.8 Hz, 1H, H-2), 2.39 (s, 3H, Me), 1.24 (s, 9H, *t*-Bu). ^{13}C NMR (150 MHz, $CDCl_3$) δ 166.36 (Bz), 149.67, 138.36, 138.09, 137.69, 136.99, 133.18, 132.88, 130.03, 129.93, 129.66, 128.67, 128.65, 128.57, 128.48, 128.34, 128.23, 128.14, 128.03, 127.98, 125.03, 88.71 (C-1), 86.97 (C-3), 81.65 (C-2), 77.72 (C-4), 77.13 (C-5), 76.11 (CH_2Ph), 75.82 (CH_2Ph), 75.37 (CH_2Ph), 63.83 (C-6), 34.54 (Cq, *t*-Bu), 31.42 (Me, *t*-Bu), 20.59 (Me). MS ESI-HRMS m/z $[M+Na]^+$ calcd for $C_{45}H_{48}O_6S$ 739.3064, found 739.3093.

(2-Methyl-5-tert-butylphenyl) 2,4,6-tri-*O*-Benzyl-3-*O*-fluorenylmethoxycarbonyl-1-thio-β-D-glucopyranoside 29d

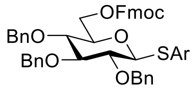

29d

To a solution of compound **S9** (0.2 g, 0.33 mmol) in anhydrous DCM (1.6 mL, 0.2 M) were added 9-fluorenylmethyl chloroformate (0.13 g, 0.49 mmol) and pyridine (0.053 mL, 0.65 mmol) at 0 °C, and the mixture was stirred for 3hr at room temperature. Then the mixture was quenched with 1M aqueous HCl, and then diluted with DCM. The organic layer was separated and aqueous phase was extracted twice with DCM. The combined organic layer was dried over $MgSO_4$ and the solvent was evaporated *in vacuo*. The crude product was purified by column chromatography on silica gel (hexane/Ethyl acetate/DCM = 9:0.5:0.5 to 9:1:0.5) to afford **29d** (0.26 g, 0.31 mmol, 95%). R_f = 0.35 (hexane/Ethyl acetate/DCM = 8:2:0.5). $[\alpha]_D^{25}$ = 19.11 (*c* = 3.05, $CHCl_3$). IR (thin film): υ = 2960, 1748, 1452, 1254, 1066 cm^{-1}; ^1H NMR (600 MHz, $CDCl_3$) δ 7.77 (dd, *J* = 7.5, 2.8 Hz, 2H), 7.66 – 7.57 (m, 3H), 7.43 – 7.37 (m, 4H), 7.35 – 7.24 (m, 15H), 7.19 (dd, *J* = 7.9, 1.9 Hz, 1H), 7.11 (d, *J* = 8.0 Hz, 1H), 5.00 (d, *J* = 10.3 Hz, 1H, C*H*HPh), 4.96 (d, *J* = 10.9 Hz, 1H, C*H*HPh), 4.89 (d, *J* = 10.9 Hz, 1H, C*H*HPh), 4.87 (d, *J* = 10.9 Hz, 1H, C*H*HPh), 4.80 (d, *J* = 10.2 Hz, 1H, C*H*HPh), 4.68 (d, *J* = 9.9 Hz, 1H, H-1), 4.61 (d, *J* = 10.9 Hz, 1H, C*H*HPh), 4.45 (dd, *J* = 11.6, 1.6 Hz, 1H, H-6), 4.38 (ddd, *J* = 24.2, 10.5, 7.5 Hz, 2H, CH_2Ph of Fmoc), 4.32 (dd, *J* = 11.6, 5.1 Hz, 1H, H-6), 4.24 (t, *J* = 7.4 Hz, 1H, CH of Fmoc), 3.75 (t, *J* = 8.7 Hz, 1H, H-3), 3.64 (t, *J* = 9.2 Hz, 1H, H-4), 3.61 – 3.54 (m, 2H, H-2, H-5), 2.39 (s, 3H, Me), 1.28 (s, 9H, *t*-Bu). ^{13}C NMR (150 MHz, $CDCl_3$) δ 155.21 (Fmoc), 149.82, 143.56, 143.48, 141.42, 138.43, 138.07, 137.75, 136.29, 133.29, 130.00, 128.92, 128.68, 128.64, 128.56, 128.35, 128.22, 128.15, 128.02, 127.92, 127.34, 127.32, 125.39, 125.34, 124.80, 120.18 (Ar), 88.46 (C-1), 86.89 (C-3), 81.29 (C-5), 77.55 (C-4), 76.88 (C-2), 75.98 (CH_2Ph), 75.78 (CH_2Ph), 75.35 (CH_2Ph), 70.12 (CH_2 of Fmoc), 67.01 (C-6), 46.86 (CH of Fmoc), 34.63 (Cq, *t*-Bu), 31.48 (Me, *t*-Bu), 20.48 (Me). MS ESI-HRMS m/z $[M+Na]^+$ calcd for $C_{45}H_{48}O_6S$ 857.3482, found 857.3505.

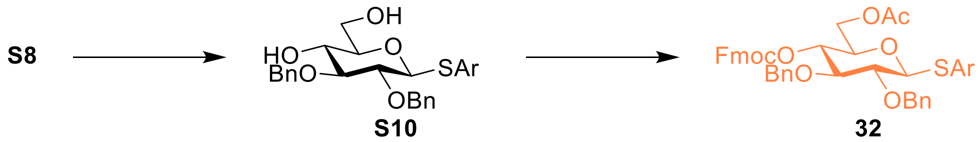

(2-Methyl-5-tert-butylphenyl) 2,3-di-*O*-benzyl-1-thio-β-D-glucopyranoside S10

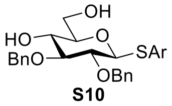

A solution of compound **S8** (4.0 g, 6.55 mmol) in DCM/Water/TFA (62 mL: 4 mL: 16 mL, 0.08 M) was stirred for 2 h at 0 °C. After the reaction was completed, saturated aqueous $NaHCO_3$ was added and the aqueous layers were extracted three times with DCM. The organic layer was separated and aqueous phase was extracted twice with DCM. The combined organic layer was dried over $MgSO_4$ and the solvent was evaporated *in vacuo*. The crude product was purified by column chromatography on silica gel (hexane/Ethyl acetate/DCM = 8:2:0.5 to 5:5:0.5) to afford **S10** (2.8 g, 5.36 mmol, 82%). R_f = 0.35 (hexane/Ethyl acetate/DCM = 8:2:0.5). $[\alpha]_D^{25}$ = 2.02 (*c* = 2.34, $CHCl_3$). IR (thin film): υ = 3449, 2964, 1455, 1071 cm^{-1}; ^1H NMR (400 MHz, $CDCl_3$) δ 7.56 (d, *J* = 1.8 Hz, 1H), 7.45 – 7.26 (m, 9H), 7.22 (dd, *J* = 8.0, 1.9 Hz, 1H), 7.14 (d, *J* = 8.0 Hz, 1H), 5.03 (d, *J* = 10.9 Hz, 1H, C*H*HPh), 4.86 (d, *J* = 11.2 Hz, 1H, C*H*HPh), 4.76 – 4.70 (m, 2H, 2 x C*H*HPh), 4.69 (d, *J* = 6.2 Hz, 1H, H-1), 3.89 (dd, *J* = 12.0, 2.6 Hz, 1H, H-6), 3.81 (t, *J* = 8.8 Hz, 1H, H-4), 3.73 (dd, *J* = 12.1, 4.7 Hz, 1H, H-6), 3.53 (t, *J* = 9.3 Hz, 1H, H-3), 3.46 – 3.34 (m, 2H, H-2, H-5), 2.40 (s, 3H, Me), 1.29 (s, 9H, *t*-Bu). ^{13}C NMR (100 MHz, $CDCl_3$) δ 149.82, 138.15, 138.03, 135.85, 133.01, 130.14, 128.75, 128.71, 128.39, 128.26, 128.25, 128.21, 128.17, 124.75 (Ar), 87.25 (C-1), 81.06 (C-2), 79.02 (C-5), 78.74 (C-4), 75.47 (C-3), 74.88 (CH_2Ph), 62.40 (CH_2Ph), 34.62 (Cq, *t*-Bu), 31.45 (Me, *t*-Bu), 20.37 (Me). MS ESI-HRMS m/z $[M+Na]^+$ calcd for $C_{31}H_{38}O_5S$ 545.2332, found 545.2276.

(2-Methyl-5-tert-butylphenyl) 6-*O*-Acetyl-2,3-di-*O*-benzyl-4-*O*-fluorenylmethoxycarbonyl-1-thio-β-D-glucopyranoside 32

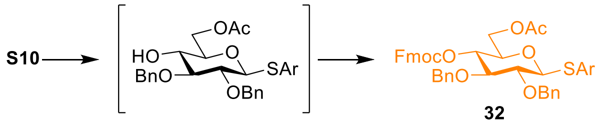

To a solution of compound **S10** (1.2 g, 2.33 mmol), 2-Chloro-1-methylpyridium iodide (1.49 mL, 5.84 mmol), DABCO (1.05 g, 9.34 mmol) in anhydrous DCM (23 mL, 0.1 M) was added acetic acid (0.15 mL, 2.57 mmol) slowly at -15 °C and the mixture was stirred for 2 h at -15 °C. After the mixture was quenched using saturated aqueous $NaHCO_3$, it was diluted with DCM. The organic layer was separated and aqueous phase was extracted twice with DCM. The

combined organic layer was dried over $MgSO_4$ and the solvent was evaporated *in vacuo*. To a solution of the crude product in anhydrous DCM (11 mL) was added 9-fluorenylmethyl chloroformate (1.10 g, 4.25 mmol) and pyridine (0.52 mL, 6.37 mmol) at 0 °C, and stirred at room temperature for overnight. After the mixture was quenched with 1M aqueous HCl, it was diluted with DCM. The organic layer was dried over $MgSO_4$ and the solvent was evaporated *in vacuo*. The crude product was purified by column chromatography on silica gel (hexane/Ethyl acetate/DCM = 9:0.5:0.5 to 8:2:0.5) to afford **32** (1.54 g, 1.96 mmol, 84% over two steps). R_f = 0.35 (hexane/Ethyl acetate/DCM = 8:2:0.5). $[\alpha]_D^{25}$ = 7.41 (*c* = 2.65, $CHCl_3$). IR (thin film): υ = 2962, 1750, 1258, 1037 cm^{-1}; ^1H NMR (600 MHz, $CDCl_3$) δ 7.67 (d, *J* = 7.6 Hz, 1H), 7.53 (d, *J* = 1.9 Hz, 1H), 7.50 (d, *J* = 7.5 Hz, 1H), 7.46 (d, *J* = 7.5 Hz, 1H), 7.33 – 7.13 (m, 8H), 7.06 (d, *J* = 8.0 Hz, 1H), 4.89 (d, *J* = 9.7 Hz, 1H), 4.91 – 4.86 (m, 1H, H-4), 4.74 (d, *J* = 11.2 Hz, 1H, C*H*HPh), 4.70 (d, *J* = 10.3 Hz, 1H, C*H*HPh), 4.59 (d, *J* = 10.4 Hz, 1H, C*H*HPh), 4.57 (d, *J* = 9.7 Hz, 1H, H-1, C*H*HPh), 4.37 (dd, *J* = 10.5, 7.1 Hz, 1H, C*H*H, Fmoc), 4.26 – 4.21 (m, 2H, H-6, , C*H*H, Fmoc), 4.09 (m, 2H, H-6, CH of Fmoc), 3.66 (t, *J* = 9.1 Hz, 1H, H-3), 3.58 (ddd, *J* = 10.0, 5.0, 2.4 Hz, 1H, H-5), 3.56 – 3.50 (m, 1H, H-2), 2.33 (s, 3H, Me), 1.96 (s, 3H, Me of Ac), 1.23 (s, 9H, *t*-Bu). ^{13}C NMR (150 MHz, $CDCl_3$) δ 170.82 (Ac), 154.43 (Fmoc), 149.75, 143.40, 143.26, 141.41, 141.39, 137.94, 137.88, 136.72, 132.82, 130.11, 129.42, 128.56, 128.44, 128.39, 128.08, 128.04, 128.02, 127.85, 127.83, 127.32, 125.23, 125.10, 125.09, 120.20, 120.18 (Ar), 88.61 (C-1), 83.99 (C-3), 80.95 (C-2), 75.83 (CH_2Ph), 75.76 (CH_2Ph), 75.52 (C-5), 74.41 (C-4), 70.41 (CH_2 of Fmoc), 62.82 (C-6), 46.82 (CH of Fmoc), 34.62 (Cq, *t*-Bu), 31.49 (Me, *t*-Bu), 20.95 (Me of Ac), 20.52 (Me). MS ESI-HRMS m/z $[M+Na]^+$ calcd for $C_{48}H_{50}O_8S$ 809.3119, found 809.3137.

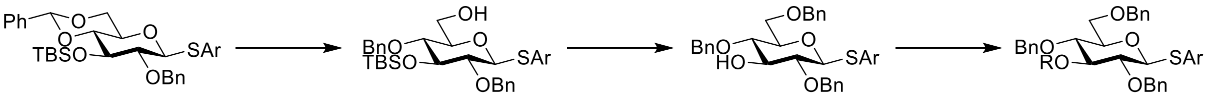

S11 **S12** **S13**

29e: R = Ac
29f: R = Bz
29g: R = Fmoc

(2-Methyl-5-tert-butylphenyl) 2-*O*-benzyl-4,6-*O*-benzylidene-3-*O*-tert-butyldimethylsilyl-1-thio-β-D-glucopyranoside S11

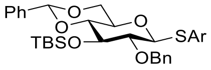

S11

To a solution of compound **S2** (9 g, 20.9 mmol) in anhydrous DCM (21 mL, 1.0 M) was added TBSCl (3.78 g, 25.mmol), imidazole (1.99 g, 29.3 mmol) dropwise at 0 °C, the mixture was

gradually wormed up to room temperature, and stirred at room temperature for overnight. After the mixture was quenched with MeOH, it was diluted with DCM. The organic layer was separated and aqueous phase was extracted twice with DCM. The combined organic layer was dried over $MgSO_4$ and the solvent was evaporated *in vacuo*. To a solution of this crude product in anhydrous DMF (91 mL, 0.2 M) were added benzyl bromide (7.46 mL, 62.7 mmol, 3.0 eq.) and sodium hydride (2.01 g, 50.2 mmol, 2.4 eq.) at 0 °C for 2 h. The mixture was quenched by saturated aqueous NH_4Cl and then diluted with DCM. The organic layer was dried over $MgSO_4$ and the solvent was evaporated *in vacuo*. The crude product passed through silica pad, then utilized for the next reaction.

(2-Methyl-5-tert-butylphenyl) 2,4-di-*O*-benzyl-3-*O*-tert-butyldimethylsilyl-1-thio-β-D-glucopyranoside S12

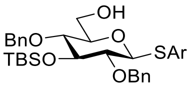

S12

The crude **S11** was co-evaporated with toluene and dissolved under an Ar atmosphere in DCM (53 mL, 0.2 M). To a solution of the crude **S11** were added 1 M solution of BH_3 in THF (52.6 mL, 52.6 mmol), and TMSOTf (0.95 mL, 5.26 mmol) at 0 °C and the mixture was stirred for 5 h at 0 °C. After completion, the mixture was quenched with saturated aqueous $NaHCO_3$, and then diluted with DCM. The organic layer was separated and aqueous phase was extracted twice with DCM. The combined organic layer was dried over $MgSO_4$ and the solvent was evaporated *in vacuo*. The crude product was purified by column chromatography on silica gel (hexane/ethyl acetate/DCM = 9:1:0.5 to 7:3:0.5) to afford **S12** (6.45 g, 10.1 mmol, 77%) over three steps. R_f : 0.27 (Hexane/ethyl acetate/DCM = 8:2:0.5). $[\alpha]_D^{25}$ = 42.23 (C= 2.00, $CHCl_3$). IR (thin film): υ = 3476, 2958, 1732, 1263, 1090 cm^{-1}; ^{1}H NMR (400 MHz, $CDCl_3$) δ 7.52 – 7.26 (m, 11H), 7.23 – 7.08 (m, 2H), 4.98 (d, *J* = 9.8 Hz, 1H, C*H*HPh), 4.88 (d, *J* = 11.5 Hz, 1H, C*H*HPh), 4.79 (d, *J* = 10.6 Hz, 1H, C*H*HPh), 4.74 (d, *J* = 9.8 Hz, 1H, H-1), 4.63 (d, *J* = 9.4 Hz, 1H, C*H*HPh), 3.86 – 3.75 (m, 2H, H-4, H-6), 3.64 (m, 1H, H-6), 3.49 (t, *J* = 9.2 Hz, 1H, H-3), 3.44 – 3.34 (m, 2H, H-2, H-5), 2.32 (s, 3H, Me), 1.28 (s, 9H, *t*-Bu), 0.96 (s, 9H, TBS), 0.05 (s, 3H, TBS), 0.00 (s, 3H, TBS). ^{13}C NMR (100 MHz, $CDCl_3$) δ 138.28, 138.12, 135.54, 129.93, 128.42, 127.98, 127.75, 127.62, 127.39 (Ar), 87.72 (C-1), 81.93 (C-2), 79.22 (C-5), 78.71 (C-4), 78.66 (C-3), 75.86 (CH_2Ph), 75.36 (CH_2Ph), 61.60 (C-6), 34.53 (Cq, t-Bu), 31.52 (t-Bu), 26.29 (TBS), 20.11 (Me), 18.13 (Cq, TBS), -3.81 (TBS), -3.89 (TBS). MS ESI-HRMS m/z $[M+Na]^+$ calcd for $C_{37}H_{52}O_5S$ 659.3197, found 659.3185.

2-Methyl-5-tert-butylphenyl 2,4,6-tri-*O*-benzyl-1-thio-β-D-glucopyranoside S13

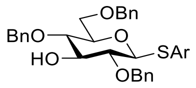

S13

To a solution of compound **S12** (1.9 g, 2.98 mmol) in co-solvent of THF, and DMF (9:1) was added benzyl bromide (1.06 mL, 8.95 mmol), and sodium hydride (0.24 mg, 5.97 mmol) dropwise at 0 °C. The mixture was stirred for 2 h at 0 °C. After completion the mixture was quenched with saturated aqueous NH_4Cl, and then was diluted with DCM. The organic layer was dried over $MgSO_4$ and the solvent was evaporated *in vacuo*. To a solution of this crude in anhydrous acetonitrile (37 mL, 0.08 M) was added boron trifluoride etherate ($BF_3{\cdot}OEt_2$) (0.38 mL, 2.98 mmol) at 0 °C and the mixture was stirred at 0 °C for 20 min. After completion the mixture was quenched with saturated aqueous $NaHCO_3$ and then it was diluted with DCM. The organic layer was separated and aqueous phase was extracted twice with DCM. The combined organic layer was dried over $MgSO_4$ and the solvent was evaporated *in vacuo*. The crude product was purified by column chromatography on silica gel (hexane/ethyl acetate/DCM = 9:1:1 to 7:3:1) to afford **S13** (1.65 g, 2.69 mmol, 90% over two steps). R_f = 0.36 (Hexane/ethyl acetate/DCM = 8:2:0.5). $[\alpha]_D^{25}$ = +0.43 (C= 3.50, $CHCl_3$). IR (thin film): υ = 3447, 3033, 2963, 2869, 1455, 1069 cm^{-1}; ^1H NMR (400 MHz, $CDCl_3$) δ 7.68 (d, *J* = 1.9 Hz, 1H), 7.42 – 7.07 (m, 17H), 5.01 (d, *J* = 11.0 Hz, 1H, C*H*HPh), 4.81 (d, *J* = 11.1 Hz, 1H, C*H*HPh), 4.71 (d, *J* = 10.9 Hz, 1H, C*H*HPh), 4.65 (d, *J* = 9.9 Hz, 1H, H-1), 4.62 (d, *J* = 10.1 Hz, 1H, C*H*HPh), 4.60 (d, *J* = 8.8 Hz, 1H, CHHPh), 4.54 (d, *J* = 12.3 Hz, 1H, C*H*HPh), 3.82 – 3.69 (m, 3H, H-3, H-6), 3.60 (t, *J* = 9.3 Hz, 1H, H-4), 3.52 – 3.43 (m, 2H, H-2, H-5), 2.41 (s, 3H), 1.27 (s, 9H). ^{13}C NMR (100 MHz, $CDCl_3$) δ 149.75, 138.32, 138.16, 135.75, 133.61, 129.90, 128.71, 128.60, 128.52, 128.47, 128.40, 128.19, 128.13, 128.08, 127.98, 127.75, 124.38 (Ar), 87.58 (C-1), 80.75 (C-2), 78.86 (C-5), 78.84 (C-3), 77.40 (C-4), 75.27 (Bn), 74.78 (Bn), 73.60 (Bn), 68.95 (C-6), 34.61 (Cq, t-Bu), 31.42 (Me, t-Bu), 20.43 (Me). MS ESI-HRMS m/z $[M+Na]^+$ calcd for $C_{38}H_{44}O_5SNa$ 635.2802, found 635.2795.

2-Methyl-5-tert-butylphenyl 3-*O*-Acetyl-2,4,6-tri-*O*-benzyl-1-thio-β-D-glucopyranoside 29e

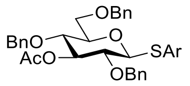

29e

To a solution of compound **S13** (0.2 g, 0.33 mmol) in anhydrous DCM (1.6 mL, 0.2 M) were added acetice anhydride (0.062 mL, 0.65 mmol), triethylamine (Et_3N, 0.18 mL, 1.31 mmol), and a catalytic amount of DMAP (8 mg, 0.065 mmol) at 0 °C, the mixture was stirred for 2 h at

room temperature. After completion the mixture was quenched with saturated aqueous $NaHCO_3$ and then diluted with DCM. The organic layer was separated and aqueous phase was extracted twice with DCM. The combined organic layer was dried over $MgSO_4$ and the solvent was evaporated *in vacuo*. The crude product was purified by column chromatography on silica gel (hexane/ethyl acetate = 9:1 to 7:3) to afford **29e** (0.19 g, 0.29 mmol, 89%). R_f = 0.34 (Hexane/ethyl acetate/DCM = 8:2:0.5). $[\alpha]_D^{25}$ = 3.20 (*c* = 3.00, $CHCl_3$). IR (thin film): υ = 2960, 1748, 1361, 1225, 1087 cm^{-1}; ^1H NMR (400 MHz, $CDCl_3$) 7.65 (d, *J* = 1.7 Hz, 1H), 7.41 – 7.04 (m, 17H), 5.28 (t, *J* = 9.3 Hz, 1H, H-3), 4.88 (d, *J* = 11.1 Hz, 1H, C*H*HPh), 4.68 (d, *J* = 9.8 Hz, 1H, H-1), 4.63 (d, *J* = 12.2 Hz, 1H, C*H*HPh), 4.57 (d, *J* = 11.1 Hz, 1H, C*H*HPh), 4.53 (d, *J* = 12.2 Hz, 1H, C*H*HPh), 4.51 (s, 2H, CH_2Ph), 3.76 – 3.68 (m, 3H, H-4, H-6), 3.59 – 3.46 (m, 2H, H-2, H-5), 2.40 (s, 3H, Me), 1.83 (s, 3H, Ac), 1.26 (s, 9H, *t*-Bu). ^{13}C NMR (100 MHz, $CDCl_3$) δ 169.86 (Ac), 149.52, 137.85, 137.70, 137.67, 136.04, 132.93, 129.79, 128.91, 128.35, 128.31, 128.16, 128.12, 127.93, 127.88, 127.75, 127.72, 127.63, 124.49 (Ar), 87.86 (C-1), 78.71 (C-2), 78.70 (C-5), 76.67 (C-3), 75.79 (C-4), 74.66 (Bn), 74.27 (Bn), 73.47 (Bn), 68.39 (C-6), 34.39 (Cq, *t*-Bu), 31.22 (Me, *t*-Bu), 20.93 (Me, Ac), 20.29 (Me). MS ESI-HRMS m/z $[M+Na]^+$ calcd for $C_{40}H_{46}O_6SNa$ 677.2913, found 677.2882.

2-Methyl-5-tert-butylphenyl 3-*O*-Benzoyl-2,4,6-tri-*O*-benzyl-1-thio-β-D-glucopyranoside 29f

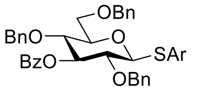

29f

To a solution of compound **S13** (0.2 g, 0.33 mmol) in anhydrous DCM (1.6 mL, 0.2 M) were added benzoic anhydride (0.15 g, 0.65 mmol), triethylamine (Et_3N, 0.18 mL, 1.31 mmol, 4.0 eq.), and a catalytic amount of DMAP (8 mg, 0.065 mmol) at 0 °C and the mixture was stirred for 2 h at room temperature. After completion the mixture was quenched with saturated aqueous $NaHCO_3$ and then diluted with DCM. The organic layer was separated and aqueous phase was extracted twice with DCM. The combined organic layer was dried over $MgSO_4$ and the solvent was evaporated *in vacuo*. The crude product was purified by column chromatography on silica gel (hexane/ethyl acetate = 9:1 to 7:3) to afford **29f** (0.21 g, 0.29 mmol, 90%). R_f = 0.40 (hexane/ethyl acetate/DCM = 7:3:0.5). $[\alpha]_D^{25}$ = 18.06 (*c* = 3.20, $CHCl_3$). IR (thin film): υ = 2960, 2867, 1728, 1453, 1266, 1088, 1069 cm^{-1}; ^1H NMR (400 MHz, $CDCl_3$) 7.97 (dd, *J* = 8.4, 1.2 Hz, 2H), 7.68 (s, 1H), 7.61 – 7.54 (m, 1H), 7.35 (dddd, *J* = 12.7, 11.5, 10.2, 5.6 Hz, 7H), 7.23 – 6.95 (m, 12H), 5.57 (t, *J* = 9.2 Hz, 1H, H-3), 4.82 (d, *J* = 10.7 Hz, 1H, C*H*HPh), 4.76 (d,

J = 9.8 Hz, 1H, H-1), 4.65 (d, J = 12.2 Hz, 1H, C*H*HPh), 4.56 (d, J = 10.6 Hz, 1H, C*H*HPh), 4.55 (d, J = 12.4 Hz, 1H, C*H*HPh), 4.51 – 4.42 (m, 2H, CH_2Ph), 3.88 (t, J = 9.5 Hz, 1H, H-4), 3.76 (s, 2H, H-6), 3.70 (t, J = 9.5 Hz, 1H, H-2), 3.58 (d, J = 9.8 Hz, 1H, H-5), 2.41 (s, 3H, Me), 1.27 (s, 9H, *t*-Bu). ^{13}C NMR (100 MHz, $CDCl_3$) δ 165.55 (Bz), 149.69, 138.03, 137.49, 137.41, 136.17, 133.22, 133.16, 130.07, 129.96, 129.84, 129.01, 128.54, 128.50, 128.48, 128.33, 128.26, 128.23, 128.13, 127.83, 127.81, 127.79, 124.63 (Ar), 88.12 (C-1), 78.87 (C-5), 78.69 (C-2), 78.18 (C-3), 75.83 (C-4), 74.90 (Bn), 74.57 (Bn), 73.68 (Bn), 68.61 (C-6), 34.58 (Cq, *t*-Bu), 31.40 (Me, *t*-Bu), 20.47 (Me). MS ESI-HRMS m/z $[M+Na]^+$ calcd for $C_{45}H_{48}O_6SNa$ 739.3069, found 739.3085.

(2-Methyl-5-tert-butylphenyl) 2,4,6-tri-*O*-benzyl-3-*O*-fluorenylmethoxycarbonyl-1-thio-β-D-glucopyranoside 29g

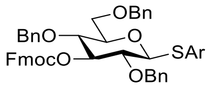

29g

To a solution of compound **S13** (0.2 g, 0.33 mmol) in anhydrous DCM (1.6 mL, 0.2 M) were added 9-fluorenylmethyl chloroformate (0.13 g, 0.49 mmol) and pyridine (0.053 mL, 0.65 mmol) successively at 0 °C and the mixture was stirred for 3hr at room temperature. After completion the mixture was quenched with 1M aqueous HCl and then diluted with DCM. The organic layer was separated and aqueous phase was extracted twice with DCM. The combined organic layer was dried over $MgSO_4$ and the solvent was evaporated *in vacuo*. The crude product was purified by column chromatography on silica gel (hexane/ethyl acetate/DCM = 9:0.5:0.5 to 9:1:0.5) to afford **29g** (0.24g, 0.29 mmol, 88%). R_f = 0.51 (hexane/ethyl acetate/DCM : 7:3:0.5). $[\alpha]_D^{25}$ = 9.40 (c = 3.21, $CHCl_3$). IR (thin film): υ = 2954, 1752, 1451, 1252, 1072 cm^{-1}; ^1H NMR (400 MHz, $CDCl_3$) δ 7.75 (d, J = 7.6 Hz, 2H), 7.64 (d, J = 1.9 Hz, 1H), 7.56 (d, J = 7.5 Hz, 2H), 7.41 – 7.16 (m, 23H), 7.14 – 7.09 (m, 3H), 5.12 (t, J = 9.3 Hz, 1H, H-3), 4.93 (d, J = 10.8 Hz, 1H, CHHPh), 4.68 (d, J = 9.9 Hz, 1H, H-1), 4.65 – 4.50 (m, 5H, 3 X C*H*HPh, CH_2Ph), 4.36 – 4.24 (m, 2H, CH_2, Fmoc), 4.13 (t, J = 7.2 Hz, 1H, CH, Fmoc), 3.79 (t, J = 9.6 Hz, 1H, H-4), 3.71 (d, J = 2.8 Hz, 2H, H-6), 3.62 (t, J = 9.5 Hz, 1H, H-2), 3.51 (dt, J = 9.8, 2.7 Hz, 1H, H-5), 2.40 (s, 3H, Me), 1.25 (s, 9H, *t*-Bu). ^{13}C NMR (100 MHz, $CDCl_3$) δ 154.77 (Fmoc), 149.74, 143.47, 143.42, 141.36, 138.05, 137.72, 136.33, 133.05, 129.98, 129.19, 128.51, 128.44, 128.37, 128.18, 128.07, 127.97, 127.90, 127.87, 127.81, 127.28, 125.21, 124.78, 120.15 (Ar), 88.01 (C-1), 82.71 (C-3), 79.04 (C-3), 78.84 (C-5), 75.92 (C-4), 75.21 (Bn), 74.79 (Bn), 73.66 (Bn), 70.19 (CH_2, Fmoc), 68.62 (C-6), 46.82 (CH, Fmoc), 34.59

(Cq, *t*-Bu), 31.41 (Me, *t*-Bu), 20.50 (Me). MS ESI-HRMS m/z $[M+Na]^+$ calcd for $C_{53}H_{54}O_7SNa$ 857.3488, found 733.3177 .

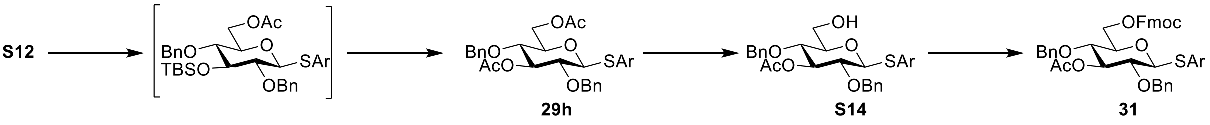

(2-Methyl-5-tert-butylphenyl) 3,6-di-*O*-Acetyl-2,4-di-*O*-benzyl-1-thio-β-D-glucopyranoside 29h

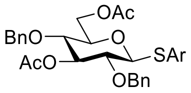

29h

To a solution of compound **S12** (3.6 g, 5.65 mmol) in anhydrous acetonitrile (71 mL, 0.08 M) was added boron trifluoride etherate ($BF_3 \cdot OEt_2$) (0.79 mL, 6.22 mmol) at 0 °C and the mixture was stirred for 20 min at 0 °C. After completion the mixture was quenched with saturated aqueous $NaHCO_3$ and then diluted with DCM. The organic layer was dried over $MgSO_4$ and the solvent was evaporated *in vacuo*. To a solution of the resulting crude protuct in anhydrous DCM (28 mL, 0.2 M) was added acetic anhydride (2.13 mL, 22.58 mmol), and triethylamine (4.72 mL, 33.9 mmol), and a catalytic amount of DMAP (0.069 g, 0.564 mmol) at 0 °C and the mixture was stirred for 1 h at 0 °C. After completion the mixture was quenched saturated aqueous $NaHCO_3$ and then diluted with DCM. The organic layer was separated and aqueous phase was extracted twice with DCM. The combined organic layer was dried over $MgSO_4$ and the solvent was evaporated *in vacuo*. The crude product was purified by column chromatography on silica gel (hexane/ethyl acetate/DCM = 9:1:0.5 to 7:3:0.5) to afford **29h** (3.12 g, 5.14 mmol, 91% over two steps). R_f = 0.27 (hexane/ethyl acetate/DCM = 8:2:0.5). $[\alpha]_D^{25}$ = +9.19 (*c* = 2.84, $CHCl_3$). IR (thin film): υ = 2958, 1742, 1455, 1363, 1219 cm^{-1}; ^1H NMR (400 MHz, $CDCl_3$) δ 7.58 (s, 1H), 7.39 – 7.01 (m, 12H), 5.32 (t, *J* = 8.4 Hz, 1H, H-3), 4.91 (d, *J* = 11.0 Hz, 1H, C*H*HPh), 4.68 (d, *J* = 9.8 Hz, 1H, H-1), 4.62 – 4.48 (m, 3H, C*H*HPh, CH_2Ph), 4.33 (d, *J* = 12.1 Hz, 1H, H-6), 4.22 (dd, *J* = 12.0, 3.5 Hz, 1H, H-6), 3.64 – 3.49 (m, 3H, H-2, H-4, H-5), 2.38 (s, 3H, Me), 2.04 (s, 3H, Ac), 1.88 (s, 3H, Ac), 1.29 (s, 9H, *t*-Bu). ^{13}C NMR (100 MHz, $CDCl_3$) δ 170.82 (Ac), 169.96 (Ac), 149.75, 137.77, 137.29, 136.53, 132.74, 130.11, 129.15, 128.71, 128.57, 128.32, 128.27, 128.19, 128.02, 125.01 (Ar), 88.29 (C-1), 78.91 (C-2), 77.30 (C-3), 76.79 (C-4), 76.07 (C-5), 75.03 (Bn), 74.64 (Bn), 63.45 (C-6), 34.62 (Cq, *t*-Bu), 31.47 (Me, *t*-Bu) 21.15 (Ac), 21.08 (Ac), 20.47 Me). MS ESI-HRMS m/z $[M+Na]^+$ calcd for $C_{35}H_{42}O_7SNa$ 629.2549, found 629.2522.

(2-Methyl-5-tert-butylphenyl) 6-di-*O*-Acetyl-2-*O*-benzoyl-4-*O*-benzyl-1-thio-β-D-glucopyranoside S14

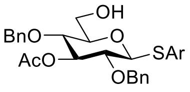

S14

Through the solution of compound **29h** (4.15 g, 6.84 mmol) in MeOH (34 mL, 0.2 M) was bubbled ammonia gas (NH_3) at 0 °C while the starting material was consumed. Excess NH_3 was expelled by bubbling Ar through the solution and then the mixture was evaporated *in vacuo*. The crude product was purified by column chromatography on silica gel (hexane/ethyl acetate/DCM = 8:2:0.5 to 7:3:0.5) to afford **S14** (3.35 g, 5.93 mmol, 87%). R_f = 0.32 (hexane/ethyl acetate/DCM = 7:3:0.5). $[\alpha]_D^{25}$ = +15.78 (C= 2.85, $CHCl_3$). IR (thin film): υ = 3489, 2960, 1748, 1455, 1362, 1231, 1072, 1030 cm^{-1}; ^{1}H NMR (600 MHz, $CDCl_3$) δ 7.55 (d, *J* = 2.0 Hz, 1H), 7.36 – 7.25 (m, 11H), 7.22 (dd, *J* = 7.9, 1.6 Hz, 1H), 7.14 (d, *J* = 8.0 Hz, 1H), 5.32 (t, *J* = 9.3 Hz, 1H, H-3), 4.91 (d, *J* = 11.0 Hz, 1H, C*H*HPh), 4.73 (d, *J* = 9.8 Hz, 1H, H-1), 4.63 – 4.58 (m, 3H, C*H*HPh, CH_2Ph), 3.88 (d, *J* = 12.1 Hz, 1H, H-6), 3.73 (d, *J* = 12.2 Hz, 1H, H-6), 3.65 (t, *J* = 9.6 Hz, 1H, H-4), 3.51 (t, *J* = 9.5 Hz, 1H, H-2), 3.44 (d, *J* = 9.7 Hz, 1H, H-5), 2.39 (s, 3H, Me), 1.87 (s, 3H, Ac), 1.29 (s, 9H, *t*-Bu). ^{13}C NMR (150 MHz, $CDCl_3$) δ 170.01 (Ac), 149.84, 137.81, 137.72, 136.30, 132.64, 130.20, 128.89, 128.66, 128.55, 128.29, 128.17, 128.15, 128.00, 125.04 (Ar), 87.84 (C-1), 79.30 (C-2), 79.16 (C-5), 77.26 (C-3), 75.79 (C-4), 75.07 (Bn), 74.67 (Bn), 62.00 (C-6), 34.62 (Cq, *t*-Bu), 31.45 (Me, *t*-Bu), 21.14 (Ac), 20.42 (Me). MS ESI-HRMS m/z $[M+Na]^+$ calcd for $C_{33}H_{40}O_6SNa$ 587.2443, found 587.2421.

(2-Methyl-5-tert-butylphenyl) 6-*O*-Acetyl-2,4-di-*O*-benzyl-3-*O*-fluorenylmethoxycarbonyl-1-thio-β-D-glucopyranoside 31

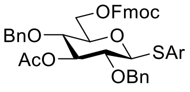

31

To a solution of compound **S12** (2.2 g, 3.90 mmol) were added 9-fluorenylmethyl chloroformate (2.02 g, 7.79 mmol) and pyridine (0.95 mL, 11.69 mmol) at 0 °C and the mixture was stirred for 2 h at room temperature. After completion the mixture was quenched with 1M aqueous HCl and then diluted with DCM. The organic layer was separated and aqueous phase was extracted twice with DCM. The combined organic layer was dried over $MgSO_4$ and the solvent was evaporated *in vacuo*. The crude product was purified by column chromatography on silica gel (hexane/ethyl acetate/DCM = 9:0.5:0.5 to 8.5:1.5:0.5) to afford **31** (2.88 g, 3.66

mmol, 94%). R_f : 0.27 (hexane/ethyl acetate/DCM : 8:2:0.5). $[\alpha]_D^{25}$ = +12.18 (C= 2.85, $CHCl_3$). IR (thin film): υ = 2960, 1747, 1452, 1255, 1226, 1090 cm^{-1}; ^1H NMR (600 MHz, $CDCl_3$) δ 7.75 (t, *J* = 7.2 Hz, 2H), 7.63 – 7.58 (m, 3H), 7.39 (dd, *J* = 15.9, 7.9 Hz, 2H), 7.34 – 7.21 (m, 12H), 7.19 (dd, *J* = 7.9, 2.0 Hz, 1H), 7.10 (d, *J* = 8.0 Hz, 1H), 5.31 (t, *J* = 9.0 Hz, 1H, H-3), 4.90 (d, *J* = 11.1 Hz, 1H, C*H*HPh), 4.68 (d, *J* = 9.8 Hz, 1H, H-1), 4.58 (d, *J* = 11.1 Hz, 1H, C*H*HPh), 4.57 – 4.51 (m, 2H, CH_2Ph), 4.45 – 4.36 (m, 3H, H-6, CH_2 of Fmoc), 4.30 (dd, *J* = 11.6, 4.3 Hz, 1H, H-6), 4.24 (t, *J* = 7.3 Hz, 1H, CH, Fmoc), 3.66 – 3.58 (m, 2H, H-4, H-5), 3.54 (t, *J* = 9.5 Hz, 1H, H-2), 2.37 (s, 3H, Me), 1.85 (s, 3H, Ac), 1.27 (s, 9H, *t*-Bu). ^{13}C NMR (150 MHz, $CDCl_3$) δ 169.92 (Ac), 155.09 (Fmoc), 149.86, 143.54, 143.39, 141.43, 141.42, 137.82, 137.36, 136.58, 132.70, 130.09, 129.43, 128.69, 128.54, 128.30, 128.21, 128.03, 127.98, 127.33, 127.30, 125.32, 125.27, 125.06, 120.21, 120.20 (Ar), 88.36 (C-1), 78.92 (C-2), 77.22 (C-3), 76.71 (C-5), 75.98 (C-4), 75.01 (Bn), 74.67 (Bn), 70.12 (CH_2, Fmoc), 66.68 (C-6), 46.87 (CH, Fmoc), 34.60 (Cq, t-Bu), 31.45 (Me, *t*-Bu), 21.11 (Ac), 20.48 (Me). MS ESI-HRMS m/z $[M+Na]^+$ calcd for $C_{48}H_{50}O_8SNa$ 809.3124, found 809.3120.

2-Methyl-5-tert-butylphenyl 6-*O*-Acetyl-2,4-di-*O*-benzyl-3-*O*-fluorenylmethoxycarbonyl-1-thio-β-D-glucopyranoside 33

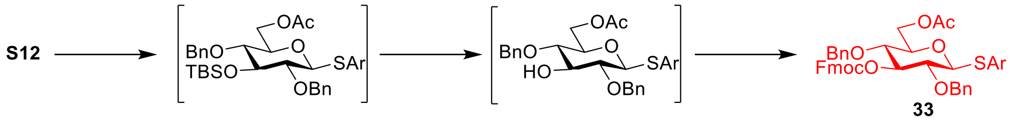

To a solution of compound **S12** (6 g, 9.42 mmol) in anhydrous DCM (47 mL, 0.2 M) were added acetic anhydride (1.78 mL, 18.84 mmol), and triethylamine (3.94 mL, 28.3 mmol), and a catalytic amount of DMAP (0.115 g, 0.942 mmol, 0.1 eq.) at 0 °C and the mixture was stirred for 1 h at 0 °C. After completion the mixture was quenched with saturated aqueous $NaHCO_3$ and then diluted with DCM. The organic layer was dried over $MgSO_4$ and the solvent was evaporated *in vacuo*. To a solution of the resulting crude product in anhydrous acetonitrile (114 mL, 0.08 M) was added boron trifluoride etherate ($BF_3{\cdot}OEt_2$) (1.27 mL, 10.04 mmol) at 0 °C. and the mixture was stirred for 20 min at 0 °C. After completion the mixture was quenched with saturated aqueous $NaHCO_3$ and then diluted with DCM. The organic layer was dried over $MgSO_4$ and the solvent was evaporated *in vacuo*. To a solution of the crude product in anhydrous DCM (47 mL) were added 9-fluorenylmethyl chloroformate (6.09 g, 23.55 mmol) and pyridine (2.24 mL, 28.3 mmol) at 0 °C and the mixture was stirred for 3 h at room temperature. After completion the mixture was quenched with 1M aqueous HCl and then diluted with DCM. The organic layer was separated and aqueous phase was extracted twice

with DCM. The combined organic layer was dried over $MgSO_4$ and the solvent was evaporated *in vacuo*. The crude product was purified by column chromatography on silica gel (hexane/ethyl acetate/DCM = 9:1:0.5 to 8:2:0.5) to afford **33** (6.2 g, 7.88 mmol, 84% over three steps). R_f : 0.26 (hexane/ethyl acetate/DCM = 9:1:0.5). $[\alpha]_D^{25}$ = +27.39 (C= 2.70, $CHCl_3$). IR (thin film): υ = 3034, 2960, 1748, 1489, 1453, 1254, 1092, 1070 cm^{-1}; ^{1}H NMR (600 MHz, $CDCl_3$) δ 7.78 – 7.73 (m, 2H), 7.60 – 7.55 (m, 3H), 7.40 – 7.35 (m, 2H), 7.30 – 7.19 (m, 13H), 7.13 (d, *J* = 8.0 Hz, 1H), 5.14 (t, *J* = 9.1 Hz, 1H, H-3), 4.95 (d, *J* = 10.8 Hz, 1H, C*H*HPh), 4.67 (d, *J* = 9.8 Hz, 1H, H-1), 4.63 (dd, *J* = 11.0, 2.4 Hz, 2H, 2 x C*H*HPh), 4.50 (d, *J* = 11.1 Hz, 1H, C*H*HPh), 4.40 – 4.31 (m, 3H, H-6, CH_2 of Fmoc), 4.21 (dd, *J* = 12.1, 4.9 Hz, 1H, H-6), 4.14 (t, *J* = 7.1 Hz, 1H, CH, Fmoc), 3.65 (t, *J* = 9.5 Hz, 1H, H-4), 3.62 – 3.54 (m, 2H, H-2, H-5), 2.39 (s, 3H, Me), 2.02 (s, 3H, Ac), 1.29 (s, 9H, *t*-Bu). ^{13}C NMR (150 MHz, $CDCl_3$) δ 173.27 (Ac), 157.19 (Fmoc), 152.28, 145.96, 145.84, 143.91, 140.14, 139.71, 139.19, 135.16, 132.61, 131.85, 131.10, 130.92, 130.71, 130.68, 130.53, 130.44, 129.81, 127.66, 127.61, 122.70 (Ar), 90.75 (C-1), 85.20 (C-3), 81.54 (C-2), 79.23 (C-5), 78.17 (C-4), 77.83 (Bn), 77.26 (Bn), 72.72 (CH_2, Fmoc), 65.88 (C-6), 49.37 (CH, Fmoc), 37.11 (Cq, *t*-Bu), 33.97(Me, *t*-Bu), 23.53 (Ac), 22.98 (Me). MS ESI-HRMS m/z $[M+Na]^+$ calcd for $C_{48}H_{50}O_8SNa$ 809.3124, found 809.3089.

Automated Synthesis and Post-Automation Steps

Preparation of Reagent Solutions and Modules (see Supplementary Table 1)

Building Block Solution:

For the glycosylation using twice 5 equivalents, 0.25 mmol of building block was dissolved in 2.0 mL of DCM.

Acidic TMSOTf wash Solution

For the acidic TMSOTf wash 480 μL TMSOTf was dissolved in 20 mL DCM.

Activator Solution

For the thioglycoside monomer *N*-Iodosuccinimide (1.35 g) was dissolved in a 9:1 mixture of anhydrous DCM and dioxane (40.0 mL) and then TfOH (60 μL) was added in ice bath.
For the phosphate monomer 480 μL TMSOTf was dissolved in 20 mL DCM.

Fmoc Deprotection Solution

Solution of 20% triethylamine in DMF (v/v) was prepared.

Preparation of the resin and the synthesizer for automated synthesis: The functionalized resin was loaded into the reaction vessel of the synthesizer and swollen in 2 mL DCM. To start the synthesis sequence, the resin was washed using Module 1. The building blocks were co-evaporated with toluene three times, dissolved in DCM under an argon atmosphere and transferred into the vials that were placed on the corresponding port in the synthesizer. Reagents were dissolved in the corresponding solvents under an Ar atmosphere in bottles that were placed on the corresponding port in the synthesizer.

Module 1 – Acidic TMSOTf Wash: The resin was washed with DMF, THF, DCM (three times each, with 2 mL for 15 s), The resin was swollen in 2 mL DCM, and the temperature of the reaction vessel was adjusted to –20 °C. Once at -20 °C 0.500 mL of the TMSOTf solution in DCM was delivered to the reaction vessel. After one minute, the solution was drained. The resin was swollen in 2 mL DCM and the temperature of the reaction vessel was adjusted to $\mathbf{T_a}$.

Module 2 – Glycosylation: During temperature adjustment, the DCM in the reaction vessel was drained and a solution of thioglycoside building block (5.0 eq. in 1.0 mL DCM) was delivered to the reaction vessel. After the set temperature was reached ($\mathbf{T_a}$), the reaction starts with the addition of 1 mL of NIS/TfOH (5.5 eq. in 1.0 mL DCM), and TfOH (0.2 eq. in 1.0 mL DCM) solution. The glycosylation mixture was activated for an activation time (**t1** = 5 minutes) at **Ta**. The temperature was linearly ramped to the incubation temperature (**Ti**) and finally incubated for an additional incubation time for $\mathbf{t_2}$ at $\mathbf{T_i}$. After the reaction the solution was drained and the resin is washed with DCM (six times with 2 mL for 15 s). This procedure was repeated **twice.**

Module 3 - Fmoc Deprotection: The resin was washed with DMF (six times with 2 mL for 15 s), swollen in 2 mL DMF and the temperature of the reaction vessel iwas adjusted to 25°C. For Fmoc deprotection the DMF is drained and 3.5 mL of a solution of 20% Et_3N in DMF was delivered to the reaction vessel. After 5 min the reaction solution was collected in the fraction collector of the oligosaccharide synthesizer. This procedure was repeated **twice**.

Resin Cleavage: To prepare the photoreactor, the FEP tubing was washed with 20 mL DCM using a flow rate of 5 mL/min. For the cleavage, the resin was slowly injected from the

disposable syringe (20 mL) into the reactor and pushed through the tubing with 18 mL DCM (flow rate: 600 μL/min). The tubing was washed with 20 mL DCM (flow rate: 2 mL/min) to remove any remaining resin. The suspension leaving the reactor was directed into a filter where the resin was filtered off. The system was re-equilibrated by washing the tubes with 20 mL DCM using a flow rate of 5 mL/min. The entire procedure was performed twice. The resulting solution was evaporated and the crude material was analyzed by NMR and HPLC[1].

3.2 Optimization of 1,2-*cis* Galactosidic Bond Formation

Synthesis of trisaccharides 15a – 15g.

The building blocks **11**, **12** and the next corresponding building blocks **13a-13g** were placed on the corresponding building block vial location. Then the automated synthesis started performing reactions with module I, II, and III to afford disaccharides **15a-15g**.

Analytical HPLC: The crude material was analyzed by HPLC (column: Luna 5µ Silica 100A, (260 X 4.60 mm); flow rate: 1 mL/min; eluents: 5% DCM in hexane / 5% DCM in ethyl acetate; gradient: 20% (5 min) 60% (in 40 min) 100% (in 5 min); detection: 280 nm). ***NOTE: The crude material were used to provide analytical HPLC data in supporting information to identify the desire oligosaccharide in the crude mixture following UV cleavage of the photolabile linker conjugated to the resin 10.***

Preparative HPLC: The crude mixture was carefully dissolved in minimum volume of DCM and 0.9 mL of 20% hexane in ethyl acetate. The crude solution was injected for purification using preparative HPLC (column: Luna 5µ Sil (260 X 10 mm); flow rate: 5 mL/min; eluents: 5% DCM in hexane / 5% DCM in ethyl acetate; gradient: 20% (5 min) 60% (in 40 min) 100% (in 5 min); detection: 280 nm) to afford the fully protected target oligosaccharide.

***N*-Benzyloxycarbonyl-5-amino-pentyl 2,3,4,6-tetra-*O*-benzyl-α-D-galactopyranosyl-(1→3)-2-*O*-bezoyl-4,6-di-*O*-benzyl-β-D-galactopyranosyl-(1→4)-2,3-di-*O*-benzoyl-6-*O*-benzyl-β-D-glucopyranoside 15a**

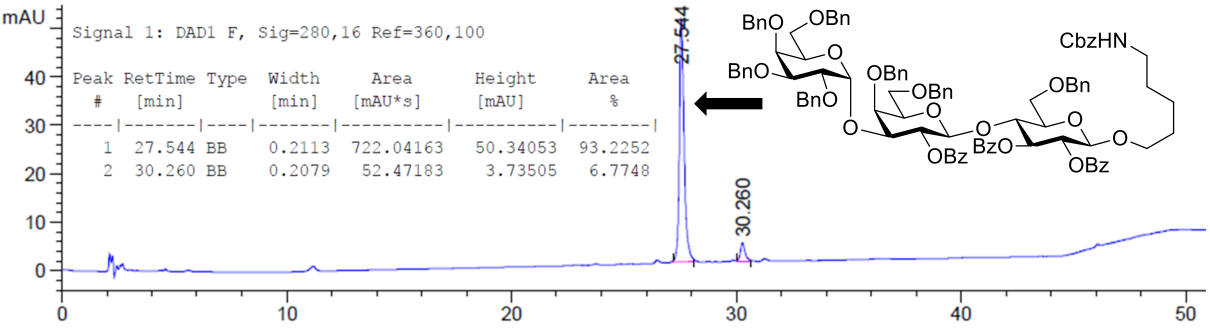

LC-MS chromatogram of **15a.**

^{1}H NMR (600 MHz, $CDCl_3$) δ 7.97 (d, *J* = 7.7 Hz, 2H), 7.87 (d, *J* = 7.6 Hz, 2H), 7.61 (t, *J* = 7.4 Hz, 1H), 7.52 (t, *J* = 7.3 Hz, 1H), 7.46 (t, *J* = 7.6 Hz, 2H), 7.41 – 7.13 (m, 38H), 7.12 – 7.04 (m, 6H), 7.01 – 6.94 (m, 3H), 5.63 – 5.54 (m, 1H, H-2), 5.13 (t, *J* = 8.5 Hz, 1H, H'-2), 5.07 – 5.02 (m, 3H, H''-1, CH_2 of Cbz), 4.87 (dd, *J* = 11.6, 7.2 Hz, 2H, 2 x C*H*HPh), 4.80 (d, *J* = 12.2 Hz, 1H, C*H*HPh), 4.74 (d, *J* = 11.8 Hz, 1H, C*H*HPh), 4.71 – 4.62 (m, 3H, H-1, 2 x C*H*HPh), 4.60 (d, *J* = 12.1 Hz, 1H, C*H*HPh), 4.56 – 4.48 (m, 3H, H-,2 x C*H*HPh), 4.41 (d, *J* = 11.3 Hz, 1H, C*H*HPh), 4.35 – 4.04 (m, 13H), 3.78 – 3.73 (m, 1H, H-6), 3.69 (t, *J* = 8.9 Hz, 1H, H'-3), 3.65 – 3.59 (m, 1H, H-6), 3.56 (t, *J* = 9.1 Hz, 3H, 2 x H-6, $OCH_2(CH_2)_4NHCbz$), 3.44 (br, 1H, H-5), 3.40 (d, *J* = 10.2 Hz, 1H, H-3), 3.29 (d, *J* = 7.4 Hz, 2H, H-5, H-6), 3.19 (dd, *J* = 8.6, 4.6 Hz, 1H, H-6), 2.85 (d, *J* = 6.0 Hz, 2H, CH_2NHCbz), 1.50 – 1.32 (m, 2H, CH_2, pentane), 1.31 – 1.19 (m, 2H, CH_2, pentane), 1.18 – 1.02 (m, 2H, CH_2, pentane). ^{13}C NMR (150 MHz, $CDCl_3$) δ 165.16 (Bz), 165.10 (Bz), 156.37 (Cbz), 139.19, 138.81, 138.76, 138.65, 138.60, 138.35, 138.31, 138.03, 136.87, 133.25, 133.01, 130.22, 130.15, 129.95, 129.89, 128.75, 128.62, 128.56, 128.48, 128.37, 128.35, 128.32, 128.26, 128.22, 128.20, 128.19, 128.16, 127.95, 127.84, 127.80, 127.58, 127.41, 127.39, 127.28, 127.18, 101.31 (C'-1), 101.15 (C''-1), 100.71 (C-1), 79.87, 79.52, 78.90, 76.79, 76.56, 75.13, 75.08, 74.92, 74.29, 74.16, 73.92, 73.62, 73.52, 73.31, 73.22, 73.00, 72.68, 72.24, 71.20, 69.48, 69.23, 68.10, 67.83, 67.46, 66.59, 40.92, 29.45, 28.96, 23.18.; MS ESI$^+$-HRMS *m/z* [M+Na]$^+$ calcd for $C_{101}H_{105}NO_{20}Na$ 1674.7128, found 1674.7037.

_N_-Benzyloxycarbonyl-5-amino-pentyl 6-_O_-acetyl-2,3,4-tri-_O_-benzyl-α-D-galactopyranosyl-(1→3)-2-_O_-bezoyl-4,6-di-_O_-benzyl-β-D-galactopyranosyl-(1→4)-2,3-di-_O_-benzoyl-6-_O_-benzyl-β-D-glucopyranoside 15b

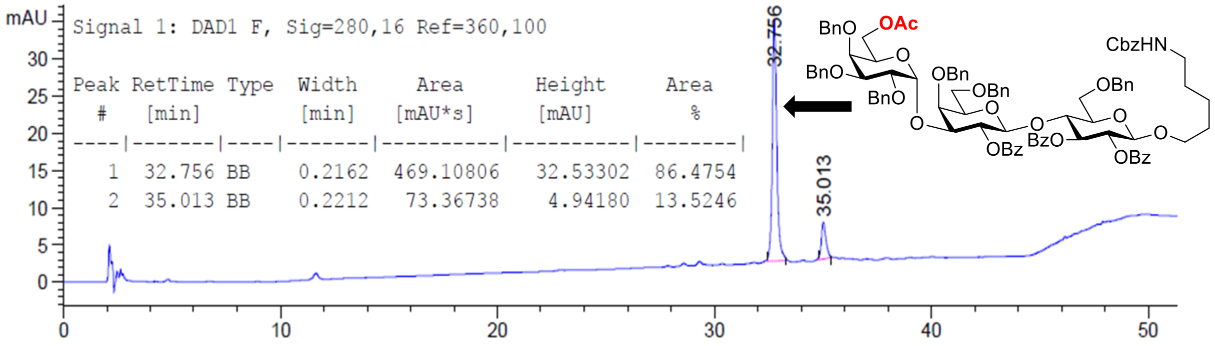

LC-MS chromatogram of **15b**.

^{1}H NMR (400 MHz, $CDCl_3$) δ 7.90 (s, 5H), 7.81 (d, _J_ = 7.6 Hz, 2H), 7.56 – 7.03 (m, 43H), 5.52 (dt, _J_ = 17.6, 9.1 Hz, 2H), 5.29 (s, 1H), 5.04 (s, 2H), 4.95 (d, _J_ = 11.8 Hz, 1H), 4.89 (d, _J_ = 2.6 Hz, 1H, **H''-1**), 4.78 (t, _J_ = 11.4 Hz, 2H), 4.62 – 4.45 (m, 7H, , **H-1** and **H'-1**), 4.39 (d, _J_ = 11.3 Hz, 1H), 4.28 (d, _J_ = 12.3 Hz, 1H), 4.21 (d, _J_ = 11.8 Hz, 1H), 4.01 (ddd, _J_ = 13.1, 12.7, 7.3 Hz, 4H), 3.83 – 3.69 (m, 5H), 3.60 – 3.40 (m, 6H), 3.35 (s, 1H), 3.21 (d, _J_ = 4.4 Hz, 1H), 2.83 (dd, _J_ = 20.7, 11.9 Hz, 4H), 1.77 (s, 3H), 1.40 (dd, _J_ = 15.9, 13.6 Hz, 2H), 1.26 (d, _J_ = 12.7 Hz, 2H), 1.15 (d, _J_ = 6.2 Hz, 2H). ^{13}C NMR (100 MHz, $CDCl_3$) δ 170.01 (Ac), 165.36 (Bz), 165.30 (Bz), 164.82 (Bz), 156.38 (Cbz), 139.38, 138.62, 138.38, 138.32, 138.18, 136.88, 133.27, 133.17, 132.59, 130.51, 129.93, 129.86, 129.83, 129.75, 128.62, 128.52, 128.49, 128.46, 128.39, 128.15, 128.08, 127.90, 127.83, 127.73, 127.63, 127.53, 127.48, 127.05 (Ar), 101.12 (**C-1** and **C'-1**), 98.69 (**C''-1**), 80.20, 79.06, 77.36, 76.43, 75.39, 74.89, 74.68, 74.39, 74.16, 73.74, 73.58, 73.51, 73.21, 73.16, 73.09, 72.22, 69.74, 68.95, 67.85, 67.27, 66.63, 62.16, 40.96, 29.53, 29.03, 23.19, 20.84.; MS ESI^{+}-HRMS _m/z_ $[M+Na]^{+}$ calcd for $C_{96}H_{99}NO_{22}Na$ 1640.6551, found 1640.6558.

***N*-Benzyloxycarbonyl-5-amino-pentyl 6-*O*-benzoyl-2,3,4-tri-*O*-benzyl-α-D-galactopyranosyl-(1→3)-2-*O*-bezoyl-4,6-di-*O*-benzyl-β-D-galactopyranosyl-(1→4)-2,3-di-*O*-benzoyl-6-*O*-benzyl-β-D-glucopyranoside 15c**

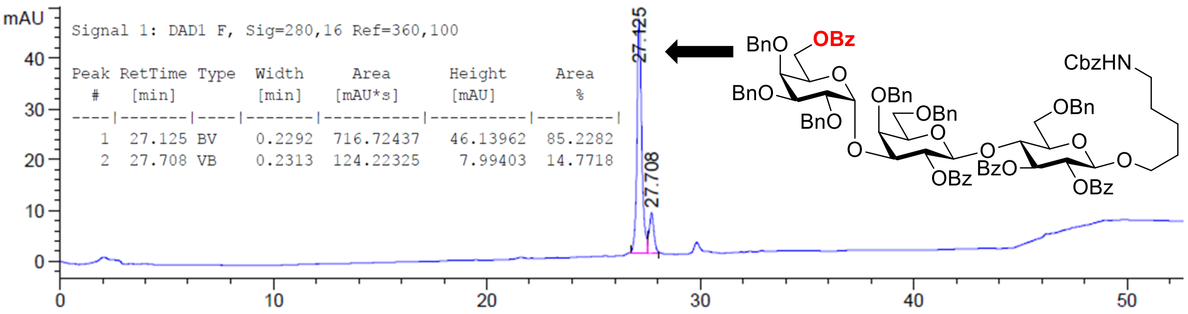

LC-MS chromatogram of **15c**.

^{1}H NMR (400 MHz, $CDCl_3$) δ 7.92 – 7.87 (m, 4H), 7.84 – 7.80 (m, 4H), 7.62 – 7.57 (m, 1H), 7.44 (dd, *J* = 10.5, 5.0 Hz, 3H), 7.39 – 7.05 (m, 43H), 5.60 – 5.52 (m, 2H), 5.30 (dd, *J* = 9.7, 7.9 Hz, 1H), 5.06 (s, 2H), 5.01 (d, *J* = 11.8 Hz, 1H), 4.96 (d, *J* = 3.4 Hz, 1H, **H''-1**), 4.79 (t, *J* = 11.5 Hz, 2H), 4.63 (dd, *J* = 20.8, 11.7 Hz, 2H), 4.54 – 4.47 (m, 4H, **H-1**, **H'-1,** 2 x x C*H*HPh), 4.41 (dd, *J* = 17.5, 11.8 Hz, 2H), 4.23 (d, *J* = 12.1 Hz, 2H), 4.09 – 3.95 (m, 5H), 3.87 (dd, *J* = 12.8, 5.5 Hz, 1H), 3.84 – 3.74 (m, 4H), 3.66 (dd, *J* = 10.2, 2.5 Hz, 1H), 3.57 – 3.42 (m, 4H), 3.37 (dd, *J* = 15.8, 6.6 Hz, 1H), 3.19 (t, *J* = 6.8 Hz, 1H), 2.93 – 2.86 (m, 2H), 2.85 (d, *J* = 7.3 Hz, 2H), 1.54 – 1.37 (m, 2H), 1.28 (d, *J* = 8.6 Hz, 2H), 1.22 – 1.08 (m, 2H). ^{13}C NMR (100 MHz, $CDCl_3$) δ 165.55 (Bz), 165.37 (Bz), 165.24 (Bz), 164.85 (Bz), 156.38 (Cbz), 139.40, 138.69, 138.36, 138.19, 138.16, 136.88, 133.30, 133.23, 133.16, 132.62, 130.48, 130.02, 129.92, 129.85, 129.75, 129.69, 128.62, 128.56, 128.50, 128.45, 128.39, 128.35, 128.20, 128.17, 128.07, 127.84, 127.74, 127.63, 127.60, 127.55, 127.03 (Ar), 101.35 (**C-1**), 101.06 (**C-1**), 98.18 (**C''-1**), 79.65, 79.09, 77.36, 76.32, 75.79, 74.83, 74.70, 74.51, 74.40, 73.79, 73.39, 73.27, 73.21, 73.07, 72.25, 72.01, 69.76, 68.98, 67.99, 67.21, 66.63, 62.38, 40.96, 29.52, 29.03, 23.19.; MS ESI^{+}-HRMS *m/z* $[M+Na]^{+}$ calcd for $C_{101}H_{101}NO_{22}Na$ 1702.6707, found 1702.6699.

***N*-Benzyloxycarbonyl-5-amino-pentyl 4-*O*-acetyl-2,3,6-tri-*O*-benzyl-α-D-galactopyranosyl-(1→3)-2-*O*-bezoyl-4,6-di-*O*-benzyl-β-D-galactopyranosyl-(1→4)-2,3-di-*O*-benzoyl-6-*O*-benzyl-β-D-glucopyranoside 15d**

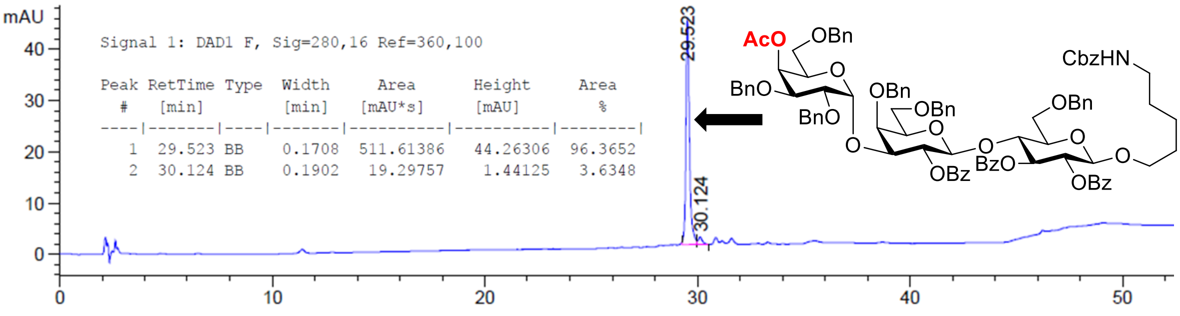

Peak #	RetTime [min]	Type	Width [min]	Area [mAU*s]	Height [mAU]	Area %
1	29.523	BB	0.1708	511.61386	44.26306	96.3652
2	30.124	BB	0.1902	19.29757	1.44125	3.6348

LC-MS chromatogram of **15d**.

^{1}H NMR (400 MHz, $CDCl_3$) δ 7.97 (d, *J* = 7.2 Hz, 2H), 7.93 – 7.89 (m, 2H), 7.83 (dd, *J* = 8.2, 1.1 Hz, 2H), 7.53 (t, *J* = 7.4 Hz, 1H), 7.46 (t, *J* = 7.4 Hz, 1H), 7.40 – 7.09 (m, 40H), 7.05 (dd, *J* = 7.2, 1.9 Hz, 2H), 5.59 (t, *J* = 9.4 Hz, 1H, H-3), 5.53 (dd, *J* = 10.0, 8.0 Hz, 1H, H'-2)), 5.31 (dd, *J* = 9.6, 8.0 Hz, 1H, H-2), 5.18 (s, 1H, H''-4), 5.06 (s, 2H, Cbz), 4.92 (d, *J* = 2.0 Hz, 1H, **H''-1**), 4.87 (d, *J* = 11.8 Hz, 1H), 4.76 (d, *J* = 11.6 Hz, 1H), 4.58 – 4.43 (m, 6H, **H-1**, **H'-1**), 4.34 – 4.15 (m, 5H), 4.10 – 3.95 (m, 4H), 3.86 – 3.76 (m, 2H), 3.74 – 3.63 (m, 3H), 3.62 – 3.47 (m, 3H), 3.38 (dd, *J* = 15.5, 6.6 Hz, 1H), 3.20 (t, *J* = 6.8 Hz, 1H), 3.08 – 2.97 (m, 2H), 2.90 (s, 2H), 2.85 (d, *J* = 6.6 Hz, 2H), 1.93 (s, 3H), 1.54 – 1.36 (m, 2H), 1.33 – 1.22 (m, 2H), 1.22 – 1.09 (m, 2H).^{13}C NMR (100 MHz, $CDCl_3$) δ 170.09 (Ac), 165.37 (Bz), 165.26 (Bz), 164.80 (Bz), 156.39, 139.23, 138.46, 138.42, 138.21, 138.13, 138.06, 136.87, 133.28, 133.17, 132.60, 130.47, 130.06, 129.95, 129.92, 129.85, 129.74, 128.62, 128.49, 128.39, 128.36, 128.16, 128.07, 128.03, 127.98, 127.82, 127.69, 127.61, 127.11, 101.25 (**C'-1**), 101.11 (**C-1**), 98.69 (**C''-1**), 80.29, 77.36, 76.23, 75.72, 75.61, 74.83, 74.66, 74.27, 73.77, 73.52, 73.36, 73.14, 73.10, 72.27, 71.69, 69.76, 68.26, 68.06, 67.84, 67.24, 66.63, 40.95, 29.51, 29.03, 23.19, 20.97.; MS ESI^{+}-HRMS *m/z* $[M+Na]^{+}$ calcd for $C_{96}H_{99}NO_{22}Na$ 1640.6551, found 1640.6561.

N-Benzyloxycarbonyl-5-amino-pentyl 4-O-benzoyl-2,3,6-tri-O-benzyl-α-D-galactopyranosyl-(1→3)-2-O-bezoyl-4,6-di-O-benzyl-β-D-galactopyranosyl-(1→4)-2,3-di-O-benzoyl-6-O-benzyl-β-D-glucopyranoside 15e

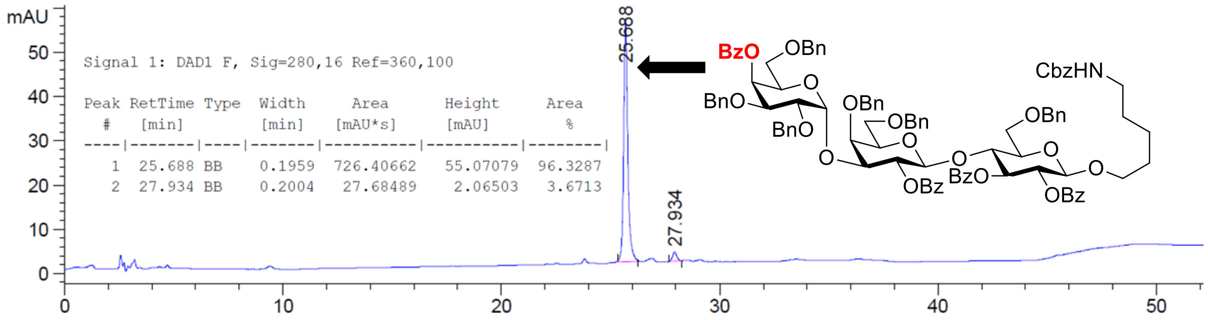

LC-MS chromatogram of **15e**.

^{1}H NMR (400 MHz, $CDCl_3$) δ 8.01 (d, J = 7.6 Hz, 2H), 7.92 (d, J = 7.2 Hz, 4H), 7.83 (d, J = 7.4 Hz, 2H), 7.53 (t, J = 6.8 Hz, 2H), 7.46 (t, J = 7.3 Hz, 1H), 7.42 – 7.06 (m, 44H), 5.63 – 5.52 (m, 2H, H-3, H’-2), 5.36 (s, 1H, H’’-4), 5.32 (d, J = 7.9 Hz, 1H, H-2), 5.06 (s, 2H, Cbz), 4.99 (s, 1H, **H’’-1**), 4.94 (d, J = 11.7 Hz, 1H), 4.73 (d, J = 11.6 Hz, 1H), 4.61 – 4.48 (m, 5H, **H’-1**, **H’-1**, 2 x C*H*HPh), 4.43 (d, J = 12.2 Hz, 1H), 4.30 – 4.21 (m, 4H), 4.16 (d, J = 11.9 Hz, 1H), 4.11 – 3.98 (m, 4H), 3.85 (d, J = 9.1 Hz, 1H), 3.81 (d, J = 10.8 Hz, 3H), 3.75 (d, J = 10.2 Hz, 1H), 3.66 – 3.47 (m, 2H), 3.39 (dd, J = 16.0, 8.8 Hz, 1H), 3.20 (dd, J = 8.0, 5.3 Hz, 1H), 3.17 – 3.03 (m, 2H), 2.95 – 2.80 (m, 4H), 1.52 – 1.38 (m, 2H), 1.33 – 1.22 (m, 2H), 1.22 – 1.05 (m, 3H). ^{13}C NMR (100 MHz, $CDCl_3$) δ 165.63 (Bz), 165.40 (Bz), 165.26 (Bz), 164.81 (Bz), 156.39 (Cbz), 139.25, 138.44, 138.36, 138.24, 138.16, 138.05, 136.89, 133.35, 133.18, 133.01, 132.64, 130.48, 130.28, 130.02, 129.94, 129.90, 129.77, 128.67, 128.63, 128.50, 128.47, 128.42, 128.36, 128.31, 128.27, 128.20, 128.11, 127.94, 127.77, 127.72, 127.68, 127.49, 127.15 (Ar), 101.37 (**C’-1**), 101.16 (**C-1**), 98.16 (**C’’-1**), 79.55, 77.36, 76.50, 75.88, 75.16, 74.91, 74.67, 74.22, 73.88, 73.54, 73.35, 73.14, 72.99, 72.31, 72.15, 71.61, 69.79, 68.58, 68.47, 68.18, 67.26, 66.64, 40.97, 29.55, 29.06, 23.21.; MS ESI$^+$-HRMS m/z $[M+Na]^+$ calcd for $C_{101}H_{101}NO_{22}Na$ 1702.6707, found 1702.6705.

_N_-Benzyloxycarbonyl-5-amino-pentyl 3,4-di-_O_-acetyl-2,6-di-_O_-benzyl-α-D-galactopyranosyl-(1→3)-2-_O_-bezoyl-4,6-di-_O_-benzyl-β-D-galactopyranosyl-(1→4)-2,3-di-_O_-benzoyl-6-_O_-benzyl-β-D-glucopyranoside 15f

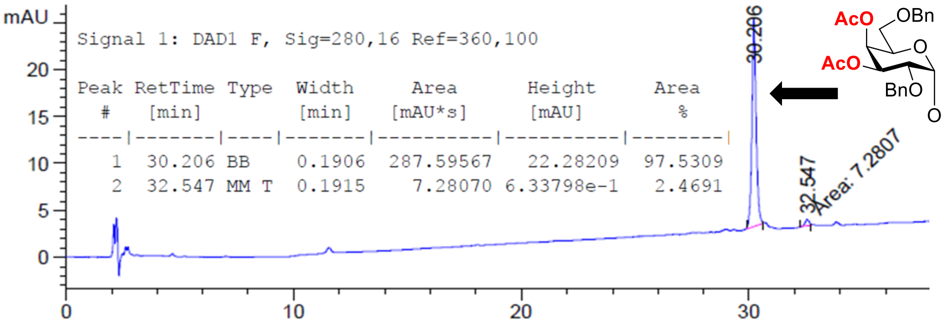

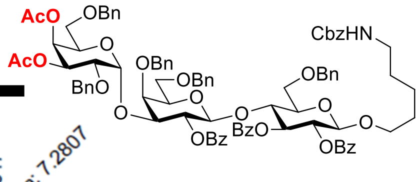

LC-MS chromatogram of **15f**.

^{1}H NMR (400 MHz, $CDCl_3$) δ 8.05 (d, _J_ = 7.4 Hz, 2H), 7.99 (d, _J_ = 7.3 Hz, 2H), 7.94 (d, _J_ = 7.2 Hz, 2H), 7.56 (dt, _J_ = 20.3, 7.3 Hz, 2H), 7.50 – 7.20 (m, 37H), 5.68 (t, _J_ = 9.3 Hz, 1H, H-3), 5.62 – 5.55 (m, 1H, H'-2), 5.42 – 5.36 (m, 1H, H-2), 5.28 (dd, _J_ = 10.5, 3.2 Hz, 1H, H''-3), 5.22 (d, _J_ = 2.0 Hz, 1H, H''-4), 5.14 (s, 2H, Cbz), 5.06 (d, _J_ = 11.8 Hz, 1H), 4.99 (d, _J_ = 3.3 Hz, 1H, **H''-1**), 4.71 (d, _J_ = 11.9 Hz, 1H), 4.64 – 4.51 (m, 5H, **H-1**, **H'-1**, 3 x C_H_HPh), 4.42 (d, _J_ = 11.8 Hz, 1H), 4.36 (d, _J_ = 12.4 Hz, 2H), 4.21 (d, _J_ = 12.0 Hz, 1H), 4.18 – 4.04 (m, 4H), 3.92 (d, _J_ = 7.9 Hz, 1H), 3.91 – 3.82 (m, 2H), 3.71 – 3.53 (m, 4H), 3.46 (dd, _J_ = 15.0, 6.7 Hz, 1H), 3.28 (t, _J_ = 6.6 Hz, 1H), 3.06 (d, _J_ = 6.4 Hz, 2H), 3.03 – 2.92 (m, 4H), 1.97 (s, 3H), 1.97 (s, 3H), 1.62 – 1.43 (m, 2H), 1.42 – 1.31 (m, 2H), 1.30 – 1.18 (m, 2H). ^{13}C NMR (150 MHz, $CDCl_3$) δ 169.92 (Ac), 169.51 (Ac), 165.39 (Bz), 165.23 (Bz), 164.77 (Bz), 156.38 (Cbz), 139.18, 138.48, 138.19, 138.09, 138.04, 136.88, 133.14, 132.63, 130.49, 130.11, 129.99, 129.95, 129.87, 129.75, 128.63, 128.56, 128.48, 128.46, 128.19, 128.12, 127.96, 127.91, 127.89, 127.83, 127.76, 127.71, 127.67, 127.13 (Ar), 101.24 (**C'-1**), 101.12 (**C-1**), 99.00 (**C''-1**), 81.39, 77.36, 75.70, 74.85, 74.53, 73.96, 73.76, 73.37, 73.18, 73.13, 72.30, 69.79, 68.99, 68.07, 67.85, 67.30, 66.63, 40.96, 29.52, 29.04, 23.20, 20.88, 20.75.; MS ESI^{+}-HRMS _m/z_ $[M+Na]^{+}$ calcd for $C_{91}H_{95}NO_{23}Na$ 1592.6187, found 1592.6199.

***N*-Benzyloxycarbonyl-5-amino-pentyl 4,6-di-*O*-acetyl-2,3-di-*O*-benzyl-α-D-galactopyranosyl-(1→3)-2-*O*-bezoyl-4,6-di-*O*-benzyl-β-D-galactopyranosyl-(1→4)-2,3-di-*O*-benzoyl-6-*O*-benzyl-β-D-glucopyranoside 15g**

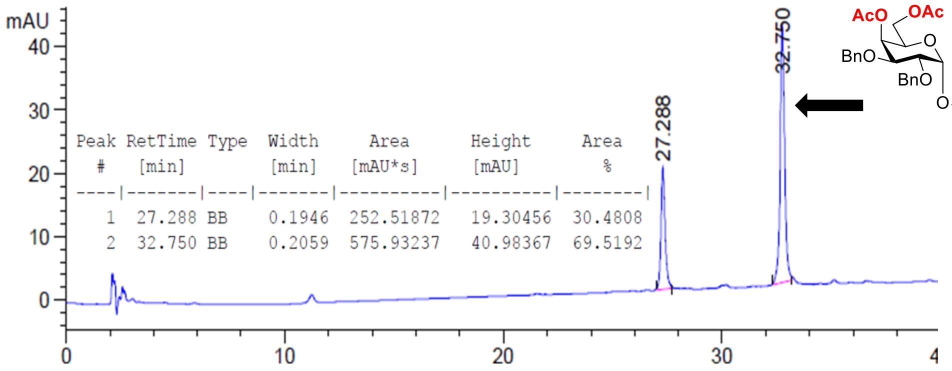

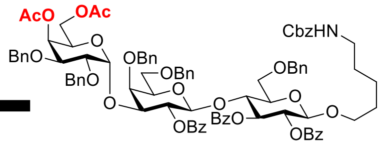

LC-MS chromatogram of **15g**.

^{1}H NMR (400 MHz, $CDCl_3$) δ 7.95 (d, *J* = 7.4 Hz, 2H), 7.91 (d, *J* = 7.4 Hz, 2H), 7.84 (d, *J* = 7.5 Hz, 2H), 7.56 (t, *J* = 7.2 Hz, 1H), 7.48 – 7.11 (m, 35H), 7.05 (d, *J* = 5.5 Hz, 3H), 5.58 (t, *J* = 8.7 Hz, 1H, H-3), 5.55 – 5.46 (m, 1H, H'-2), 5.30 (d, *J* = 5.1 Hz, 1H, H''-4), 5.11 (d, *J* = 12.7 Hz, 1H, H-2), 5.06 (s, 2H, Cbz), 4.90 (d, *J* = 2.6 Hz, 1H, **H''-1**), 4.86 (d, *J* = 11.9 Hz, 1H), 4.77 (d, *J* = 11.5 Hz, 1H), 4.59 – 4.44 (m, 6H, **H-1**, **H'-1**, 4 x C*H*HPh), 4.29 (dd, *J* = 16.6, 11.8 Hz, 2H), 4.20 (d, *J* = 11.9 Hz, 1H), 4.06 (dd, *J* = 16.6, 11.8 Hz, 3H), 3.96 – 3.87 (m, 1H), 3.84 – 3.66 (m, 5H), 3.61 (s, 2H), 3.54 – 3.34 (m, 4H), 3.26 – 3.19 (m, 1H), 2.94 – 2.79 (m, 4H), 2.00 (s, 3H), 1.91 (s, 3H), 1.53 – 1.38 (m, 2H), 1.34 – 1.22 (m, 2H), 1.22 – 1.04 (m, 2H). ^{13}C NMR (150 MHz, $CDCl_3$) δ 170.26 (Ac), 170.19 (Ac), 165.37 (Bz), 165.29 (Bz), 164.81 (Bz), 156.38 (Cbz), 139.25, 138.40, 138.36, 138.12, 138.00, 136.85, 133.44, 133.20, 132.62, 130.48, 129.94, 129.89, 129.82, 129.70, 128.70, 128.63, 128.54, 128.47, 128.44, 128.40, 128.23, 128.18, 128.16, 128.09, 128.07, 127.99, 127.89, 127.88, 127.76, 127.69, 127.49, 127.11 (Ar), 101.17 (**C'-1**), 101.05 (**C-1**), 98.79 (**C''-1**), 80.59, 75.90, 75.54, 75.45, 74.74, 74.40, 73.66, 73.51, 73.22, 73.05, 72.20, 71.85, 69.80, 67.86, 67.17, 67.15, 66.63, 61.13, 40.95, 29.53, 29.03, 23.20, 20.93, 20.83.; MS ESI$^+$-HRMS *m/z* $[M+Na]^+$ calcd for $C_{91}H_{95}NO_{23}Na$ 1592.6187, found 1592.6179.

***N*-Benzyloxycarbonyl-5-amino-pentyl 2-*O*-benzoyl-4,6-di-*O*-benzyl-β-D-galactopyranosyl-(1→4)-2,3-di-*O*-benzoyl-6-*O*-benzyl-β-D-glucopyranoside 15g-byproduct**

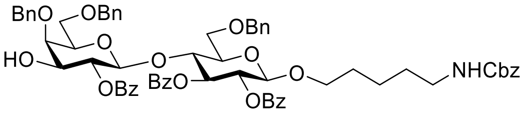

Deletion sequence of 15g: 15g-byproduct

^{1}H NMR (400 MHz, $CDCl_3$) δ 7.97 – 7.89 (m, 6H), 7.58 – 7.53 (m, 1H), 7.49 – 7.38 (m, 4H), 7.37 – 7.27 (m, 18H), 7.23 (dt, J = 8.3, 5.3 Hz, 6H), 5.59 (t, J = 9.4 Hz, 1H, H-3), 5.35 (dd, J = 9.8, 8.0 Hz, 1H, H-2), 5.11 (dd, J = 10.0, 7.9 Hz, 1H, H'-2), 5.06 (s, 2H, Cbz), 4.59 (d, J = 12.2 Hz, 1H, C*H*HPh), 4.57 – 4.49 (m, 5H, **H-1**, **H'-1**, CH_2Ph, NH), 4.36 (d, J = 12.2 Hz, 1H, C*H*HPh), 4.17 – 4.08 (m, 3H, H-4, 2 x C*H*HPh), 3.86 – 3.80 (m, 1H, OC*H*H, linker), 3.75 (d, J = 3.4 Hz, 1H, H'-4), 3.71 (dd, J = 10.9, 3.9 Hz, 1H, H-6), 3.61 (dd, J = 10.9, 1.6 Hz, 1H, H-6), 3.56 – 3.49 (m, 2H, H-5, H'-3), 3.40 (dd, J = 15.5, 6.8 Hz, 1H, OC*H*H, linker), 3.31 (dd, J = 9.3, 5.2 Hz, 1H, H'-5), 2.96 (dd, J = 9.0, 4.9 Hz, 1H, H'-6), 2.91 (td, J = 12.7, 6.3 Hz, 2H, CH_2NHCbz), 2.85 (t, J = 9.2 Hz, 1H, H'-6), 2.24 (d, J = 10.4 Hz, 1H, OH), 1.48 (ddd, J = 20.5, 12.9, 6.5 Hz, 2H), 1.34 – 1.24 (m, 2H), 1.23 – 1.10 (m, 2H). ^{13}C NMR (100 MHz, $CDCl_3$) δ 166.23 (Bz), 165.34 (Bz), 165.31 (Bz), 156.34 (Cbz), 138.22, 138.16, 137.73, 136.76, 133.32, 133.25, 132.77, 130.45, 129.98, 129.91, 129.84, 129.77, 129.57, 128.61, 128.58, 128.57, 128.51, 128.47, 128.22, 128.18, 127.99, 127.94, 127.87, 127.77, 127.67 (Ar), 101.15 (**C-1**), 100.63 (**C'-1**), 76.02 (C'-4), 75.61 (C-4), 75.14 (CH_2Ph), 74.72 (C'-3), 74.29 (C'-2), 73.63 (C-3), 73.54 (CH_2Ph), 73.21 (CH_2Ph), 72.93 (C-5), 72.74 (C'-5), 72.03 (C-2), 69.86 (OCH_2, linker), 67.82 (C-6), 66.89 (C'-6), 66.60 (Cbz), 40.88 (*C*H_2NHCbz), 29.47, 28.97, 23.15. MS ESI+-HRMS *m/z* $[M+Na]^+$ calcd for $C_{67}H_{69}O_{16}NNa$ 1166.4509, found 1166.1548.

_N_-Benzyloxycarbonyl-5-amino-pentyl 3,4-di-_O_-acetyl-2,6-di-_O_-benzyl-α-D-galactopyranosyl-(1→3)-2-_O_-bezoyl-4,6-di-_O_-benzyl-β-D-galactopyranosyl-(1→4)-3-_O_-benzoyl-6-_O_-benzyl-2-deoxy-2-trichloroacetamino-β-D-glucopyranosyl-(1→3)-2-_O_-bezoyl-4,6-di-_O_-benzyl-β-D-galactopyranosyl-(1→4)-2,3-di-_O_-benzoyl-6-_O_-benzyl-β-D-glucopyranoside 23

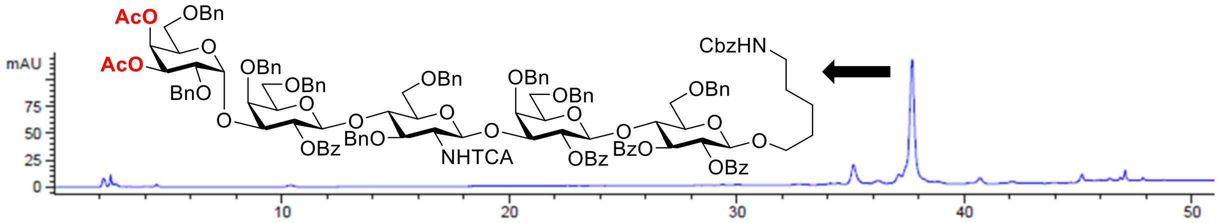

LC-MS chromatogram of **23**.

^{1}H NMR (600 MHz, $CDCl_3$) δ 7.97 (d, *J* = 8.0 Hz, 2H), 7.93 – 7.87 (m, 4H), 7.83 (d, *J* = 8.1 Hz, 2H), 7.52 (dt, *J* = 7.4, 3.7 Hz, 2H), 7.45 (t, *J* = 7.3 Hz, 1H), 7.41 – 7.10 (m, 57H), 7.05 (t, *J* = 7.4 Hz, 2H), 6.53 (d, *J* = 8.1 Hz, 1H, NHTCA), 5.56 (dt, *J* = 15.3, 8.9 Hz, 2H), 5.37 (dd, *J* = 9.9, 8.1 Hz, 1H), 5.32 – 5.25 (m, 2H), 5.24 – 5.21 (m, 1H), 5.07 (d, *J* = 7.9 Hz, 3H), 4.97 (d, *J* = 3.1 Hz, 1H, **H-1**), 4.90 – 4.83 (m, 2H), 4.68 (dd, *J* = 9.7, 5.3 Hz, 2H, **H-1**), 4.56 (dd, *J* = 9.7, 6.6 Hz, 3H, **H-1**), 4.49 (t, *J* = 8.2 Hz, 2H, **H-1**), 4.45 – 4.38 (m, 4H, **H-1**), 4.31 (d, *J* = 11.9 Hz, 1H), 4.27 – 4.20 (m, 4H), 4.15 (d, *J* = 11.8 Hz, 1H), 4.11 – 3.91 (m, 6H), 3.85 – 3.81 (m, 2H), 3.81 – 3.76 (m, 1H), 3.68 (dd, *J* = 17.0, 8.6 Hz, 2H), 3.63 (d, *J* = 10.2 Hz, 1H), 3.58 – 3.45 (m, 4H), 3.39 (dt, *J* = 16.9, 10.0 Hz, 4H), 3.27 – 3.21 (m, 3H), 3.04 – 2.96 (m, 2H), 2.89 (ddd, *J* = 13.5, 10.8, 5.5 Hz, 3H), 2.79 (t, *J* = 8.7 Hz, 1H), 1.92 (s, 3H), 1.91 (s, 3H), 1.52 – 1.36 (m, 2H), 1.34 – 1.23 (m, 2H), 1.16 (dd, *J* = 13.7, 6.2 Hz, 2H). ^{13}C NMR (150 MHz, $CDCl_3$) δ 169.93 (Ac), 169.61 (Ac), 165.33 (Bz), 165.29 (Bz), 164.98 (Bz), 164.60 (Bz), 161.70 (TCA), 156.35 (Cbz), 139.21, 138.94, 138.34, 138.30, 138.20, 138.12, 138.06, 137.99, 137.89, 136.83, 133.38, 133.31, 133.15, 132.46, 130.58, 129.96, 129.93, 129.81, 129.69, 128.68, 128.63, 128.60, 128.55, 128.48, 128.43, 128.42, 128.22, 128.19, 128.14, 128.05, 127.96, 127.94, 127.92, 127.90, 127.88, 127.82, 127.74, 127.70, 127.68, 127.63, 127.36, 127.24, 127.13, 101.04 (**C-1**), 100.97 (**C-1**), 100.83 (**C-1**), 100.32 (**C-1**), 99.24 (**C-1**), 92.12 (CCl_3), 81.49, 79.19, 77.89, 76.24, 75.94, 75.23, 75.16, 75.11, 74.73, 74.53, 74.32, 74.28, 74.15, 73.69, 73.53, 73.40, 73.38, 73.34, 73.08, 72.97, 72.39, 72.14, 69.85, 69.72, 68.90, 68.28, 68.03, 67.86, 67.70, 67.36, 67.13, 66.58, 57.69, 40.91, 29.47, 28.97, 23.15, 20.90, 20.75.; MS ESI^+-HRMS *m/z* $[M+Na]^+$ calcd for $C_{140}H_{141}N_2O_{35}Cl_3Na$ 2537.8273, found 2537.8301.

***N*-Benzyloxycarbonyl-5-amino-pentyl 2,3,4,6-*O*-tetra-benzyl-α-D-galactopyranosyl-(1→4)-2-*O*-bezoyl-3,6-di-*O*-benzyl-β-D-galactopyranosyl-(1→4)-2-*O*-benzoyl-3,6-di-benzyl-β-D-glucopyranoside 24**

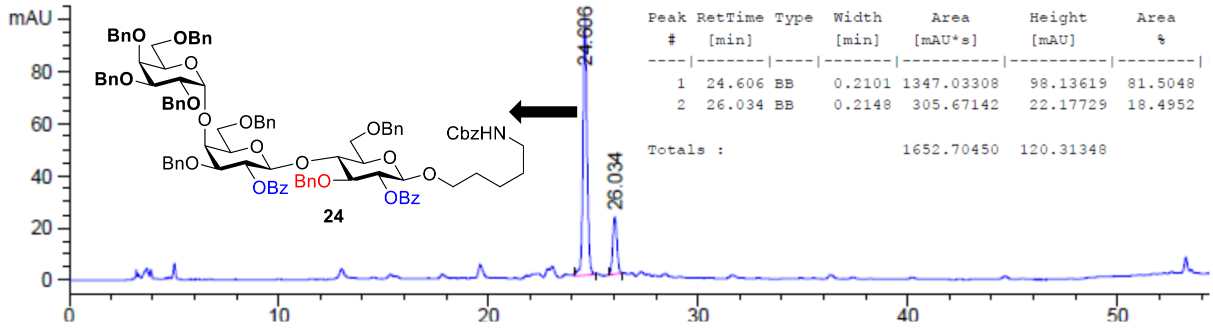

LC-MS chromatogram of **24**.

^{1}H NMR (600 MHz, $CDCl_3$) δ 7.97 (d, *J* = 7.7 Hz, 2H), 7.87 (d, *J* = 7.6 Hz, 2H), 7.61 (t, *J* = 7.4 Hz, 1H), 7.52 (t, *J* = 7.3 Hz, 1H), 7.46 (t, *J* = 7.6 Hz, 2H), 7.41 – 7.13 (m, 38H), 7.12 – 7.04 (m, 6H), 7.01 – 6.94 (m, 3H), 5.63 – 5.54 (m, 1H, H-2), 5.13 (t, *J* = 8.5 Hz, 1H, H'-2), 5.07 – 5.02 (m, 3H, H''-1, CH_2 of Cbz), 4.87 (dd, *J* = 11.6, 7.2 Hz, 2H, 2 x C*H*HPh), 4.80 (d, *J* = 12.2 Hz, 1H, C*H*HPh), 4.74 (d, *J* = 11.8 Hz, 1H, C*H*HPh), 4.71 – 4.62 (m, 3H, H-1, 2 x C*H*HPh), 4.60 (d, *J* = 12.1 Hz, 1H, C*H*HPh), 4.56 – 4.48 (m, 3H, H-,2 x C*H*HPh), 4.41 (d, *J* = 11.3 Hz, 1H, C*H*HPh), 4.35 – 4.04 (m, 13H), 3.78 – 3.73 (m, 1H, H-6), 3.69 (t, *J* = 8.9 Hz, 1H, H'-3), 3.65 – 3.59 (m, 1H, H-6), 3.56 (t, *J* = 9.1 Hz, 3H, 2 x H-6, $OCH_2(CH_2)_4NHCbz$), 3.44 (br, 1H, H-5), 3.40 (d, *J* = 10.2 Hz, 1H, H-3), 3.29 (d, *J* = 7.4 Hz, 2H, H-5, H-6), 3.19 (dd, *J* = 8.6, 4.6 Hz, 1H, H-6), 2.85 (d, *J* = 6.0 Hz, 2H, CH_2NHCbz), 1.50 – 1.32 (m, 2H, CH_2, pentane), 1.31 – 1.19 (m, 2H, CH_2, pentane), 1.18 – 1.02 (m, 2H, CH_2, pentane). ^{13}C NMR (150 MHz, $CDCl_3$) δ 165.16 (Bz), 165.10 (Bz), 156.37 (Cbz), 139.19, 138.81, 138.76, 138.65, 138.60, 138.35, 138.31, 138.03, 136.87, 133.25, 133.01, 130.22, 130.15, 129.95, 129.89, 128.75, 128.62, 128.56, 128.48, 128.37, 128.35, 128.32, 128.26, 128.22, 128.20, 128.19, 128.16, 127.95, 127.84, 127.80, 127.58, 127.41, 127.39, 127.28, 127.18, 101.31 (C'-1), 101.15 (C''-1), 100.71 (C-1), 79.87, 79.52, 78.90, 76.79, 76.56, 75.13, 75.08, 74.92, 74.29, 74.16, 73.92, 73.62, 73.52, 73.31, 73.22, 73.00, 72.68, 72.24, 71.20, 69.48, 69.23, 68.10, 67.83, 67.46, 66.59, 40.92, 29.45, 28.96, 23.18.; MS ESI^{+}-HRMS *m/z* $[M+Na]^{+}$ calcd for $C_{101}H_{105}NO_{20}Na$ 1674.7128, found 1674.7037.

***N*-Benzyloxycarbonyl-5-amino-pentyl 4-*O*-acetyl-2,6-di-*O*-benzyl-α-D-galactopyranosyl-(1→4)-2-*O*-bezoyl-3,6-di-*O*-benzyl-β-D-galactopyranosyl-(1→4)-2-*O*-benzoyl-3,6-di-benzyl-β-D-glucopyranoside 25**

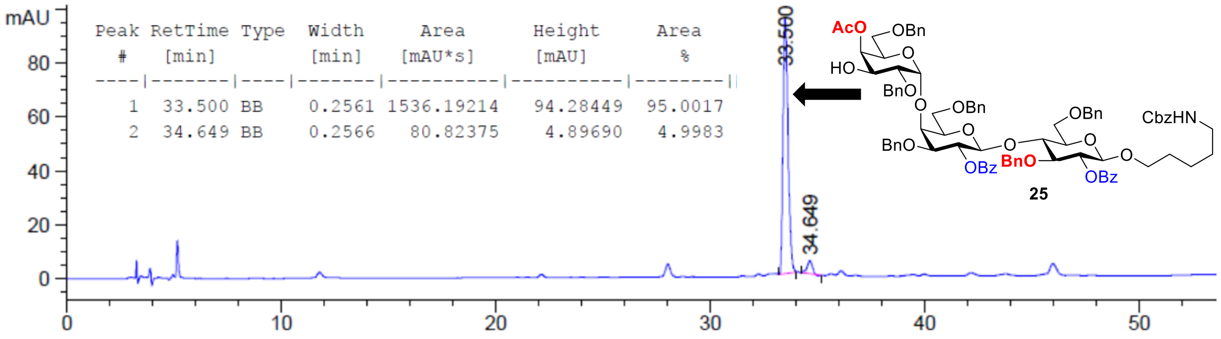

LC-MS chromatogram of **25**.

^{1}H NMR (600 MHz, $CDCl_3$) δ 7.97 – 7.94 (m, 2H), 7.92 (d, J = 7.2 Hz, 2H), 7.62 (t, J = 7.4 Hz, 1H), 7.54 (t, J = 7.4 Hz, 1H), 7.47 (t, J = 7.8 Hz, 2H), 7.42 – 7.29 (m, 10H), 7.28 – 7.14 (m, 20H), 7.13 – 7.08 (m, 4H), 7.07 – 7.00 (m, 3H), 5.51 (q, J = 8.0 Hz, 2H, H-2, H''-4), 5.17 (dd, J = 9.1, 8.2 Hz, 1H, H'-2), 5.13 (d, J = 3.2 Hz, 1H, **H''-1**), 5.07 (s, 2H), 4.92 (d, J = 12.3 Hz, 1H), 4.75 – 4.50 (m, 9H, **H'-1**), 4.36 – 4.25 (m, 7H, **H-1**), 4.17 (d, J = 2.0 Hz, 1H), 4.12 – 4.03 (m, 3H), 3.75 (tdd, J = 14.6, 9.5, 5.0 Hz, 3H), 3.63 (dd, J = 10.9, 3.9 Hz, 1H), 3.59 – 3.48 (m, 2H), 3.43 (ddd, J = 12.9, 9.4, 4.0 Hz, 2H), 3.30 (t, J = 8.6 Hz, 3H), 3.21 (dd, J = 9.6, 4.8 Hz, 1H), 2.88 (d, J = 4.3 Hz, 2H), 1.98 (s, 3H), 1.51 – 1.34 (m, 2H), 1.27 (d, J = 14.7 Hz, 2H), 1.20 – 1.03 (m, 2H). ^{13}C NMR (150 MHz, $CDCl_3$) δ 171.09 (Ac), 165.22 (Bz), 165.01 (Bz), 156.35 (Cbz), 138.90, 138.36, 138.21, 138.20, 138.19, 137.82, 136.86, 133.27, 133.06, 130.20, 130.03, 129.92, 129.85, 128.61, 128.59, 128.55, 128.46, 128.39, 128.37, 128.24, 128.20, 128.16, 128.04, 128.01, 127.92, 127.91, 127.88, 127.82, 127.76, 127.69, 127.57, 127.46, 127.22 (Ar), 101.29 (**C-1** or **C'-1**), 100.61 (**C'-1** or **C-1**), 100.11 (**C''-1**), 80.42, 78.72, 76.74, 74.90, 74.49, 74.07, 73.47, 73.45, 73.36, 73.29, 73.15, 72.13, 71.49, 71.36, 69.50, 68.63, 68.11, 67.86, 67.63, 67.33, 66.58, 40.91, 32.07, 29.84, 29.46, 28.96, 23.18, 22.83, 20.99, 14.26.; MS ESI^{+}-HRMS m/z $[M+Na]^{+}$ calcd for $C_{89}H_{95}NO_{21}Na$ 1536.6289, found 1536.6278.

***N*-Benzyloxycarbonyl-5-amino-pentyl 3,4-di-*O*-acetyl-2-*O*-benzoyl-α-L-fucopyranosyl-2-*O*-bezoyl-4,6-di-*O*-benzyl-β-D-galactopyranosyl-(1→3)-4,6-di-*O*-benzyl-2-deoxy-2-trichloroacetamino-β-D-galactopyranosyl-(1→3)-4-*O*-acetyl-2,6-di-*O*-benzyl-α-D-galactopyranosyl-(1→4)-2-O-bezoyl-3,6-di-*O*-benzyl-β-D-galactopyranosyl-(1→4)-2-*O*-benzoyl-3,6-di-benzyl-β-D-glucopyranoside 26**

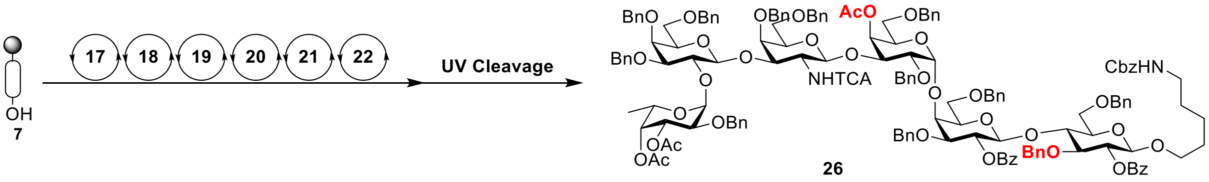

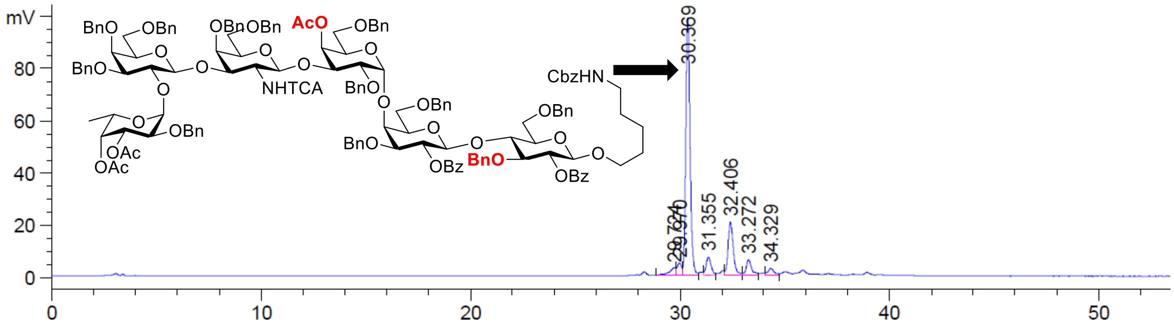

LC-MS chromatogram of **26**.

^{1}H NMR (600 MHz, $CDCl_3$) δ 7.93 (dd, *J* = 7.3, 5.4 Hz, 4H), 7.57 (t, *J* = 7.4 Hz, 1H), 7.49 (t, *J* = 8.1 Hz, 2H), 7.43 – 7.10 (m, 57H), 7.07 – 6.97 (m, 11H), 5.66 (d, *J* = 3.1 Hz, 1H, **H-1**), 5.57 (d, *J* = 2.5 Hz, 1H), 5.49 – 5.43 (m, 1H), 5.41 (dd, *J* = 10.6, 2.8 Hz, 1H), 5.19 (d, *J* = 1.5 Hz, 1H), 5.16 (t, *J* = 8.5 Hz, 1H), 5.10 (d, *J* = 8.0 Hz, 1H, **H-1**), 5.06 (s, 2H), 4.97 – 4.95 (m, 2H, **H-1**), 4.92 – 4.86 (m, 3H, **H-1**), 4.74 – 4.71 (m, 2H), 4.69 – 4.63 (m, 4H), 4.57 – 4.50 (m, 5H, **H-1**), 4.50 – 4.38 (m, 8H), 4.33 – 4.27 (m, 3H, **H-1**), 4.22 (dd, *J* = 17.3, 7.9 Hz, 4H), 4.18 (d, *J* = 2.7 Hz, 1H), 4.15 (d, *J* = 13.2 Hz, 1H), 4.09 (d, *J* = 11.7 Hz, 1H), 4.06 (d, *J* = 7.9 Hz, 1H), 4.05 – 4.01 (m, 2H), 3.98 – 3.93 (m, 2H), 3.92 (t, *J* = 9.0 Hz, 1H), 3.74 (dt, *J* = 11.6, 8.8 Hz, 4H), 3.69 – 3.64 (m, 2H), 3.63 – 3.59 (m, 1H), 3.59 – 3.47 (m, 6H), 3.42 – 3.25 (m, 8H), 2.87 (dd, *J* = 12.4, 6.1 Hz, 2H), 2.05 (s, 3H), 2.03 (s, 3H), 1.79 (s, 3H), 1.49 – 1.32 (m, 2H), 1.30 – 1.21 (m, 2H), 1.18 – 1.02 (m, 2H), 0.83 (d, *J* = 6.4 Hz, 3H). ^{13}C NMR (150 MHz, $CDCl_3$) δ 170.97 (Ac), 170.52 (Ac), 170.28 (Ac), 165.15 (Bz), 164.85 (Bz), 162.68 (NHTCA), 156.36 (Cbz), 139.18, 139.04, 138.93, 138.86, 138.77, 138.50, 138.37, 138.26, 138.03, 137.84, 136.88, 133.11, 133.06, 130.21, 129.98, 129.84, 128.63, 128.62, 128.54, 128.51, 128.46, 128.42, 128.35, 128.32, 128.26, 128.19, 128.15, 128.07, 128.01, 127.99, 127.90, 127.76, 127.70, 127.68, 127.64, 127.59, 127.53, 127.48, 127.36, 127.31, 127.07, 126.78, 102.41 (**C-1**), 101.46 (**C-1**), 101.10 (**C-1**), 100.67 (**C-1**), 98.66 (**C-1**), 96.71 (**C-1**), 92.58 (CCl_3), 83.99, 81.06, 78.56,

77.65, 75.98, 75.54, 75.29, 74.78, 74.71, 74.32, 74.25, 73.92, 73.85, 73.76, 73.45, 73.40, 73.24, 73.17, 73.11, 72.78, 72.53, 72.20, 72.02, 71.77, 71.45, 71.12, 70.33, 69.45, 69.18, 68.46, 68.35, 68.28, 67.90, 66.58, 64.88, 57.48, 40.93, 29.85, 29.47, 29.00, 23.20, 21.25, 20.88, 20.79, 15.61.; MS ESI$^+$-HRMS *m/z* [M+Na]$^+$ calcd for $C_{155}H_{165}N_2O_{37}Cl_3Na$ 2774.0049, found 2773.9809.

3.5 Post-Automation Steps: Deprotection and the Final Purification

Deprotection Conditions: To a solution of the fully protected oligosaccharide in MeOH (5 mL) was added 58 µL of 0.5 M NaOMe solution (0.25 eq. per acetyl or benzoyl group) in MeOH at 40 ° C. The mixture is stirred until LC-MS analysis indicated complete deprotection, then neutralized by 200 mg of Amberite (400 mg per 100 µL of NaOMe solution) . The amberlite was filtered off and the crude filtrate evapoarted andre-dissolved in MeOH, ethyl acetate, and AcOH (v/v/v =5:0.5:0.2) added 5% Pd/C (W/V), purged first with argon and then with hydrogen, left to stir overnight at room temperature under balloon pressure. The reaction mixture was filtered through syringe filter, washed with 20 mL of Water/MeOH, 9:1 and the combined solution was evaporated to provide the crude product.

Analytical HPLC: The crude material was analyzed by HPLC (column: Hypercarb®, (150 X 4.60 mm); flow rate: 0.8 mL/min; eluents: 0.1% FA in Acetonitrile / 0.1% FA in TDW; gradient: 0% (10 min) 30% (in 30 min) 100% (in 5 min); detection: ELSD).
NOTE: The crude material was analysized to provide analytical HPLC data in supporting information to identify the desire oligosaccharide in the crude mixture following deprotection steps from pure protected oligosacchride.

Preparative HPLC: The crude solution is purified by preparative HPLC (column: Hypercarb®, (150 X 10.00 mm); flow rate: 3.6 mL/min; eluents: 0.1% FA in Acetonitrile / 0.1% FA in TDW; gradient: 0% (10 min) 30% (in 30 min) 100% (in 5 min); detection: ELSD) to afford the unprotected oligosaccharide.
Note: HPLC purifications using 0.1% formic acid (FA) sometime result in the fromation of formic acid salt with conjugation-ready oligosaccharide. The detection of formic acid by ^{1}H ***and*** ^{13}C ***NMR does not imply of impurity.***

5-Amino-pentyl α-D-galactopyranosyl-(1→3)-β-D-galactopyranosyl-(1→4)-β-D-glucopyranoside 1

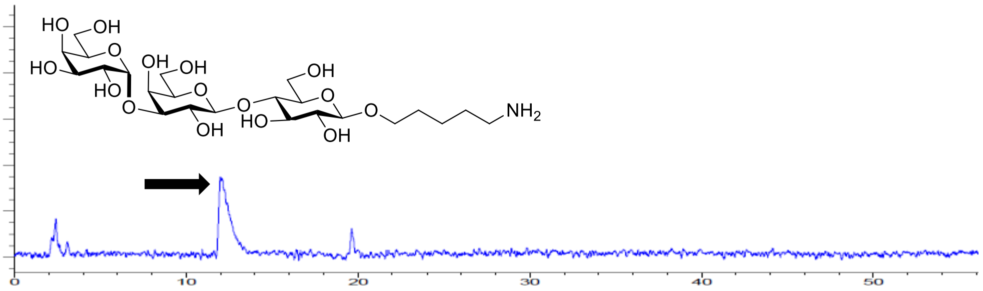

LC-MS chromatogram of **1**.

^{1}H NMR (600 MHz, D_2O) δ 8.48 (s, 1H, HCO_2H), 5.17 (d, J = 4.0 Hz, 1H, H, H''-1), 4.55 (d, J = 8.0 Hz, 1H, H-1), 4.52 (d, J = 8.1 Hz, 1H, H-1), 4.25 – 4.19 (m, 2H), 4.05 (d, J = 3.1 Hz, 1H), 4.02 (dd, J = 12.3, 1.8 Hz, 1H), 3.99 – 3.94 (m, 2H), 3.89 (dd, J = 10.4, 3.9 Hz, 1H), 3.86 – 3.65 (m, 12H), 3.64 – 3.61 (m, 1H), 3.36 – 3.32 (m, 1H), 2.98 (t, J = 7.3 Hz, 2H, $OCH_2(CH_2)_4NH_2$), 1.73 – 1.66 (m, 4H, $OCH_2CH_2CH_2CH_2CH_2NH_2$), 1.52 – 1.44 (m, 2H $OCH_2CH_2CH_2CH_2CH_2NH_2$). ^{13}C NMR (150 MHz, D_2O) δ 173.63 (CO_2H), 105.45 (C-1), 104.62(C-1), 98.03(C''-1), 81.28, 79.81, 77.66, 77.35, 77.13, 75.41, 73.44, 72.75, 72.18, 71.89, 71.73, 70.79, 67.41, 63.59, 63.53, 62.77, 42.09 (CH_2NH_2), 30.80, 29.63, 24.73.; MS ESI^{+}-HRMS *m/z* $[M+Na]^{+}$ calcd for $C_{23}H_{44}NO_{16}Na$ 590.2655, found 590.2644.

5-Amino-pentyl α-D-galactopyranosyl-(1→3)-β-D-galactopyranosyl-(1→3)-2-deoxy-2-acetoamido-β-D-galactopyranosyl-(1→3)-β-D-galactopyranosyl-(1→4)-β-D-glucopyranoside 2

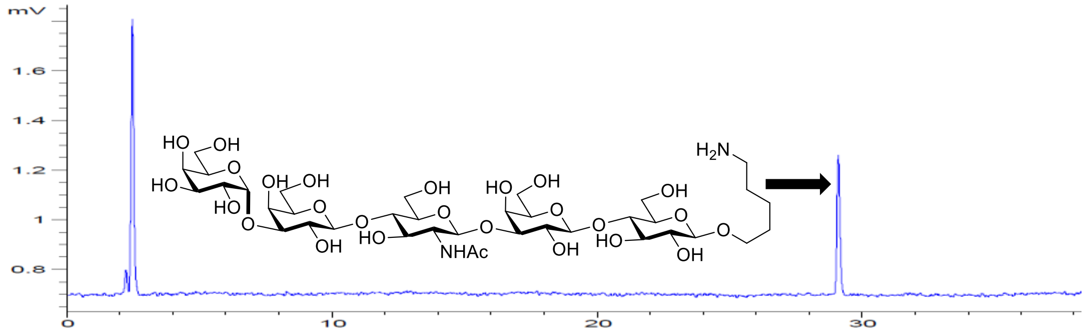

LC-MS chromatogram of **2.**

^{1}H NMR (500 MHz, D_2O) δ 5.13 (d, *J* = 3.8 Hz, 1H, **H''''-1**), 4.69 (d, *J* = 8.3 Hz, 1H, **H-1**), 4.54 (d, *J* = 7.8 Hz, 1H, **H-1**), 4.47 (d, *J* = 8.0 Hz, 1H, **H-1**), 4.42 (d, *J* = 7.9 Hz, 1H, **H-1**), 4.17 (dd, *J* = 8.3, 4.6 Hz, 2H), 4.14 (d, *J* = 2.9 Hz, 1H), 4.00 (d, *J* = 2.8 Hz, 1H), 3.98 – 3.89 (m, 4H), 3.86 – 3.53 (m, 23H), 3.29 (dd, *J* = 11.3, 5.7 Hz, 1H), 3.01 – 2.96 (m, 2H), 2.02 (s, 3H), 1.71 – 1.59 (m, 4H), 1.44 (dt, *J* = 15.2, 7.7 Hz, 2H). ^{13}C NMR (176 MHz, D_2O) δ 177.49 (NHAc), 105.54 (**C-1**), 105.38 (**C-1**), 105.34 (**C-1**), 104.59 (**C-1**), 98.04 (**C''''-1**), 84.67, 81.03, 79.81, 77.65, 77.49, 77.38, 77.14, 77.06, 75.41, 74.85, 73.45, 72.68, 72.55, 72.20, 71.89, 71.73, 70.91, 70.79, 67.41, 63.59, 63.55, 63.53, 62.69, 62.55, 57.78, 41.97, 30.75, 29.05, 25.87, 24.79, 24.68.; MS ESI+-HRMS *m/z* [M+Na]+ calcd for $C_{39}H_{70}N_2O_{26}Na$ 996.4242, found 996.4228.

5-Amino-pentyl α-D-galactopyranosyl-(1→4)-β-D-galactopyranosyl-(1→4)-β-D-glucopyranoside 3

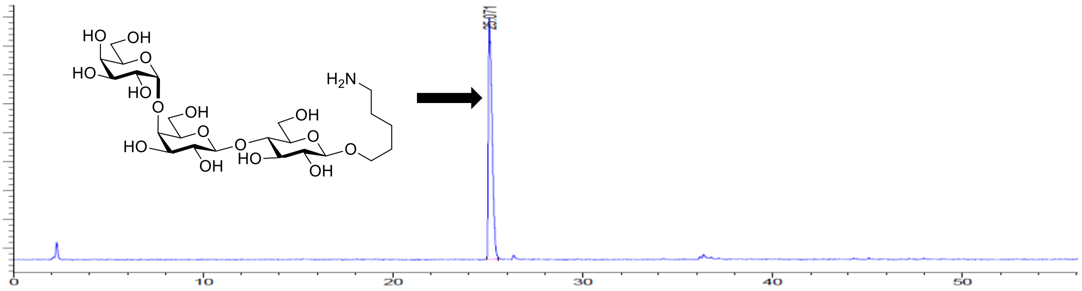

LC-MS chromatogram of **3.**

^{1}H NMR (600 MHz, D_2O) δ 4.96 (d, *J* = 3.9 Hz, 1H, H''-1), 4.52 (d, *J* = 7.8 Hz, 1H, H-1), 4.50 (d, *J* = 8.0 Hz, 1H, H-1), 4.37 (t, *J* = 6.4 Hz, 1H), 4.07 – 3.56 (m, 18H), 3.34 – 3.29 (m, 1H), 3.04 – 3.00 (m, 2H, $OCH_2(CH_2)_4NH_2$), 1.75 – 1.64 (m, 4H, 2H $OCH_2CH_2CH_2CH_2CH_2NH_2$), 1.47 (dt, *J* = 15.3, 7.6 Hz, 2H, $-CH_2CH_2CH_2-$). ^{13}C NMR (150 MHz, D_2O) δ 108.44 (C-1), 107.14 (C-1), 105.48 (C''-1), 83.90, 82.54, 80.62, 79.99, 79.70, 78.09, 77.35, 76.10, 76.01, 75.25, 74.31, 74.12, 73.73, 65.69, 65.56, 65.24, 44.54 (CH_2NH_2), 33.33, 31.58, 28.43, 27.26.; MS ESI+-HRMS *m/z* [M+Na]+ calcd for $C_{23}H_{43}NO_{16}Na$ 612.2474, found 612.2484.

5-Amino-pentyl α-L-fucopyranosyl-(1→2)-β-D-galactopyranosyl-(1→3)-2-depxy-2-amidoaceto-β-D-galactopyranosyl-(1→3)-α-D-galactopyranosyl-(1→4)-β-D-galactopyranosyl-(1→4)-β-D-glucopyranoside 4

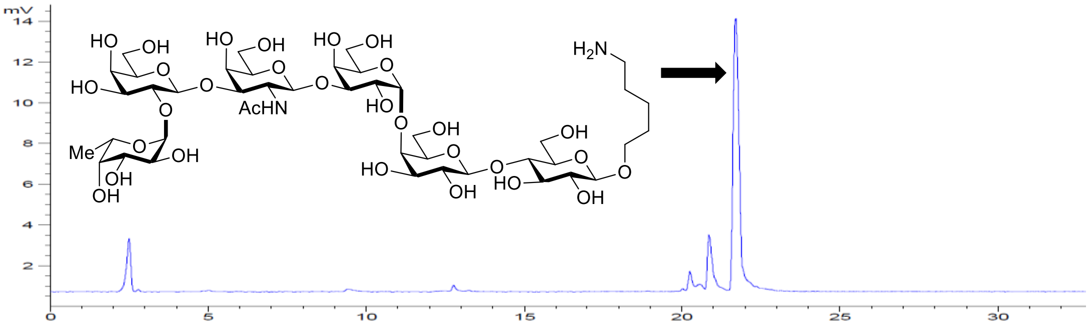

LC-MS chromatogram of **4.**

^{1}H NMR (500 MHz, D_2O) δ 8.46 (s, 1H, formic acid), 5.25 (d, *J* = 4.1 Hz, 1H, **H-1**), 4.82 (d, *J* = 3.9 Hz, 1H, **H-1**), 4.63 (d, *J* = 7.6 Hz, 1H, **H-1**), 4.56 (d, *J* = 7.1 Hz, 1H, **H-1**), 4.53 (d, *J* = 7.6 Hz, 1H, **H-1**), 4.50 (d, *J* = 8.1 Hz, 1H, **H-1**), 4.40 (t, *J* = 6.6 Hz, 1H), 4.25 (d, *J* = 7.1 Hz, 1H), 4.07 – 3.54 (m, 31H), 3.33 (d, *J* = 6.6 Hz, 1H), 3.02 (t, *J* = 7.7 Hz, 2H), 2.06 (s, 3H), 1.76 – 1.64 (m, 4H), 1.50 – 1.41 (m, 2H), 1.23 (d, *J* = 6.6 Hz, 3H). ^{13}C NMR (176 MHz, D_2O) δ 174.29 (NHAc), 170.81 (formic acid), 103.96 (**C-1**), 103.32 (**C-1**), 102.05 (**C-1**), 101.97 (**C-1**), 100.45 (**C-1**), 99.28 (**C-1**), 78.83, 78.26, 77.20, 76.38, 76.11, 75.50, 75.08, 74.81, 74.63, 74.55, 73.59, 72.95, 72.13, 71.86, 70.88, 70.18, 70.09, 69.53, 69.18, 69.12, 68.49, 68.03, 67.83, 66.79, 60.99, 60.96, 60.38, 60.07, 51.65, 39.35, 28.17, 26.40, 22.26, 22.08, 15.32.; MS ESI+-HRMS *m/z* [M+H]+ calcd for $C_{43}H_{78}N_2O_{30}$ 1101.4556, found 1101.4518.

3.6 Optimization of 1,2-*cis*-Glucosidic Formation

Synthesis of Disaccharides 30a – 30j

The building blocks **27, 17, or 28** and the next corresponding building block B (**29a** to **29h**) were placed on the corresponding building block vial location. Then the automated synthesis started performing reactions with module 1, 2, and to afford disaccharides **30a – 30j**.

Analytical HPLC: The crude material was analyzed by HPLC (column: Luna 5µ Silica 100A, (260 X 4.60 mm); flow rate: 1 mL/min; eluents: 5% DCM in hexane / 5% DCM in ethyl acetate; gradient: 20% (5 min) 60% (in 40 min) 100% (in 5 min); detection: 280 nm). ***NOTE: The crude material were used to provide analytical HPLC data in supporting information to identify the desire oligosaccharide in the crude mixture followed by UV cleavage of the photolabile linker conjugated to the resin 10.***

Preparative HPLC: The crude mixture was carefully dissolved in minimum volume of DCM and 0.9 mL of 20% hexane in ethyl acetate. The crude solution was injected for purification using preparative HPLC (column: Luna 5µ Sil (260 X 10 mm); flow rate: 5 mL/min; eluents: 5% DCM in hexane / 5% DCM in ethyl acetate; gradient: 20% (5 min) 60% (in 40 min) 100% (in 5 min); detection: 280 nm) to afford the fully protected target oligosaccharide.

N-Benzyloxycarbonyl-5-amino-pentyl (2,3,4,6-tetra-O-benzyl-α-D-glucopyranosyl)-(1→6)-2,3-di-O-benzoyl-β-D-glucopyranoside 30a

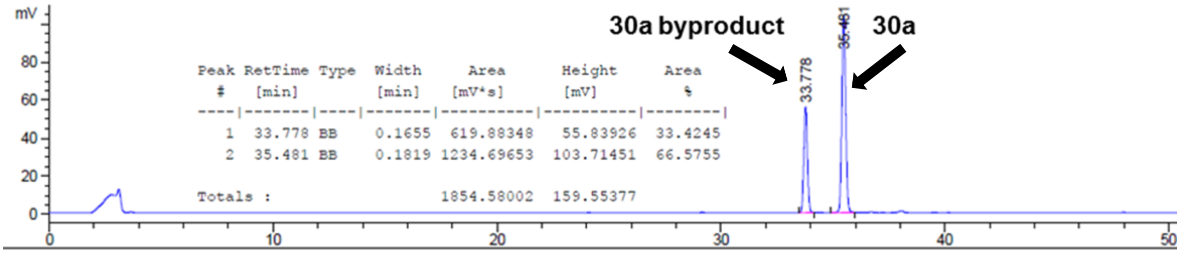

LC-MS chromatogram of **30a**

N-Benzyloxycarbonyl-5-amino-pentyl (2,3,4,6-tetra-O-benzyl-β-D-glucopyranosyl)-(1→6)-2,3-di-O-benzoyl-β-D-glucopyranoside 30a -byproduct

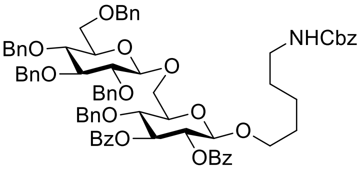

^{1}H NMR (600 MHz, $CDCl_3$) δ 7.93 (d, J = 7.7 Hz, 2H), 7.89 (d, J = 7.8 Hz, 2H), 7.52 – 7.44 (m, 2H), 7.39 – 7.23 (m, 27H), 7.18 – 7.12 (m, 5H), 7.07 (dd, J = 6.4, 2.8 Hz, 2H), 5.72 – 5.68 (m, 1H, H-3), 5.35 – 5.30 (m, 1H, H-2), 5.06 (s, 2H, CH_2, Cbz), 5.01 (d, J = 11.1 Hz, 1H, C*H*HPh), 4.93 (d, J = 10.9 Hz, 1H, C*H*HPh), 4.84 – 4.77 (m, 3H, 3 x C*H*HPh), 4.64 (d, J = 12.2 Hz, 1H, C*H*HPh), 4.58 – 4.51 (m, 4H, H-1, NH, 2 x C*H*HPh), 4.50 – 4.43 (m, 3H, H'-1, CH_2Ph), 4.25 (d, J = 10.0 Hz, 1H, H-6_a), 3.83 – 3.69 (m, 6H, H-4, H-5, H-6_b, H'-6, OCHH$(CH_2)_4$NHCbz), 3.66 – 3.59 (m, 2H, H'-3. H'-4), 3.51 (t, J = 8.4 Hz, 1H, H'-2), 3.43 (d, J = 3.0 Hz, 1H, H'-5), 3.32 (dd, J = 15.2, 6.6 Hz, 1H, OCHH$(CH_2)_4$NHCbz), 2.86 (dd, J = 12.8, 6.4 Hz, 2H, CH_2NHCbz), 1.43 – 1.33 (m, 2H), 1.28 – 1.20 (m, 2H), 1.14 – 1.04 (m, 2H). ^{13}C NMR (150 MHz, $CDCl_3$) δ 165.80 (Bz), 165.38 (Bz), 156.38 (Cbz), 138.69, 138.33, 138.26, 137.40, 136.90, 133.29, 133.25, 129.88, 129.85, 129.69, 129.58, 128.63, 128.56, 128.53, 128.50, 128.49, 128.45, 128.25, 128.17, 128.14, 128.12, 128.10, 128.01, 127.98, 127.94, 127.90, 127.76, 104.20 (C'-1), 101.19 (C-1), 84.94 (C'-3 or -4), 82.29 (C'-2), 77.98(C'-3 or -4), 76.84 (C-4 or -5), 75.84 (CH_2Ph), 75.35 (C-3), 75.26 (C-4 or -5), 75.17 (C'-5), 75.13 (CH_2Ph), 74.95 (CH_2Ph), 74.90 (CH_2Ph), 73.69 (CH_2Ph), 72.38 (C-2), 69.85 (OCH_2, linker), 69.03 (C'-6), 68.63 (C-6), 66.61 (CH_2Ph, Cbz), 40.96 (CH_2NHCbz), 29.47, 28.98, 23.17.; MS ESI+-HRMS m/z [M+Na]+ calcd for $C_{74}H_{77}NO_{15}$ 1242.5185, found 1242.5177.

_N_-Benzyloxycarbonyl-5-amino-pentyl (2,3,4,6-tetra-_O_-benzyl-α-D-glucopyranosyl)-(1→6)-2,3-di-_O_-benzoyl-β-D-glucopyranoside 30a

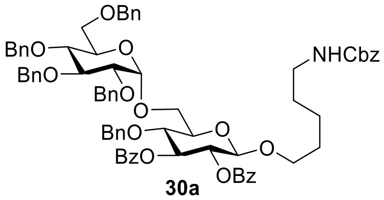

30a

^{1}H NMR (600 MHz, $CDCl_3$) δ 7.93 (d, *J* = 7.9 Hz, 2H), 7.82 (d, *J* = 8.0 Hz, 2H), 7.48 (dd, *J* = 17.0, 7.3 Hz, 4H), 7.40 – 7.22 (m, 25H), 7.17 – 7.03 (m, 7H), 5.67 (t, *J* = 9.6 Hz, 1H, H-3), 5.33 – 5.28 (m, 1H, H-1), 5.16 (d, *J* = 3.3 Hz, 1H, H'-1), 5.06 (s, 2H, CH_2Ph, Cbz), 5.03 (d, *J* = 10.9 Hz, 1H, C*H*HPh), 4.83 (dd, *J* = 11.0, 5.7 Hz, 3H, 3 x C*H*HPh), 4.77 (d, *J* = 11.8 Hz, 1H, C*H*HPh), 4.64 – 4.53 (m, 5H, H-1, NH, 3 x C*H*HPh), 4.46 (dd, *J* = 11.4, 7.5 Hz, 2H, 2 x C*H*HPh), 4.02 (t, *J* = 9.3 Hz, 1H, H'-3), 3.96 – 3.83 (m, 5H, H-4, H'-5, H'-6, OCHH of linker), 3.73 – 3.63 (m, 5H, H-5, H-6, H'-2, H'-4), 3.43 (dd, *J* = 14.6, 7.0 Hz, 1H OCHH, linker), 2.88 (dd, *J* = 12.8, 6.4 Hz, 2H, CH_2NHCbz), 1.49 – 1.35 (m, 2H), 1.31 – 1.22 (m, 2H), 1.20 – 1.09 (m, 2H). ^{13}C NMR (150 MHz, $CDCl_3$) δ 165.82 (Bz), 165.29 (Bz), 156.39 (Cbz), 138.97, 138.52, 138.36, 138.10, 137.47, 136.89, 133.21, 133.17, 129.92, 129.84, 129.72, 129.58, 128.71, 128.62, 128.52, 128.51, 128.47, 128.43, 128.38, 128.36, 128.25, 128.17, 128.09, 128.06, 127.94, 127.91, 127.83, 127.74, 127.70, 101.33 (C-1), 97.13 (C'-1), 82.00, 80.22, 77.77, 76.10, 75.74, 75.47, 75.17, 75.05, 74.74, 73.55, 72.94, 72.47, 70.36, 69.83, 68.66, 66.61, 64.98, 40.97, 29.52, 29.07, 23.27.; MS ESI+-HRMS m/z [M+Na]+ calcd for $C_{74}H_{77}NO_{15}$ 1242.5185, found 1242.5177.

_N_-Benzyloxycarbonyl-5-amino-pentyl (6-_O_-acetyl-2,3,4-tri-_O_-benzyl-α-D-glucopyranosyl)-(1→6)-2,3-di-_O_-benzoyl-β-D-glucopyranoside 30b

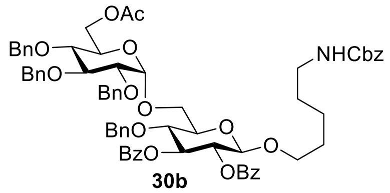

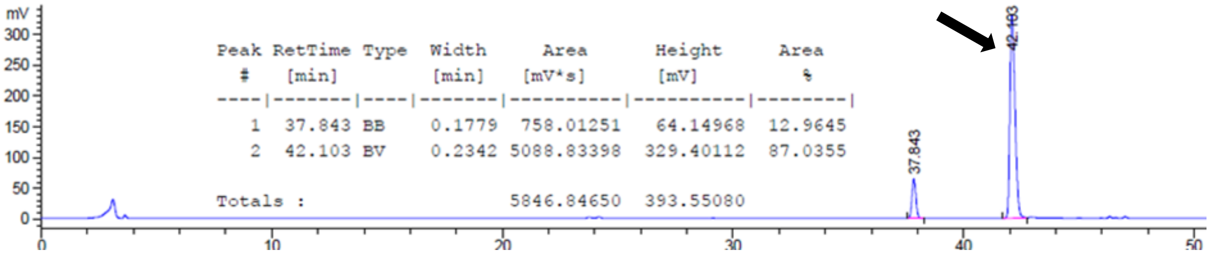

LC-MS chromatogram of **30b**

^{1}H NMR (600 MHz, $CDCl_3$) δ 7.93 (dd, J = 8.2, 1.0 Hz, 2H), 7.84 – 7.79 (m, 2H), 7.52 – 7.45 (m, 4H), 7.41 – 7.24 (m, 22H), 7.15 (dd, J = 7.3, 1.8 Hz, 2H), 7.12 – 7.06 (m, 3H), 5.68 (t, J = 9.6 Hz, 1H, H-3), 5.31 (dd, J = 9.9, 8.0 Hz, 1H, H-2), 5.16 (d, J = 3.1 Hz, 1H, H'-1), 5.07 (d, J = 10.0 Hz, 3H, C*H*HPh, CH_2Ph of Cbz), 4.89 (d, J = 10.9 Hz, 1H, C*H*HPh), 4.85 (dd, J = 11.2, 3.6 Hz, 2H, 2 x C*H*HPh), 4.78 (d, J = 11.7 Hz, 1H, C*H*HPh), 4.61 (d, J = 7.8 Hz, 2H, H-1, NHCbz), 4.59 (s, 2H, CH_2Ph), 4.56 (d, J = 10.9 Hz, 1H, C*H*HPh), 4.30 – 4.22 (m, 2H, H'-6), 4.05 (t, J = 9.2 Hz, 1H, H'-3), 3.99 (t, J = 9.5 Hz, 1H, H-4), 3.96 – 3.88 (m, 3H, H-6, H'-5), 3.88 – 3.83 (m, 1H, OCHH, linker), 3.72 – 3.67 (m, 1H, H-5), 3.62 (dd, J = 9.6, 3.5 Hz, 1H, H'-2), 3.53 (t, J = 9.4 Hz, 1H, H'-4), 3.45 (dd, J = 14.8, 7.0 Hz, 1H, OCHH, linker), 2.89 (m, 2H, CH_2NHCbz), 2.03 (s, 3H, Ac), 1.53 – 1.37 (m, 2H), 1.33 – 1.25 (m, 2H), 1.18 (m, 2H). ^{13}C NMR (150 MHz, $CDCl_3$) δ 170.86 (Ac), 165.82 (Bz), 165.27 (Bz), 156.40 (Cbz), 138.76, 138.22, 138.09, 137.43, 136.88, 133.22, 133.19, 129.92, 129.83, 129.69, 129.55, 128.75, 128.62, 128.57, 128.47, 128.41, 128.40, 128.37, 128.26, 128.19, 128.17, 128.15, 128.14, 128.01, 127.97, 127.82, 101.43 (C-1), 97.08 (C'-1), 81.90 (C'-3), 80.17 (C'-2), 77.33 (C'-4), 75.96 (C-4), 75.81 (CH_2Ph), 75.53 (C-5), 75.20 (CH_2Ph), 75.03 (C-3), 74.78 (CH_2Ph), 72.90 (CH_2Ph), 72.43 (C-2), 69.94 (OCH_2, linker), 69.01 (C'-5), 66.61 (CH_2Ph, Cbz), 65.06 (C-6), 63.12 (C'-6), 40.97 (CH_2NHCbz), 29.53, 29.10, 23.29, 21.00 (Ac).; MS ESI+-HRMS m/z $[M+Na]^+$ calcd for $C_{69}H_{73}NO_{16}Na$ 1194.4822, found 1194.4798.

_N_-Benzyloxycarbonyl-5-amino-pentyl (6-_O_-benzoyl-2,3,4-tri-_O_-benzyl-α-D-glucopyranosyl)-(1→6)-2,3-di-_O_-benzoyl-β-D-glucopyranoside 30c

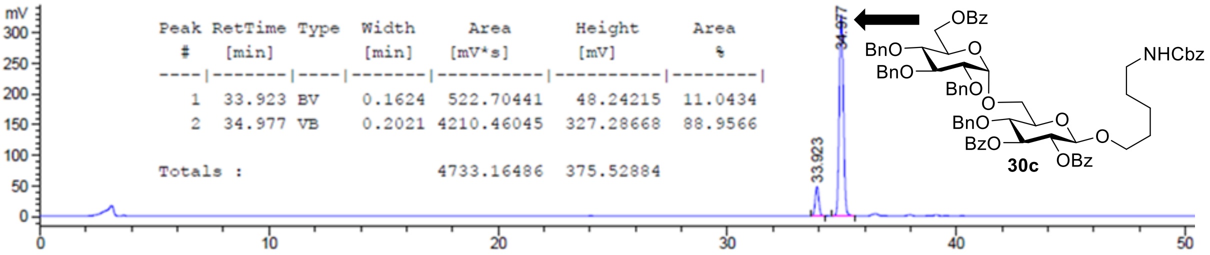

LC-MS chromatogram of **30c**

^{1}H NMR (600 MHz, $CDCl_3$) δ 8.04 (d, J = 7.8 Hz, 2H), 7.93 (d, J = 7.9 Hz, 2H), 7.84 (d, J = 7.9 Hz, 2H), 7.58 (t, J = 7.1 Hz, 1H), 7.52 – 7.44 (m, 6H), 7.41 – 7.20 (m, 22H), 7.16 – 7.06 (m, 5H), 5.68 (t, J = 9.6 Hz, 1H, H-3), 5.33 – 5.28 (m, 1H, H-2), 5.13 (d, J = 3.1 Hz, 1H, H'-1), 5.09 – 5.04 (m, 3H, C*H*HPh, CH_2Ph of Cbz), 4.93 (d, J = 10.9 Hz, 1H, C*H*HPh), 4.88 – 4.83 (m, 2H, 2 x C*H*HPh), 4.79 (d, J = 11.7 Hz, 1H, C*H*HPh), 4.65- 51 (m, 6H, H-1, NH, 2 x C*H*HPh, CH_2Ph), 4.50 (dd, J = 11.9, 4.4 Hz, 1H, C*H*HPh), 4.13 - 05 (m, 2H, H'-3, H'-5), 3.93 – 3.89 (m, 3H, H-4, H-6), 3.89 – 3.84 (m, 1H, OCHH, linker), 3.71 (d, J = 9.6 Hz, 1H, H-5), 3.68 – 3.61 (m, 2H, H'-2, H'-4), 3.44 (d, J = 7.5 Hz, 1H, OCHH, linker), 2.87 (d, J = 5.7 Hz, 2H, CH_2NHCbz), 1.43 (ddt, J = 24.3, 17.9, 8.8 Hz, 2H), 1.26 (dt, J = 12.0, 6.7 Hz, 2H), 1.21 – 1.09 (m, 2H). ^{13}C NMR (150 MHz, $CDCl_3$) δ 166.35 (Bz), 165.80 (Bz), 165.28 (Bz), 156.38 (Cbz), 138.69, 138.25, 138.04, 137.40, 136.89, 133.24, 133.22, 130.10, 129.91, 129.85, 129.84, 129.70, 129.57, 128.75, 128.61, 128.60, 128.57, 128.47, 128.42, 128.34, 128.26, 128.14, 127.99, 127.88, 101.36 (C-1), 96.97 (C'-1), 82.03 (C'-3), 80.38 (C'-2), 77.73 (C'-4), 76.16 (C-4), 75.97 (CH_2Ph), 75.40 (C-5), 75.34 (CH_2Ph), 75.06 (C-3), 74.81 (CH_2Ph), 72.99 (CH_2Ph), 72.45 (C-2), 69.93 (OCH_2, linker), 69.22 (C'-5), 66.59 (CH_2Ph), 65.20 (C-6), 63.59 (C'-6), 40.95 (CH_2Ph, CBz), 29.50, 29.08, 23.28.; MS ESI+-HRMS m/z $[M+Na]^+$ calcd for $C_{74}H_{75}NO_{16}Na$ 1256.4978, found 1256.4967.

***N*-Benzyloxycarbonyl-5-amino-pentyl (2,3,4-tri-*O*-benzyl-α-D-glucopyranosyl)-(1→6)-2,3-di-*O*-benzoyl-β-D-glucopyranoside 30d**

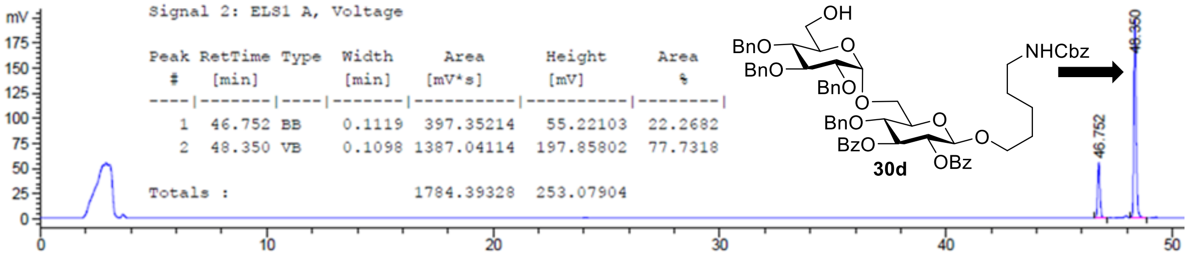

LC-MS chromatogram of **30d**

^{1}H NMR (600 MHz, $CDCl_3$) δ 7.93 (d, *J* = 7.9 Hz, 2H), 7.81 (d, *J* = 7.5 Hz, 2H), 7.52 – 7.45 (m, 5H), 7.42 – 7.25 (m, 21H), 7.15 (d, *J* = 6.9 Hz, 2H), 7.08 (d, *J* = 6.1 Hz, 3H), 5.68 (t, *J* = 9.5 Hz, 1H, H-3), 5.32 (t, *J* = 8.9 Hz, 1H, H-2), 5.16 (br, 1H, H'-1), 5.07 (s, 2H, Cbz), 5.05 (d, *J* = 11.0 Hz, 1H, C*H*HPh), 4.90 (t, *J* = 9.3 Hz, 1H, C*H*HPh), 4.88 – 4.82 (m, 2H, 2 x C*H*HPh), 4.79 (d, *J* = 11.7 Hz, 1H, C*H*HPh), 4.67 – 4.56 (m, 5H, H-1, NH, C*H*HPh, CH_2Ph), 4.06 (t, *J* = 9.1 Hz, 1H, H'-3), 4.01 (t, *J* = 9.4 Hz, 1H, H-4), 3.94 (d, *J* = 13.0 Hz, 2H, H-6), 3.85 (s, 1H, OCHH, linker), 3.78 (t, *J* = 9.4 Hz, 2H, H-5, H'-6), 3.74 – 3.65 (m, 2H, H'-5, H'-6), 3.62 – 3.55 (m, 2H, H'-2, H'-4), 3.49 (d, *J* = 7.7 Hz, 1H, OCHH, linker), 2.91 (d, *J* = 5.9 Hz, 2H, CH_2NHCbz), 1.47 (s, 2H), 1.37 – 1.12 (m, 4H). ^{13}C NMR (150 MHz, $CDCl_3$) δ 165.81 (Bz), 165.27 (Bz), 156.40 (Cbz), 138.90, 138.39, 138.29, 137.47, 136.82, 133.21, 133.16, 129.92, 129.84, 129.69, 129.55, 128.75, 128.64, 128.57, 128.52, 128.46, 128.39, 128.28, 128.20, 128.13, 128.07, 127.93, 127.72, 101.47 (C-1), 97.11 (C'-1), 81.80 (C'-3), 80.30 (C'-2 or C'-4), 77.49 (C'-2 or C'-4), 75.93 (C-4), 75.72 (CH_2Ph), 75.62 (C'-5), 75.21 (CH_2Ph), 75.01 (C-3), 74.76 (CH_2Ph), 72.98 (CH_2Ph), 72.43 (C-2), 71.20 (C-5), 69.94 (OCH_2, linker), 66.67 (CH_2Ph), 64.86 (C-6), 61.95 (C'-6), 41.00 (CH_2Ph, Cbz), 29.50, 29.12, 23.28. MS ESI+-HRMS m/z $[M+Na]^+$ calcd for $C_{67}H_{71}NO_{15}Na$ 1152.4716, found 1152.4707.

_N_-Benzyloxycarbonyl-5-amino-pentyl (3-_O_-acetyl-2,4,6-tri-_O_-benzyl-α-D-glucopyranosyl)-(1→6)-2,3-di-_O_-benzoyl-β-D-glucopyranoside 30e

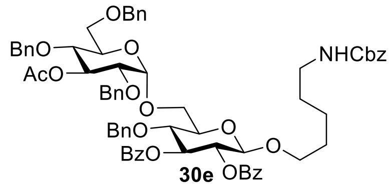

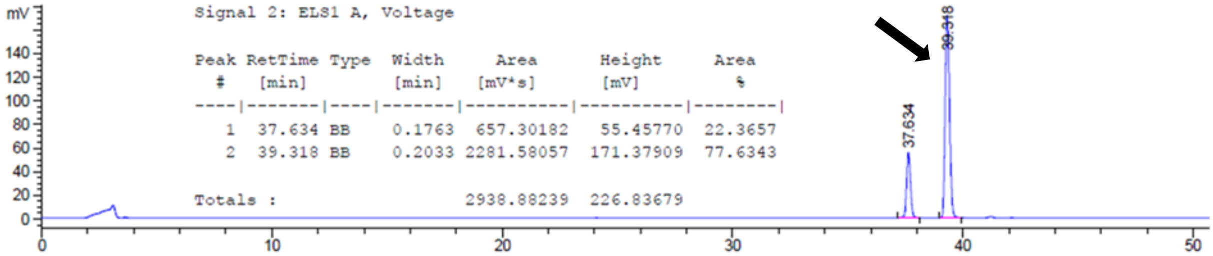

LC-MS chromatogram of **30e**

^{1}H NMR (600 MHz, $CDCl_3$) δ 7.93 – 7.90 (m, 2H), 7.81 (d, J = 7.2 Hz, 2H), 7.50 – 7.44 (m, 2H), 7.40 – 7.22 (m, 22H), 7.16 – 7.07 (m, 7H), 5.66 (t, J = 9.6 Hz, 1H, H-3), 5.59 (t, J = 9.6 Hz, 1H, H'-3), 5.24 (dd, J = 9.9, 8.0 Hz, 1H, H-2), 5.19 (d, J = 3.3 Hz, 1H, H'-1), 5.06 (s, 2H, CH_2Ph, Cbz), 4.78 (d, J = 12.4 Hz, 1H, C*H*HPh), 4.73 (br, 1H, NHCbz), 4.64 (m, 2H, , 2 x CHHPh), 4.61 – 4.55 (m, 3H, H-1, CH_2Ph), 4.48 (dd, J = 11.5, 6.0 Hz, 2H, 2 x C*H*HPh), 4.40 (d, J = 11.1 Hz, 1H, C*H*HPh), 3.95 – 3.87 (m, 3H, H-4, H'-5, H'-6), 3.86 – 3.80 (m, 2H, H'-6, OCH_2 of linker), 3.73 (dd, J = 10.7, 3.1 Hz, 1H, H-6), 3.71 – 3.66 (m, 2H, H-5, H'-4), 3.64 (dd, J = 10.6, 1.6 Hz, 1H, H-6), 3.53 (dd, J = 10.0, 3.4 Hz, 1H, H'-2), 3.45 (dd, J = 15.8, 6.6 Hz, 1H OCH_2, linker), 2.89 (dd, J = 12.8, 6.5 Hz, 2H, CH_2NHCbz), 1.98 (s, 3H, Ac), 1.49 – 1.35 (m, 2H), 1.33 – 1.21 (m, 2H), 1.20 – 1.10 (m, 2H). ^{13}C NMR (150 MHz, $CDCl_3$) δ 170.24 (Ac), 165.83 (Bz), 165.27 (Bz), 156.43 (Cbz), 138.13, 138.11, 137.92, 137.49, 136.94, 133.18, 133.16, 129.93, 129.84, 129.71, 129.53, 128.64, 128.61, 128.58, 128.48, 128.45, 128.40, 128.39, 128.33, 128.24, 128.15, 127.97, 127.92, 127.88, 127.84, 127.84, 101.29 (C-1), 96.79 (C'-1), 77.34 (C'-2), 76.27 (C-5 or C'-4), 76.19 (C-4), 75.47 (C-5 or C'-4), 75.06 (C-3), 74.94 (CH_2Ph), 74.55 (CH_2Ph), 73.73 (C'-3), 73.68 (CH_2Ph), 72.45 (CH_2Ph), 72.36 (C-2), 70.02 (CH_2Ph), 69.96 (C'-5), 68.36 (OCH_2, linker), 66.58 (C-6), 64.96 (C'-6), 41.03 (CH_2Ph, Cbz), 29.44, 29.07, 23.26, 21.27 (Ac).; MS ESI+-HRMS m/z $[M+Na]^+$ calcd for $C_{69}H_{73}NO_{16}Na$ 1194.4822, found 1194.4825.

_N_-Benzyloxycarbonyl-5-amino-pentyl (3-_O_-benzoyl-2,4,6-tri-_O_-benzyl-α-ᴅ-glucopyranosyl)-(1→6)-2,3-di-_O_-benzoyl-β-ᴅ-glucopyranoside 30f

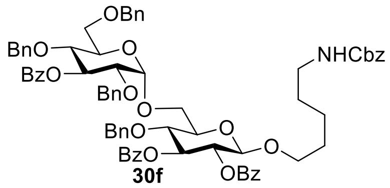

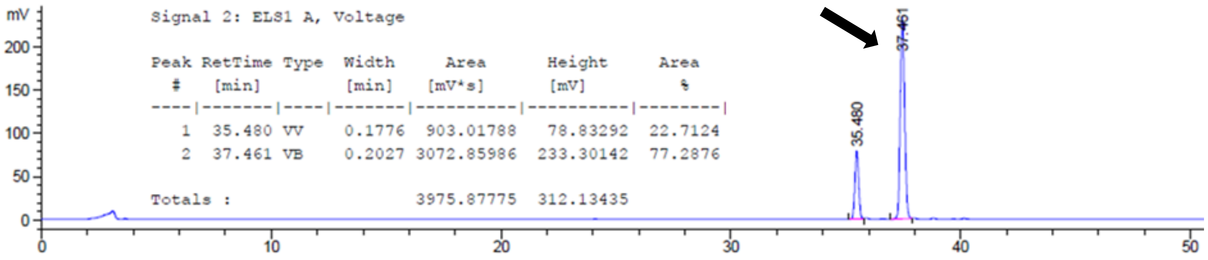

LC-MS chromatogram of **30f**

^{1}H NMR (600 MHz, $CDCl_3$) δ 8.05 (d, _J_ = 7.8 Hz, 2H), 7.92 (d, _J_ = 8.1 Hz, 2H), 7.84 (d, _J_ = 8.1 Hz, 2H), 7.59 (t, _J_ = 7.0 Hz, 1H), 7.47 (dt, _J_ = 14.1, 4.1 Hz, 5H), 7.38 – 7.29 (m, 13H), 7.26 (dd, _J_ = 6.6, 2.1 Hz, 1H), 7.18 (dd, _J_ = 7.8, 6.8 Hz, 5H), 7.15 – 7.08 (m, 7H), 6.97 (d, _J_ = 5.0 Hz, 2H), 5.87 (t, _J_ = 9.5 Hz, 1H, H'-3), 5.69 (t, _J_ = 9.6 Hz, 1H, H-3), 5.29 – 5.25 (m, 1H, H-2), 5.24 (d, _J_ = 3.2 Hz, 1H, H'-1), 5.06 (s, 2H, Cbz), 4.76 (d, _J_ = 12.5 Hz, 1H, C_H_HPh), 4.72 (br, 1H, NHCbz), 4.67 (d, _J_ = 12.1 Hz, 1H, C_H_HPh), 4.64 – 4.59 (m, 4H, H-1, CH_2Ph, C_H_HPh), 4.52 – 4.45 (m, 2H, 2 x C_H_HPh), 4.37 (d, _J_ = 10.8 Hz, 1H, C_H_HPh), 3.98 (dd, _J_ = 12.2, 5.8 Hz, 2H, H-4, H'-5), 3.94 – 3.82 (m, 4H, H'-4, H'-6, OCHH of linker), 3.77 (dd, _J_ = 10.7, 2.7 Hz, 1H, H-6), 3.72 (dd, _J_ = 9.6, 4.2 Hz, 1H, H-5), 3.67 (dd, _J_ = 9.6, 4.1 Hz, 2H, H-6, H'-2), 3.48 (dd, _J_ = 15.4, 6.5 Hz, 1H, OC_H_H, linker), 2.89 (dt, _J_ = 13.0, 6.5 Hz, 2H, CH_2NHCbz), 1.51 – 1.39 (m, 2H), 1.32 – 1.19 (m, 2H), 1.21 – 1.12 (m, 2H). ^{13}C NMR (150 MHz, $CDCl_3$) δ 165.85 (Bz), 165.83 (Bz), 165.28 (Bz), 156.43 (Cbz), 137.88, 137.79, 137.50, 136.94, 133.18, 133.09, 130.44, 129.94, 129.83, 129.70, 129.53, 128.60, 128.59, 128.51, 128.45, 128.40, 128.39, 128.30, 128.20, 128.12, 128.09, 128.02, 127.99, 127.86, 127.81, 127.75, 101.33 (C-1), 96.86 (C'-1), 76.96 (C'-2), 76.18 (C-4 or C'-5), 76.14 (C'-4), 75.49 (C'-4), 75.09 (C-5), 74.96 (CH_2Ph), 74.67 (CH_2Ph), 74.53 (C'-3), 73.74 (CH_2Ph), 72.44 (C-2), 72.25 (CH_2Ph), 69.99 (C-4 or C'-5), 68.39, 66.55, 65.00, 41.01, 29.47, 29.08, 23.24.; MS ESI+-HRMS m/z $[M+Na]^+$ calcd for $C_{74}H_{75}NO_{16}Na$ 1256.4978, found 1256.4927.

N-Benzyloxycarbonyl-5-amino-pentyl (2,4,6-tri-O-benzyl-α-D-glucopyranosyl)-(1→6)-(2,3-di-O-benzoyl-β-D-glucopyranoside 30g

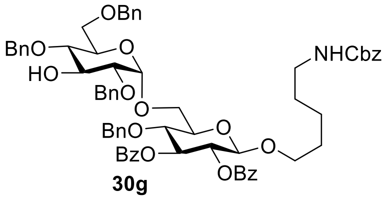

30g

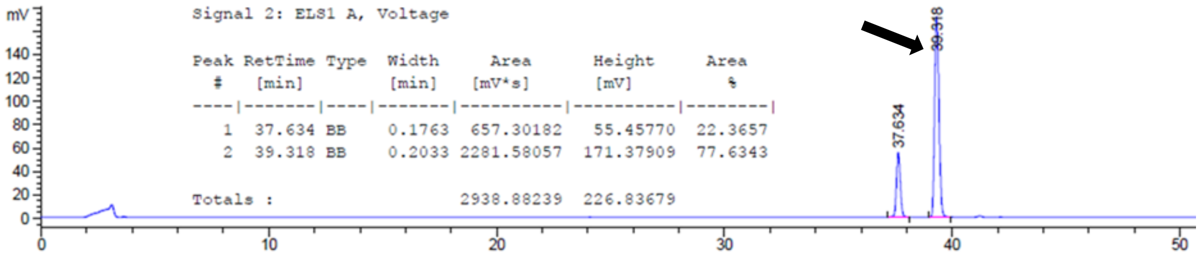

LC-MS chromatogram of **30g**

^{1}H NMR (400 MHz, $CDCl_3$) δ 7.93 (d, J = 8.0 Hz, 2H), 7.86 (d, J = 7.7 Hz, 2H), 7.48 (dd, J = 13.3, 6.1 Hz, 4H), 7.42 – 7.22 (m, 20H), 7.19 (d, J = 7.7 Hz, 2H), 7.10 (s, 5H), 5.69 (t, J = 9.6 Hz, 1H, H-3), 5.30 (dd, J = 9.7, 8.1 Hz, 1H, H-2), 5.14 (d, J = 2.6 Hz, 1H, H'-1), 5.06 (s, 2H, Cbz), 4.86 (d, J = 5.8 Hz, 1H, C*H*HPh), 4.83 (d, J = 6.4 Hz, 1H, C*H*HPh), 4.70 (d, J = 11.7 Hz, 1H, C*H*HPh), 4.62 (t, J = 9.2 Hz, 3H, H-1, 2 x C*H*HPh), 4.55 (s, 2H, CH_2Ph), 4.49 (t, J = 11.9 Hz, 2H, 2 x C*H*HPh), 4.17 – 4.09 (m, 1H, H'-3), 3.98 – 3.77 (m, 5H, H-4, H'-5, H'-6, OC*H*H of linker), 3.75 – 3.57 (m, 4H, H-5, H-6, H'-4), 3.52 – 3.40 (m, 2H, H'-2, OC*H*H of linker), 2.87 (dd, J = 12.8, 6.4 Hz, 2H, CH_2NHCbz), 2.61 (s, 1H), 1.50 – 1.35 (m, 2H), 1.33 – 1.19 (m, 2H), 1.19 – 1.05 (m, 2H). ^{13}C NMR (100 MHz, $CDCl_3$) δ 165.65 (Bz), 165.15 (Bz), 156.24 (Cbz), 138.46, 137.89, 137.85, 137.26, 136.68, 133.08, 129.74, 129.69, 129.50, 129.35, 128.67, 128.48, 128.38, 128.32, 128.29, 128.26, 128.13, 128.02, 127.95, 127.83, 127.76, 127.71, 127.62, 101.11 (C-1), 96.50 (C'-1), 79.29 (C'-2), 77.22 (C'-4), 76.13 (C-4), 75.06 (C-5), 74.98 (C-3), 74.81 (CH_2Ph), 74.62 (CH_2Ph), 73.52 (C'-3), 73.40 (CH_2Ph), 72.42 (C-2), 72.26 (CH_2Ph), 69.83 (C'-5), 69.75 (OCH_2, linker), 68.43 (C-6), 66.48 (CH_2Ph, Cbz), 65.05 (C'-6), 40.80 (CH_2NHCbz), 29.32, 28.88, 23.06.; MS ESI+-HRMS m/z $[M+Na]^+$ calcd for $C_{67}H_{71}NO_{15}Na$ 1152.4716, found 1152.4700.

_N_-Benzyloxycarbonyl-5-amino-pentyl (3,6-di-_O_-acetyl-2,4-di-_O_-benzyl-α-D-glucopyranosyl)-(1→6)-2,3-di-_O_-benzoyl-β-D-glucopyranoside 30h

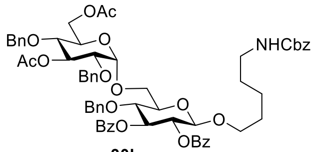

30h

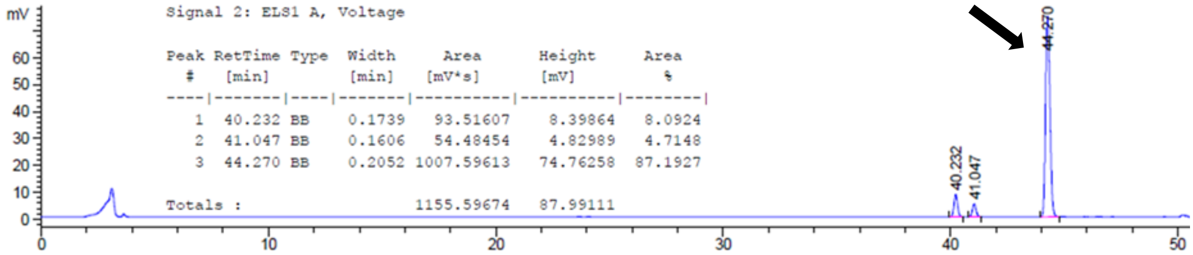

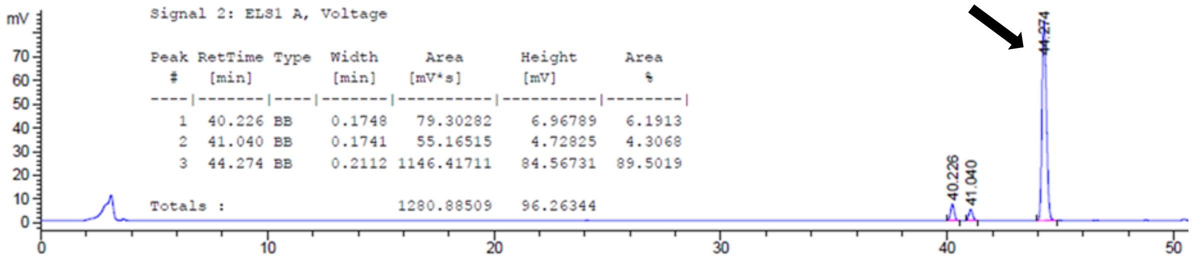

LC-MS chromatogram of **30h** (entry 8 (top) and entry 11 (bottom))

^{1}H NMR (600 MHz, $CDCl_3$) δ 7.91 (d, J = 7.4 Hz, 2H), 7.80 (d, J = 7.4 Hz, 2H), 7.47 (dd, J = 17.7, 7.5 Hz, 2H), 7.40 – 7.23 (m, 19H), 7.17 – 7.08 (m, 5H), 5.65 (dt, J = 19.2, 9.6 Hz, 2H, H-3, H'-3), 5.23 (dd, J = 9.8, 8.1 Hz, 1H, H-2), 5.20 (d, J = 2.9 Hz, 1H, H'-1), 5.06 (s, 2H, Cbz), 4.82 (d, J = 12.4 Hz, 1H, C*H*HPh), 4.71 (br, 1H, NH), 4.64 (d, J = 12.4 Hz, 1H, C*H*HPh), 4.62 – 4.54 (m, 4H, H-1, CH_2Ph, C*H*HPh), 4.50 (d, J = 11.0 Hz, 1H, C*H*HPh), 4.32 – 4.23 (m, 2H, H'-6), 4.00 – 3.94 (m, 2H, H'-3, H'-5), 3.89 (dd, J = 15.3, 7.6 Hz, 2H, H-6), 3.86 – 3.79 (m, 1H, OC*H*H, linker), 3.67 (dd, J = 9.6, 2.3 Hz, 1H, H-5), 3.57 – 3.50 (m, 2H, H'-2, H'-4), 3.49 – 3.42 (m, 1H, OC*H*H, linker), 2.90 (d, J = 6.0 Hz, 2H, CH_2NHCbz), 2.06 (s, 3H, Ac), 2.03 (s, 3H, Ac), 1.50 – 1.37 (m, 2H), 1.35 – 1.22 (m, 2H), 1.22 – 1.09 (m, 2H). ^{13}C NMR (150 MHz, $CDCl_3$) δ 170.78 (Ac), 170.11 (Ac), 165.84 (Bz), 165.25 (Bz), 156.43 (Cbz), 138.02, 137.61, 137.47, 136.92, 133.18, 129.94, 129.84, 129.70, 129.50, 128.70, 128.64, 128.63, 128.47, 128.42, 128.41, 128.37, 128.28, 128.15, 127.94, 127.90, 127.81, 101.42 (C-1), 96.75 (C'-1), 77.22 (C'-2), 76.15 (C'-4), 76.01 (C'-3 or C'-5), 75.58 (C-5), 75.03 (C-3 or C'-3), 75.00 (CH_2Ph), 74.59 (CH_2Ph), 73.64 (C-3 or C'-3), 72.42 (CH_2Ph), 72.32 (C-2), 70.07 (OCH_2, linker), 68.70 (C'-3 or C'-5), 66.60 (Cbz), 65.01 (C-6), 62.94 (C'-

6), 41.02 (CH_2NHCbz), 29.48, 29.11, 23.28, 21.30 (Ac), 21.03 (Ac).; MS ESI+-HRMS m/z $[M+Na]^+$ calcd for $C_{64}H_{69}NO_{17}Na$ 1146.4458, found 1146.4420.

***N*-Benzyloxycarbonyl-5-amino-pentanyl (3,6-di-*O*-acetyl-2,4-di-*O*-benzyl-α-D-glucopyranosyl)-(1→4)-2-*O*-benzoyl-3,6-di-*O*-benzyl-β-D-glucipyranoside 30i**

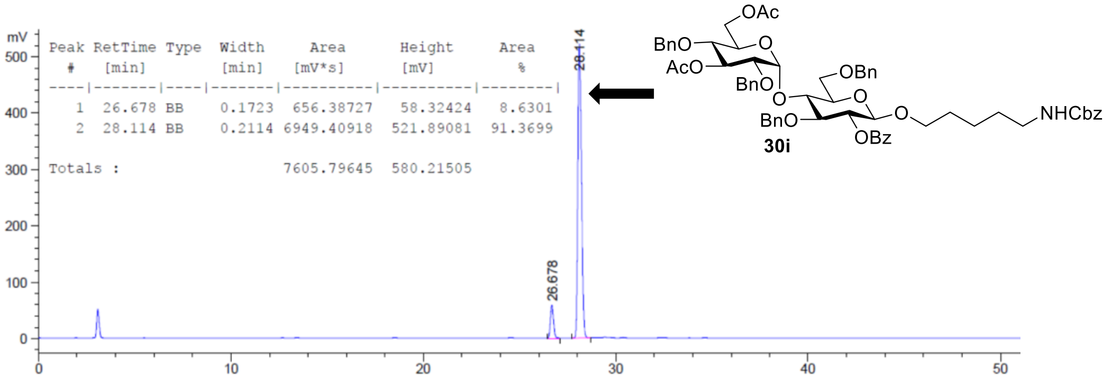

LC-MS chromatogram of **30i** (entry 9)

^{1}H NMR (400 MHz, $CDCl_3$) δ 7.98 (d, *J* = 7.7 Hz, 2H), 7.52 (t, *J* = 7.3 Hz, 1H), 7.42 – 7.21 (m, 20H), 7.10 (d, *J* = 8.1 Hz, 7H), 5.53 (t, *J* = 9.7 Hz, 1H), 5.50 (d, *J* = 3.3 Hz, 1H), 5.34 (t, *J* = 8.4 Hz, 1H), 5.07 (s, 2H), 4.75 (d, *J* = 11.3 Hz, 1H), 4.65 (d, *J* = 11.3 Hz, 1H), 4.61 – 4.53 (m, 4H), 4.53 – 4.41 (m, 3H), 4.25 (d, *J* = 12.4 Hz, 1H), 4.19 – 4.06 (m, 3H), 3.98 (t, *J* = 8.5 Hz, 2H), 3.89 (td, *J* = 12.1, 5.0 Hz, 2H), 3.78 (d, *J* = 10.7 Hz, 1H), 3.63 (d, *J* = 7.2 Hz, 1H), 3.44 (dd, *J* = 21.1, 11.7 Hz, 2H), 3.35 (dd, *J* = 10.1, 3.3 Hz, 1H), 2.91 (dd, *J* = 12.6, 6.3 Hz, 2H), 2.00 (s, 3H), 1.94 (s, 3H), 1.58 – 1.40 (m, 2H), 1.36 – 1.25 (m, 2H), 1.25 – 1.12 (m, 2H). ^{13}C NMR (100 MHz, $CDCl_3$) δ 170.69, 169.99, 165.29, 156.37, 138.23, 138.21, 137.78, 137.54, 136.81, 133.27, 129.94, 129.82, 128.64, 128.62, 128.51, 128.44, 128.21, 128.17, 127.87, 127.84, 127.72, 127.68, 127.41, 127.31, 101.18, 96.75, 82.69, 77.36, 76.99, 76.11, 75.03, 74.56, 74.39, 73.50, 73.37, 73.31, 72.96, 72.48, 69.51, 69.29, 68.85, 66.61, 63.03, 40.93, 29.51, 29.05, 23.24, 21.23, 21.01.; MS ESI+-HRMS m/z $[M+Na]^+$ calcd for $C_{64}H_{71}NO_{17}Na$ 1132.4665, found 1132.4763.

_N_-Benzyloxycarbonyl-5-amino-pentanyl (3,6-di-_O_-acetyl-2,4-di-_O_-benzyl-α-D-glucopyranosyl)-(1→4)-2-_O_-benzoyl-4,6-di-_O_-benzyl-β-D-glucipyranoside 30j

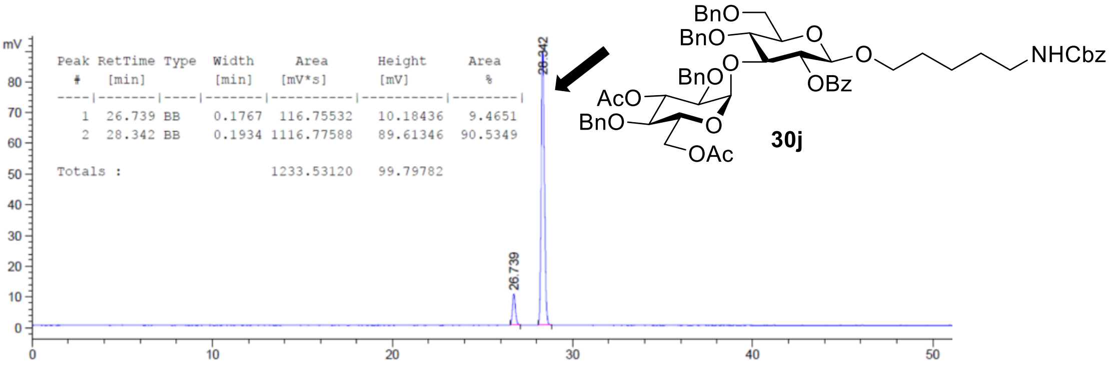

LC-MS chromatogram of **30j** (entry 10)

^{1}H NMR (400 MHz, $CDCl_3$) δ 7.95 (d, J = 7.5 Hz, 2H), 7.50 (t, J = 7.0 Hz, 1H), 7.43 – 7.09 (m, 27H), 5.58 (t, J = 9.6 Hz, 1H), 5.23 (t, J = 8.0 Hz, 1H), 5.15 (d, J = 11.2 Hz, 1H), 5.07 (s, 3H), 4.65 – 4.50 (m, 4H), 4.40 (dt, J = 22.4, 11.1 Hz, 5H), 4.11 (d, J = 12.0 Hz, 1H), 4.06 – 3.91 (m, 3H), 3.91 – 3.83 (m, 1H), 3.81 – 3.66 (m, 3H), 3.65 – 3.54 (m, 1H), 3.46 (t, J = 9.6 Hz, 1H), 3.36 (d, J = 10.3 Hz, 2H), 2.87 (d, J = 5.8 Hz, 2H), 1.91 (s, 3H), 1.88 (s, 3H), 1.57 – 1.36 (m, 2H), 1.34 – 1.21 (m, 2H), 1.21 – 1.05 (m, 2H). ^{13}C NMR (100 MHz, $CDCl_3$) δ 170.50, 169.78, 165.04, 156.37, 138.75, 138.16, 138.03, 137.62, 136.85, 133.11, 130.24, 129.68, 128.61, 128.50, 128.49, 128.44, 128.21, 128.15, 128.03, 127.96, 127.94, 127.90, 127.86, 127.74, 127.63, 127.30, 101.07, 98.65, 83.86, 77.81, 77.36, 77.28, 75.35, 75.13, 74.96, 74.03, 73.63, 73.52, 73.19, 72.88, 69.48, 69.18, 69.04, 66.57, 62.30, 40.89, 29.43, 29.02, 23.18, 21.19, 20.89.; MS ESI+-HRMS m/z $[M+Na]^{+}$ calcd for $C_{64}H_{71}NO_{17}Na$ 1132.4665, found 1132.4768.

3.7 Analysis and Characterization of α-glucans 34 - 38

***N*-Benzyloxycarbonyl-5-amino-pentyl (3-*O*-acetyl-2,4-di-*O*-benzyl-α-D-glucopyranosyl)-(1→6)-(3-*O*-acetyl-2,4-di-*O*-benzyl-α-D-glucopyranosyl)-(1→6)-(3-*O*-acetyl-2,4-di-*O*-benzyl-α-D-glucopyranosyl)-(1→6)-(3-*O*-acetyl-2,4-di-*O*-benzyl-α-D-glucopyranosyl)-(1→6)-2,3-di-*O*-benzoyl-β-D-glucopyranoside 34**

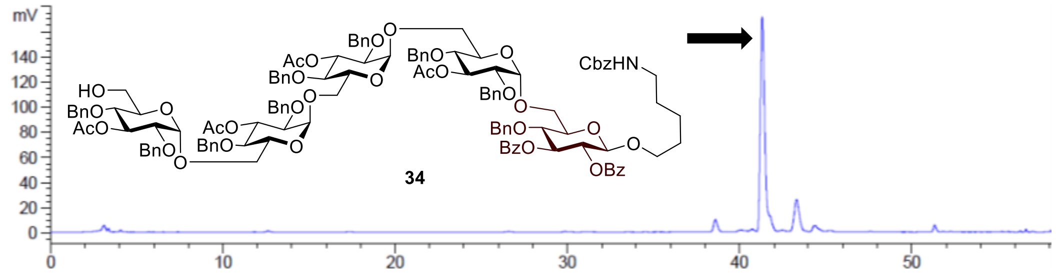

LC-MS chromatogram of **34**

^{1}H NMR (600 MHz, $CDCl_3$) δ 7.92 (d, *J* = 7.9 Hz, 2H), 7.78 (d, *J* = 7.9 Hz, 2H), 7.38 – 7.17 (m, 50H), 7.15 – 7.07 (m, 4H), 5.68 – 5.61 (m, 2H, 2 x H-3), 5.57 – 5.49 (m, 3H, 3 x H-3), 5.25 – 5.20 (m, 1H, H-2), 5.14 (d, *J* = 3.3 Hz, 1H, H-1), 5.06 – 5.03 (m, 4H, 2 x H-1, CH_2 of Cbz), 4.99 (d, *J* = 3.2 Hz, 1H, H-1), 4.72 – 4.47 (m, 15H, H-1), 4.46 – 4.37 (m, 3H), 4.30 (d, *J* = 12.3 Hz, 2H), 3.93 (t, *J* = 9.5 Hz, 1H), 3.88 – 3.57 (m, 21H), 3.43 (dd, *J* = 9.9, 3.2 Hz, 2H), 3.22 – 3.11 (m, 3H), 2.85 (dd, *J* = 12.5, 6.2 Hz, 2H, CH_2NHCbz), 1.98 (s, 3H, Ac), 1.89 (s, 3H, Ac), 1.88 (s, 3H, Ac), 1.87 (s, 3H, Ac), 1.49 – 1.37 (m, 2H, CH_2, pentane), 1.31 – 1.19 (m, 2H, CH_2, pentane), 1.18 – 1.05 (m, 2H, CH_2, pentane). ^{13}C NMR (150 MHz, $CDCl_3$) δ 170.31 (Ac), 170.24 (Ac), 170.23 (Ac), 170.14 (Ac), 165.82 (Bz), 165.26 (Bz), 156.41 (Cbz), 138.44, 138.39, 138.36, 138.27, 138.18, 137.94, 137.59, 136.88, 133.19, 133.13, 129.92, 129.82, 129.70, 129.52, 128.64, 128.61, 128.59, 128.55, 128.54, 128.51, 128.50, 128.49, 128.48, 128.37, 128.35, 128.30, 128.24, 128.19, 128.16, 128.12, 128.10, 127.76, 127.74, 127.70, 127.68, 127.60, 127.56, 127.24, 127.02, 127.01, 101.36 (C-1), 97.18 (2 x C-1), 97.09 (C-1), 96.73 (C-1), 77.86, 77.85, 77.80, 77.63, 76.12, 75.93, 75.87, 75.72, 75.57, 75.02, 74.88, 74.70, 74.68, 74.63, 74.59, 73.55, 73.49, 73.46, 73.43, 72.51, 72.43, 72.03, 71.92, 71.81, 71.20, 70.98, 70.00, 66.58, 65.41, 65.20, 64.89, 61.59, 40.97, 29.42, 29.05, 23.22, 21.28 (Ac), 21.25 (3 x Ac).; MS ESI+-HRMS m/z [M+H]+ calcd for $C_{128}H_{140}NO_{34}$ 2234.9251, found 2234.9198.

***N*-Benzyloxycarbonyl-5-amino-pentyl (6-*O*-acetyl-2,3-di-*O*-benzyl-α-D-glucopyranosyl)-(1→4)-(6-*O*-acetyl-2,3-di-*O*-benzyl-α-D-glucopyranosyl)-(1→4)-(6-*O*-acetyl-2,3-di-*O*-benzyl-α-D-glucopyranosyl)-(1→4)-(6-*O*-acetyl-2,3-di-*O*-benzyl-α-D-glucopyranosyl)-(1→6)-2,3-di-*O*-benzoyl-β-D-glucopyranoside 35**

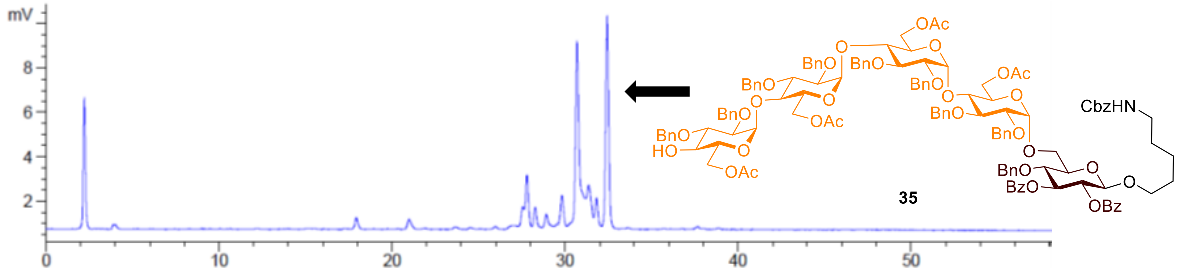

LC-MS chromatogram of **35**

^{1}H NMR (600 MHz, $CDCl_3$) δ 7.93 (d, *J* = 8.1 Hz, 2H), 7.81 (d, *J* = 8.1 Hz, 2H), 7.48 (dt, *J* = 13.2, 7.5 Hz, 2H), 7.42 – 7.01 (m, 54H), 5.69 (t, *J* = 9.6 Hz, 1H, H-3), 5.56 (d, *J* = 3.6 Hz, 1H, H-1), 5.42 (d, *J* = 3.4 Hz, 1H, H-1), 5.38 (d, *J* = 3.4 Hz, 1H, H-1), 5.35 – 5.30 (m, 1H, H-2), 5.28 (d, *J* = 3.2 Hz, 1H, H-1), 5.10 (d, *J* = 11.6 Hz, 1H), 5.06 (br, 2H, CH_2, Cbz), 4.88 – 4.51 (m, 16H, H-1), 4.44 (dt, *J* = 15.3, 10.6 Hz, 6H), 4.35 (t, *J* = 9.6 Hz, 2H), 4.26 (ddd, *J* = 20.9, 11.9, 4.3 Hz, 2H), 4.14 (t, *J* = 9.7 Hz, 2H), 4.08 (t, *J* = 9.5 Hz, 1H), 4.04 – 3.87 (m, 9H), 3.80 (dt, *J* = 15.3, 9.1 Hz, 4H), 3.70 – 3.64 (m, 2H), 3.50 – 3.39 (m, 4H), 2.91 – 2.84 (m, 2H, CH_2NHCbz), 2.70 (s, 1H, OH), 2.11 (s, 3H, Ac), 2.10 (s, 3H, Ac), 2.08 (s, 3H, Ac), 2.07 (s, 3H, Ac), 1.54 – 1.40 (m, 2H, CH_2, pentane), 1.33 – 1.22 (m, 2H, CH_2, pentane), 1.22 – 1.11 (m, 2H, CH_2, pentane). ^{13}C NMR (150 MHz, $CDCl_3$) δ 171.62 (Ac), 170.88 (Ac), 170.73 (Ac), 170.71 (Ac), 165.84 (Bz), 165.23 (Bz), 156.37 (Cbz), 139.10, 138.90, 138.74, 138.07, 137.90, 137.50, 136.85, 133.17, 129.95, 129.82, 129.67, 129.54, 128.74, 128.63, 128.55, 128.47, 128.38, 128.31, 128.24, 128.11, 128.04, 127.98, 127.74, 127.71, 127.66, 127.27, 127.22, 127.17, 126.70, 126.55, 126.53, 101.55 (C-1), 98.26 (C-1), 97.38 (C-1), 97.27 (C-1), 96.77 (C-1), 81.24, 81.06, 80.78, 80.73, 80.00, 79.04, 78.95, 78.92, 75.66, 75.55, 75.34, 74.98, 74.82, 74.18, 74.17, 73.42, 73.32, 73.28, 72.67, 72.42, 70.89, 70.34, 70.01, 69.57, 68.58, 66.62, 64.72, 63.64, 63.55, 63.31, 40.94, 29.54, 29.11, 23.31, 21.13 (Ac), 21.12 (Ac), 21.06 (Ac), 21.00 (Ac).; MS ESI+-HRMS m/z [M+H]+ calcd for $C_{128}H_{140}NO_{34}$ 2234.9251, found 2234.9142.

_N_-Benzyloxycarbonyl-5-amino-pentyl (3,6-di-_O_-acetyl-2,4-di-_O_-benzyl-α-D-glucopyranosyl)-(1→3)-(6-_O_-acetyl-2,4-di-_O_-benzyl-α-D-glucopyranosyl)-(1→3)-(6-_O_-acetyl-2,4-di-_O_-benzyl-α-D-glucopyranosyl)-(1→3)-(6-_O_-acetyl-2,4-di-_O_-benzyl-α-D-glucopyranosyl)-(1→3)-2,3-di-_O_-benzoyl-β-D-glucopyranoside 36

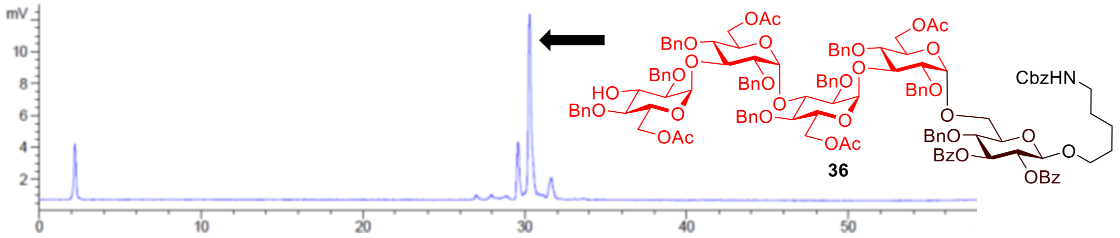

LC-MS chromatogram of **36**

^{1}H NMR (600 MHz, $CDCl_3$) δ 7.93 (d, J = 7.3 Hz, 2H), 7.88 (d, J = 7.5 Hz, 2H), 7.50 – 7.43 (m, 4H), 7.40 – 7.05 (m, 47H), 6.98 (s, 5H), 5.71 (t, J = 9.4 Hz, 1H, H-3), 5.67 (d, J = 0.4 Hz, 1H, H-1), 5.60 (d, J = 1.3 Hz, 1H, H-1), 5.52 (d, J = 1.3 Hz, 1H, H-1), 5.30 (t, J = 8.6 Hz, 1H, H-2), 5.06 (s, 3H, H-1, CH_2 of Cbz), 4.79 (dd, J = 18.8, 11.8 Hz, 2H), 4.73 – 4.22 (m, 25H, H-1), 4.17 (d, J = 11.8 Hz, 1H), 4.07 (dd, J = 30.6, 9.5 Hz, 3H), 3.86 (dt, J = 30.1, 18.3 Hz, 8H), 3.72 – 3.41 (m, 8H), 3.32 (dd, J = 18.1, 8.9 Hz, 2H), 2.86 (br, 2H, CH_2NHCbz), 2.05 (s, 3H, Ac), 2.05 (s, 3H, Ac), 2.03 (s, 3H, Ac), 1.97 (s, 3H, Ac), 1.47 – 1.34 (m, 2H, CH_2, pentane), 1.34 – 1.18 (m, 2H, CH_2, pentane), 1.17 – 1.05 (m, 2H, CH_2, pentane). ^{13}C NMR (150 MHz, $CDCl_3$) δ 170.80 (Ac), 170.76 (Ac), 170.70 (Ac), 170.61 (Ac), 165.83 (Bz), 165.23 (Bz), 156.36 (Cbz), 138.38, 138.06, 137.88, 137.79, 137.73, 137.65, 137.55, 137.32, 136.89, 133.22, 129.90, 129.81, 129.65, 129.46, 128.76, 128.60, 128.56, 128.45, 128.36, 128.28, 128.23, 128.14, 128.08, 127.97, 127.90, 127.74, 127.63, 127.53, 127.48, 127.00, 126.86, 126.74, 101.34 (C-1), 96.83 (C-1), 96.37 (C-1), 96.14 (C-1), 95.90 (C-1), 79.30, 79.24, 78.91, 78.63, 78.57, 78.20, 77.37, 77.16, 76.95, 76.56, 76.40, 76.09, 75.24, 75.18, 75.04, 74.95, 74.33, 73.76, 73.46, 73.42, 73.15, 72.96, 72.58, 72.45, 72.38, 69.93, 68.21, 68.16, 68.10, 66.56, 65.66, 63.25, 63.00, 40.90, 29.43, 29.03, 23.22, 21.12 (Ac), 21.09 (Ac), 21.05 (Ac).; MS ESI+-HRMS m/z [M+Na]+ calcd for $C_{129}H_{140}NO_{34}$ 2234.9251, found 2234.9178.

_N_-Benzyloxycarbonyl-5-amino-pentyl (3,6-di-_O_-acetyl-2,3-di-_O_-benzyl-α-D-glucopyranosyl)-(1→3)-(6-_O_-acetyl-2,4-di-_O_-benzyl-α-D-glucopyranosyl)-(1→6)-(3-_O_-acetyl-2,3-di-_O_-benzyl-α-D-glucopyranosyl)-(1→6)-(3-_O_-acetyl-2,3-di-_O_-benzyl-α-D-glucopyranosyl)-(1→6)-2,3-di-_O_-benzoyl-α-D-glucopyranoside 37

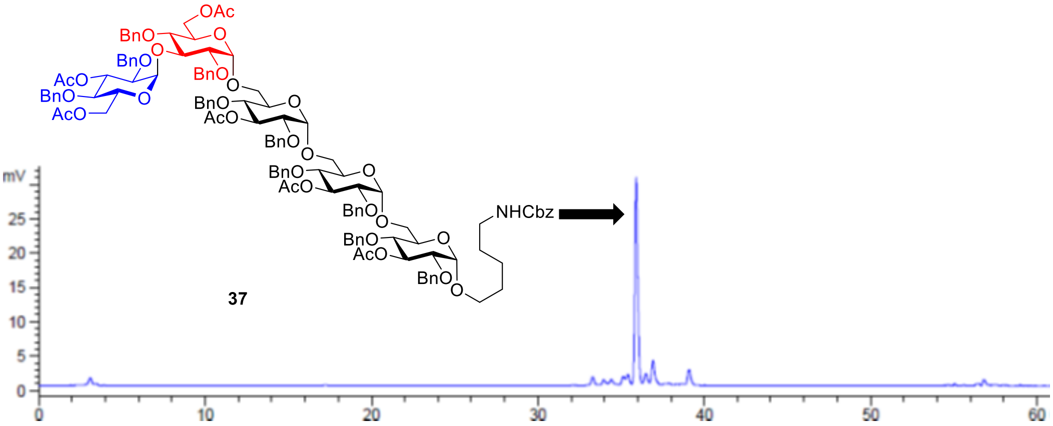

LC-MS chromatogram of **37**

^{1}H NMR (600 MHz, $CDCl_3$) δ 7.37 – 7.13 (m, 53H), 7.09 – 7.05 (m, 2H), 5.65 (t, _J_ = 9.7 Hz, 1H, H-3), 5.59 (t, _J_ = 9.6 Hz, 1H, H-3), 5.55 (d, _J_ = 3.4 Hz, 1H, H-1), 5.51 – 5.46 (m, 1H, H-3), 5.43 (t, _J_ = 9.6 Hz, 1H, H-3), 5.19 (d, _J_ = 3.4 Hz, 1H, H-1), 5.06 (s, 2H, CH_2, Cbz), 5.04 (d, _J_ = 3.3 Hz, 1H, H-1), 4.93 (d, _J_ = 11.7 Hz, 1H), 4.90 (d, _J_ = 3.3 Hz, 1H, H-1), 4.67 – 4.56 (m, 8H, H-1, 7 x CHHPh), 4.50 – 4.32 (m, 12H), 4.28 – 4.22 (m, 3H), 4.07 (dd, _J_ = 12.1, 3.9 Hz, 1H), 3.96 – 3.92 (m, 1H), 3.86 – 3.74 (m, 10H), 3.70 – 3.61 (m, 6H), 3.55 (dd, _J_ = 9.6, 3.4 Hz, 1H, H’’’-2), 3.52 – 3.43 (m, 2H), 3.30 (dd, _J_ = 15.6, 6.4 Hz, 1H), 3.25 (dd, _J_ = 10.0, 3.4 Hz, 1H), 3.17 – 3.07 (m, 4H), 2.02 (s, 3H, Ac), 1.99 (s, 3H, Ac), 1.94 (s, 3H, Ac), 1.91 (s, 3H, Ac), 1.86 (s, 3H, Ac), 1.82 (s, 3H, Ac), 1.63 – 1.55 (m, 2H, CH_2, pentane), 1.54 – 1.44 (m, 2H, CH_2, pentane), 1.41 – 1.31 (m, 2H, CH_2, pentane). ^{13}C NMR (150 MHz, $CDCl_3$) δ 170.75 (Ac), 170.53 (Ac), 170.36 (Ac), 170.21 (Ac), 170.11 (Ac), 170.03 (Ac), 156.58, 138.38, 138.31, 138.24, 138.17, 138.11, 137.93, 137.62, 137.54, 136.99, 128.60, 128.57, 128.52, 128.47, 128.45, 128.36, 128.22, 128.17, 128.08, 128.04, 128.00, 127.90, 127.87, 127.82, 127.75, 127.60, 127.09, 127.02, 126.86, 97.35 (C-1), 97.14 (C-1), 96.97 (C-1), 96.69 (C-1), 96.44 (C-1), 78.70, 78.05, 78.01, 77.97, 77.93, 77.87, 76.81, 76.31, 76.06, 75.68, 74.74, 74.58, 74.55, 74.18, 73.90, 73.81, 73.59, 73.53, 73.42, 72.63, 72.02, 71.95, 71.11, 70.91, 70.75, 68.65, 68.26, 66.54, 65.68, 65.23, 64.67, 62.74, 41.10, 29.66, 29.00, 23.61, 21.29 (Ac), 21.23 (Ac), 21.21 (Ac), 21.19 (Ac), 21.05 (Ac), 21.02 (Ac). ; MS ESI+-HRMS m/z $[M+NH_4]^+$ calcd for $C_{125}H_{145}N_2O_{34}$ 2217.9673, found 2217.9589.

***N*-Benzyloxycarbonyl-5-amino-pentyl (3,6-di-*O*-acetyl-2,3-di-*O*-benzyl-α-D-glucopyranosyl)-(1→6)-(3-*O*-acetyl-2,4-di-*O*-benzyl-α-D-glucopyranosyl)-(1→4)-(6-*O*-acetyl-2,3-di-*O*-benzyl-α-D-glucopyranosyl)-(1→4)-6-*O*-acetyl-2,3-di-*O*-benzyl-α-D-glucopyranoside 38**

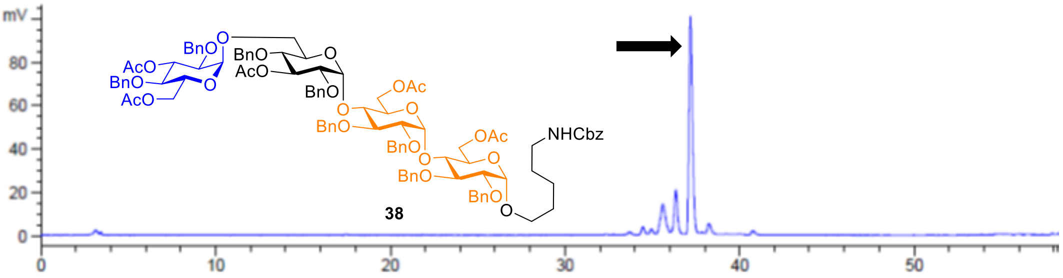

LC-MS chromatogram of **38**

^{1}H NMR (600 MHz, $CDCl_3$) δ 7.38 – 7.17 (m, 32H), 7.17 – 7.07 (m, 9H), 7.04 (d, *J* = 7.5 Hz, 2H), 6.93 (d, *J* = 6.6 Hz, 2H), 5.63 (t, *J* = 9.4 Hz, 1H, H-3), 5.42 (d, *J* = 2.5 Hz, 1H, H-1), 5.41 (d, *J* = 2.0 Hz, 1H, H-1), 5.36 (t, *J* = 9.5 Hz, 1H, H-3), 5.22 (d, *J* = 1.3 Hz, 1H, H-1), 5.09 (br, 2H, CH_2, Cbz), 5.00 (d, *J* = 11.7 Hz, 1H), 4.90 (s, 1H, NHCbz), 4.81 (t, *J* = 10.7 Hz, 2H), 4.70 (d, *J* = 1.4 Hz, 1H, H-1), 4.67 – 4.49 (m, 9H), 4.44 – 4.38 (m, 4H), 4.33 – 4.27 (m, 2H), 4.22 (s, 2H), 4.18 (dd, *J* = 12.2, 3.5 Hz, 1H), 4.03 (dd, *J* = 19.0, 10.4 Hz, 2H), 3.98 – 3.84 (m, 6H), 3.82 – 3.68 (m, 5H), 3.66 (dd, *J* = 14.9, 6.9 Hz, 1H), 3.52 (t, *J* = 9.7 Hz, 2H), 3.48 – 3.41 (m, 2H), 3.38 (dd, *J* = 15.3, 6.9 Hz, 1H), 3.21 (d, *J* = 6.2 Hz, 2H, CH_2NHCbz), 2.96 (dd, *J* = 10.1, 2.3 Hz, 1H, H-2), 2.06 (s, 3H, Ac), 2.05 (s, 6H, 2 x Ac), 2.00 (s, 3H, Ac), 1.73 (s, 3H, Ac), 1.72 – 1.65 (m, 2H, CH_2, pentane), 1.61 – 1.53 (m, 2H, CH_2, pentane), 1.48 – 1.38 (m, 2H, CH_2, pentane). ^{13}C NMR (150 MHz, $CDCl_3$) δ 170.78 (Ac), 170.62 (Ac), 170.08 (Ac), 170.05 (Ac), 156.55 (Cbz), 139.33, 139.17, 138.23, 138.20, 138.13, 137.90, 137.79, 137.60, 136.84, 128.65, 128.63, 128.56, 128.54, 128.35, 128.31, 128.29, 128.23, 128.16, 128.15, 128.05, 127.85, 127.77, 127.70, 127.64, 127.32, 127.11, 127.08, 126.92, 126.65, 126.35 (Ar), 97.66 (C-1), 97.47 (C-1), 97.04 (C-1), 96.46 (C-1), 81.26, 80.88, 80.24, 79.23, 77.62, 77.37, 77.32, 77.16, 76.95, 76.19, 76.16, 75.40, 74.97, 74.64, 74.56, 74.42, 73.93, 73.50, 73.34, 73.31, 73.18, 73.13, 72.17, 71.24, 69.16, 68.74, 68.31, 68.27, 66.69, 65.06, 63.74, 63.64, 62.99, 41.06, 29.89, 29.05, 23.51, 21.28 (Ac), 21.11 (2 x Ac), 21.01 (2 x Ac).; MS ESI+-HRMS m/z $[M+NH_4]^+$ calcd for $C_{103}H_{118}NO_{28}$ 1816.7835, found 1816.7816.

3.8 Post-Automation Steps: Removal of Protecting Groups, and Final Purification

Deprotection Conditions: To a solution of the fully protected oligosaccharide **4** in MeOH (5 mL) was added 58 µL of 0.5 M NaOMe solution (0.25 eq. per acetyl or benzoyl group) in MeOH at 40 ° C. The mixture is stirred utill completed, then neutralized by 200 mg of Amberite (400 mg per 100 µL of NaOMe solution) after completion of the reaction. This crude mixture is dissolved in MeOH, ethyl acetate, and AcOH (v/v/v =5:0.5:0.2) added 5% Pd/C (W/V), purged first with argon and then with hydrogen, left to stir overnight at room temperature under balloon pressure. The reaction mixture was filtered through modified cellulose filter, washed with 20 mL of Water/MeOH, 9:1 and the combined solution was evaporated to provide the crude mixture.

Analytical HPLC: The crude material was analyzed by HPLC (column: Hypercarb®, (150 X 4.60 mm); flow rate: 0.8 mL/min; eluents: 0.1% FA in Acetonitrile / 0.1% FA in TDW; gradient: 0% (10 min) 30% (in 30 min) 100% (in 5 min); detection: ELSD).
NOTE: The crude material were used to provide analytical HPLC data in supporting information to identify the conjugation-ready oligosaccharide in the crude mixture followed by deprotection steps from the pure protected oligosaccharide.

Preparative HPLC: The crude solution is purified by preparative HPLC (column: Hypercarb®, (150 X 10.00 mm); flow rate: 3.6 mL/min; eluents: 0.1% FA in Acetonitrile / 0.1% FA in TDW; gradient: 0% (10 min) 30% (in 30 min) 100% (in 5 min); detection: ELSD) to afford the unprotected oligosaccharide.
Note: HPLC purifications using 0.1% formic acid (FA) sometime result in the fromation of formic acid salt with conjugation-ready oligosaccharide. The detection of formic acid by ^{1}H and ^{13}C NMR does not imply of impurity.

5-Amino-pentyl α-D-glucopyranosyl-(1→6)-α-D-glucopyranosyl-(1→6)-α-D-glucopyranosyl-(1→6)-α-D-glucopyranosyl-(1→6)-β-D-glucopyranoside 5

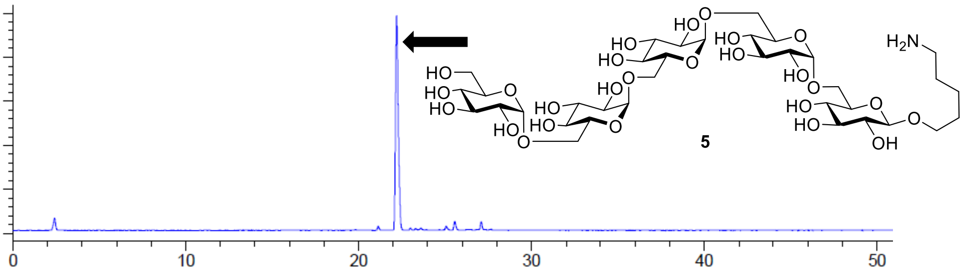

LC-MS chromatogram of **5**

^{1}H NMR (700 MHz, D_2O) δ8.31 (s, 1H, *H*CO_2H), 4.92 – 4.88 (m, 4H, 4 x H-1), 4.42 (d, *J* = 8.0 Hz, 1H, H-1), 3.93 – 3.89 (m, 4H), 3.86 – 3.82 (m, 4H), 3.78 (dd, *J* = 12.3, 2.2 Hz, 1H), 3.72 – 3.60 (m, 11H), 3.57 (ddd, *J* = 9.8, 4.4, 2.0 Hz, 1H), 3.53 – 3.40 (m, 9H), 3.37 – 3.34 (m, 1H), 3.20 (dd, *J* = 9.2, 8.1 Hz, 1H, H-2), 2.94 (t, *J* = 7.5 Hz, 2H, -CH_2NH_2), 1.66 – 1.57 (m, 4H, $OCH_2CH_2CH_2CH_2CH_2NH_2$), 1.42 – 1.36 (m, 2H, -$CH_2CH_2CH_2$-). ^{13}C NMR (175 MHz, D_2O) δ 168.62 (HCO_2H), 102.31 (C-1), 97.77 (C-1), 97.72 (C-1), 97.67 (2 x C-1), 76.06, 74.10, 73.37, 73.10, 73.06, 71.81, 71.45, 71.36, 70.23, 70.15, 70.12, 69.47, 69.30, 65.52, 65.42, 60.44, 39.34 (CH_2NH_2), 28.18, 26.41, 22.10.; MS ESI+-HRMS m/z $[M+Na]^+$ calcd for $C_{35}H_{64}NO_{26}$ 914.3711, found 914.3709.

5-Amino-pentyl α-D-glucopyranosyl-(1→4)-α-D-glucopyranosyl-(1→4)-α-D-glucopyranosyl-(1→4)-α-D-glucopyranosyl-(1→4)-β-D-glucopyranoside 6

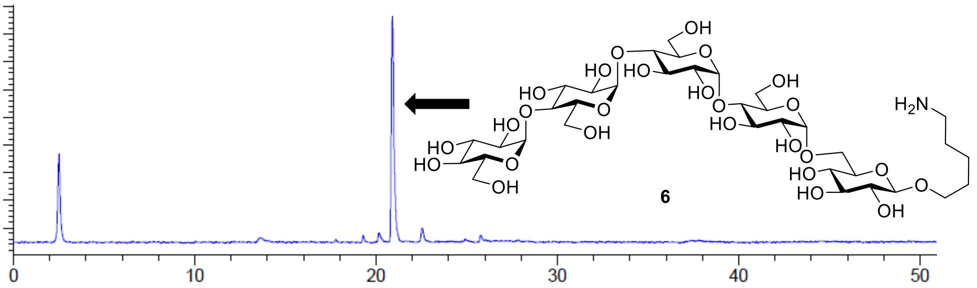

LC-MS chromatogram of **6**

^{1}H NMR (700 MHz, D_2O) δ 8.50 (s, 1H, HCO_2H), 5.40 (d, J = 4.0 Hz, 1H, H-1), 5.39 (d, J = 3.9 Hz, 1H, H-1), 5.37 (d, J = 3.9 Hz, 1H, H-1), 4.98 (d, J = 3.7 Hz, 1H, H-1), 4.52 (d, J = 8.0 Hz, 1H, H-1), 4.03 – 3.99 (m, 1H), 3.97 – 3.82 (m, 14H), 3.81 – 3.77 (m, 2H), 3.76 – 3.71 (m, 2H), 3.71 – 3.60 (m, 9H), 3.55 – 3.49 (m, 2H), 3.47 – 3.42 (m, 1H), 3.31 – 3.27 (m, 1H, H-2), 3.04 (t, J = 7.6 Hz, 2H, $-CH_2NH_2$), 1.76 – 1.68 (m, 4H, $OCH_2CH_2CH_2CH_2CH_2NH_2$), 1.56 – 1.46 (m, 2H, $-CH_2CH_2CH_2-$). ^{13}C NMR (175 MHz, D_2O) δ 170.16 (HCO_2H), 102.13 (C-1), 99.97 (C-1), 99.93 (C-1), 99.89 (C-1), 97.48 (C-1), 77.52, 77.09, 76.03, 74.20, 73.51, 73.41, 73.35, 73.15, 72.89, 72.73, 71.73, 71.52, 71.45, 71.26, 71.13, 70.24, 69.86, 69.44, 69.30, 65.45, 60.44, 60.33, 39.38 (CH_2NH_2), 28.16, 26.42, 22.15.; MS ESI+-HRMS m/z $[M+Na]^+$ calcd for $C_{35}H_{64}NO_{26}$ 914.3711, found 914.3700.

5-Amino-pentyl α-D-glucopyranosyl-(1→3)-α-D-glucopyranosyl-(1→3)-α-D-glucopyranosyl-(1→3)-α-D-glucopyranosyl-(1→3)-β-D-glucopyranoside 7

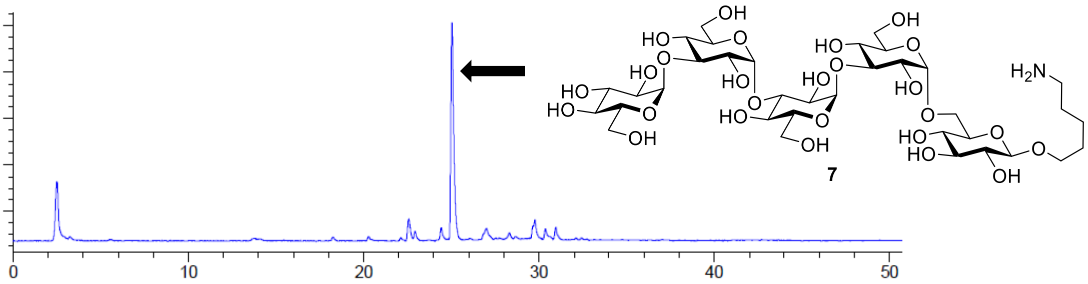

7

LC-MS chromatogram of **7**

^{1}H NMR (700 MHz, D_2O) δ 8.48 (s, 1H, HCO_2H), 5.41 (d, *J* = 3.9 Hz, 1H, H-1), 5.40 (d, *J* = 4.0 Hz, 1H, H-1), 5.38 (d, *J* = 3.9 Hz, 1H, H-1), 4.98 (d, *J* = 3.6 Hz, 1H, H-1), 4.52 (d, *J* = 8.0 Hz, 1H, H-1), 4.08 – 4.03 (m, 3H), 4.01 (dd, *J* = 11.1, 4.3 Hz, 1H), 3.96 – 3.65 (m, 23H), 3.59 (dd, *J* = 9.9, 3.9 Hz, 1H), 3.56 (t, *J* = 9.5 Hz, 1H), 3.51 (t, *J* = 9.2 Hz, 1H), 3.46 (dd, *J* = 10.1, 9.2 Hz, 1H), 3.29 (dd, *J* = 9.3, 8.0 Hz, 1H, H-2), 3.05 – 3.01 (m, 2H, $-CH_2NH_2$), 1.76 – 1.66 (m, 4H, $OCH_2CH_2CH_2CH_2CH_2NH_2$), 1.52 – 1.43 (m, 2H, $-CH_2CH_2CH_2-$). ^{13}C NMR (175 MHz, D_2O) δ 171.04 (HCO_2H), 102.32 (C-1), 99.33 (C-1), 99.18 (2 x C-1), 97.94 (C-1), 79.94, 79.87, 79.48, 76.06, 74.14, 73.13, 72.85, 71.80, 71.68, 71.59, 71.42, 70.33, 70.10, 70.04, 69.85, 69.71, 69.47, 69.28, 65.30, 60.48, 60.27, 60.19, 60.07, 39.35 (CH_2NH_2), 28.17, 26.41, 22.10.; MS ESI+-HRMS m/z $[M+Na]^+$ calcd for $C_{35}H_{64}NO_{26}$ 914.3711, found 914.3719.

5-Amino-pentyl α-D-glucopyranosyl-(1→3)-α-D-glucopyranosyl-(1→6)-α-D-glucopyranosyl-(1→6)-α-D-glucopyranosyl-(1→6)-α-D-glucopyranoside 8

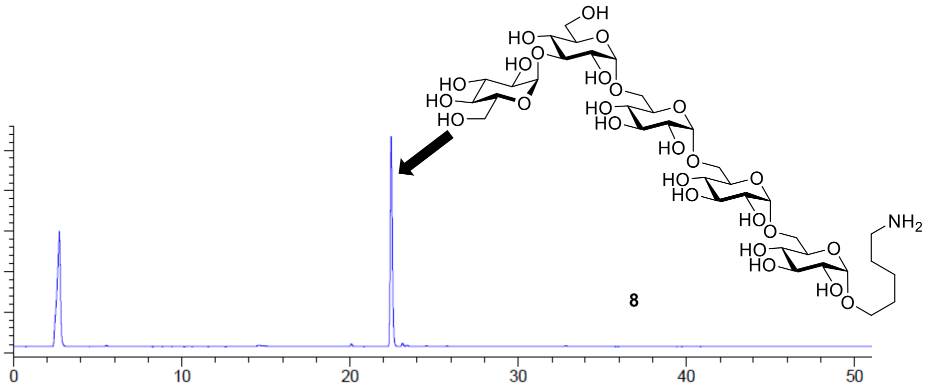

LC-MS chromatogram of **8**

^{1}H NMR (700 MHz, D_2O) δ 8.37 (s, 1H, *H*CO_2H), 5.28 (d, *J* = 3.8 Hz, 1H, H-1), 4.91 (d, *J* = 3.6 Hz, 1H, H-1), 4.90 (d, *J* = 3.6 Hz, 1H, H-1), 4.89 (d, *J* = 3.5 Hz, 1H, H-1), 4.86 (d, *J* = 3.6 Hz, 1H, H-1), 3.97 – 3.88 (m, 4H), 3.87 – 3.83 (m, 2H), 3.80 – 3.57 (m, 17H), 3.53 – 3.42 (m, 8H), 3.38 (t, *J* = 9.6 Hz, 1H), 2.94 (t, *J* = 7.5 Hz, 2H, -CH_2NH_2), 1.67 – 1.59 (m, 4H, $OCH_2CH_2CH_2CH_2CH_2NH_2$), 1.47 – 1.34 (m, 2H, -$CH_2CH_2CH_2$-). ^{13}C NMR (175 MHz, D_2O) δ 169.99 (HCO_2H), 99.14 (C-1), 98.14 (C-1), 97.78 (2 x C-1), 97.72 (C-1), 79.71, 73.45, 73.37, 72.88, 71.67, 71.62, 71.34, 71.18, 70.14, 70.08, 69.93, 69.57, 69.49, 69.35, 67.90, 65.67, 65.58, 65.30, 60.30, 60.24, 39.37 (CH_2NH_2), 28.03, 26.51, 22.44.; MS ESI+-HRMS m/z $[M+Na]^+$ calcd for $C_{35}H_{64}NO_{26}$ 914.3711, found 914.3686.

5-Amino-pentyl α-D-glucopyranosyl-(1→6)-α-D-glucopyranosyl-(1→4)-α-D-glucopyranosyl-(1→4)-α-D-glucopyranoside 9

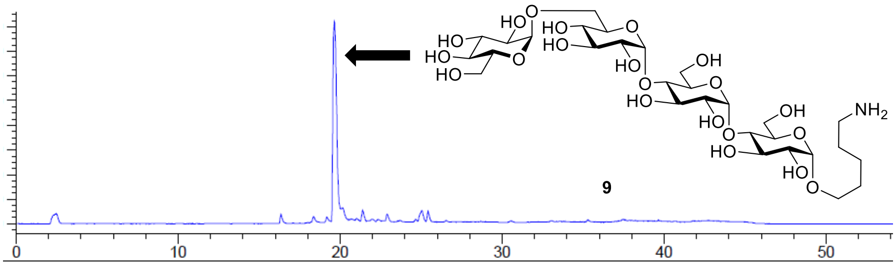

LC-MS chromatogram of **9**

^{1}H NMR (700 MHz, D_2O) δ 8.60 (s, 1H, *H*CO_2H), 5.33 (d, *J* = 4.1 Hz, 1H, H-1), 5.32 (d, *J* = 4.0 Hz, 1H, H-1), 4.88 (d, *J* = 3.7 Hz, 1H, H-1), 4.84 (d, *J* = 3.8 Hz, 1H, H-1), 3.93 – 3.41 (m, 25H), 3.38 – 3.34 (m, 1H), 2.94 (t, *J* = 7.6 Hz, 2H, -CH_2NH_2), 1.66 – 1.56 (m, 4H, $OCH_2CH_2CH_2CH_2CH_2NH_2$), 1.45 – 1.34 (m, 2H, -$CH_2CH_2CH_2$-). ^{13}C NMR (175 MHz, D_2O) δ 99.86 (C-1), 99.46 (C-1), 98.07 (C-1), 97.90 (C-1), 77.13, 76.99, 73.56, 73.29, 73.06, 71.80, 71.62, 71.43, 71.34, 71.19, 71.08, 70.26, 69.51, 69.37, 67.89, 65.92, 60.54, 60.46, 39.35 (CH_2NH_2), 28.03, 26.50, 22.39.; MS ESI+-HRMS m/z $[M+H]^+$ calcd for $C_{29}H_{54}NO_{21}$ 752.3183, found 752.3171.

Supplementary References

1. Hoffman, J., Hahm, H. S., Seeberger, P. H. & Pagel, K. Identification of Carbohydrate Anomers Using Ion Mobility-Mass Spectrometry, Nature, **526**, 241-244 (2015)

2. Eller, S., Collot, M., Yin, J., Hahm, H. S. & Seeberger, P. H. Automated solid-phase synthesis of chondroitin sulfate glycosaminoglycans. *Angew. Chem. Int. Ed.* **52**, 5858-5861 (2013).

3. Kreock, L., Esposito, D., Castagner, B., Wang, C.-C., Bindscheadler, P. & Seeberger P. H. Streamlined access to conjugation-ready glycans by automated synthesis. *Chem. Sci.,* **3**, 1617-1622 (2012).

4. Lourenco, E. C. & Ventura, M. R. The synthesis of compatible solute analogues—solvent effects on selective glycosylation. *Carbohydr. Res.,* **346** 163-168 (2011).

5. Daragics, K. & Feufedi, P. Regio- and chemoselective reductive cleavage of 4,6-O-benzylidene-type acetals of hexopyranosides using BH3·THF–TMSOTf. *Tetrahedron Lett.* **50**, 2914-2916 (2009).

6. Demchenko, A. V., Rousson, E., & Boons, G.-J. Stereoselective 1,2-cis-galactosylation assisted by remote neighboring groups participation and solvent effects. *Tetrahedron Lett.* **40**, 6523-6526 (1999).

7. Liu, L., Bytheway, I., Karoli, T., Fairweather, J. F., Cochran, S., Li, C. & Ferro, V. Design, synthesis, FGF-1 binding, and molecular modeling studies of conformationally flexible heparin mimetic disaccharides. *Bioorg. Med. Chem. Lett.* **18**, 344-364 (2008)

8. Werz, D. B., Carstagner, B. & Seeberger, P. H. Automated Synthesis of the Tumor-Associated Carbohydrate Antigens Gb-3 and Globo-H: Incorporation of α-Galactosidic Linkages. *J. Am. Chem. Soc*. **129**, 2770-2771 (2007).

9. Thijssen, M.-J. L. M. Halkens, K. Kamerling, J. P. & Vliegenthart, J. K. G. Synthesis of a spacer-containing tetrasaccharide representing a repeating unit of the capsular polysaccharide of Streptococcus pneumoniae type 6B. *Bioorg. Med. Chem.*, **2**, 1309-1318 (1994).

10. Kiyoi, T. Nakai, Y. Kondo, H. Ishida, H. Kiso M. & Hasegawa, A. A highly practical synthesis of the sialyl Lewis X pentasaccharide and an investigation of binding to E-, P-, and L-Selectins. *Bioorg. Med. Chem.* **4**, 1167-1176 (1996).
